# Effect of α-Substitution on the Reactivity of C(sp^3^)–H Bonds in Pd^0^-Catalyzed C–H Arylation

**DOI:** 10.1021/acscatal.3c03806

**Published:** 2023-09-11

**Authors:** Matthew Wheatley, Marco Zuccarello, Maria Tsitopoulou, Stuart A. Macgregor, Olivier Baudoin

**Affiliations:** † Department of Chemistry, University of Basel, 4056 Basel, Switzerland; ‡ Institute of Chemical Sciences, Heriot-Watt University, Edinburgh EH14 4AS, U.K.

**Keywords:** C−H activation, DFT, kinetics, palladium, reaction mechanism, reactivity series, relative rates

## Abstract

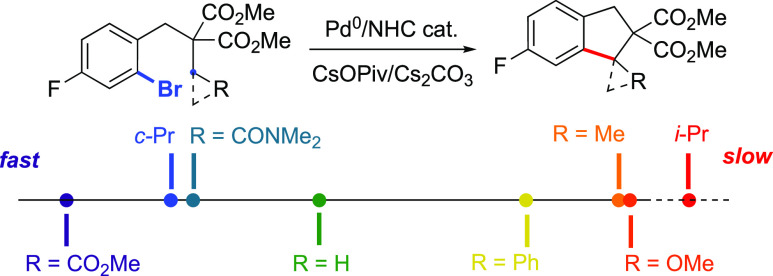

We report mechanistic studies on the reactivity of different α-substituted C(sp^3^)–H bonds, −CH_*n*_R (R = H, Me, CO_2_Me, CONMe_2_, OMe, and Ph, as well as the cyclopropyl and isopropyl derivatives −CH(CH_2_)_2_ and −CHMe_2_) in the context of Pd^0^-catalyzed C(sp^3^)–H arylation. Primary kinetic isotope effects, *k*_H_/*k*_D_, were determined experimentally for R = H (3.2) and Me (3.5), and these, along with the determination of reaction orders and computational studies, indicate rate-limiting C–H activation for all substituents except when R = CO_2_Me. This last result was confirmed experimentally (*k*_H_/*k*_D_ ∼ 1). A reactivity scale for C(sp^3^)–H activation was then determined: C*H*_2_CO_2_Me > C*H*(CH_2_)_2_ ≥ C*H*_2_CONMe_2_ > C*H*_3_ ≫ C*H*_2_Ph > C*H*_2_Me > C*H*_2_OMe ≫ C*H*Me_2_. C–H activation involves AMLA/CMD transition states featuring intramolecular O → H–C H-bonding assisted by C–H → Pd agostic bonding. The “AMLA coefficient”, χ, is introduced to quantify the energies associated with these interactions via natural bond orbital 2nd order perturbation theory analysis. Higher barriers correlate with lower χ values, which in turn signal a greater agostic interaction in the transition state. We believe that this reactivity scale and the underlying factors that determine this will be of use for future studies in transition-metal-catalyzed C(sp^3^)–H activation proceeding via the AMLA/CMD mechanism.

## Introduction

Mechanistic studies have guided reaction development in C–H activation, leading to more efficient and applicable procedures while elucidating previously unknown features of the reaction mechanism.^1^ To this end, the use of computational tools such as density functional theory (DFT) has played an increasingly important role in the study of reaction mechanisms, guiding experimental setup and providing mechanistic insights that would be challenging or impossible through experimentation alone.^2^

**Scheme 1:**
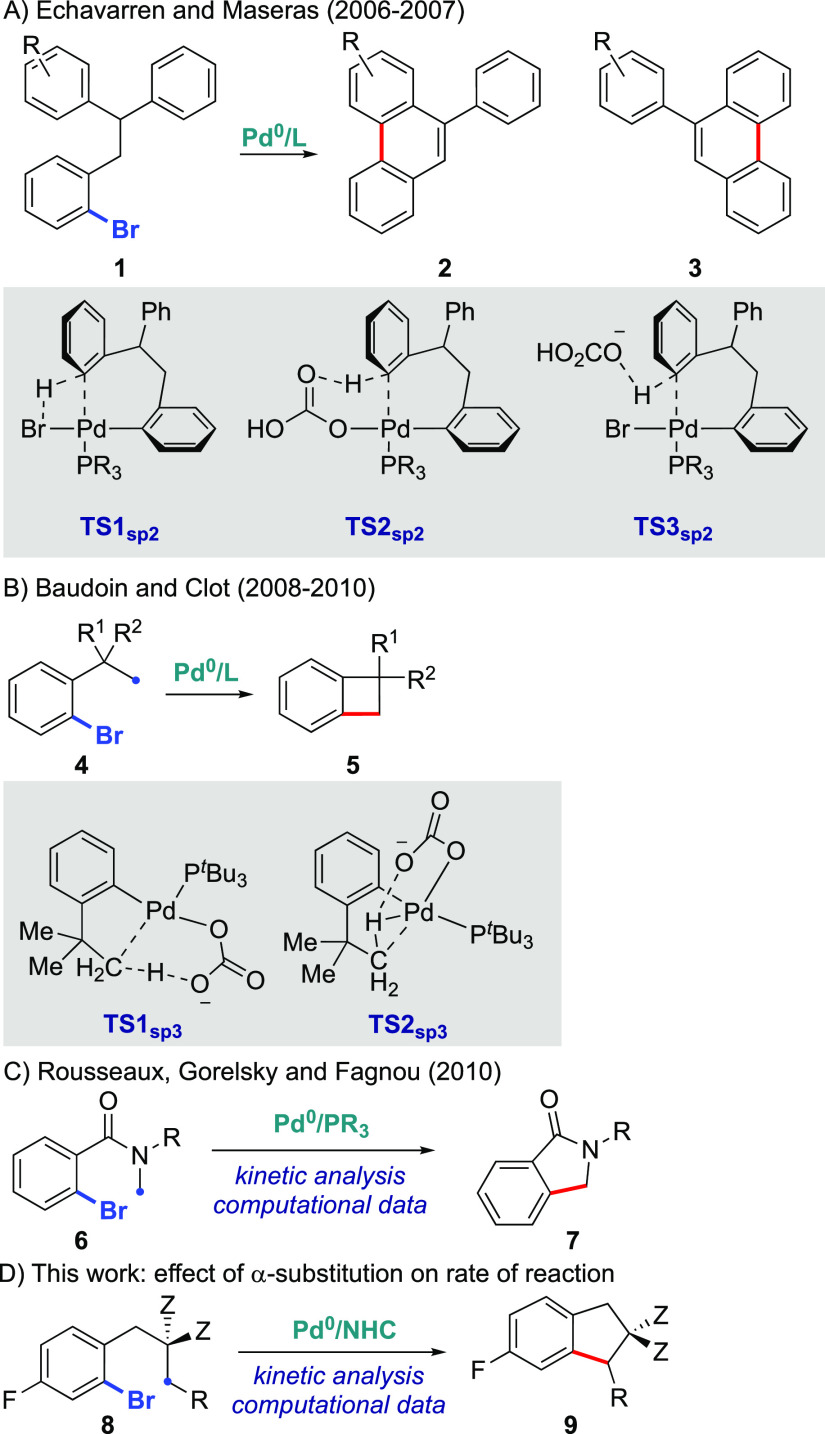
Mechanistic Studies on Pd^0^-Catalyzed C–H Activation: (A–C) Previous Studies and (D) Current Study

The use of Pd^0^-catalyzed C(sp^2^/sp^3^)–H activation in organic synthesis has ascended to the level of a valuable synthetic strategy since the turn of the century and now constitutes a reliable method for the construction of valuable compounds from simple (pseudo)halide starting materials.^3^ In 2006, Echavarren and Maseras reported the synthesis of fused rings by C(sp^2^)–H arylation (Scheme 1a).^4^ In this study, it was shown that the reaction of substituted aryl bromides **1** could lead to the formation of isomeric products **2** and **3** depending on the nature of the R group. In particular, it was shown that when R was electron-withdrawing, arylation took place preferentially on the substituted ring due to electronically favored C–H activation on this ring. DFT calculations suggested that direct proton transfer to the bromide ligand (**TS1**_**sp2**_) is unlikely due to the high computed energy barrier of 43.3 kcal mol^–1^. Thus, the proton abstraction was proposed to occur via an intramolecular (**TS2**_**sp2**_) or an intermolecular (**TS3**_**sp2**_) base-assisted mechanism, depending on the electronic properties of the ortho-substituent to the activated C–H bond. This mechanism was later termed concerted metalation-deprotonation (CMD) or ambiphilic metal–ligand activation (AMLA).^5^ In 2008, our group contributed to mechanistic studies of Pd^0^-catalyzed C(sp^3^)–H activation in the formation of benzocyclobutenes **5** (Scheme 1b).^6^ It was found that C–H activation was the rate-limiting step and was proposed to proceed via a carbonate-assisted AMLA/CMD mechanism. Computational studies showed that two transition states are energetically accessible, with a *cis* (**TS1**_**sp3**_) or *trans* (**TS2**_**sp3**_) orientation of the carbonate base relative to the activated C–H bond, with the *trans* geometry being favored with the considered substrate/ligand/base combination.^7^ In 2010, Fagnou and co-workers reported mechanistic studies on the Pd-catalyzed C(sp^3^)–H arylation of aryl bromides to form lactams (**7**) and cyclic sulfonamides (Scheme 1c).^8^ Detailed kinetic analysis revealed a rapid oxidative addition followed by a rate-limiting C–H activation, supported by a significant primary kinetic isotope effect (KIE). The authors noted that both pivalate and carbonate bases were required in the reaction on the basis of stoichiometric studies of the corresponding Pd^II^ oxidative addition complexes, which they attributed to a reversible CMD with pivalate and an irreversible deprotonation of the formed Pd-bound pivalic acid by carbonate. Computational studies supported the proposed C–H activation proceeding through a CMD mechanism.

The electronic effects of substitution on the arene ring are well understood for C(sp^2^)–H activation. Indeed, Hammett plots constitute a reliable tool to quantify these effects and, in some cases, predict the selectivity of reactions.^9^ However, the influence of α-substitution on C(sp^3^)–H bond activation remains unexplored in this context.^10^ This represents a significant issue in the field, as practitioners studying this class of reaction have to rely on chemical intuition rather than the use of accurate data. This lack of understanding in the field of Pd^0^-catalyzed C(sp^3^)–H activation could be due to the significant challenge associated with the activation of methylene C–H bonds. Indeed, previous examples were limited to *gem*-dialkyl groups^11^ or benzylic secondary positions,^12^ which precluded comparative studies of reactivity on a broad range of α-substituents. Recently, however, our group reported an extremely active Pd/NHC system that allowed for the arylation of nonactivated secondary C–H bonds.^13^ In this reaction, the IBiox-type NHC ligand was essential for the high reactivity and enantioselectivity observed, presumably due to its rigid bisoxazoline scaffold and strong electron-donating properties compared to phosphine ligands.^14^ Unlocking this reactivity has thus opened the door to a quantitative study of substituent effects on the reactivity of secondary C–H bonds.

We report herein the construction of a reactivity scale which allows the first quantitative analysis of the effect of α-substituents on the rate of activation of C(sp^3^)–H bonds using a Pd^0^/NHC catalytic system (Scheme 1d). This study, combining experimental and computational methods, shines a light on key factors affecting the differences in observed reactivity between different types of C(sp^3^)–H bonds.

## Results and Discussion

### Kinetic Studies

**Scheme 2:**
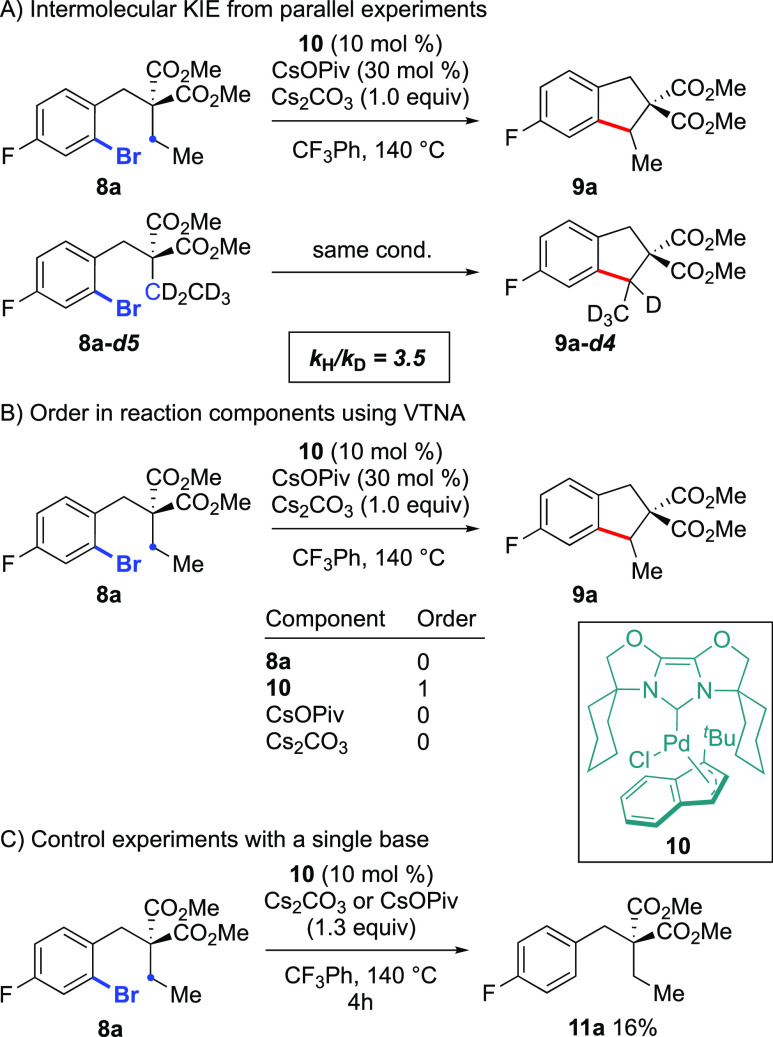
Kinetic Studies: (A) KIE; (B) Kinetic Data Using VTNA; (C) Control Experiments

At the onset of this study, a practical challenge was the observation of a significant induction period, which we ascribed to the slow activation of the employed [Pd^II^(NHC)(η^3^-allyl)Cl] precatalyst to form the active Pd^0^ species.^13a^ We first sought to suppress this induction period, which would be detrimental to obtaining reliable kinetic data. Gratifyingly, we found that complex **10** (Scheme 2), which contains a bulky η^3^-1-*t*Bu-indenyl ancillary ligand that, according to Nova, Hazari, and co-workers,^15^ avoids the formation of off-cycle Pd^I^ species, successfully realized this task. The spirocyclic IBiox6 ligand, initially developed by Glorius and co-workers,^16^ already proved optimal for the arylation of secondary C–H bonds in racemic mode,^13a^ and it was therefore retained for this study.

In order to examine the relative reactivity of the current system effectively and correlate differences between distinct C–H bonds, we first needed to ensure that the C–H activation was the rate-limiting step, as had been previously reported by our group and Fagnou for primary C–H bonds and Pd/phosphine catalysts.^8a^ To this end, we examined the deuterium KIE in parallel experiments (Figure S5). Our observed KIE of 3.5 strongly suggests that the C–H activation is the rate-limiting step for this process (Scheme 2a).^17^ Furthermore, this experimental KIE is in excellent agreement with the calculated value (vide infra). This confirmed that the different rates displayed for different substrates would reflect the differences between C–H bonds during the C–H activation step, as intended in this study.

In order to further characterize the reaction mechanism experimentally with the current substrate/catalyst combination prior to DFT studies, the orders in reactants were first obtained using the VTNA method developed by Burés (Scheme 2b and Figures S1–S4).^18^ The data obtained were found to be broadly in agreement with what was disclosed by Fagnou and co-workers with primary C–H bonds and the Pd/PCy_3_ catalyst.^8a^ Zero order was observed for the aryl bromide substrate **8a**, which is consistent with a fast and irreversible oxidative addition taking place. The reaction was also determined to be first order with respect to catalyst **10**, as expected for catalysis by a mononuclear Pd complex. Interestingly, unlike what was reported by Fagnou and co-workers for the Pd/PCy_3_ system, we observed zero order with respect to the concentration of the pivalate additive. However, upon examining the solubility of CsOPiv in trifluorotoluene at 140 °C, it was found that even in the lowest concentration studied, this base was insoluble, meaning that obtaining meaningful kinetic data on this species is a significant challenge. This was also the case with carbonate, which is sparingly soluble in the reaction solvent. Interestingly, the reaction does not proceed in the absence of pivalate or carbonate (Scheme 2c and Figures S11–S12), with both being required in order for product formation to occur. In the absence of either of these bases, only the corresponding protodehalogenation product **11a** was detected. Due to the heterogeneous nature of these transformations, the effect of stirring on the rate of reaction was examined (Figure S13). When the stirring rate was low (250 rpm), the reaction did not proceed, with only trace amounts of product formation being observed. This is consistent with a species that is not entirely soluble in the reaction media being involved in a kinetically relevant step in the reaction, as with a higher stirring rate, there is a higher concentration of this species in solution.^19^ Finally, we hypothesized that the activation of the Pd^II^ precatalyst **10** to generate the active Pd^0^-NHC species was mediated by CsOPiv.^15a^ Interestingly, upon heating the precatalyst with this additive, the rapid formation of an unexpected bis-indene cross-coupling product was observed, indicating an unusual catalyst activation mode (Figure S8). Moreover, when the corresponding experiment was performed with carbonate as an additive, this reaction was slowed down (Figure S9). These data correspond to pivalate playing a major role in the activation of the palladium catalyst; however, when taken together with the previous observations on kinetic orders, it does not rule out pivalate also being involved in the subsequent C–H functionalization.

**Scheme 3:**
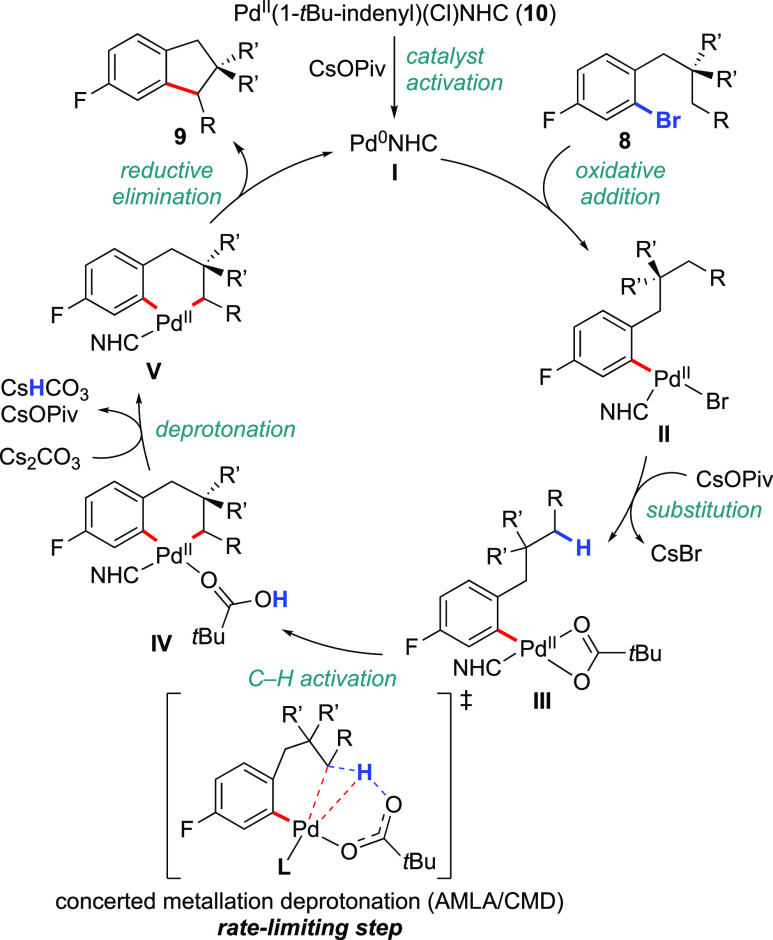
Proposed Catalytic Cycle

Based on our experimental observations supported by DFT calculations (vide infra), we propose the following catalytic cycle (Scheme 3). Complex **10** undergoes activation to generate the required Pd^0^ catalyst, **I**. The latter undergoes rapid and irreversible oxidative addition with aryl bromide **8** to generate complex **II**. Subsequent ligand exchange with pivalate to form **III** takes place. The activation of the secondary C–H bond occurs via AMLA/CMD,^5^ followed by exergonic deprotonation of the Pd-bound pivalic acid **IV** by carbonate. This dual-base activation mode is supported by the fact that both bases are experimentally required (Scheme 2c). The C–H activation step is rate-limiting, consistent with the measured primary KIE of 3.5 (Scheme 2a). Finally, reductive elimination from palladacycle **V** leads to the indane product **9** and restores the active Pd^0^ species.

**Figure 1:**
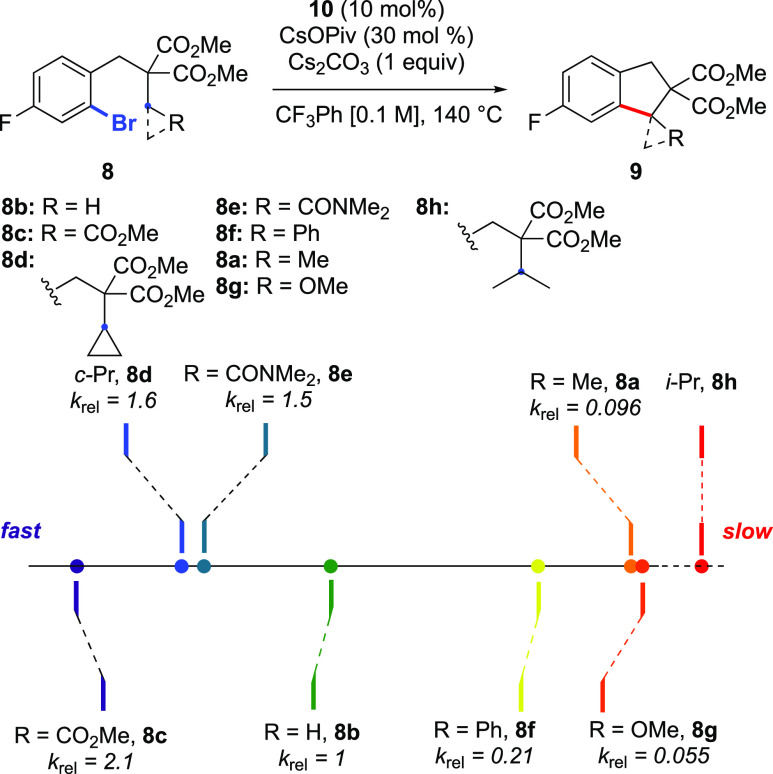
Initial rate experiments to determine relative rate constants *k*_rel_.

Following this preliminary study, we turned our attention to the relative rates of compounds with different α-substituents. It is important to note that the exo-position of the R substituent in substrate **8** relative to the activated C–H bond is required for this study, as a modification of the endo α-position would lead to conformational and reactivity biases being introduced. A *gem*-diester group on this endo-position facilitates the substrate synthesis, prevents competitive C–H activation, and exerts a Thorpe-Ingold effect, which allows a broader range of exo-substituents to be tested. We selected R = H (**8b**) as the “neutral” substituent in analogy to the Hammett plots, and as such, this relative rate was set to 1.0. All subsequent rates for the various R groups are compared to this substrate (Figure 1). The most acidic substrate with respect to the activated C–H bonds (**8c**, R = CO_2_Me) displayed the fastest rate, with a reaction that was 2.1 times faster than the standard reaction. At the other end of the scale, when R = OMe (**8g**), the rate was observed to be much slower than the standard reaction. Cyclopropyl-containing substrate **8d** underwent C–H activation with a relative rate of 1.6. Consistent with previous work,^20^ this reaction formed the spirocyclic product **9d**, meaning that the C–H activation at the tertiary position was favored due to the preferential formation of a 6-membered palladacycle over a larger ring. This high rate presumably is due to the increased sp^2^ character associated with cyclopropane rings.^21^ Substrate **8e**, containing an electron-withdrawing *N,N*-dimethylamide group, reacted 1.5 times faster than the standard substrate. The difference in reactivity between **8c** and **8e** is consistent with the decreased electron-withdrawing ability of amides compared to esters, meaning that the activated C–H bond is less acidic in this case. Although the p*K*_a_ value of a methyl proton is higher than that of a benzylic proton, **8b** is significantly faster than **8f** in this reaction. When comparing these two R groups, it is clear that the methyl group is significantly smaller near the C–H bond being activated. Interestingly, a methyl group (**8b**) reacts ca. 10× faster than an ethyl group (**8a**). This is consistent with the wealth of empirical data accumulated in this field in the past two decades by our group and others,^3d,8,11,12,13a^ but the current study now allows a precise figure to be put on this well-known reactivity difference between primary and secondary C–H bonds. Due to this significant difference in reactivity between the methyl and ethyl groups, we again examined the KIE on the former to see if there was a different value with a reaction that was significantly faster. We observed a *k*_H_/*k*_D_ of 3.2 for this CH_3_/CD_3_ system (Figure S6), suggesting that the C–H activation is still the rate-limiting step for this substrate.

Finally, substrate **8h** bearing an isopropyl group was also tested, but no sign of C–H activation at the tertiary C–H bond was detected. Instead, the 6-membered ring product arising from C–H arylation at one of the terminal methyl groups was mainly observed (48% NMR yield), together with the protodehalogenated product (35%). This further confirms previous observations that nonbiased tertiary C–H bonds do not readily undergo C–H activation in such transformations. Although a relative rate cannot be measured for this case, we propose to position it at the extreme right of the reactivity scale on the basis of the calculated C–H activation barrier (vide infra).

This study leads to the following overall order based on the measured relative reaction rates
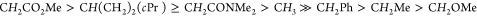


### Computational Studies

In order to rationalize the observed differences in reactivity for these substrates, we turned to DFT calculations (see Supporting Information for details). We take Pd(NHC), **I**, as the active species (cf. Scheme 2), and the initial C–Br oxidative addition at this species was assessed for substrate **8b** (Figure S14). This proceeds with a barrier of only 6.4 kcal/mol to access T-shaped Pd(NHC)(Ar^H^)Br, **II**^**H**^ (the superscript will indicate the α-substituent), the most stable isomer of which lies at −27.7 kcal/mol. Br/OPiv substitution then gives **III**^**H**^ at −34.7 kcal/mol with a κ^2^-OPiv ligand. This facile oxidative addition process is consistent with the observed zero-order kinetics in [ArBr]. **III**^**H**^ is the rate-limiting intermediate for the subsequent C–H functionalization catalysis, and so in the following, all free energies will be quoted relative to this species, set to 0.0 kcal/mol.

**Figure 2:**
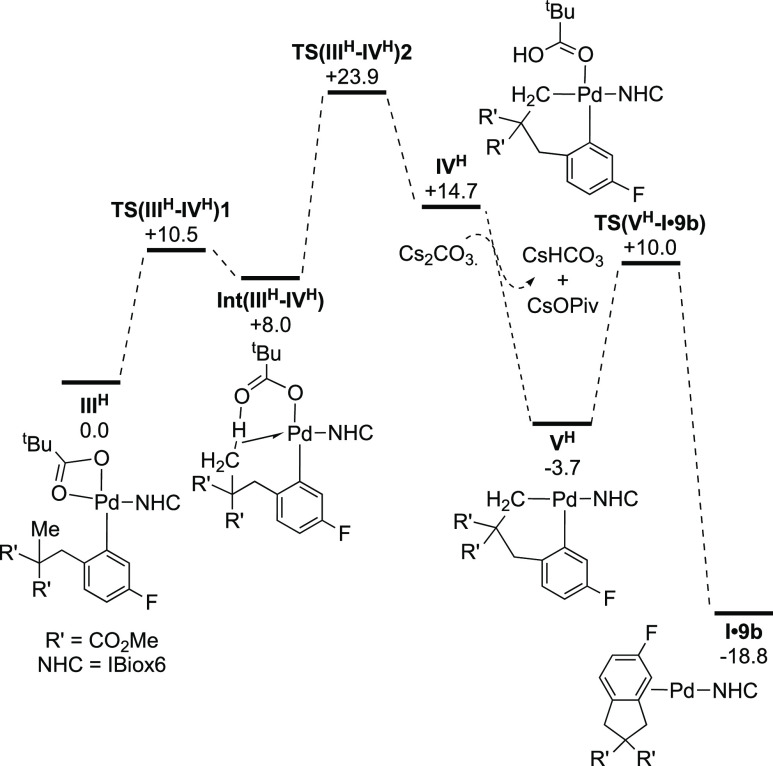
Computed free energy reaction profile (kcal/mol at 413 K) for the C–H cyclization of **8b** starting from intermediate **III**^**H**^. Level of theory: B97D(def2tzvp,1,2-C_6_H_4_Cl_2_)//BP86(SDD, 6-31G**). 1,2-C_6_H_4_Cl_2_ (ε = 9.99) is used as a substitute for CF_3_Ph (ε = 9.18), as parameters for the latter are not available.

The computed catalytic cycle starting from **III**^**H**^ is shown in Figure 2. C–H activation proceeds in a 2-step process via an agostic intermediate, **Int(III**^**H**^**–IV**^**H**^**)**, formed via the κ^2^**–**κ^1^-displacement of one arm of the OPiv ligand. This sets up the system for an AMLA/CMD C–H activation via **TS(III**^**H**^**–IV**^**H**^**)2** at +23.9 kcal/mol and forms the cyclometalated intermediate **IV**^**H**^ at +14.7 kcal/mol. At this point, following Fagnou,^8a^ we consider HOPiv to dissociate from **IV**^**H**^ with H^+^ transfer to carbonate, where any anions present were modeled as ion pairs with Cs^+^ counterions (see Scheme S1 for model testing). This exergonic step gives the 3-coordinate intermediate **V**^**H**^ at −3.7 kcal/mol from which C–C coupling proceeds via **TS(V**^**H**^**–I·9b)** at +10.0 kcal/mol. This initially leads to **I·9b** in which the indane product forms a π-complex with the Pd(NHC) fragment. Catalysis is therefore computed to be strongly exergonic and proceeds with an overall barrier of 23.9 kcal/mol via **TS(III**^**H**^**–IV**^**H**^**)2**, implying rate-limiting C–H activation. A computed KIE using **III**^**H**^**-*d3*** gave a value of 3.6, in good agreement with the experimental value of 3.2.

This reaction profile was recomputed for R = Me, CO_2_Me, C(CH_2_)_2_, Ph, and OMe (Figures S15–S20), and in all cases, the mechanism outlined in Figure 2 was followed. With one exception (R = CO_2_Me, vide infra), the overall energy span for the cyclization process corresponds to C–H bond cleavage via **TS(III**^**R**^**–IV**^**R**^**)2** and a computed KIE when R = Me returned a value of 3.5, in excellent agreement with the experiment. Significant variations in the barriers to C–H activation were also seen (Δ*G*^⧧^_CHA_, Table 1), and the computed trend follows that of the relative rates in Figure 1, with the exception of the anomalously high value when R = Ph. This outcome was independent of functional choice (Figures S22–S23),^22^ and these tests also showed some variation in the relative positioning for the cyclopropyl group. For the remaining substituents, the trend in Δ*G*^⧧^_CHA_ (R = CO_2_Me ≪ H < Me < OMe) was robust across all functionals, so our initial analyses focused on these cases.

**Table 1:** Computed Overall Barriers (kcal/mol) for C–H Activation as a Function of Substituent, R

C*H*_*n*_R	C*H*_2_CO_2_Me	C*H*(CH_2_)_2_	C*H*_3_
Δ*G*^⧧^_CHA_	19.1	23.5	23.9
C*H*_*n*_R	C*H*_2_Ph	C*H*_2_Me	C*H*_2_OMe
Δ*G*^⧧^_CHA_	27.0	25.4	27.7

**Figure 3:**
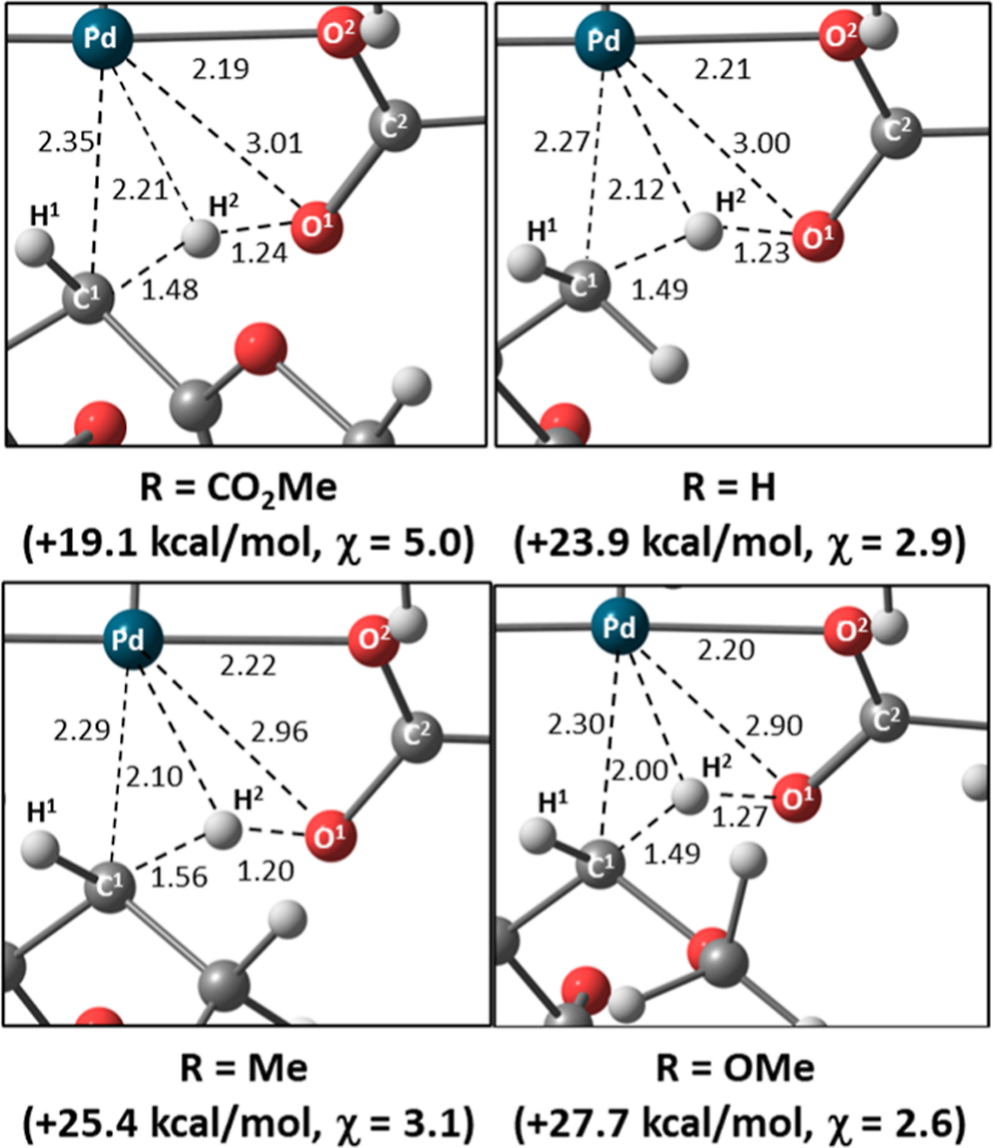
Details of the computed geometries of **TS(III**^**R**^**–IV**^**R**^**)2** for R = CO_2_Me, H, Me, and OMe, with selected distances in Å and relative free energies indicated in kcal/mol. χ is the AMLA coefficient (see text for details).

Details of the computed C–H activation transition states, **TS(III**^**R**^**–IV**^**R**^**)2**, for R = CO_2_Me, H, Me, and OMe are shown in Figure 3. In all cases, the transferring hydrogen, H^2^, shows short contacts with both the Pd metal center and the pendent oxygen, O^1^, of the κ^1^-pivalate base, consistent with the synergic combination of C^1^–H^2^ → Pd agostic and O^1^ → H^2^–C^1^ H-bonding interactions that facilitate C^1^–H^2^ bond cleavage.^5c^ Within this series, an increased barrier is associated with a shorter Pd···H^2^ contact, with this distance decreasing from 2.21 Å for R = CO_2_Me to 2.00 Å for R = OMe. Similar trends are also seen in the agostic intermediate **Int(III**^**R**^**–IV**^**R**^**)** with H-bonding being more significant for R = CO_2_Me and agostic bonding being more prominent for R = OMe (Figure S25).

The relative energies associated with these donor–acceptor interactions were quantified through NBO 2nd order perturbation analyses on the **TS(III**^**R**^**–IV**^**R**^**)2** structures. First, these indicated that O^1^ → H^2^–C^1^ H-bonding is a more significant component than the C^1^–H^2^ → Pd agostic interaction (Table S1). Moreover, increased barriers are associated with a greater relative contribution from the agostic interaction. To quantify this, we introduce the “AMLA coefficient”, χ, the ratio of the O^1^ → H^2^–C^1^ donation to the C^1^–H^2^ → Pd agostic interaction, as defined via NBO 2nd order perturbation analyses. χ is highest for R = CO_2_Me (5.0) and lowest for R = OMe (2.6), and a plot of Δ*G*^⧧^ vs χ provides a straight line with a reasonable correlation coefficient, *R*^2^, of 0.91 (Figure S27). C–H activation is therefore characterized as an intramolecular deprotonation that is assisted by an agostic interaction with the Pd center.

Given the above, enhanced reactivity is seen with more acidic bonds (i.e., R = CO_2_Me) where a reduced contribution from the agostic interaction is necessary to polarize the C–H bond. Consistent with this, the average computed NBO charge at the C*H*_*2*_R methylene hydrogens in **III**^**R**^ is the highest for R = CO_2_Me (+0.300), intermediate for R = H and Me (+0.272 and +0.277, respectively), and the lowest for R = OMe (+0.241). In this last case, delocalization of the *O*Me lone pair into the C^1^–H^2^ σ* orbital may account for the lower charge (quantified via the 2nd order perturbation analysis at 6.0 kcal/mol). In general, as the C–H activation proceeds, the computed charge at the transferring hydrogen (H^2^) increases in first **Int(III**^**R**^**–IV**^**R**^**)** and then **TS(III**^**R**^**–IV**^**R**^**)2** (Table S2). The only exception is a reduction in charge in **Int(III**^**R**^**–IV**^**R**^**)** when R = OMe (+0.191), and this matches both an increased O_LP_ → C^1^–H^2^ σ* donation (8.5 kcal/mol) and a shortening of the CH_2_–OMe distance, from 1.43 Å in **III**^**R**^ to 1.41 Å in **Int(III**^**R**^**–IV**^**R**^**)**. This less electron-deficient C–H bond therefore requires greater interaction with the Pd center for activation to occur, and this results in an increased barrier. For the R = H vs Me comparison, both the computed charge at C*H*_*2*_R and the χ value are slightly higher when R = Me, and these are both contrary to the higher barrier (and lower observed rate) in that case. We speculate that steric effects may be important here, and while this is difficult to quantify, additional calculations on the *i*Pr analogue (i.e., C–H activation of a −C*H*Me_2_ group) gave a significantly higher barrier of 37.2 kcal/mol. This value is in agreement with the observed lack of C–H arylation at the tertiary C–H bond of substrate **8h** (vide supra). With the cyclopropyl group, the computed Pd···H^2^ distance in **TS(III**^**R**^**–IV**^**R**^**)2** is the longest of the systems studied here (2.28 Å), although the computed value of χ = 3.9 does correctly place it between R = CO_2_Me and R = H. This relatively high χ value may reflect the greater C s-character in the cyclopropyl C^1^–H^2^ bond as well as being consistent with a relatively high computed charge on C*H* in **III**^**R**^ (+0.293, see Table S2).

Returning to the experiment, we noted above that for R = CO_2_Me (**8c**), the identity of the rate-limiting transition is less clear-cut: the energy span for C–H activation via **TS(III**^**R**^**–IV**^**R**^**)2** is only 19.1 kcal/mol while that for C–C coupling via **TS(V**^**R**^**–VI**^**R**^**)** is the highest of those systems studied here at 18.5 kcal/mol; this balance is also functionally dependent (see Scheme S2). Previous experimental^23^ and computational^24^ studies have shown electron-withdrawing substituents tend to increase the barrier to reductive elimination. In the present system, this implies a potential change in the rate-determining process, and this was investigated experimentally. A *k*_H_/*k*_D_ of ∼1 was indeed obtained (Figure S7), which supports the suggestion from the calculations that, when R = CO_2_Me, C–H activation is not rate-limiting.

## Conclusions

We have studied the effect of α-substitution on the alkyl fragment in ring-forming Pd^0^-catalyzed C(sp^3^)–H arylation for various −CH_*n*_R groups. To this end, we have developed a reactivity scale, which, for the first time, places substituents in a series of most to least reactive: C*H*_2_CO_2_Me > C*H*(CH_2_)_2_ ≥ C*H*_2_CONMe_2_ > C*H*_3_ ≫ C*H*_2_Ph > C*H*_2_Me > C*H*_2_OMe ≫ C*H*Me_2_. Furthermore, kinetic analysis and parallel computational studies suggest that the C–H activation is the rate-limiting step in most cases, with significant primary KIE values being recorded for two of the substrates (R = H and Me). A notable exception was observed when the substrate bearing the most acidic C–H bonds adjacent to an ester group was used, with a *k*_H_/*k*_D_ ∼1 being recorded. This is consistent with the wealth of empirical observations in the field that the acidity of the C–H bond being activated is a crucial factor in determining the rate of the reaction. NBO analyses characterize C–H activation as an intramolecular deprotonation assisted by agostic bonding at the Pd^2+^ center. This is quantified by the AMLA coefficient, χ, the ratio of the energies associated with O → H–C donation and C–H → Pd agostic interaction. Higher barriers are associated with a greater agostic interaction in the transition state (a lower χ) and a correlation with the computed charge at the reacting H atom is also seen. Future studies will assess the utility of these descriptors in understanding and predicting the reactivity of C(sp^3^)–H bonds bearing diverse α-substituents in other reactions proceeding via the AMLA/CMD mechanism.

## Supporting Information Available

General methods, optimization studies, catalyst preparation, substrate synthesis, product synthesis, kinetic experiments, kinetic analysis, spectra, computational details, and computed results (PDF)
Coordinates for DFT-optimized structures (XYZ)

## Supplementary Material

cs3c03806_si_001.pdf

cs3c03806_si_002.xyz

## References

[ref1] aSun H. Y.; Gorelsky S. I.; Stuart D. R.; Campeau L. C.; Fagnou K. Mechanistic Analysis of Azine N-Oxide Direct Arylation: Evidence for a Critical Role of Acetate in the Pd(OAc)_2_ Precatalyst. J. Org. Chem. 2010, 75, 8180–8189. 10.1021/jo101821r. 21053903

[ref2] aGorelsky S. I.; Lapointe D.; Fagnou K. Analysis of the concerted metalation-deprotonation mechanism in palladium-catalyzed direct arylation across a broad range of aromatic substrates. J. Am. Chem. Soc. 2008, 130, 10848–10849. 10.1021/ja802533u. 18661978

[ref3] aCampeau L.-C.; Fagnou K. Palladium-Catalyzed Direct Arylation of Simple Arenes in Synthesis of Biaryl Molecules. Chem. Commun. 2006, 1253–1264. 10.1039/B515481M. 16538241

[ref4] aGarcía-Cuadrado D.; Braga A. A. C.; Maseras F.; Echavarren A. M. Proton Abstraction Mechanism for the Palladium-Catalyzed Intramolecular Arylation. J. Am. Chem. Soc. 2006, 128, 1066–1067. 10.1021/ja056165v. 16433509

[ref5] aLapointe D.; Fagnou K. Overview of the Mechanistic Work on the Concerted MetallationDeprotonation Pathway. Chem. Lett. 2010, 39, 1118–1126. 10.1246/cl.2010.1118.

[ref6] Chaumontet M.; Piccardi R.; Audic N.; Hitce J.; Peglion J.-L.; Clot E.; Baudoin O. Synthesis of Benzocyclobutenes by Palladium-Catalyzed C–H Activation of Methyl Groups: Method and Mechanistic Study. J. Am. Chem. Soc. 2008, 130, 15157–15166. 10.1021/ja805598s. 18928284

[ref7] Kefalidis C. E.; Baudoin O.; Clot E. DFT Study of the Mechanism of Benzocyclobutene Formation by Palladium-Catalysed C(sp^3^)–H Activation: Role of the Nature of the Base and the Phosphine. Dalton Trans. 2010, 39, 10528–10535. 10.1039/c0dt00578a. 20927457

[ref8] aRousseaux S.; Gorelsky S. I.; Chung B. K. W.; Fagnou K. Investigation of the Mechanism of C(sp^3^)–H bond cleavage in Pd(0)-catalyzed Intramolecular Alkane Arylation Adjacent to Amides and Sulfonamides. J. Am. Chem. Soc. 2010, 132, 10692–10705. For other studies on related reactions: 10.1021/ja103081n. 20681702

[ref9] aHammett L. P. The Effect of Structure upon the Reactions of Organic Compounds. Benzene Derivatives. J. Am. Chem. Soc. 1937, 59, 96–103. 10.1021/ja01280a022.

[ref10] aOlmos A.; Gava R.; Noverges B.; Bellezza D.; Jacob K.; Besora M.; Sameera W. M. C.; Etienne M.; Maseras F.; Asensio G.; Caballero A.; Pérez P. J. Measuring the Relative Reactivity of the Carbon–Hydrogen Bonds of Alkanes as Nucleophiles. Angew. Chem., Int. Ed. 2018, 57, 13848–13852. 10.1002/anie.201807448. 30015368

[ref11] aBaudoin O.; Herrbach A.; Guéritte F. The Palladium-Catalyzed C–H Activation of Benzylic *gem*-Dialkyl Groups. Angew. Chem., Int. Ed. 2003, 42, 5736–5740. 10.1002/anie.200352461. 14661210

[ref12] Pedroni J.; Boghi M.; Saget T.; Cramer N. Access to β-Lactams by Enantioselective Palladium(0)-Catalyzed C(sp^3^)–H Alkylation. Angew. Chem., Int. Ed. 2014, 53, 9064–9067. 10.1002/anie.201405508. 24986088

[ref13] aMelot R.; Zuccarello M.; Cavalli D.; Niggli N.; Devereux M.; Bürgi T.; Baudoin O. Palladium(0)-Catalyzed Enantioselective Intramolecular Arylation of Enantiotopic Secondary C–H Bonds. Angew. Chem., Int. Ed. 2021, 60, 7245–7250. For recent examples of Pd^II^-catalyzed methylene C–H activation: 10.1002/anie.202014605. 33325596

[ref14] aWürtz S.; Glorius F. Surveying Sterically Demanding N-Heterocyclic Carbene Ligands with Restricted Flexibility for Palladium-catalyzed Cross-Coupling Reactions. Acc. Chem. Res. 2008, 41, 1523–1533. 10.1021/ar8000876. 18720995

[ref15] aMelvin P. R.; Nova A.; Balcells D.; Dai W.; Hazari N.; Hruszkewycz D. P.; Shah H. P.; Tudge M. T. Design of a Versatile and Improved Precatalyst Scaffold for Palladium-Catalyzed Cross-Coupling: (η^3^-1-^t^Bu-indenyl)_2_(μ-Cl)_2_Pd_2_. ACS Catal. 2015, 5, 3680–3688. 10.1021/acscatal.5b00878.

[ref16] Altenhoff G.; Goddard R.; Lehmann C. W.; Glorius F. An N-Heterocyclic Carbene Ligand with Flexible Steric Bulk Allows Suzuki Cross-Coupling of Sterically Hindered Aryl Chlorides at Room Temperature. Angew. Chem., Int. Ed. 2003, 42, 3690–3693. 10.1002/anie.200351325. 12916049

[ref17] Simmons E. M.; Hartwig J. F. On the interpretation of Deuterium Kinetic Isotope Effects in C–H Bond Functionalizations by Transition-Metal Complexes. Angew. Chem., Int. Ed. 2012, 51, 3066–3072. 10.1002/anie.201107334. 22392731

[ref18] aBurés J. A. A Simple Graphical Method to Determine the Order in Catalyst. Angew. Chem., Int. Ed. 2016, 55, 2028–2031. 10.1002/anie.201508983. PMC4797368 26749539

[ref19] aPlata R. E.; Hill D. E.; Haines B. E.; Musaev D. G.; Chu L.; Hickey D. P.; Sigman M. S.; Yu J.-Q.; Blackmond D. G. A Role for Pd(IV) in Catalytic Enantioselective C–H Functionalization with Monoprotected Amino Acid Ligands under Mild Conditions. J. Am. Chem. Soc. 2017, 139, 9238–9245. 10.1021/jacs.7b03716. 28605190

[ref20] aLadd C. L.; Sustac Roman D.; Charette A. B. Silver-Promoted, Palladium-Catalyzed Direct Arylation of Cyclopropanes: Facile Access to Spiro 3,3′-Cyclopropyl Oxindoles. Org. Lett. 2013, 15, 1350–1353. 10.1021/ol4003338. 23452033

[ref21] Veillard A.; Re G. Hybridization in Cyclopropane, Cyclobutane and Cubane. Theor. Chim. Acta 1964, 2, 55–62. 10.1007/bf00529465.

[ref22] The possibility that carbonate could be the active base in the C–H activation step was also assessed, but this gave poorer agreement with the experimental trends with, in particular, too large a barrier when R = H and cPr (see Figure S24). If the deprotonation of intermediate **IV^R^** did not occur the subsequent C–C coupling would become rate-limiting. Not only would be inconsistent with the KIEs determined when R = H and Me, but energy spans computed on this basis again provided poor agreement with the experimental rate data (see Figure 1).

[ref23] aCulkin D. A.; Hartwig J. F. Carbon–Carbon Bond-Forming Reductive Elimination from Arylpalladium Complexes Containing Functionalized Alkyl Groups. Influence of Ligand Steric and Electronic Properties on Structure, Stability, and Reactivity. Organometallics 2004, 23, 3398–3416. 10.1021/om049726k.

[ref24] aSakaki S.; Biswas B.; Sugimoto M. A Theoretical Study of the C–H Activation of Methane Derivatives. Significant Effects of Electron-Withdrawing Substituents. Organometallics 1998, 17, 1278–1289. 10.1021/om970705i.

