# Supplementary material for: Effect of α-Substitution on the Reactivity of C(sp3)–H Bonds in Pd0-Catalyzed C–H Arylation
Source: ACS Catal. 2023 Sep 11;13(19):12563–70. doi: 10.1021/acscatal.3c03806 (PMC10563019; doi:10.1021/acscatal.3c03806)
Supplement: Supplementary file 1 — cs3c03806_si_001.pdf [file cs3c03806_si_001.pdf]

The Effect of α-Substitution on the Reactivity of C(sp^3)–H Bonds in Pd^0-Catalyzed C–H Arylation

Matthew Wheatley[a], Marco Zuccarello[a], Maria Tsitopoulou[a], Stuart A. Macgregor[b*], and Olivier Baudoin[a*]

[a] *Department of Chemistry, University of Basel, 4056 Basel, Switzerland*
[b] *Institute of Chemical Sciences, Heriot-Watt University, Edinburgh EH14 4AS, U.K.*

* E-mail: olivier.baudoin@unibas.ch; S.A.Macgregor@hw.ac.uk

Contents

1. General methods 4

2. Optimization Studies 5

3. Catalyst preparation 6

4. Substrate synthesis 7

4.1 General procedure A: alkylation of 2-substituted dimethyl malonates 7

4.2 General procedure B: alkylation of dimethyl benzylmalonate 8

5. Product synthesis 18

5.1 General procedure C: Synthesis of indane products by Pd(0)-catalyzed C(sp^3)−H activation of methylene C−H bonds. 18

6. Kinetic experiments 24

6.1. General procedure for kinetic experiments 24

6.2. Initial rate experiments 25

6.2.1. 8a 25

6.2.2. 8a-*d5* 25

6.2.3. 8b 26

6.2.4. 8c 26

6.2.5. 8d 27

6.2.6. 8e 28

6.2.7. 8f 28

6.2.8. 8g 29

6.3. Kinetic analysis 29

6.3.1. Order on aryl bromide 29

6.3.2. Order on catalyst 30

6.3.3. Order on CsOPiv 32

6.3.4. Order on Cs_2CO_3 33

6.3.5. Kinetic Isotope Effects 34

6.3.6. Catalyst activation studies 37
6.3.7. Reaction in absence of CsOPiv 40
6.3.8. Reaction in absence of Cs_2CO_3 40
6.3.9. Stirring rate effect on reaction 41
7. Spectra 43
8. Computational Details. 108
9. Computed Results 109
9.1. Ar–Br Oxidative Addition 109
9.2. Reaction Profiles for Various R Substituents 110
9.3. Comparison of Different Reductive Elimination Pathways 116
9.4. Proton Transfer to Terminal Carbonate Base 117
9.5. Computed Rate-Determining Process when R = CO_2Me 118
9.6. Model and Functional Testing 120
9.7. Geometries of the Agostic Intermediates for Selected Substituents 123
9.8. Natural Bond Orbital Analyses 124
9.9. Computed Geometries (Å) and Energies (hartrees) 129
10. References 216

1. General methods

Techniques:

All reactions involving air-sensitive material were carried out in pre-dried glassware under an argon atmosphere by using Schlenk techniques employing double-line argon-vacuum lines and working in an argon-filled glove box. Analytical thin layer chromatography (TLC) was performed using pre-coated Merck silica gel 60 F254 plates (0.25 mm). Visualization of the developed chromatogram was performed by UV absorbance (254 nm) or TLC stains ($KMnO_4$ and Phosphomolybdic acid). Flash chromatography was performed using Silicycle SiliaFlash P60 (230-400 mesh) with the indicated solvent system, using gradients of increasing polarity in most cases.

Chemicals:

Anhydrous solvents were purchased from Acros Organics or Sigma-Aldrich. The solvents were degassed by three cycles of freeze-pump-thaw and storing in single-necked flasks equipped with a J-Young PTFE valve when necessary. Chemicals were purchased from Sigma-Aldrich, Acros Organics, Alfa Aesar Apollo scientific and Fluorochem and used as received without further purification unless otherwise stated.

Instrumentation:

GCMS analyses were performed with a Shimadzu QP2010SB GCMS apparatus on a Rtx®-5ms-Low-Bleed column lined with a mass (EI) detection system. Melting points were obtained on a Büchi melting point M-565, and are uncorrected. IR spectra were recorded on an ATR Varian Scimitar 800 and are reported in reciprocal centimeters (cm^{-1}). Nuclear magnetic resonance spectra were recorded on a Bruker Advance 400 (400 MHz), Advance 500 (500 MHz) and Advance 600 (600 MHz) in deuterated chloroform (residual peaks ^{1}H δ 7.26 ppm, ^{13}C δ 77.16 ppm) unless otherwise noted. Both, ^{13}C and ^{19}F NMR spectra are ^{1}H ($\{^{1}H\}$) decoupled unless otherwise stated. Data are reported in parts per million (ppm) as follows: chemical shift, multiplicity (s = singlet, d = doublet, t = triplet, q = quartet, quint = quintuplet, sept = septuplet, m = multiplet and brs = broad singlet), coupling constant in Hz and integration. High resolution mass spectra were recorded by Dr. M. Pfeffer and S. Mittelheisser (Department of Chemistry, University of Basel) on a Bruker maXis 4G QTOF ESI mass spectrometer.

2. Optimization Studies

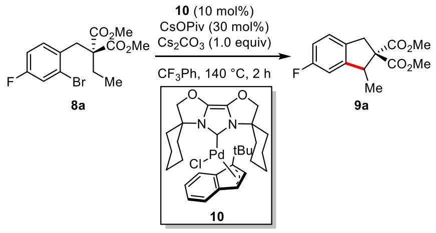

Entry	Deviation from standard conditions	^{19}F NMR Yield (%)[a]
**1**	**none**	**35**
2	KOPiv (30 mol%)	19
3	CsOAc (30 mol%)	9
4	DMBA (30 mol%)	0
5	PivNHOH (30 mol%)	2
6	CF_3Ph/DMSO 90:10	37
7	CF_3Ph/DMSO 95:5	45

3. Catalyst preparation

^{tBuInd}Pd(IBiox6)Cl (10):

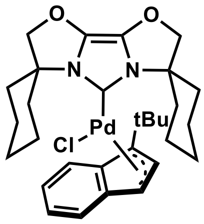

10

$C_{30}H_{40}ClN_2O_2Pd$

M= 602,53 g.mol^{-1}

The title compound (**10**) was prepared using a modified procedure from Kündig.[1] In the glovebox a schlenk tube was charged with [^{tBuInd}PdCl]$_2$ (1.07 g, 1.71 mmol, 1.0 eq.) IBiox6.HOTf (1.50 g, 3.42 mmol, 2.0 equiv) and NaOtBu (329 mg, 3.42 mmol, 2.0 equiv) and sealed with a rubber septum. Outside the glovebox, 1,2-dimethoxyethane (70 mL) was added and the reaction was stirred at 24 °C for 18 h. The reaction was quenched with aq. NH_4Cl and extracted with CH_2Cl_2. The combined organic layers were washed with brine, dried over and evaporated under reduced pressure. The crude mixture was purified by column chromatography (cyclohexane:EtOAc, 80:20) to obtain a deep red slurry which was triturated in a diethyl ether/pentane mixture to obtain product **10** (1.25 g, 2.08 mmol, 61%) as bright yellow solid.

^{1}H NMR (500 MHz, C_6D_6): δ (ppm) 7.52 (dd, *J* = 7.7, 0.9 Hz, 1H), 6.98 (dt, *J* = 7.7, 4.3 Hz, 1H), 6.81 (d, *J* = 4.2 Hz, 2H), 6.64 (d, *J* = 2.8 Hz, 1H), 5.08 (d, *J* = 2.8 Hz, 1H), 4.03 – 3.99 (m, 3H), 3.96 (d, *J* = 8.5 Hz, 1H), 3.17 (td, *J* = 14.0, 4.1 Hz, 1H), 2.77 (td, *J* = 13.9, 4.1 Hz, 1H), 2.13 (td, *J* = 13.6, 12.9, 4.4 Hz, 1H), 1.88 – 1.83 (m, 1H), 1.83 – 1.77 (m, 2H), 1.73 (s, 9H), 1.51 – 1.41 (m, 5 H), 1.39 – 1.31 (m, 2H), 1.26 (td, *J* = 7.6, 6.9, 3.6 Hz, 1H), 1.23 – 1.17 (m, 1H), 1.10 (qt, *J* = 13.4, 3.6 Hz, 1H), 0.96 – 0.83 (m, 1H), 0.72 – 0.62 (m, 2H), 0.60 – 0.52 (m, 1H), 0.51 – 0.40 (m, 1H).

^{13}C NMR (126 MHz, C_6D_6): δ (ppm) 141.5, 141.3, 139.3, 126.5, 126.2, 124.8, 124.0, 120.8, 117.8, 116.7, 109.6, 84.4, 84.3, 65.1, 64.1, l63.1, 36.3, 35.6, 35.1, 35.0, 34.5, 29.9, 25.4, 24.2, 24.0, 24.0, 23.7, 23.4.

IR (neat): ν (cm^{-1}) 2939, 2859, 1756, 1453, 1415, 1350, 1209, 959, 853, 750

HRMS (ESI): Calcd for $C_{30}H_{39}N_2O_2$ Pd [M-Cl]$^+$: 565.2052, found 565.2061

Rf: 0.18 (cyclohexane/EtOAc 80:20)

Mp: decomposition upon heating

4. Substrate synthesis

Dimethyl 2-(2-bromo-4-fluorobenzyl)malonate (S1):

The title compound was prepared according to a literature procedure.[1] **S1** was obtained as a colorless solid (8.00 g, 25.08 mmol, 84%). The NMR spectra are in accordance to the report.

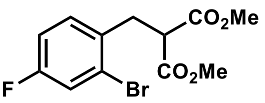

S1
$C_{12}H_{12}BrFO_4$
M= 319,13 g.mol^{-1}

^{1}H NMR (500 MHz, $CDCl_3$): δ (ppm) 7.29 (dd, $^3J_{HF}$ = 8.2, 2.6 Hz, 1H), 7.22 (dd, J = 8.6, 6.0 Hz, 1H), 6.94 (ddd, J = 8.2, 8.3, 2.6 Hz, 1H), 3.83 (t, J = 7.8 Hz, 1H), 3.70 (s, 6H) 3.31 (d, J = 7.8 Hz, 2H)

^{13}C NMR (126 MHz, $CDCl_3$): δ (ppm) 169.0, 161.6 (d, J = 250.2 Hz), 133.0 (d, J = 3.6 Hz), 132.4 (d, J = 8.3 Hz), 124.5 (d, J = 9.5 Hz), 120.3 (d, J = 24.4 Hz), 114.7 (d, J = 20.9 Hz), 52.8, 51.4, 34.5

^{19}F NMR (471 MHz, $CDCl_3$): δ (ppm) -113.4

4.1 General procedure A: alkylation of 2-substituted dimethyl malonates

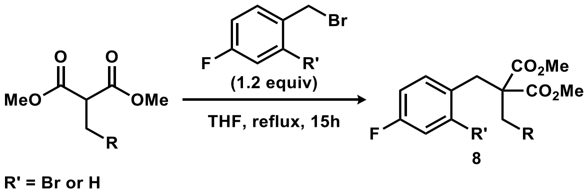

In a 10-20 mL microwave reaction vial, dimethyl 2-alkylmalonate (1.0 equiv) was diluted in THF (3 mL/mmol), then sodium hydride (60% in mineral oil, 1.2 equiv) was added in one portion and the mixture was stirred for 30 min at room temperature. 2-Bromo-4-fluorobenzyl bromide was dissolved in THF (1 mL/mmol) and added to the reaction mixture. The vial was capped and heated to reflux in an aluminium block for 15 h. The reaction was quenched by addition of H_2O and brine. The phases were separated, and the aqueous layer was extracted with diethyl ether or ethyl acetate. The combined organic phases were washed with brine and dried over Na_2SO_4. After evaporation of the volatiles, the crude product was purified by flash column chromatography (pentane/EtOAc) to yield the corresponding product **1**.

4.2 General procedure B: alkylation of dimethyl benzylmalonate

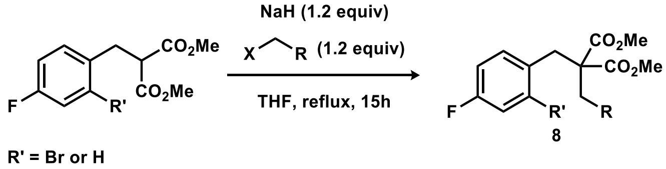

In a 10-20 mL microwave reaction vial, dimethyl 2-(2-bromo-4-fluorobenzyl)malonate (1.0 equiv) was diluted in THF (3 mL/mmol), then sodium hydride (60% in mineral oil, 1.2 equiv) was added in one portion and the mixture was stirred for 30 min at room temperature. Alkyl halide was dissolved in THF (1 mL/mmol) and added to the reaction mixture. The vial was capped and heated to reflux in an aluminium block for 15 h. The reaction was quenched by the addition of H_2O and brine. The phases were separated, and the aqueous layer was extracted with diethyl ether or ethyl acetate. The combined organic phases were washed with brine and dried over Na_2SO_4. After evaporation of the volatiles, the crude product was purified by flash column chromatography (pentane/EtOAc) to yield the corresponding product **1**.

Dimethyl 2-ethyl-2-(4-fluorobenzyl)malonate (8a-dehalo):

According to **general procedure A**, dimethyl 2-ethylmalonate (497 mg, 3.10 mmol, 1.0 equiv) 1-(bromomethyl)-4-fluorobenzene (700 mg, 3.70 mmol, 1.2 equiv) **8a-dehalo** was obtained as a colorless oil (704 mg, 2.62 mmol, 85%).

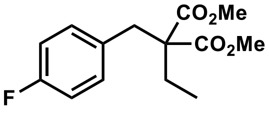

8a-dehalo
$C_{14}H_{17}FO_4$
M= 268,28 g.mol^{-1}

^{1}H NMR (500 MHz, $CDCl_3$): δ (ppm) 7.08 – 7.00 (m, 2H), 6.98 – 6.92 (m, 2H), 3.71 (s, 6H), 3.20 (s, 2H), 1.84 (q, *J* = 7.6 Hz, 2H), 0.91 (t, *J* = 7.6 Hz, 3H).

^{13}C NMR (126 MHz, $CDCl_3$): δ (ppm) 171.7, 162.1 (d, *J* = 245.2 Hz), 132.0 (d, *J* = 3.2 Hz), 131.4 (d, *J* = 7.9 Hz), 115.3 (d, *J* = 21.2 Hz), 70.0 (d, *J* = 1.3 Hz), 52.5, 37.2, 25.2, 8.9.

^{19}F NMR (471 MHz, $CDCl_3$): δ (ppm) -115.9

IR (neat): ν (cm^{-1}) 2954, 1730, 1510, 1436, 1219, 1119, 841, 740

HRMS (ESI): Calcd for $C_{14}H_{17}FNaO_4$ $[M+Na]^+$: 291.1003, found 291.1007

Rf: 0.36 (cyclohexane/EtOAc 90:10)

Dimethyl 2-(ethyl-d5)-2-(4-fluorobenzyl)malonate (8a-*d5*-dehalo):

According to **general procedure A**, 2-Methoxycarbonyl-succinic acid dimethyl ester (633 mg, 3.10 mmol, 1.0 equiv) was reacted with 1-(bromomethyl)-4-fluorobenzene (700 mg, 3.70 mmol, 1.2 equiv). **8a-*d5*-dehalo** was obtained as a colorless oil (94.5 mg, 346 µmol, 33%).

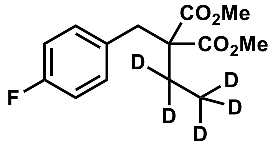

8a-*d5*-dehalo
$C_{14}H_{12}D_5FO_4$
M=273,31 g.mol^{-1}

^{1}H NMR (500 MHz, $CDCl_3$): δ (ppm) 7.07 – 7.00 (m, 2H), 6.96 – 6.89 (m, 2H), 3.69 (s, 6H), 3.19 (s, 2H).

^{13}C NMR (126 MHz, $CDCl_3$): δ (ppm) 171.7, 162.1 (d, J = 245.2 Hz), 132.0 (d, J = 3.4 Hz), 131.4 (d, J = 7.9 Hz), 115.3 (d, J = 21.3 Hz), 59.5 52.4, 37.2.

^{19}F NMR (471MHz, $CDCl_3$): δ (ppm) -115.9

IR (neat): ν (cm^{-1}) 2954, 1730, 1510, 1436, 1222, 1102, 1069, 843

HRMS (ESI): Calcd for $C_{14}H_{12}D_5FNaO_4$ [M+Na]$^+$: 296.1317, found 296.1317

Rf: 0.24 (cyclohexane/EtOAc 95:5)

Dimethyl 2-(4-fluorobenzyl)-2-methylmalonate (8b-dehalo):

According to **general procedure A**, dimethyl 2-methylmalonate (453 mg, 3.10 mmol, 1.0 equiv) was reacted with 1-(bromomethyl)-4-fluorobenzene (700 mg, 3.70 mmol, 1.2 equiv). **8b-dehalo** was obtained as a colorless liquid (640 mg, 2.52 mmol, 81%).

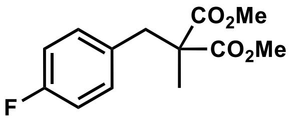

8b-dehalo
$C_{13}H_{15}FO_4$
M= 254,26 g.mol^{-1}

^{1}H NMR (400 MHz, $CDCl_3$): δ (ppm) 7.10–7.04 (m, 2H), 6.99 – 6.90 (m, 2H), 3.73 (s, 6H), 3.20 (s, 2H), 1.34 (s, 3H).

^{13}C NMR (126 MHz, $CDCl_3$): δ (ppm) 172.3, 162.1 (d, J = 245.3 Hz), 131.9 (d, J = 3.4 Hz), 131.8 (d, J = 7.9 Hz), 115.2 (d, J = 21.2 Hz), 55.0 (d, J = 1.3 Hz), 52.7, 40.6, 19.9.

^{19}F NMR (376 MHz, $CDCl_3$): δ (ppm) -115.9

IR (neat): ν (cm^{-1}) 2955, 1731, 1510, 1221, 1107, 841, 792

HRMS (ESI): Calcd for $C_{13}H_{15}FNaO_4$ [M+Na]$^+$: 277.0847, found 277.0852

Rf: 0.31 (cyclohexane/EtOAc 90:10)

Trimethyl 3-(4-fluorophenyl)propane-1,2,2-tricarboxylate (8c-dehalo):

According to **general procedure A**, 2-Methoxycarbonyl-succinic acid dimethyl ester (633 mg, 3.10 mmol, 1.0 equiv) was reacted with 1-(bromomethyl)-4-fluorobenzene (700 mg, 3.70 mmol, 1.2 equiv). **8c-dehalo** was obtained as a colorless oil (682 mg, 2.18 mmol, 70%).

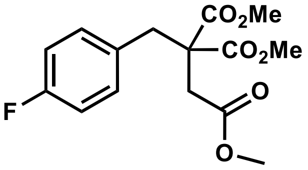

8c-dehalo
$C_{15}H_{17}FO_6$
M= 312,29 g.mol-1

^{1}H NMR (400 MHz, $CDCl_3$): δ (ppm) 7.07 – 7.01 (m, 2H), 6.99 – 6.92 (m, 2H), 3.75 (s, 6H), 3.70 (s, 3H), 3.35 (s, 2H), 2.86 (s, 2H)
^{13}C NMR (126 MHz, $CDCl_3$): δ (ppm) 171.1, 170.4, 162.2 (d, *J* = 245.9 Hz), 131.7 (d, *J* = 8.0 Hz), 131.4 (d, *J* = 3.4 Hz), 115.5 (d, *J* = 21.2 Hz), 56.8 (d, *J* = 1.4 Hz), 53.0, 52.1, 38.1, 36.8
^{19}F NMR (376 MHz, $CDCl_3$): δ (ppm) -115.4
IR (neat): ν (cm^{-1}) 2956, 1733, 1511, 1282, 1221, 1168, 846,
HRMS (ESI): Calcd for $C_{15}H_{17}FNaO_6$ $[M+Na]^+$: 335.0901 found 335.0905
Rf: 0.19 (cyclohexane/EtOAc 90:10)

Dimethyl 2-cyclopropyl-2-(4-fluorobenzyl)malonate (8d-dehalo):

To a solution of **(8d)** (400 mg, 1.1 mmol, 1.0 eq.) and Et_3N (134 mg, 1.32 mmol, 1.2 eq.) in MeOH (X mL) under argon atmosphere Pd/C was added and the mixture was purged with H_2. The reaction was stirred for 18 h at 24 °C. The mixture was wiltered over a pad of celite and the volatiles removed under reduced pressure giving **8d-dehalo** (301 mg, 1.07 mmol, 97%) as a colorless liquid.

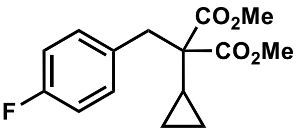

8d-dehalo
$C_{15}H_{17}FO_4$
M= 280,30 g.mol^{-1}

^{1}H NMR (500 MHz, $CDCl_3$): δ (ppm) 7.17 – 7.11 (m, 2H), 6.97 – 6.91 (m, 2H), 3.70 (s, 6H), 3.27 (s, 2H), 1.14 (tt, *J* = 8.6, 5.6 Hz, 1H), 0.61 – 0.56 (m, 2H), 0.41 – 0.35 (m, 2H).
^{13}C NMR (126 MHz, $CDCl_3$): δ (ppm) 171.0, 162.1 (d, *J* = 245.1 Hz), 132.0 (d, *J* = 3.3 Hz), 131.8 (d, *J* = 7.9 Hz), 115.1 (d, *J* = 21.2 Hz), 60.2 (d, *J* = 1.3 Hz), 52.4, 40.7, 14.5, 2.9.
^{19}F NMR (376 MHz, $CDCl_3$): δ (ppm) -116.0
IR (neat): ν (cm^{-1}) 2954, 1729, 1510, 1436, 1245, 1199, 1048, 828
HRMS (ESI): Calcd for $C_{15}H_{17}FNaO_4$ $[M+Na]^+$: 303.1003, found 303.1004
Rf: 0.43 (cyclohexane/EtOAc 90:10)

Dimethyl 2-(2-(dimethylamino)-2-oxoethyl)-2-(4-fluorobenzyl)malonate (8e-dehalo):

According to **general procedure B**, dimethyl 2-(4-fluorobenzyl)malonate (500 mg, 2.08 mmol, 1.0 equiv) was reacted with 2-chloro-*N*,*N*-dimethylacetamide (354 mg, 2.91 mmol, 1.4 equiv). **8e-dehalo** was obtained as a colorless solid (552 mg, 2.08 mmol, 82%).

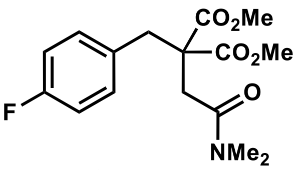

8e-dehalo
$C_{16}H_{20}FNO_5$
M= 325,34 g.mol-1

^{1}H NMR (400 MHz, $CDCl_3$): δ (ppm) 6.99 – 6.78 (m, 4H), 3.75 (s, 6H), 3.47 (s, 2H), 2.95 (s, 3H), 2.89 (s, 3H), 2.81 (s, 2H).
^{13}C NMR (126 MHz, $CDCl_3$): δ (ppm) 171.1, 169.6, 162.1 (d, *J* = 245.6 Hz), 132.5 (d, *J* = 3.4 Hz), 131.4 (d, *J* = 7.9 Hz), 115.4 (d, *J* = 21.1 Hz), 57.0 (d, *J* = 1.3 Hz), 52.9, 37.8, 37.2, 35.8, 35.6.
^{19}F NMR (376 MHz, $CDCl_3$): δ (ppm) -115.7
IR (neat): ν (cm^{-1}) 2962, 1729, 1641, 1510, 1209, 1182, 823
HRMS (ESI): Calcd for $C_{16}H_{21}FNNaO$ $[M+Na]^+$: 348.1218 found 348.1218
Rf: 0.43 (cyclohexane/EtOAc 50:50)

Dimethyl 2-benzyl-2-(4-fluorobenzyl)malonate (8f-dehalo):

According to **general procedure A**, dimethyl 2-benzylmalonate (689 mg, 3.10 mmol, 1.0 equiv) was reacted with 1-(bromomethyl)-4-fluorobenzene (700 mg, 3.70 mmol, 1.2 equiv). **8f-dehalo** was obtained as a colorless oil (610 mg, 1.85 mmol, 60%).

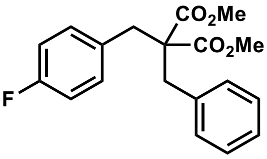

8f-dehalo
$C_{19}H_{19}FO_4$
M= 330,36 g.mol^{-1}

^{1}H NMR (400 MHz, $CDCl_3$): δ (ppm) 7.33 – 7.22 (m, 3H), 7.16 – 7.09 (m, 4H), 7.00 – 6.92 (m, 2H), 3.64 (s, 6H), 3.23 (s, 2H), 3.18 (s, 2H)
^{13}C NMR (126 MHz, $CDCl_3$): δ (ppm) 171.3, 162.1 (d, *J* = 245.5 Hz), 136.1, 132.1 (d, *J* = 3.4 Hz), 131.7 (d, *J* = 7.9 Hz), 130.1, 128.5, 127.2, 115.2 (d, *J* = 21.3 Hz), 60.6 (d, *J* = 1.3 Hz), 52.4, 39.7, 38.8.
^{19}F NMR (376 MHz, $CDCl_3$): δ (ppm) -115.8
IR (neat): ν (cm^{-1}) 2952, 1728, 1510, 1201, 1173, 843, 701
HRMS (ESI): Calcd for $C_{19}H_{19}FNaO_4$ $[M+Na]^+$: 353.1160, found 353.1163
Rf: 0.42 (cyclohexane/EtOAc 90:10)

Dimethyl 2-(4-fluorobenzyl)-2-(methoxymethyl)malonate (8g-dehalo):

According to **general procedure B**, dimethyl 2-(4-fluorobenzyl)malonate (500 mg, 2.08 mmol, 1.0 equiv) was reacted with MOMBr (364 mg, 2.91 mmol, 1.1 equiv). **8g-dehalo** was obtained as a colorless oil (280.0 mg, 2.08 mmol, 48%).

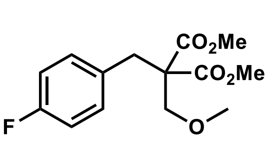

8g-dehalo
$C_{14}H_{17}FO_5$
M= 284,11 g.mol-1

^{1}H NMR (500 MHz, $CDCl_3$): δ (ppm) 7.09 – 7.02 (m, 2H), 6.98 – 6.92 (m, 2H), 3.73 (s, 6H), 3.59 (s, 2H), 3.35 (s, 3H), 3.32 (s, 2H).
^{13}C NMR (126 MHz, $CDCl_3$): δ (ppm) 170.0, 162.1 (d, *J* = 245.2 Hz), 131.7 (d, *J* = 3.3 Hz), 131.6 (d, *J* = 7.9 Hz), 115.3 (d, *J* = 21.2 Hz), 71.1, 59.6 (d, *J* = 1.1 Hz), 59.1, 52.7, 35.6.
^{19}F NMR (471 MHz, $CDCl_3$): δ (ppm) -115.9

IR (neat): ν (cm^{-1}) 2954, 1734, 1511, 1219, 1159, 1099, 848,

HRMS (ESI): Calcd for $C_{14}H_{17}FNaO_5$ [M+Na]$^+$: 307.0952 found 307.0956

Rf: 0.32 (cyclohexane/EtOAc 90:10)

Dimethyl 2-(4-fluorobenzyl)-2-isopropylmalonate (8h-dehalo):

According to **general procedure C** using 0.1 mmol of substrate the title compound (6 mg, 21 μmol, 21%) was obtained as a colorless oil (side product of C–H activation).

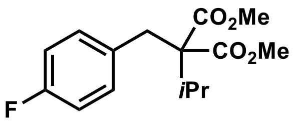

8h-dehalo
$C_{15}H_{19}FO_4$
M = 282.31 g.mol^{-1}

^{1}H NMR (500 MHz, $CDCl_3$): δ (ppm) 7.12 (m, 2H), 6.93 (m, 2H), 3.63 (s, 6H), 3.21 (s, 2H), 2.32 (sept, *J* = 6.9 Hz, 1H), 1.03 (s, 3H), 1.02 (s, 3H).

^{13}C NMR (126 MHz, $CDCl_3$): δ (ppm) 171.01, 162.01 (d, *J* = 245.2 Hz), 132.70 (d, *J* = 3.4 Hz), 131.64 (d, *J* = 7.9 Hz), 114.98 (d, *J* = 21.1 Hz), 64.21, 51.92, 39.46, 33.11, 18.88.

^{19}F NMR (376 MHz, $CDCl_3$): δ (ppm) -116.1

IR (neat): ν (cm^{-1}) 2954, 2342, 1731, 1488, 1435, 1256, 1226, 1042, 872, 660

HRMS (ESI): Calcd for $C_{15}H_{19}FNaO_4$ [M+Na]$^+$: 305.1160, found 305.1161

Rf: 0.19 (cyclohexane/EtOAc 94:6)

Dimethyl 2-(2-bromo-4-fluorobenzyl)-2-ethylmalonate (8a):

According to **general procedure B**, 2-(2-bromo-4-fluorobenzyl)malonate (1.00 g, 3.13 mmol, 1.0 equiv) was reacted with EtI (683 mg, 4.38 mmol, 1.4 equiv). **8a** was obtained as a colorless oil (842 mg, 2.42 mmol, 78%).

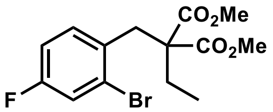

8a
$C_{14}H_{16}FBrO_4$
M= 347,18 g.mol^{-1}

^{1}H NMR (400 MHz, $CDCl_3$): δ (ppm) 7.30-7.25 (m, 1H), 7.18 (dd, *J*= 8.7, 6.0 Hz, 1H), 6.94 (ddd, *J*= 8.7, 7.8, 2.7 Hz, 1H), 3.70 (s, 6H), 3.42 (s, 2H), 1.92 (q, *J* = 7.5 Hz, 2H), 0.93 (t, *J* = 7.5 Hz, 3H)

^{13}C NMR (126 MHz, $CDCl_3$): δ (ppm) 171.6, 161.2 (d, *J* = 250.4 Hz), 132.4 (d, *J* = 3.79 Hz) 132.2 (d, *J* = 8.2 Hz), 125.9 (d, *J* = 9.0 Hz), 120.2 (d, *J* = 24.2 Hz), 114.6 (d, *J* = 20.8 Hz), 59.5, 52.5, 36.8, 26.4, 9.4

^{19}F NMR (471 MHz, $CDCl_3$): δ (ppm) -113.7

IR (neat): ν (cm^{-1}) 2952, 1729, 1599, 1487, 1221, 1115, 1032, 879, 778

HRMS (ESI): Calcd for $C_{14}H_{16}{}^{79}BrFNaO_4$ [M+Na]$^+$: 369.0108, found 369.0111

Rf: 0.23 (pentane/EtOAc 95:5)

Dimethyl 2-(2-bromo-4-fluorobenzyl)-2-(ethyl-*d*$_5$)malonate (8a-*d5*):

According to **general procedure B**, 2-(2-bromo-4-fluorobenzyl)malonate (1.02 g, 3.20 mmol, 1.0 equiv) was reacted with iodoethane-*d5* (721 mg, 4.48 mmol, 1.4 equiv). **8a-*d5*** was obtained as a colorless oil (934 mg, 2.65 mmol, 83%).

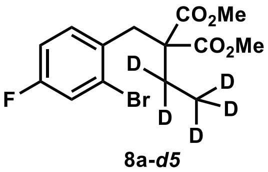

8a-*d5*
$C_{14}H_{11}D_5BrFO_4$
M= 352,21 g.mol^{-1}

^{1}H NMR (400 MHz, CDCl$_3$): δ (ppm) 7.27 (dd, *J* = 8.2, 2.6 Hz, 1H), 7.17 (dd, *J* = 8.7, 6.1 Hz, 1H), 6.97-6.90 (m, 1H), 3.70 (s, 6H), 3.41 (s, 2H)

^{13}C NMR (126 MHz, CDCl$_3$): δ (ppm) 171.6, 161.2 (d, *J* = 250.3 Hz), 132.4 (d, *J* = 3.7 Hz), 132.2 (d, *J* = 8.3 Hz), 125.9 (d, *J* = 9.3 Hz), 120.2 (d, *J* = 24.1 Hz), 114.6 (d, *J* = 20.7 Hz), 59.3, 52.5, 36.7, 26.0-25.1 (m), 8.8-7.8 (m)

^{19}F NMR (471 MHz, CDCl$_3$): δ (ppm) -113.7

IR (neat): ν (cm^{-1}) 2953, 1729, 1600, 1488, 1227, 1069, 1033, 882, 859, 831

HRMS (ESI): Calcd for $C_{14}H_{11}{}^{79}BrD_5NaO_4$ [M+Na]$^+$: 374.0422, found 374.0424

Rf: 0.23 (pentane/EtOAc 95:5)

Dimethyl 2-(2-bromo-4-fluorobenzyl)-2-methylmalonate (8b):

According to **general procedure B**, 2-(2-bromo-4-fluorobenzyl)malonate (500 mg, 1.57 mmol, 1.0 equiv) was reacted with MeI (267 mg, 1.88 mmol, 1.2 equiv). **8b** was obtained as a colorless solid (296 mg, 888 µmol, 57%).

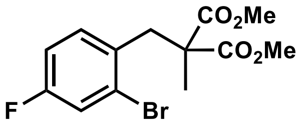

8b
$C_{13}H_{14}BrFO_4$
M= 333,15 g.mol^{-1}

^{1}H NMR (400 MHz, CDCl$_3$): δ (ppm) 7.30 (dd, *J* = 8.3, 2.7 Hz, 1H), 7.13 (dd, *J* = 8.7, 6.1 Hz, 1H), 6.94 (ddd, *J* = 8.7, 7.9, 2.7 Hz, 1H), 3.75 (s, 6H), 3.47 (s, 2H), 1.39 (s, 3H).

^{13}C NMR (126 MHz, CDCl$_3$): δ (ppm) 172.2, 161.3 (d, *J* = 250.5 Hz), 132.2 (d, *J* = 3.7 Hz), 132.2 (d, *J* = 8.2 Hz), 126.2 (d, *J* = 9.3 Hz), 120.3 (d, *J* = 24.2 Hz), 114.7 (d, *J* = 20.7 Hz), 55.2 (d, *J* = 1.3 Hz), 52.8, 38.9, 19.6.

^{19}F NMR (376 MHz, CDCl$_3$): δ (ppm) -113.5

IR (neat): ν (cm^{-1}) 2954, 1731, 1600, 1488, 1226, 1112, 882, 827

HRMS (ESI): Calcd for $C_{13}H_{14}{}^{79}BrFNaO_4$ [M+Na]$^+$: 354.9952, found 354.9954

Rf: 0.28 (cyclohexane/EtOAc 90:10)

Mp: 51.3 °C

Dimethyl 2-(2-bromo-4-fluorobenzyl)-2-(methyl-d3)malonate (8b-*d3*)

According to **general procedure B**, 2-(2-bromo-4-fluorobenzyl)malonate (1500 mg, 4.7 mmol, 1.0 equiv) was reacted with CD_3I (1022 mg, 7.05 mmol, 1.5 equiv). **8b-*d3*** was obtained as a colorless solid (1100 mg, 3.27 mmol, 70%).

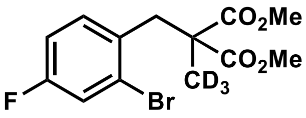

8b-*d3*
$C_{13}H_{11}D_3BrFO_4$
M= 336.17 g.mol^{-1}

^{1}H NMR (500 MHz, $CDCl_3$) δ (ppm) 7.28 (dd, *J* = 8.3, 2.7 Hz, 1H), 7.12 (dd, *J* = 8.7, 6.0 Hz, 1H), 6.93 (ddd, *J* = 8.8, 7.8, 2.7 Hz, 1H), 3.73 (s, 6H), 3.45 (s, 2H).

^{13}C NMR (126 MHz, $CDCl_3$) δ (ppm) 172.2, 161.3 (d, *J* = 250.5 Hz), 132.9 – 131.7 (m) (2C), 126.1 (d, *J* = 9.3 Hz), 120.2 (d, *J* = 24.1 Hz), 114.7 (d, *J* = 20.9 Hz), 55.0, 52.8, 38.8, 18.8 (hept, *J* = 19.9 Hz).

^{19}F NMR (376 MHz, $CDCl_3$): δ (ppm) -113.5

HRMS (ESI): Calcd for $C_{13}H_{11}D_3BrFO_4$[M+Na]$^+$: 305.0796, found 305.0801

IR (neat): ν (cm^{-1}) 2958, 1729, 1485, 1387, 1271, 1187, 1068

Rf: 0.20 (cyclohexane/EtOAc 95:5)

Mp: 46-48°C

Trimethyl 3-(2-bromo-4-fluorophenyl)propane-1,2,2-tricarboxylate (8c):

According to **general procedure A**, 2-Methoxycarbonyl-succinic acid dimethyl ester (954 mg, 4.67 mmol, 1.0 equiv) was reacted with 2-bromo-4-fluorobenzyl bromide (1.50 g, 5.60 mmol, 1.2 equiv). **8c** was obtained as a colorless oil (1.29 g, 3.30 mmol, 71%).

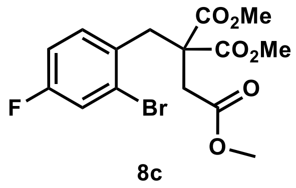

8c
$C_{15}H_{16}BrFO_6$
M= 391,19 g.mol-1

^{1}H NMR (500 MHz, $CDCl_3$): δ (ppm) 7.29 (dd, *J* = 8.3, 2.7 Hz, 1H), 7.10 (dd, *J* = 8.7, 6.0 Hz, 1H), 6.95 (td, *J* = 8.3, 2.7 Hz, 1H), 3.76 (s, 6H), 3.67 (s, 3H), 3.61 (s, 2H), 2.92 (s, 2H).

^{13}C NMR (126 MHz, $CDCl_3$): δ (ppm) 171.2, 170.4, 161.5 (d, *J* = 251.1 Hz), 132.5 (d, *J* = 8.3 Hz), 131.7 (d, *J* = 3.7 Hz), 126.0 (d, *J* = 9.4 Hz), 120.5 (d, *J* = 24.2 Hz), 114.9 (d, *J* = 20.9 Hz), 56.5 (d, *J* = 1.2 Hz), 53.1, 52.1, 37.2, 36.9.

^{19}F NMR (376 MHz, $CDCl_3$): δ (ppm) -112.8

IR (neat): ν (cm^{-1}) 2950, 2361, 1487, 1256, 1196, 1041, 846, 758

HRMS (ESI): Calcd for $C_{15}H_{16}{}^{79}BrFNaO_6$ [M+Na]$^+$: 413.0007, found 413.0011

Rf: 0.19 (cyclohexane/EtOAc 90:10)

Mp: 85.4 °C

Trimethyl 3-(2-bromo-4-fluorophenyl)propane-1,2,2-tricarboxylate-1,1-d2 (8c-*d2*)

According to **general procedure A**, 2-(2-bromo-4-fluorobenzyl)malonate (638 mg, 2.0 mmol, 1.0 equiv) was reacted with methyl bromoacetate-2,2-d2 (370 mg, 2.40 mmol, 1.2 equiv). **8c** was obtained as a colorless oil (652 mg, 1.66 mmol, 83%).

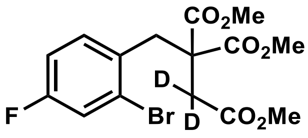

8c-*d2*
$C_{15}H_{14}D_2BrFO_6$
M= 393.20 g.mol-1

^{1}H NMR (500 MHz, CDCl3) δ (ppm) 7.28 (dd, *J* = 8.2, 2.7 Hz, 1H), 7.10 (dd, *J* = 8.7, 6.0 Hz, 1H), 6.95 (ddd, *J* = 8.7, 7.8, 2.7 Hz, 1H), 3.75 (s, 6H), 3.66 (s, 3H), 3.60 (s, 2H).

^{13}C NMR (126 MHz, CDCl3) δ (ppm) 171.2 , 170.4 , 161.5 (d, *J* = 251.1 Hz), 132.5 (d, *J* = 8.4 Hz), 131.7 (d, *J* = 3.6 Hz), 126.0 (d, *J* = 9.5 Hz), 120.4 (d, *J* = 24.3 Hz), 114.8 (d, *J* = 20.9 Hz), 56.3, 53.1, 52.0, 37.1, 36.7 – 36.1 (t, *J* = 19.8 Hz).

^{19}F NMR (471 MHz, CDCl3) δ -112.9

HRMS (ESI): Calcd for $C_{15}H_{15}BrD_2FO_6$ [M+H]$^+$: 393.0313, found 393.0305

IR (neat): ν (cm^{-1}) 3067, 2967, 1702, 1597, 1489, 1244

Rf: 0.19 (cyclohexane/EtOAc 90:10)

Mp: 82-85 °C

Dimethyl 2-(2-bromo-4-fluorobenzyl)-2-cyclopropylmalonate (8d):

According to **general procedure A**, dimethyl 2-cyclopropylmalonate (1.34 g, 7.81 mmol, 1.0 equiv) was reacted with 2-bromo-4-fluorobenzyl bromide (2.72 g, 10.2 mmol, 1.3 equiv). **8d** was obtained as a colorless oil (1.76 g, 4.89 mmol, 63%).

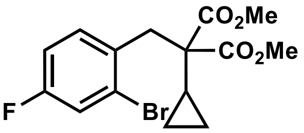

8d
$C_{15}H_{16}BrFO_4$
M= 359,19 g.mol^{-1}

^{1}H NMR (400 MHz, $CDCl_3$): δ (ppm) 7.28 (dd, J= 8.3, 2.7), 7.23 (dd, *J* = 8.7, 6.1 Hz, 1H), 6.94 (ddd, *J* = 8.6, 7.9, 2.7 Hz, 1H), 3.71 (s, 6H), 3.51 (s, 2H), 1.19 (tt, *J* = 8.6, 5.6, Hz, 1H), 0.62 – 0.52 (m, 2H), 0.49 – 0.36 (m, 2H).

^{13}C NMR (126 MHz, $CDCl_3$): δ (ppm) δ 170.9, 116.3 (d, *J* = 250.3 Hz), 132.4 (d, *J* = 3.7 Hz), 132.3 (d, *J* = 8.3 Hz), 126.3 (d, *J* = 9.4 Hz), 120.2 (d, *J* = 24.0 Hz), 114.5 (d, *J* = 20.7 Hz), 59.8 (d, *J* = 1.1 Hz), 52.6, 39.1, 14.8, 3.5.

^{19}F NMR (376 MHz, $CDCl_3$): δ (ppm) -113.8

IR (neat): ν (cm^{-1}) 2950, 1727, 1599, 1486, 1242, 1195, 1174, 1045, 860, 698

HRMS (ESI): Calcd for $C_{15}H_{16}{}^{79}BrFNaO_4$ [M+Na]$^+$: 381.0108, found 381.0104

Rf: 0.35 (cyclohexane/EtOAc 90:10)

Dimethyl 2-(2-bromo-4-fluorobenzyl)-2-(2-(dimethylamino)-2-oxoethyl)malonate (8e):

According to **general procedure B**, 2-(2-bromo-4-fluorobenzyl)malonate (1.00 g, 3.13 mmol, 1.0 equiv) was reacted with 2-chloro-*N*,*N*-dimethylacetamide (533 mg, 4.38 mmol, 1.4 equiv). **8e** was obtained as a colorless solid (1.15 g, 2.84 mmol, 91%).

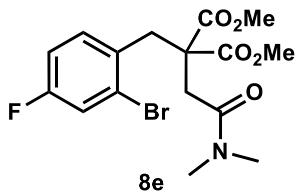

8e
$C_{16}H_{19}BrFNO_5$
M= 404,23 g.mol-1

^{1}H NMR (500 MHz, $CDCl_3$): δ (ppm) 7.28 (dd, *J* = 8.3, 2.7 Hz, 1H), 7.03 (dd, *J* = 8.6, 6.0 Hz, 1H), 6.93 (td, *J* = 8.3, 2.7 Hz, 1H), 3.75 (s, 6H), 3.68 (s, 2H), 2.92 (s, 3H), 2.92 (s, 3H), 2.87 (s, 2H).

^{13}C NMR (126 MHz, $CDCl_3$): δ (ppm) 171.1, 169.6, 161.4 (d, *J* = 250.7 Hz), 132.7 (d, *J* = 8.4 Hz), 132.6 (d, *J* = 3.7 Hz), 125.8 (d, *J* = 9.3 Hz), 120.4 (d, *J* = 24.1 Hz), 114.7 (d, *J* = 20.8 Hz), 56.7 (d, *J* = 1.3 Hz), 53.0, 37.3, 37.0, 36.4, 35.6.

^{19}F NMR (235 MHz, $CDCl_3$): δ (ppm) -113.2

IR (neat): ν (cm^{-1}) 2959, 1735, 1638 1485, 1203, 1134, 864, 674

HRMS (ESI): Calcd for $C_{16}H_{19}{}^{79}BrFNNaO_5$ $[M+Na]^+$: 426.0323, found 426.0325

Rf: 0.41 (cyclohexane/EtOAc 50:50)

Mp: 117.7 °C

Dimethyl 2-benzyl-2-(2-bromo-4-fluorobenzyl)malonate (8f):

According to **general procedure B**, 2-(2-bromo-4-fluorobenzyl)malonate (500 mg, 1.57 mmol, 1.0 equiv) was reacted with benzyl bromide (376 mg, 2.20 mmol, 1.4 equiv). **8f** was obtained as a colorless solid (615 mg, 1.50 mmol, 96%).

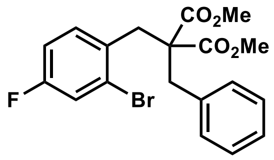

8f
$C_{19}H_{18}BrFO_4$
M= 409,5 g.mol^{-1}

^{1}H NMR (500 MHz, $CDCl_3$): δ (ppm) 7.34 (dd, *J* = 8.7, 6.1 Hz, 1H), 7.31 – 7.22 (m, 4H), 7.14 – 7.10 (m, 2H), 6.98 – 6.90 (m, 1H), 3.62 (s, 6H), 3.41 (s, 2H), 3.35 (s, 2H).

^{13}C NMR (126 MHz, $CDCl_3$): δ (ppm) 171.1, 161.2 (d, *J* = 250.2 Hz), 136.0, 132.6 (d, *J* = 3.7 Hz), 132.3 (d, *J* = 8.2 Hz), 130.1, 128.4, 127.3, 126.1 (d, *J* = 9.3 Hz), 119.9 (d, *J* = 24.2 Hz), 114.5 (d, *J* = 20.6 Hz), 60.1, 52.6, 41.2, 38.3.

^{19}F NMR (235 MHz, $CDCl_3$): δ (ppm) -113.8

IR (neat): ν (cm^{-1}) 2947, 1726, 1485, 1242, 1194, 1173, 849, 698

HRMS (ESI): Calcd for $C_{19}H_{18}{}^{79}BrFNaO_4$ $[M+Na]^+$: 431.0265 found 431.0269

Rf: 0.32 (cyclohexane/EtOAc 95:5)

Mp: 70.0 °C

Dimethyl 2-(2-bromo-4-fluorobenzyl)-2-(methoxymethyl)malonate (8g):

According to **general procedure B**, 2-(2-bromo-4-fluorobenzyl)malonate (700 mg, 2.19 mmol, 1.0 equiv) was reacted with MOMBr (328 mg, 2.63 mmol, 1.2 equiv). **8g** was obtained as a colorless solid (710 mg, 1.96 mmol, 89%).

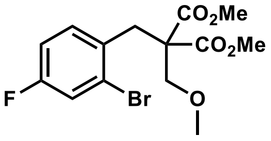

8g
$C_{14}H_{16}BrFO_5$
M= 363,18 g.mol^{-1}

^{1}H NMR (500 MHz, $CDCl_3$): δ (ppm) δ 7.27 (dd, J = 8.3, 2.7 Hz, 1H), 7.23 (dd, J = 8.7, 6.1 Hz, 1H), 6.96 (ddd, J = 8.6, 7.9, 2.7 Hz, 1H), 3.74 (s, 6H), 3.64 (s, 2H), 3.53 (s, 2H), 3.34 (s, 3H).

^{13}C NMR (126 MHz, $CDCl_3$): δ (ppm) 169.9, 161.3 (d, J = 250.5 Hz), 133.0 (d, J = 8.3 Hz), 131.9 (d, J = 3.7 Hz), 125.6 (d, J = 9.5 Hz), 120.2 (d, J = 24.2 Hz), 114.6 (d, J = 20.8 Hz), 71.6, 59.1, 58.9, 52.8, 35.0.

^{19}F NMR (235 MHz, $CDCl_3$): δ (ppm) -113.5

IR (neat): ν (cm^{-1}) 2921, 2885, 1728, 1487, 1227, 1201, 1100, 872, 797

HRMS (ESI): Calcd for $C_{14}H_{16}{}^{79}BrFNaO_5$ [M+Na]$^+$: 385.0057, found 385.0056

Rf: 0.25 (cyclohexane/EtOAc 90:10)

Mp: 86.3 °C

Dimethyl 2-(2-bromo-4-fluorobenzyl)-2-isopropylmalonate (8h):

According to **general procedure B**, 2-(2-bromo-4-fluorobenzyl)malonate (495 mg, 1.55 mmol, 1.0 equiv) was reacted with isopropyl iodide (369 mg, 2.17 mmol, 1.4 equiv). **8h** was obtained as a white solid (398 mg, 1.10 mmol, 71%).

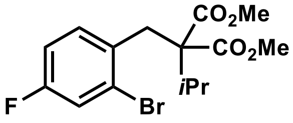

8h
$C_{15}H_{18}BrFO_4$
M = 361.21 g.mol^{-1}

^{1}H NMR (500 MHz, $CDCl_3$): δ (ppm) 7.36 (dd, J = 8.7, 6.1 Hz, 1H), 7.25 (dd, J = 8.3, 2.7 Hz, 1H), 6.93 (ddd, J = 8.7, 7.9, 2.7 Hz, 1H), 3.62 (s, 6H), 3.42 (s, 2H), 2.47 (sept, J = 6.8 Hz, 1H), 1.06 (s, 3H), 1.05 (s, 3H).

^{13}C NMR (126 MHz, $CDCl_3$): δ (ppm) 170.95, 161.06 (d, J = 249.8 Hz), 133.33 (d, J = 3.9 Hz), 132.31 (d, J = 8.2 Hz), 125.98 (d, J = 9.3 Hz), 119.76 (d, J = 24.1 Hz), 114.39 (d, J = 20.7 Hz), 63.04, 52.20, 36.97, 33.86, 18.87.

^{19}F NMR (376 MHz, $CDCl_3$): δ (ppm) -114.2

IR (neat): ν (cm^{-1}) 2361, 1728, 1604, 1510, 1435, 1223, 1123, 1046, 841, 632

HRMS (ESI): Calcd for $C_{15}H_{18}{}^{79}BrFNaO_4$ [M+Na]$^+$: 383.0265, found 383.0260

Rf: 0.27 (cyclohexane/EtOAc 95:5)

Mp: 65-67 °C

5. Product synthesis

5.1 General procedure C: Synthesis of indane products by Pd(0)-catalyzed C(sp^3)−H activation of methylene C−H bonds.

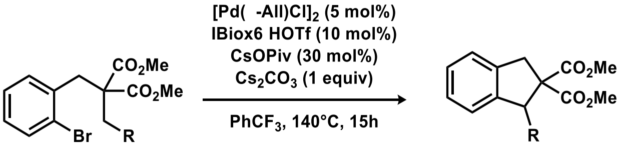

8 **9**

In an 2-5 mL microwave reaction vial, substrate (0.2 mmol) was introduced. Then the tube was transferred in the glovebox and [Pd(π-allyl)Cl]$_2$ (3.7 mg, 10 μmol, 5 mol%), IBiox6·HOTf (11.4 mg, 20 μmol, 10 mol%), cesium pivalate (14 mg, 60 μmol, 30 mol%), caesium carbonate (65 mg, 0.2 mmol, 1 equiv) and 5Å molecular sieves powder (50 mg) were introduced and the vial was then sealed. Outside of the glovebox, *α*,*α*,*α*-trifluorotoluene (2 mL) was added. The reaction was stirred at room temperature for 10 min. The vial was then introduced in a 140°C preheated aluminum block and stirred at this temperature for 15 hours. After this period the reaction was cooled to room temperature, diluted with CH_2Cl_2 (1.0 mL), filtered over a pad of Celite (washed three times with 1 mL of CH_2Cl_2). The crude material was analyzed by GC-MS and then concentrated and purified by preparative HPLC chromatography (EtOAc/hexane) to yield the corresponding indane product.

Dimethyl 6-fluoro-1-methyl-1,3-dihydro-2*H*-indene-2,2-dicarboxylate (9a):
According to **general procedure C** the title compound (27.0 mg, 101 μmol, 51%) was obtained as a colorless oil.

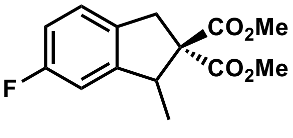

9a
$C_{14}H_{15}FO_4$
M= 266,27 g.mol-1

^{1}H NMR (500 MHz, $CDCl_3$): δ (ppm) 7.10 (dd, *J* = 9.0, 5.1 Hz, 1H), 6.89 – 6.80 (m, 2H), 4.00 (q, *J* = 7.2 Hz, 1H), 3.74 (s, 3H), 3.73 (s, 3H), 3.70 (d, *J* = 16.2 Hz, 1H), 3.25 (d, *J* = 16.2 Hz, 1H), 1.22 (d, *J* = 7.2 Hz, 3H).

^{13}C NMR (126 MHz, $CDCl_3$): δ (ppm) 172.1, 170.6, 162.7 (d, *J* = 243.7 Hz), 147.7 (d, *J* = 7.8 Hz), 134.4 (d, *J* = 2.6 Hz), 125.2 (d, *J* = 8.6 Hz), 114.1 (d, *J* = 22.5 Hz), 110.7 (d, *J* = 22.7 Hz), 65.4, 53.0, 52.6, 45.2 (d, *J* = 2.2 Hz), 38.5, 16.5.

^{19}F NMR (376 MHz, $CDCl_3$): δ (ppm) -116.2

IR (neat): ν (cm^{-1}) 2956, 2361, 1731, 1489, 1239, 1077, 872, 812

HRMS (ESI): Calcd for $C_{14}H_{15}FNaO_4$ [M+Na]$^+$: 289.0847, found 289.0845
Rf: 0.20 (cyclohexane/EtOAc 95:5)

Dimethyl 6-fluoro-1-(methyl-*d*$_3$)-1,3-dihydro-2*H*-indene-2,2-dicarboxylate-1-*d* (9a-*d4*):
According to **general procedure C** the title compound (17.0 mg, 62.9 µmol, 32%) was obtained as a colorless oil.

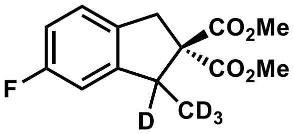

9a-*d4*
$C_{14}H_{11}D_4FO_4$
M= 270,29 g.mol-1

^{1}H NMR (250 MHz, $CDCl_3$): δ (ppm) 7.13 – 7.05 (m, 1H), 6.89 – 6.80 (m, 2H), 3.74 (s, 3H), 3.73 (s, 3H), 3.70 (d, *J* = 16.4 Hz, 1H), 3.25 (d, *J* = 16.4 Hz, 1H).
^{13}C NMR (126 MHz, $CDCl_3$): δ (ppm) 172.1, 170.6, 162.6 (d, *J* = 243.6 Hz), 147.6 (d, *J* = 7.9 Hz), 134.4 (d, *J* = 2.6 Hz), 125.2 (d, *J* = 8.6 Hz), 114.1 (d, *J* = 22.5 Hz), 110.7 (d, *J* = 22.6 Hz), 65.2, 53.0, 52.5, 38.5, 44.8-44.5 (m), 15.9-14.7 (m).
^{19}F NMR (376 MHz, $CDCl_3$): δ (ppm) -116.2
IR (neat): ν (cm^{-1}) 2955, 2848, 1732, 1489, 1257, 1164, 906, 729
HRMS (ESI): Calcd for $C_{14}H_{11}D_4FNaO_4$ [M+Na]$^+$: 293.1098, found 293.1102
Rf: 0.22 (cyclohexane/EtOAc 95:5)

Dimethyl 5-fluoro-1,3-dihydro-2*H*-indene-2,2-dicarboxylate (9b):
According to **general procedure C** the title compound (39.0 mg, 155 µmol, 77%) was obtained as a colorless oil.

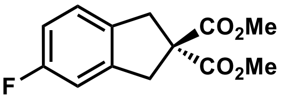

9b
$C_{13}H_{13}FO_4$
M= 252,24 g.mol-1

^{1}H NMR (500 MHz, $CDCl_3$): δ (ppm) 7.15 – 7.09 (m, 1H), 6.92 – 6.82 (m, 2H), 3.75 (s, 6H), 3.57 (s, 2H), 3.54 (s, 2H).
^{13}C NMR (126 MHz, $CDCl_3$): δ (ppm) 172.0, 162.5 (d, *J* = 243.7 Hz), 142.1 (d, *J* = 8.4 Hz), 135.4 (d, *J* = 2.5 Hz), 125.3 (d, *J* = 8.9 Hz), 114.1 (d, *J* = 22.5 Hz), 111.5 (d, *J* = 22.6 Hz), 60.9, 53.2, 40.7 (d, *J* = 2.3 Hz), 39.9.
^{19}F NMR (376 MHz, $CDCl_3$): δ (ppm) -116.5
IR (neat): ν (cm^{-1}) 2956, 2934, 1734, 1486, 1239, 1210, 1076, 809, 714
HRMS (ESI): Calcd for $C_{13}H_{13}FNaO_4$ [M+Na]$^+$: 275.0690, found 275.0695
Rf: 0.30 (cyclohexane/EtOAc 90:10)
Mp: 83.7 °C

Dimethyl 5-fluoro-1,3-dihydro-2H-indene-2,2-dicarboxylate-3,3-d2 (9b-*d2*)

According to **general procedure C**, the title compound was obtained as a white solid (48.8mg, 0.16 mmol, 82%).

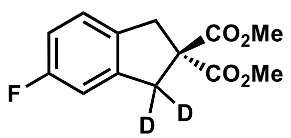

9b-*d2*
$C_{13}H_{11}D_2FO_4$
M= 254.25 g.mol-1

^{1}H NMR (500 MHz, $CDCl_3$) δ (ppm) 7.14 – 7.09 (m, 1H), 6.92 – 6.80 (m, 2H), 3.75 (s, 6H), 3.54 (s, 2H).

^{13}C NMR (126 MHz, CDCl3) δ (ppm) 171.9, 162.5 (d, *J* = 243.6 Hz), 141.9 (d, *J* = 8.5 Hz), 135.4 (d, *J* = 2.5 Hz), 125.3 (d, *J* = 8.8 Hz), 114.1 (d, *J* = 22.5 Hz), 111.5 (d, *J* = 22.7 Hz), 60.8, 53.1, 39.9.

^{19}F NMR (376 MHz, $CDCl_3$): δ (ppm) -116.5

HRMS (ESI): Calcd for $C_{13}H_{11}D_3BrFO_4$[M+Na]$^+$: 305.0796, found 305.0801

IR (neat): ν (cm^{-1}) 2962, 1731, 1614, 1433, 1265, 1100, 1063

Rf: 0.20 (cyclohexane/EtOAc 95:5)

Mp: 72-74 °C

Trimethyl 6-fluoro-1,3-dihydro-2*H*-indene-1,2,2-tricarboxylate (9c):

According to **general procedure C** the title compound (52.0 mg, 168 μmol, 84%) was obtained as a colorless oil.

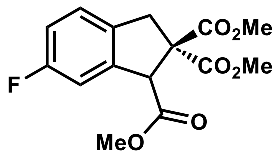

9c
$C_{15}H_{15}FO_6$
M= 310,28 g.mol-1

^{1}H NMR (400 MHz, $CDCl_3$): δ (ppm) 7.16 (dd, *J* = 8.3, 5.1 Hz, 1H), 7.07 (dd, *J* = 8.6, 2.5 Hz, 1H), 6.92 (td, *J* = 8.7, 2.5 Hz, 1H), 4.80 (s, 1H), 3.93 (d, *J* = 16.0 Hz, 1H), 3.75 (s, 3H), 3.72 (s, 3H), 3.68 (s, 3H), 3.43 (d, *J* = 16.0 Hz, 1H).

^{13}C NMR (126 MHz, $CDCl_3$): δ (ppm) 171.2, 170.7, 169.5, 162.4 (d, *J* = 245.0 Hz), 140.4 (d, *J* = 8.5 Hz), 135.8 (d, *J* = 2.7 Hz), 125.9 (d, *J* = 8.6 Hz), 115.7 (d, *J* = 22.5 Hz), 112.2 (d, *J* = 23.3 Hz), 64.4, 56.3 (d, *J* = 2.2 Hz), 53.6, 53.1, 52.6, 39.1.

^{19}F NMR (376 MHz, $CDCl_3$): δ (ppm) -115.3

IR (neat): ν (cm^{-1}) 2956, 2361, 1733, 1435, 1238, 1163, 1011, 879

HRMS (ESI): Calcd for $C_{15}H_{15}FNaO_6$ [M+Na]$^+$: 333.0754, found 333.0752

Rf: 0.23 (cyclohexane/EtOAc 90:10)

Trimethyl 6-fluoro-1,3-dihydro-2H-indene-1,2,2-tricarboxylate-1-d (9c-*d1*)

According to **general procedure C**, the title compound was obtained as a yellow oil (43.6mg, 0.14 mmol, 72%). This compound contains significant impurities that are not able to be separated. However, for the purposes of this study, we have confirmed the identity of the

compound and can use this data to confirm the values obtained in our KIE study for this reaction.

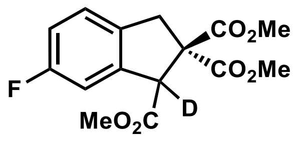

9c-*d1*
$C_{15}H_{14}DFO_6$
M= 311.28 g.mol-1

^{1}H NMR (500 MHz, $CDCl_3$) δ (ppm) 7.15 (dd, *J* = 8.4, 5.1 Hz, 1H), 7.06 (dd, *J* = 8.5, 2.4 Hz, 1H), 6.92 (ddd, *J* = 9.1, 8.3, 2.5 Hz, 1H), 3.92 (d, *J* = 16.2 Hz, 1H), 3.75 (s, 3H), 3.72 (s, 3H), 3.67 (s, 3H), 3.43 (d, *J* = 16.2, 1H).

^{13}C NMR (126 MHz, $CDCl_3$) δ (ppm) 171.2, 170.6, 169.5, 162.4 (d, *J* = 244.9 Hz), 140.3 (dd, *J* = 8.5, 6.5 Hz), 135.8 (dd, *J* = 4.8, 2.7 Hz), 125.9 (d, *J* = 8.6 Hz), 115.7 (d, *J* = 22.5 Hz), 112.2 (d, *J* = 23.3 Hz), 64.3 (d, *J* = 9.5 Hz), 56.3 (d, *J* = 2.3 Hz), 53.6, 53.1, 52.6, 39.0.

^{19}F NMR (471 MHz, CDCl3) δ (ppm) -115.3

HRMS (ESI): Calcd for $C_{15}H_{15}DFO_6$ [M+H]$^{+}$: 312.0988, found 312.0986

IR (neat): ν (cm^{-1}) 2956, 1734, 1612, 1435, 1251, 1100

Rf: 0.23 (cyclohexane/EtOAc 90:10)

Dimethyl 6'-fluorospiro[cyclopropane-1,1'-indene]-2',2'(3'H)-dicarboxylate (9d):

According to **general procedure C** the title compound (37.0 mg, 133 µmol, 67 %) was obtained as a colorless solid.

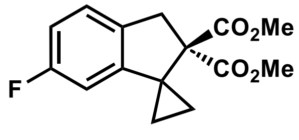

9d
$C_{15}H_{15}FO_4$
M= 278,28 g.mol-1

^{1}H NMR (500 MHz, $CDCl_3$): δ (ppm) δ 7.11 – 7.05 (m, 1H), 6.79 (ddd, *J* = 9.2, 8.3, 2.4 Hz, 1H), 6.37 (dd, *J* = 9.0, 2.4 Hz, 1H), 3.71 (s, 6H), 3.67 (s, 2H), 1.22 – 1.18 (m, 2H), 1.02 – 0.98 (m, 2H).

^{13}C NMR (126 MHz, $CDCl_3$): δ (ppm) 170.8, 163.1 (d, *J* = 243.4 Hz), 148.4 (d, *J* = 8.3 Hz), 133.9 (d, *J* = 2.5 Hz), 125.0 (d, *J* = 8.9 Hz), 113.3 (d, *J* = 22.7 Hz), 105.9 (d, *J* = 23.5 Hz), 64.5, 52.8, 39.7, 31.7 (d, *J* = 2.5 Hz), 15.0.

^{19}F NMR (376 MHz, $CDCl_3$): δ (ppm) -115.9

IR (neat): ν (cm^{-1}) 2958, 2361, 1730, 1493, 1251, 1155, 1059, 859

HRMS (ESI): Calcd for $C_{15}H_{15}FNaO_4$ [M+Na]$^{+}$: 301.0847, found 301.0853

Rf: 0.15 (cyclohexane/EtOAc 95:5)

Mp: 110.5 °C

Dimethyl 1-(dimethylcarbamoyl)-6-fluoro-1,3-dihydro-2*H*-indene-2,2-dicarboxylate (9e):

According to **general procedure C** the title compound (47.0 mg, 145 µmol, 73%) was obtained as a colorless oil.

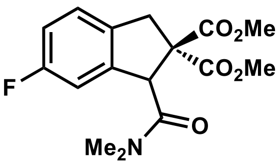

9e
$C_{16}H_{18}FNO_5$
M= 323,32 g.mol-1

^{1}H NMR (500 MHz, $CDCl_3$): δ (ppm) 7.15 (dd, *J* = 8.3, 5.1 Hz, 1H), 6.91 – 6.84 (m, 2H), 5.21 (s, 1H), 4.14 (d, *J* = 16.1 Hz, 1H), 3.72 (s, 3H), 3.71 (s, 3H), 3.37 (s, 3H), 3.37 (d, *J* = 16.1 Hz, 1H), 2.92 (s, 3H).
^{13}C NMR (126 MHz, $CDCl_3$): δ (ppm) 171.6, 171.4, 169.9, 162.3 (d, *J* = 244.1 Hz), 142.3 (d, *J* = 8.2 Hz), 136.9 (d, *J* = 2.7 Hz), 126.1 (d, *J* = 8.9 Hz), 115.1 (d, *J* = 22.4 Hz), 111.1 (d, *J* = 23.0 Hz), 65.5, 53.5, 53.1 (d, *J* = 2.0 Hz), 53.0, 39.6, 38.6, 36.0.
^{19}F NMR (376 MHz, $CDCl_3$): δ (ppm) -116.1
IR (neat): ν (cm^{-1}) 2959, 2361, 1730, 1640, 1492, 1219, 1161, 835, 628
HRMS (ESI): Calcd for $C_{16}H_{18}FNNaO_5$ $[M+Na]^+$: 346.1061, found 346.1067
Rf: 0.36 (cyclohexane/EtOAc 50:50)
Mp: 126.4 °C

Dimethyl 6-fluoro-1-phenyl-1,3-dihydro-2*H*-indene-2,2-dicarboxylatedicarboxylate (9f): Under Ar atmosphere at 0 °C dimethyl malonate (291 mg, 2.2 mmol, 1.1 eq.) was added to a suspension of NaH (264 mg, 60%, 6.60 mmol, 3.3 eq.) in THF (10 mL). After stirring for 30 min a solution of 2-(bromo(phenyl)methyl)-1-(bromomethyl)-4-fluorobenzene (715 mg, 2.00 mmol, 1.0 eq.) in THF (6.0 mL) was added. The reaction was heated to reflux for 16 h and then quenched by the addition of H_2O. The layers were separated and the aqueous phase was extracted with Et_2O (3 x 15 mL). The combined organic layers were dried over Na_2SO_4 and concentrated under reduced pressure. The crude product was purified by flash column chromatography (cyclohexane/EtOAc 90:10) to obtain the title compound (360 mg, 1.10 mmol, 55 %) as a colorless solid.

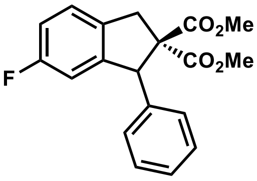

9f
$C_{19}H_{17}FO_5$
M= 328,34 g.mol-1

^{1}H NMR (250 MHz, $CDCl_3$): δ (ppm) 7.25 – 7.19 (m, 4H), 7.11 – 7.01 (m, 2H), 6.96 – 6.89 (m, 1H), 6.73 (dd, *J* = 8.8, 2.5 Hz, 1H), 5.33 (s, 1H), 3.97 (d, *J* = 16.6 Hz, 1H), 3.76 (s, 3H), 3.32 (d, *J* = 16.6 Hz, 1H), 3.21 (s, 3H).
^{13}C NMR (126 MHz, $CDCl_3$): δ (ppm) 172.1, 169.6, 162.7 (d, *J* = 244.3 Hz), 145.7 (d, *J* = 8.0 Hz), 139.3, 135.6 (d, *J* = 2.5 Hz), 129.3, 128.3, 127.6, 125.4 (d, *J* = 8.7 Hz), 114.9 (d, *J* = 22.7 Hz), 112.4 (d, *J* = 22.6 Hz), 67.1, 56.8 (d, *J* = 2.3 Hz), 53.2, 52.2, 39.1
^{19}F NMR (376 MHz, $CDCl_3$): δ (ppm) -115.6
IR (neat): ν (cm^{-1}) 2951, 2361, 1727, 1487, 1248, 1206, 747, 705
HRMS (ESI): Calcd for $C_{19}H_{17}FNaO_4$ $[M+Na]^+$: 351.1003, found 351.1008
Rf: 0.25 (cyclohexane/EtOAc 90:10)

Mp: 110.7 °C

Dimethyl 6-fluoro-1-methoxy-1,3-dihydro-2*H*-indene-2,2-dicarboxylate (9g):

According to **general procedure C** the title compound (9.00 mg, 31.9 µmol, 16%) was obtained as a colorless oil.

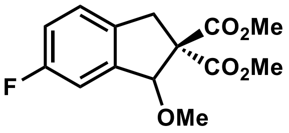

9g

$C_{14}H_{15}FO_5$

M= 282,27 g.mol-1

^{1}H NMR (500 MHz, $CDCl_3$): δ (ppm) 7.16 (dd, *J* = 8.3, 5.0 Hz, 1H), 7.06 (dd, *J* = 8.4, 2.5 Hz, 1H), 6.98 (td, *J* = 8.7, 2.5 Hz, 1H), 5.30 (s, 1H), 3.86 (d, *J* = 16.4 Hz, 1H), 3.77 (s, 3H), 3.74 (s, 3H), 3.49 (s, 3H), 3.17 (d, *J* = 16.1 Hz, 1H).

^{13}C NMR (151 MHz, $CDCl_3$): δ (ppm) 171.3, 168.8, 162.3 (d, *J* = 244.7 Hz), 142.1 (d, *J* = 7.7 Hz), 135.6 (d, *J* = 2.6 Hz), 126.0 (d, *J* = 8.5 Hz), 116.4 (d, *J* = 22.6 Hz), 112.3 (d, *J* = 22.5 Hz), 87.0 (d, *J* = 2.2 Hz), 66.6, 58.4, 53.1, 52.9, 37.9.

^{19}F NMR (376 MHz, $CDCl_3$): δ (ppm) -115.9

IR (neat): ν (cm^{-1}) 2954, 2842, 1735, 1436, 1243, 1120, 1087, 650

HRMS (ESI): Calcd for $C_{14}H_{15}FNaO_5$ $[M+Na]^+$: 305.0796, found 305.0801

Rf: 0.31 (cyclohexane/EtOAc 90:10)

Dimethyl 6-fluoro-3-methyl-3,4-dihydronaphthalene-2,2(1H)-dicarboxylate (9΄h):

According to **general procedure C** using 0.1 mmol of substrate the title compound (11.5 mg, 41 µmol, 41%) was obtained as a colorless oil.

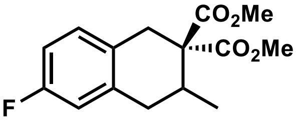

9'h

$C_{15}H_{17}FO_4$

M = 280.29 g.mol-1

^{1}H NMR (500 MHz, $CDCl_3$): δ (ppm) 7.06 (dd, *J* = 8.5, 5.8 Hz, 1H), 6.81 (td, *J* = 8.5, 2.7 Hz, 1H), 6.74 (dd, *J* = 9.5, 2.7 Hz, 1H), 3.71 (s, 3H), 3.70 (s, 3H), 3.27 (d, *J* = 3.1 Hz, 2H), 3.07 (dd, *J* = 17.4, 5.7 Hz, 1H), 2.74 (m, 1H), 2.62 (dd, *J* = 17.3, 6.1 Hz, 1H), 1.07 (d, *J* = 6.9 Hz, 3H).

^{13}C NMR (126 MHz, $CDCl_3$): δ (ppm) 171.72, 170.92, 161.44 (d, *J* = 243.6 Hz), 136.31 (d, *J* = 7.3 Hz), 130.16 (d, *J* = 8.2 Hz), 128.70, 115.26 (d, *J* = 20.7 Hz), 113.24 (d, *J* = 21.3 Hz), 57.64, 52.86, 52.65, 32.48, 16.94.

^{19}F NMR (235 MHz, $CDCl_3$): δ (ppm) -117.3

IR (neat): ν (cm^{-1}) 2955, 1733, 1615, 1502, 1435, 1258, 1215, 1042, 869, 632

HRMS (ESI): Calcd for $C_{15}H_{17}FNaO_4$ $[M+Na]^+$: 303.1003, found 303.1000

Rf: 0.20 (cyclohexane/EtOAc 95:5)

6. Kinetic experiments

6.1. General procedure for kinetic experiments

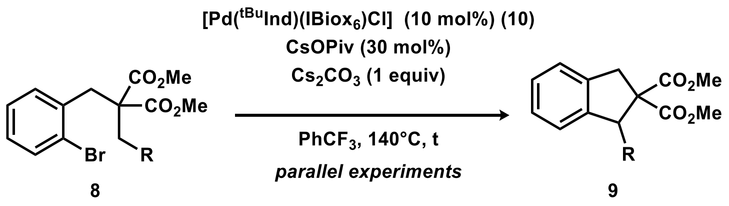

Inside a glovebox, CsOpiv (14.0 mg, 60 µmol, 0.3 eq.) and Cs_2CO_3 (65.2 mg, 0.2 mmol, 1.0 eq) were weighted 7 times and charged in 7 different catalysis tubes individually. Stock solutions of [Pd(^{tBu}Ind)(IBiox6)Cl] (20 µmmol/mL) and substrate (200 µmol/mL) in CF_3Ph were prepared and 1 mL of each was added in each tube to reach a concentration of 0.1 M. The tubes were capped, taken out of the glovebox and inserted in a preheated metal block at 140 °C. The reactions were stopped at different times and immediately cooled to 24 °C by inserting the tubes into another metal block. A stock solution of the standard (200 µmol/mL) in CF_3Ph was prepared and 1 mL was added to each tube after the reaction. The mixture was filtered over a short pad of silica and analysed by ^{19}F NMR against the standard. Kinetic orders determined using the VTNA method developed by Bures.

These conditions provided excellent reproducibility of kinetic data as shown below with substrate **8a**.

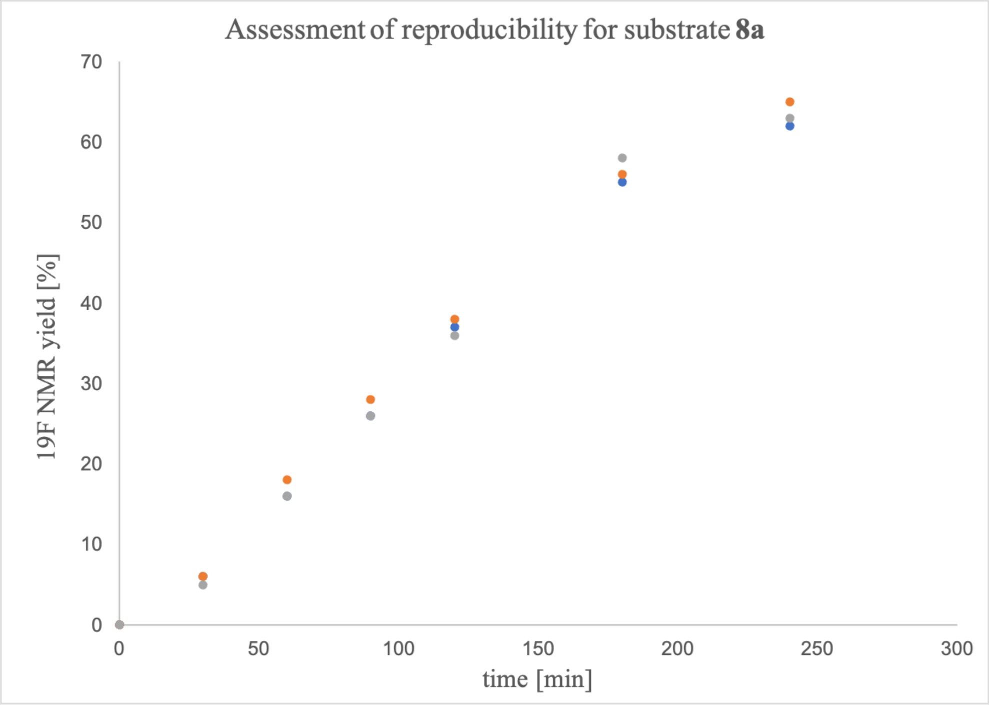

6.2. Initial rate experiments

6.2.1. 8a

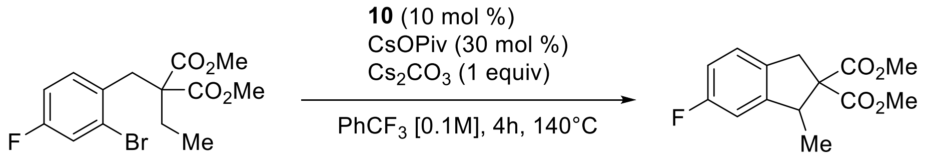

8a
0.2 mmol

9a

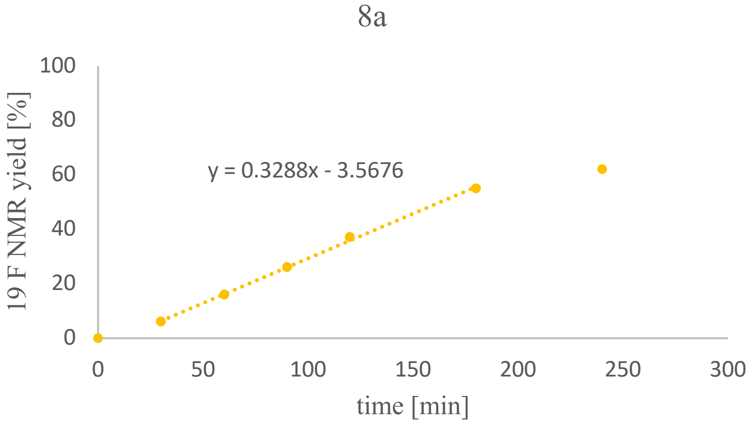

Time [min]	^{19}F NMR yield [%]
30	6
60	16
90	26
120	37
180	55
240	62

6.2.2. 8a-*d5*

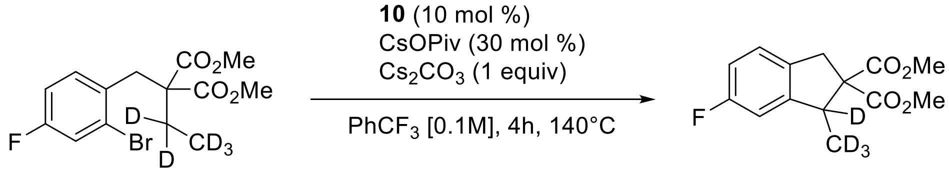

8a-*d5*
0.2 mmol

9a-*d4*

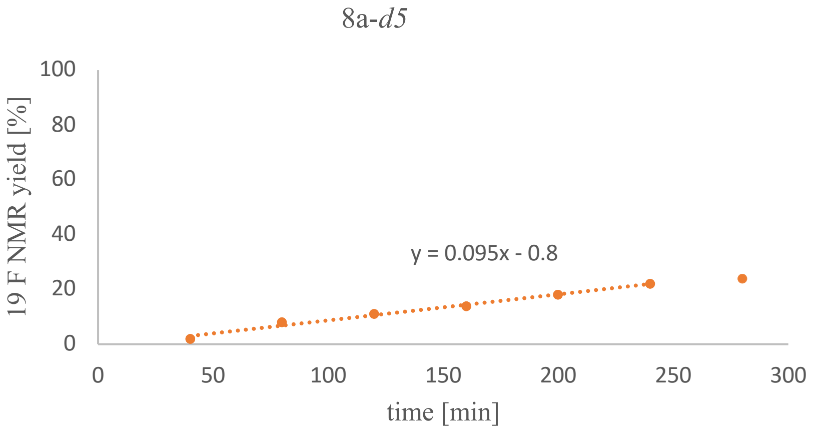

Time [min]	^{19}F NMR yield [%]
40	2
80	8
120	11
160	14
200	18
240	22
280	24

6.2.3. 8b

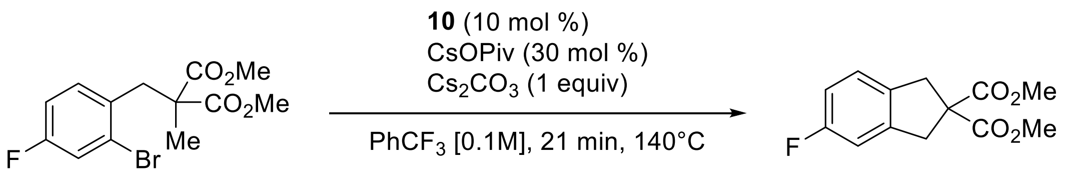

8b
0.2 mmol

9b

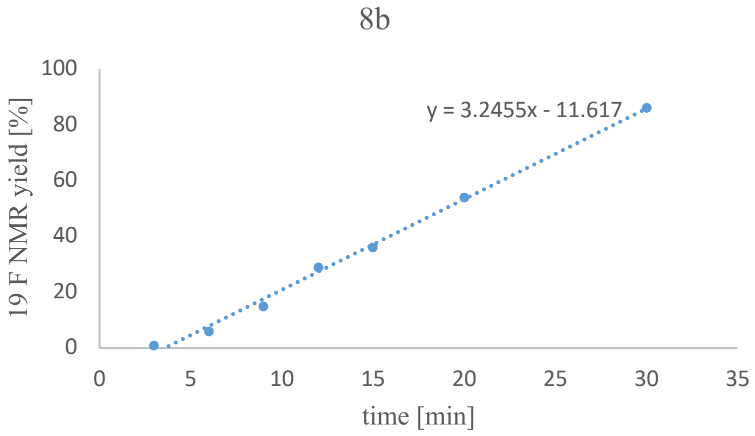

Time [min]	^{19}F NMR yield [%]
3	1
6	6
9	15
12	29
15	36
20	54
30	86

6.2.4. 8c

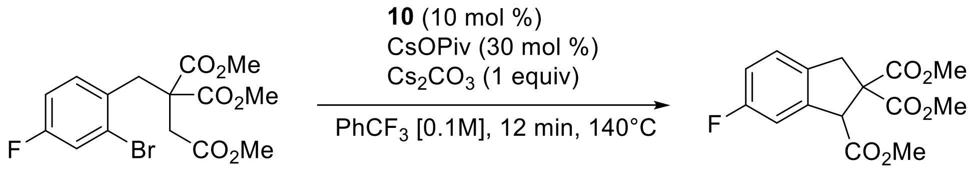

8c
0.2 mmol

9c

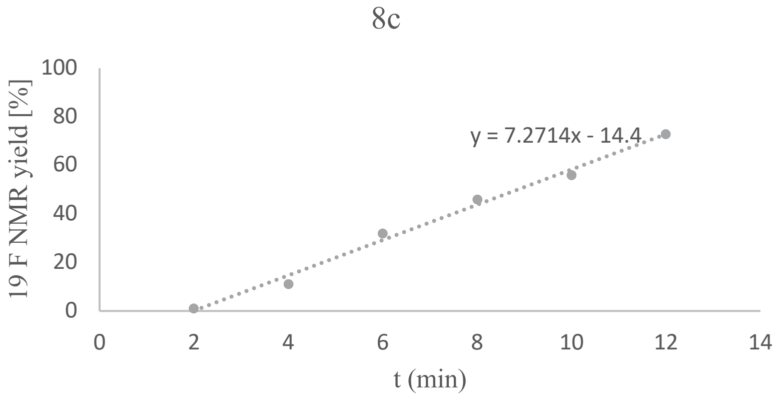

Time [min]	^{19}F NMR yield [%]
2	1
4	11
6	32
8	46
10	56
12	73

6.2.5. 8d

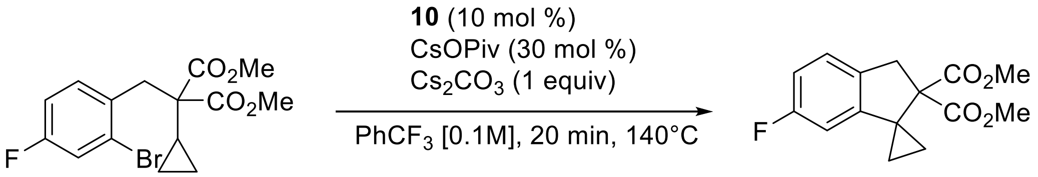

8d
0.2 mmol

9d

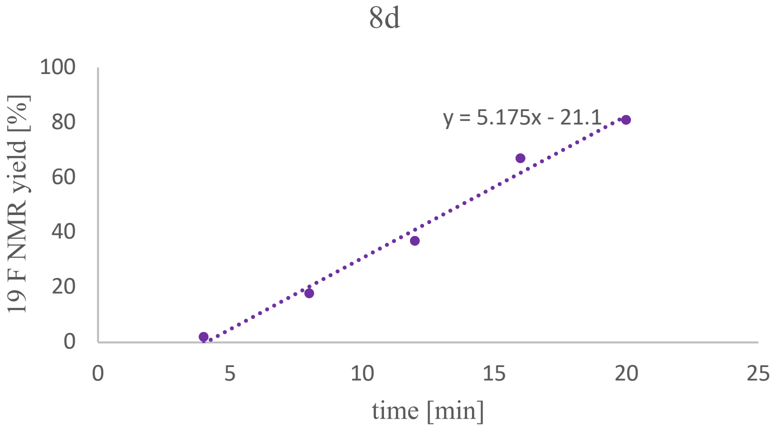

Time [min]	^{19}F NMR yield [%]
4	2
8	18
12	37
16	67
20	81

6.2.6. 8e

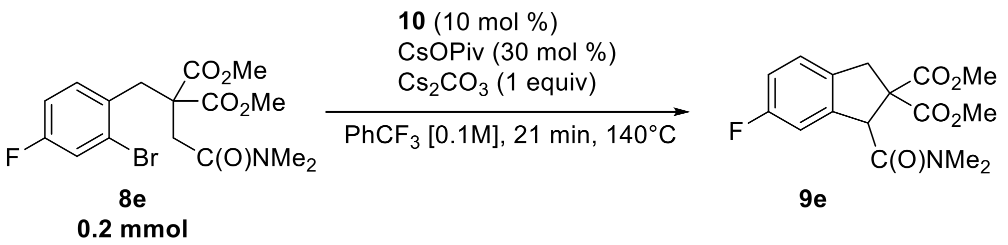

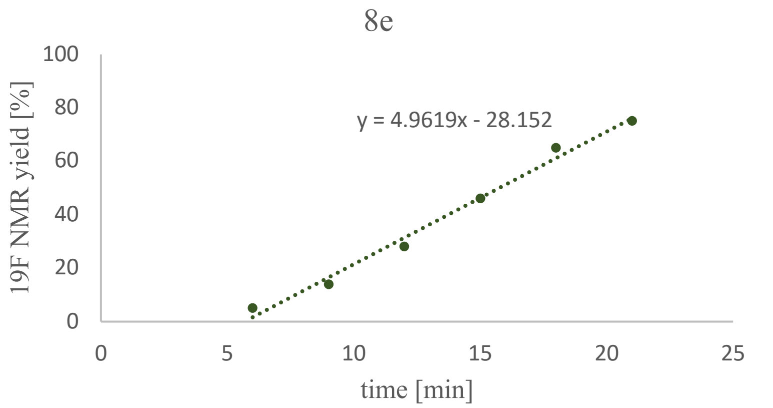

Time [min]	^{19}F NMR yield [%]
6	5
9	14
12	28
15	46
18	65
21	75

6.2.7. 8f

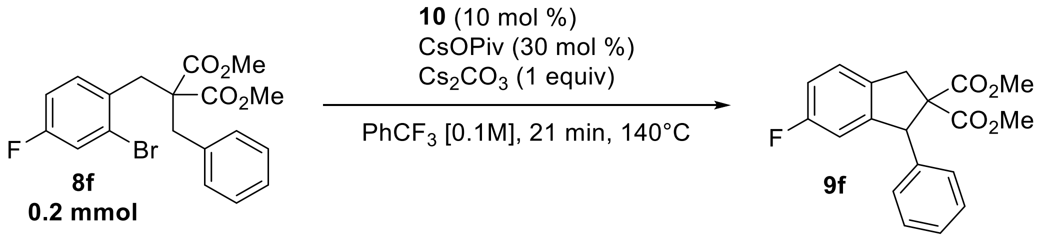

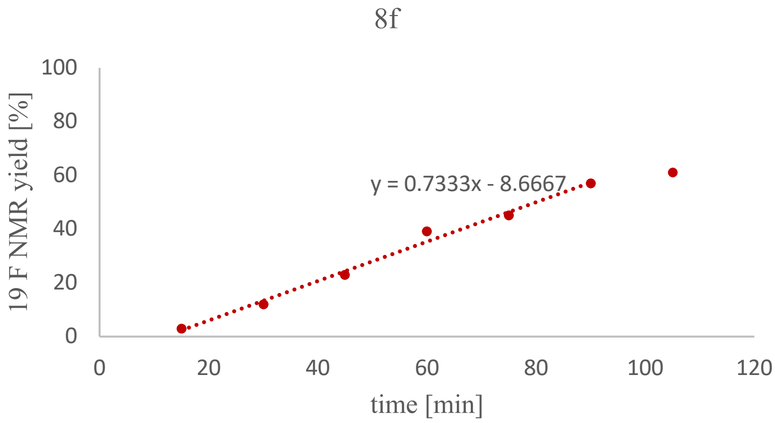

Time [min]	^{19}F NMR yield [%]
15	3
30	12
45	23
60	39
75	45
90	57
105	61

6.2.8. 8g

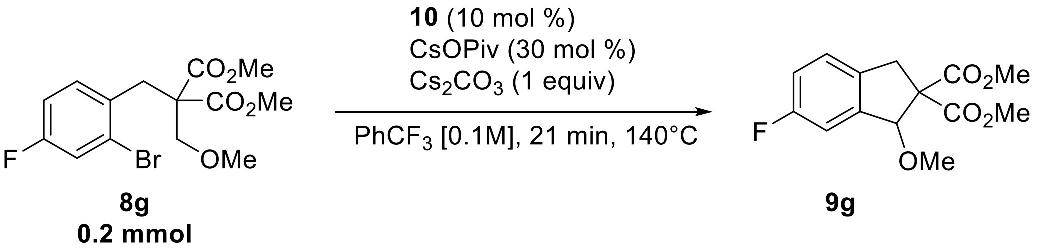

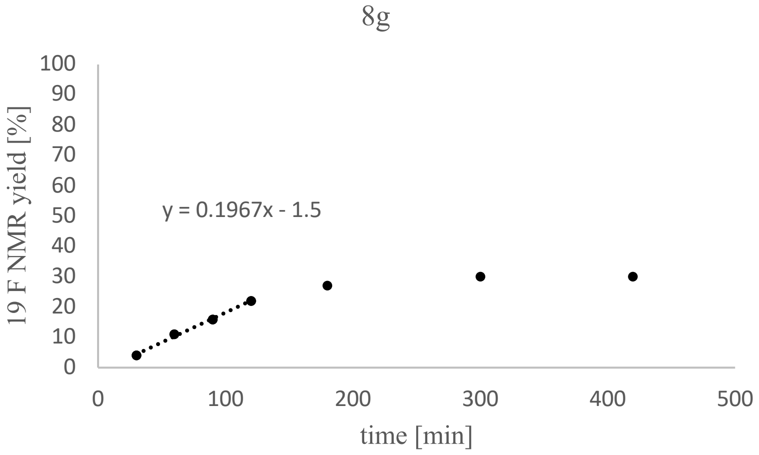

Time [min]	^{19}F NMR yield [%]
30	4
60	11
90	16
120	22
180	27
300	30
420	30

6.3. Kinetic analysis

6.3.1. Order on aryl bromide

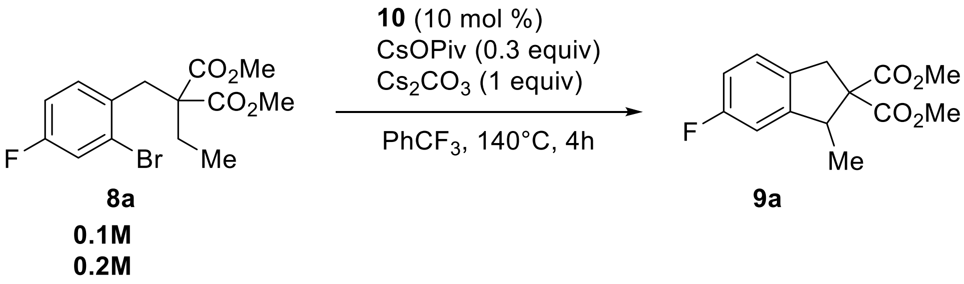

The overlay clearly shows 0 order on aryl bromide

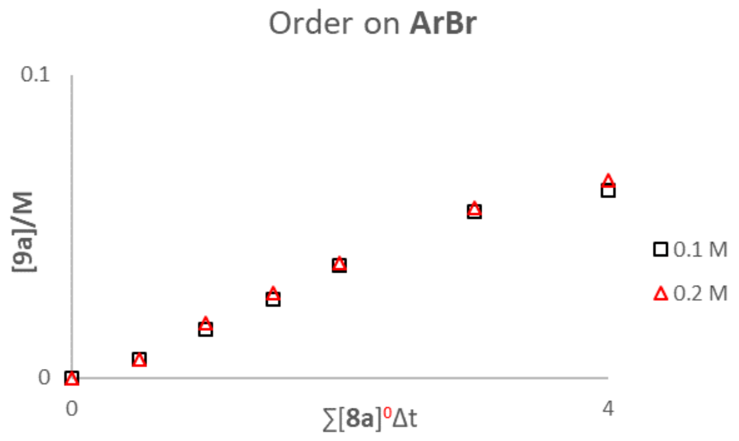

Figure S1. VTNA order for ArBr

Time (min)	**9a (0.1M)**	**9a (0.2M)**
0	0	0
30	6	6
60	16	18
90	26	28
120	37	38
180	55	56
240	62	65

Reaction data for kinetic runs to determine order on aryl bromide **8a**. Yield determined by ^{19}F NMR yield using fluorobenzene as internal standard.

6.3.2. Order on catalyst

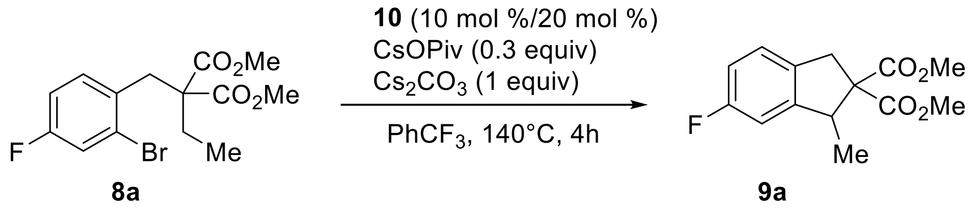

The overlay shows a first order dependency in catalyst **10** at these concentrations. This suggests that a mononuclear palladium species is the active catalyst.

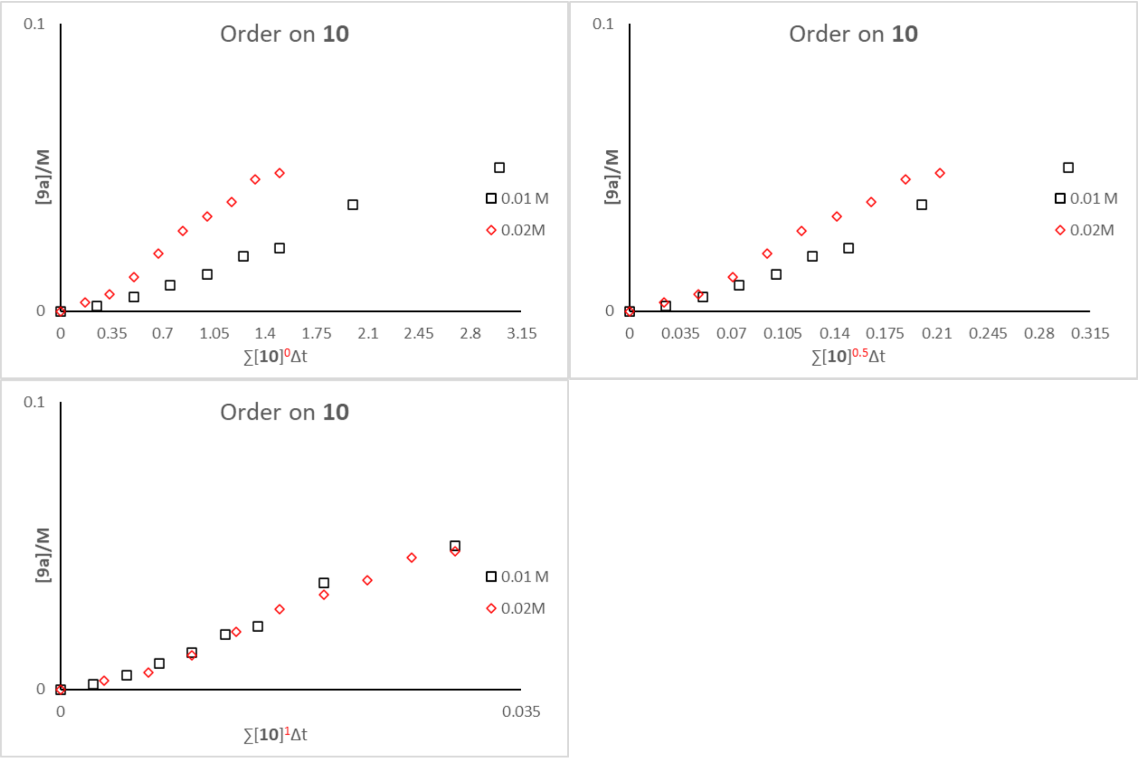

Figure S2. VTNA order for catalyst **10.** Top left: 0 order on catalyst. Top right: 0.5 order on catalyst. Bottom left: 1st order on catalyst

Time (min)	**9a (0.01M)**	**9a (0.02M)**
0	0	0
10	-	3
15	2	-
20	-	6
30	5	12
40	-	20
45	9	-
50	-	28
60	13	33
70	-	38
75	19	-
90	22	48
120	37	-
180	50	-

Reaction data for kinetic runs to determine order on catalyst **10**. Yield determined by ^{19}F NMR yield using fluorobenzene as internal standard.

6.3.3. Order on CsOPiv

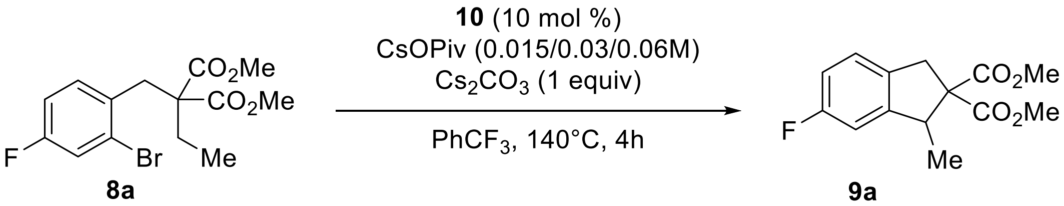

The overlay clearly shows 0 order on CsOPiv. However, this could be due to the low solubility of this species in the reaction medium.

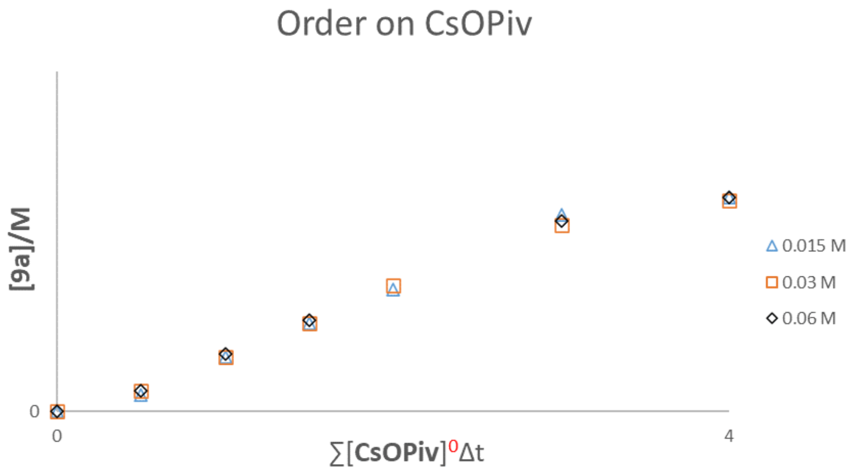

Figure S3. VTNA order for CsOPiv

Time (min)	**9a (0.015)**	**9a (0.03M)**	**9a (0.06M)**
0	0	0	0
30	5	6	6
60	16	16	17
90	26	26	27
120	36	37	-
180	58	55	56
240	63	62	63

Reaction data for kinetic runs to determine order on CsOPiv. Yield determined by ^{19}F NMR yield using fluorobenzene as internal standard.

6.3.4. Order on Cs_2CO_3

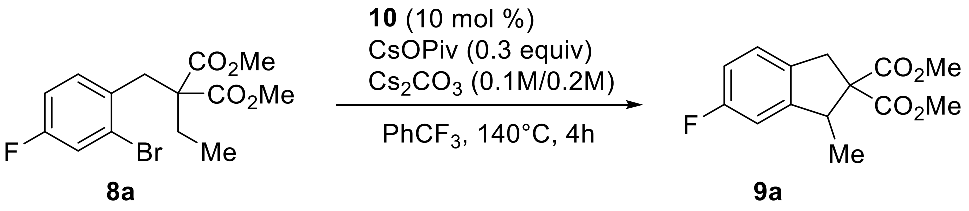

The overlay clearly shows 0 order on Cs_2CO_3. However, this could be due to the low solubility of this species in the reaction medium.

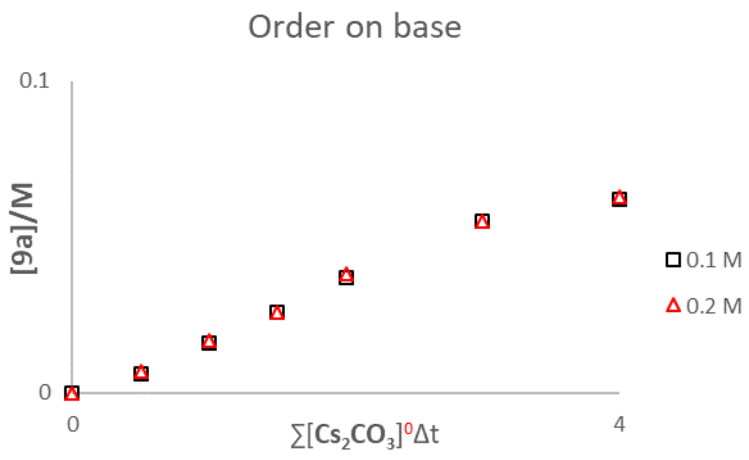

Figure S4. VTNA order for Cs_2CO_3

Time (min)	**9a (0.01M)**	**9a (0.02M)**
0	0	0
30	6	7
60	16	17
90	26	26
120	37	38
180	55	55
240	62	63

Reaction data for kinetic runs to determine order on Cs_2CO_3. Yield determined by ^{19}F NMR yield using fluorobenzene as internal standard.

6.3.5. Kinetic Isotope Effects

6.3.5.1 CH_2CH_3/CD_2CD_3

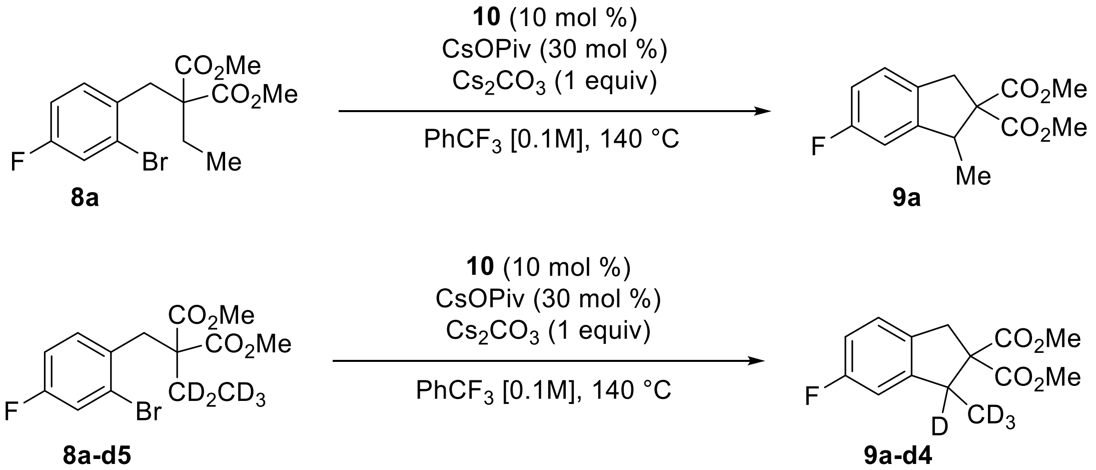

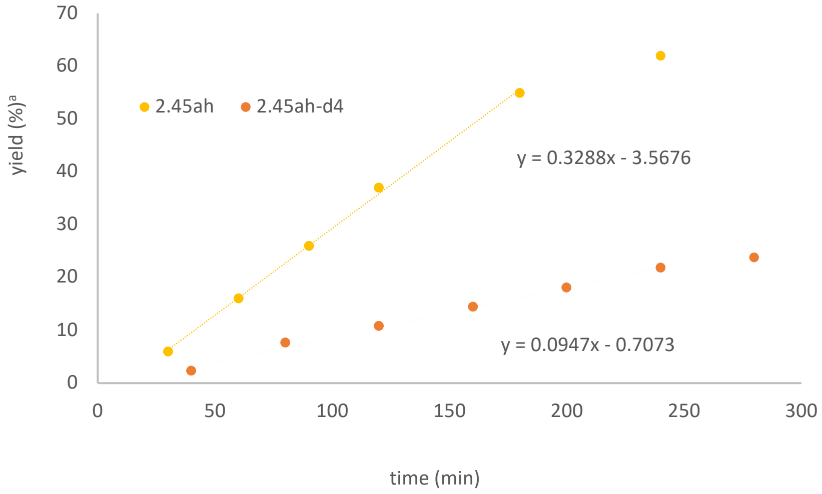

Figure S5. Kinetic Isotope Effect for **8a**/**8a-d5**. $k_H/k_D = 3.5$, suggesting that C–H activation is rate-limiting for this substrate. Determined by ^{19}F NMR using fluorobenzene as external standard.

Time for 8a (min)	CH_2CH_3	CD_2CD_3	Time for 8a-d5 (min)
0	0	0	0
30	6	2.35	40
60	16	7.7	80
90	26	10.82	120
120	37	14.43	160
180	55	18.11	200
240	62	21.9	240

6.3.5.2 CH_3/CD_3

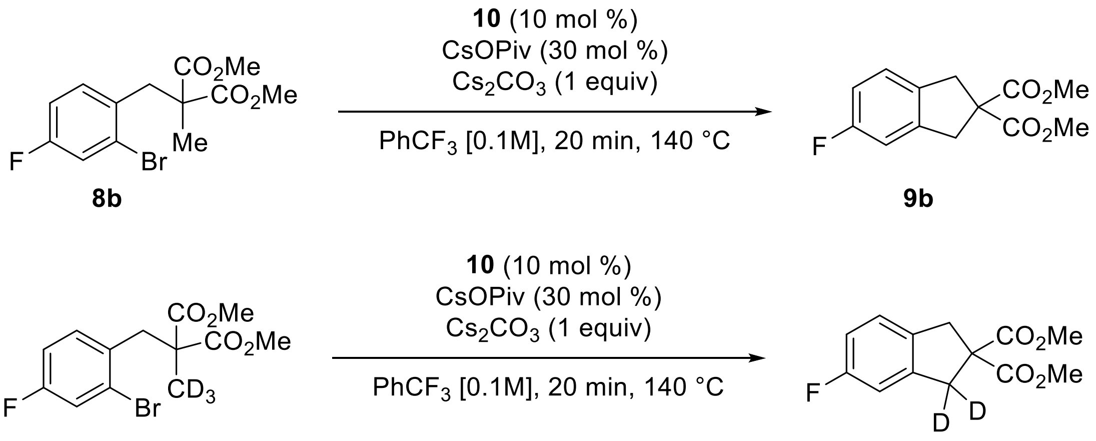

8b-d3 **9d-d2**

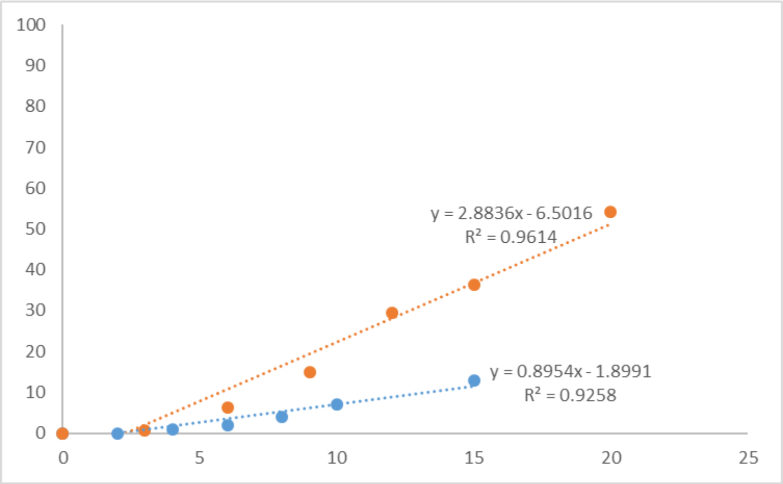

Figure S6. Kinetic Isotope Effect for **8b**/**8b-d3**. k_H/k_D = 3.2, suggesting that for this substrate C–H activation is the rate-limiting step of the reaction. Determined by ^{19}F NMR using fluorobenzene as external standard.

Time (min)	CD_3	CH_3
0	0	0
2	0	-
3	-	1
4	1	-
6	2	6
8	4	-
9	-	15
10	8	-
15	14	36
20	-	54

6.3.5.3 CH_2CO_2Me/CD_2CO_2Me

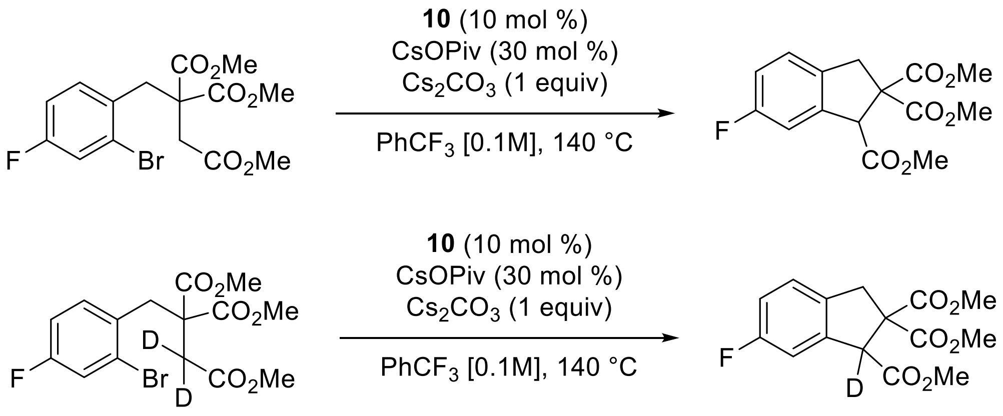

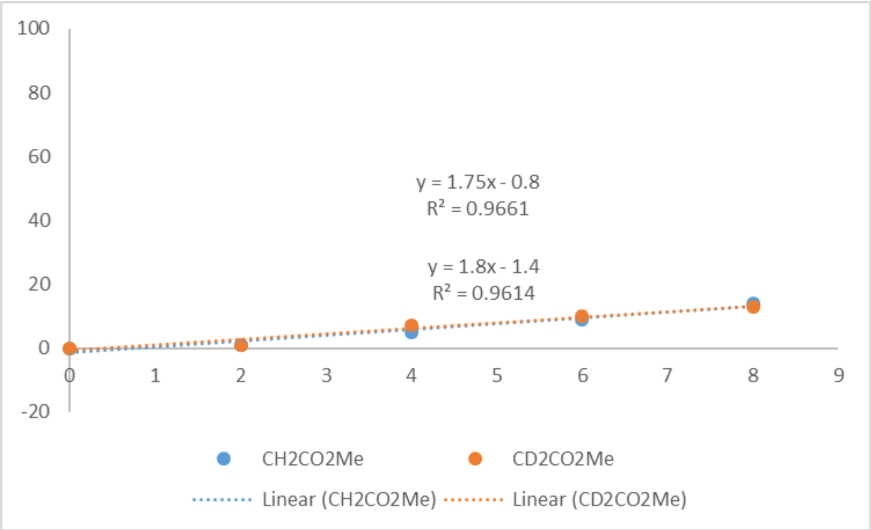

Figure S7. Kinetic Isotope Effect for **8c**/**8c-d2**. In the initial reaction period, k_H/k_D ~1, suggesting that the C–H activation is not rate-limiting for this substrate. This is supported by our calculations. Determined by ^{19}F NMR using fluorobenzene as external standard.

Time (min)	CD_2CO_2Me	CH_2CO_2Me
0	0	0
2	1	2
4	6	7
6	10	9
8	12	13

6.3.6. Catalyst activation studies

The activation of the catalyst is a key step in the reaction. We propose that CsOPiv plays a role in activating this complex.

6.3.6.1 Reaction of catalyst with CsOPiv

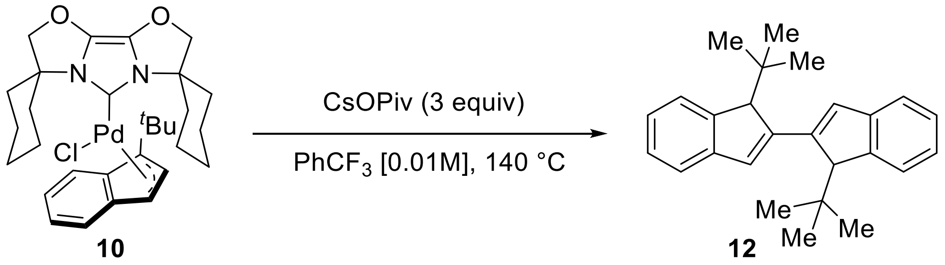

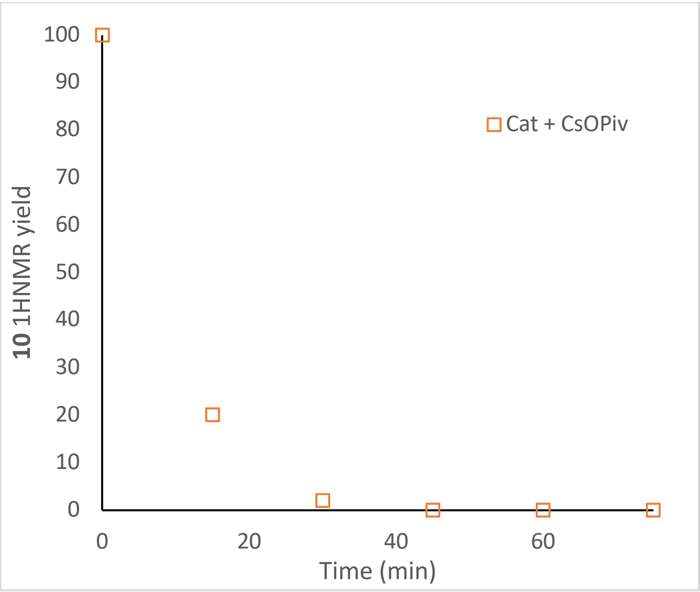

Figure S8. Catalyst decomposition study with Cat **10** + CsOPiv. Yield determined by ^{1}H NMR using 1,3,5-trimethoxybenzene as internal standard.

6.3.6.2 Reaction of catalyst with Cs_2CO_3

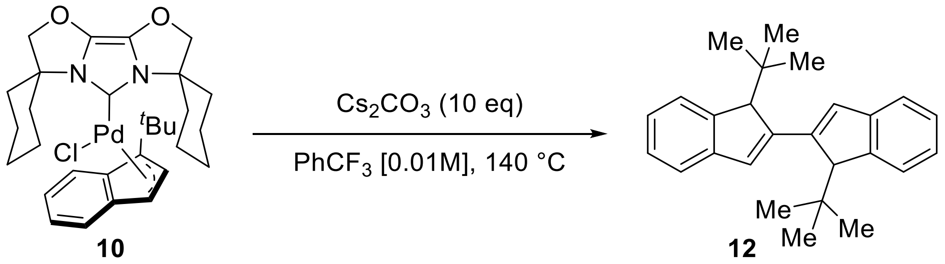

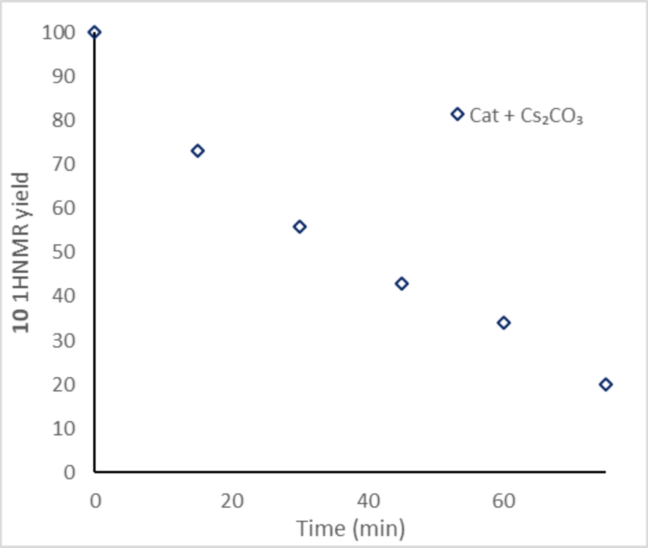

Figure S9. Catalyst decomposition study with Cat **10** + Cs_2CO_3. Yield determined by ^{1}H NMR using 1,3,5-trimethoxybenzene as internal standard.

6.3.6.3 Reaction of catalyst with CsOPiv and Cs_2CO_3

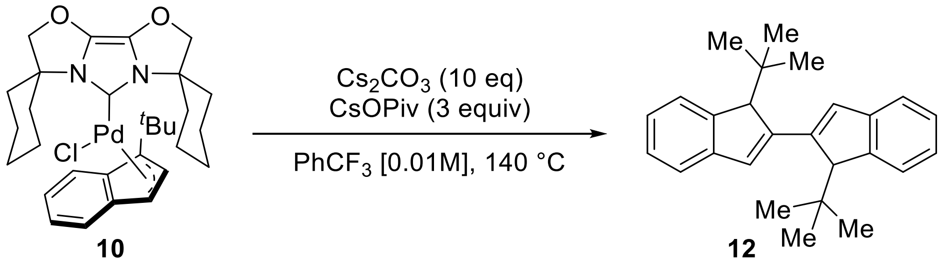

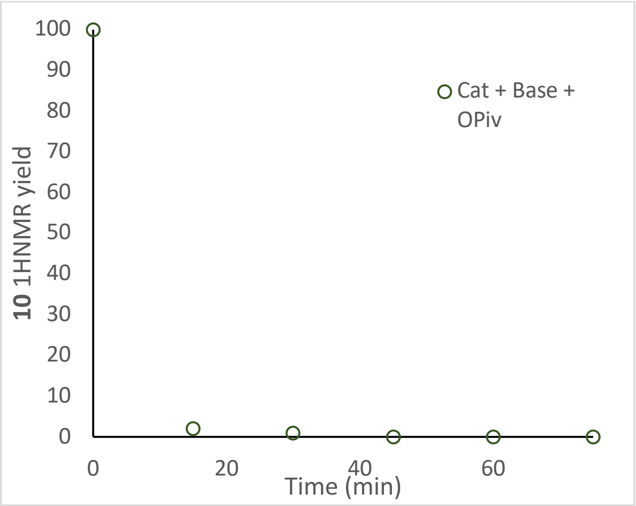

Figure S10. Catalyst decomposition study with Cat **10** + CsOPiv + Cs_2CO_3. Yield determined by ^{1}H NMR using 1,3,5-trimethoxybenzene as internal standard.

6.3.6.4 Product characterization for bis-indene

Compound 12 isolated as a 2:1 mixture of diastereomers

12 Major- Catalyst **10** was heated in solution ($PhCF_3$ [0.01M]) at 140°C for 40 minutes. The compound was loaded onto silica and columned using flash techniques to yield the pure product in 17% yield as a yellow wax. Compound yield is low due to requiring multiple purification attempts to yield the pure product.

^{1}H NMR (500 MHz, CD_2Cl_2) δ 7.49 (d, *J* = 7.7 Hz, 2H), 7.20 (td, *J* = 7.6, 1.2 Hz, 2H), 6.96 (td, *J* = 7.4, 1.0 Hz, 2H), 6.78 (d, *J* = 7.5 Hz, 2H), 5.93 (s, 2H), 3.97 (s, 2H), 1.21 (s, 18H).

^{13}C NMR (126 MHz, CD_2Cl_2) δ 153.9, 147.7, 144.5, 129.5, 126.5, 124.3, 123.5, 122.5, 49.6, 33.4, 29.6

HRMS (ESI): Calcd for $C_{26}H_{30}Ag$: 449.1393, found 449.1393

IR (neat): ν (cm^{-1}) 3727, 2962, 2361, 1706, 1461, 1203

Rf: 0.3 (Cyclohexane:EtOAc 90:10)

12 Minor Catalyst **10** was heated in solution ($PhCF_3$ [0.01M]) at 140°C for 40 minutes. The compound was loaded onto silica and columned using flash techniques to yield the pure product in 12% yield as a yellow wax. Compound yield is low due to requiring multiple purification attempts to yield the pure product.

^{1}H NMR (600 MHz, CD_2Cl_2) δ 7.54 – 7.53 (m, 2H), 7.52 (m, 2H), 7.24 (td, *J* = 7.6, 1.2 Hz, 2H), 7.13 (td, J = 7.4, 1.1 Hz, 2H), 5.58 (s, 2H), 4.01 (s, 2H), 1.26 (s, 18H).

^{13}C NMR (151 MHz, CD_2Cl_2) δ 153.4, 148.2, 144.2, 128.9, 126.4, 124.3, 123.3, 122.5, 49.6, 33.3, 29.4.

HRMS (ESI): Calcd for $C_{26}H_{30}Ag$: 449.1393, found 449.1385

IR (neat): ν (cm^{-1}) 2954, 2866, 2361, 1459, 1361, 1265

Rf: 0.29 (Cyclohexane:EtOAc 90:10)

6.3.7. Reaction in absence of CsOPiv

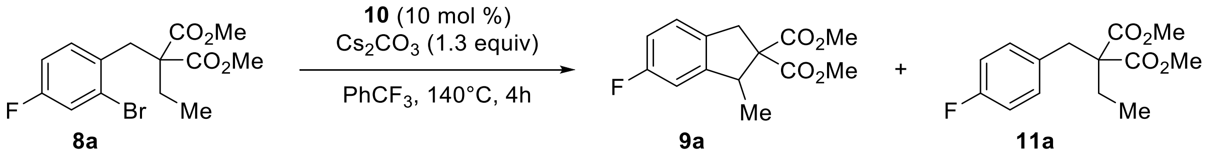

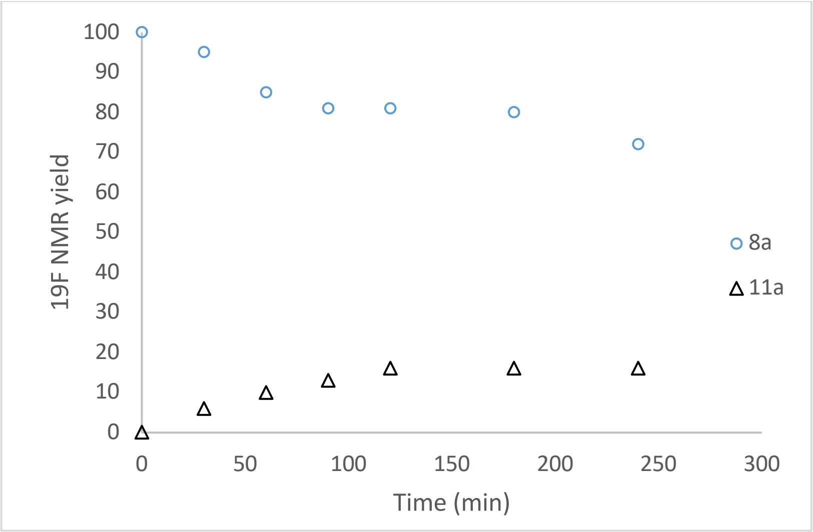

Figure S11. Reaction in the absence of CsOPiv

Formation of **9a** not detected in absence of CsOPiv. Only corresponding dehalogenation product **11a** was detected.

6.3.8. Reaction in absence of Cs_2CO_3

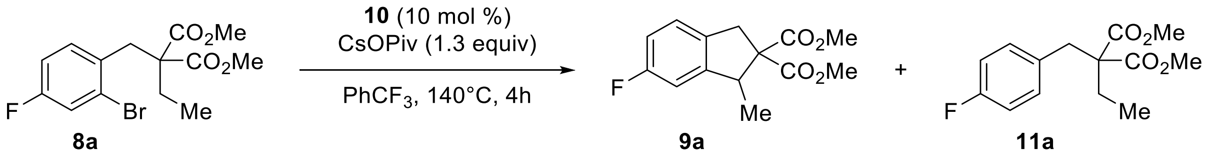

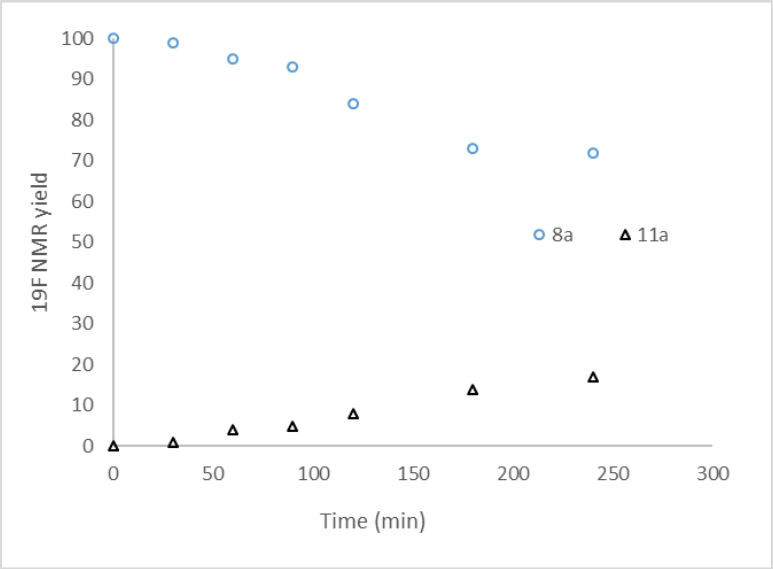

Figure S12. Reaction in the absence of Cs_2CO_3

Formation of **9a** not detected in absence of Cs_2CO_3. Only corresponding dehalogenation product **11a** was detected. This result (when taken with that from SI section 6.3.7. shows that for this reaction to be productive both bases are required)

6.3.9. Stirring rate effect on reaction

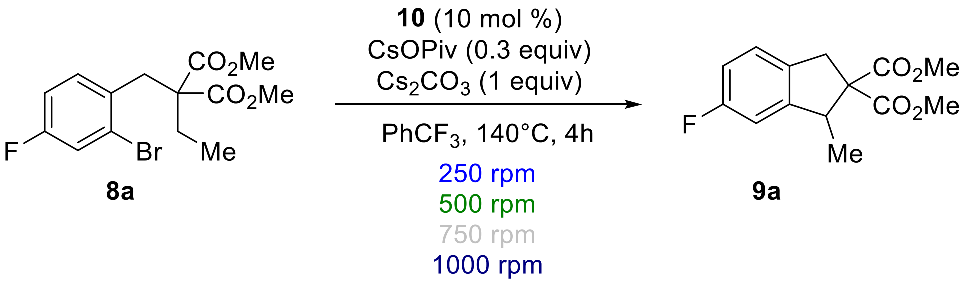

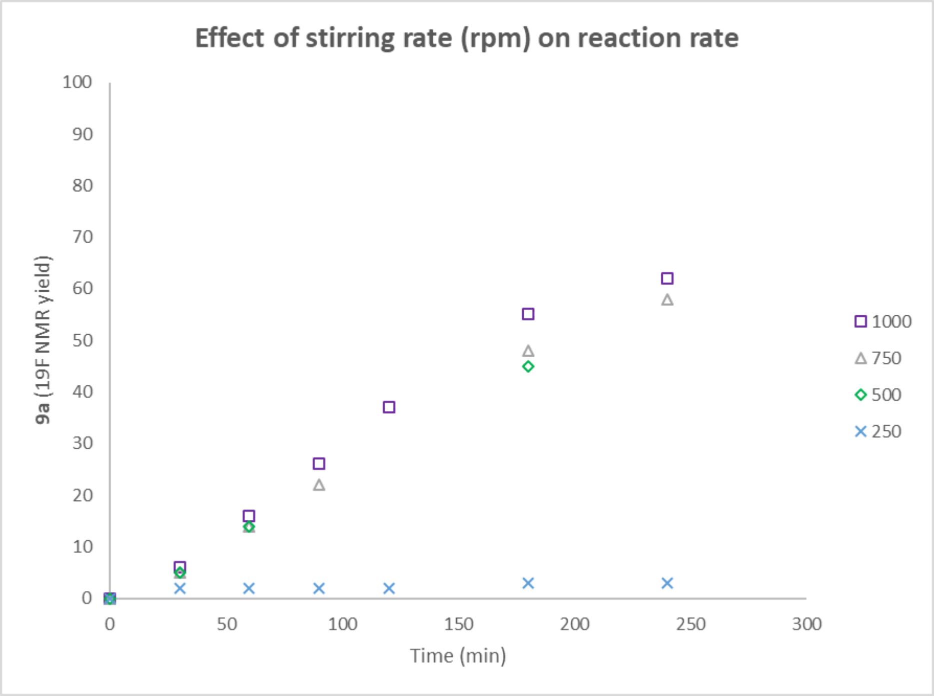

Figure S13. Effect of stirring rate on the reaction rate

The figure shows a clear dependency of the rate of the reaction on the stirring rate. This is consistent with a heterogeneous base being involved in a kinetically relevant step in the reaction. When the stirring is diminished to 250 rpm, the reaction is almost completely suppressed when compared with the standard stirring rate (1000 rpm).

7. Spectra

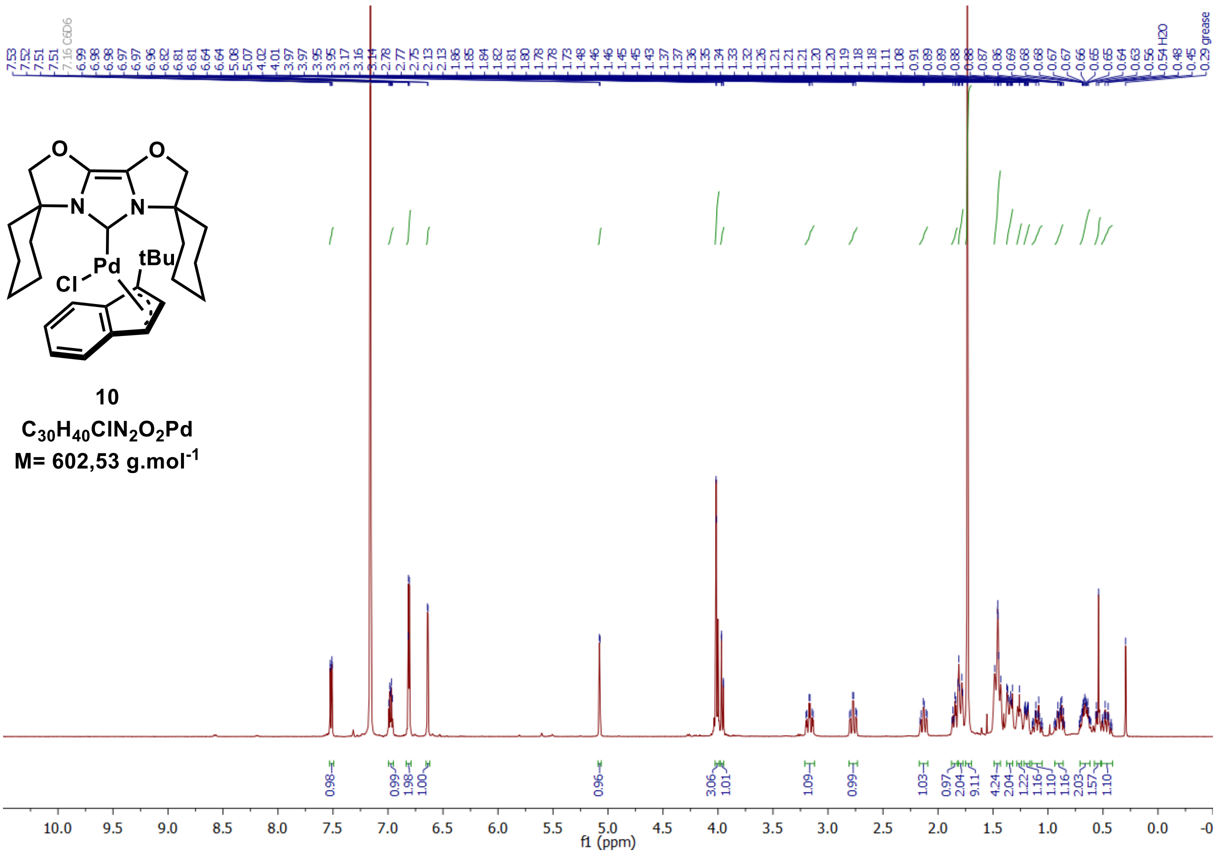

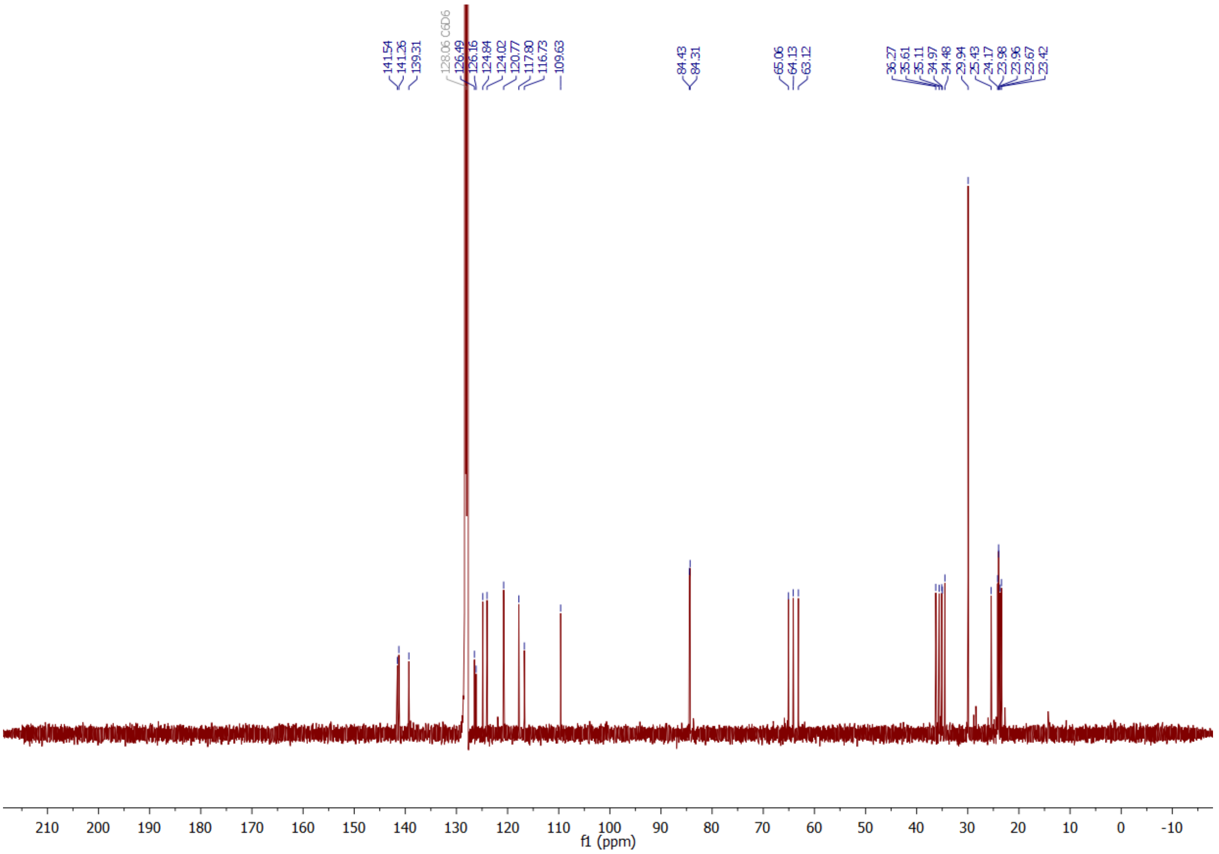

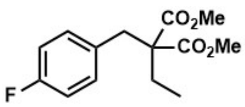

8a-dehalo

$C_{14}H_{17}FO_4$

M= 268,28 g.mol[-1]

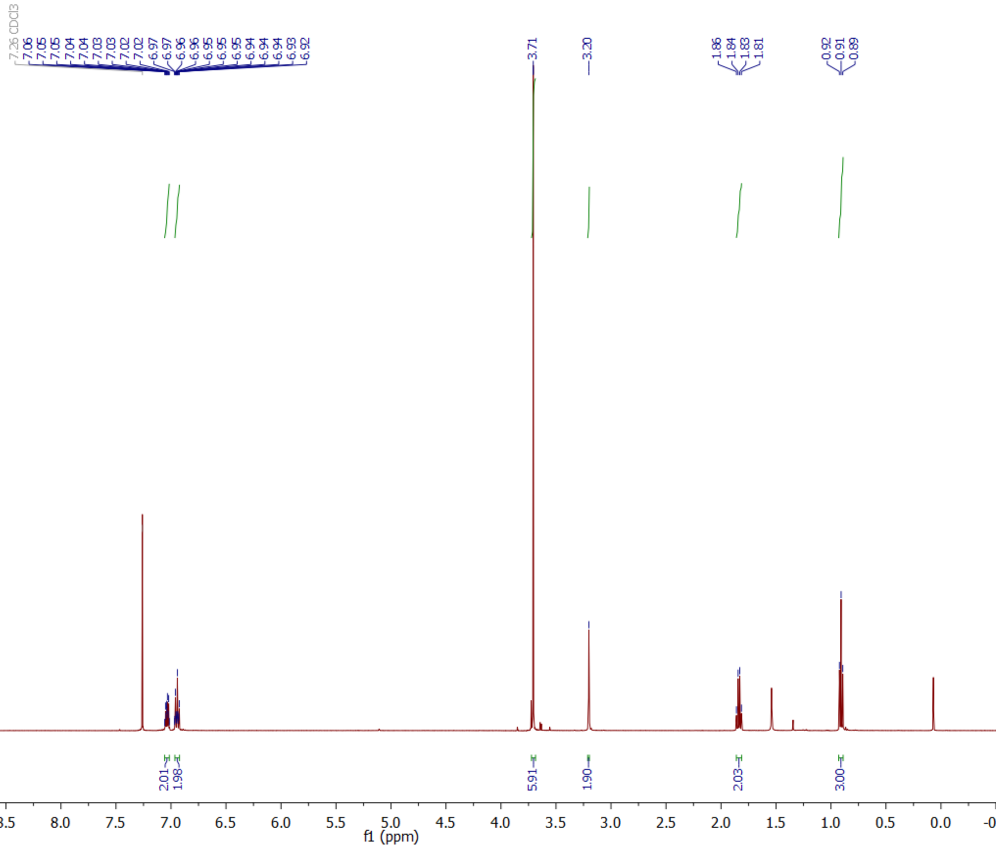

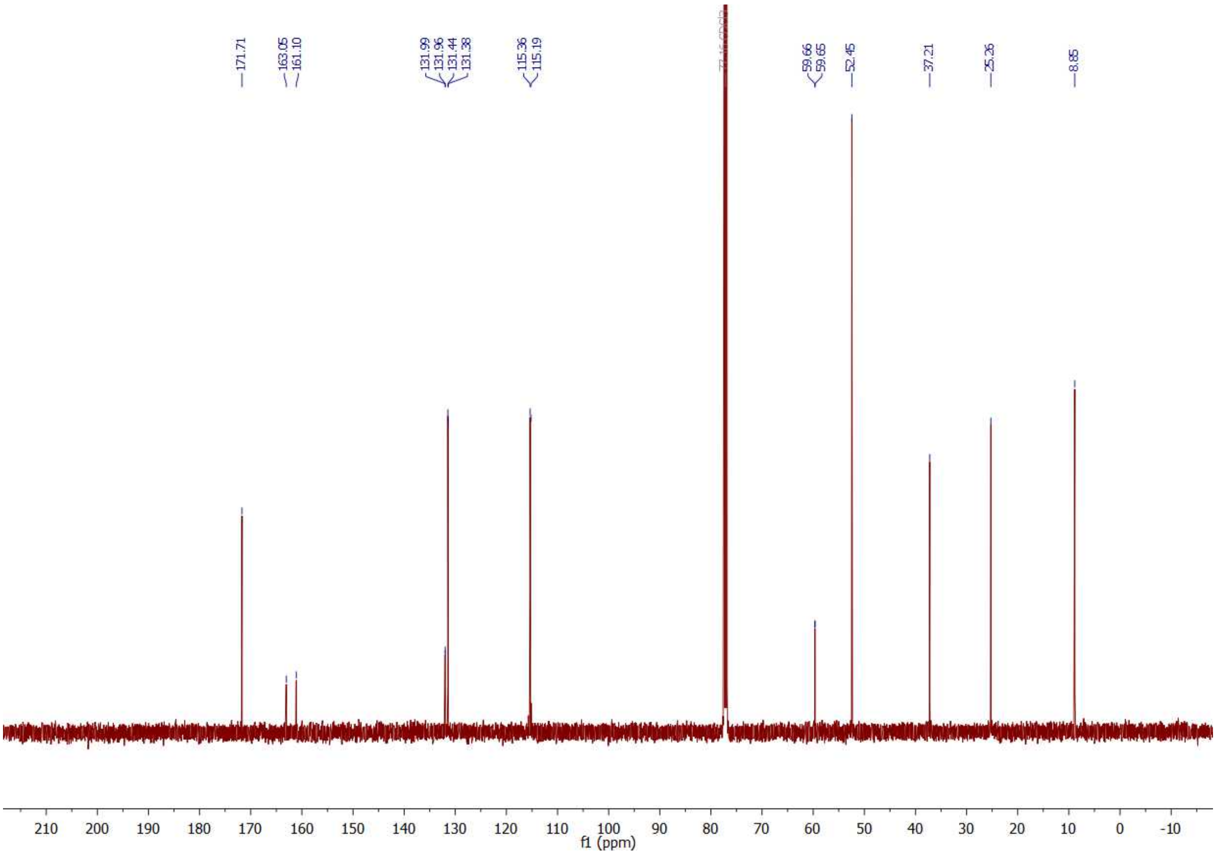

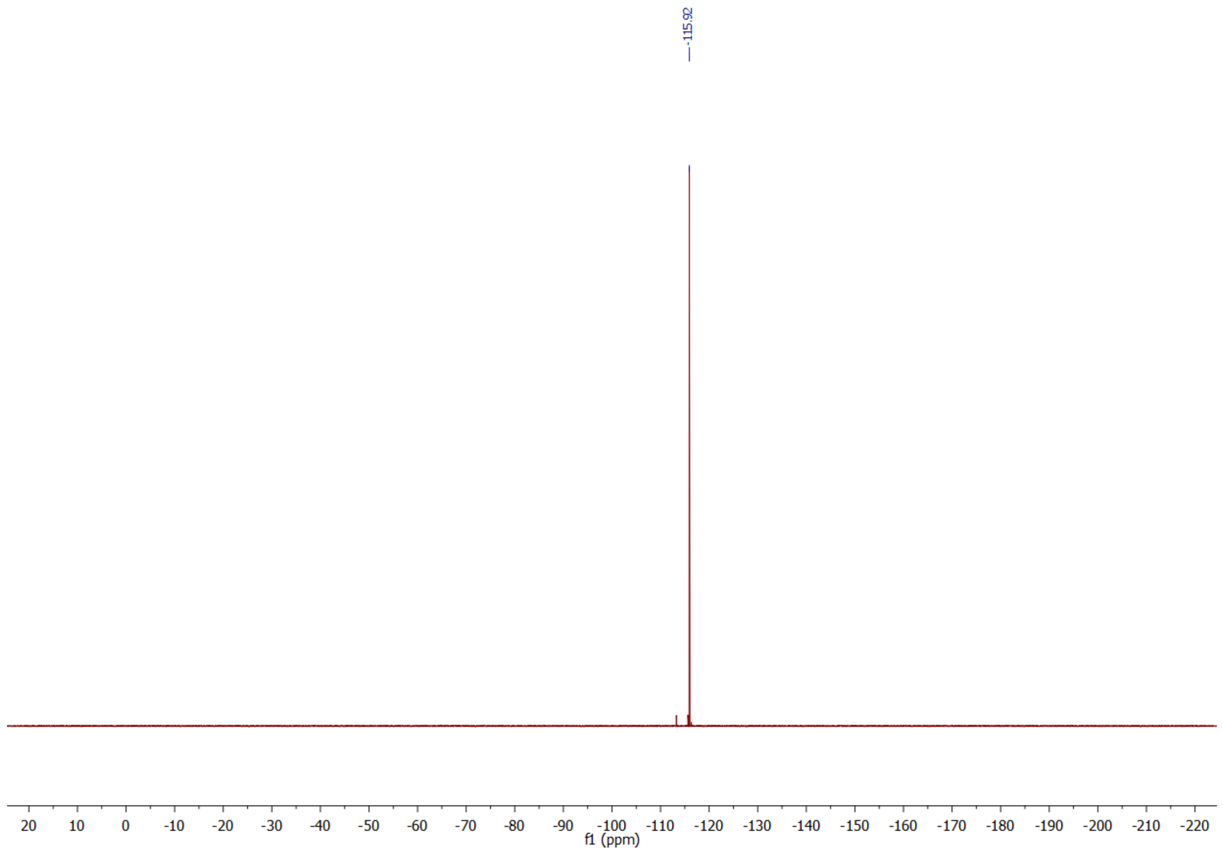
-115.92
20
10
0
-10
-20
-30
-40
-50
-60
-70
-80
-90
-100
-110
-120
-130
-140
-150
-160
-170
-180
-190
-200
-210
-220
f1 (ppm)

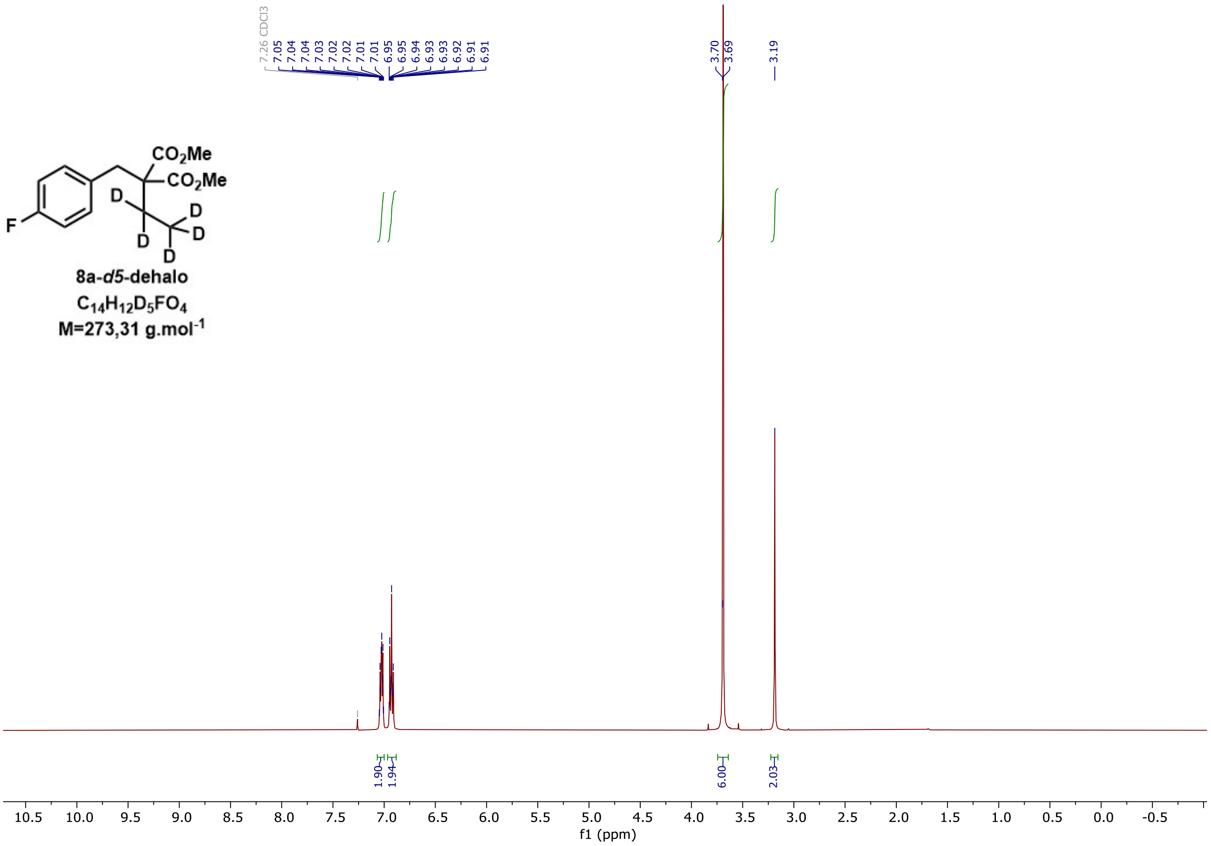

8a-*d5*-dehalo
$C_{14}H_{12}D_5FO_4$
M=273,31 g.mol^{-1}

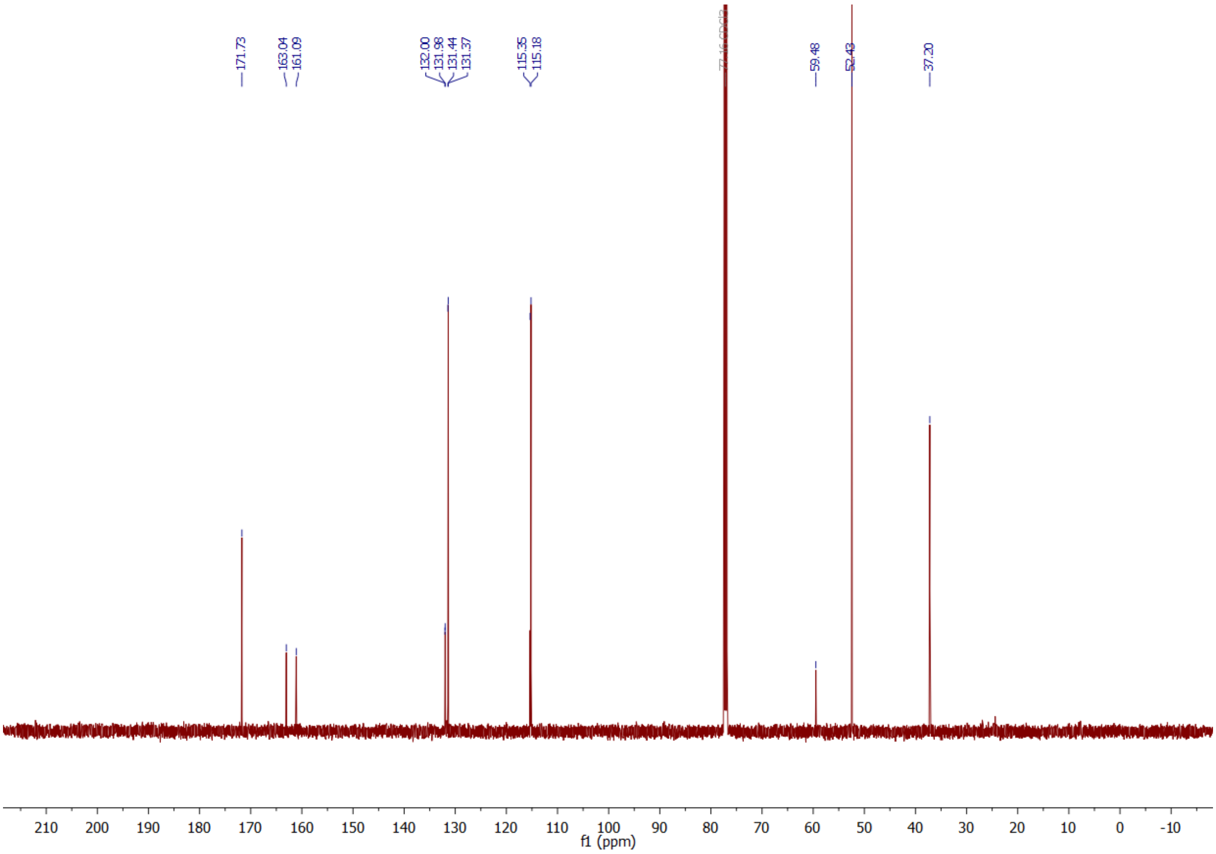

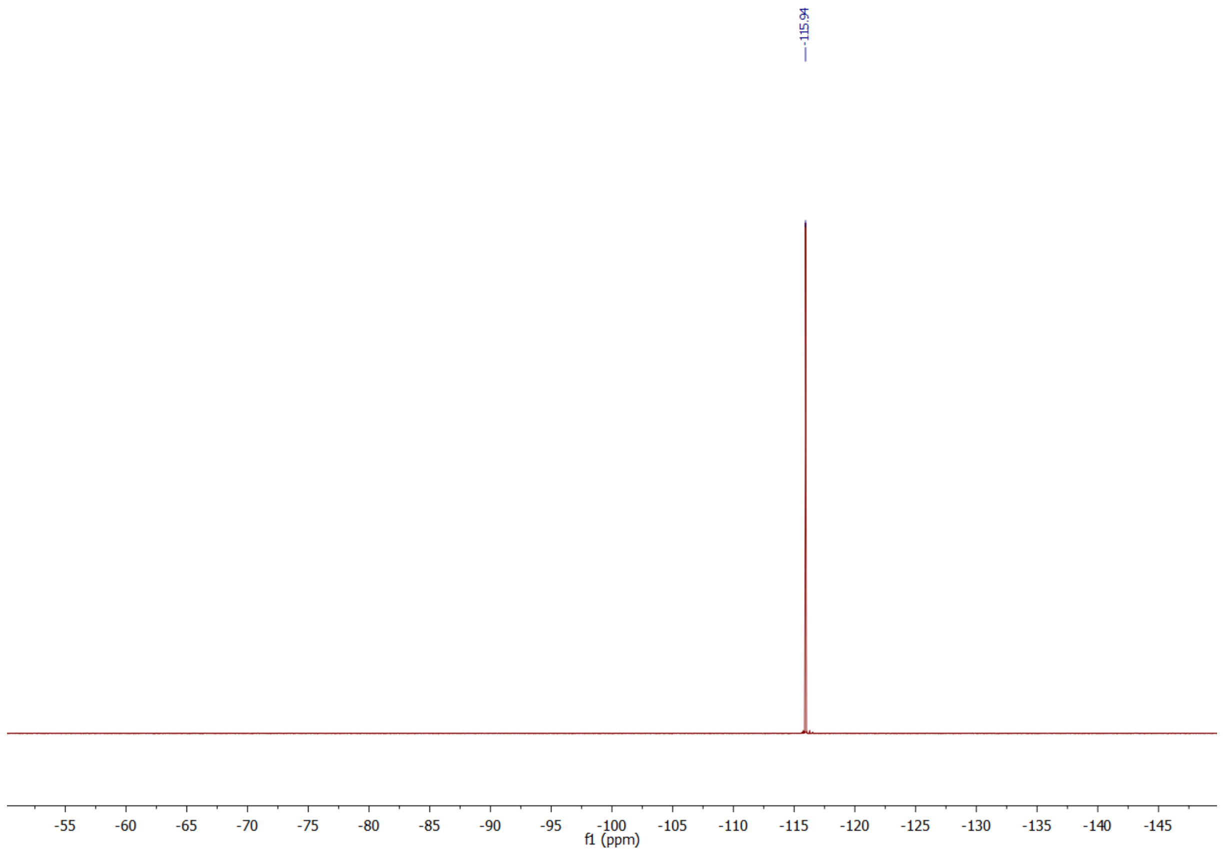
-115.94
-55
-60
-65
-70
-75
-80
-85
-90
-95
-100
-105
-110
-115
-120
-125
-130
-135
-140
-145
f1 (ppm)

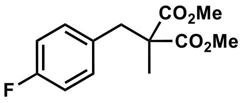

8b-dehalo
$C_{13}H_{15}FO_4$
M= 254,26 g.mol^{-1}

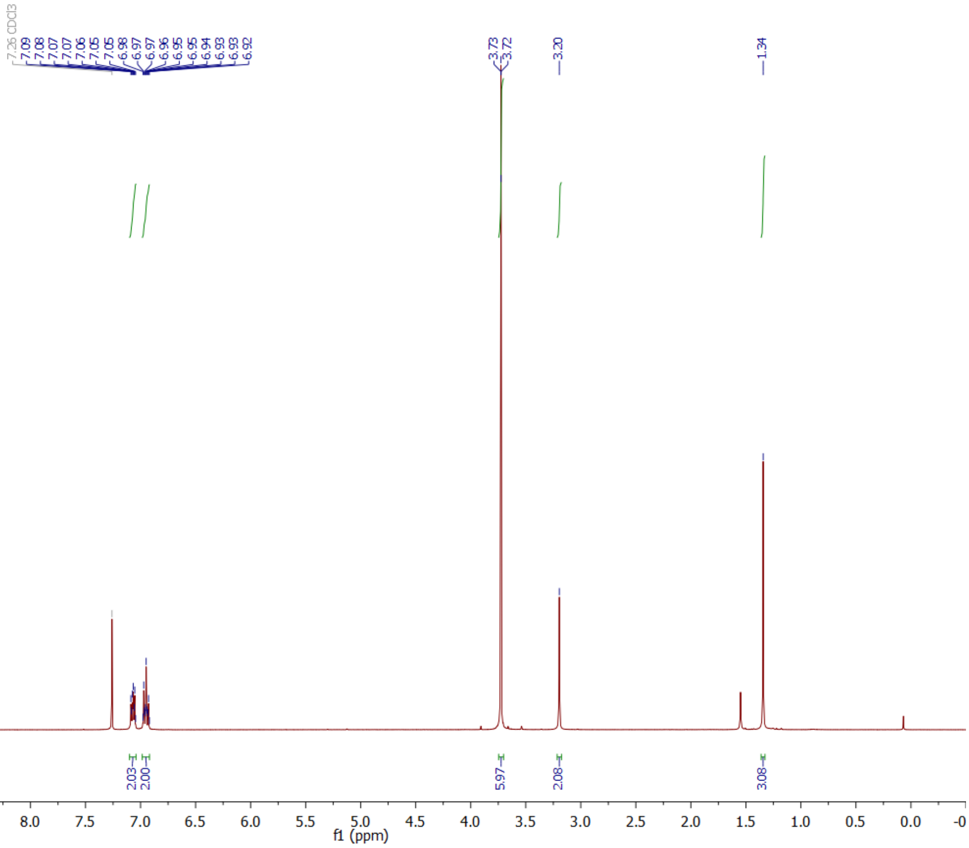

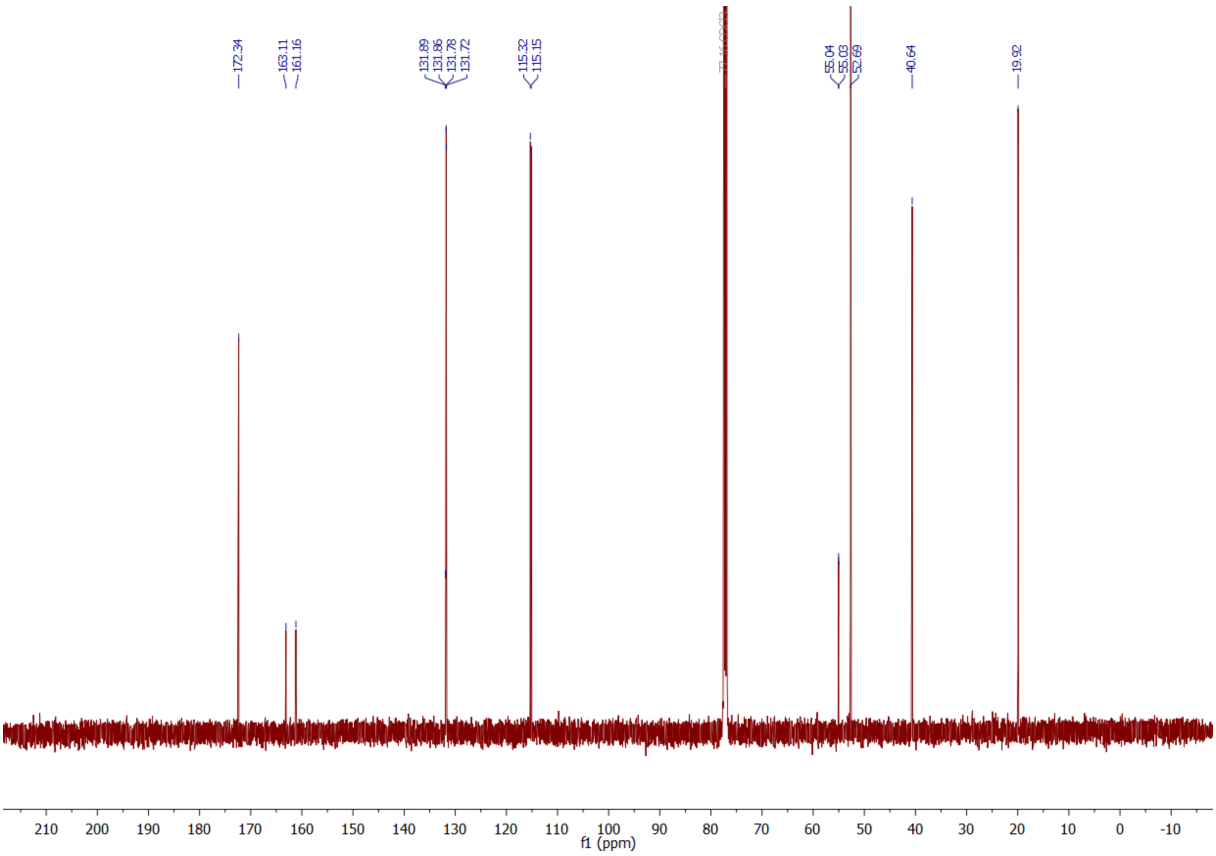

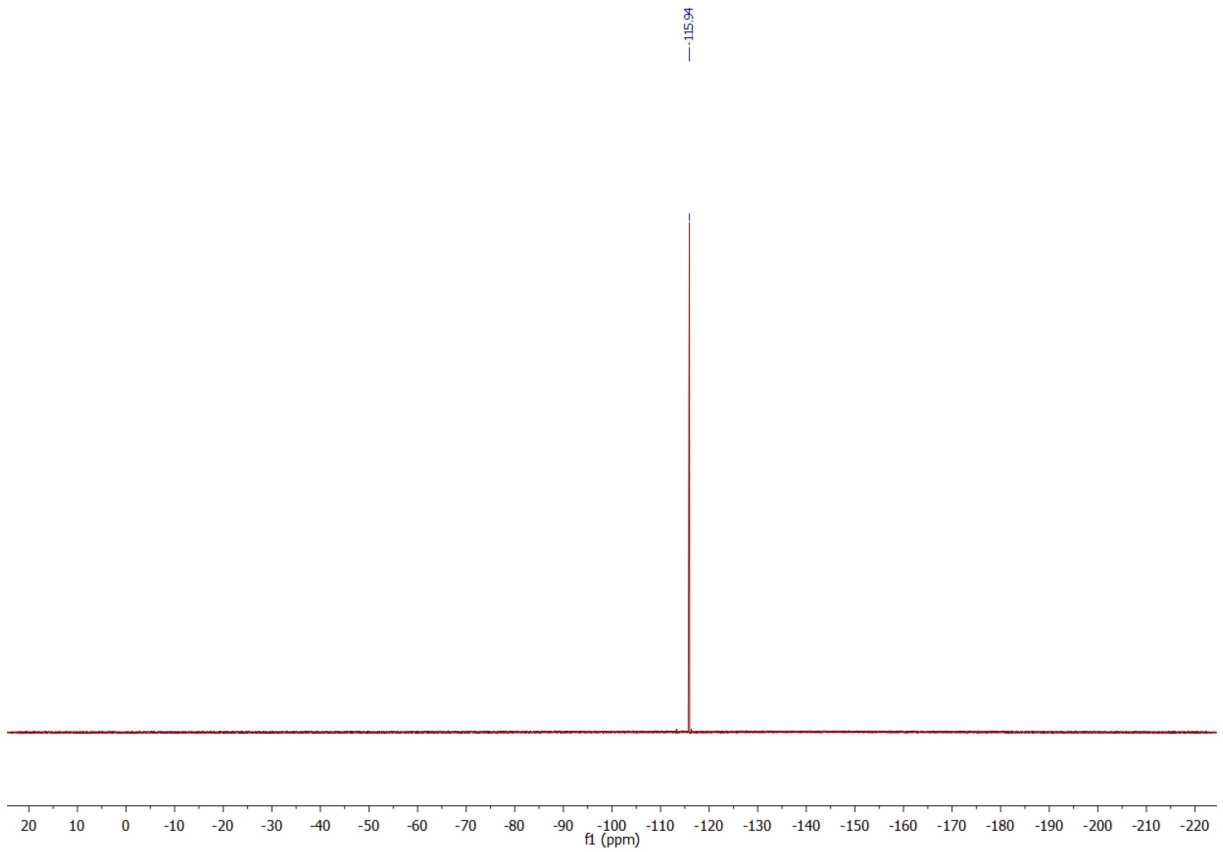
-115.94
20 10 0 -10 -20 -30 -40 -50 -60 -70 -80 -90 -100 -110 -120 -130 -140 -150 -160 -170 -180 -190 -200 -210 -220
f1 (ppm)

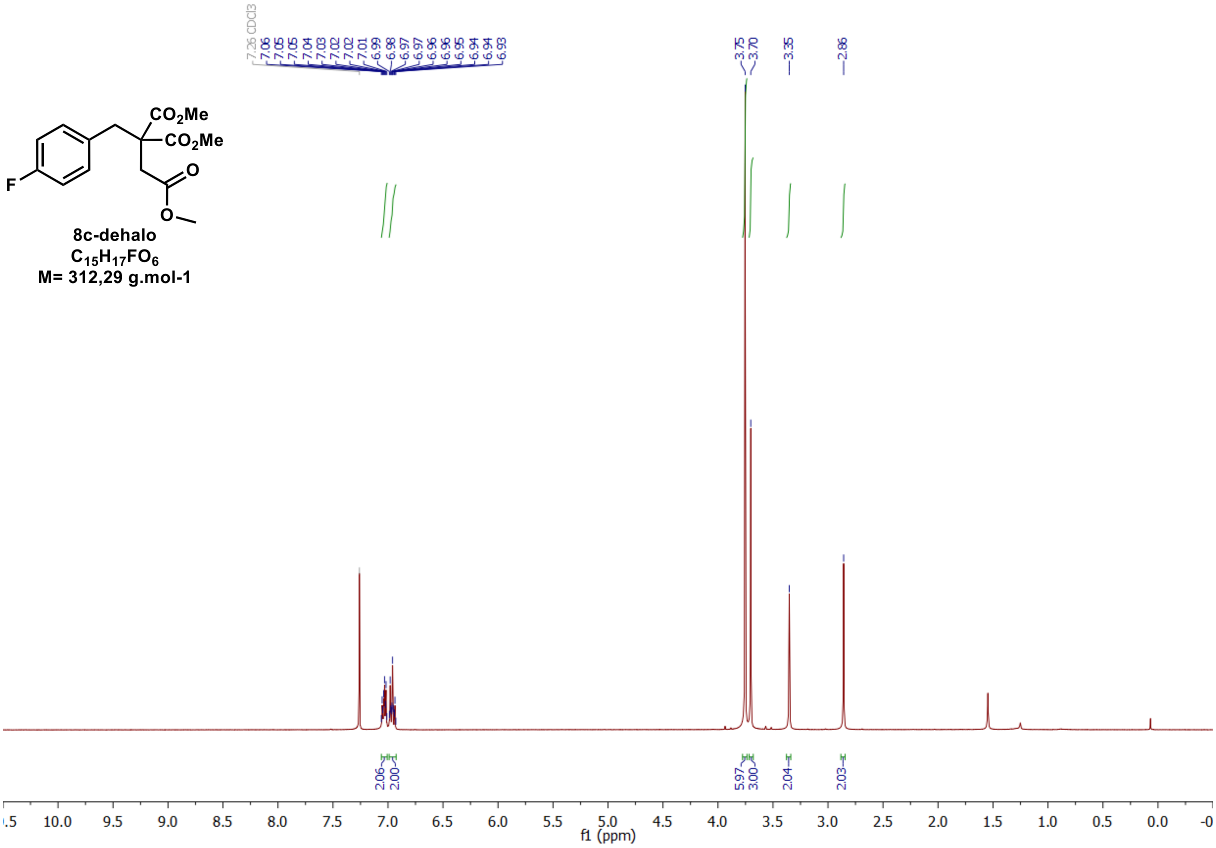

CO2Me
CO2Me
O
F
O
8c-dehalo
C15H17FO6
M= 312,29 g.mol-1
7.26 CDCl3
7.06 7.05 7.05 7.04 7.03 7.02 7.02 7.01 6.99 6.98 6.97 6.97 6.96 6.96 6.95 6.94 6.94 6.93
3.75 3.70
3.35
2.86
2.06 2.00
5.97 3.00
2.04
2.03
10.0 9.5 9.0 8.5 8.0 7.5 7.0 6.5 6.0 5.5 5.0 4.5 4.0 3.5 3.0 2.5 2.0 1.5 1.0 0.5 0.0
f1 (ppm)

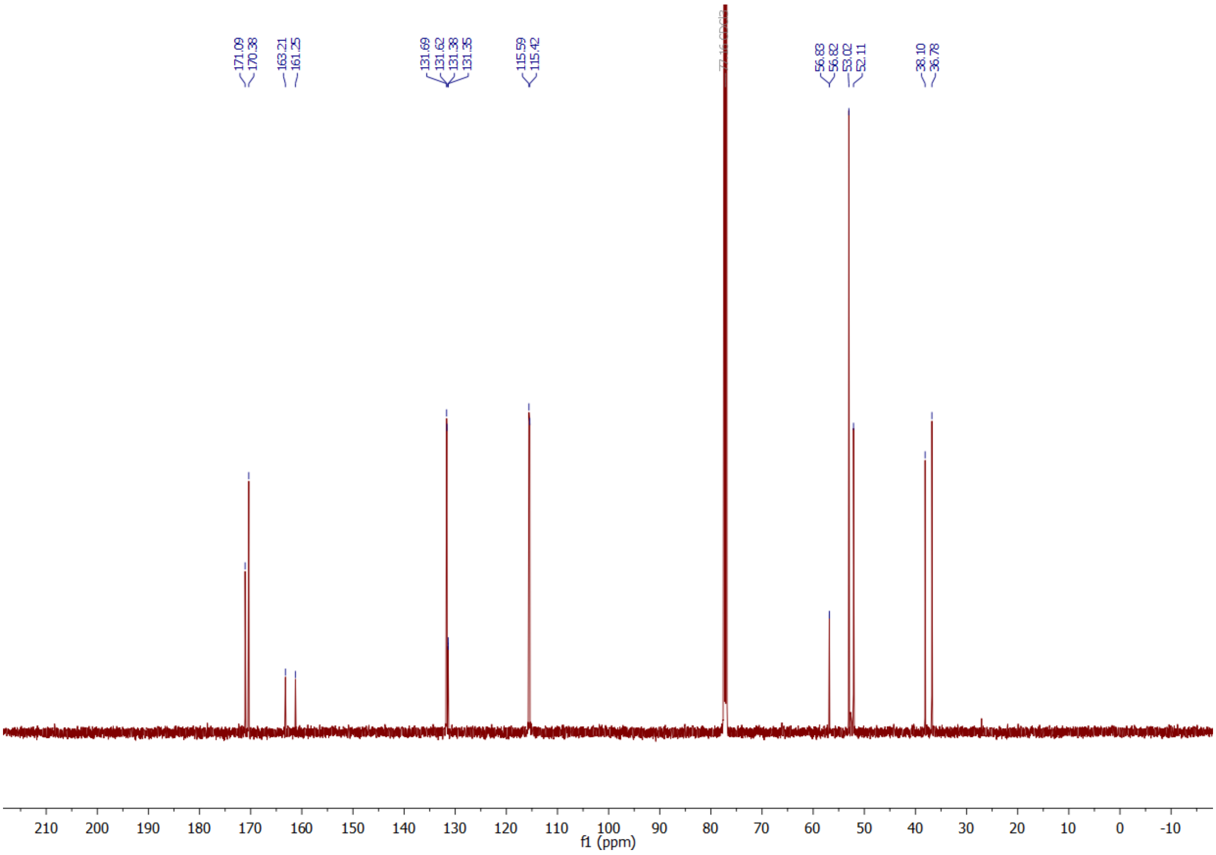

171.09 170.38
163.21 161.25
131.69 131.62 131.38 131.35
115.59 115.42
56.83 56.82 53.02 52.11
38.10 36.78
210 200 190 180 170 160 150 140 130 120 110 100 90 80 70 60 50 40 30 20 10 0 -10
f1 (ppm)

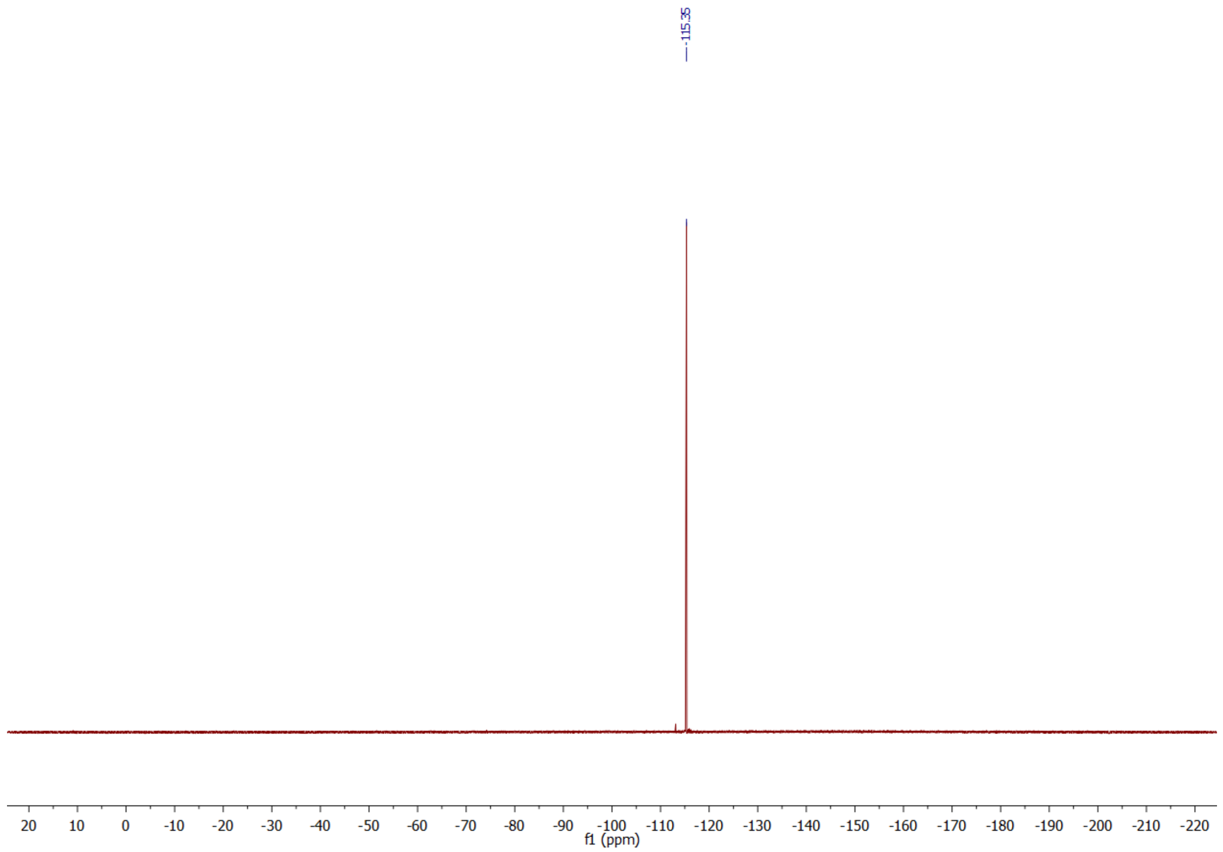
-115.35
20
10
0
-10
-20
-30
-40
-50
-60
-70
-80
-90
-100
-110
-120
-130
-140
-150
-160
-170
-180
-190
-200
-210
-220
f1 (ppm)

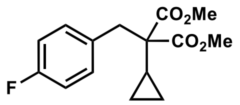

8d-dehalo
$C_{15}H_{17}FO_4$
M= 280,30 g.mol[-1]

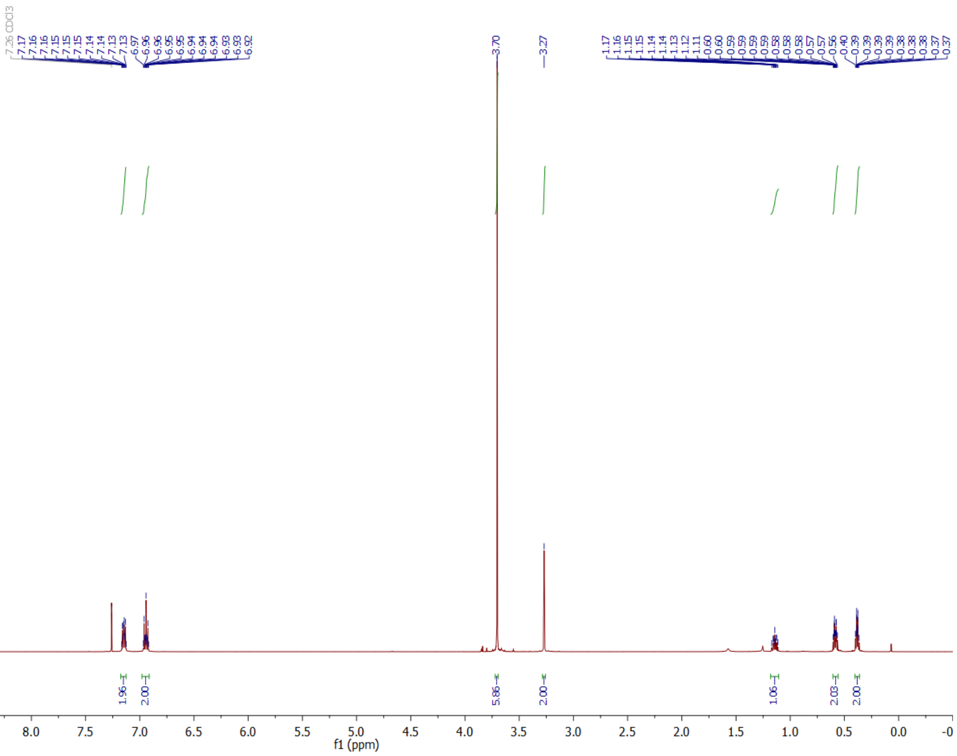

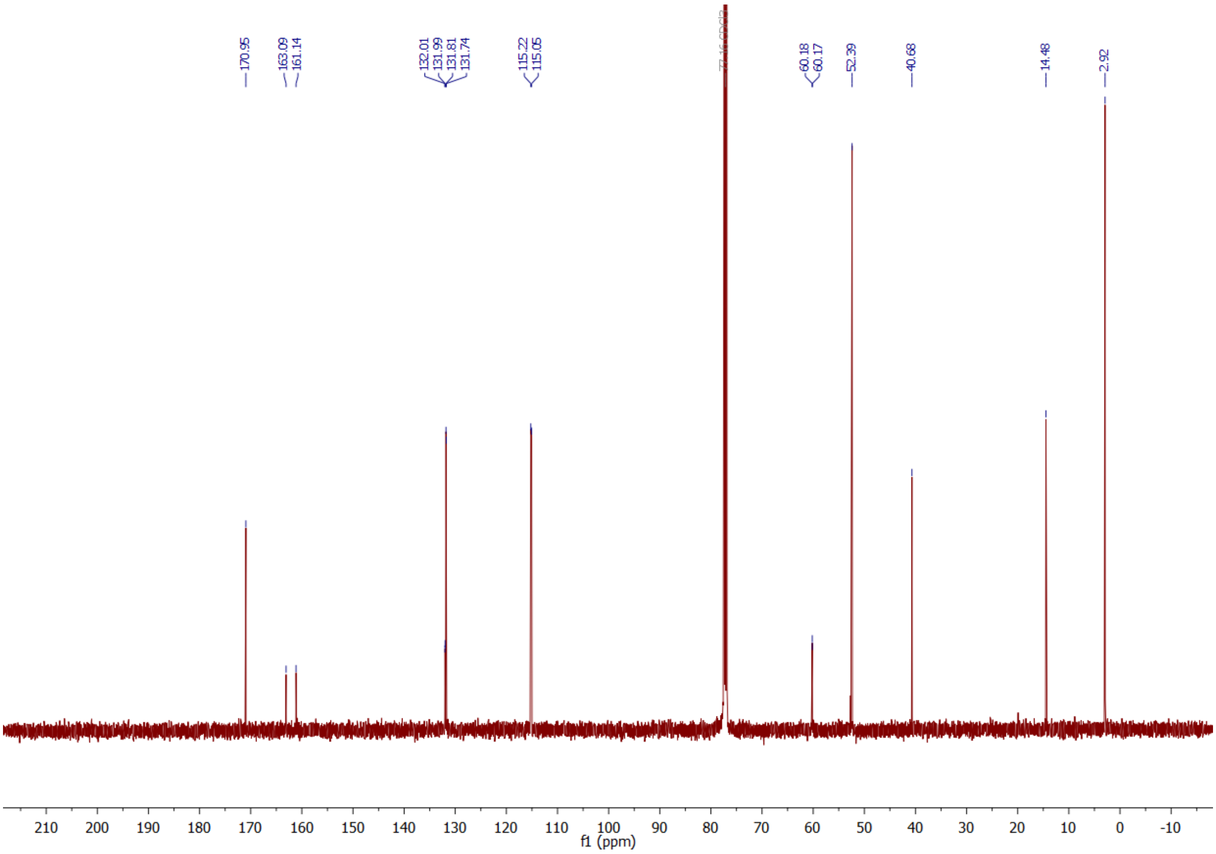

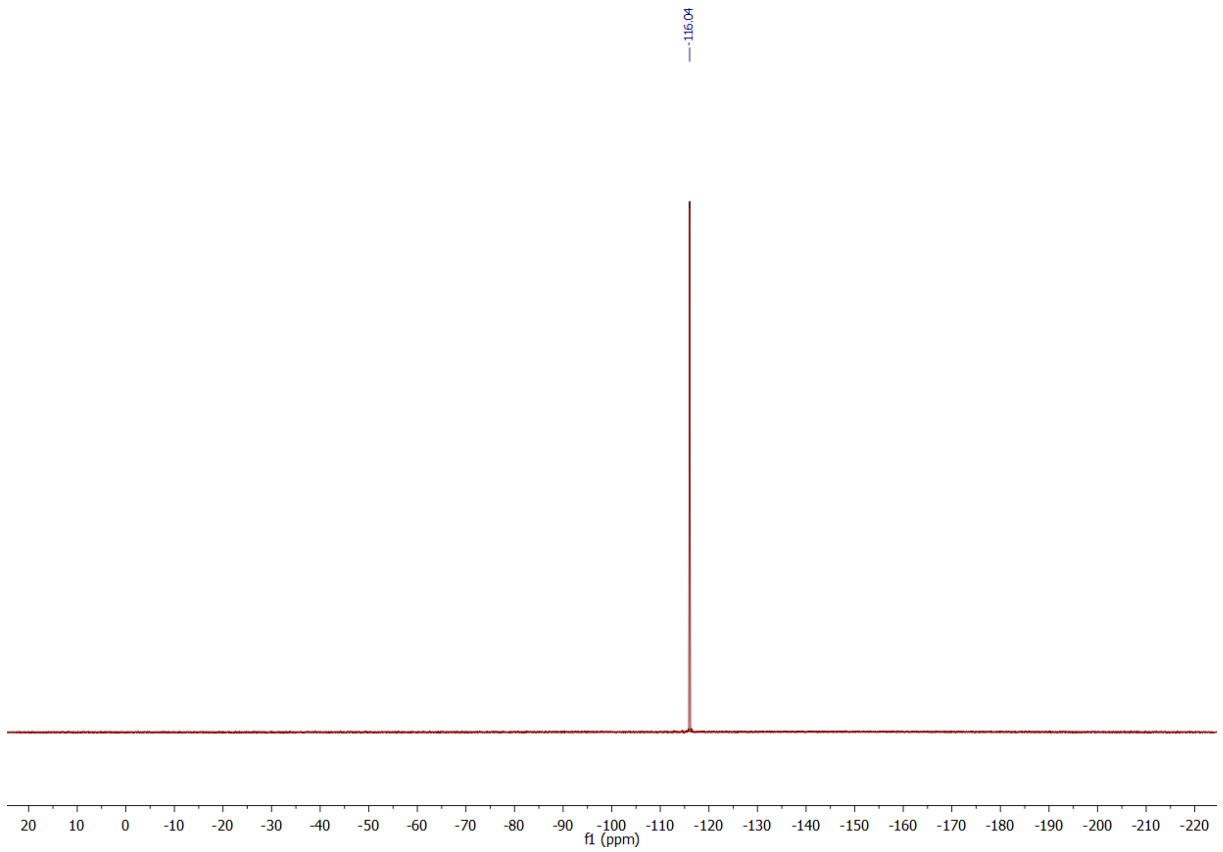
-116.04
20 10 0 -10 -20 -30 -40 -50 -60 -70 -80 -90 -100 -110 -120 -130 -140 -150 -160 -170 -180 -190 -200 -210 -220
f1 (ppm)

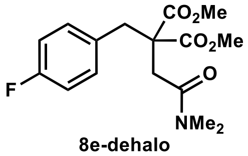

8e-dehalo
$C_{16}H_{20}FNO_5$
M= 325,34 g.mol-1

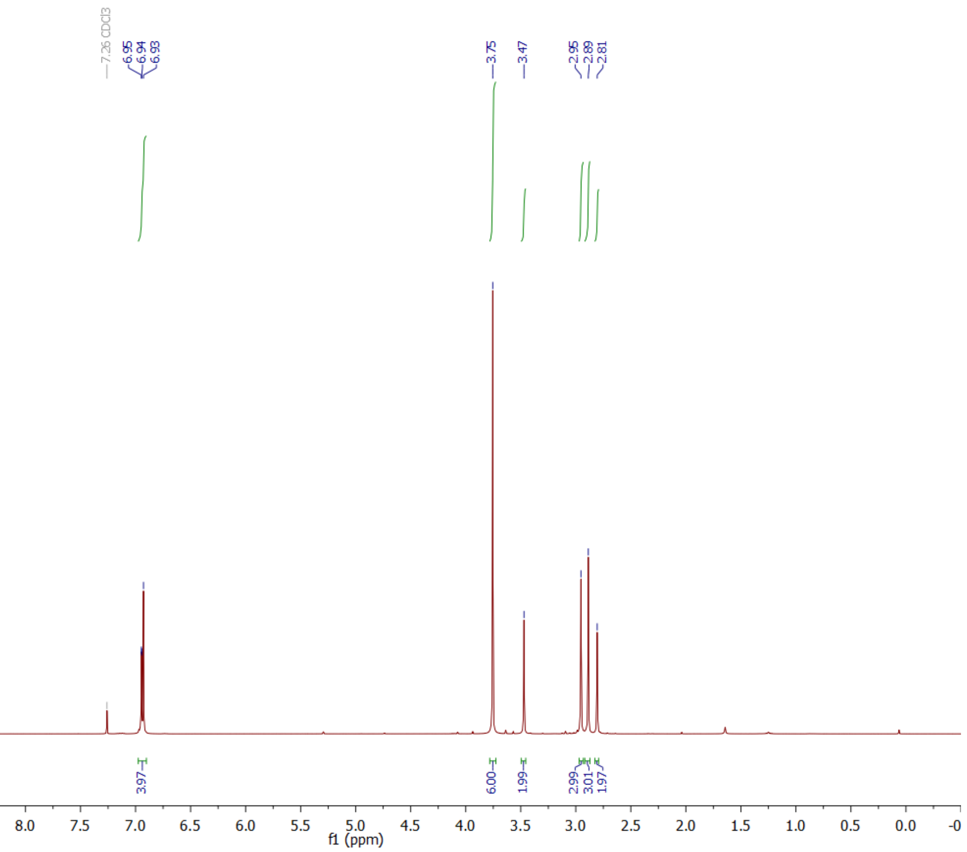

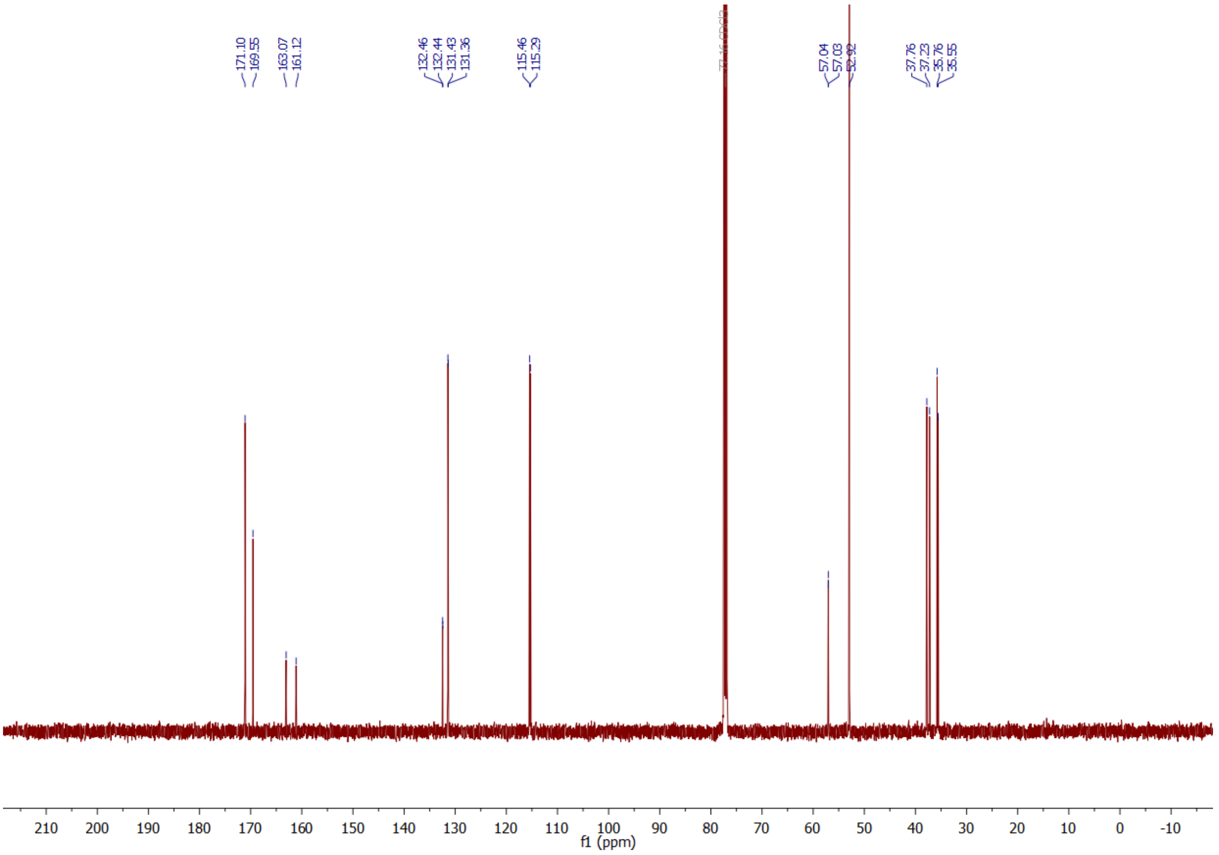

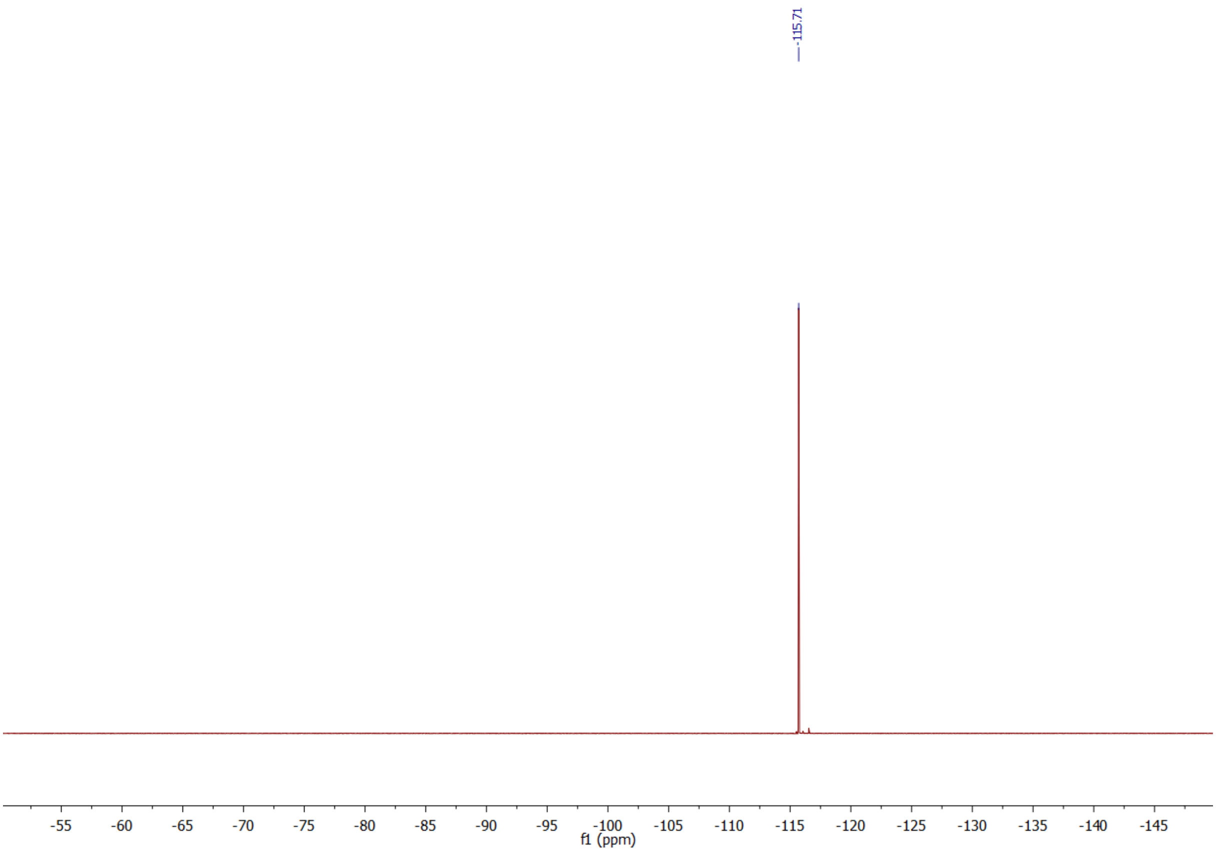
-115.71
-55 -60 -65 -70 -75 -80 -85 -90 -95 -100 -105 -110 -115 -120 -125 -130 -135 -140 -145
f1 (ppm)

8f-dehalo

$C_{19}H_{19}FO_4$

M= 330,36 g.mol[-1]

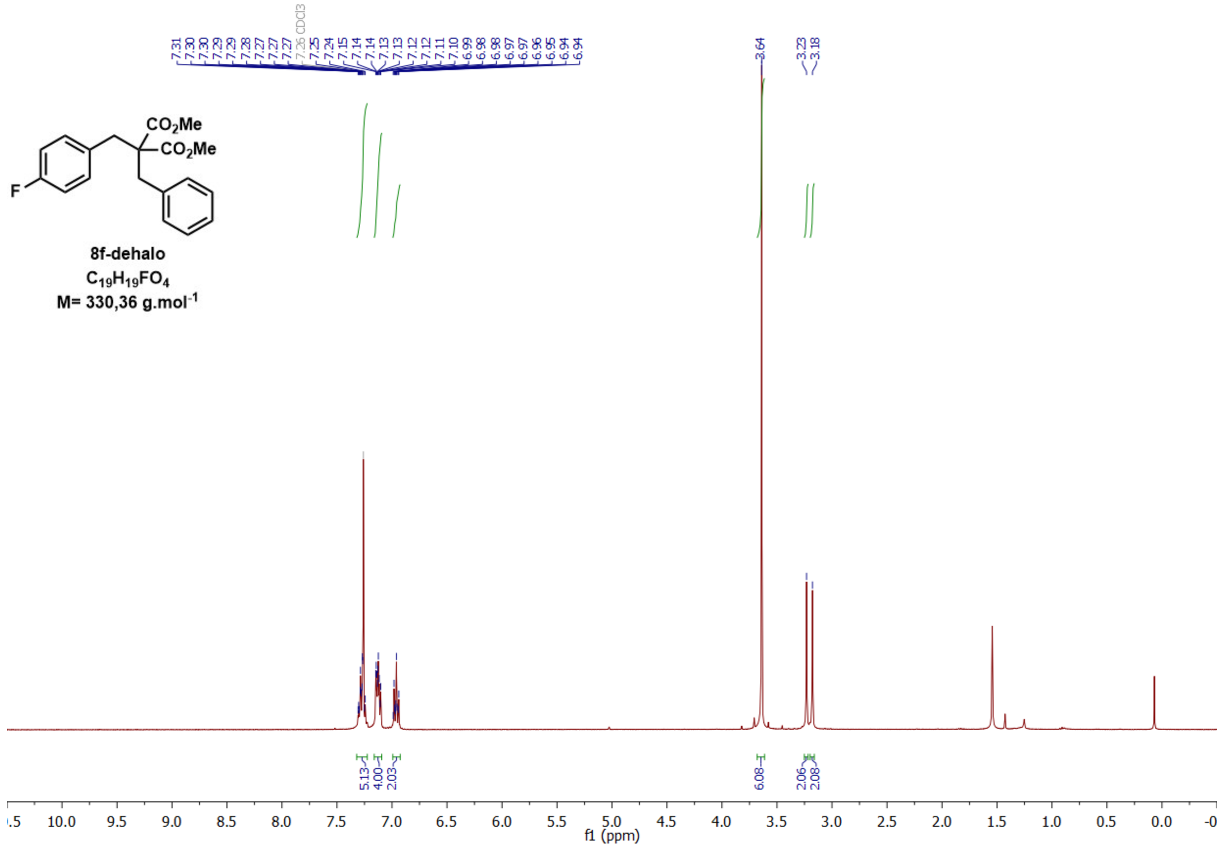

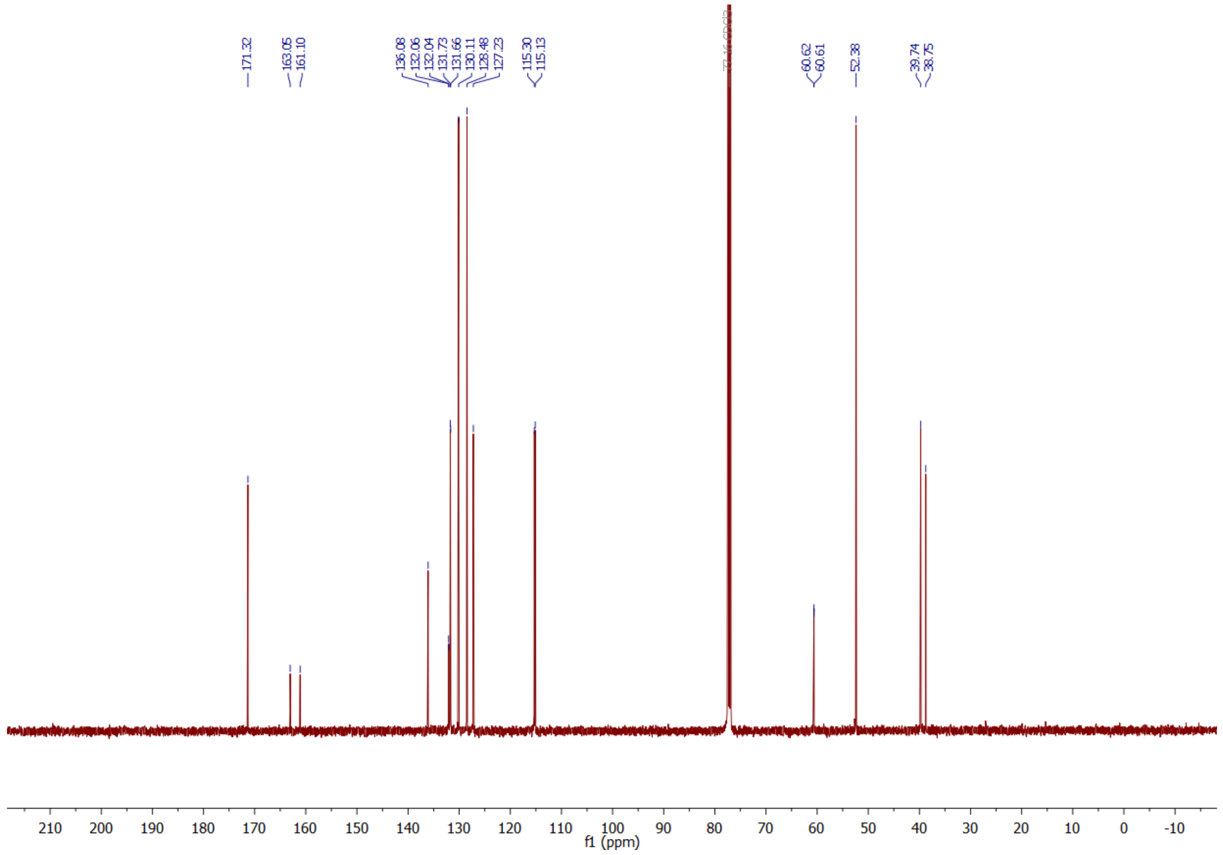

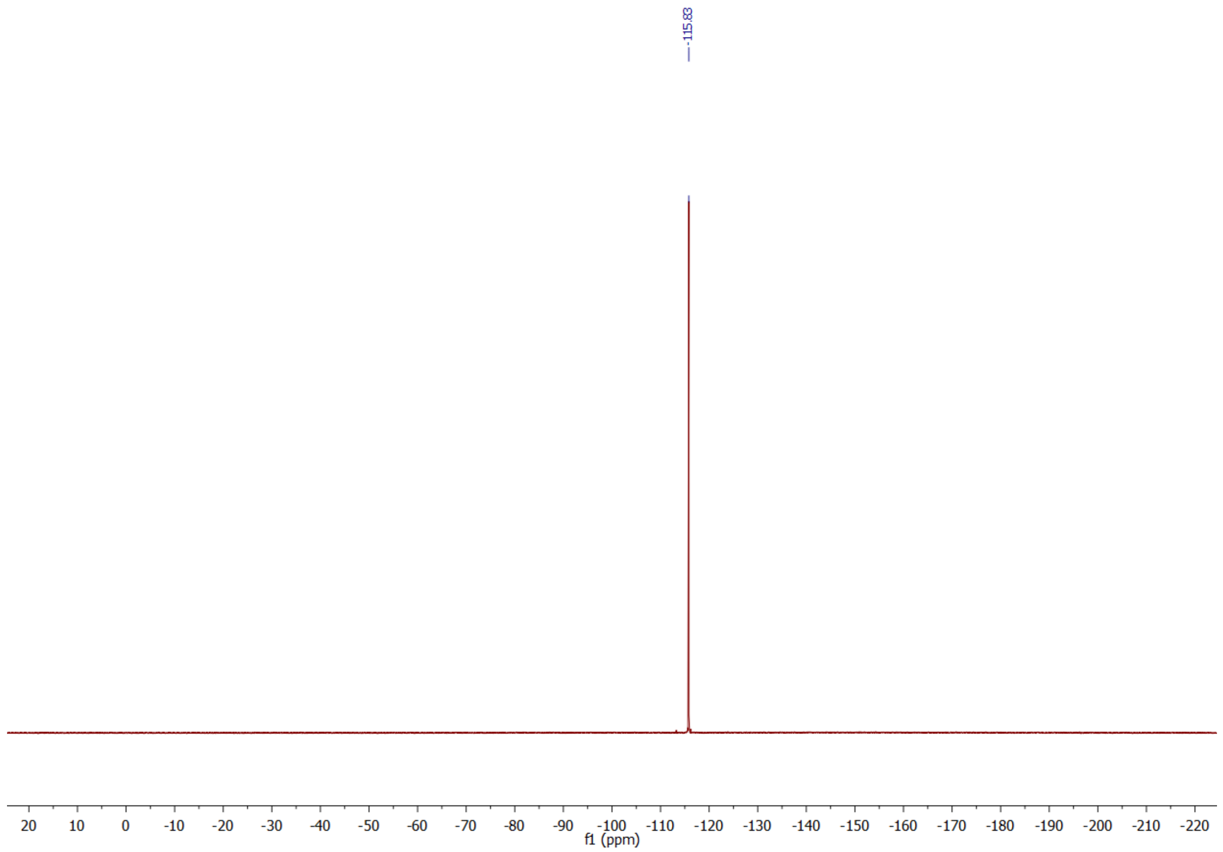
-115.83
20 10 0 -10 -20 -30 -40 -50 -60 -70 -80 -90 -100 -110 -120 -130 -140 -150 -160 -170 -180 -190 -200 -210 -220
f1 (ppm)

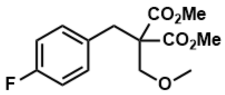

8g-dehalo
$C_{14}H_{17}FO_5$
M= 284,11 g.mol-1

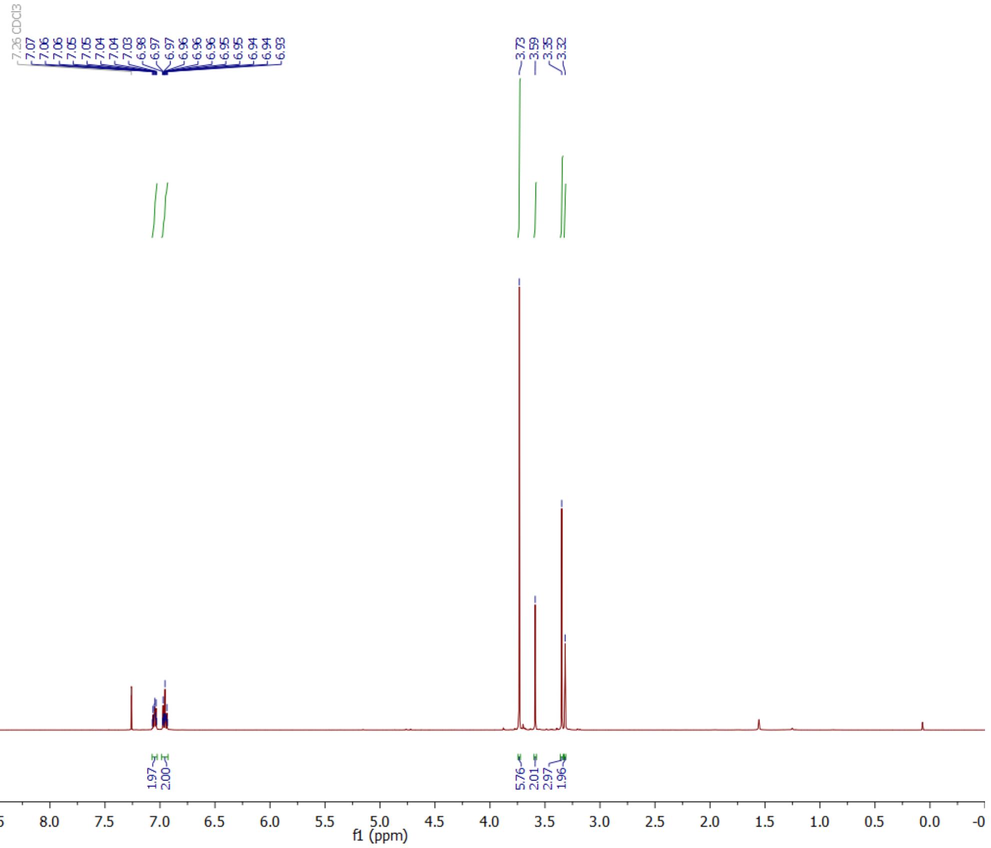

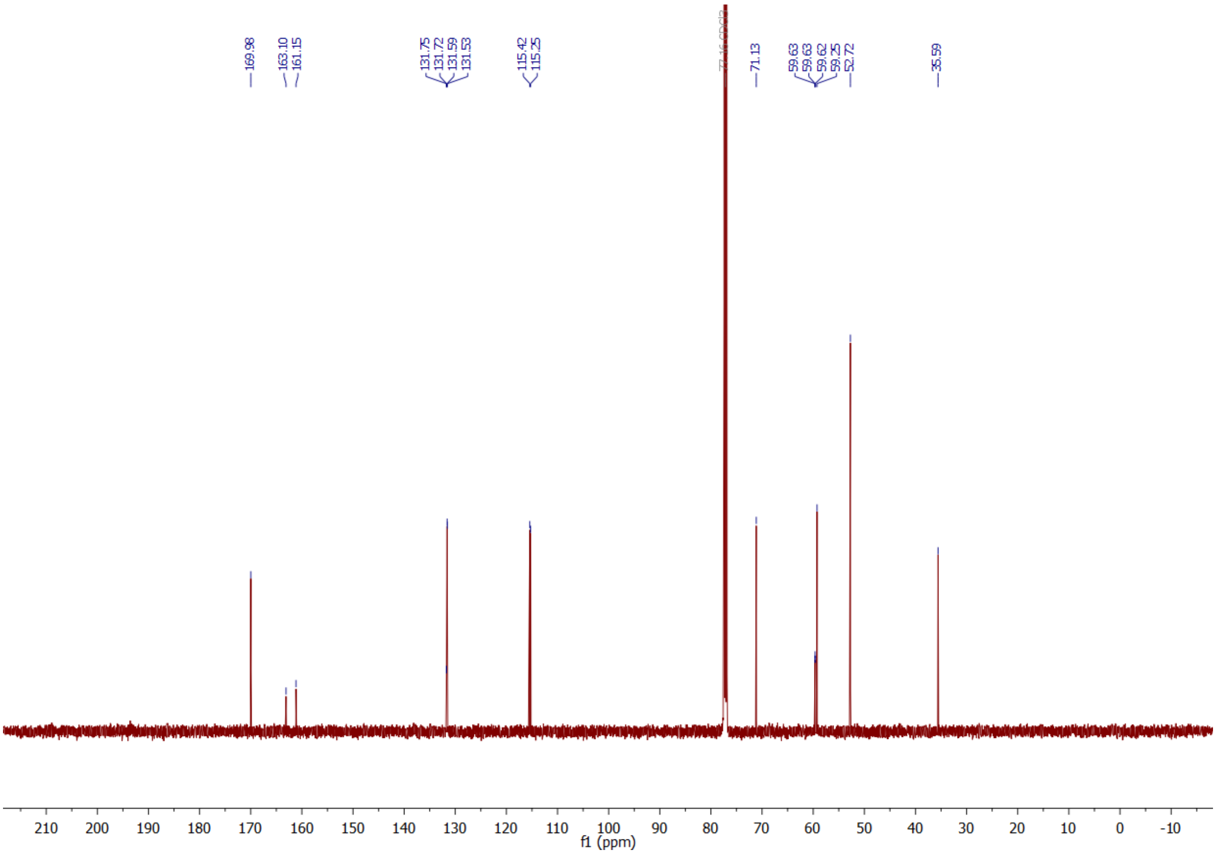

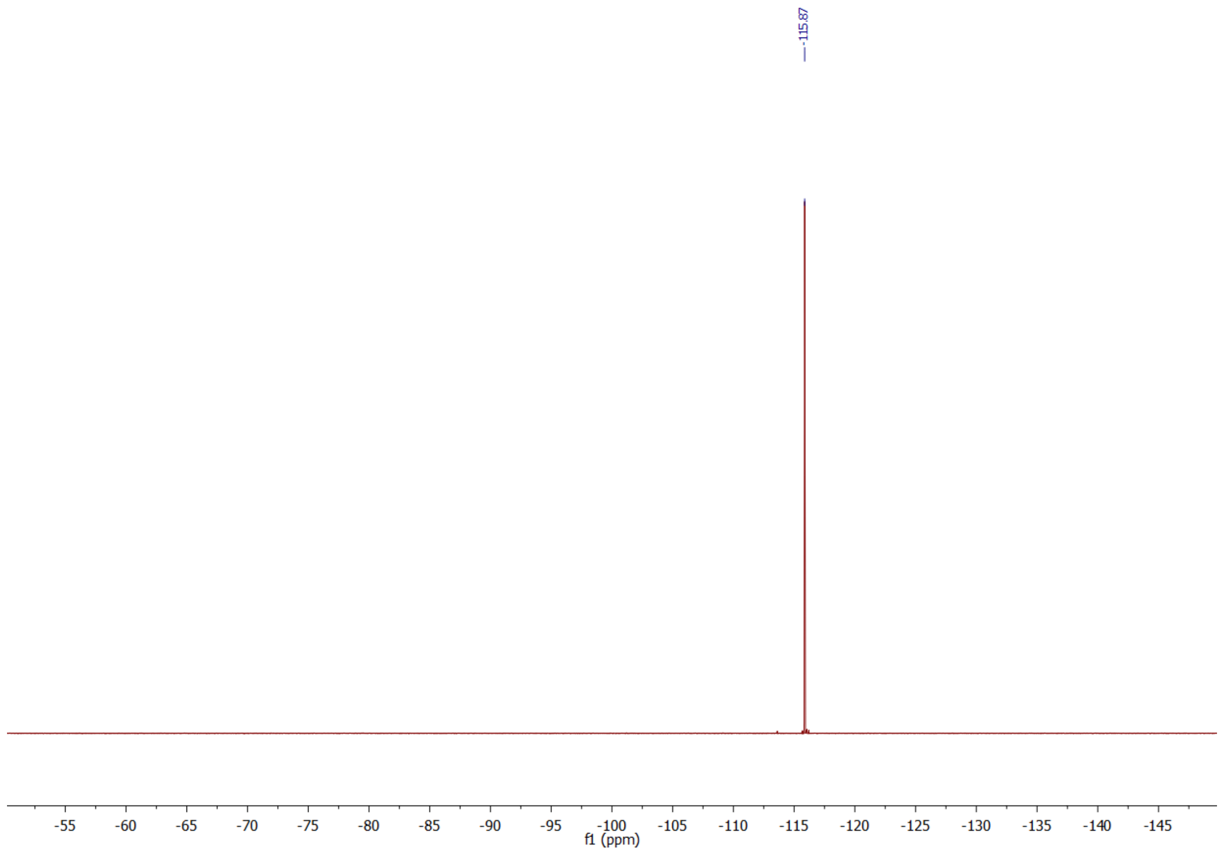

-115.87
-55 -60 -65 -70 -75 -80 -85 -90 -95 -100 -105 -110 -115 -120 -125 -130 -135 -140 -145
f1 (ppm)

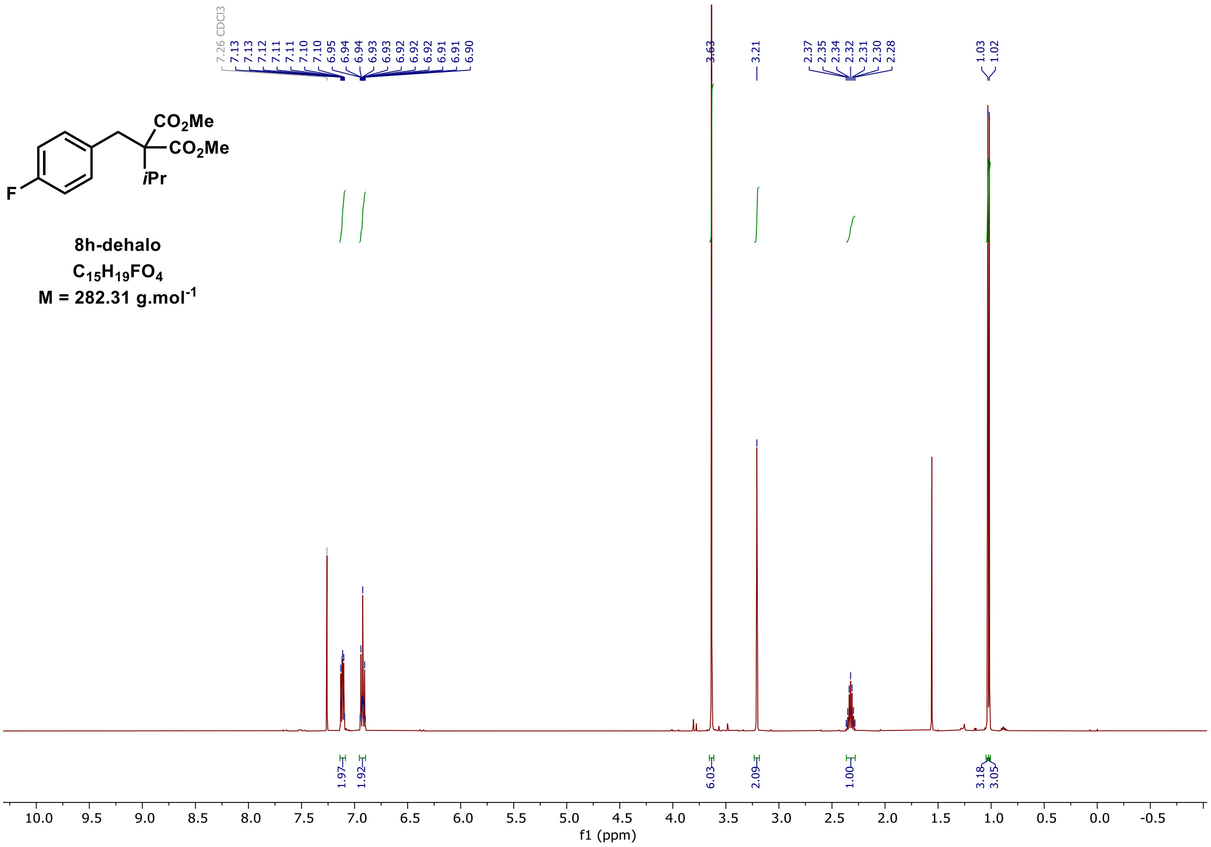
CO2Me
CO2Me
iPr
F
8h-dehalo
C15H19FO4
M = 282.31 g.mol-1
7.26 CDCl3
7.13
7.13
7.12
7.11
7.11
7.10
7.10
6.95
6.94
6.94
6.93
6.93
6.92
6.92
6.92
6.91
6.91
6.90
3.63
3.21
2.37
2.35
2.34
2.32
2.31
2.30
2.28
1.03
1.02
1.97
1.92
6.03
2.09
1.00
3.18
3.05
10.0
9.5
9.0
8.5
8.0
7.5
7.0
6.5
6.0
5.5
5.0
4.5
4.0
3.5
3.0
2.5
2.0
1.5
1.0
0.5
0.0
-0.5
f1 (ppm)

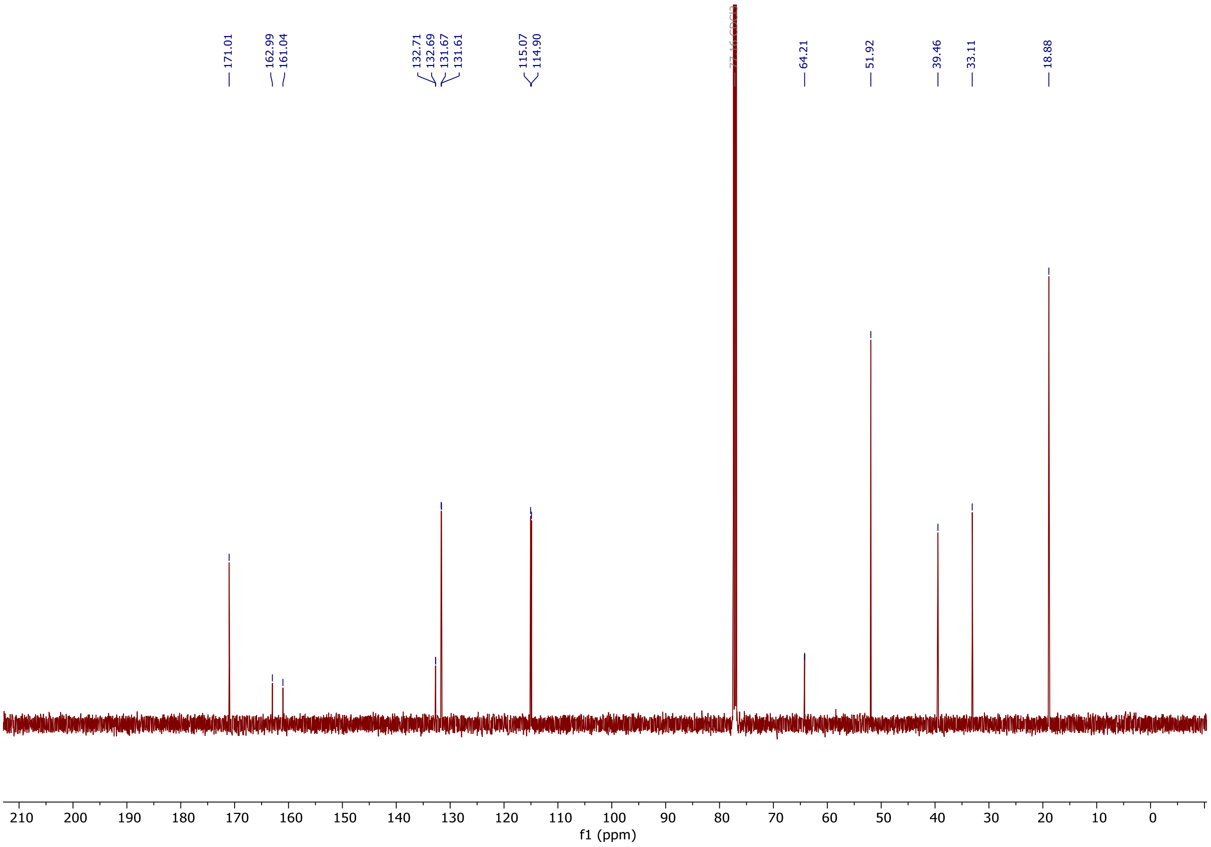
171.01
162.99
161.04
132.71
132.69
131.67
131.61
115.07
114.90
64.21
51.92
39.46
33.11
18.88
210
200
190
180
170
160
150
140
130
120
110
100
90
80
70
60
50
40
30
20
10
0
f1 (ppm)

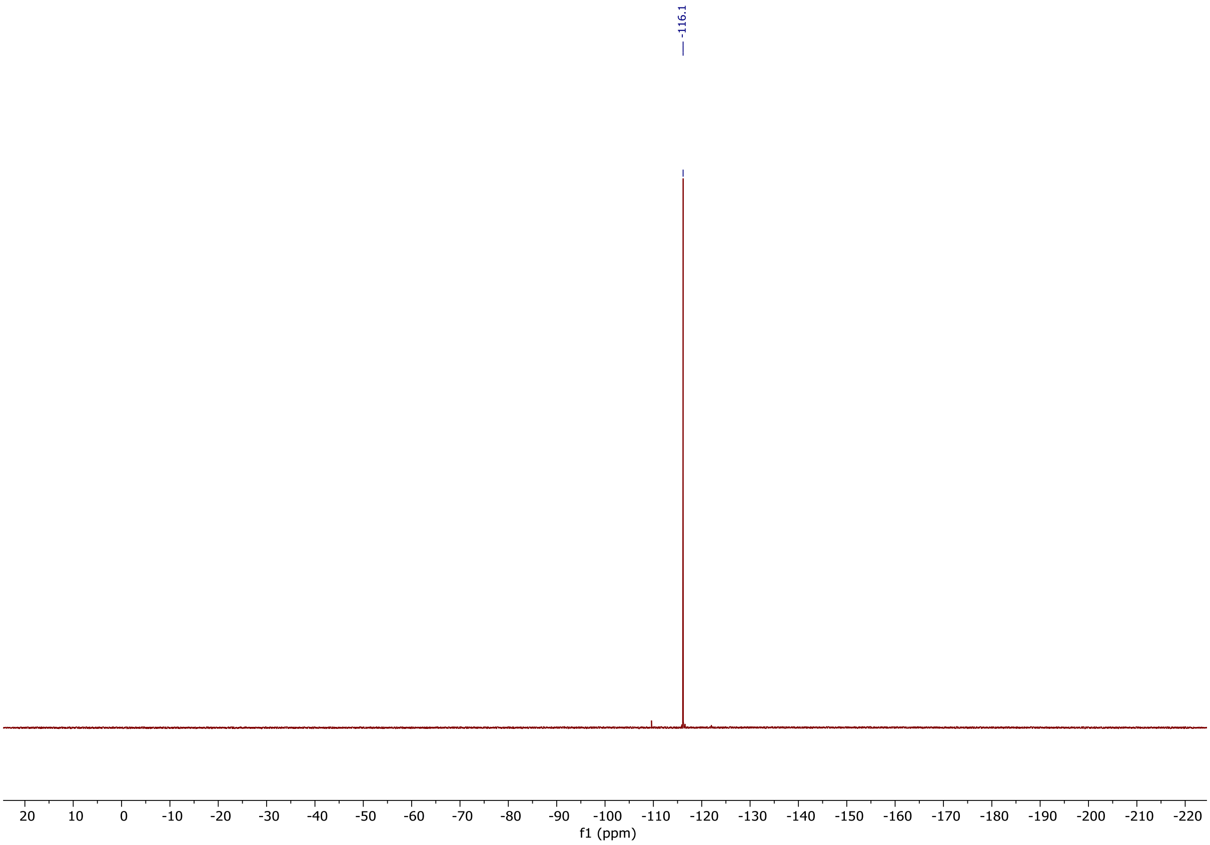
-116.1
20 10 0 -10 -20 -30 -40 -50 -60 -70 -80 -90 -100 -110 -120 -130 -140 -150 -160 -170 -180 -190 -200 -210 -220
f1 (ppm)

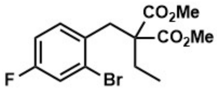

8a

$C_{14}H_{16}FBrO_4$

M= 347,18 g.mol[-1]

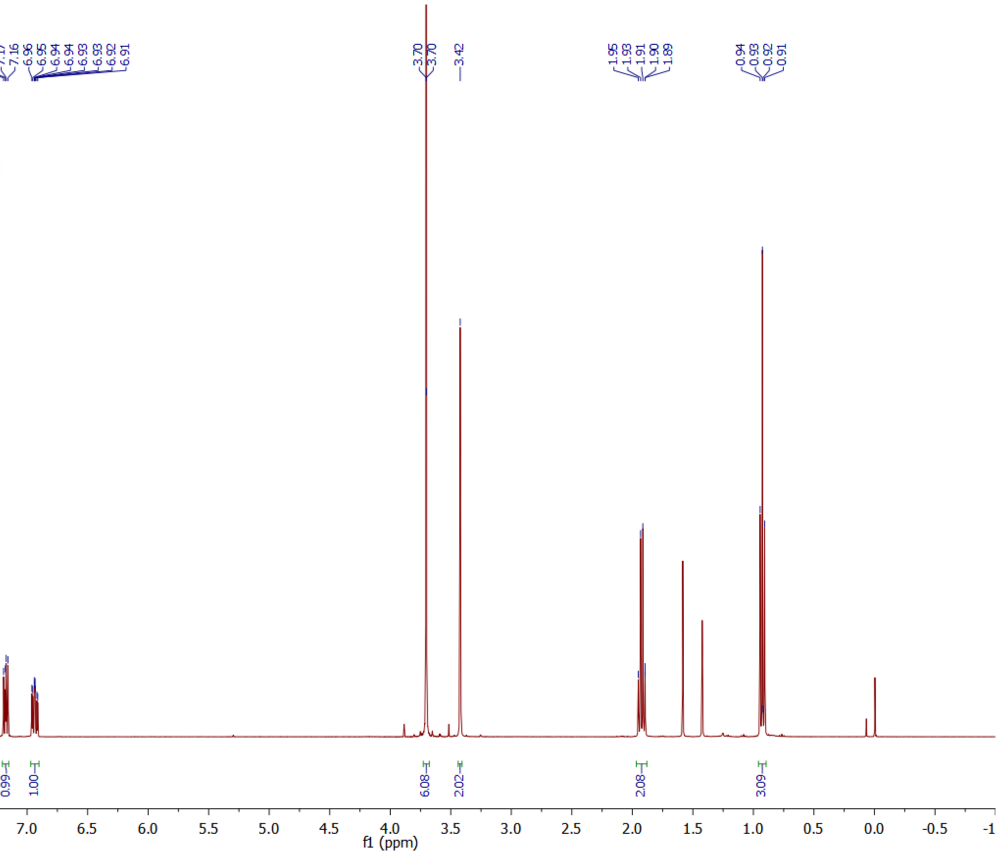

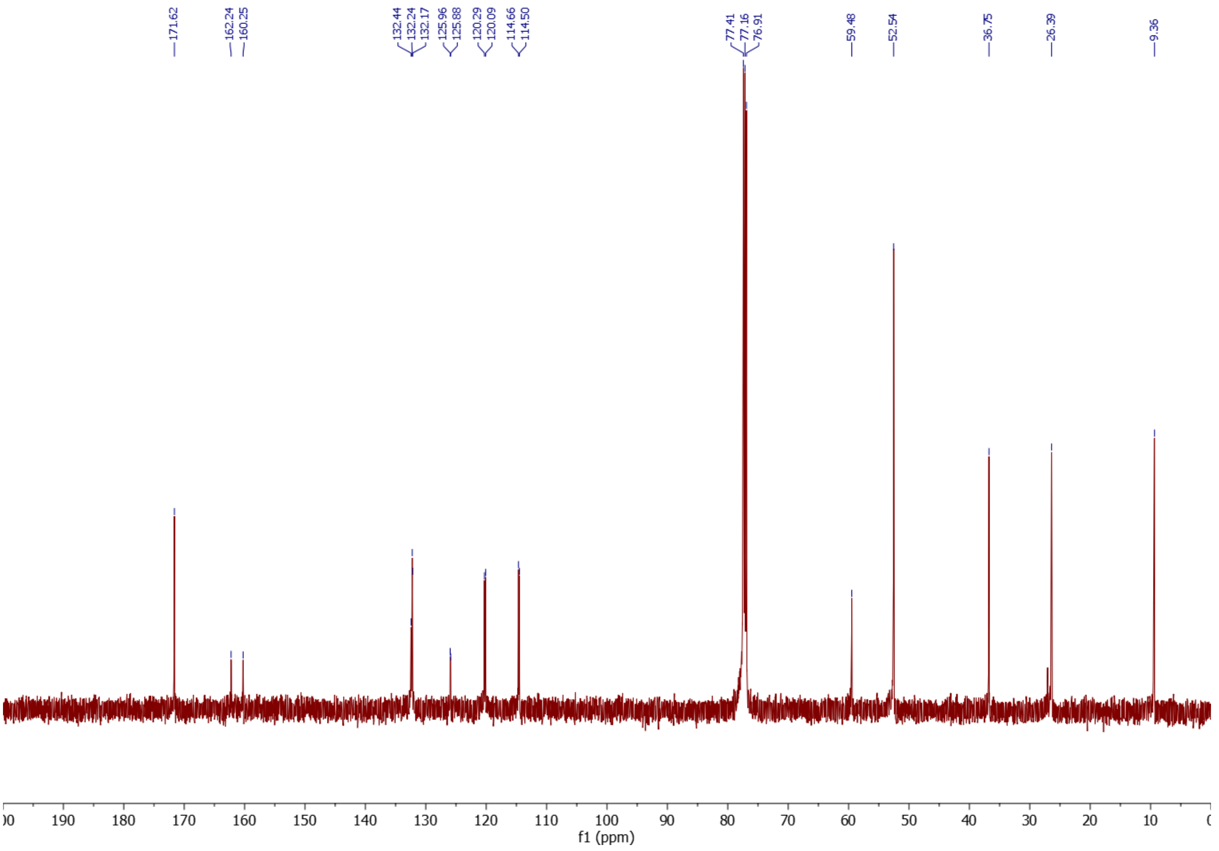

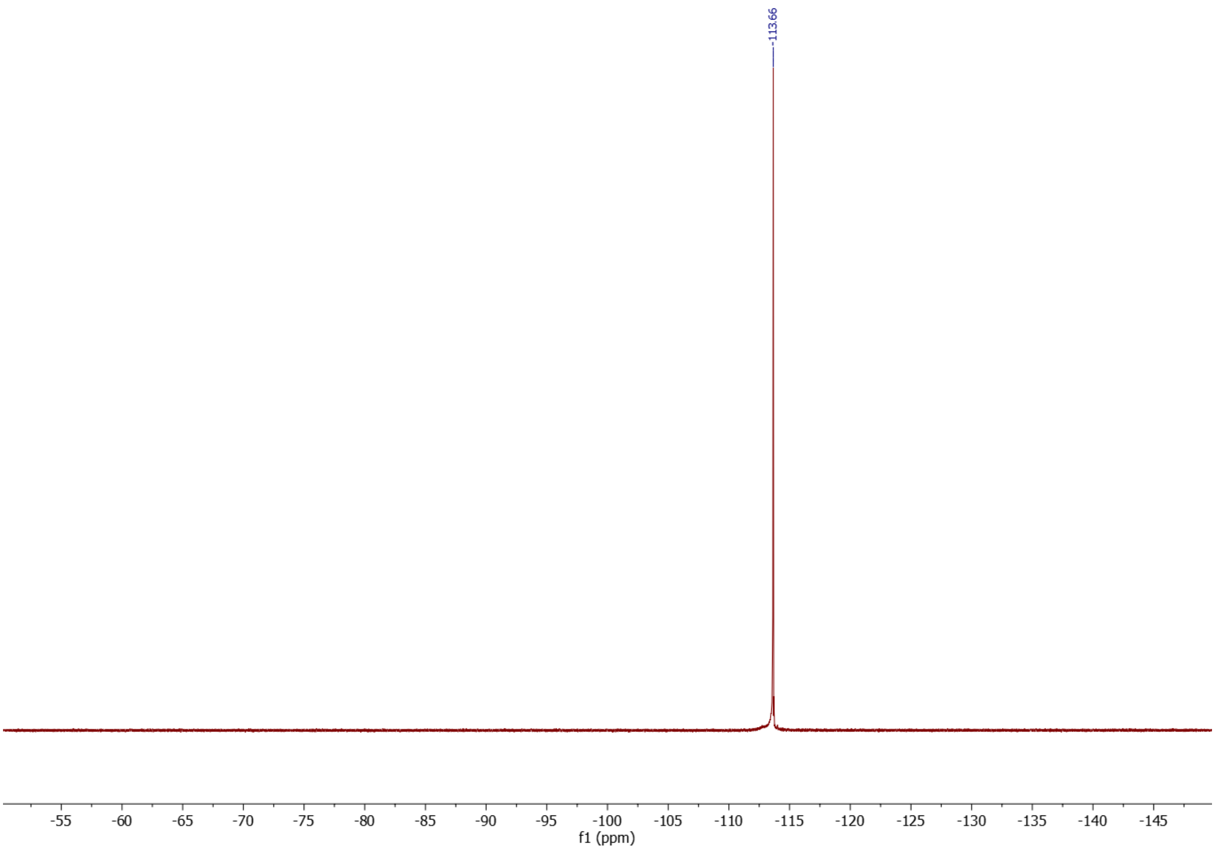
-113.66
-55
-60
-65
-70
-75
-80
-85
-90
-95
-100
-105
-110
-115
-120
-125
-130
-135
-140
-145
f1 (ppm)

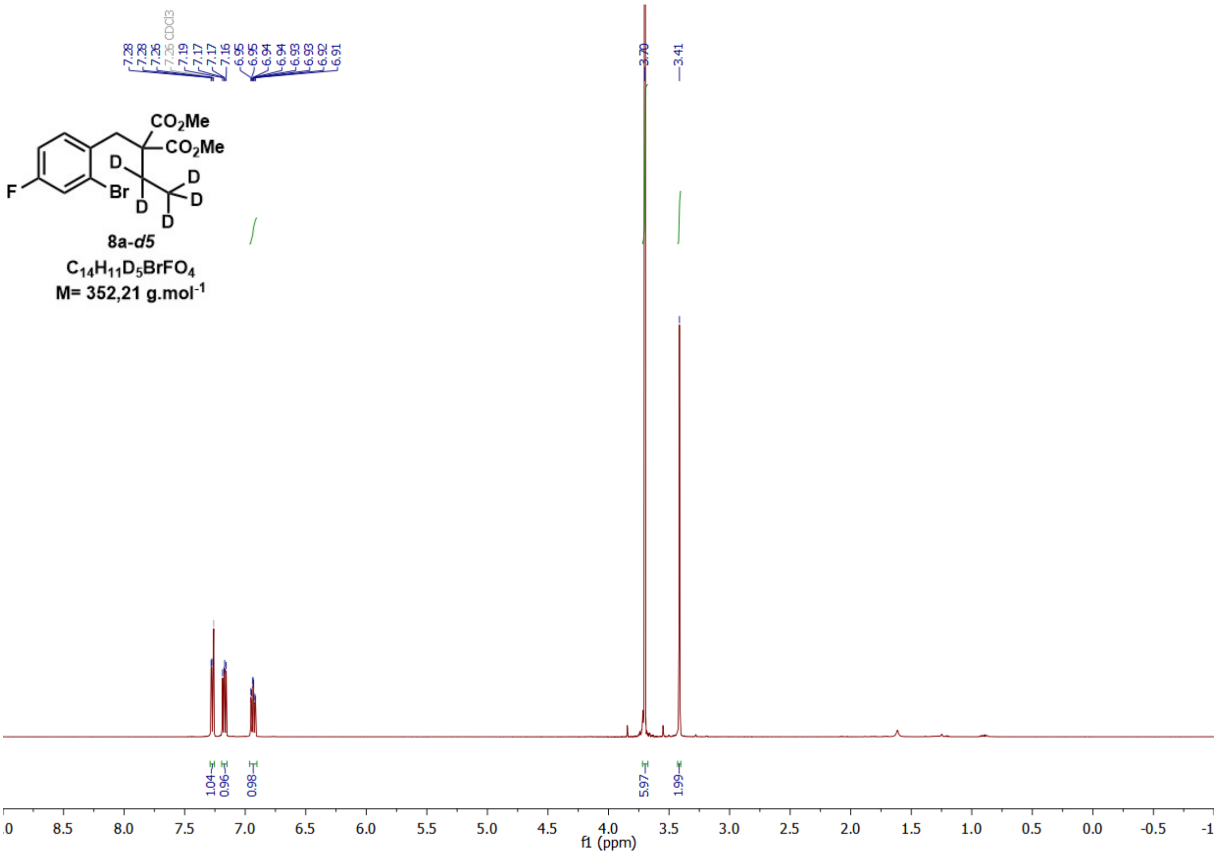
CO2Me
CO2Me
D
F
Br
D
D
D
D
8a-d5
C14H11D5BrFO4
M= 352,21 g.mol-1
7.28 7.28 7.26 7.26 CDCl3 7.19 7.17 7.17 7.16 6.95 6.95 6.94 6.94 6.93 6.93 6.92 6.91
3.70
3.41
1.04
0.96
0.98
5.97
1.99
f1 (ppm)

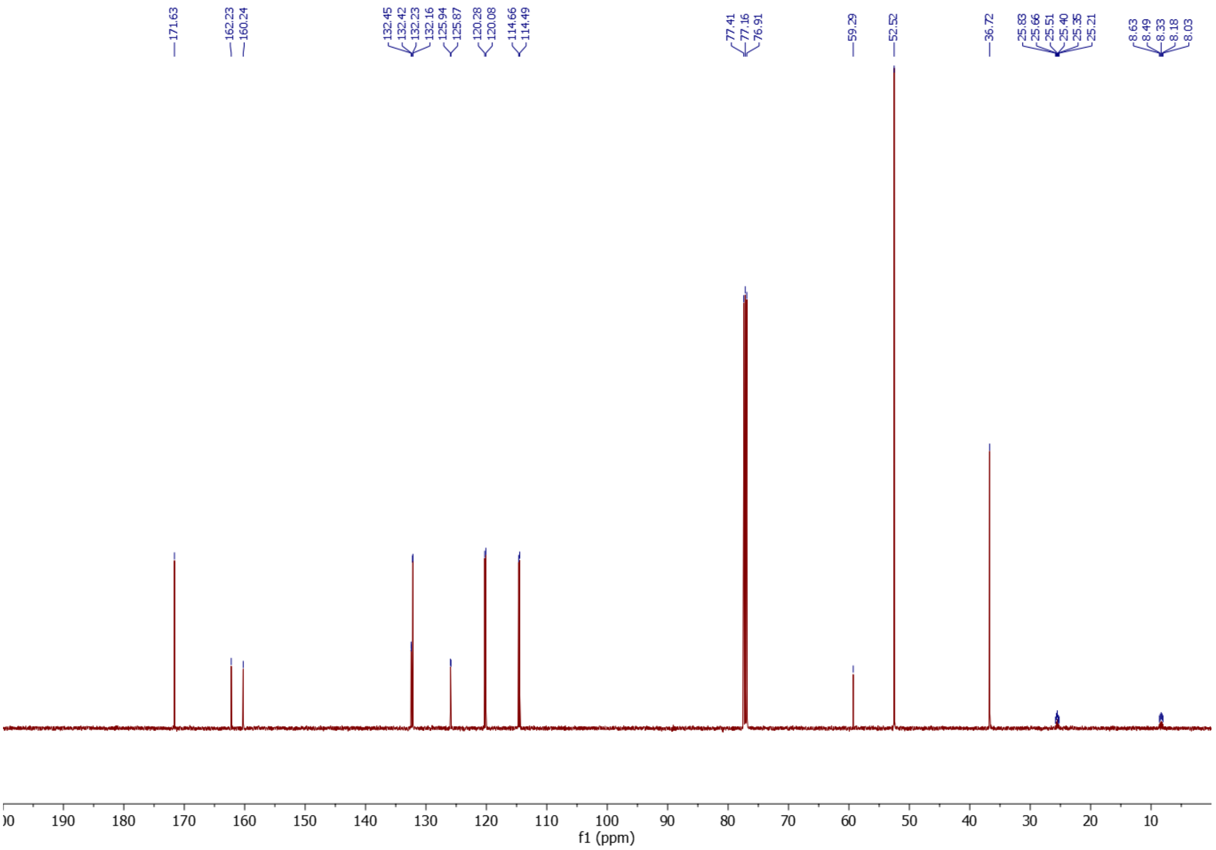
171.63
162.23 160.24
132.45 132.42 132.23 132.16 125.94 125.87 120.28 120.08 114.66 114.49
77.41 77.16 76.91
59.29
52.52
36.72
25.83 25.66 25.51 25.40 25.35 25.21
8.63 8.49 8.33 8.18 8.03
f1 (ppm)

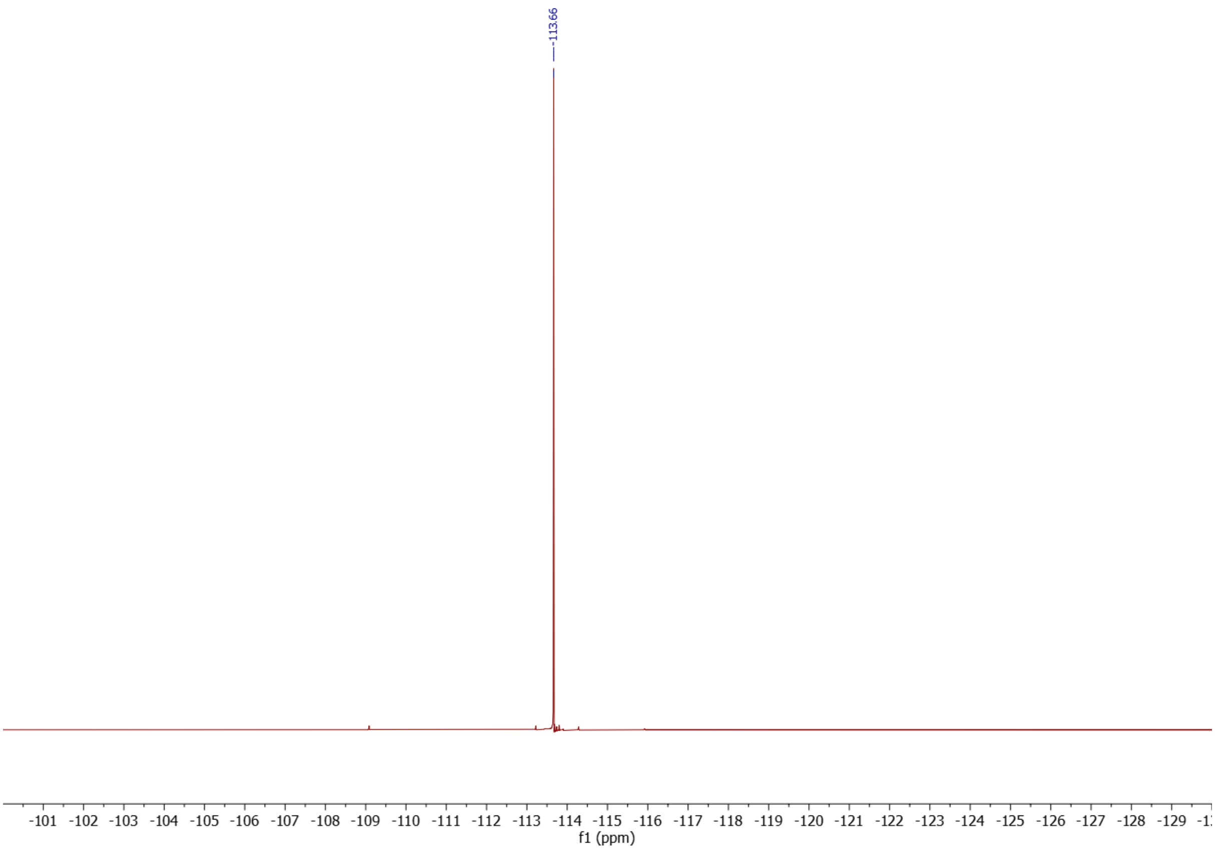
-113.66
-101 -102 -103 -104 -105 -106 -107 -108 -109 -110 -111 -112 -113 -114 -115 -116 -117 -118 -119 -120 -121 -122 -123 -124 -125 -126 -127 -128 -129
f1 (ppm)

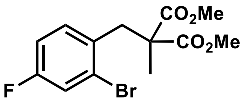

8b

$C_{13}H_{14}BrFO_4$

M= 333,15 g.mol^{-1}

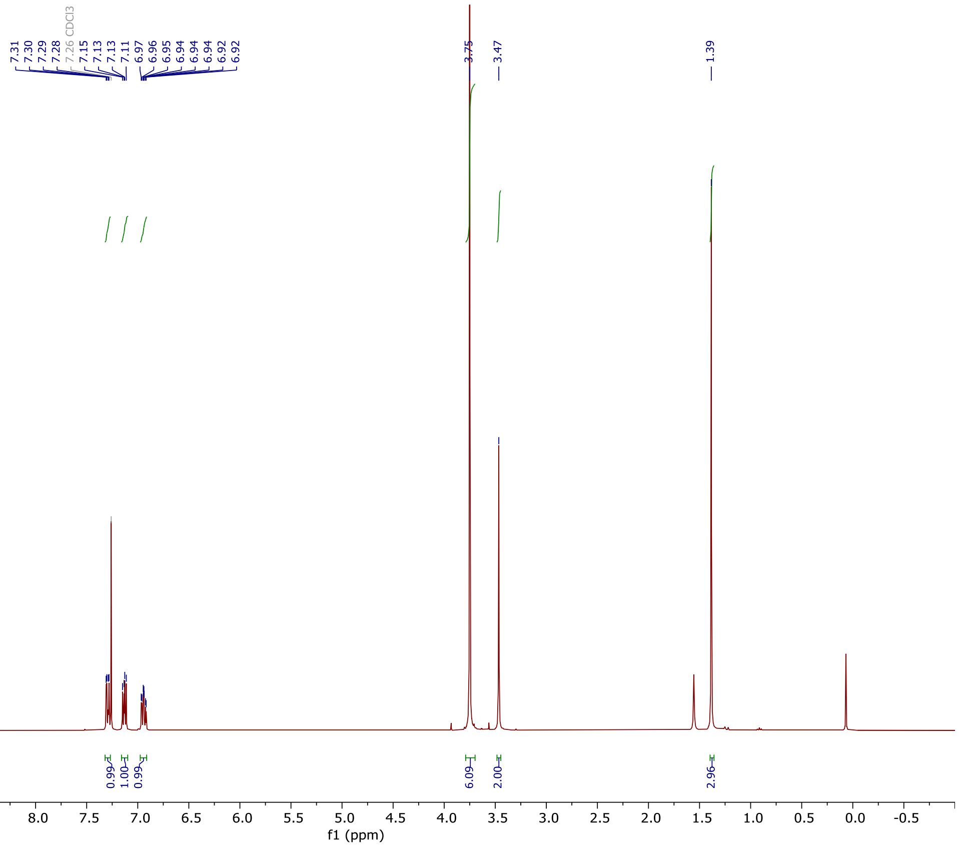

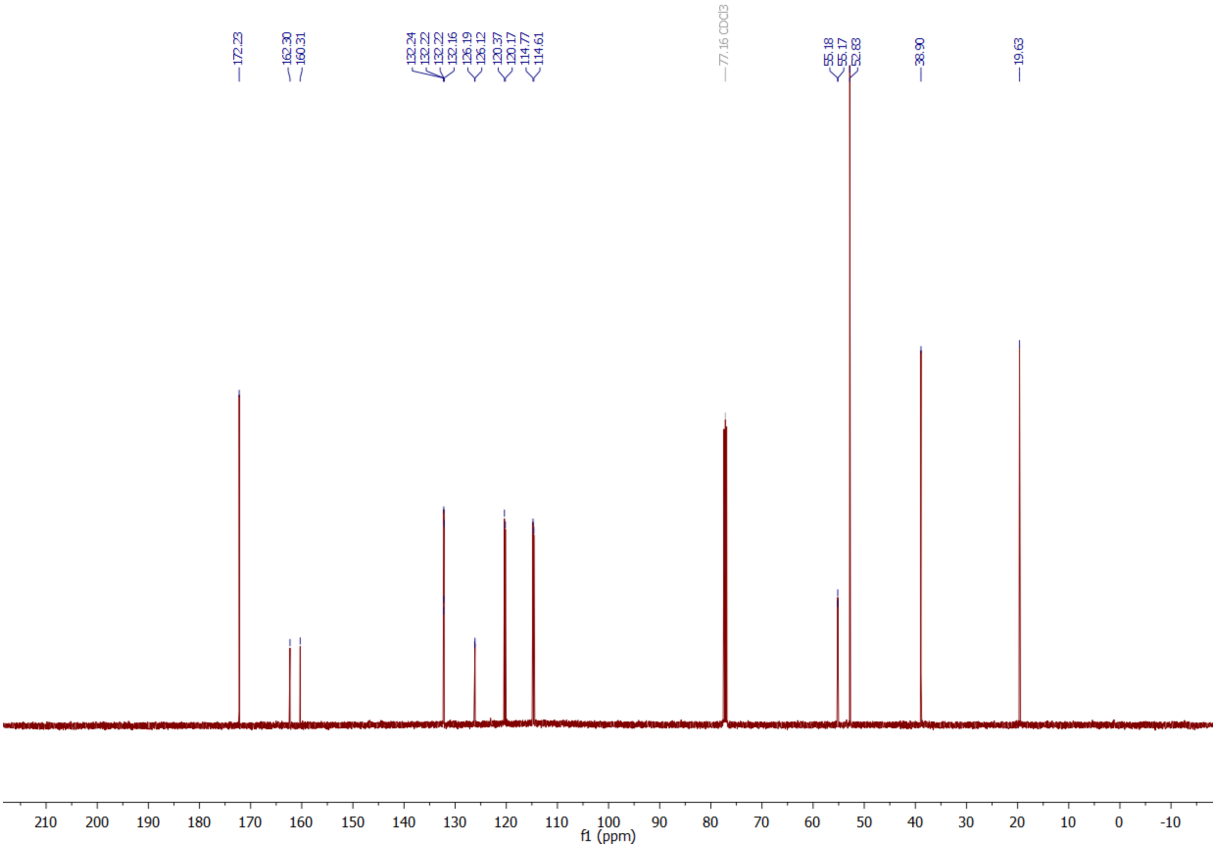

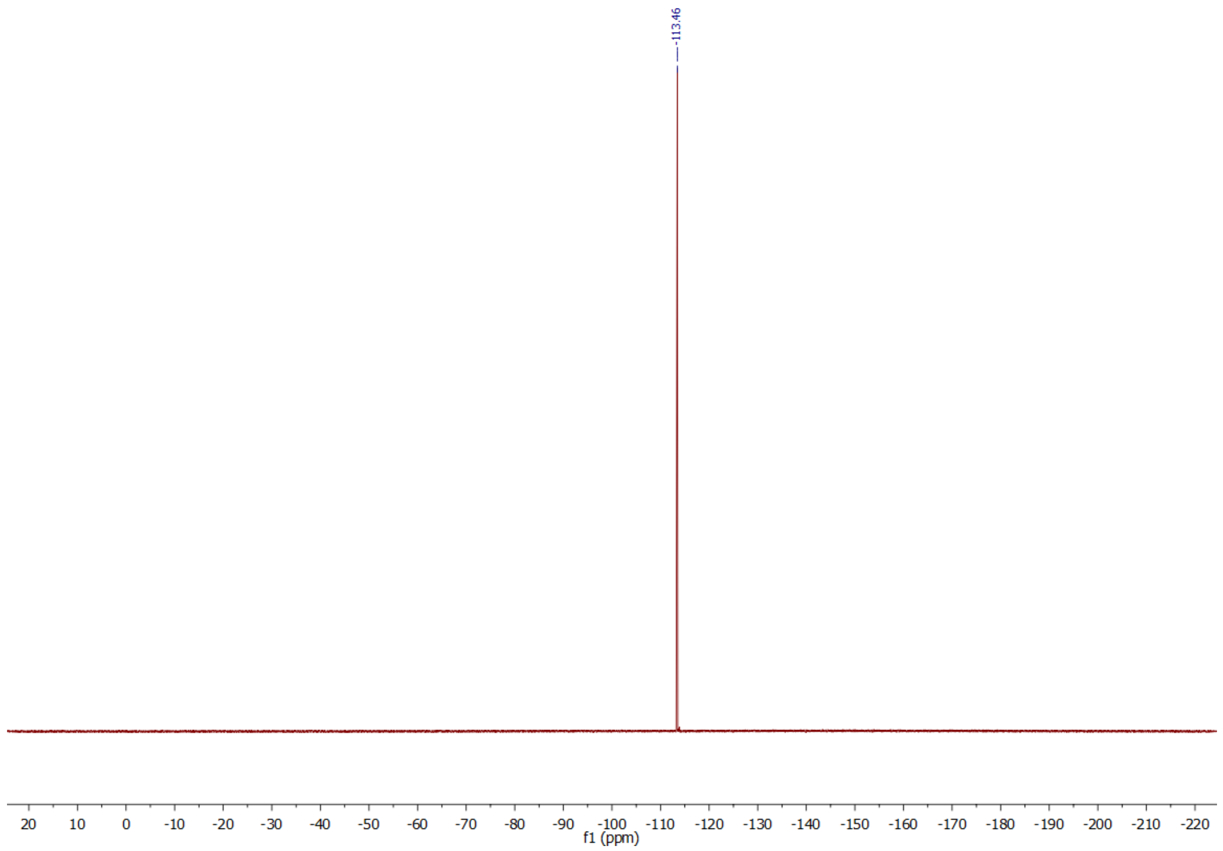
-113.46
20 10 0 -10 -20 -30 -40 -50 -60 -70 -80 -90 -100 -110 -120 -130 -140 -150 -160 -170 -180 -190 -200 -210 -220
f1 (ppm)

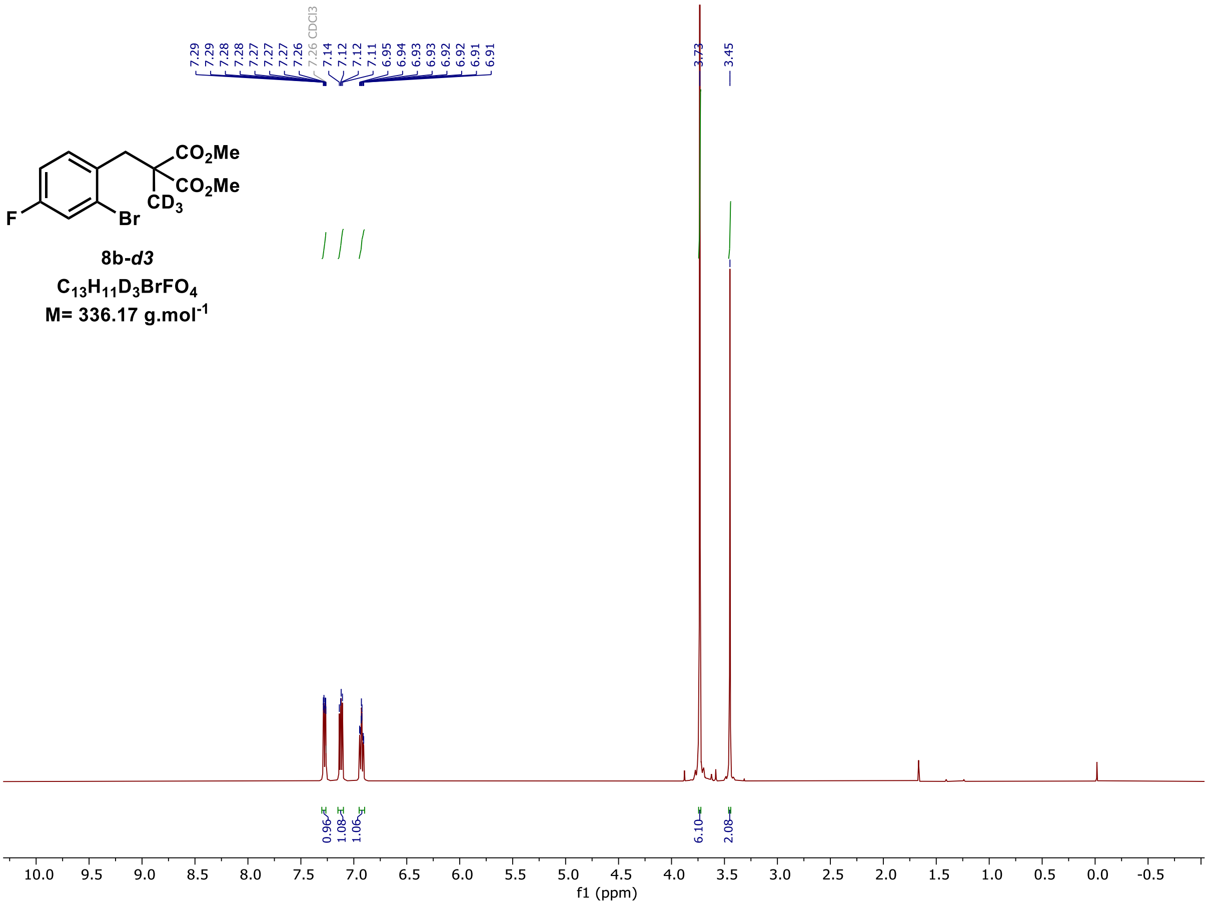

8b-*d3*

$C_{13}H_{11}D_3BrFO_4$

M= 336.17 g.mol^{-1}

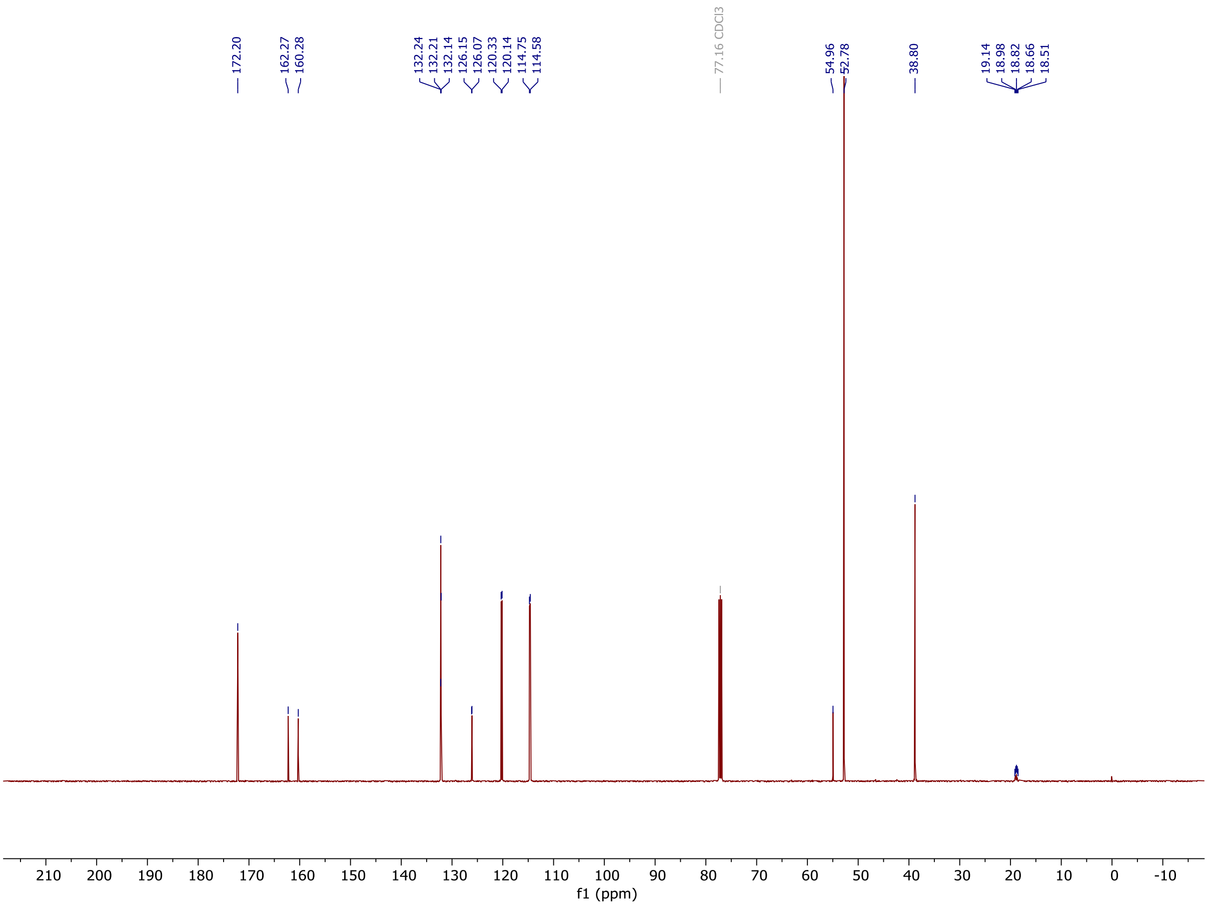

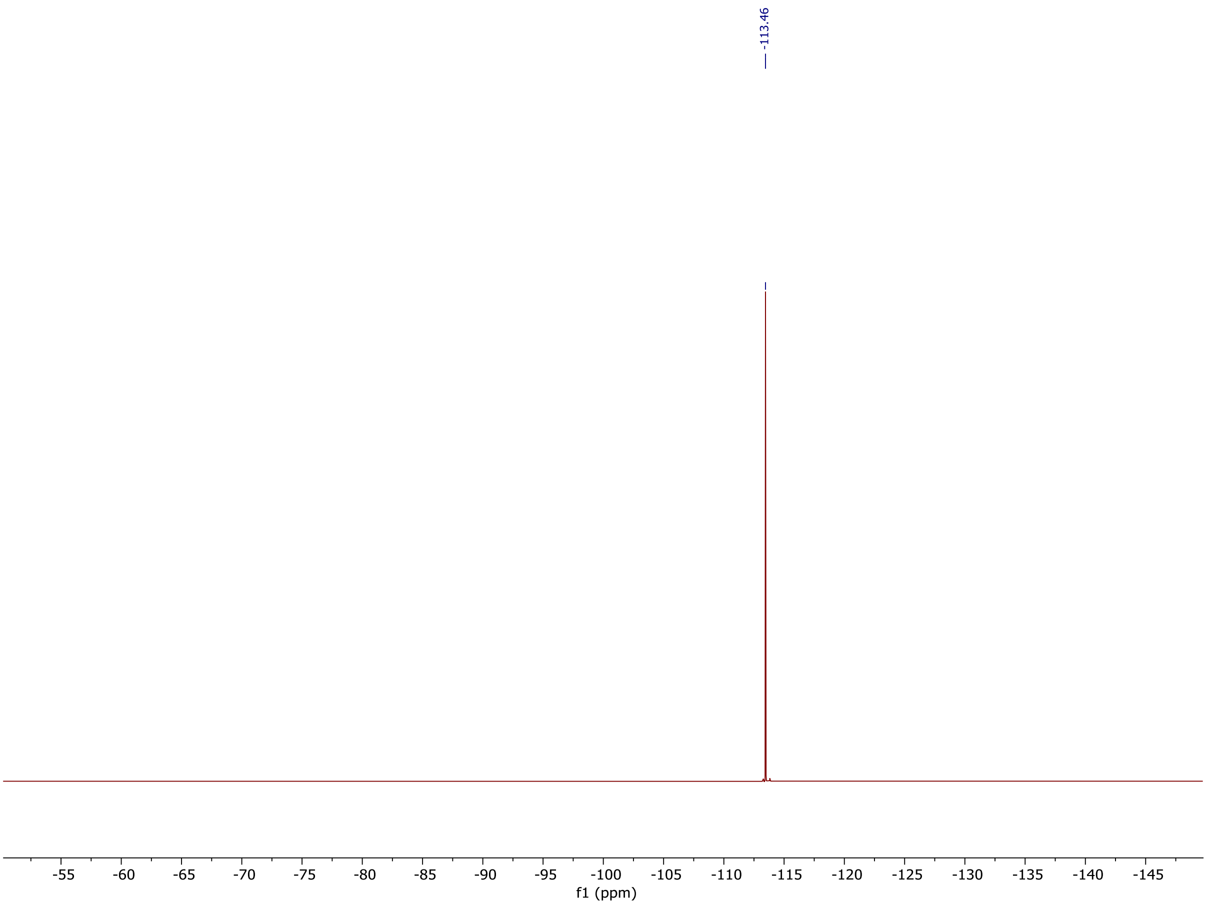
-113.46
-55
-60
-65
-70
-75
-80
-85
-90
-95
-100
-105
-110
-115
-120
-125
-130
-135
-140
-145
f1 (ppm)

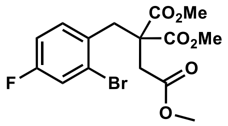

8c
$C_{15}H_{16}BrFO_6$
M= 391,19 g.mol-1

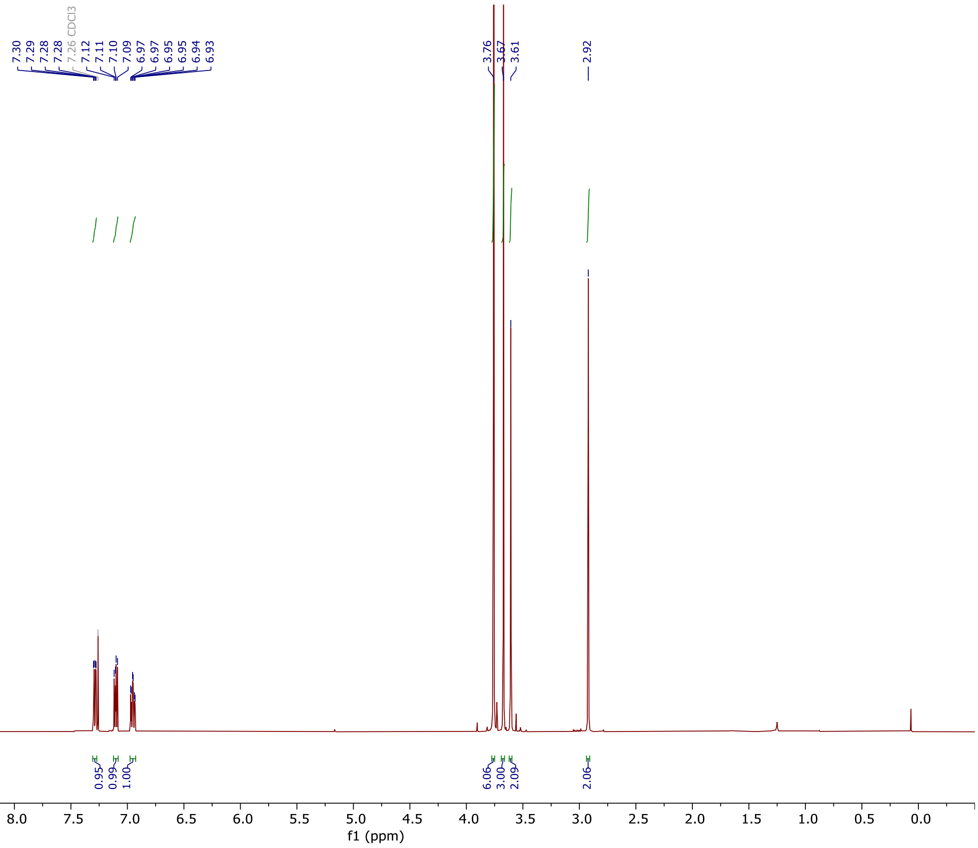

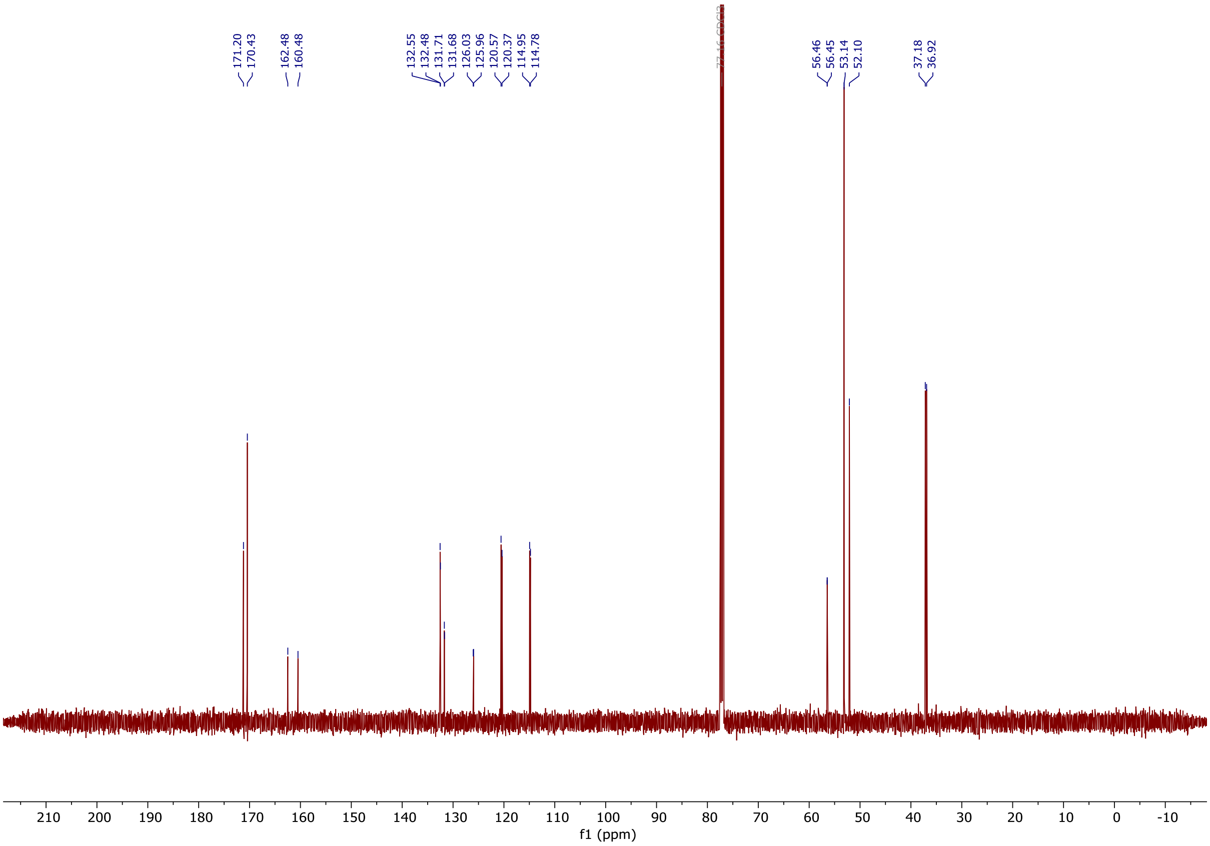

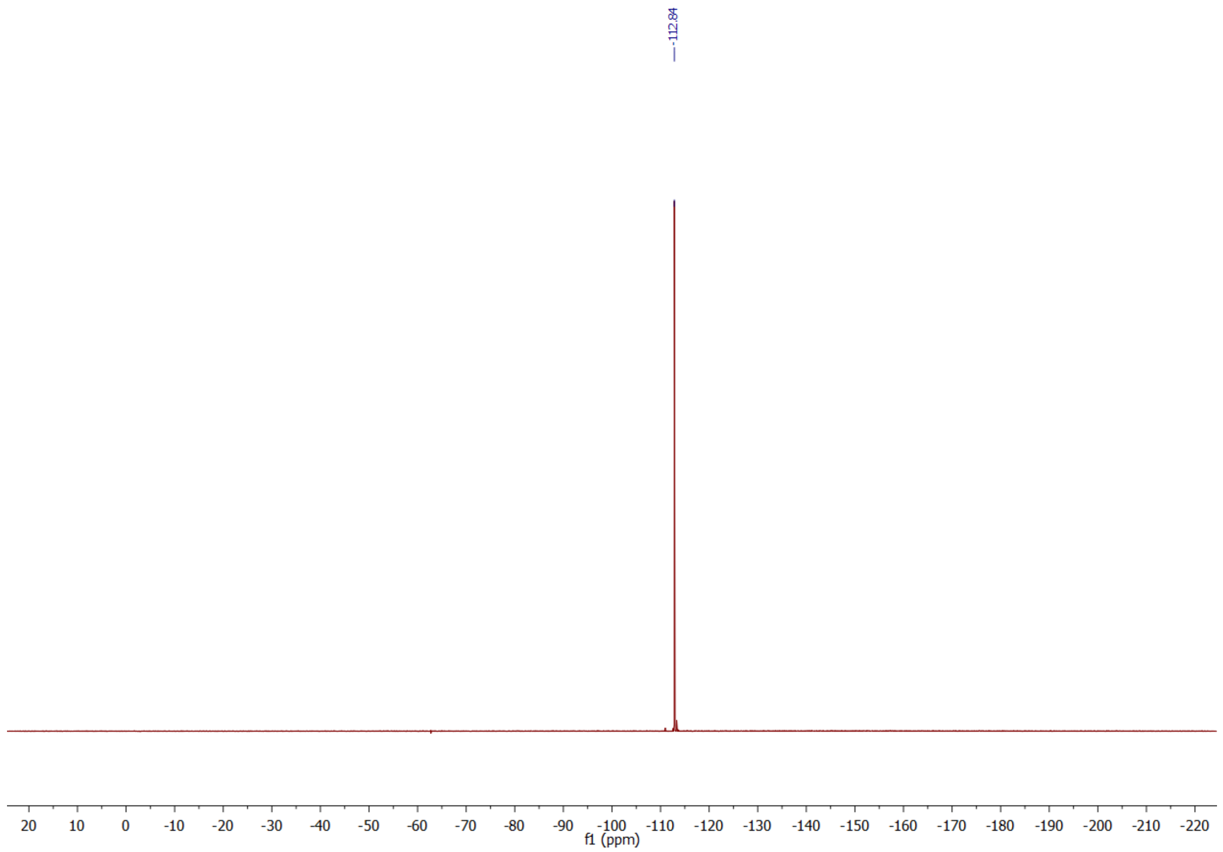
-112.84
20 10 0 -10 -20 -30 -40 -50 -60 -70 -80 -90 -100 -110 -120 -130 -140 -150 -160 -170 -180 -190 -200 -210 -220
f1 (ppm)

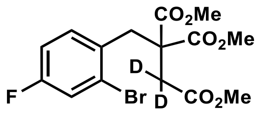

8c-*d2*

$C_{15}H_{14}D_2BrFO_6$

M= 393.20 g.mol-1

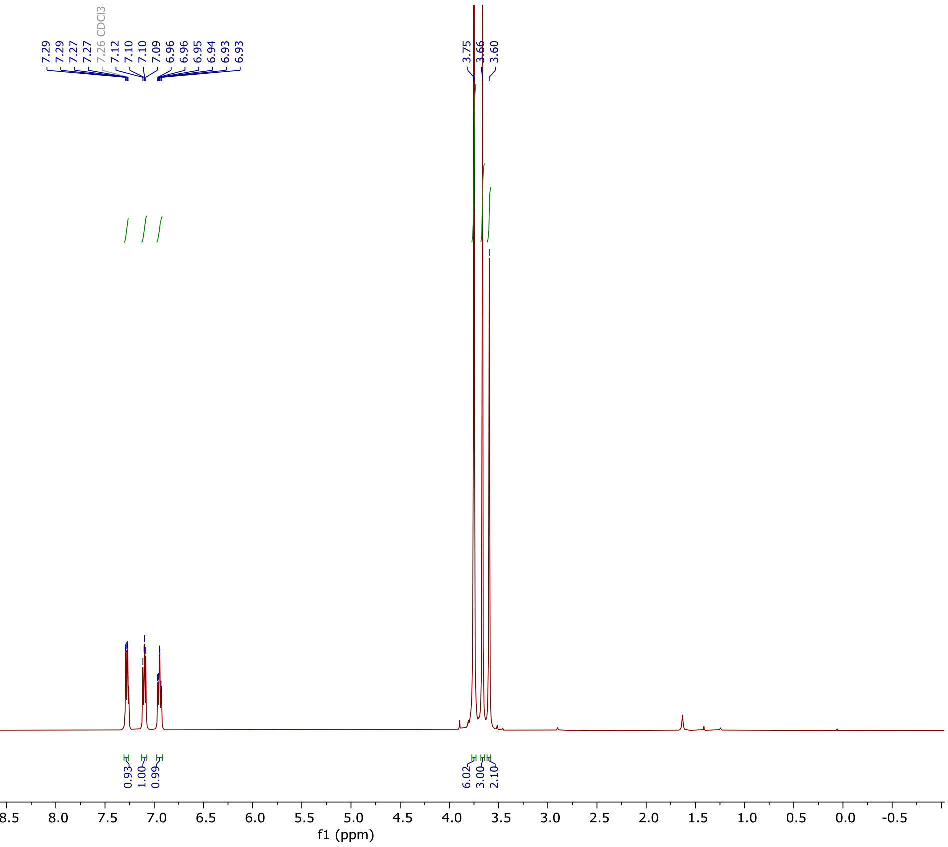

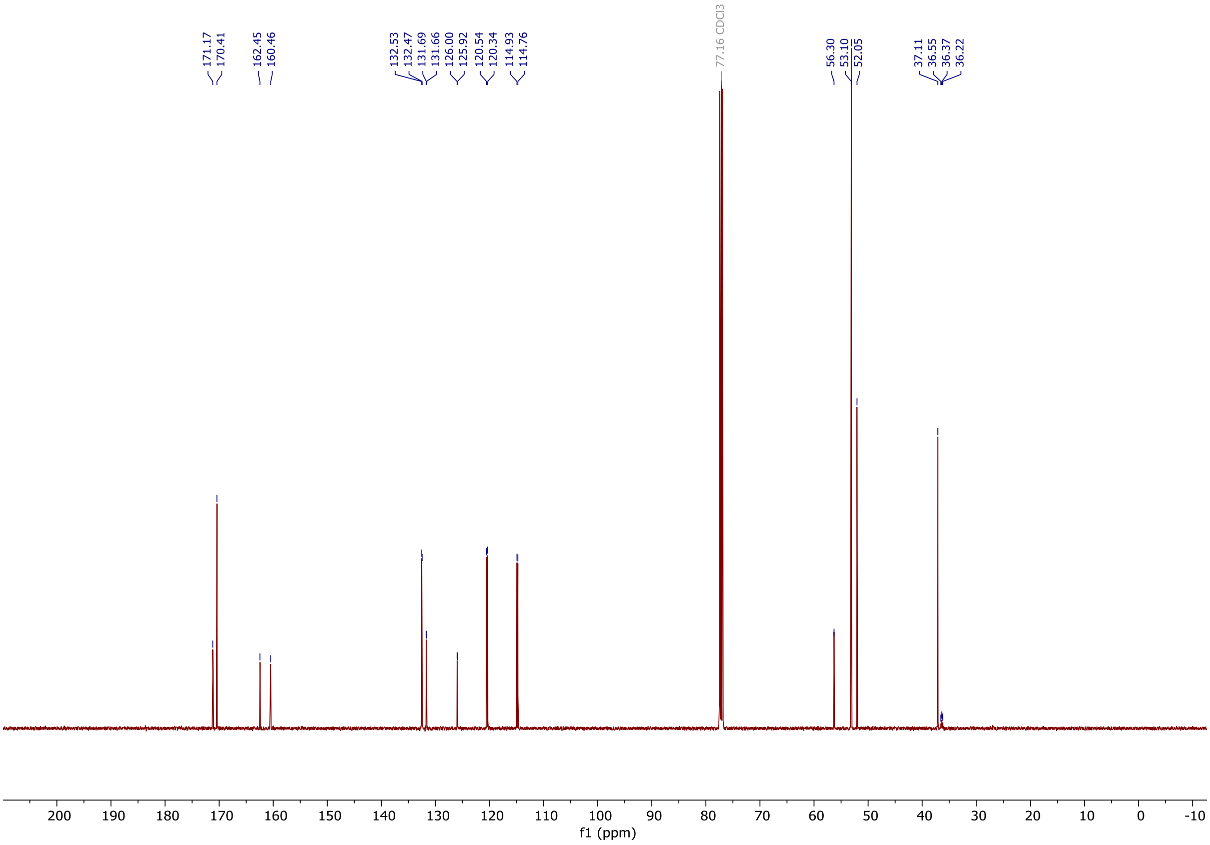

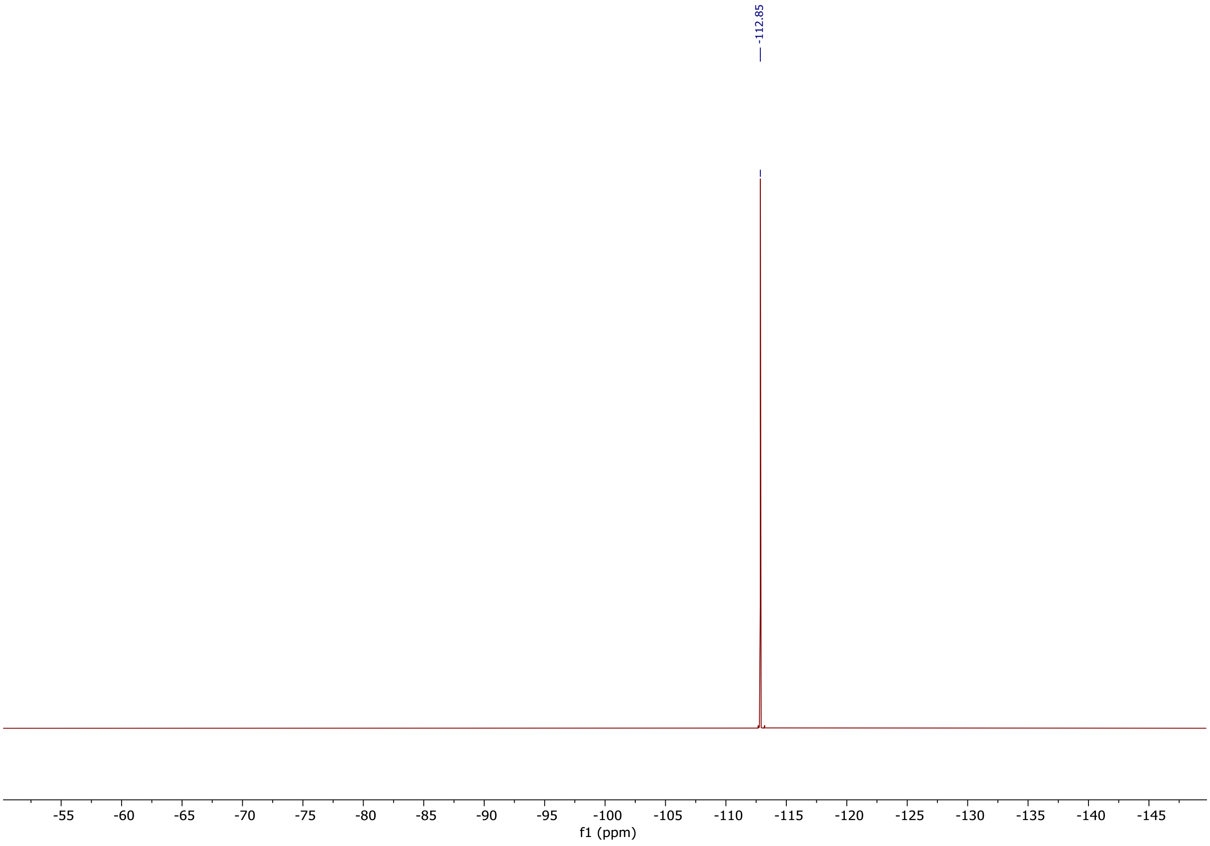

-112.85
-55
-60
-65
-70
-75
-80
-85
-90
-95
-100
-105
-110
-115
-120
-125
-130
-135
-140
-145
f1 (ppm)

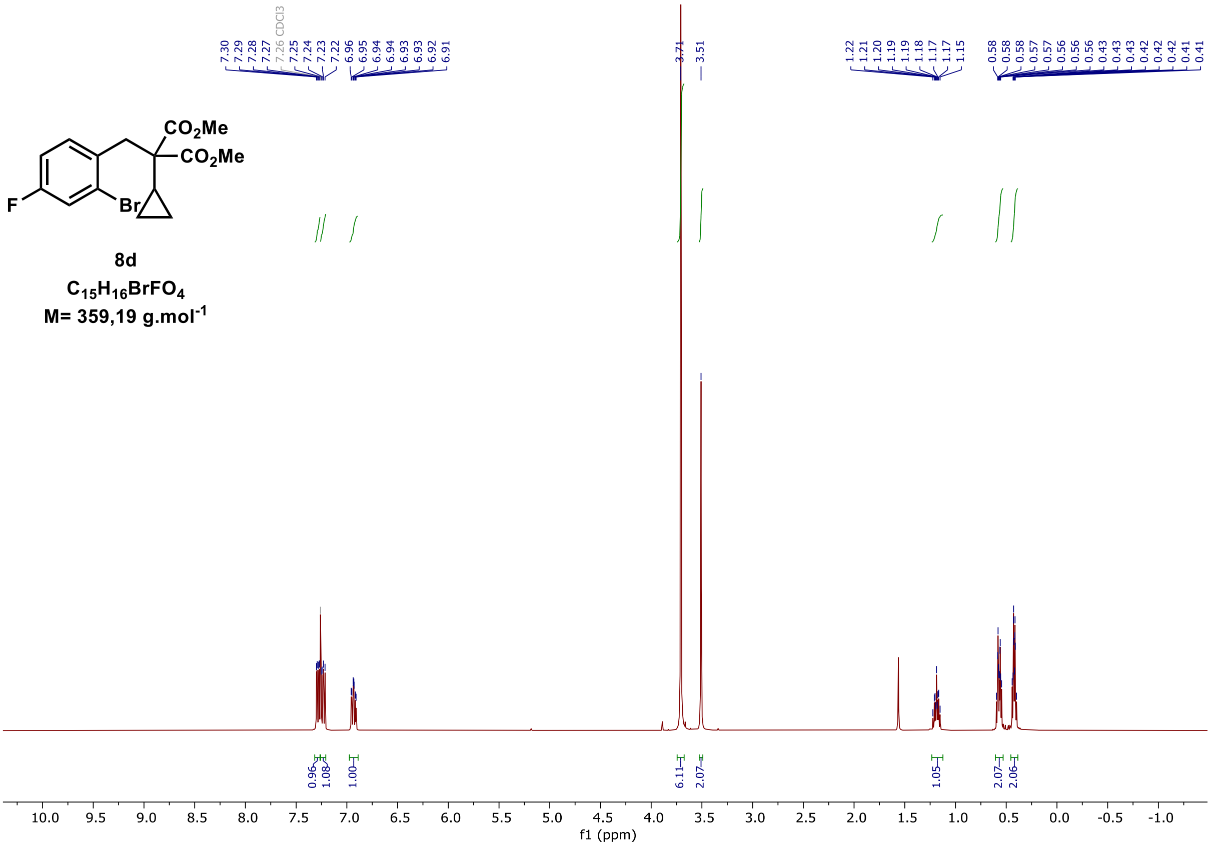

CO2Me
CO2Me
F
Br
8d
C15H16BrFO4
M= 359,19 g.mol-1
7.30
7.29
7.28
7.27
7.26 CDCl3
7.25
7.24
7.23
7.22
6.96
6.95
6.94
6.94
6.93
6.93
6.92
6.91
3.71
3.51
1.22
1.21
1.20
1.19
1.19
1.18
1.17
1.17
1.15
0.58
0.58
0.58
0.57
0.57
0.56
0.56
0.56
0.43
0.43
0.43
0.42
0.42
0.42
0.41
0.41
0.96
1.08
1.00
6.11
2.07
1.05
2.07
2.06
10.0
9.5
9.0
8.5
8.0
7.5
7.0
6.5
6.0
5.5
5.0
4.5
4.0
3.5
3.0
2.5
2.0
1.5
1.0
0.5
0.0
-0.5
-1.0
f1 (ppm)

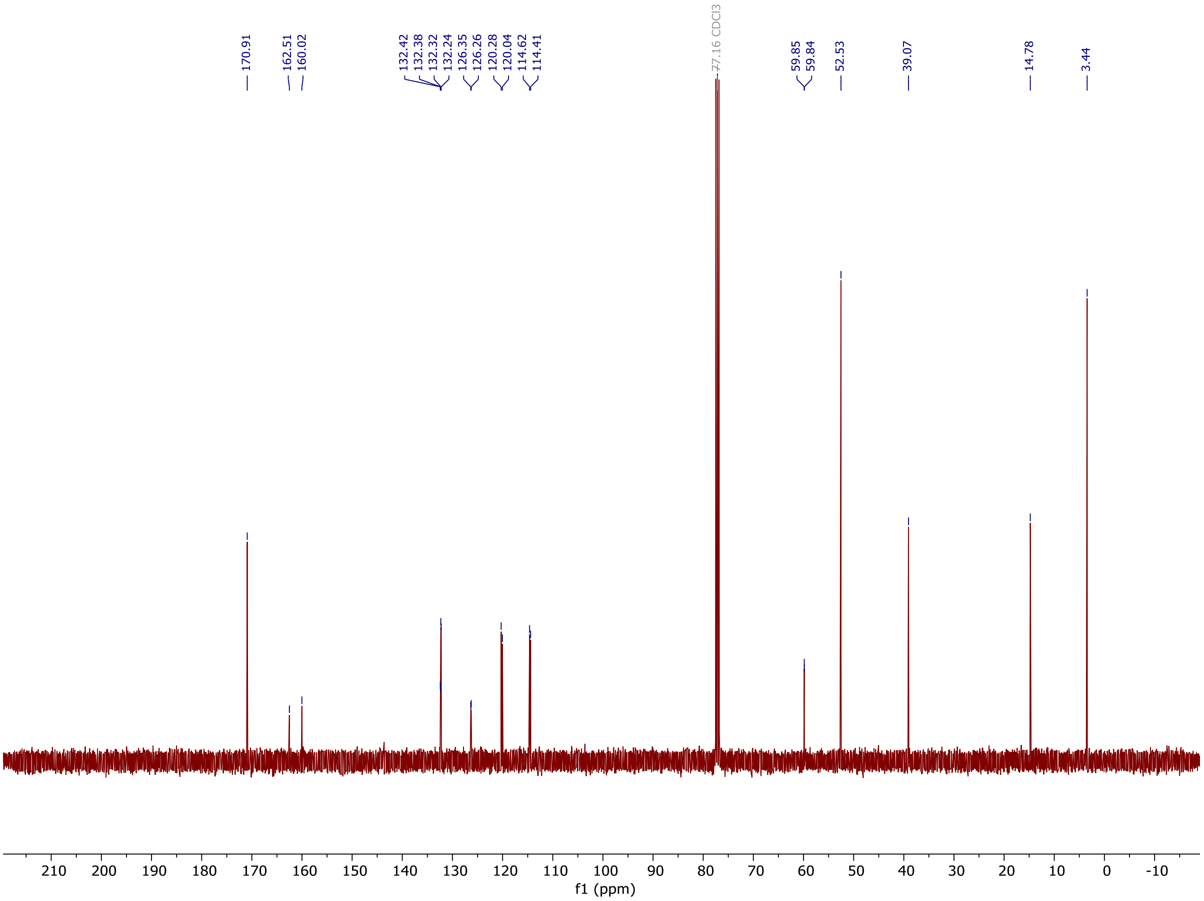

170.91
162.51
160.02
132.42
132.38
132.32
132.24
126.35
126.26
120.28
120.04
114.62
114.41
77.16 CDCl3
59.85
59.84
52.53
39.07
14.78
3.44
210
200
190
180
170
160
150
140
130
120
110
100
90
80
70
60
50
40
30
20
10
0
-10
f1 (ppm)

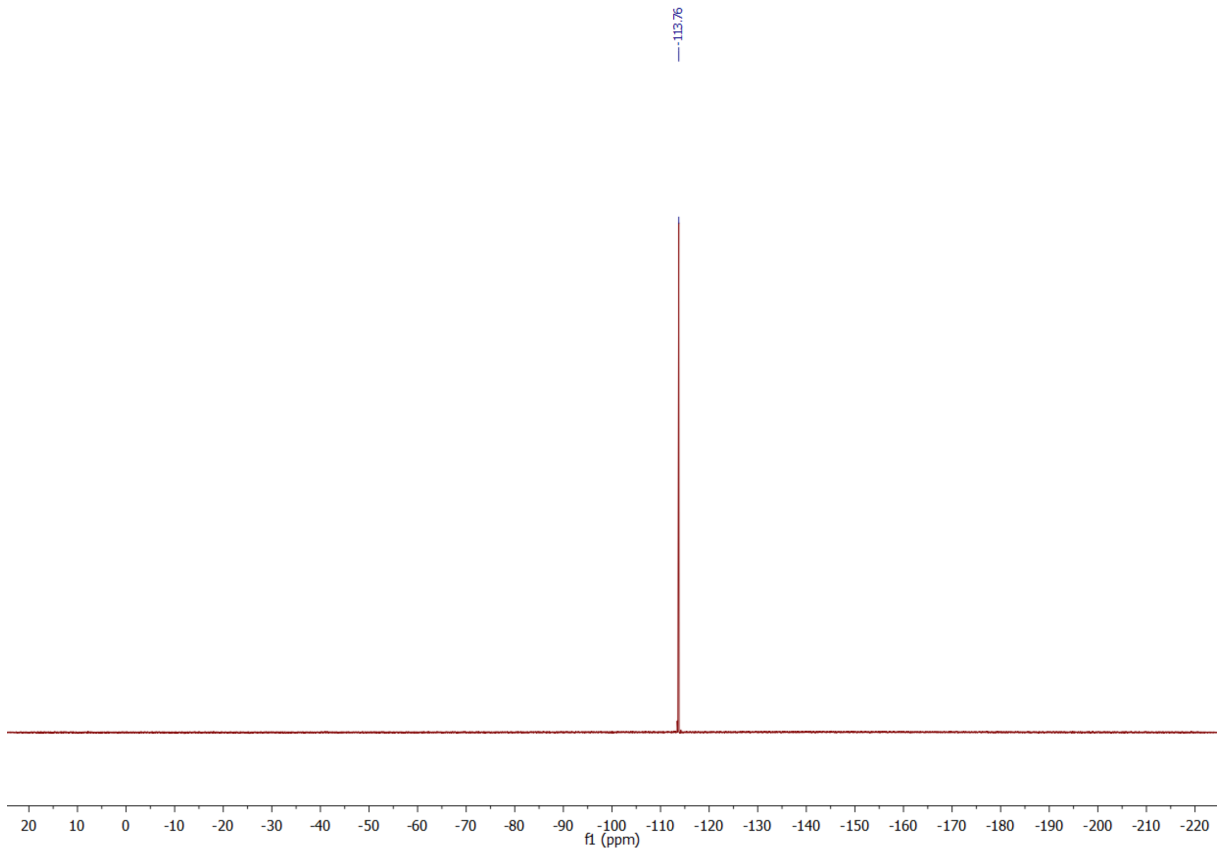
-113.76
20
10
0
-10
-20
-30
-40
-50
-60
-70
-80
-90
-100
-110
-120
-130
-140
-150
-160
-170
-180
-190
-200
-210
-220
f1 (ppm)

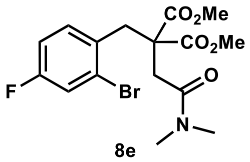

8e

$C_{16}H_{19}BrFNO_5$

M= 404,23 g.mol-1

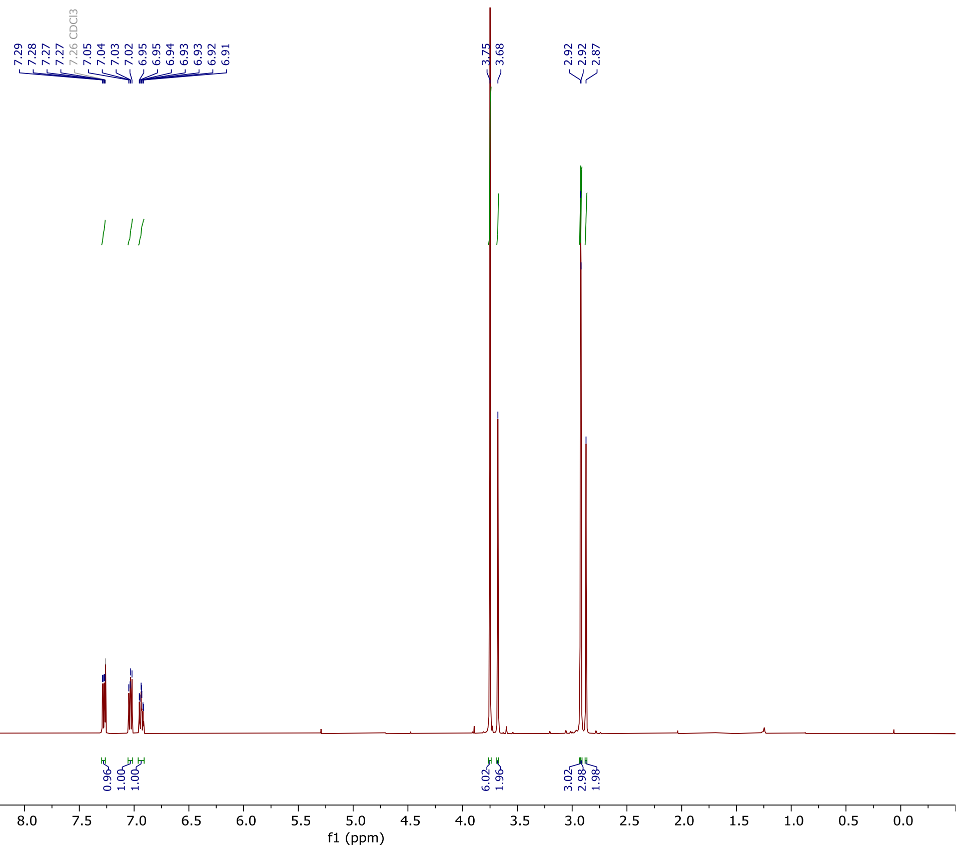

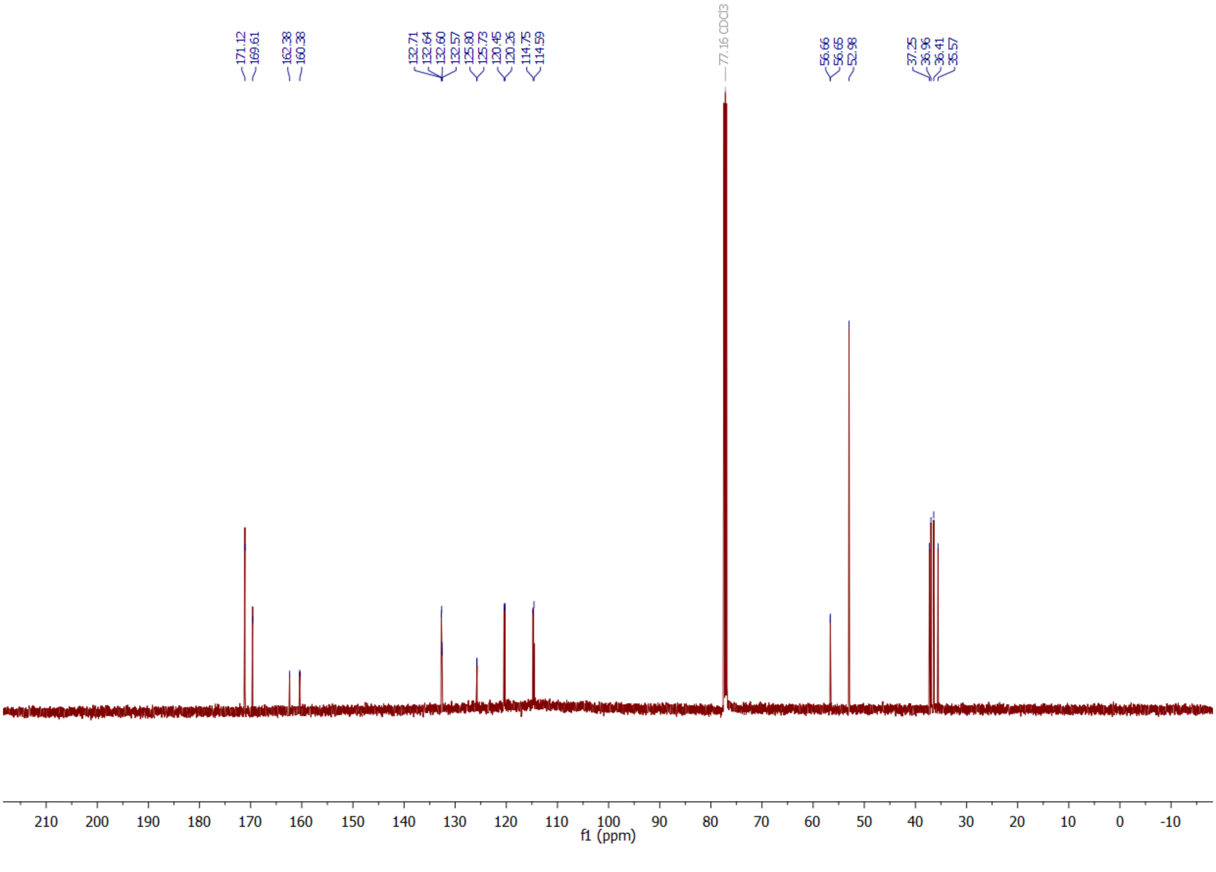

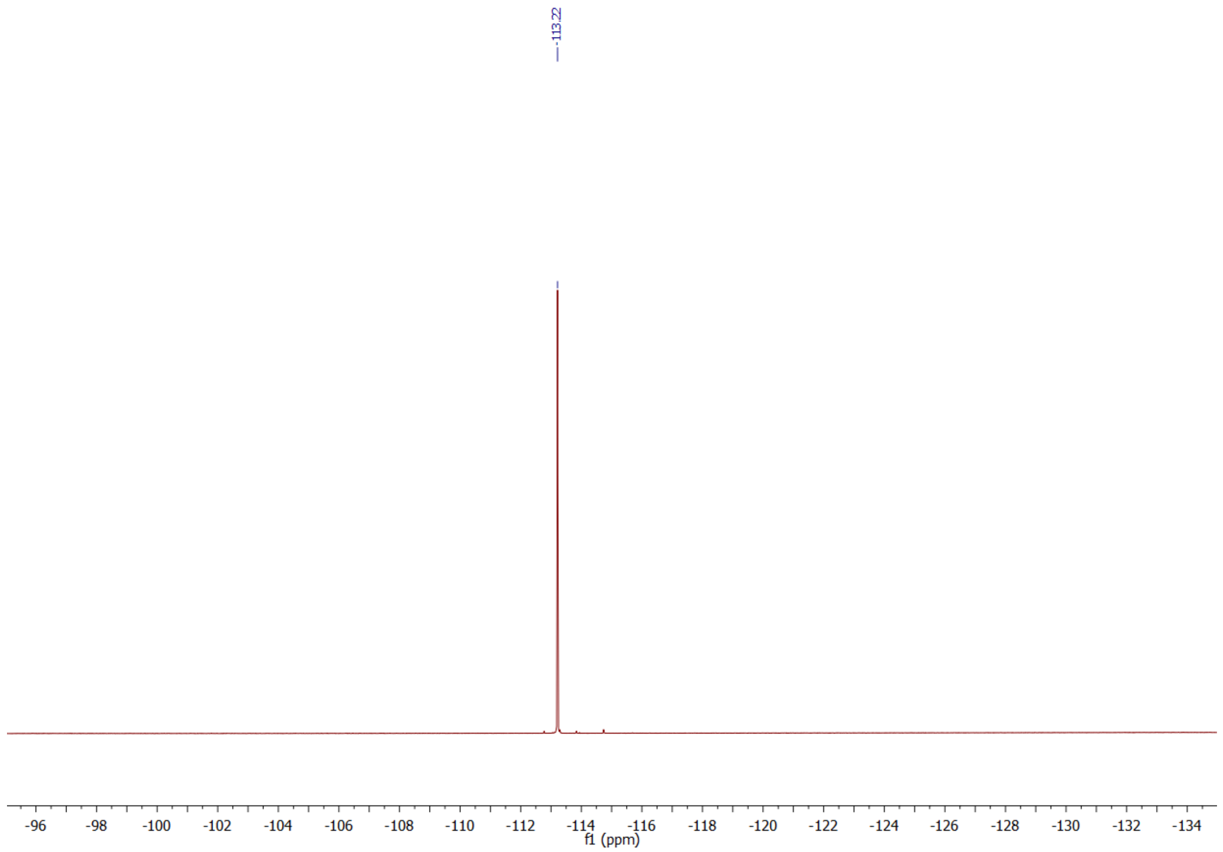
-113.22
-96
-98
-100
-102
-104
-106
-108
-110
-112
-114
-116
-118
-120
-122
-124
-126
-128
-130
-132
-134
f1 (ppm)

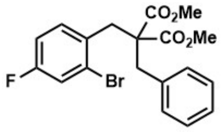

8f

$C_{19}H_{18}BrFO_4$

M= 409,5 g.mol^{-1}

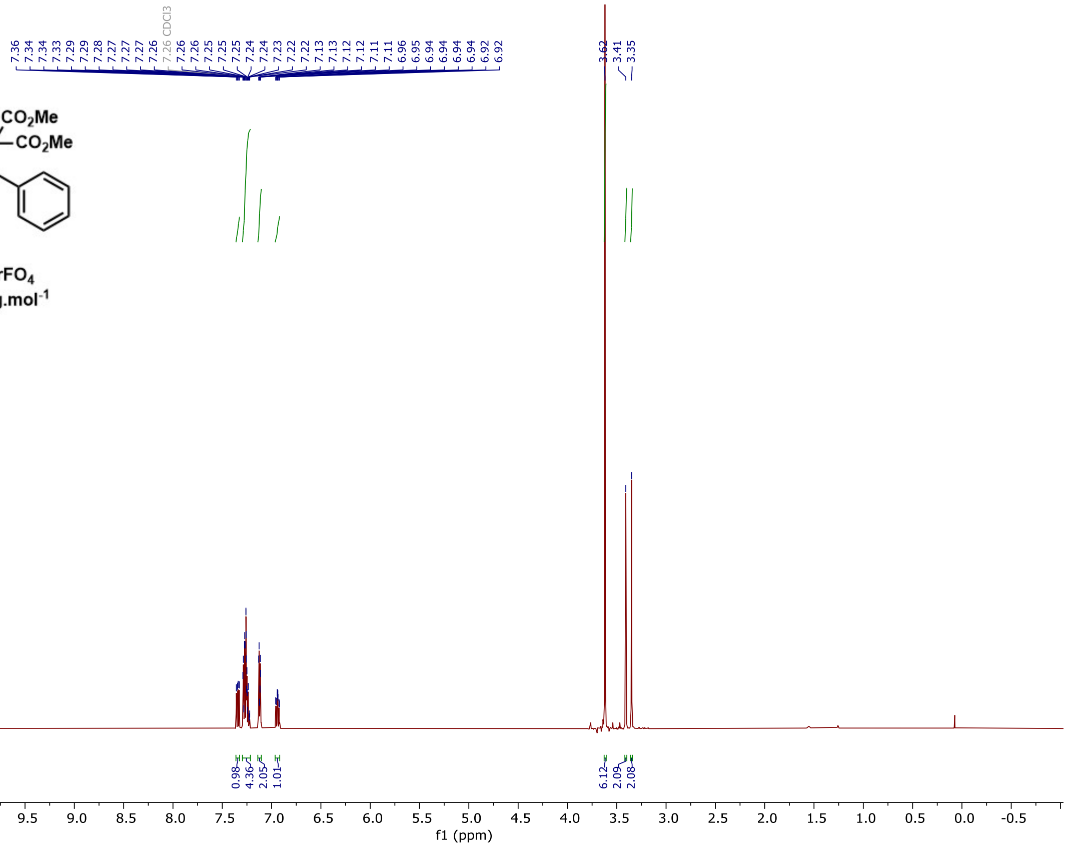

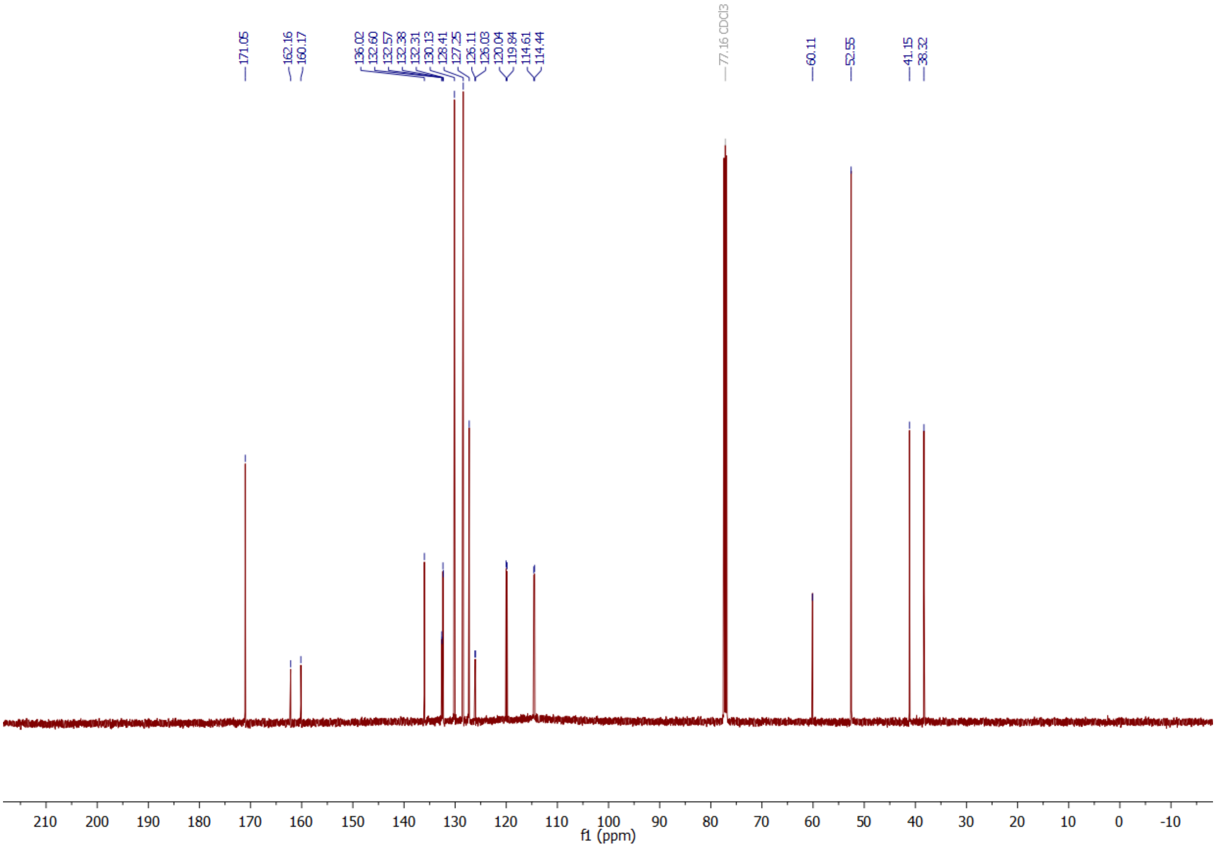

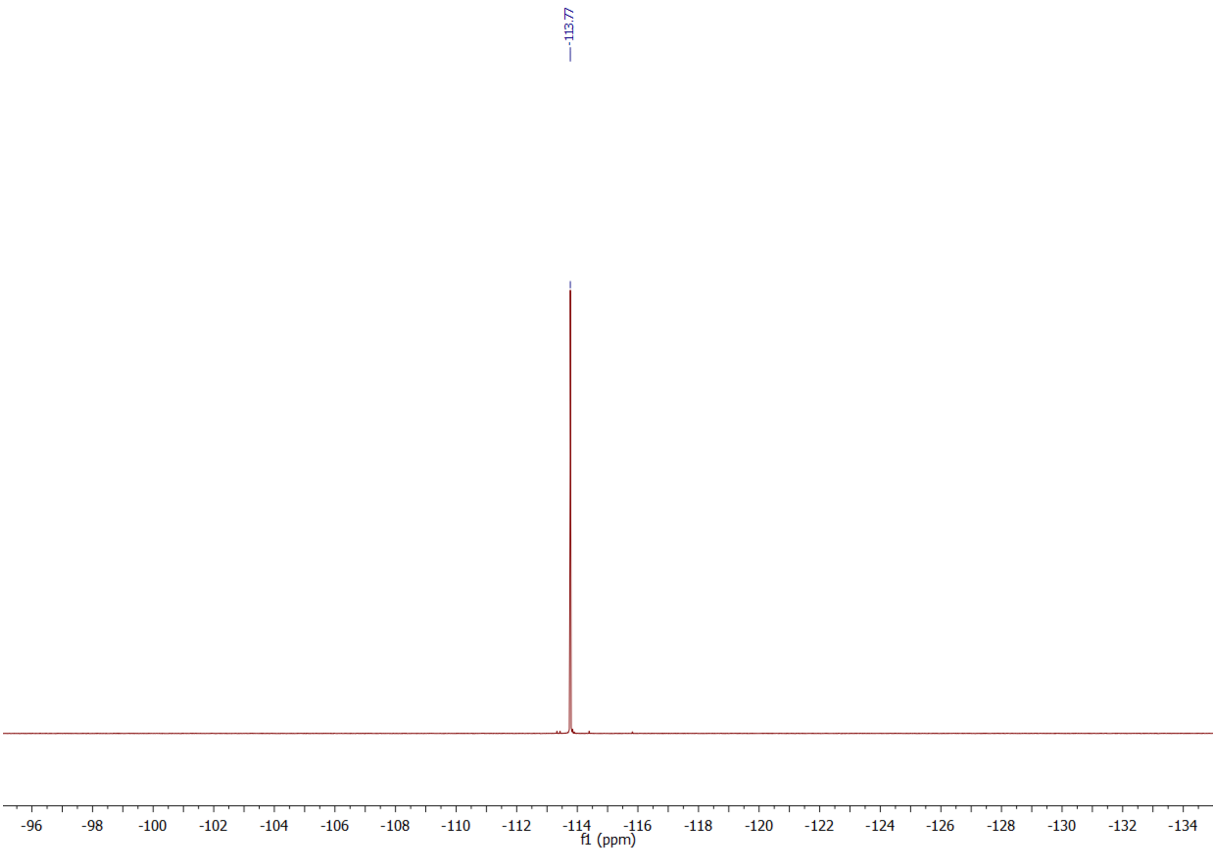
-113.77
-96
-98
-100
-102
-104
-106
-108
-110
-112
-114
-116
-118
-120
-122
-124
-126
-128
-130
-132
-134
f1 (ppm)

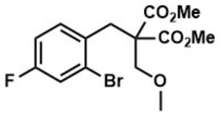

8g

$C_{14}H_{16}BrFO_5$

M= 363,18 g.mol^{-1}

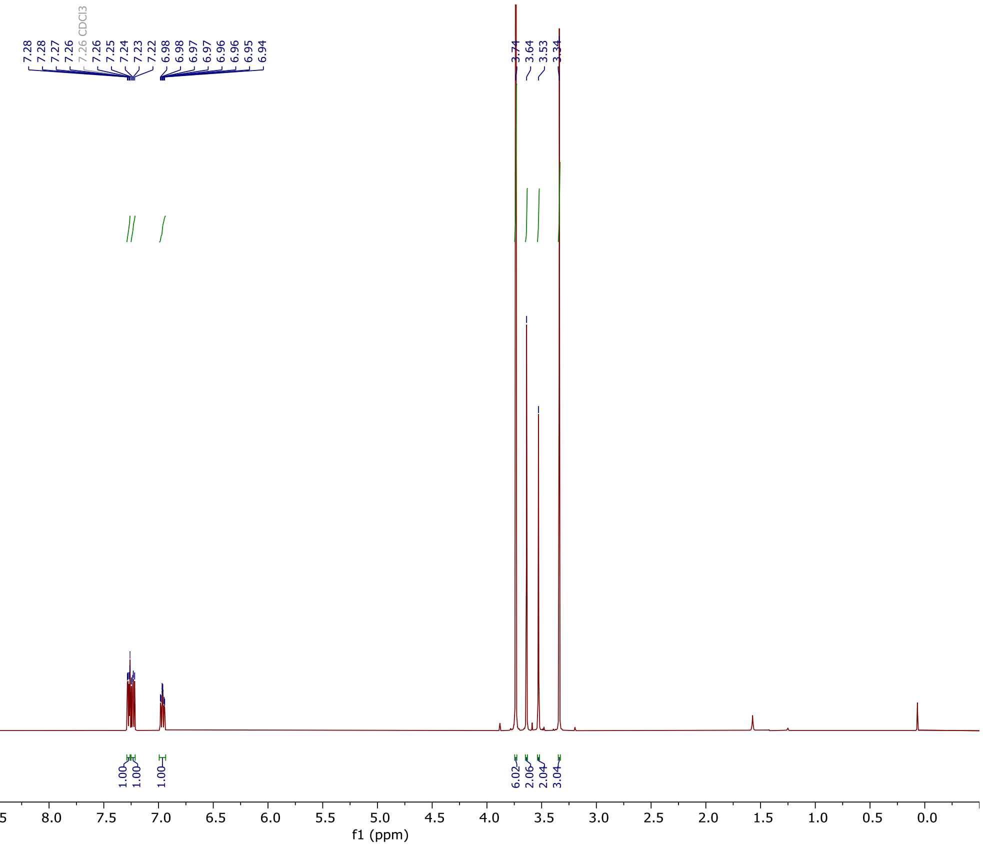

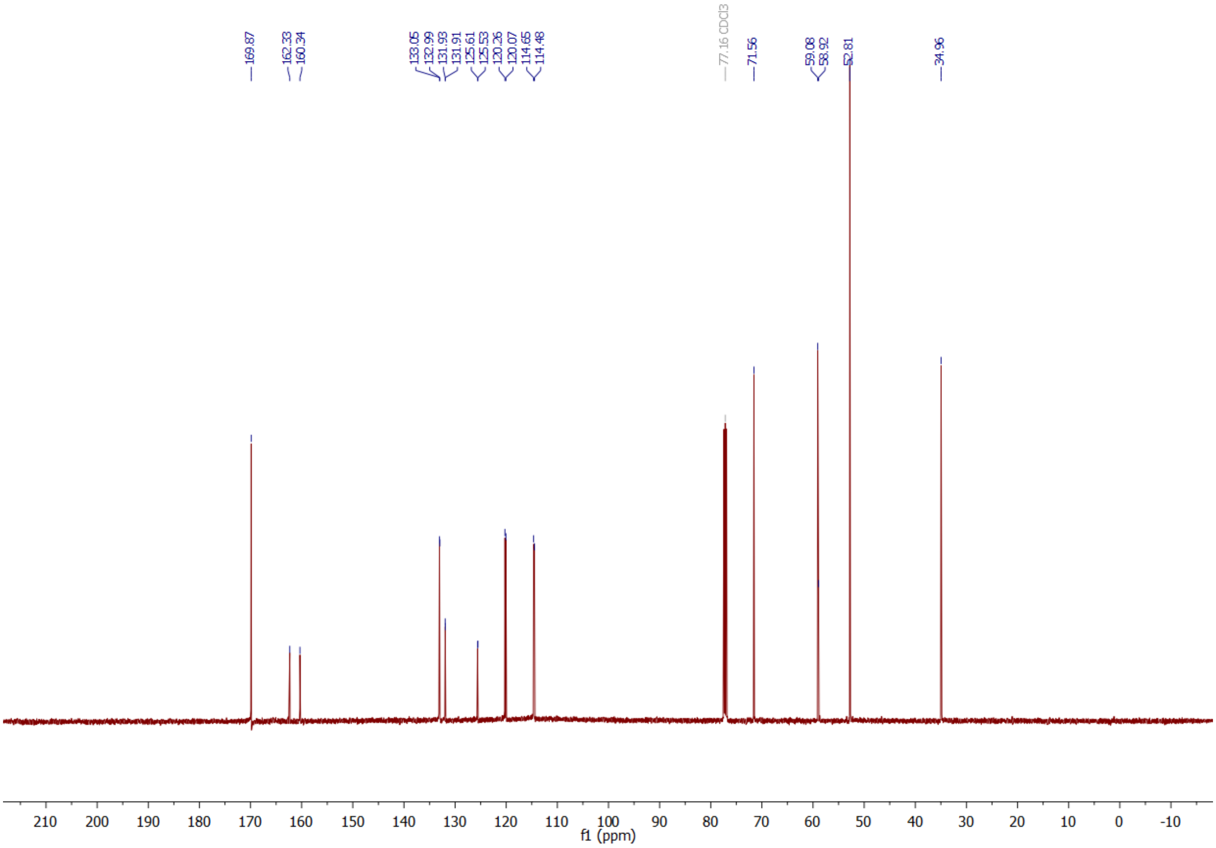

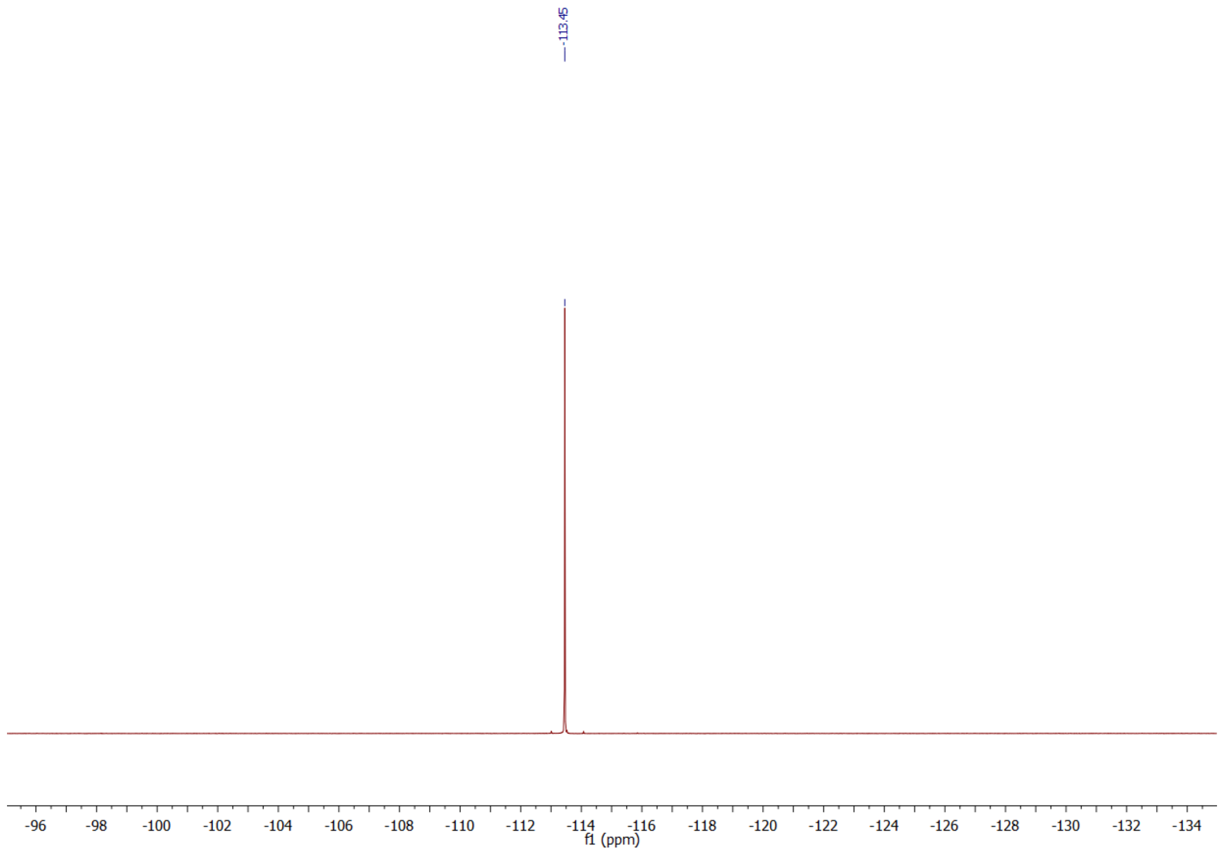
-113.45
-96
-98
-100
-102
-104
-106
-108
-110
-112
-114
-116
-118
-120
-122
-124
-126
-128
-130
-132
-134
f1 (ppm)

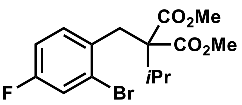

8h

$C_{15}H_{18}BrFO_4$

M = 361.21 g.mol^{-1}

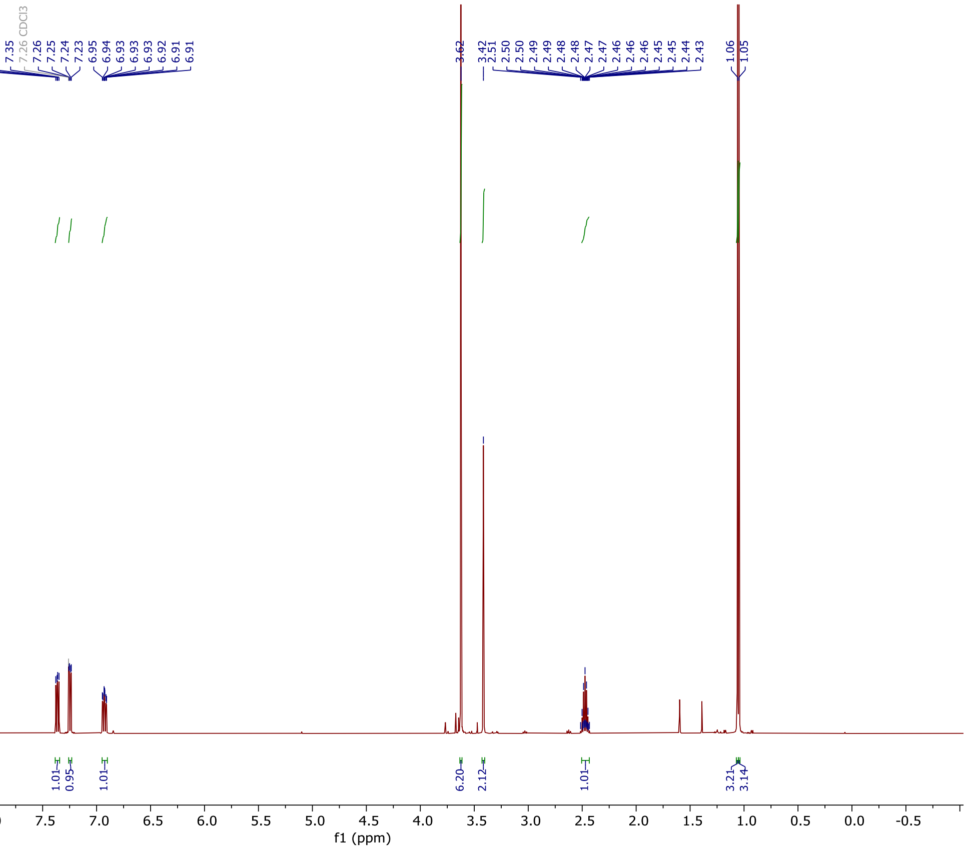

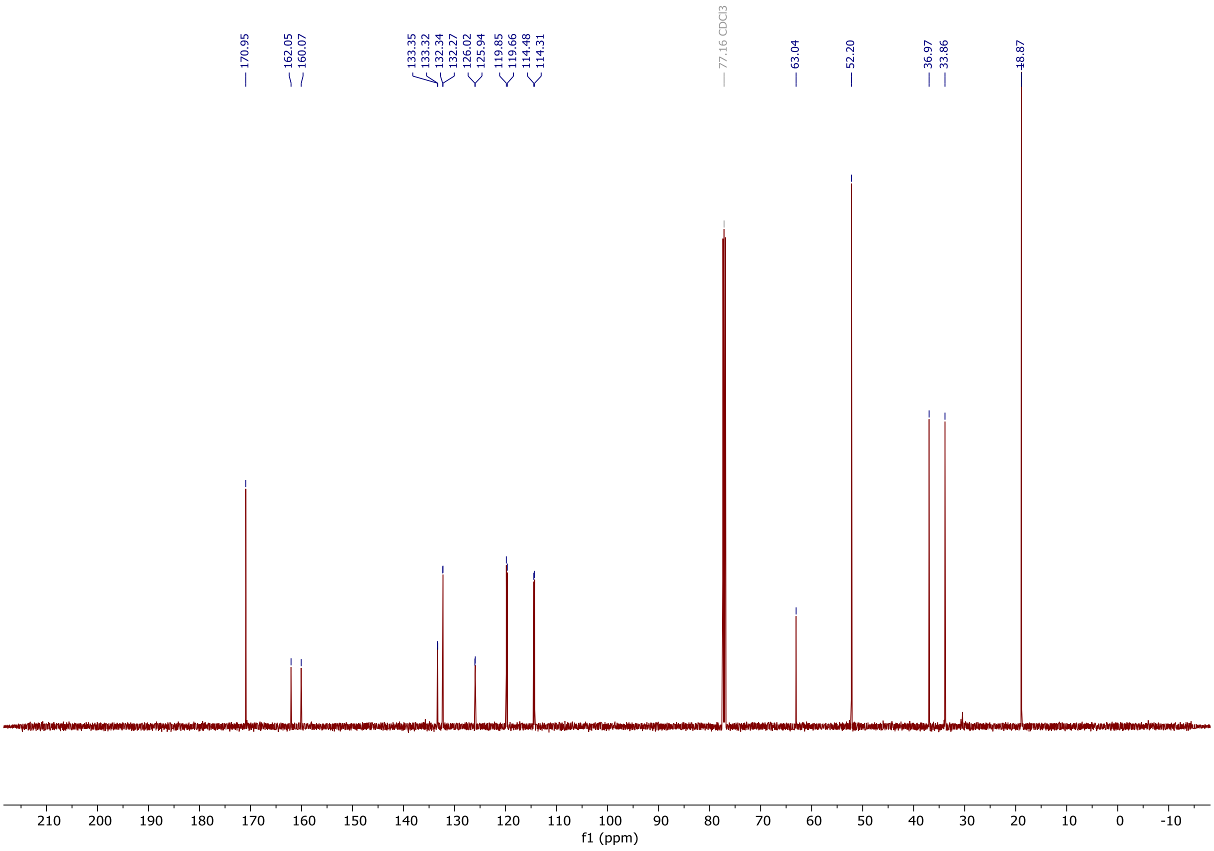

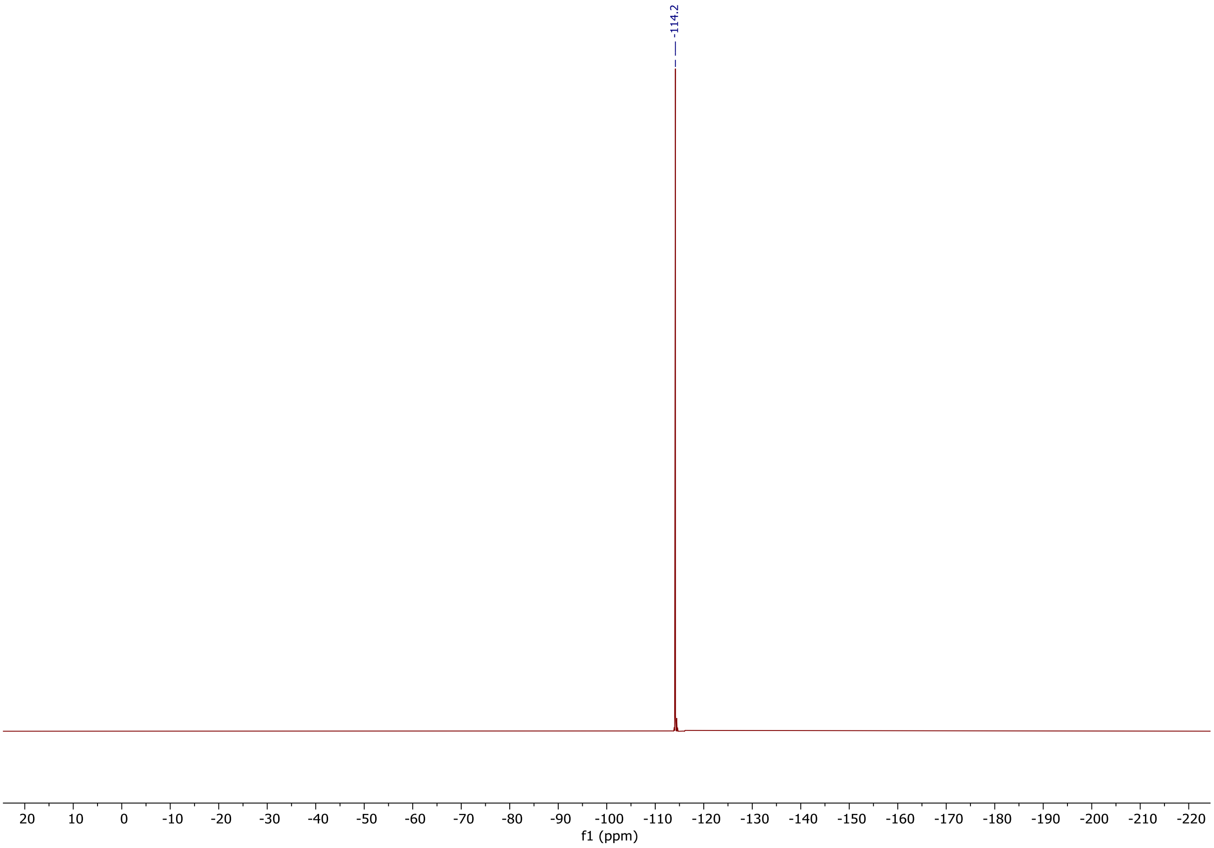

-114.2
20 10 0 -10 -20 -30 -40 -50 -60 -70 -80 -90 -100 -110 -120 -130 -140 -150 -160 -170 -180 -190 -200 -210 -220
f1 (ppm)

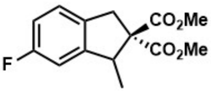

9a
$C_{14}H_{15}FO_4$
M= 266,27 g.mol-1

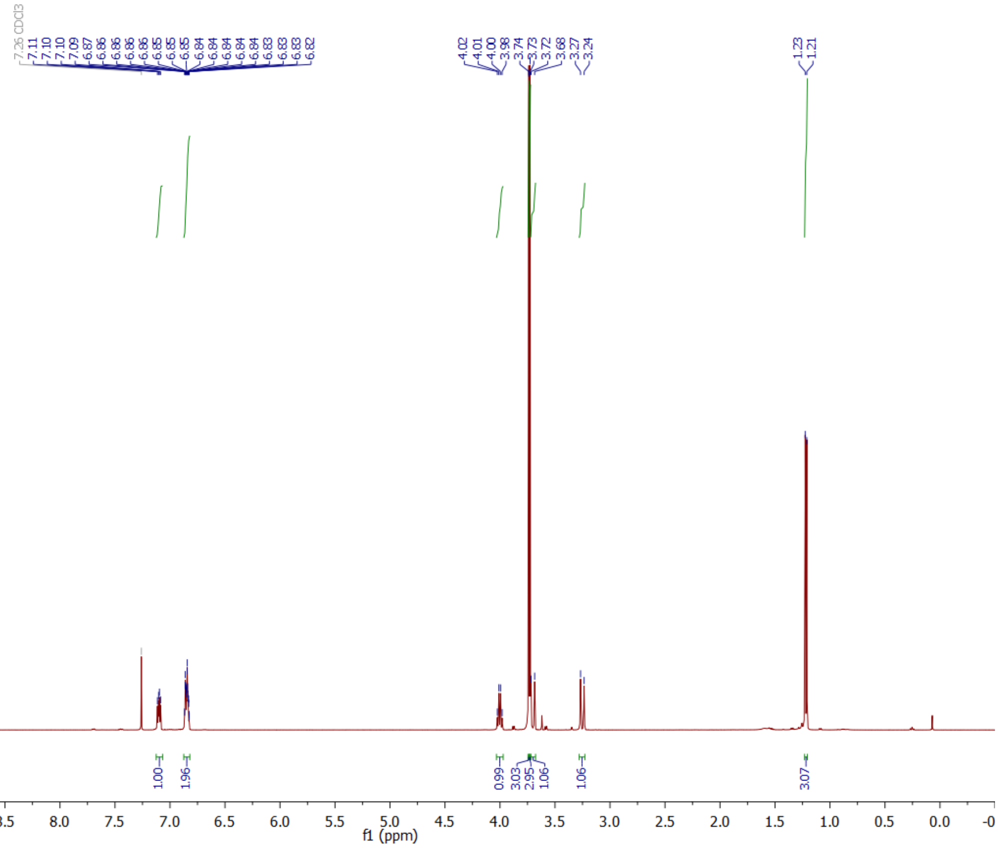

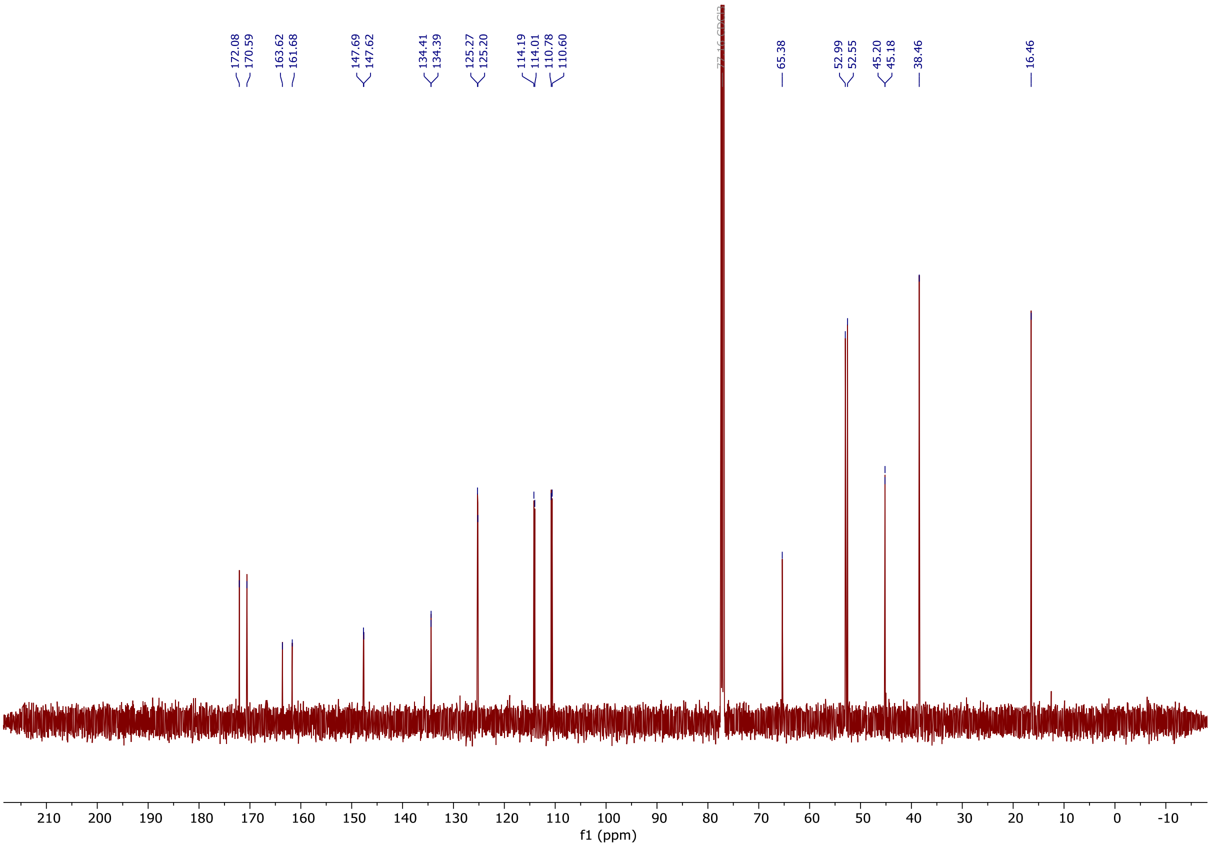

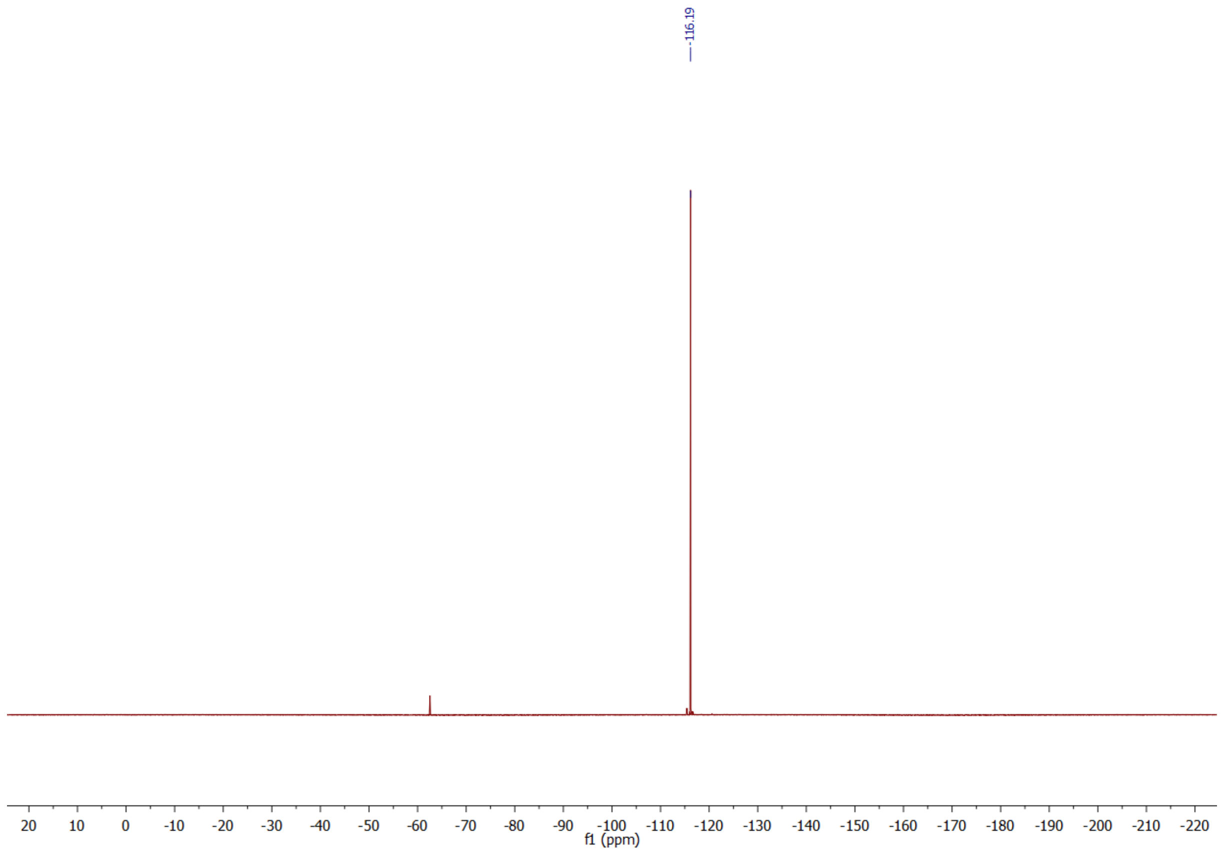
-116.19
20
10
0
-10
-20
-30
-40
-50
-60
-70
-80
-90
-100
-110
-120
-130
-140
-150
-160
-170
-180
-190
-200
-210
-220
f1 (ppm)

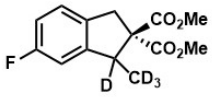

9a-*d4*
$C_{14}H_{11}D_4FO_4$
M= 270,29 g.mol-1

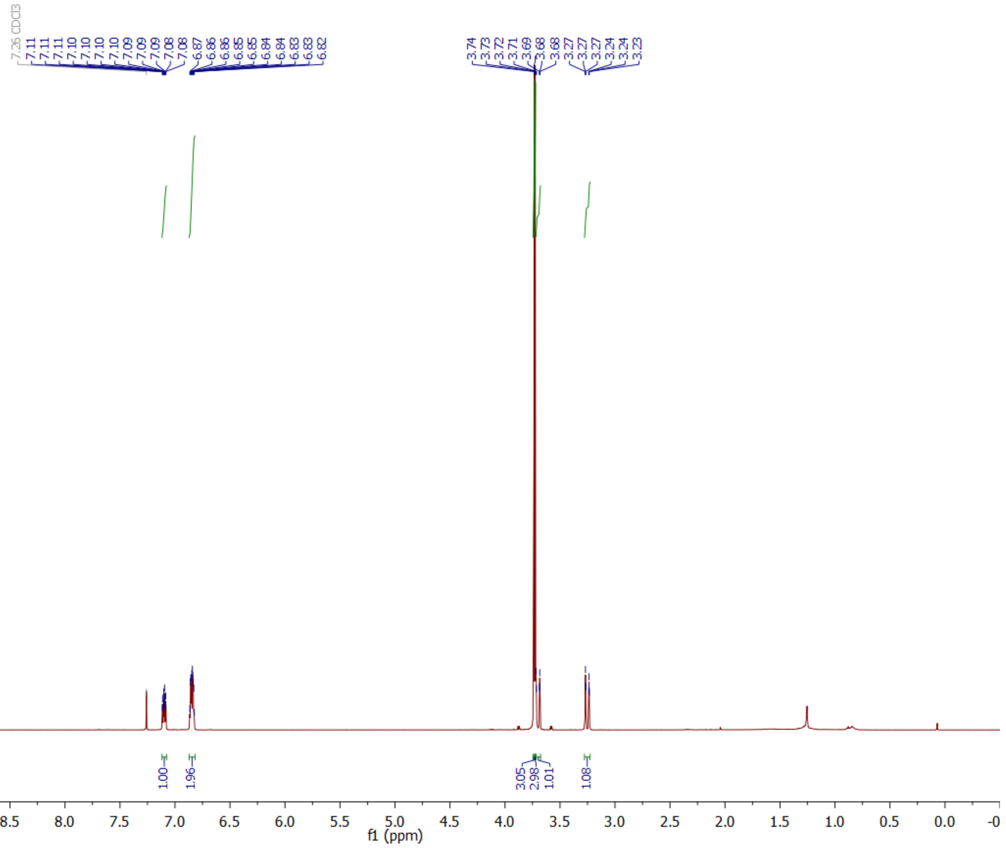

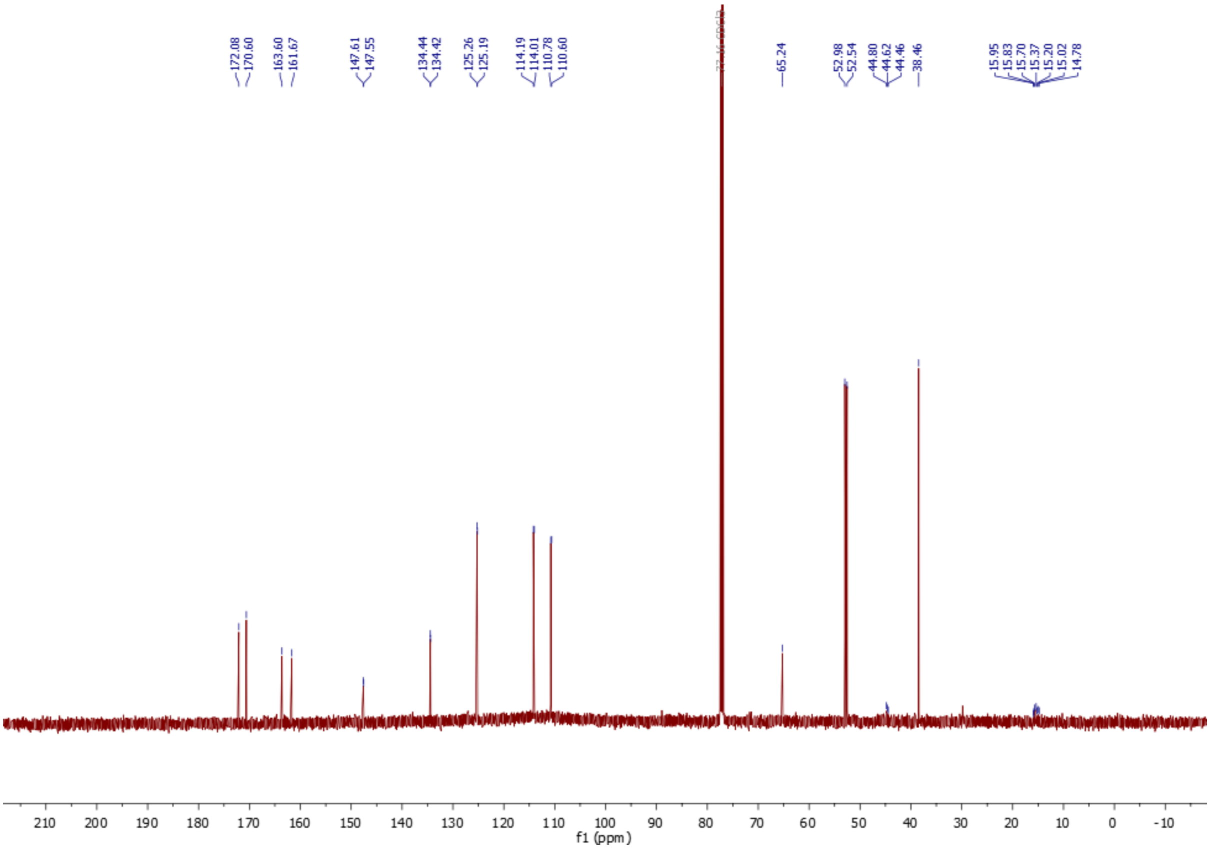

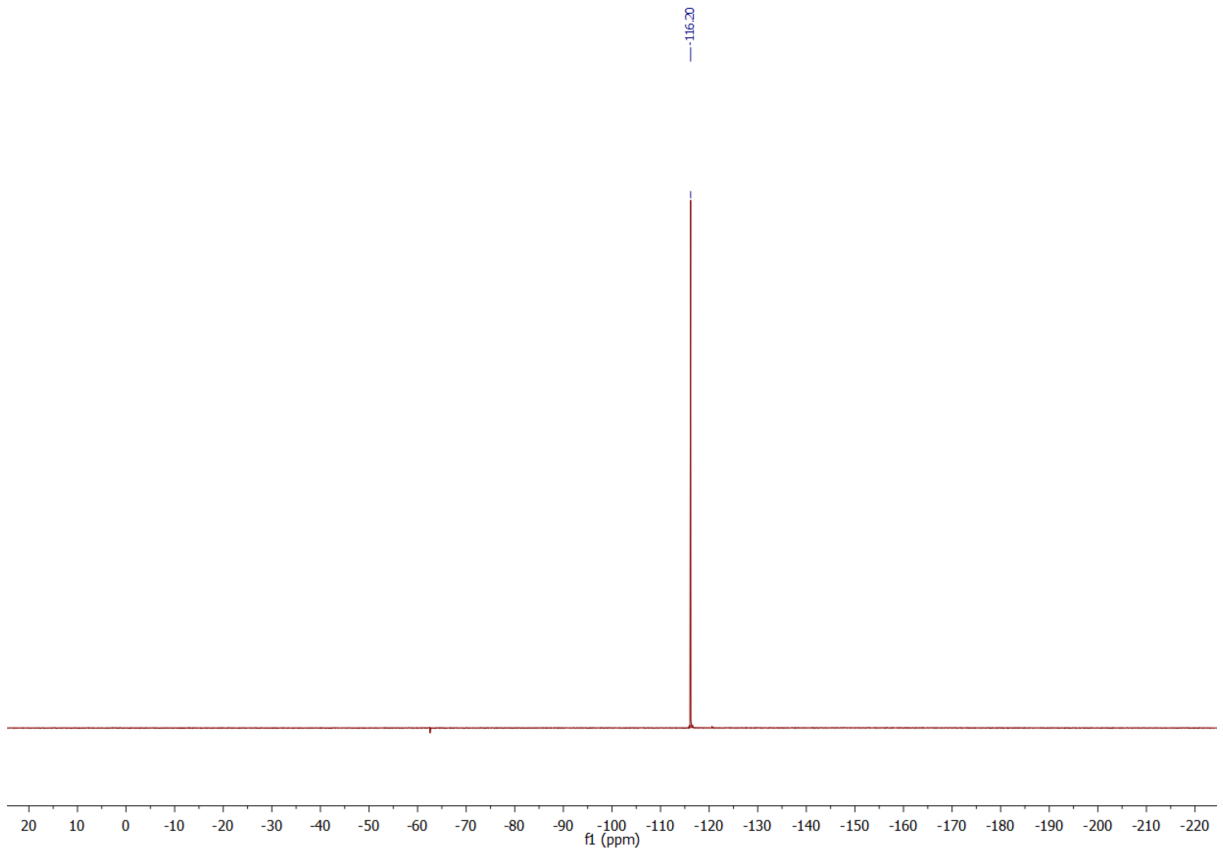

-116.20
20
10
0
-10
-20
-30
-40
-50
-60
-70
-80
-90
-100
-110
-120
-130
-140
-150
-160
-170
-180
-190
-200
-210
-220
f1 (ppm)

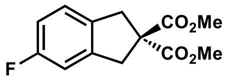

9b
$C_{13}H_{13}FO_4$
M= 252,24 g.mol-1

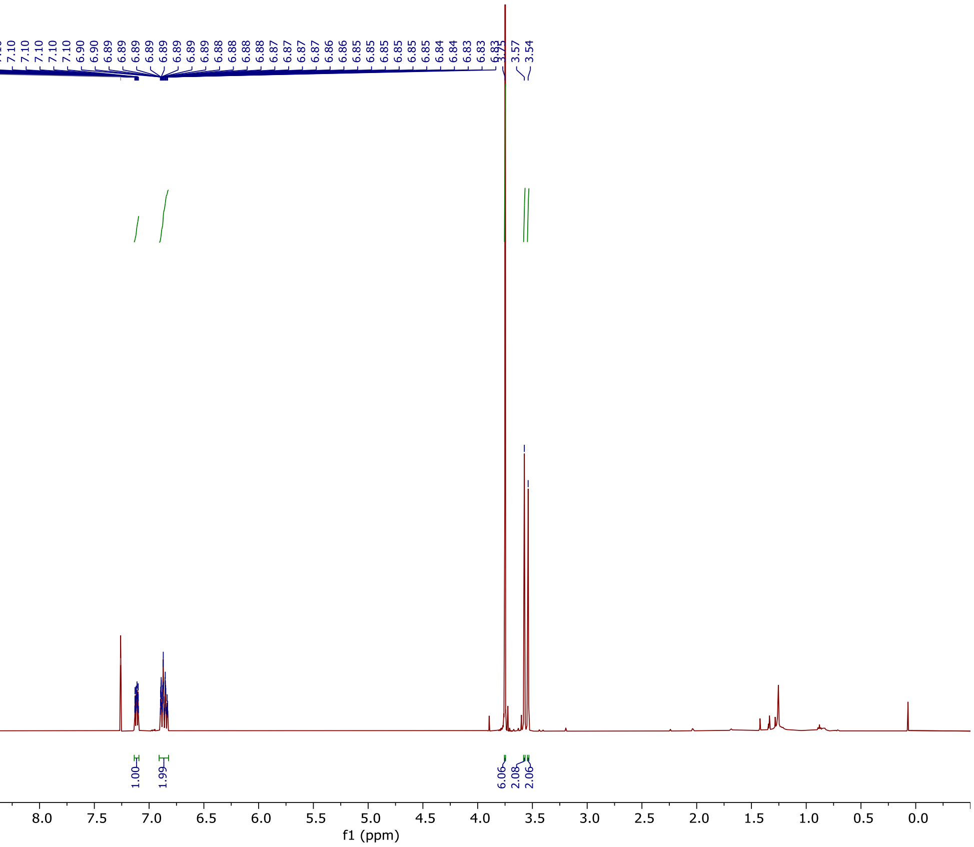

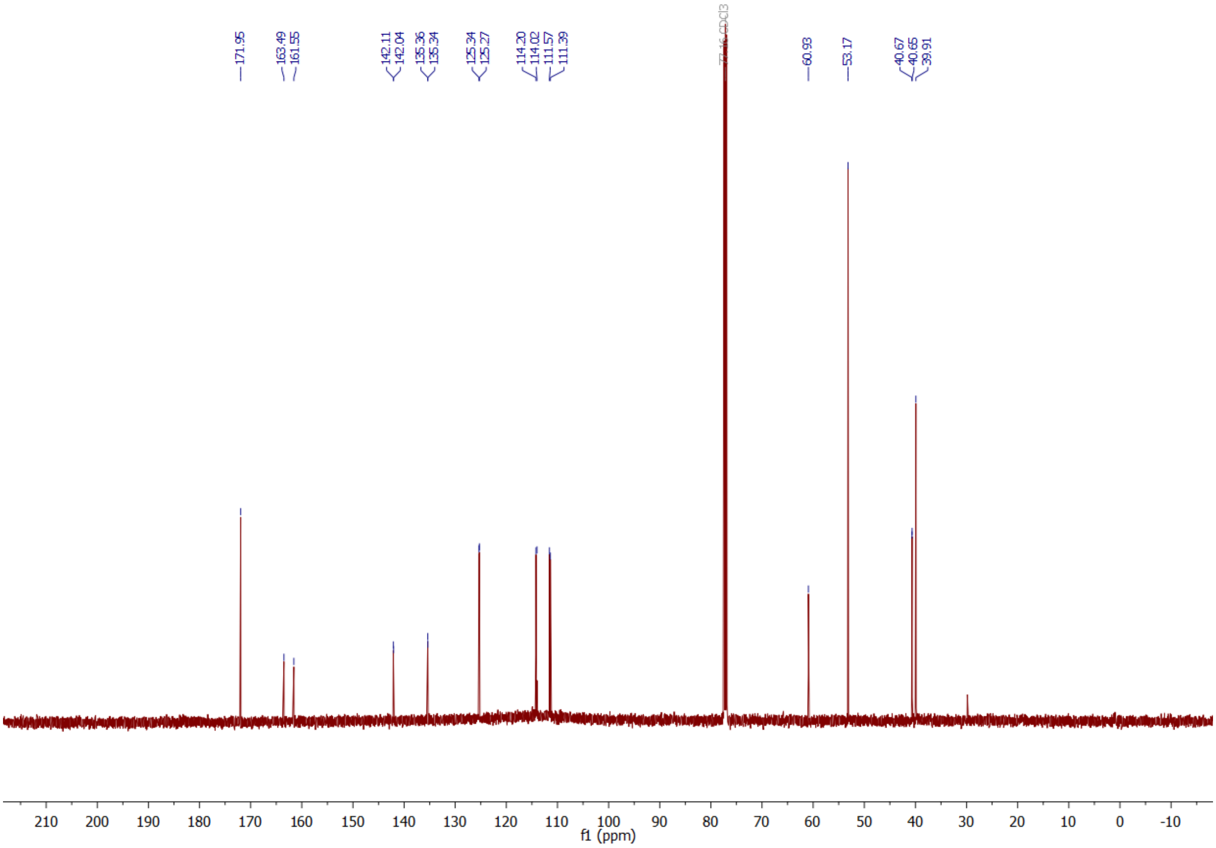

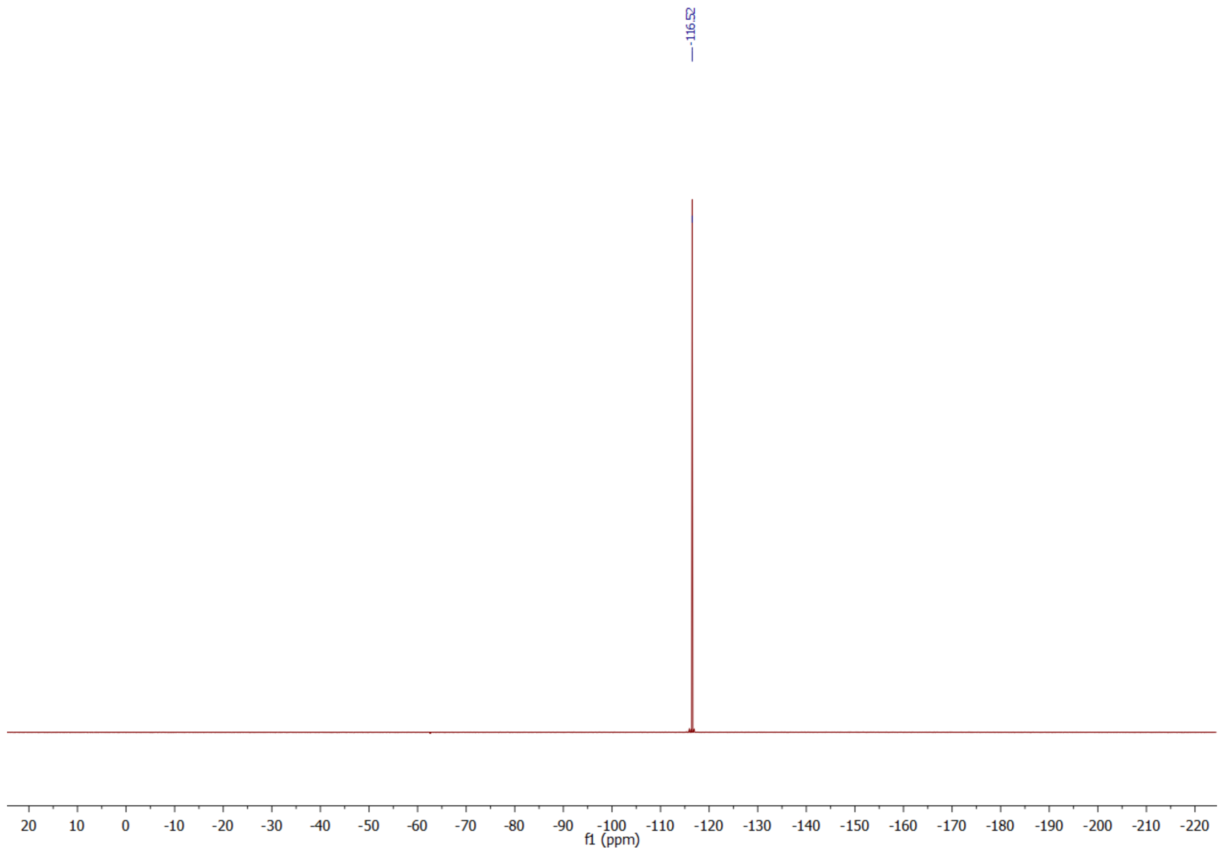

-116.52
20
10
0
-10
-20
-30
-40
-50
-60
-70
-80
-90
-100
-110
-120
-130
-140
-150
-160
-170
-180
-190
-200
-210
-220
f1 (ppm)

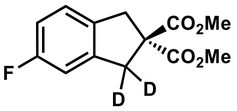

9b-*d2*

$C_{13}H_{11}D_2FO_4$

M= 254.25 g.mol-1

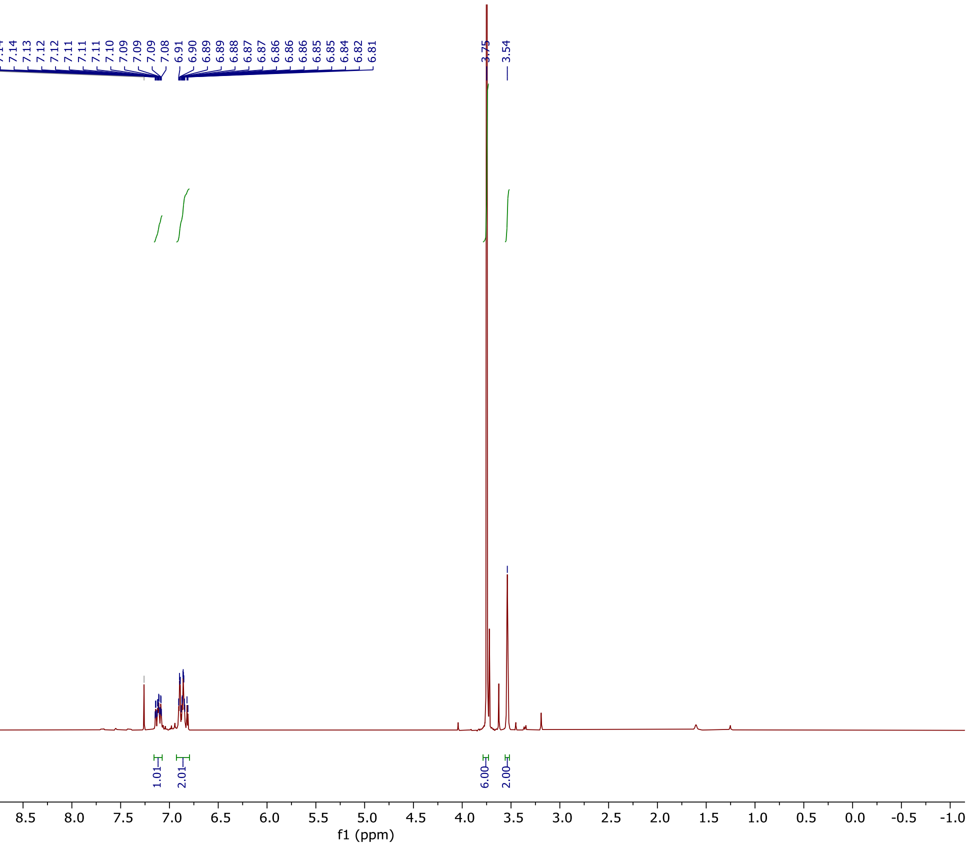

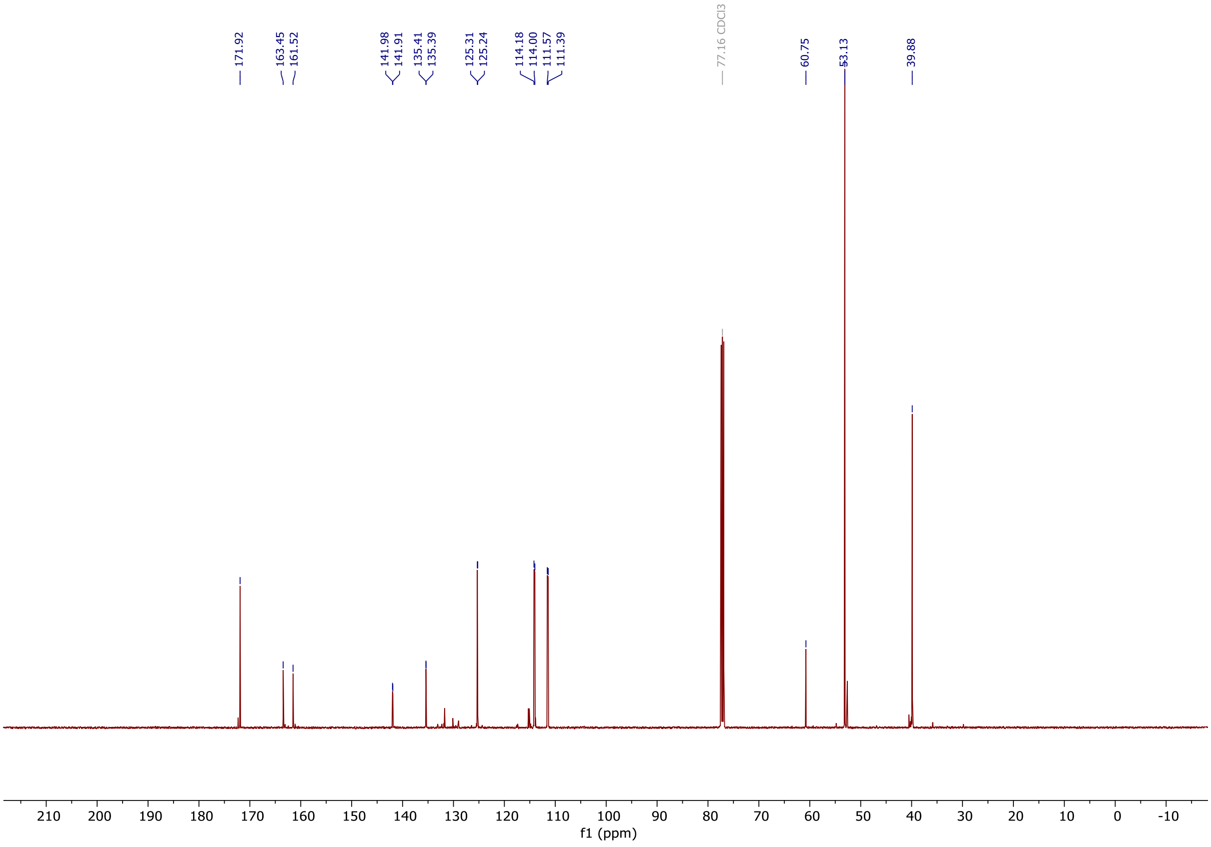

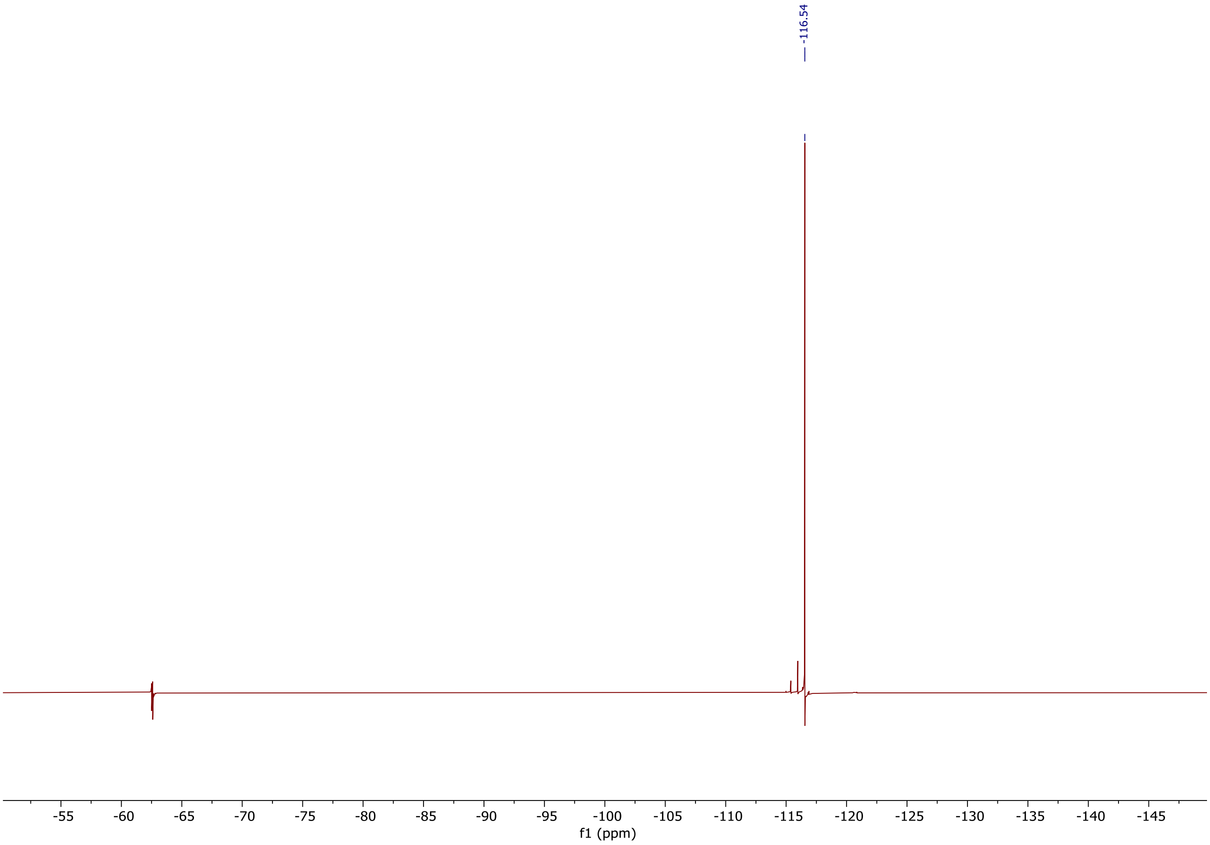
-116.54
-55
-60
-65
-70
-75
-80
-85
-90
-95
-100
-105
-110
-115
-120
-125
-130
-135
-140
-145
f1 (ppm)

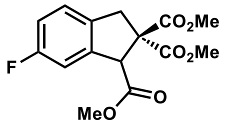

9c

$C_{15}H_{15}FO_6$

M= 310,28 g.mol-1

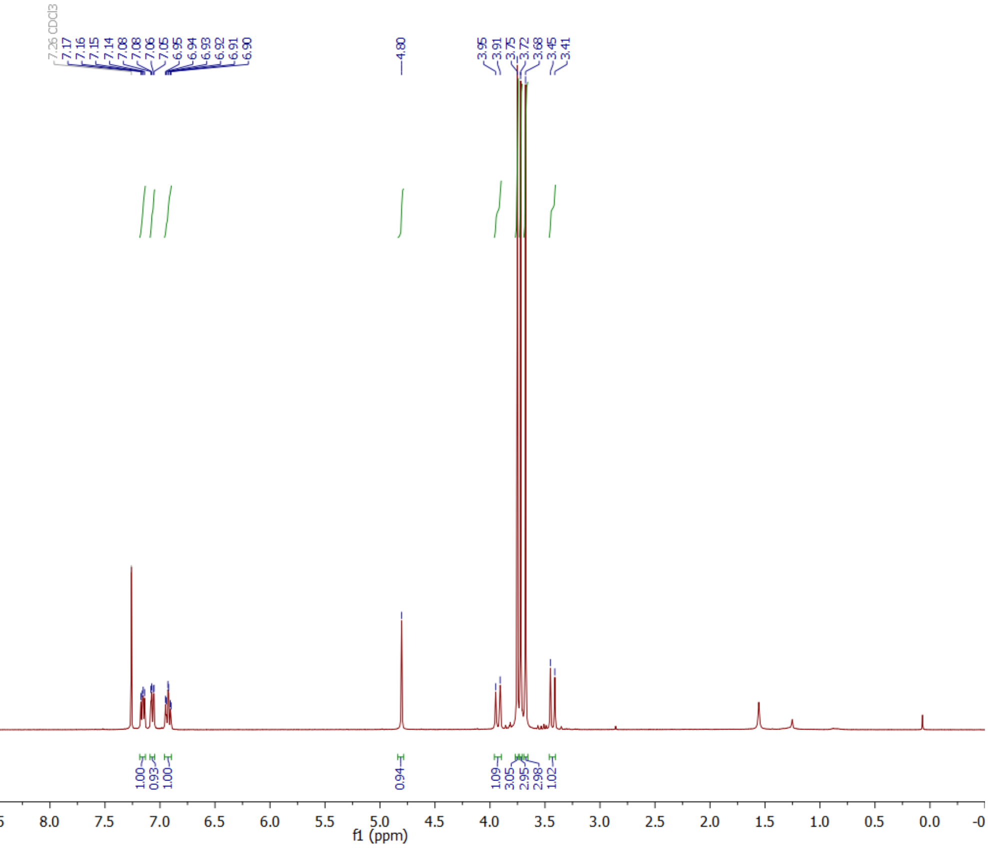

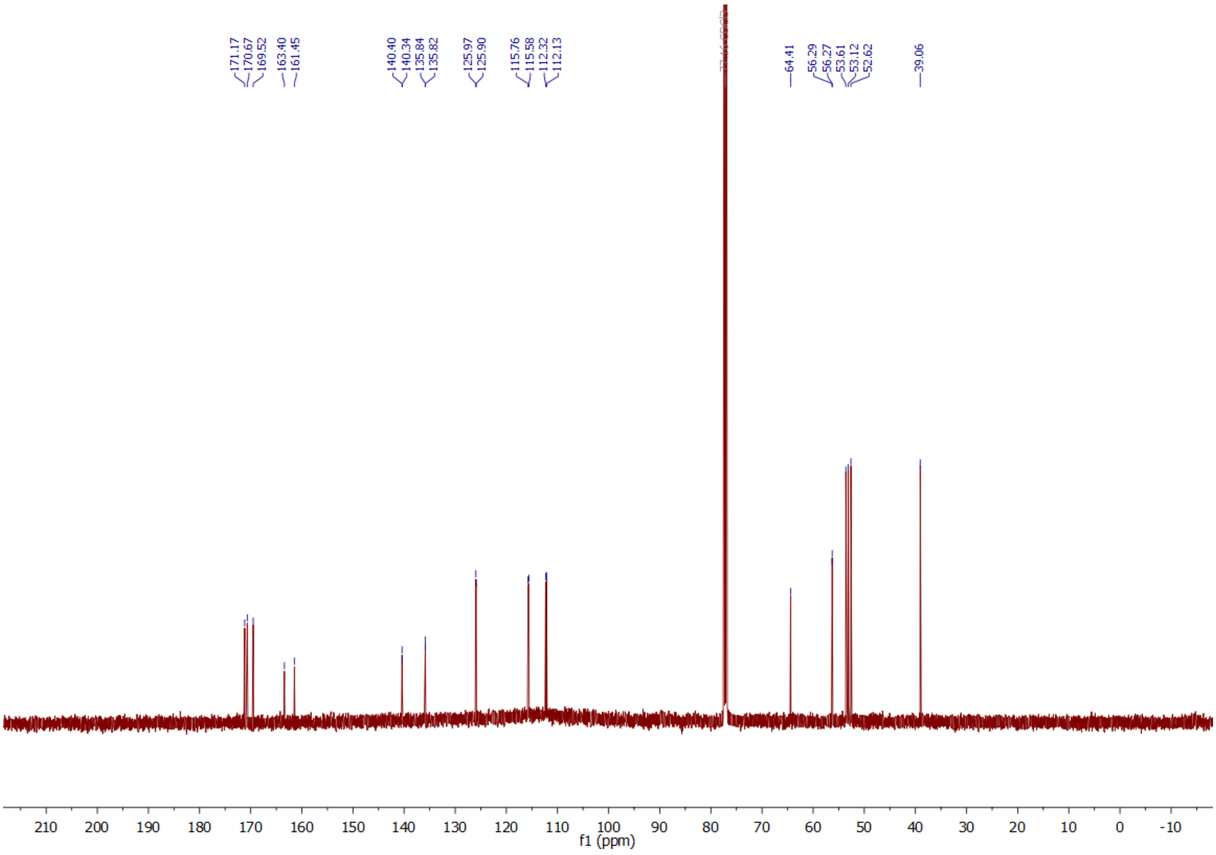

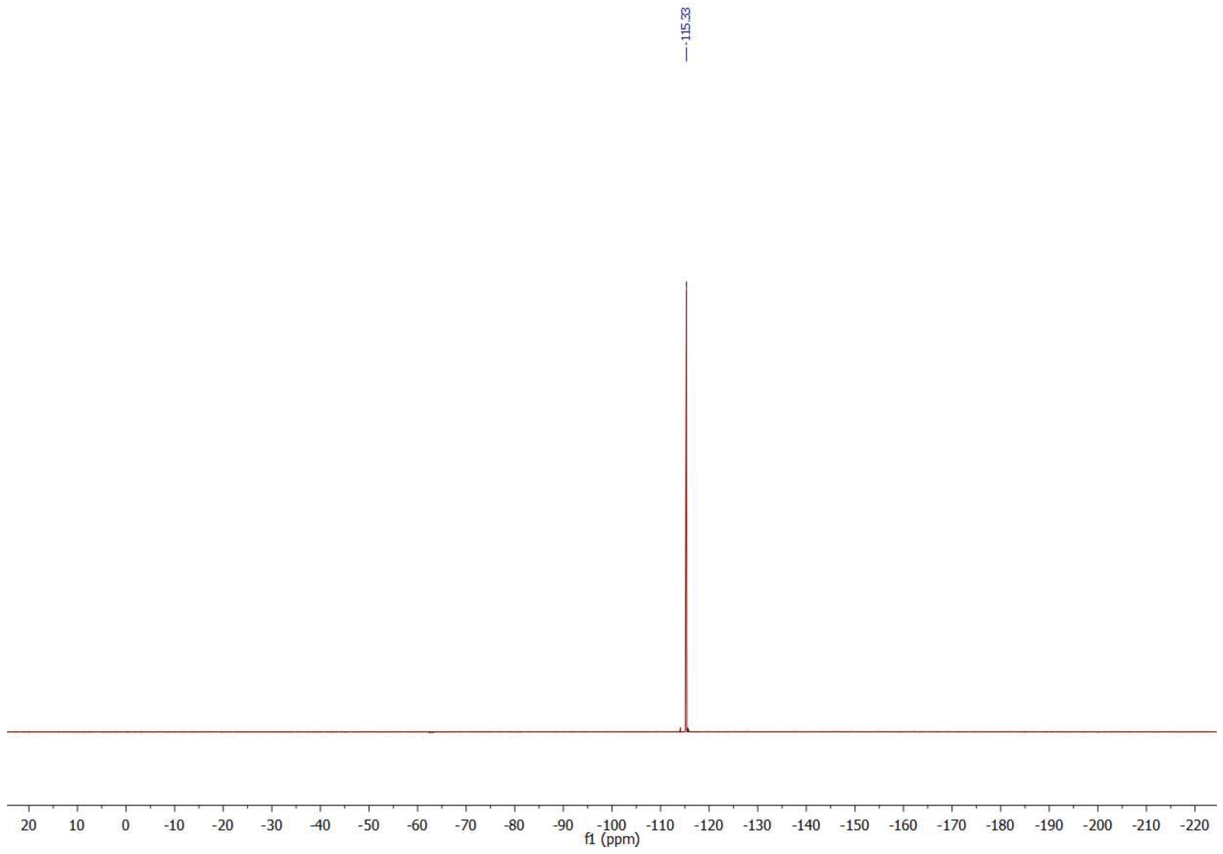

-115.33
20 10 0 -10 -20 -30 -40 -50 -60 -70 -80 -90 -100 -110 -120 -130 -140 -150 -160 -170 -180 -190 -200 -210 -220
f1 (ppm)

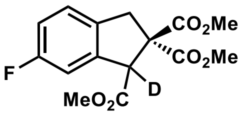

9c-*d1*
$C_{15}H_{14}DFO_6$
M= 311.28 g.mol-1

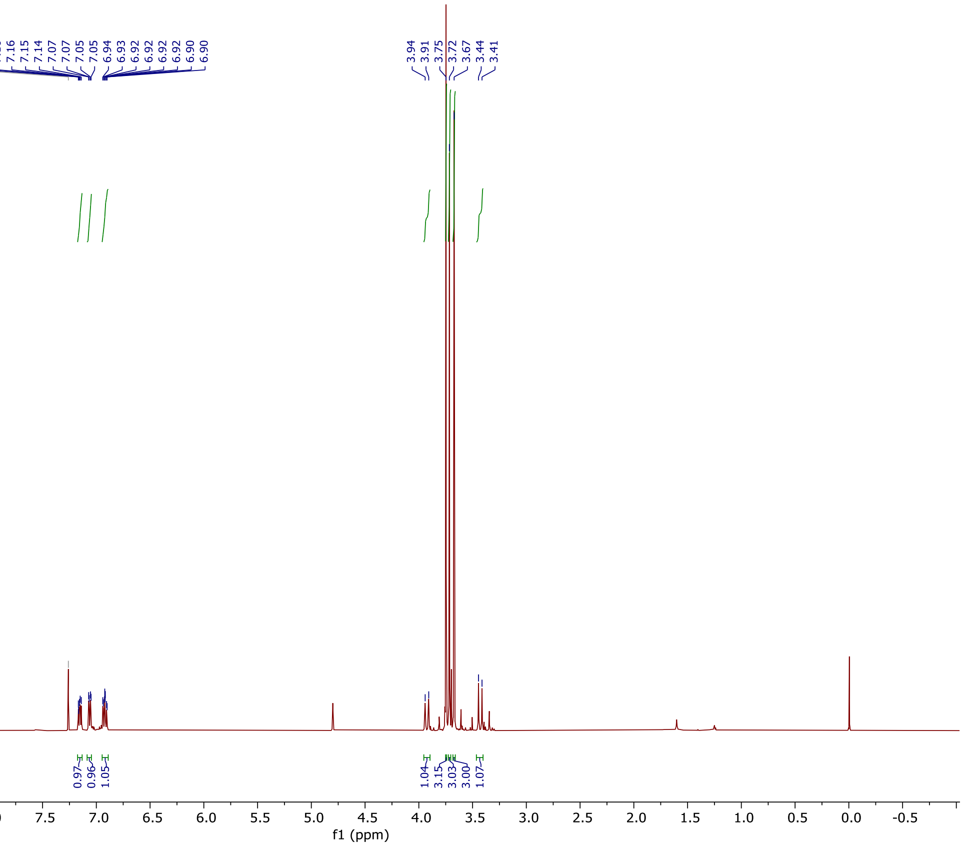

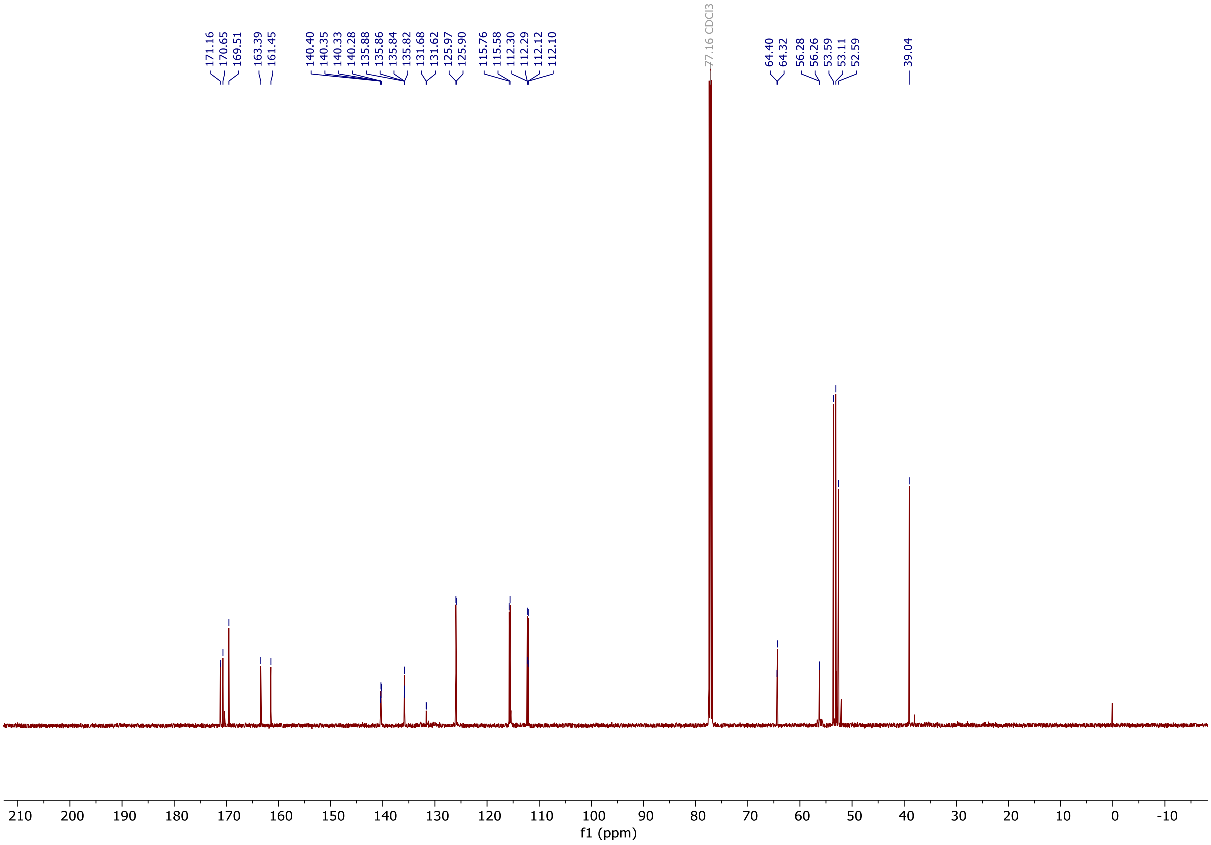

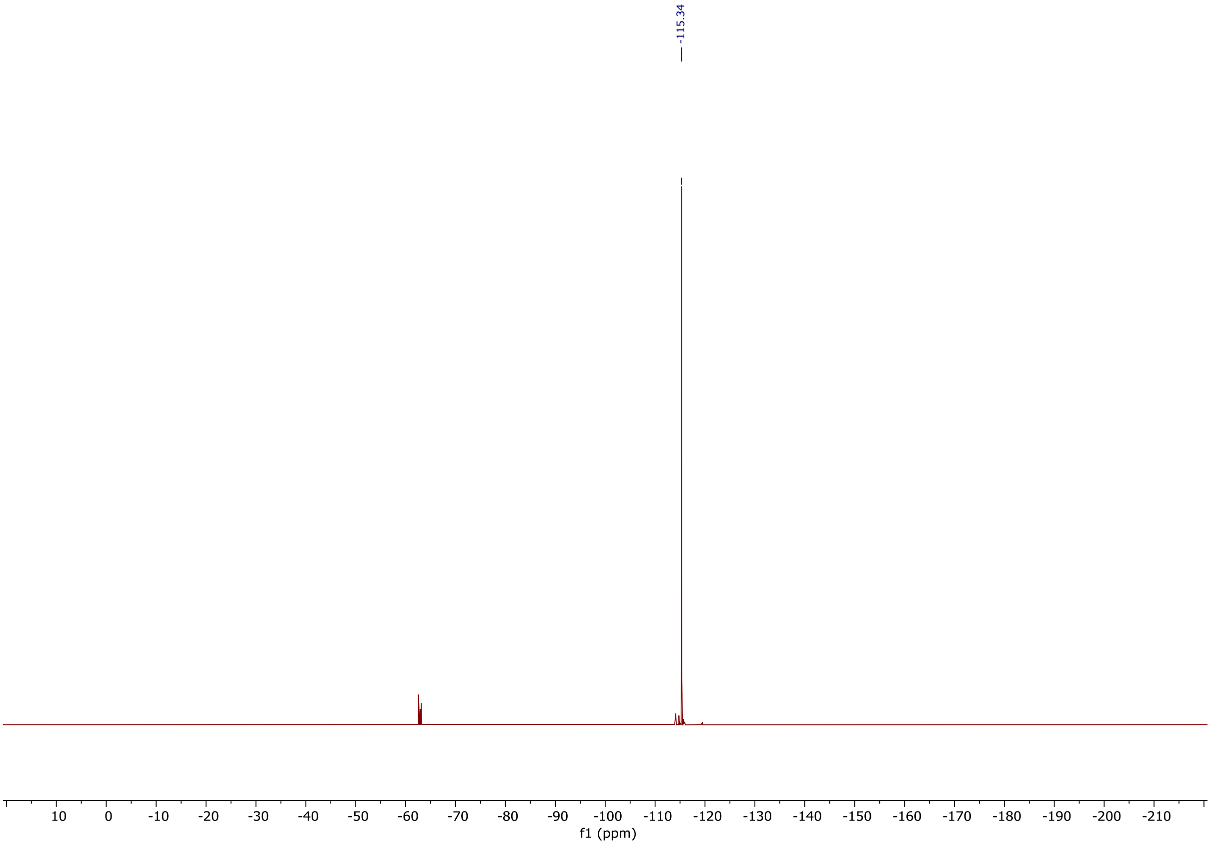
-115.34
10
0
-10
-20
-30
-40
-50
-60
-70
-80
-90
-100
-110
-120
-130
-140
-150
-160
-170
-180
-190
-200
-210
f1 (ppm)

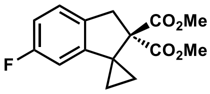

9d

$C_{15}H_{15}FO_4$

M= 278,28 g.mol-1

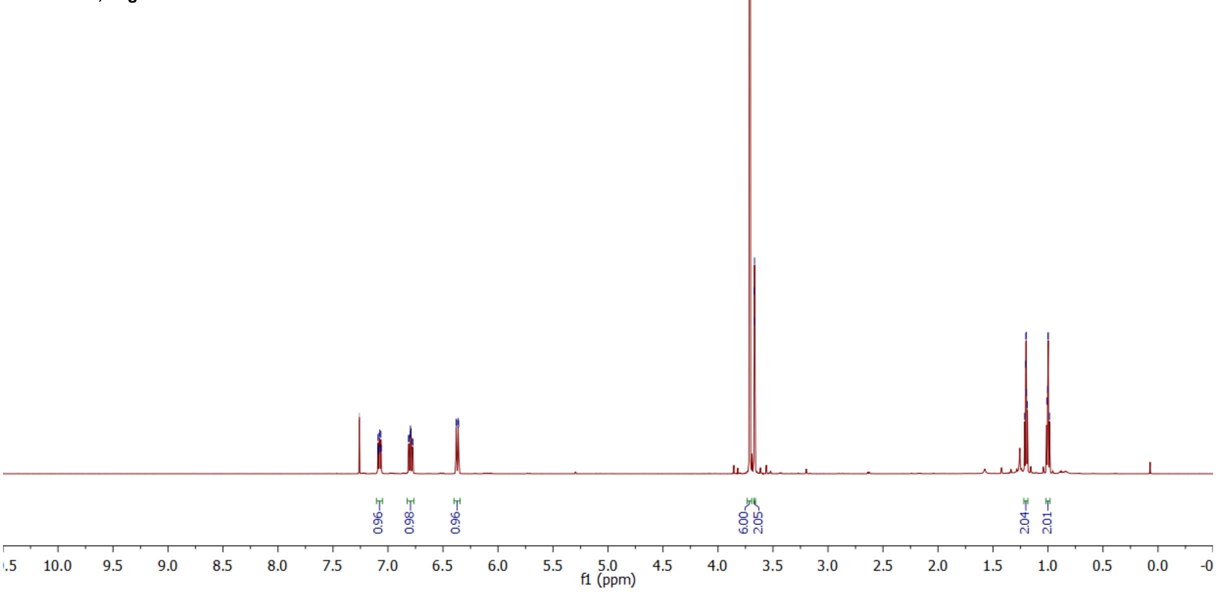

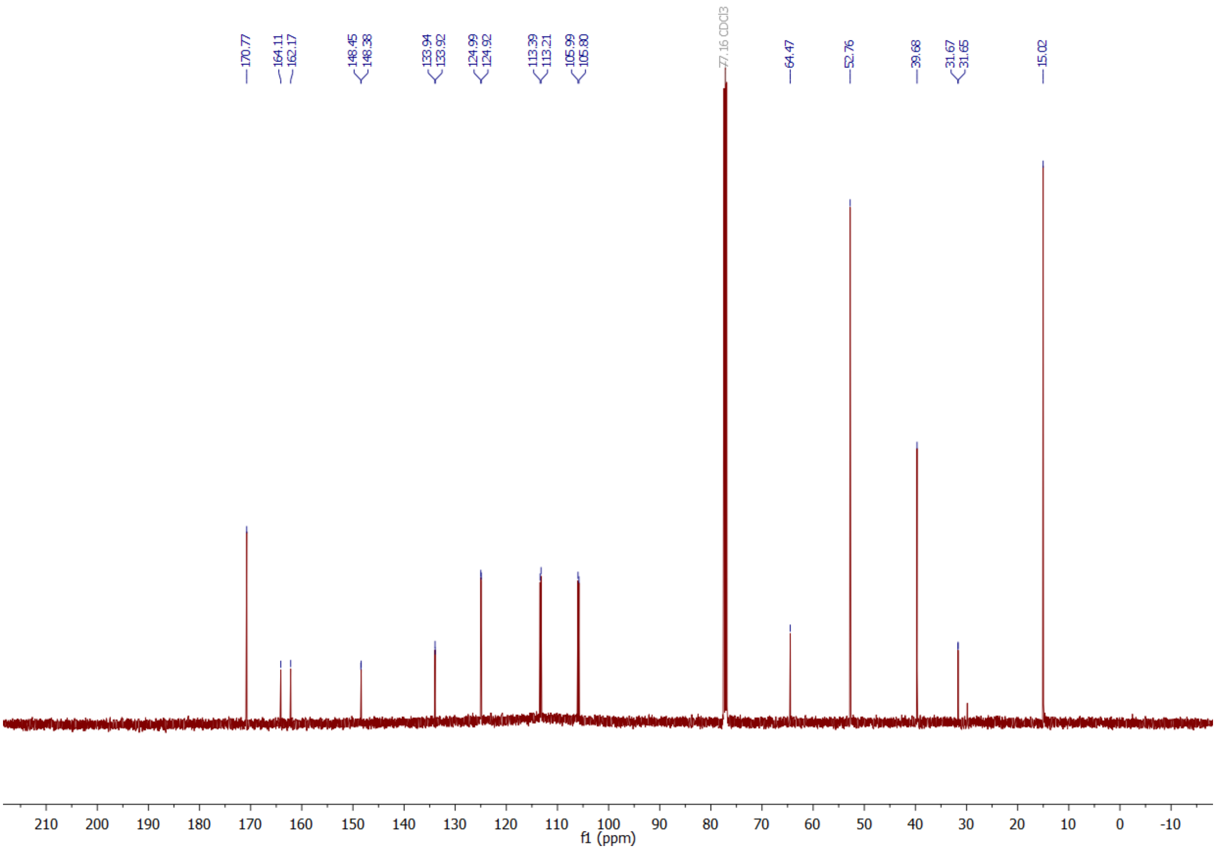

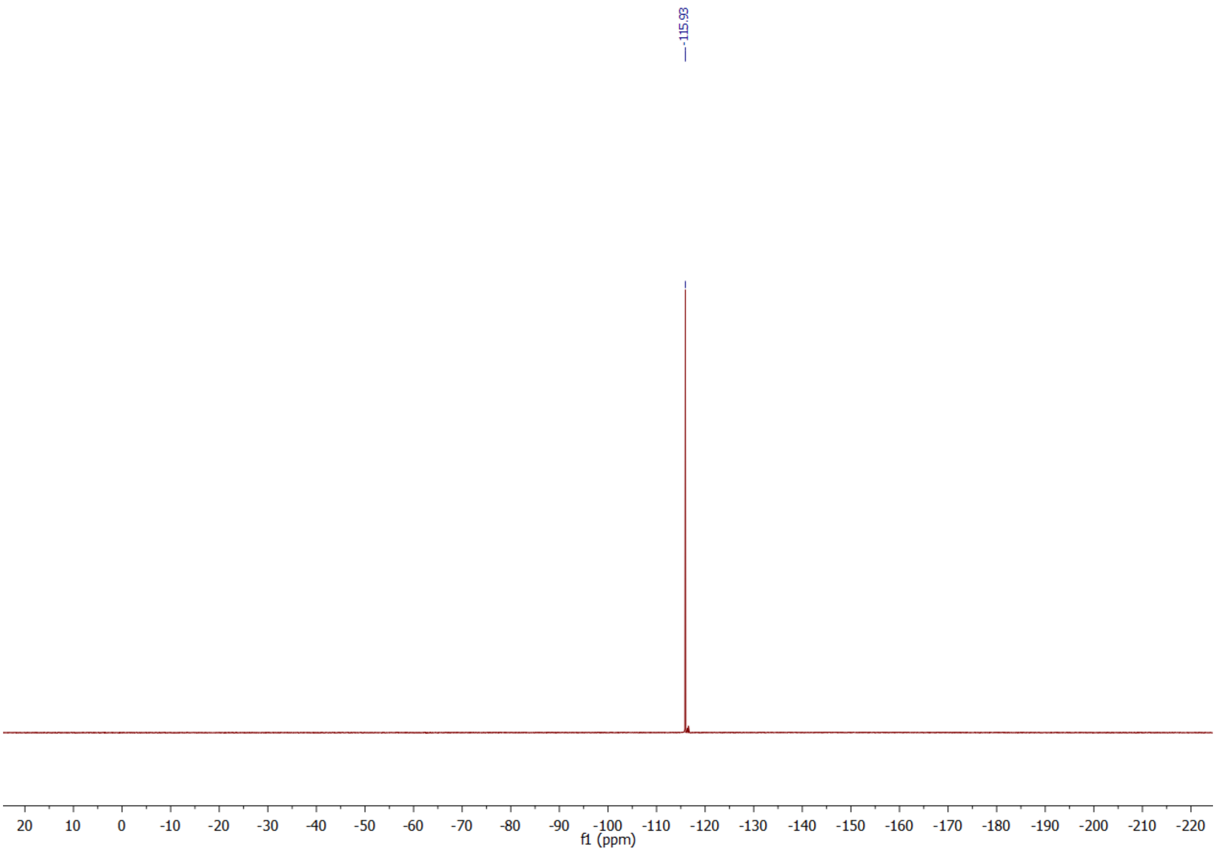
-115.93
20
10
0
-10
-20
-30
-40
-50
-60
-70
-80
-90
-100
-110
-120
-130
-140
-150
-160
-170
-180
-190
-200
-210
-220
f1 (ppm)

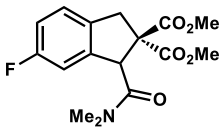

9e
$C_{16}H_{18}FNO_5$
M= 323,32 g.mol-1

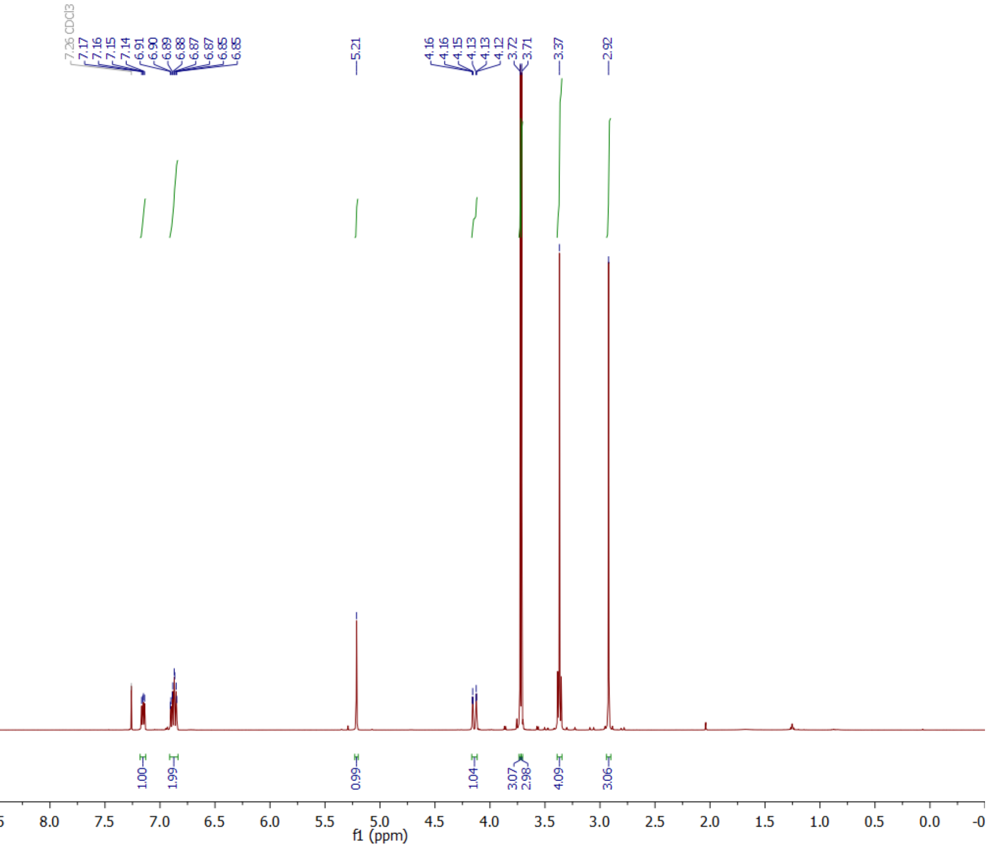

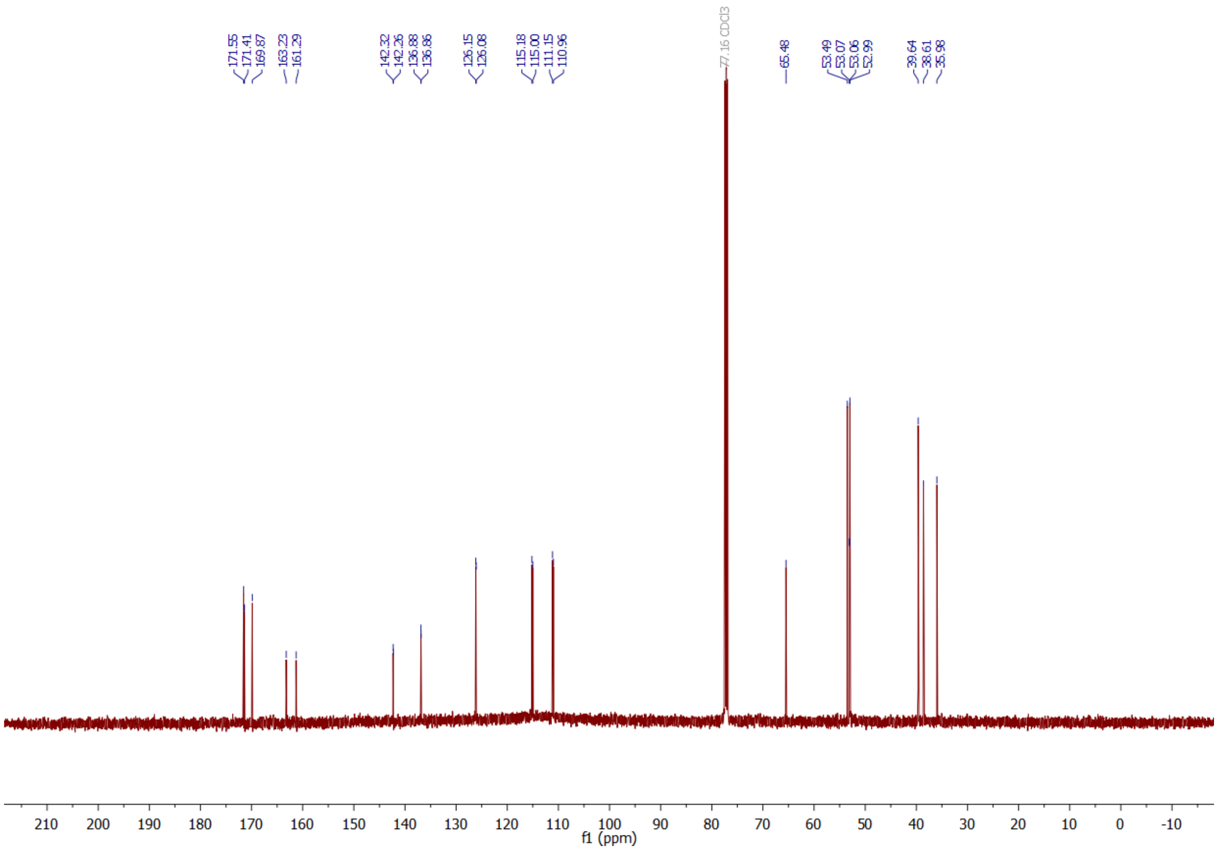

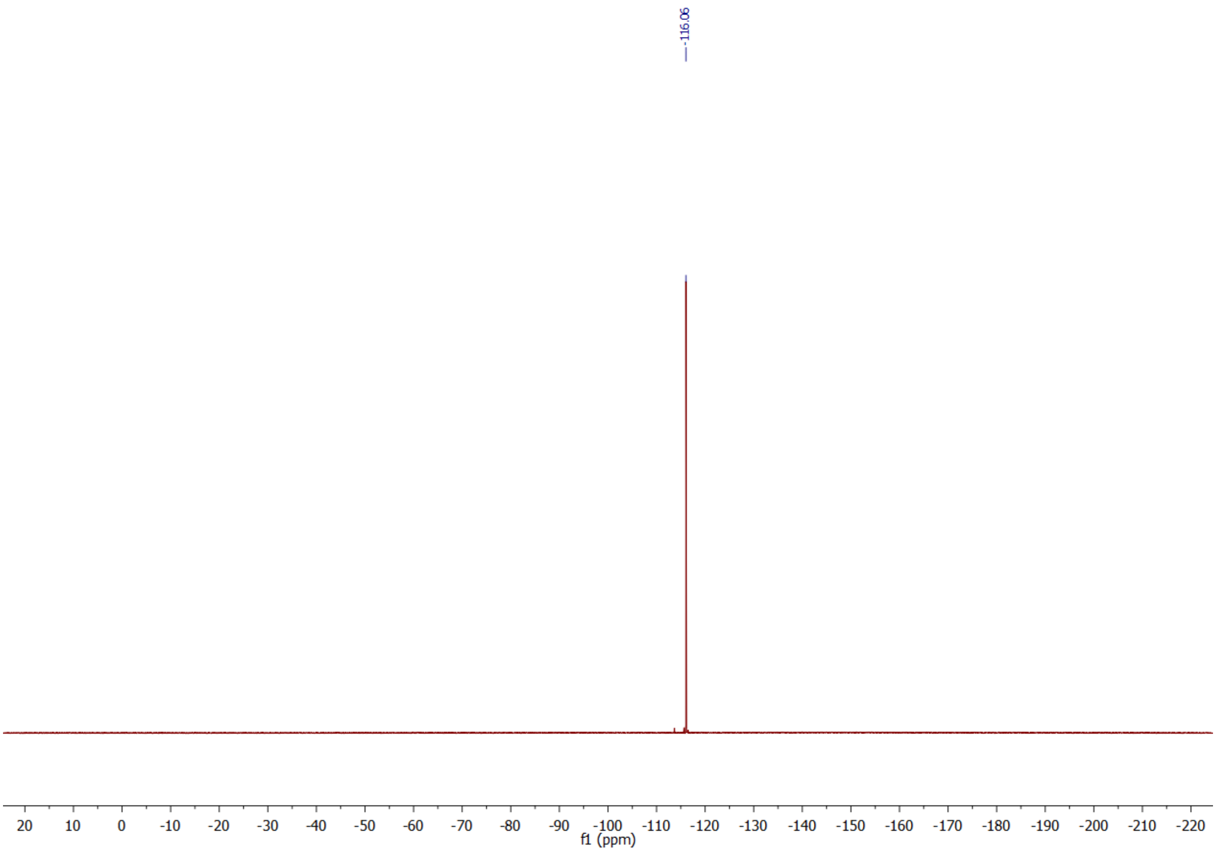
-116.06
20
10
0
-10
-20
-30
-40
-50
-60
-70
-80
-90
-100
-110
-120
-130
-140
-150
-160
-170
-180
-190
-200
-210
-220
f1 (ppm)

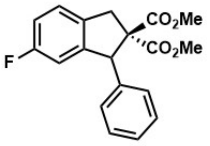

9f

$C_{19}H_{17}FO_5$

M= 328,34 g.mol-1

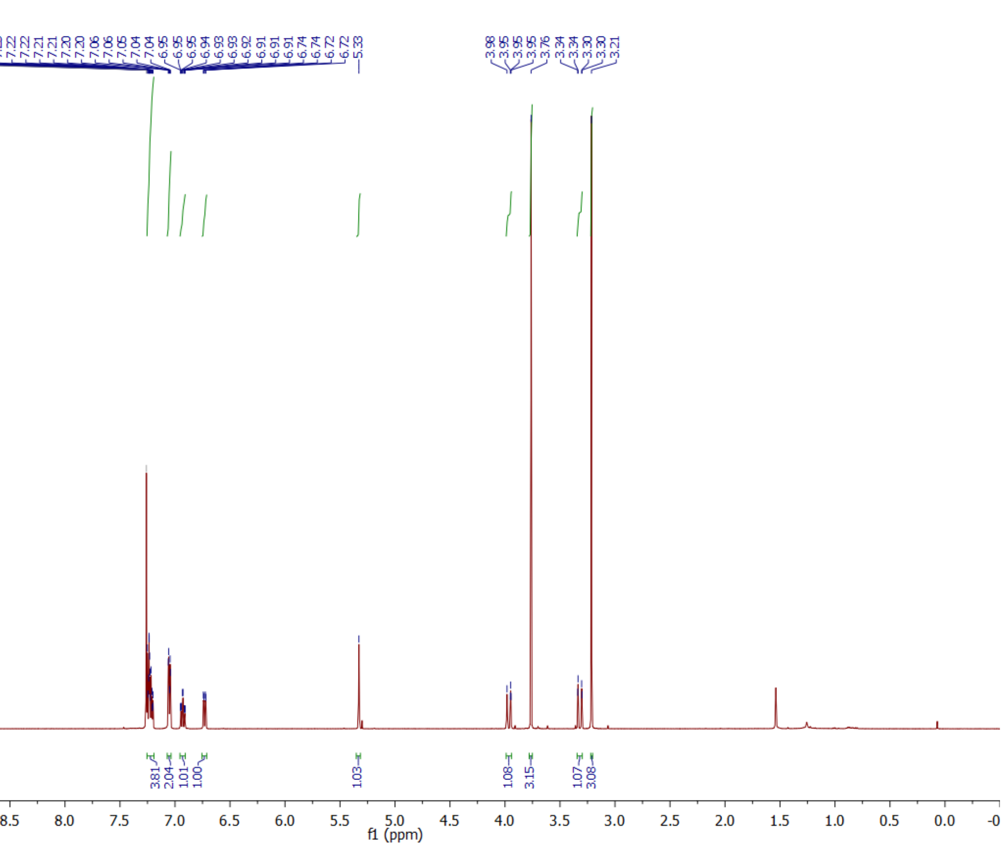

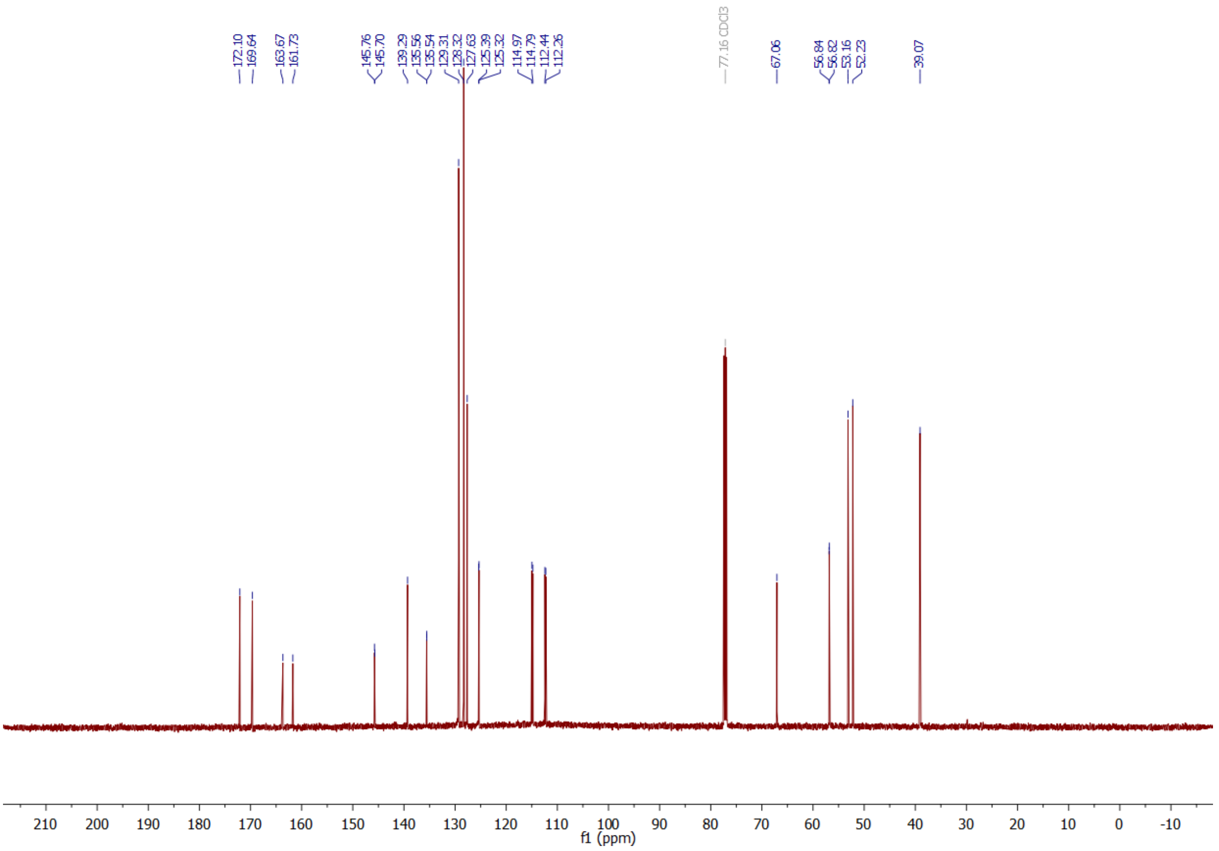

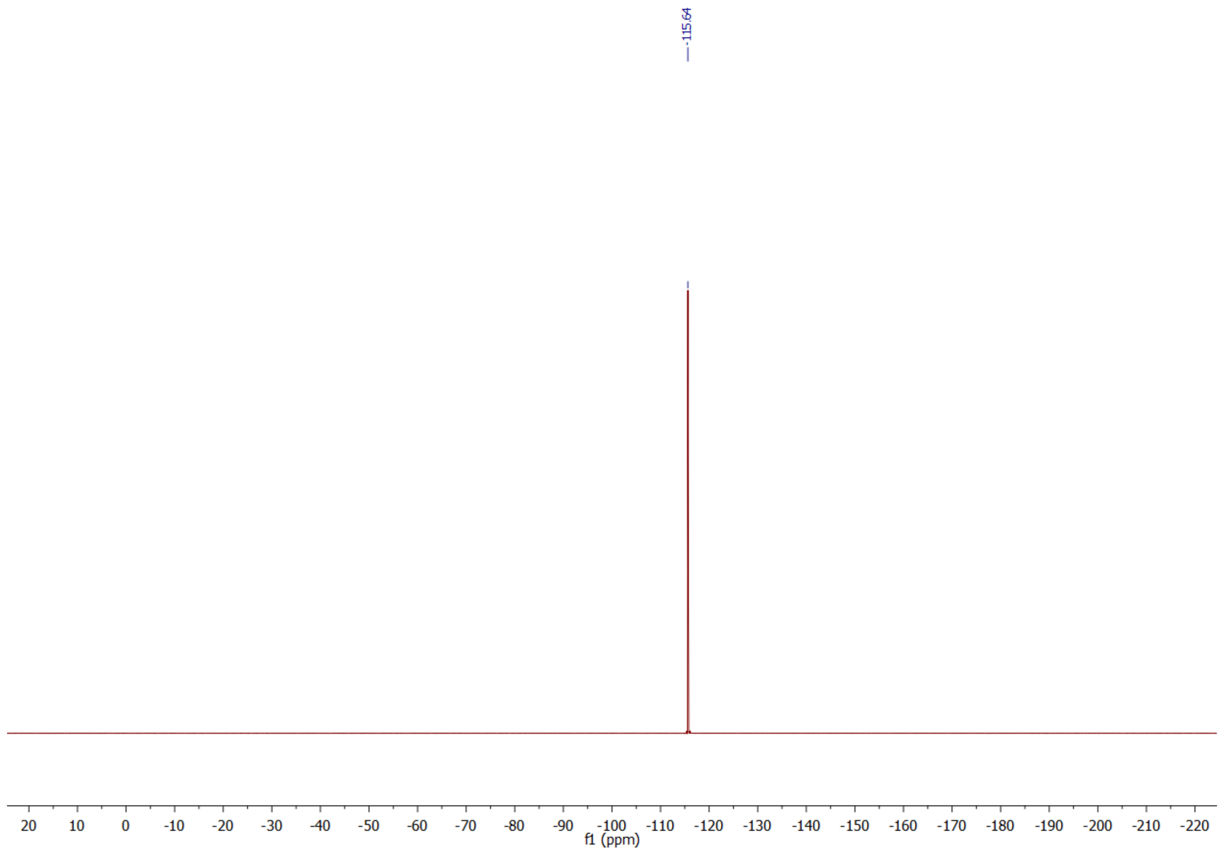
-115.64
20 10 0 -10 -20 -30 -40 -50 -60 -70 -80 -90 -100 -110 -120 -130 -140 -150 -160 -170 -180 -190 -200 -210 -220
f1 (ppm)

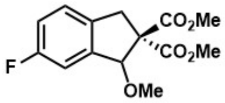

9g
$C_{14}H_{15}FO_5$
M= 282,27 g.mol-1

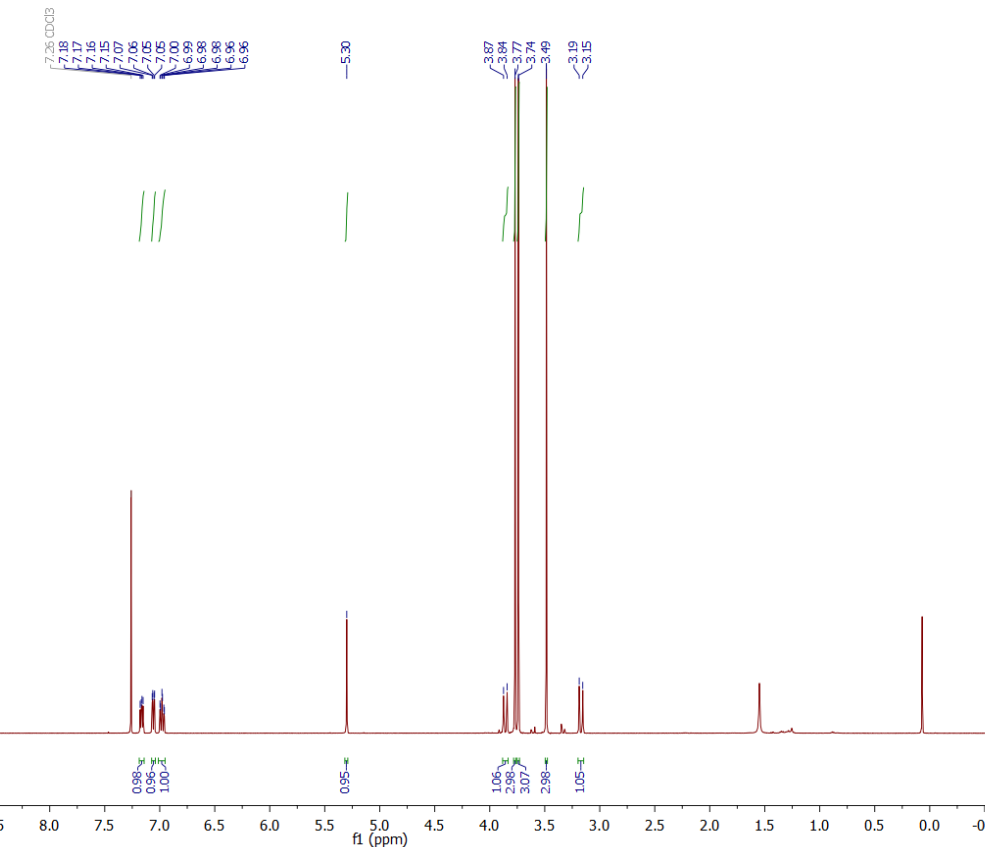

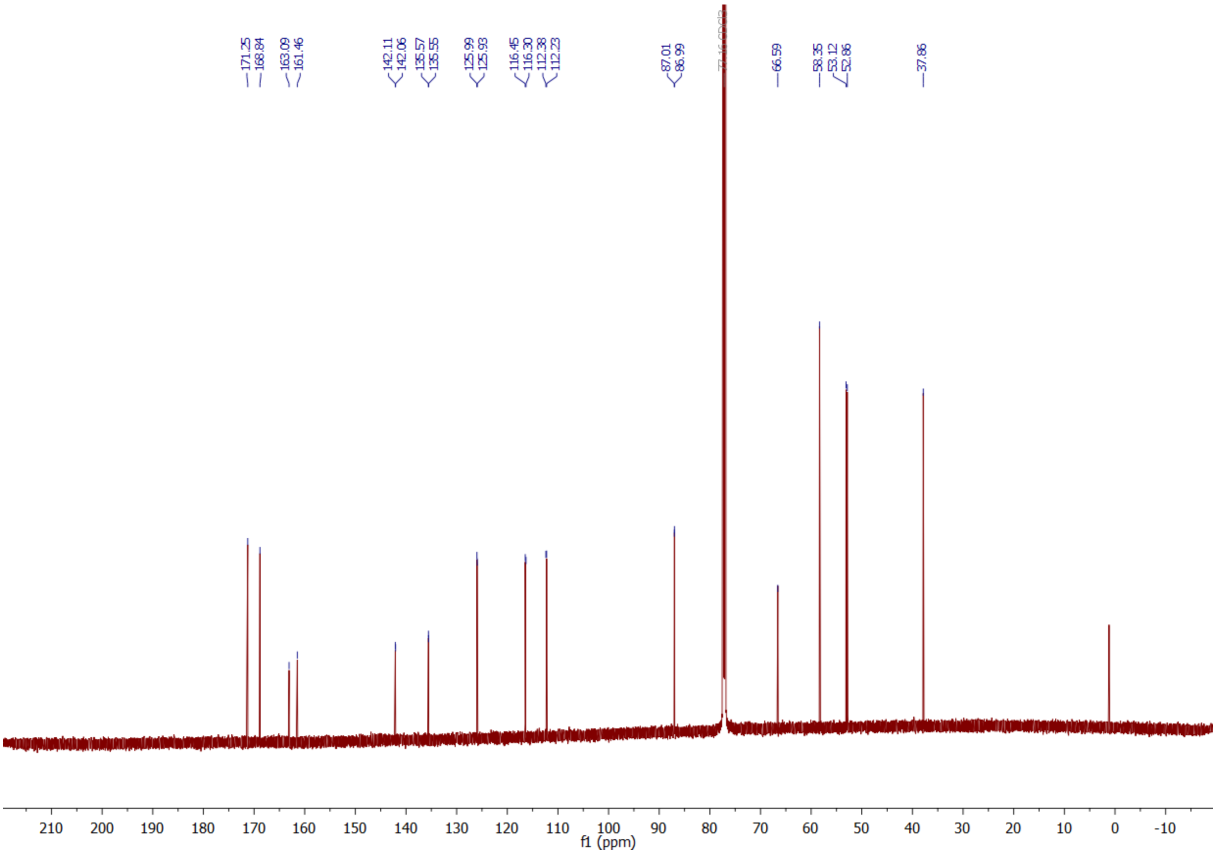

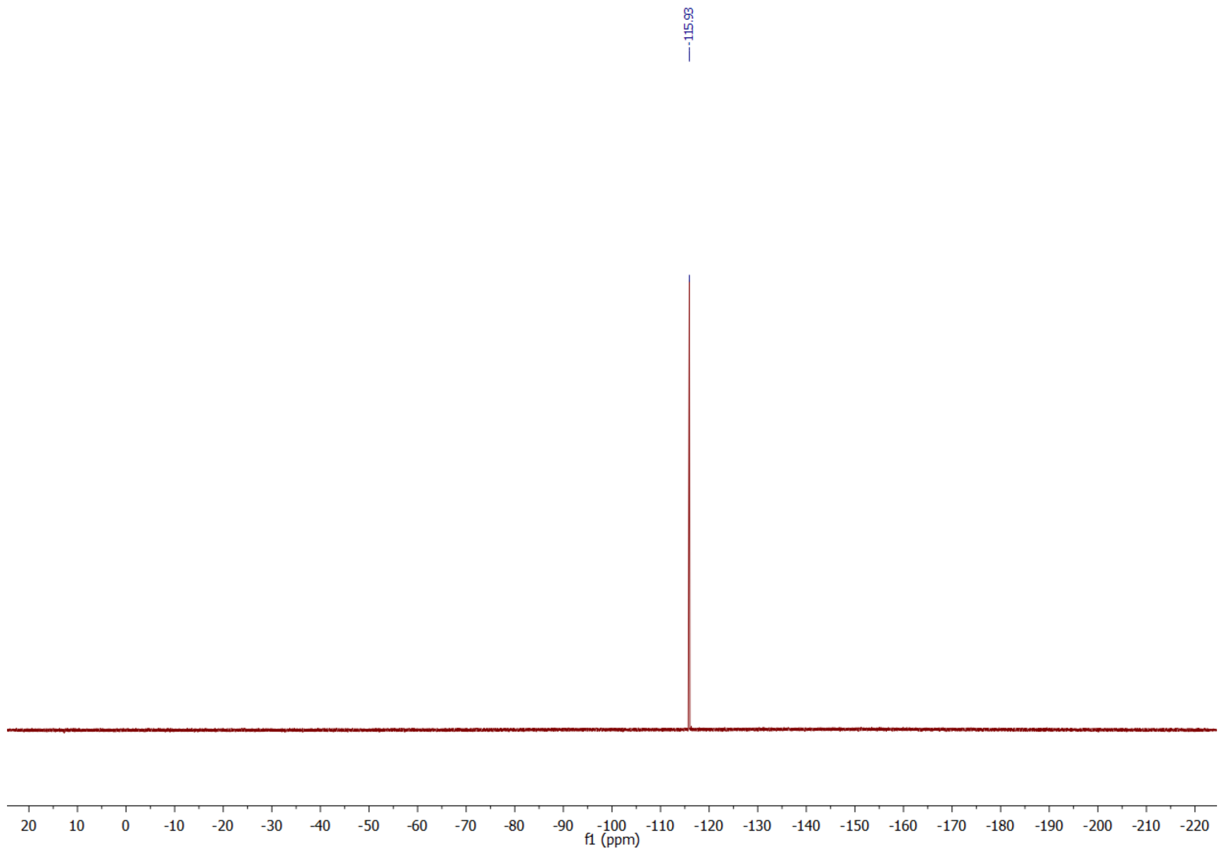
-115.93
20 10 0 -10 -20 -30 -40 -50 -60 -70 -80 -90 -100 -110 -120 -130 -140 -150 -160 -170 -180 -190 -200 -210 -220
f1 (ppm)

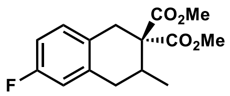

9'h

$C_{15}H_{17}FO_4$

M= 280.29 g.mol-1

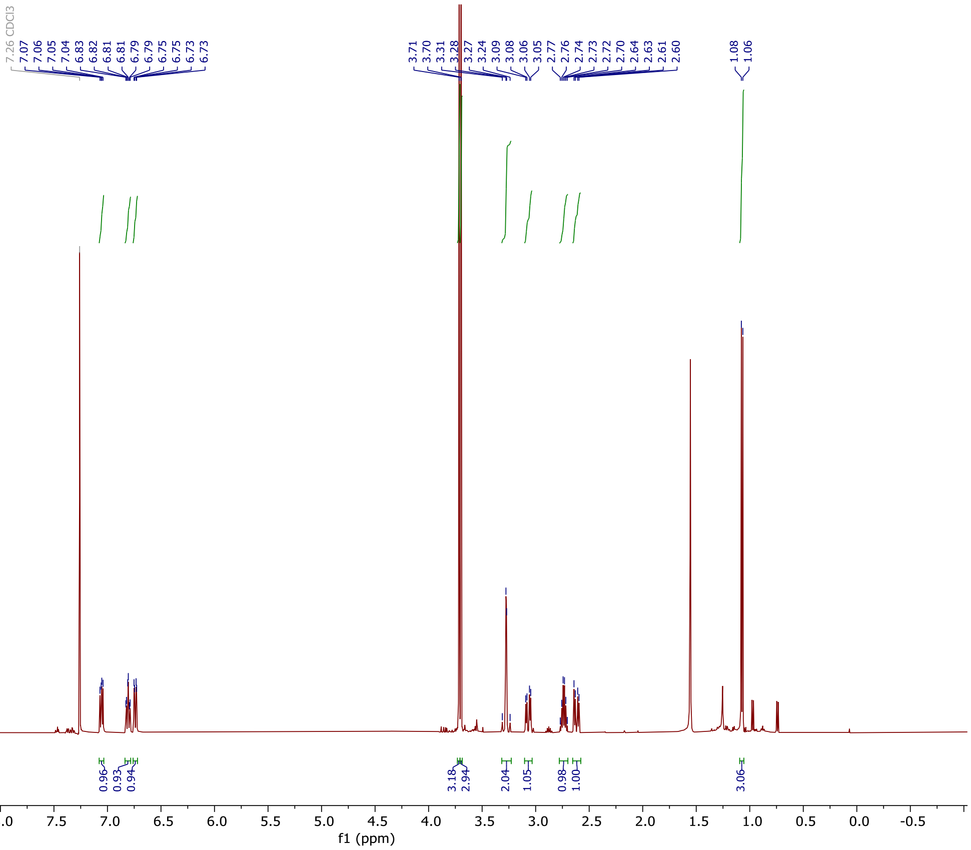

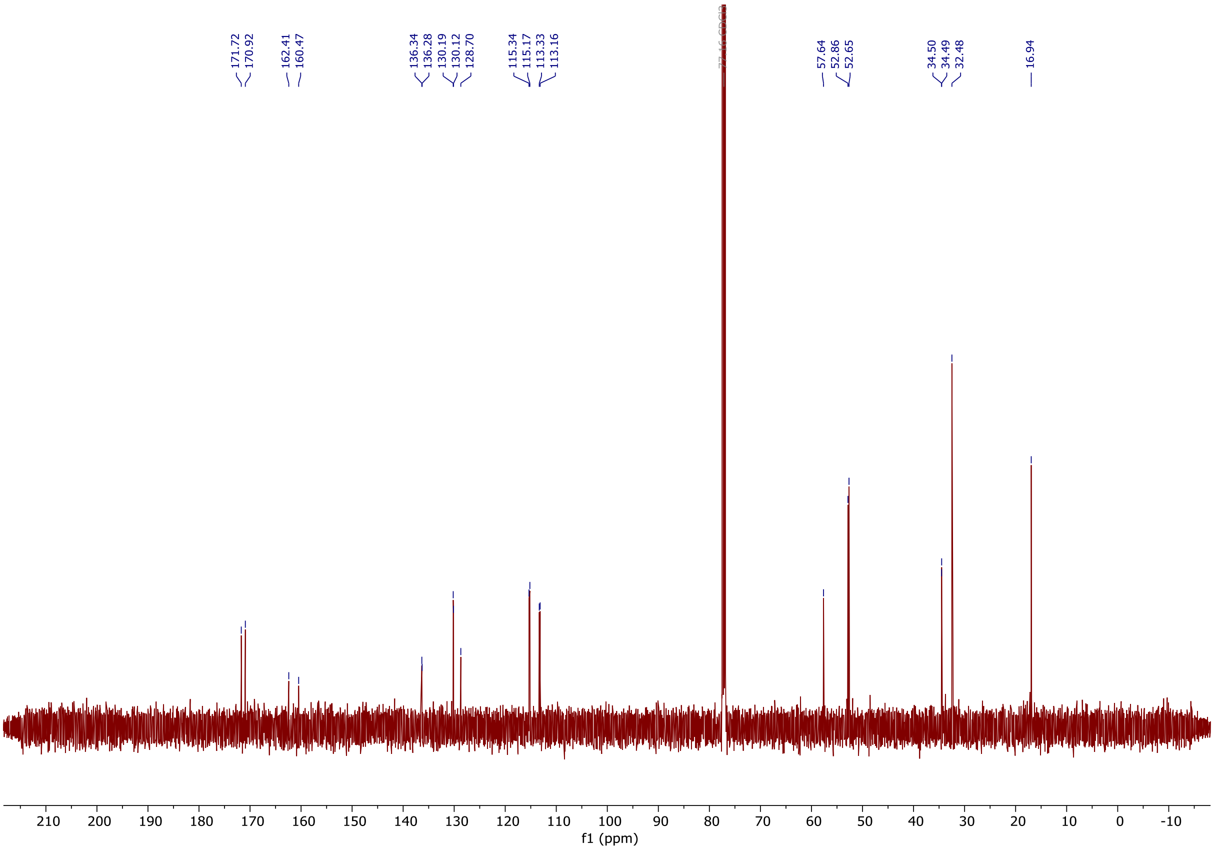

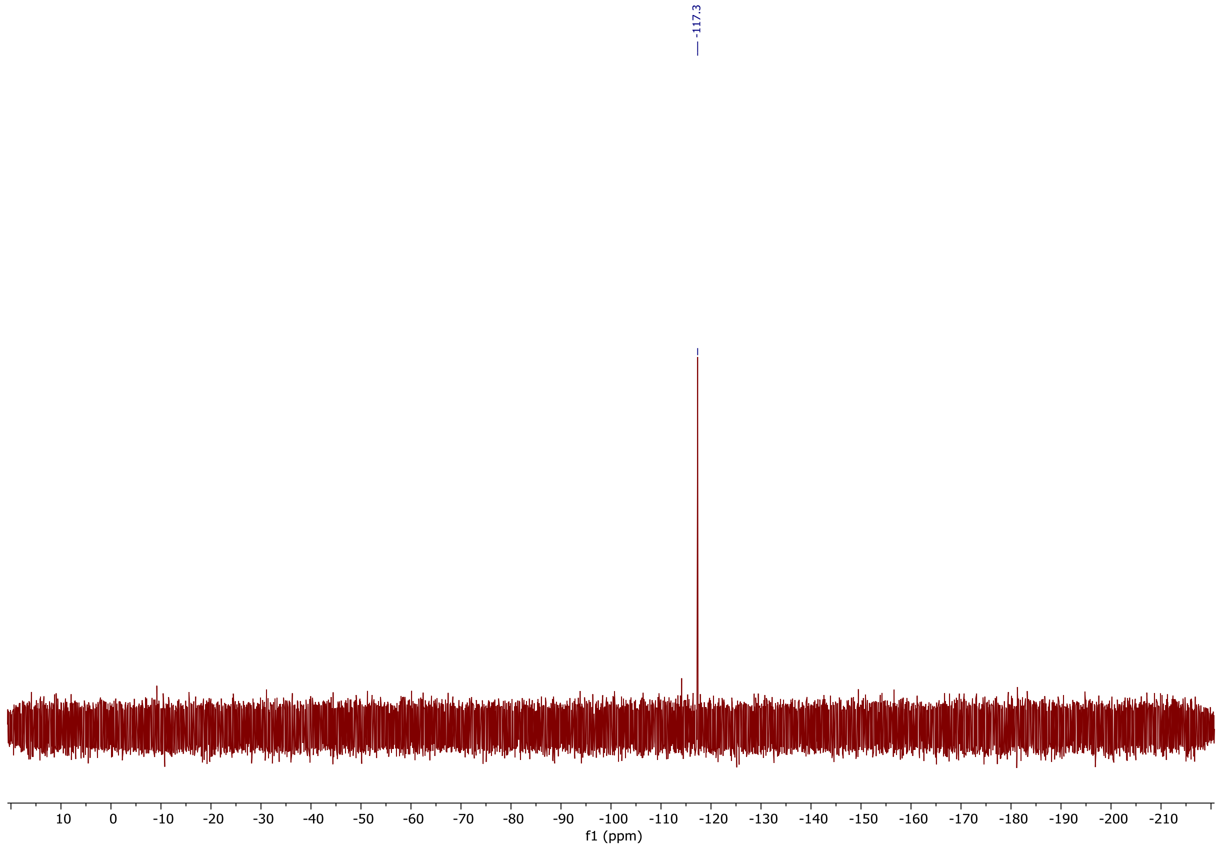
-117.3
10 0 -10 -20 -30 -40 -50 -60 -70 -80 -90 -100 -110 -120 -130 -140 -150 -160 -170 -180 -190 -200 -210
f1 (ppm)

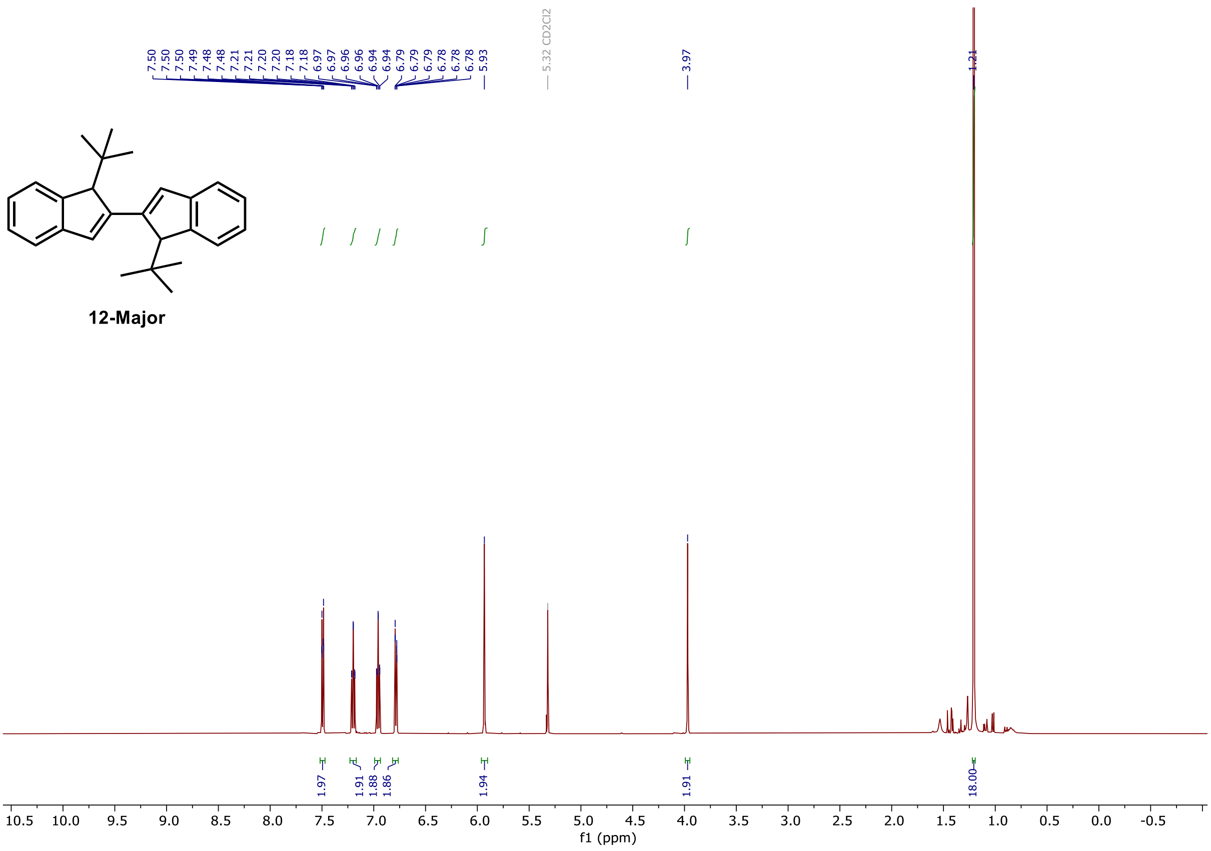

12-Major

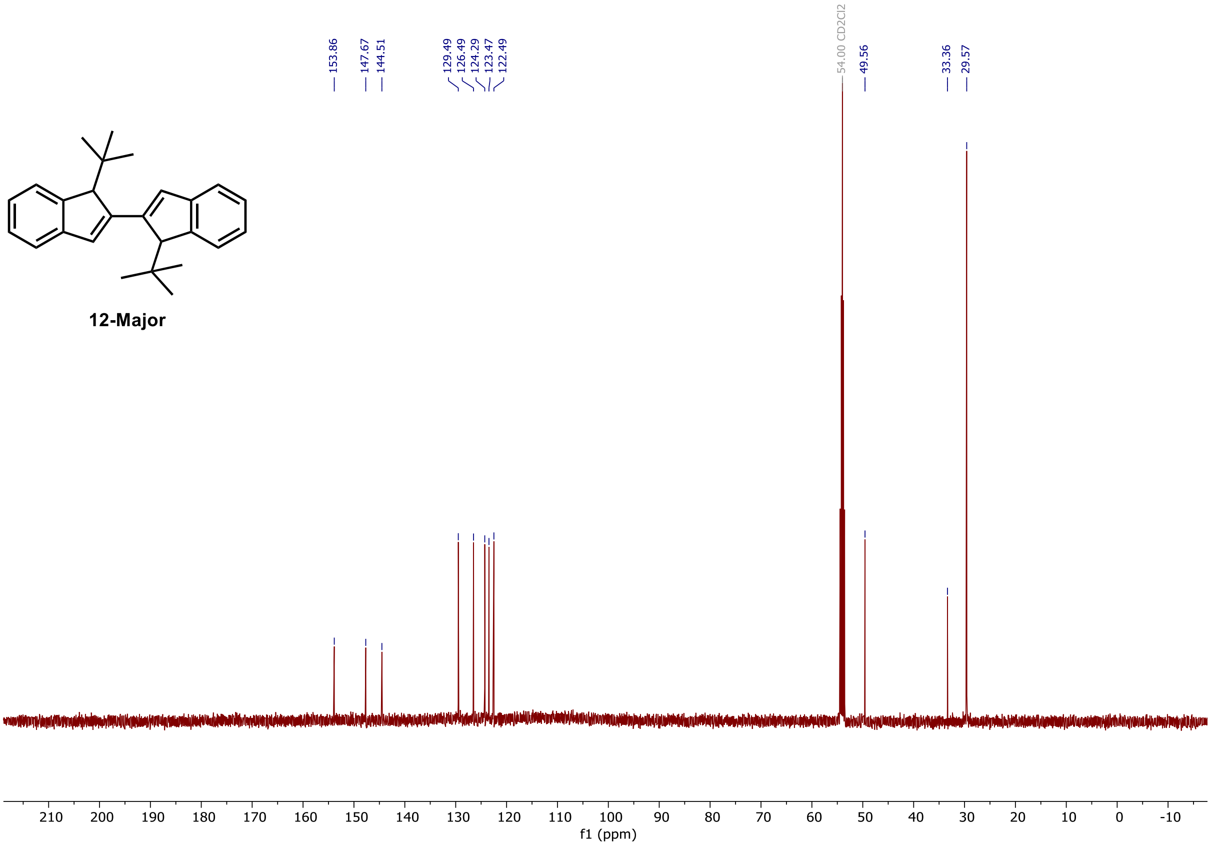

12-Major

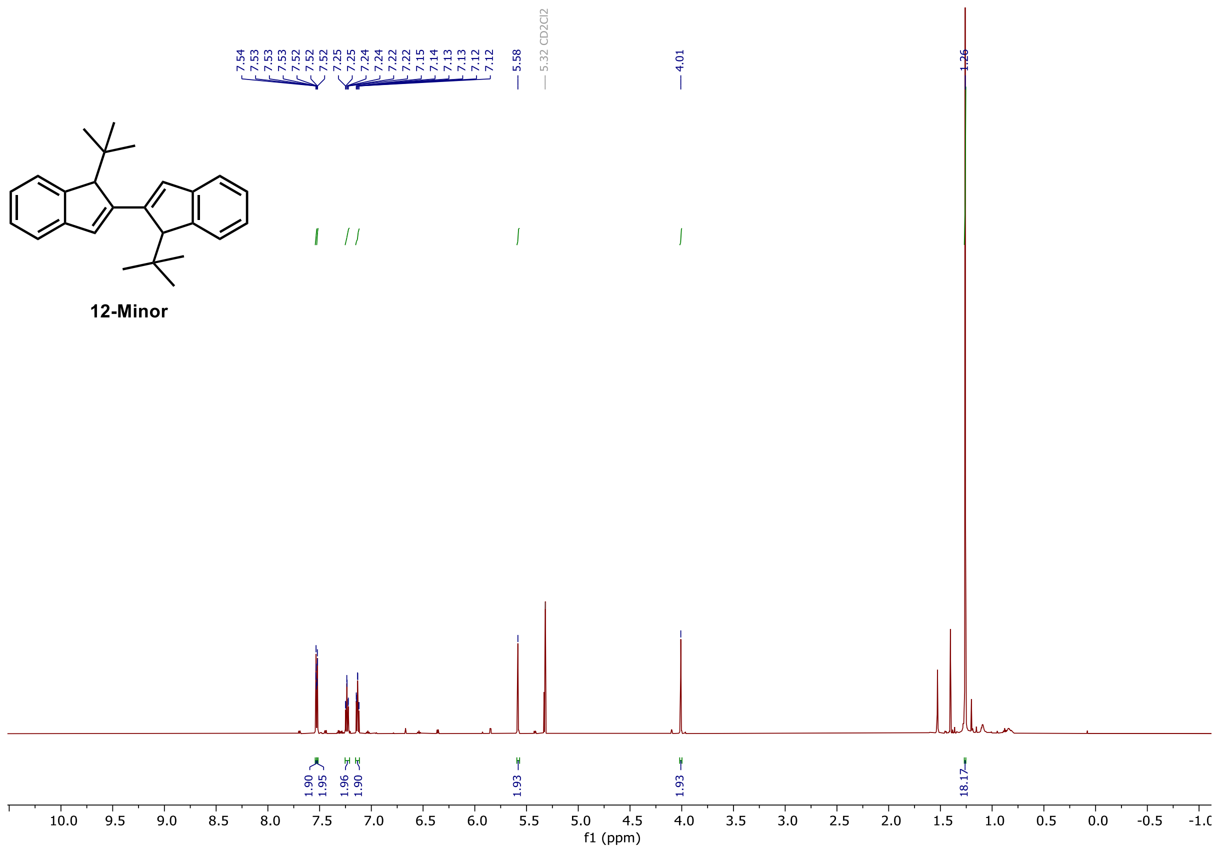
12-Minor
7.54 7.53 7.53 7.53 7.52 7.52 7.52 7.25 7.25 7.24 7.24 7.22 7.22 7.15 7.14 7.13 7.13 7.12 7.12
5.58
5.32 CD2Cl2
4.01
1.26
1.90 1.95 1.96 1.90 1.93 1.93 18.17
f1 (ppm)

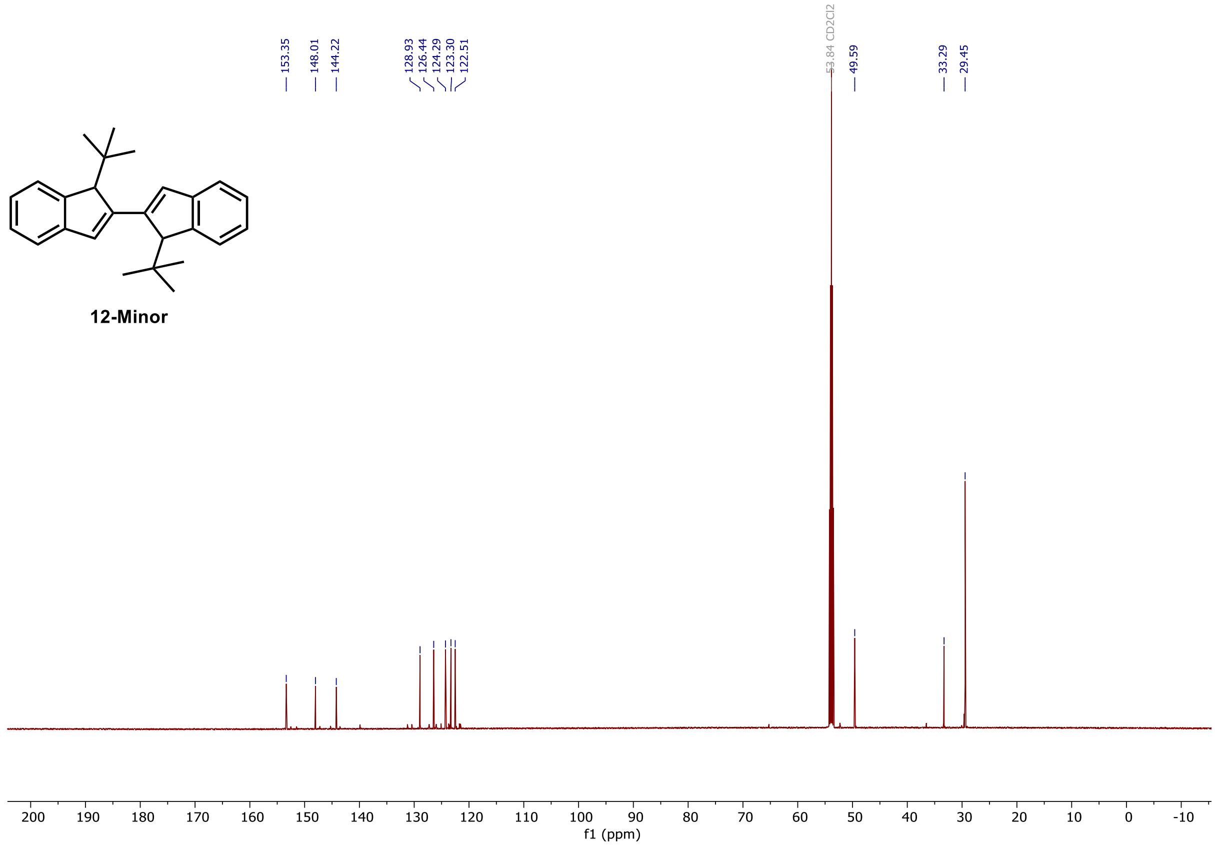
12-Minor
153.35 148.01 144.22
128.93 126.44 124.29 123.30 122.51
53.84 CD2Cl2
49.59
33.29 29.45
f1 (ppm)

8. Computational Details.

All geometry optimizations were run with Gaussian 16 (Revision A.03)[2] using the BP86 functional.[3,4] Pd and Br centers were described with the Stuttgart RECPs and associated basis sets[5] and 6-31G** basis sets were used for all other atoms.[6,7] A set of d-orbital polarization functions was also added to Br (ζ^d=0.428).[8] This basis set is termed BS1. Geometries of key stationary points (i.e. **III^R** and **TS(III^R-IV^R)2**) were subjected to conformational searches using our published protocol[9] and the most stable forms employed. Stationary points were characterized with analytical frequency calculations. Transition states (one negative frequency) were characterized via IRC calculations and subsequent geometry optimizations to confirm the adjacent minima. Final free energies were computed using the triple-ζ basis set Def2-TZVP[10,11] (BS2) and include corrections for dispersion using the D3BJ method[12] and solvation with 1,2-$C_6H_4Cl_2$ (ε=9.99),[13] this being employed to mimic the dielectric constant of trifluorotoluene (ε = 9.19) as the latter is not available in Gaussian16. Free energies were corrected to reflect the reaction temperature of 413 K. NBO calculations used NBO 6.0[14] implemented within Gaussian 09 Revision D.01.[15] The overall barrier to C–H activation was tested with a range of GGA, hybrid-GGA and meta-GGA functionals (BLYP,[3,16,17] B3LYP,[18] PBE,[19] PBE0,[20] B97D3,[21,12] B97D,[21] M06,[22] ωB97x-D,[23] TPSS[24] and B3PW9[18,25]). Additional reaction profiles not shown in the main text are provided below, along with details of functional testing and changes in the chemical model adopted. All geometries are supplied as a separate XYZ file. Ball and stick models and NBO orbital diagrams were created with Chemcraft[26] and the latter used an isosurface value of 0.03 au.

9. Computed Results

9.1. Ar−Br Oxidative Addition

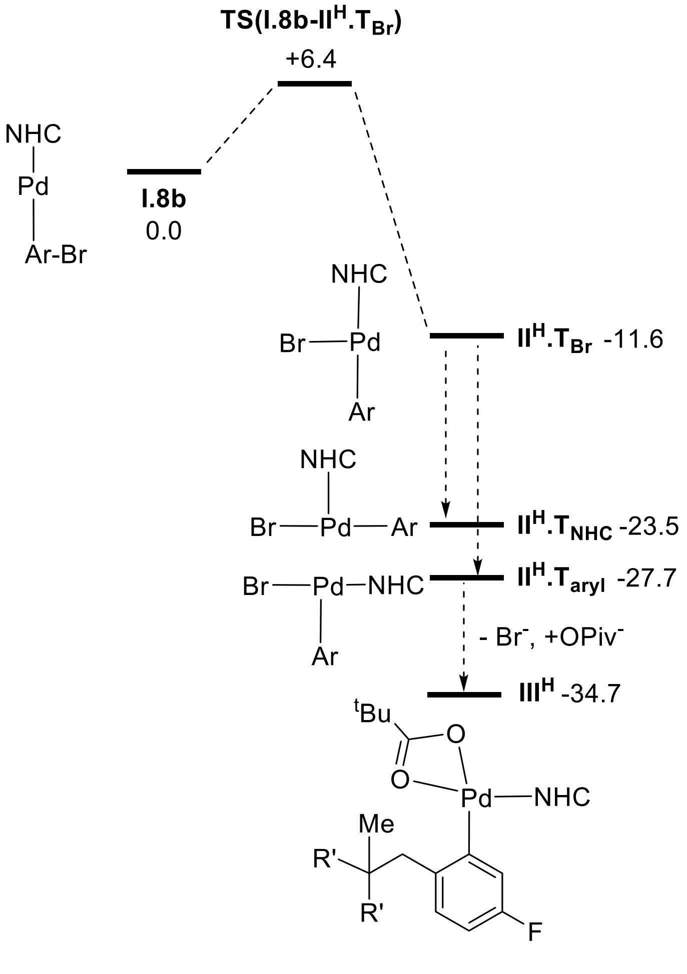

Figure S14. Computed free energy reaction profile (kcal/mol at 413 K) for the oxidative addition of **8b** at Pd-NHC, I. Level of theory: B97D(def2tzvp, 1,2-$C_6H_4Cl_2$)//BP86(SDD, 6-31G**). **TS(1.8b-II^H.T$_{Br}$)** leads initially to the T_{Br} isomer of **II^H**; isomerization to the T_{aryl} or T_{NHC} isomers is assumed to be facile.

9.2. Reaction Profiles for Various R Substituents

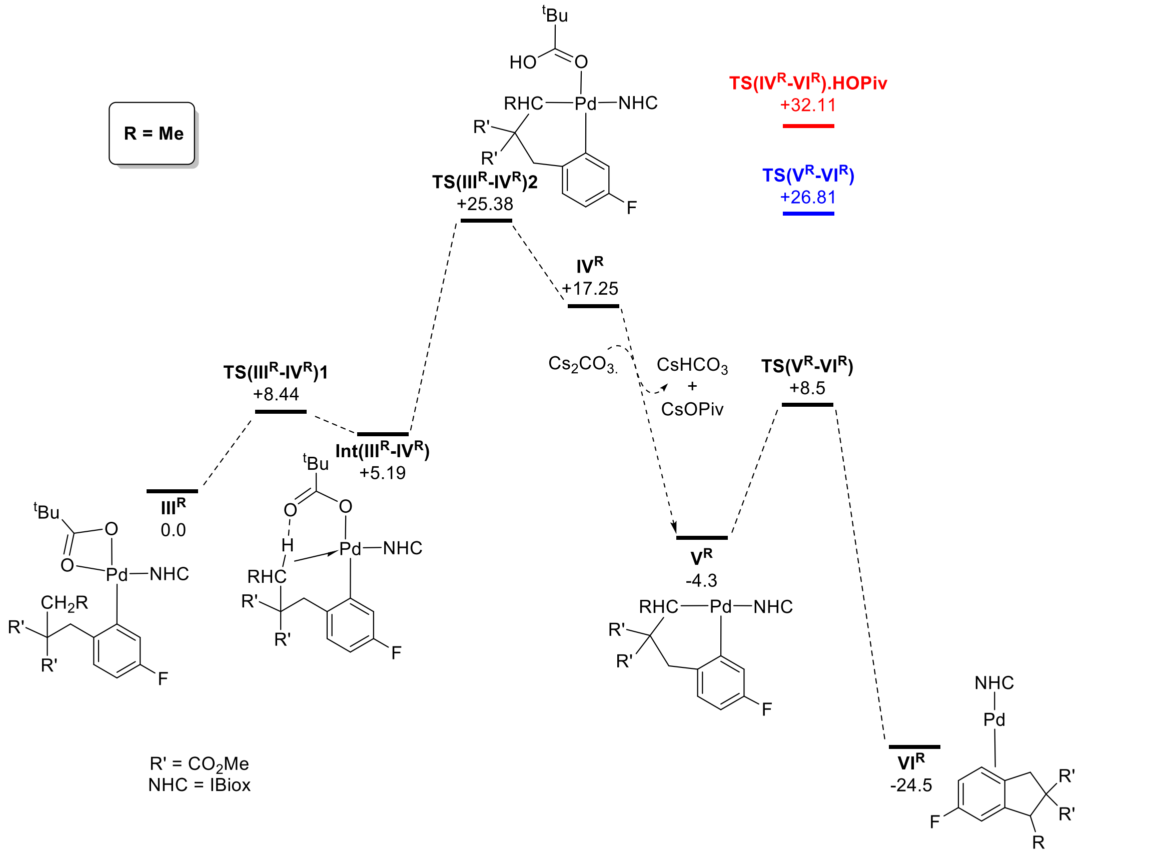

Figure S15. Computed free energy reaction profile (kcal/mol at 413 K) for the C–H cyclization of **8a** starting from intermediate **III**$^{\mathbf{R}}$ when R = Me. Level of theory: B97D(def2tzvp, 1,2-$C_6H_4Cl_2$)//BP86(SDD, 6-31G**). Alternative transition state energies are also indicated for C–C coupling from **V**$^{\mathbf{R}}$ without proton transfer to carbonate (blue) and direct from **IV**$^{\mathbf{R}}$ without loss of HOPiv (red). In both cases C–C coupling would be rate-limiting, contrary to experiment.

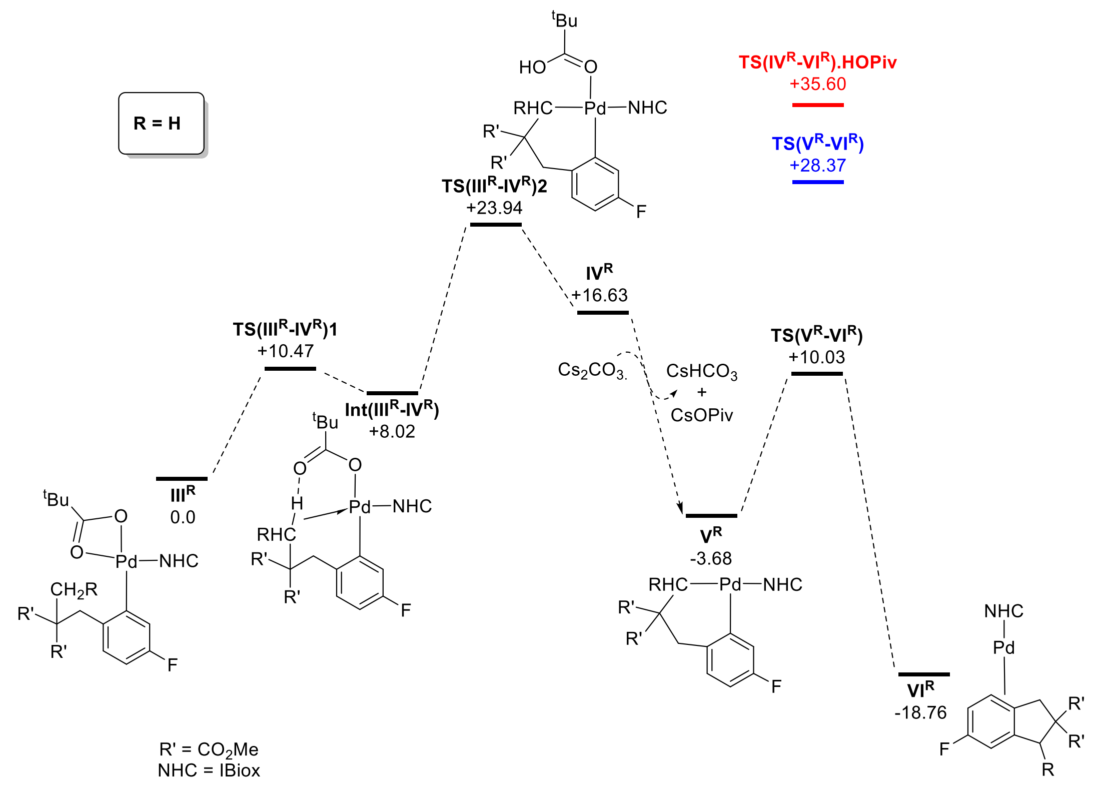

Figure S16. Computed free energy reaction profile (kcal/mol at 413 K) for the C–H cyclization of **8b** starting from intermediate **III**^{R} when R = H. Level of theory: B97D(def2tzvp, 1,2-$C_6H_4Cl_2$)//BP86(SDD, 6-31G**). Alternative transition state energies are also indicated for C–C coupling from **V**^{R} without proton transfer to carbonate (blue) and direct from **IV**^{R} without loss of HOPiv (red). In both cases C–C coupling would be rate-limiting, contrary to experiment.

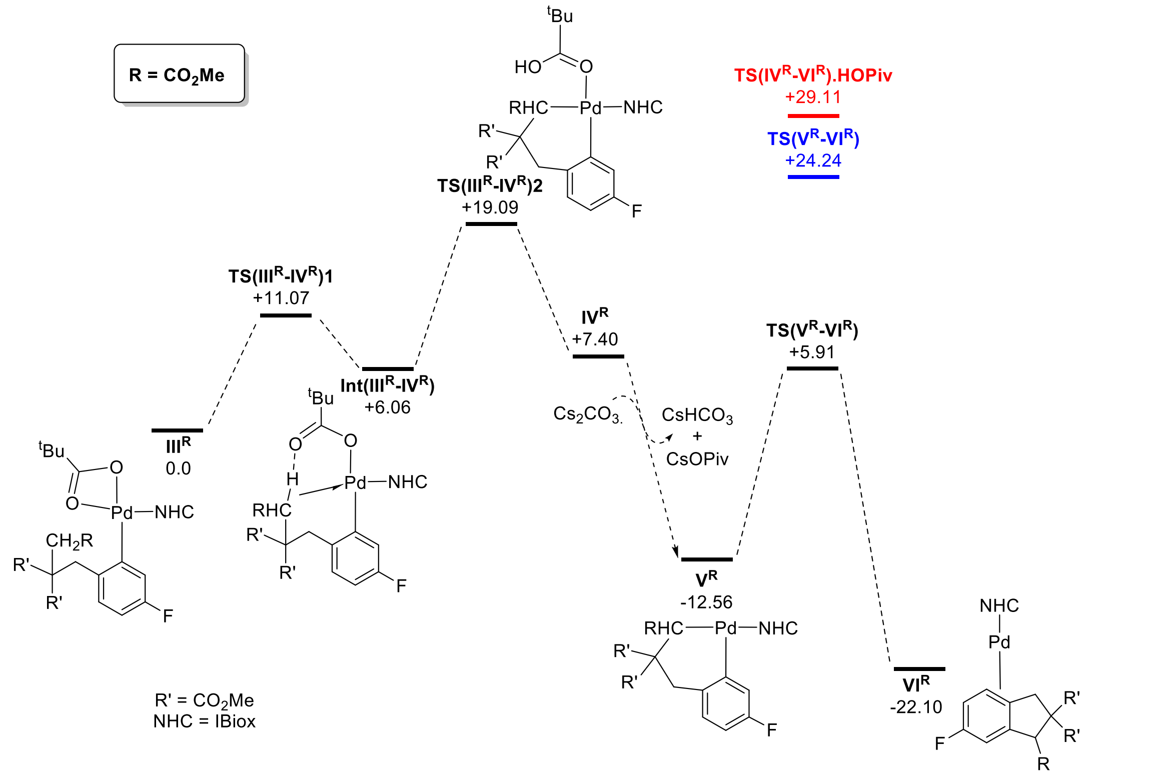

Figure S17. Computed free energy reaction profile (kcal/mol at 413 K) for the C–H cyclization of **8c** starting from intermediate **III^R** when R = CO_2Me. Level of theory: B97D(def2tzvp, 1,2-$C_6H_4Cl_2$)//BP86(SDD, 6-31G**). Alternative transition state energies are also indicated for C–C coupling from **V^R** without proton transfer to carbonate (blue) and direct from **IV^R** without loss of HOPiv (red). In both cases C–C coupling would be rate-limiting, contrary to experiment.

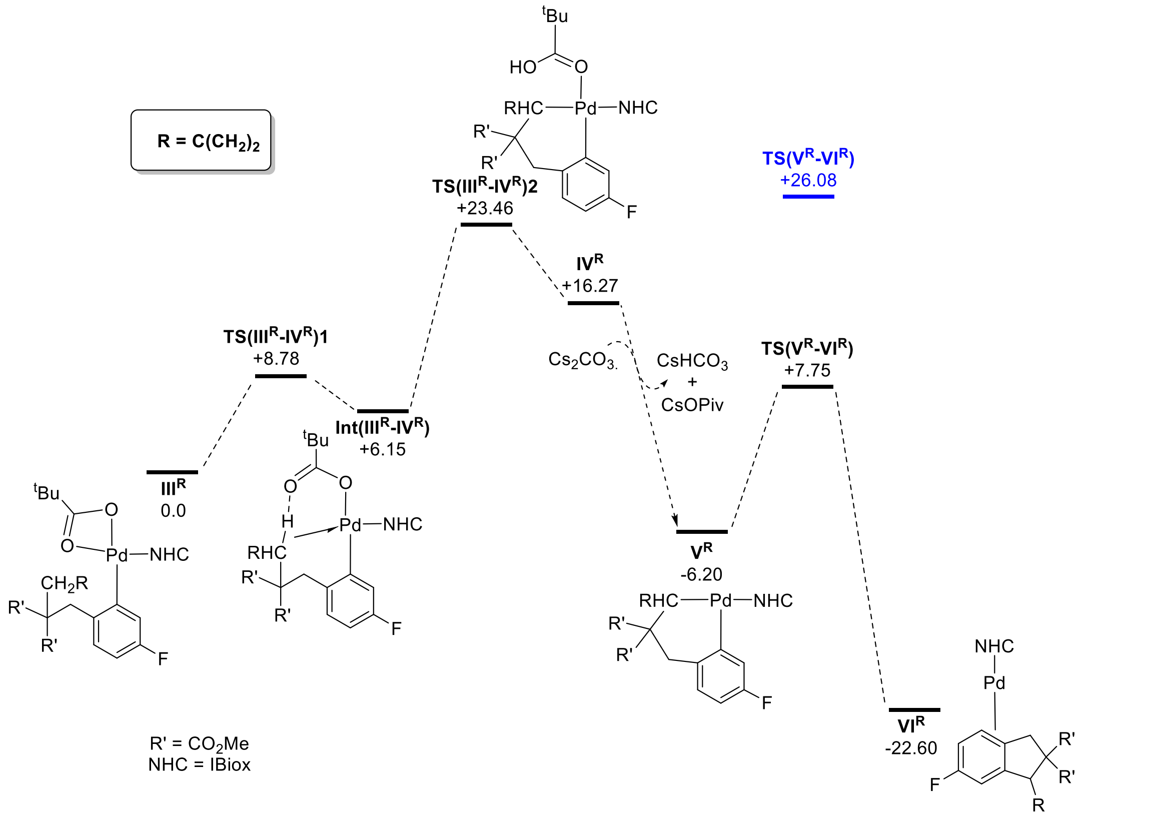

Figure S18. Computed free energy reaction profile (kcal/mol at 413 K) for the C–H cyclization of **8d** starting from intermediate $\mathbf{III^R}$ when R = $C(CH_2)_2$. Level of theory: B97D(def2tzvp, 1,2-$C_6H_4Cl_2$)//BP86(SDD, 6-31G**). Alternative transition state energy for C–C coupling from $\mathbf{V^R}$ without proton transfer to carbonate indicated in blue.

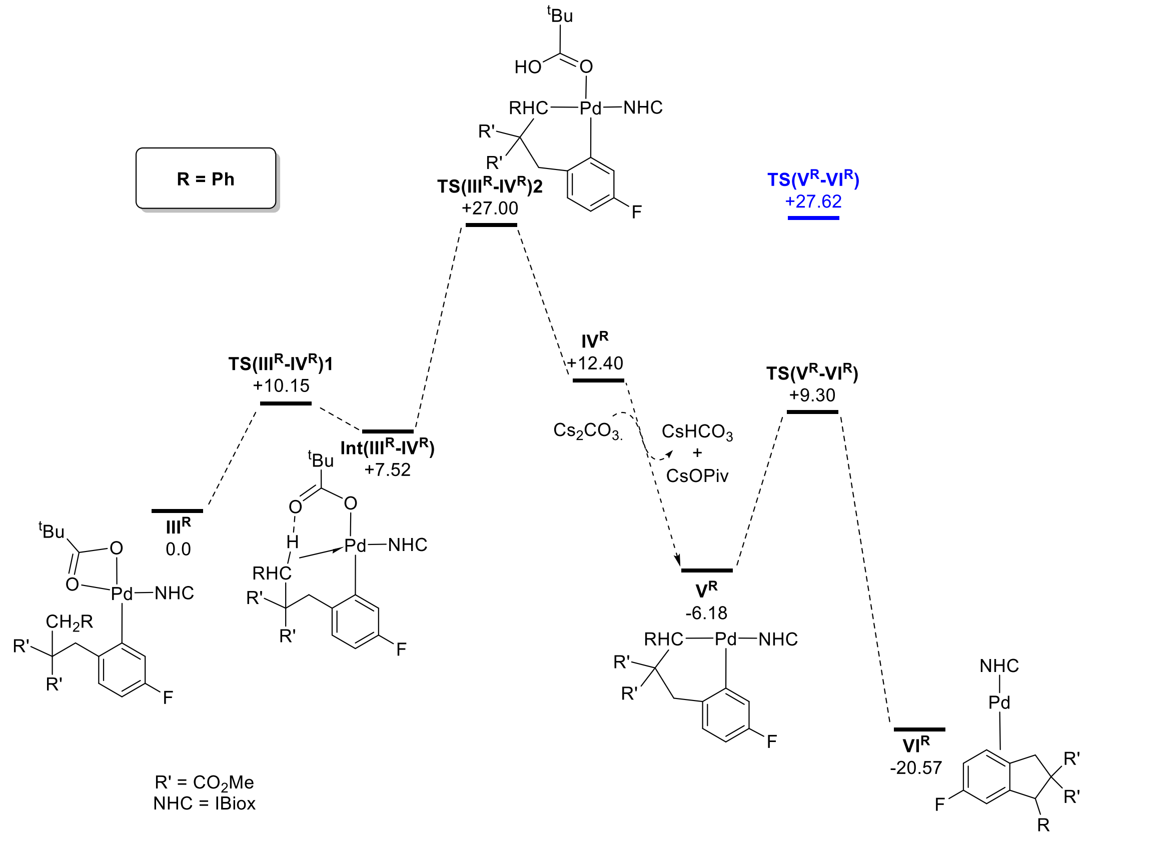

Figure S19. Computed free energy reaction profile (kcal/mol at 413 K) for the C–H cyclization of **8f** starting from intermediate **III^{R}** when R = Ph. Level of theory: B97D(def2tzvp, 1,2-$C_6H_4Cl_2$)//BP86(SDD, 6-31G**). Alternative transition state energy for C–C coupling from **V^{R}** without proton transfer to carbonate indicated in blue.

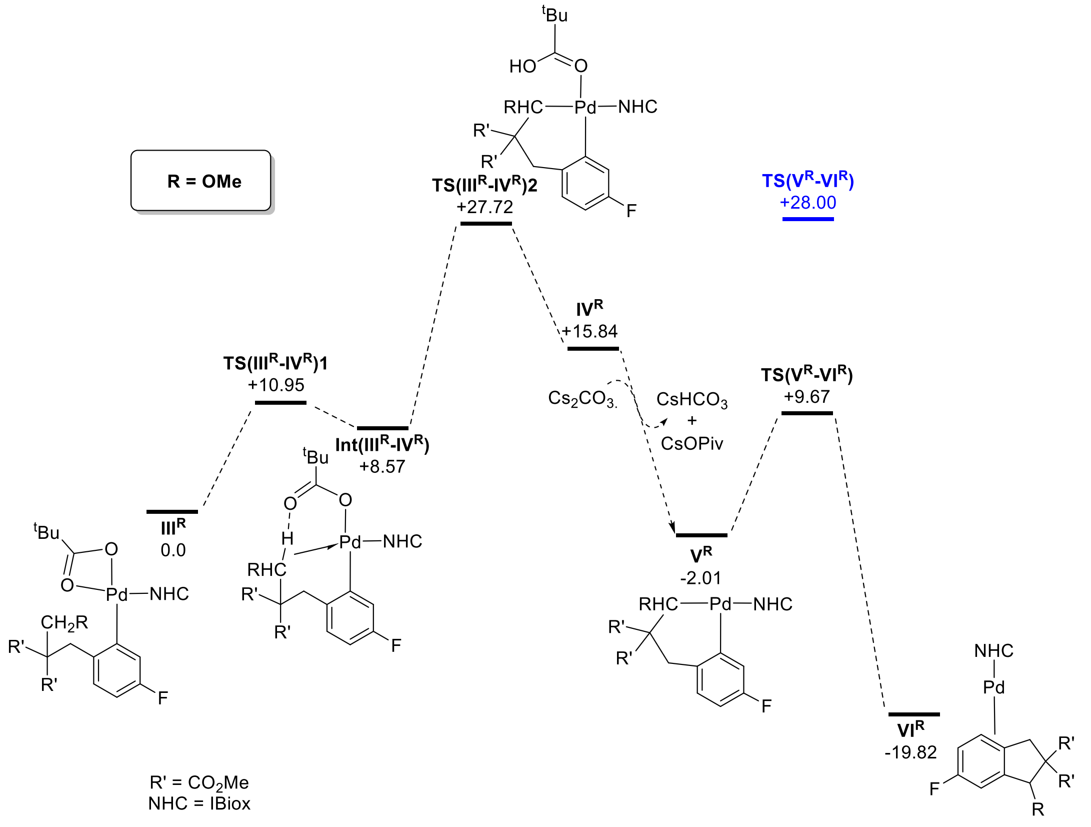

Figure S20. Computed free energy reaction profile (kcal/mol at 413 K) for the C–H cyclization of **8g** starting from intermediate **III**$^{\mathbf{R}}$ when R = OMe. Level of theory: B97D(def2tzvp, 1,2-$C_6H_4Cl_2$)//BP86(SDD, 6-31G**). Alternative transition state energy for C–C coupling from **V**$^{\mathbf{R}}$ without proton transfer to carbonate indicated in blue.

9.3. Comparison of Different Reductive Elimination Pathways

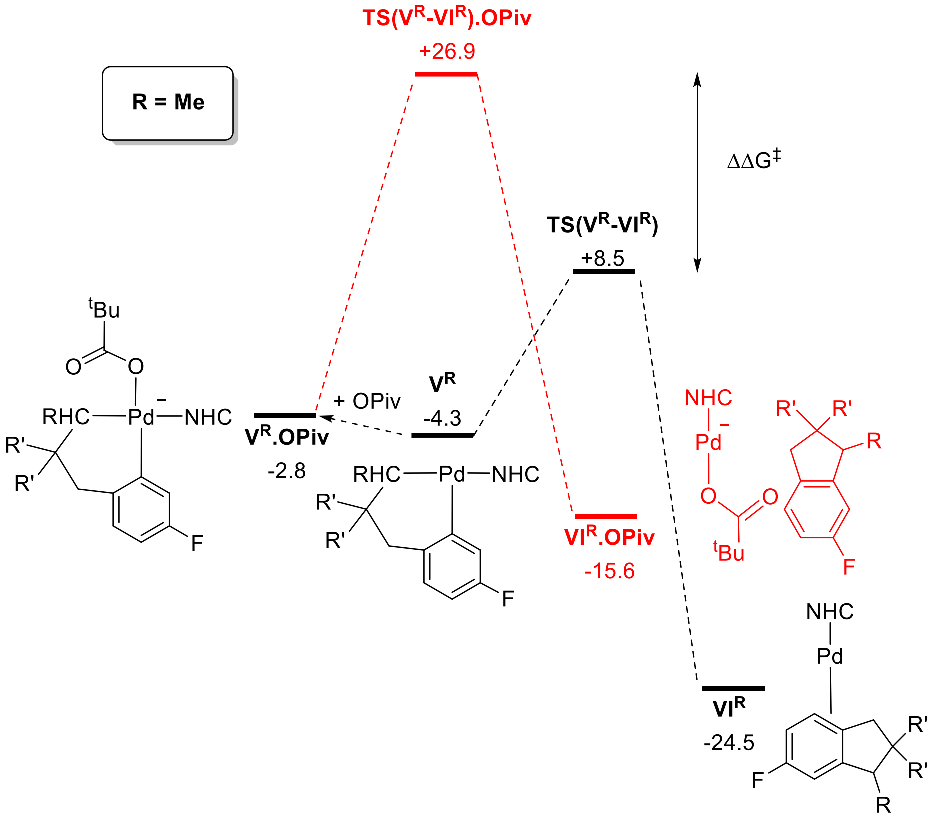

R	Me	H	CO_2Me	$C(CH_2)_2$	Ph	OMe
ΔΔG‡/kcal/mol	12.4	13.7	13.6	18.7	15.4	17.2

Figure S21. Computed free energy reaction profile (kcal/mol at 413 K) comparing C–C coupling from 3-coordinate $\mathbf{V^R}$ (black) and from 4-coordinate $\mathbf{V^R}$**.OPiv** (red), the latter formed by OPiv addition to $\mathbf{V^R}$. Profiles show the case where R = Me and tabulated ΔΔG‡ data for other substituents show a clear preference for reaction via $\mathbf{V^R}$ for all R. Level of theory: B97D(def2tzvp, 1,2-$C_6H_4Cl_2$)//BP86(SDD, 6-31G**).

9.4. Proton Transfer to Terminal Carbonate Base

Three models were assessed: (1) free anions; (2) tight ion-pairs, including the Cs^+ counterions and (3) a 'cluster' model with one $PhCF_3$ solvent molecule associated with each Cs^+ cation. In the latter case geometries featuring Cs···F and O···H contacts were favoured over K···π-arene geometries. The computed free energies of proton transfer shown in Scheme S1 indicate a significant effect of including the Cs^+ counterion, but that the subsequent effect of specific solvation as computed in Model 3 is relatively small. Data in the full paper are based on Model 2.

Model		G/kcal/mol
1.	HOPiv + CO_3^{2-} ⟶ HCO_3^- + $^-$OPiv	-37.6
2.	HOPiv + Cs_2CO_3 ⟶ $CsHCO_3$ + CsOPiv	-17.0
3.	HOPiv + $(PhCF_3)_2Cs_2CO_3$ ⟶ $(PhCF_3)CsHCO_3$ + $(PhCF_3)$CsOPiv	-18.9

Scheme S1. Computed free energies for proton transfer to the terminal carbonate base using three different chemical models. Level of theory: B97D(def2tzvp, 1,2-$C_6H_4Cl_2$)//BP86(SDD, 6-31G**).

9.5. Computed Rate-Determining Process when R = CO_2Me

With substrate **8c** the overall barriers computed for C–H activation (**III^R → TS(III^R-IV^R)2**, 19.1 kcal/mol) and C–C coupling (**V^R → TS(V^R-VI^R),** 18.5 kcal/mol) are relatively close (see Figure S17). This difference, along with the computed binding energy of adding the CsOPiv ion-pair to **V^R** to form **IV^R.CsOPiv** were both found to be functional dependent (see Scheme S2).

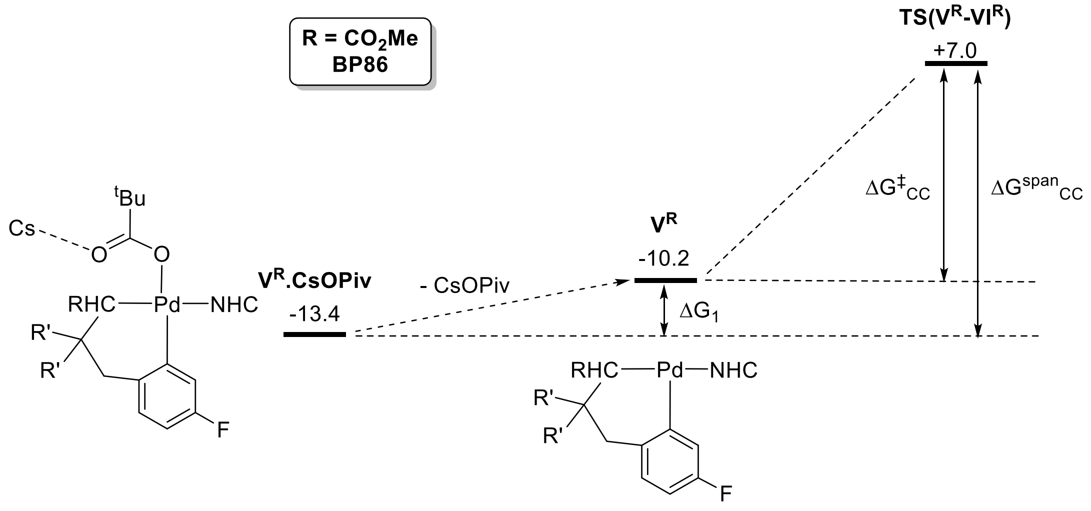

Functional	BP86	BLYP	B3LYP	PBE	PBE0	B97D3	B97D	M06	wB97xD	TPSS	B3PW91
						R = CO_2Me					
$\Delta G^{span}{}_{CHA}$	14.6	18.5	19.4	15.9	17.0	17.8	19.1	20.2	22.2	18.5	15.9
ΔG_1 (Cs^+)	+3.2	+0.7	+0.6	-2.9	-2.0	-4.3	+15.8	-3.6	+9.8	-0.8	+1.0
ΔG_1 (K^+)	-1.2	-3.9	-4.0	-7.7	-6.9	-10.0	-4.4	-8.3	-4.5	-5.9	-3.8
$\Delta G^{\ddagger}{}_{CC}$	17.2	19.4	21.1	16.6	19.2	18.4	18.5	12.8	20.0	19.5	19.4
$\Delta G^{span}{}_{CC}$	20.4	20.1	21.7	16.6	19.2	18.4	34.3	12.8	29.8	19.5	20.4
$\Delta\Delta G^{span}{}_{CC\text{-}CHA}$	+5.8	+1.6	+2.3	+0.7	+2.2	+0.6	+15.2	-7.4	+7.6	+1.0	+4.5
						R = Me					
$\Delta G^{span}{}_{CHA}$	21.4	26.1	27.6	23.2	24.9	25.2	25.4	26.5	29.2	25.7	23.3
ΔG_1	-3.3	-6.7	-7.1	-10.5	-9.8	-12.4	+21.3	-9.3	+6.3	-8.0	-6.4
$\Delta G^{\ddagger}{}_{CC}$	11.9	13.3	14.6	11.5	13.4	12.6	12.8	7.7	13.5	13.6	13.4
$\Delta G^{span}{}_{CC}$	11.9	13.3	14.6	11.5	13.4	12.6	34.1	7.7	19.8	13.6	13.4
$\Delta\Delta G^{span}{}_{CC\text{-}CHA}$	-9.5	-12.8	-13.0	-11.7	-11.5	-12.6	+8.7	-18.8	-9.3	-12.1	-9.9

Scheme S2. Functional dependence for the computed free energies (kcal/mol, R = CO_2Me, Me) for **$\Delta G^{span}{}_{CHA}$** (the overall barrier for C–H activation, see Figures S15-S20) **ΔG_1**, **$\Delta G^{\ddagger}{}_{CC}$** and **$\Delta G^{span}{}_{CC}$** (as defined in the figure) and **$\Delta\Delta G^{span}{}_{CC\text{-}CHA}$**, the difference in the overall energy spans for C–H activation and C–C coupling; a positive value indicates a higher barrier for C–C coupling. Level of theory: XC(def2tzvp, 1,2-$C_6H_4Cl_2$)//BP86(SDD, 6-31G**), where XC is the functional employed; the figure illustrates results obtained with XC = BP86 and R = CO_2Me.

In general, with R = CO_2Me the barrier for C–C coupling is higher than that for C–H activation ($\Delta\Delta G^{span}_{CC\text{-}CHA}$ is +ve), the only exception being with the M06 functional. Note that the **IV^R.KOPiv** ion-pair shows unusual stability when computed with the B97D and ωB97xD functionals. With R= Me the higher energy span is associated with C–H activation, although the spurious results with B97D and ωB97xD remain. Moreover, this appears to be linked to the use of Cs, as calculation of KOPiv dissociation from **IV^R.KOPiv** gives far more consistent results, with the data for B97D and ωB97xD lying within the range defined by the other functionals.

9.6. Model and Functional Testing

The evolution of the computed energy as the different corrections to the SCF energy computed with BS1 were assessed. The anomalously large barrier when R = Ph is computed throughout. "Final (B97D)" is the level of theory employed in the main text.

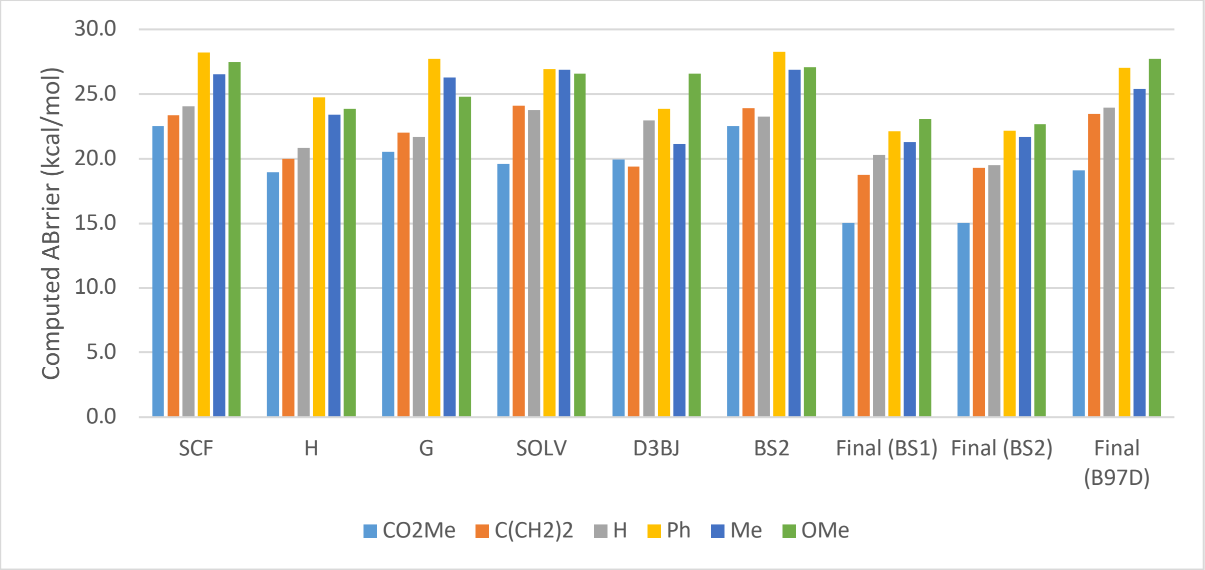

Figure S22. C–H activation barriers (kcal/mol) computed for Pd(IBiox)Ar(OPiv) as a function of α-substituent R and the energy corrections applied.

Single point energy corrections based on the BP86-optimised geometries were performed with a range of different functionals. “B97D” is the level employed for the main text and shows a good trend in $\Delta G^{\ddagger}$ as a function of R, with the exception of the anomalously high barrier when R = Ph. Similar trends are seen for BP86, BLYP and TPSS. For other functionals the barrier for R = cC_3H_4 is also too high but for all functionals the trend R = CO_2Me < H < Me < OMe is computed.

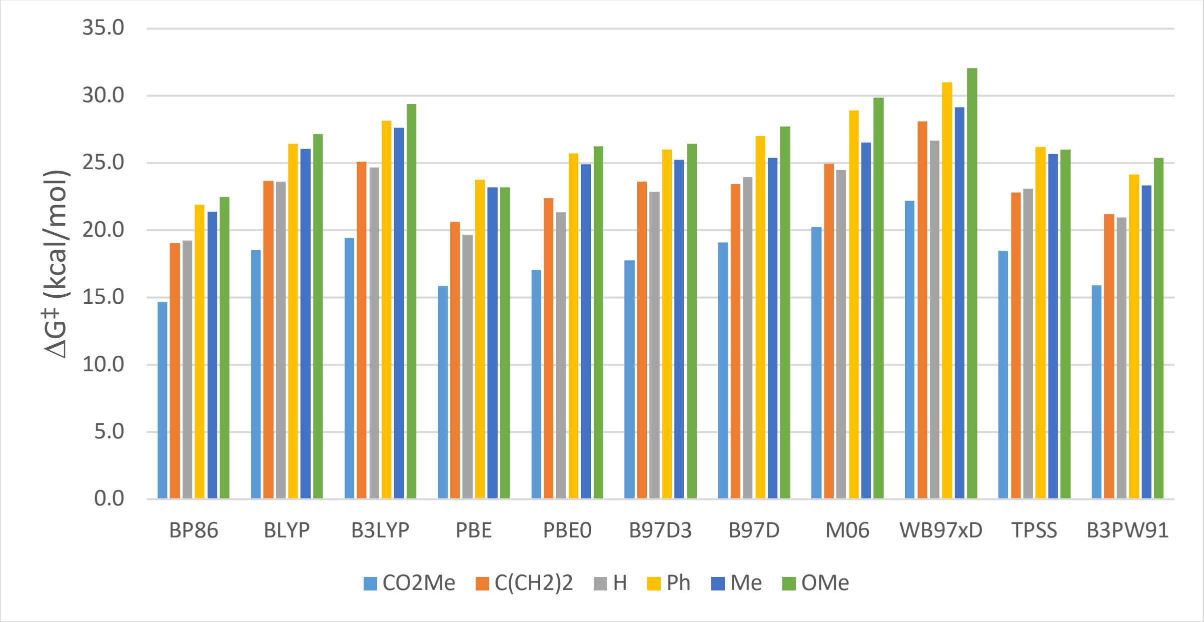

Figure S23. C–H activation barriers (kcal/mol) computed for Pd(IBiox)Ar(OPiv) with different functionals as a function of α-substituent R. Free energy barriers are computed at 413 K and include corrections for solvent and (for BP86, BLYP, B3LYP, PBE, PBE0, TPSS and B3PW91) dispersion.

An alternative model where carbonate acts as an intramolecular base was also considered. Computed C–H activation barriers relative to a $[Pd(IBiox)Ar(CO_3)]^-$ reactant are shown in Figure S10 for the range of functionals tested. Single point energy corrections based on the BP86-optimised geometries were performed with a range of different functionals. Barriers for R = $C(CH_2)_2$ and H are systematically too high, in particular that for R = H is higher than R = Me, contrary to a body of experimental evidence suggesting the reverse relative reactivity. In several cases the barriers for R = $C(CH_2)_2$ and H exceed that for R = OMe, the least reactive substituent experimentally.

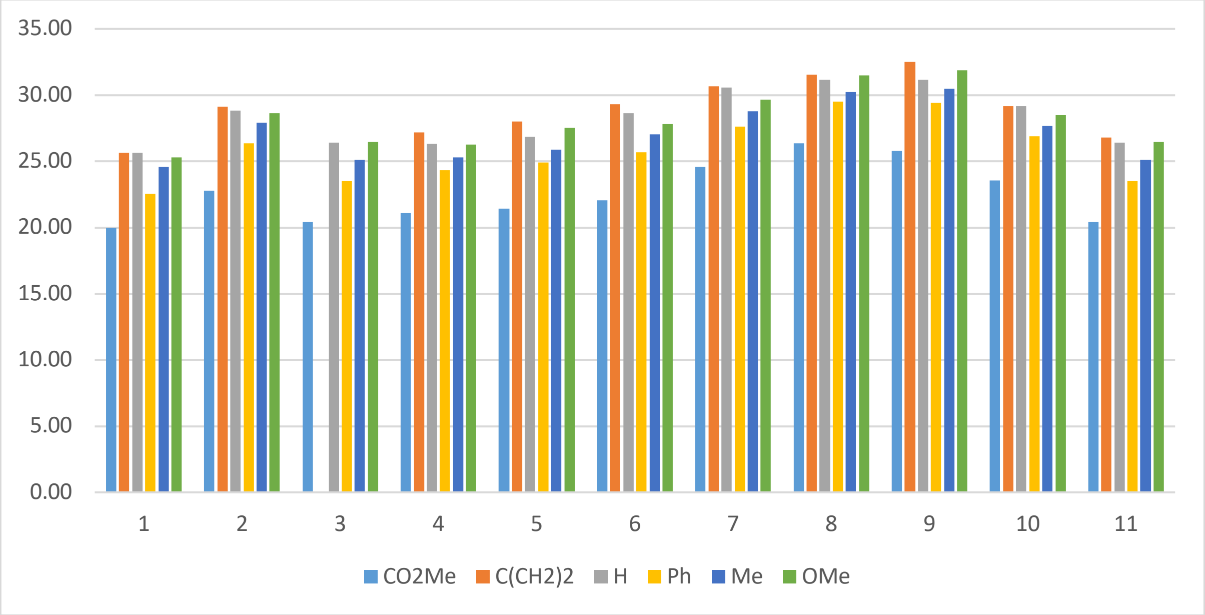

Figure S24. C–H activation barriers (kcal/mol) computed for $[Pd(IBiox)Ar(CO_3)]^-$ with different functionals as a function of α-substituent R. Free energy barriers are computed at 413 K and include corrections for solvent and (for BP86, BLYP, B3LYP, PBE, PBE0, TPSS and B3PW91) dispersion.

9.7. Geometries of the Agostic Intermediates for Selected Substituents

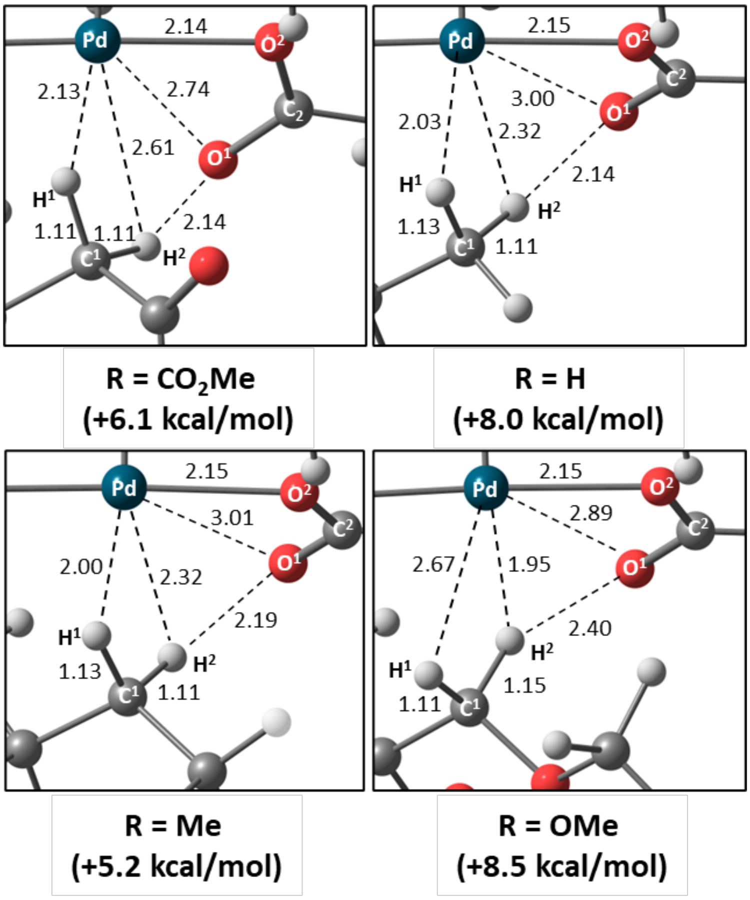

Figure S25. Details of the computed geometries of **Int(III^R-IV^R)** for R = CO_2Me, H, Me and OMe, with selected distances in Å and relative free energies indicated in kcal/mol..

9.8. Natural Bond Orbital Analyses

<table>
<tr><th colspan="2">Pd → C–H σ* back-donation (Σ = 4.8 kcal/mol)</th></tr>
<tr><td>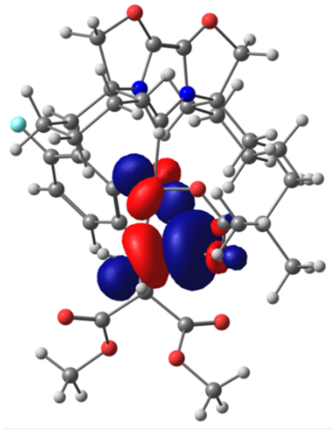
Pd LP → C-H σ* (2.5 kcal/mol)</td><td>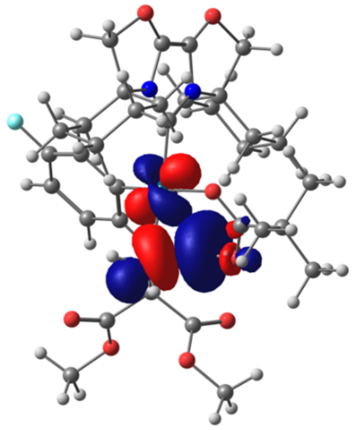
Pd LP → C-H σ* (2.4 kcal/mol)</td></tr>
<tr><th colspan="2">C–H → Pd σ-donation (Σ =44.3 kcal/mol)</th></tr>
<tr><td>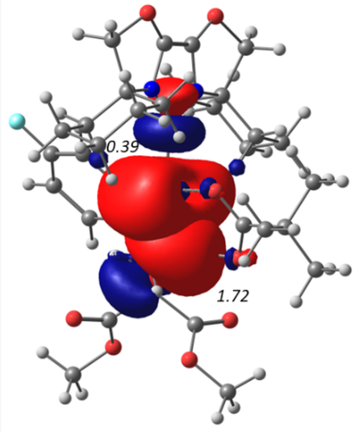

C-H σ → Pd-C_{NHC} σ* (42.4 kcal/mol)</td><td>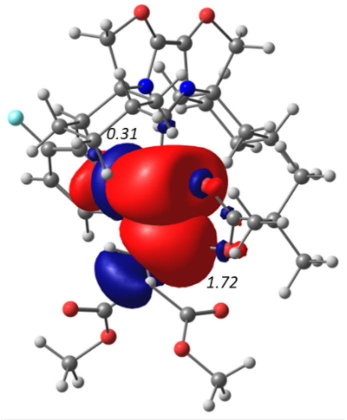

C-H σ → Pd-C_{Ar} σ* (1.9 kcal/mol)</td></tr>
<tr><th colspan="2">O → H–C σ* donation (Σ = 144.0 kcal/mol)</th></tr>
<tr><td>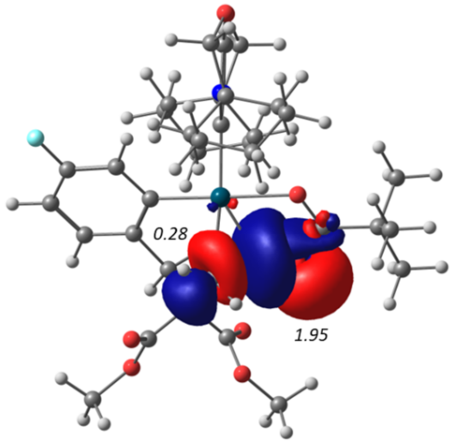

O LP → C-H σ* (10.8 kcal/mol)</td><td>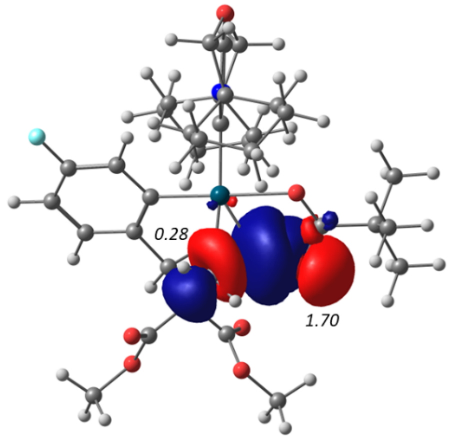

O LP → C-H σ* (98.0 kcal/mol)</td></tr>
</table>

<table>
<tr><td>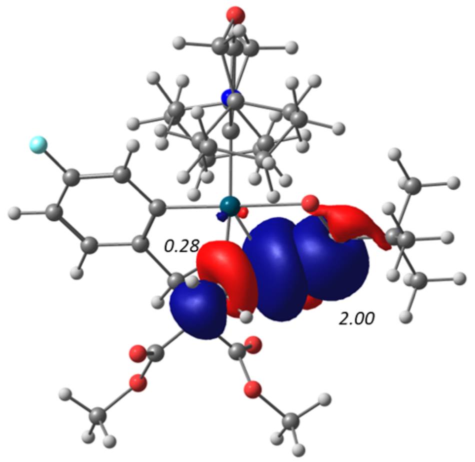

C-O σ → C-H σ* (7.1 kcal/mol)</td><td>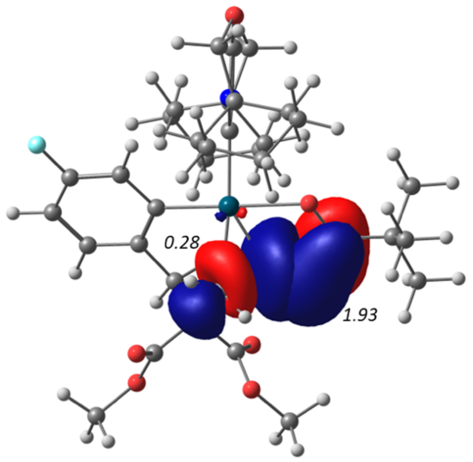

C=O π → C-H σ* (28.1 kcal/mol)</td></tr>
<tr><td colspan="2">Total C-H···Pd agostic interaction = 49.1 kcal/mol</td></tr>
<tr><td colspan="2">Total O→C-H σ* donation = 144.0 kcal/mol</td></tr>
<tr><td colspan="2">χ = 144.0/49.1 = 2.93</td></tr>
</table>

Figure S26. Donor-acceptor interactions between NBOs within **TS(III^{H}-IV^{H})2** with 2nd order interaction energies in kcal/mol and NBO occupations in italics. $O_1 \rightarrow H_2 – C_1$ donation include components from O lone pairs, the C-O σ-BMO and C=O π-BMO; the $C_1 – H_2 \cdots$Pd agostic interactions include both σ- and π-back donation.

Table S1. Computed values of the $O_1 \rightarrow H_2–C_1$ donation and the $C_1–H_2 \rightarrow Pd$ agostic interaction as a function of R in **TS(III^R-IV^R)2** as quantified via NBO 2nd order perturbation analyses. Each value is made up of several contributions – see Figure S26 for details of how these values are arrived at for R = H. χ is the ratio of $O_1 \rightarrow H_2–C_1$ donation to the $C_1–H_2 \rightarrow Pd$ agostic interaction and the overall barriers to C–H activation are also indicated.

R	$O_1 \rightarrow H_2–C_1$ donation (kcal/mol)	$C_1–H_2 \rightarrow Pd$ interaction (kcal/mol)	χ	Computed Barrier (kcal/mol)
CO_2Me	138.9	27.9	4.97	19.09
$C(CH_2)_2$	159.4	40.5	3.93	23.46
H	144.0	49.1	2.93	23.94
Ph	156.5	43.4	3.60	27.72
Me	158.7	51.0	3.11	25.38
OMe	127.0	49.8	2.55	27.00
$(Me)_2$	138.7	50.3	2.76	37.18

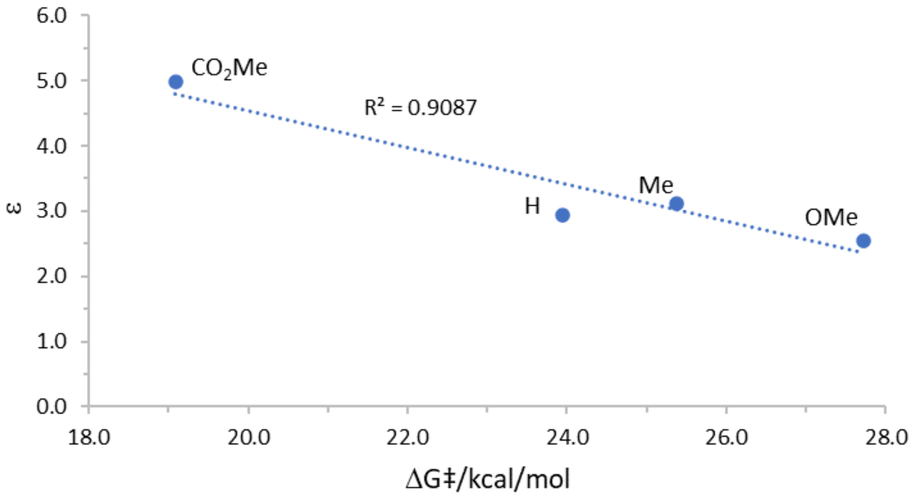

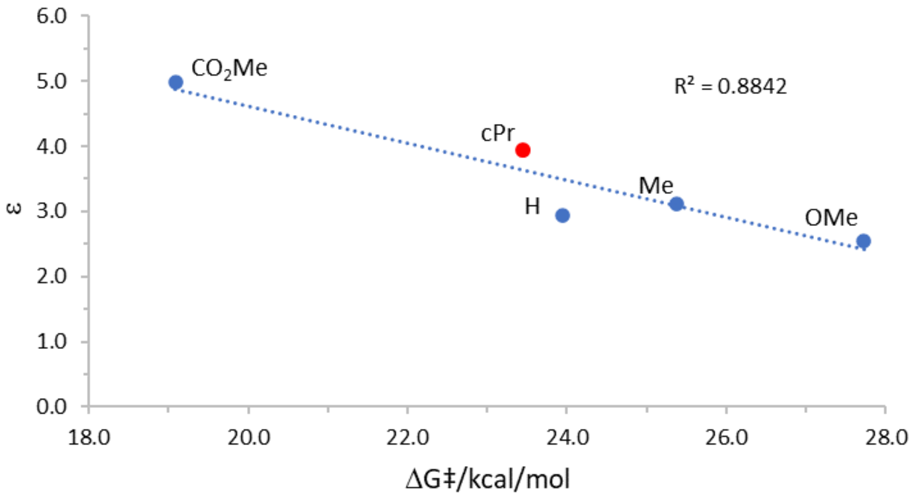

Figure S27. A plot of χ (the ratio of $O_1 \rightarrow H_2–C_1$ donation to the $C_1–H_2 \rightarrow Pd$ agostic interaction) vs $\Delta G^{\ddagger}$ in **TS(III^R-IV^R)2** for various R. Top plot for R = CO_2Me, H, Me and OMe; lower plot includes data for R = $C(CH_2)_2$.

Table S2. Computed NBO Charges at the transfering hydrogen, H^2, in **III^R**, **Int(III^R-IV^R)** and **TS(III^R-IV^R)2** and (after the slash) n, the sp^n hybridisation state of the C-H bond undergoing activation.[a]

R	III^R	Int(III^R-IV^R)	TS(III^R-IV^R)2
CO_2Me	0.300/3.2	0.328/3.2	0.404/4.3
cPr	0.293/2.7[b]	0.312/2.5	0.435/2.5
H	0.272/3.1[c]	0.291/2.9	0.393/3.4
Ph	0.282/3.3	0.290/3.4	0.400/4.7
Me	0.277/3.3	0.296/3.1	0.403/4.3
OMe	0.241/2.8	0.191/3.3	0.36/3.6

[a]For **III^R** an average of the two methylene H atoms/C-H bonds was taken; no agaostic intermediate was loacted when R = $(Me)_2$; [b]for R = $C(CH_2)_2$ there is only the tertiary hydrogen to consider; [c]for R = H the two hydrogens/CH bonds in equivalent positions to the CH_2 methylene group when R = Me were taken;

9.9. Computed Geometries (Å) and Energies (hartrees)

Oxidative Addition of 8b.

I.8b

```
79
BP86
SCF  =          -1968.20710513
H(0 K)=         -1967.578255
H(413 K)=       -1967.500282
G(413 K)=       -1967.713771
B97D       (1,2-C6Cl2H4)=       -
4529.95152186
Low Freq. = 8.4197cm-1,12.6222cm-
1
```

79
I.8b

```
C  -0.03159 -2.17705 -1.61526
C   0.94826 -1.17733 -1.29341
C   1.78433 -1.29562 -0.12091
C   1.62167 -2.45031  0.66073
C   0.70215 -3.47677  0.34606
C  -0.11699 -3.31100 -0.76077
Pd -0.91343 -0.39383 -0.63052
C   4.70389 -1.08401 -1.16755
C   4.27790 -0.55861  0.22149
C   4.66493 -1.60541  1.28694
O   5.15350 -2.70283  1.07135
C   2.75522 -0.20548  0.29486
F  -1.01918 -4.27779 -1.08662
C  -2.39655  0.76888  0.15313
N  -2.35414  2.08937  0.53656
C  -3.54797  2.56123  1.08913
C  -4.39924  1.49166  1.09141
N  -3.68609  0.44365  0.50386
C  -1.39471  3.20630  0.36760
C  -2.07836  4.22812  1.33969
O  -3.50434  3.88857  1.39837
C   0.02404  2.80046  0.80920
C   1.07219  3.89564  0.52978
C   1.05207  4.32981 -0.94777
C  -0.35629  4.78284 -1.37348
C  -1.40261  3.68334 -1.10708
O  -5.64330  1.12303  1.51000
C  -5.87610 -0.17707  0.87361
C  -4.47505 -0.80788  0.55326
C  -3.94342 -1.71974  1.68782
C  -4.65107 -3.08835  1.72613
C  -4.58201 -3.79766  0.36102
C  -5.16441 -2.90241 -0.74851
C  -4.44483 -1.54085 -0.80268
Br  1.51937 -0.01301 -2.79020
C   5.06231  0.73921  0.52619
O   4.59880  1.87040  0.56006
O   6.38312  0.46349  0.71775
C   7.21176  1.62282  0.97471
O   4.37727 -1.14129  2.54262
C   4.74315 -2.04416  3.61292
H  -3.37713 -1.68396 -1.06707
H  -4.87869 -0.88655 -1.58103
H  -6.24912 -2.75224 -0.57050
H  -5.08028 -3.39815 -1.73186
H  -3.52757 -4.03189  0.11920
H  -5.12260 -4.76051  0.40135
H  -4.19488 -3.71269  2.51511
H  -5.71367 -2.95810  2.01631
H  -4.04181 -1.19253  2.65481
H  -2.86152 -1.86023  1.50163
H  -6.45014 -0.01110 -0.05804
H  -6.47566 -0.77550  1.57518
H  -1.65535  4.13812  2.35865
H  -2.01052  5.27175  1.00021
H  -2.41955  4.02664 -1.37267
H  -1.18265  2.79849 -1.73361
H  -0.37430  5.05136 -2.44490
H  -0.62739  5.70650 -0.82254
H   1.36531  3.47568 -1.57909
H   1.78568  5.13792 -1.11892
H   0.88003  4.77694  1.17570
H   2.07196  3.51660  0.80404
H   0.28220  1.87772  0.24767
H   0.00921  2.52190  1.87896
H  -0.48905 -2.22564 -2.60750
H   2.21203 -2.54532  1.57724
H   0.61296 -4.37220  0.96597
H   2.60651  0.68571 -0.33297
H   2.53473  0.10207  1.33062
H   4.49323 -0.32939 -1.94114
H   5.77529 -1.33074 -1.17438
H   4.13779 -1.99740 -1.40671
H   4.46199 -1.52818  4.54091
H   4.20166 -2.99938  3.51963
H   5.82536 -2.24929  3.59144
H   7.17343  2.32159  0.12396
H   6.87152  2.14774  1.88153
H   8.22857  1.23042  1.10965
```

$TS(1.8b-II^{H}.T_{Br})$

```
79
BP86
SCF  =          -1968.19955579
H(0 K)=         -1967.571300
H(413 K)=       -1967.494146
G(413 K)=       -1967.704974
B97D       (1,2-C6Cl2H4)=       -
4529.94261643
Low Freq.= -80.8679cm-1,9.9384cm-
1
```

79
$TS(1.8b-II^{H}.T_{Br})$

```
C   0.04299 -2.70974 -1.20658
C   0.81091 -1.54043 -0.94829
```

C   1.79689 -1.49272 0.08715
C   1.90119 -2.64120 0.89988
C   1.12989 -3.79905 0.69638
C   0.20691 -3.80685 -0.35230
Pd -0.75563 -0.24908 -0.63420
C   4.47919 -0.89327 -1.33580
C   4.17345 -0.40447 0.09790
C   4.83678 -1.36788 1.10349
O   5.43708 -2.39395 0.82722
C   2.63844 -0.26038 0.36811
F -0.56553 -4.91090 -0.55610
C -2.33864 0.80767 0.11045
N -2.39241 2.11460 0.54404
C -3.61396 2.48062 1.11435
C -4.38607 1.35312 1.08176
N -3.60137 0.37831 0.45985
C -1.52709 3.30633 0.38888
C -2.27319 4.24857 1.39524
O -3.66765 3.79824 1.46222
C -0.07363 3.00293 0.79982
C   0.88152 4.18249 0.53346
C   0.80448 4.64808 -0.93249
C -0.64136 4.99919 -1.32798
C -1.59607 3.81661 -1.07318
O -5.60017 0.88268 1.48798
C -5.74092 -0.40415 0.80084
C -4.29658 -0.92806 0.48410
C -3.71487 -1.81493 1.61466
C -4.33493 -3.22585 1.63548
C -4.21245 -3.91916 0.26565
C -4.83092 -3.04955 -0.84535
C -4.20177 -1.64263 -0.87795
Br 1.00051 -0.24658 -2.65780
C   4.80618 0.99302 0.29286
O   4.20213 2.05272 0.37982
O   6.16591 0.90093 0.31417
C   6.85569 2.16616 0.45400
O   4.65613 -0.92219 2.38602
C   5.28134 -1.74537 3.39888
H -3.12459 -1.71338 -1.13088
H -4.66847 -1.01127 -1.65604
H -5.92545 -2.96990 -0.68368
H -4.70153 -3.52970 -1.83159
H -3.14487 -4.10262 0.03820
H -4.70337 -4.90869 0.29347
H -3.84651 -3.82760 2.42253
H -5.40536 -3.16416 1.91892
H -3.85429 -1.30445 2.58545
H -2.62478 -1.88644 1.43834
H -6.30852 -0.24084 -0.13545
H -6.31209 -1.06466 1.46937
H -1.83037 4.16626 2.40637
H -2.29066 5.30251 1.08222
H -2.64047 4.08797 -1.31431
H -1.32159 2.96660 -1.72559
H -0.69675 5.29008 -2.39228
H -0.97418 5.88624 -0.75139
H   1.17482 3.83659 -1.58853
H   1.46957 5.51534 -1.09429
H   0.63110 5.03053 1.20351
H   1.91060 3.87336 0.78442
H   0.24668 2.11774 0.21136
H -0.04891 2.69840 1.86228
H -0.62572 -2.77184 -2.06750
H   2.59532 -2.61908 1.74636
H   1.23879 -4.67821 1.33612
H   2.28433 0.57903 -0.24824
H   2.50999 0.04632 1.41960
H   4.06010 -0.19116 -2.07326
H   5.56393 -0.98722 -1.48877
H   4.02075 -1.88129 -1.49508
H   5.05553 -1.25547 4.35571
H   4.87089 -2.76790 3.37887
H   6.36932 -1.79974 3.23425
H   6.61557 2.83265 -0.38963
H   6.56557 2.66302 1.39349
H   7.92513 1.91636 0.45931

II^{H}.T$_{Br}$
79
BP86
SCF =   -1968.21758155
H(0 K)=   -1967.588447
H(413 K)=   -1967.510441
G(413 K)=   -1967.724536
B97D   (1,2-C6Cl2H4)=   -
4529.96972396
Low Freq.= 8.3334cm-1, 11.5620cm-
1

79
II^{H}.T$_{Br}$
C   0.49042 -3.15054 -1.07984
C   0.85766 -1.93215 -0.46784
C   2.02931 -1.85324 0.33429
C   2.71954 -3.05329 0.61741
C   2.32774 -4.28394 0.07098
C   1.21957 -4.30787 -0.78346
Pd -0.55094 -0.52851 -0.32261
C   3.65846 0.12381 -1.32831
C   3.81611 0.02961 0.20702
C   5.04739 -0.84004 0.53416
O   5.76069 -1.41086 -0.27429
C   2.52587 -0.52986 0.89528
F   0.84100 -5.49030 -1.34092
C -2.17468 0.67533 0.24257
N -2.31369 2.02502 0.48019
C -3.58535 2.39485 0.91693
C -4.30470 1.23161 0.99321
N -3.43855 0.22842 0.56483
C -1.51276 3.24591 0.19808
C -2.36044 4.25689 1.04184
O -3.73237 3.73419 1.09137
C -0.06426 3.11187 0.69778
C   0.78697 4.34612 0.34094
C   0.75618 4.62417 -1.17346
C -0.68930 4.78561 -1.67941
C -1.55469 3.56127 -1.31894
O -5.52201 0.76347 1.38183
C -5.56808 -0.60417 0.85327
C -4.08617 -1.09743 0.70700
C -3.56563 -1.80808 1.98301

C	-4.14478	-3.22752	2.14163
C	-3.89855	-4.08075	0.88368
C	-4.45607	-3.38551	-0.37242
C	-3.86673	-1.97142	-0.54275
Br	-0.17904	0.02063	-2.67594
C	4.06873	1.44925	0.75709
O	3.24855	2.15389	1.33156
O	5.33737	1.85701	0.47424
C	5.65888	3.19879	0.91060
O	5.24386	-0.90358	1.88928
C	6.40530	-1.66932	2.28682
H	-2.77227	-2.03553	-0.71465
H	-4.28900	-1.46118	-1.42694
H	-5.56145	-3.32805	-0.30205
H	-4.23694	-3.98137	-1.27563
H	-2.80995	-4.24001	0.75753
H	-4.35328	-5.08018	1.00341
H	-3.69976	-3.70531	3.03232
H	-5.23430	-3.17011	2.33864
H	-3.79040	-1.18344	2.86702
H	-2.46321	-1.86664	1.90019
H	-6.06935	-0.57977	-0.13292
H	-6.16384	-1.20100	1.55854
H	-1.97784	4.32305	2.07817
H	-2.41581	5.26126	0.59926
H	-2.60475	3.71480	-1.62962
H	-1.18203	2.66820	-1.85454
H	-0.70616	4.92891	-2.77435
H	-1.12859	5.70640	-1.24407
H	1.23337	3.77904	-1.70609
H	1.34810	5.52660	-1.41113
H	0.41348	5.23572	0.88873
H	1.81804	4.16814	0.68898
H	0.37305	2.21534	0.21896
H	-0.06081	2.92581	1.78701
H	-0.35355	-3.20711	-1.77349
H	3.58602	-3.02303	1.28799
H	2.87299	-5.20743	0.28302
H	1.74437	0.23969	0.76737
H	2.72117	-0.61072	1.97848
H	2.77088	0.72313	-1.58607
H	4.55577	0.56869	-1.78313
H	3.52088	-0.88378	-1.74738
H	6.42464	-1.62567	3.38434
H	6.32264	-2.71185	1.93953
H	7.32174	-1.22906	1.86221
H	5.03461	3.93734	0.38241
H	5.49527	3.30338	1.99484
H	6.71851	3.34111	0.65951

II^{H}.T$_{aryl}$

79

BP86

SCF = -1968.23833179

H(0 K)= -1967.608008

H(413 K)= -1967.530476

G(413 K)= -1967.739114

B97D (1,2-C6Cl2H4)= -4530.00154771

Low Freq.= 15.8244cm-1,21.0165cm-1

79

II^{H}.T$_{aryl}$

C	1.29967	-2.27596	1.57479
C	0.41970	-1.42706	0.87190
C	-0.46560	-1.94953	-0.10491
C	-0.37313	-3.33397	-0.39171
C	0.51830	-4.19026	0.26613
C	1.33374	-3.63814	1.25757
Pd	0.57084	0.48734	1.38720
C	-3.15042	-0.99434	1.11312
C	-2.96785	-1.30303	-0.39000
C	-3.46357	-2.73798	-0.66942
O	-3.86084	-3.53703	0.16137
C	-1.48399	-1.11236	-0.85602
F	2.21849	-4.43775	1.91556
C	1.27929	1.12198	-0.37919
N	0.75133	2.23001	-1.01795
C	1.47499	2.61089	-2.14765
C	2.47259	1.68748	-2.28197
N	2.33820	0.80572	-1.20368
C	-0.17482	3.33935	-0.64199
C	-0.23697	4.03681	-2.03972
O	1.04546	3.77373	-2.70341
C	-1.54849	2.84375	-0.16182
C	-2.44932	4.00854	0.29795
C	-1.76926	4.85216	1.39166
C	-0.40364	5.37598	0.91110
C	0.50658	4.22745	0.43321
O	3.51620	1.39368	-3.10035
C	4.36138	0.51595	-2.29171
C	3.42654	-0.21535	-1.26408
C	2.90062	-1.56162	-1.81933
C	4.00751	-2.63695	-1.86385
C	4.70205	-2.80830	-0.49934
C	5.23515	-1.46180	0.02593
C	4.11701	-0.40121	0.10020
Br	-0.25300	0.36945	3.68100
C	-3.84975	-0.33653	-1.20846
O	-3.45745	0.61309	-1.87579
O	-5.16702	-0.65008	-1.06454
C	-6.08202	0.22273	-1.76893
O	-3.39684	-3.01582	-2.01113
C	-3.88863	-4.32869	-2.37098
H	3.35153	-0.71543	0.83069
H	4.51179	0.57159	0.44611
H	6.05162	-1.10313	-0.63351
H	5.68349	-1.58605	1.02732
H	3.98576	-3.22814	0.23065
H	5.52832	-3.53683	-0.58399
H	3.56476	-3.59238	-2.19582
H	4.76581	-2.37133	-2.62788
H	2.47329	-1.39666	-2.82556
H	2.07912	-1.90315	-1.16752
H	5.11002	1.13871	-1.76516
H	4.86950	-0.16986	-2.98371
H	-1.04614	3.60133	-2.65527
H	-0.35115	5.12828	-1.98398
H	1.46229	4.61680	0.03657
H	0.75461	3.57585	1.29385
H	0.10866	5.93185	1.71618

```
H  -0.55650  6.10289  0.08810
H  -1.62435  4.22690  2.29378
H  -2.41866  5.69489  1.68888
H  -2.70363  4.65485 -0.56671
H  -3.40613  3.59754  0.66431
H  -1.37435  2.15718  0.69362
H  -2.03590  2.24880 -0.95343
H   1.94535 -1.89934  2.37135
H  -1.01621 -3.74283 -1.17872
H   0.58081 -5.25521  0.02885
H  -1.24201 -0.04629 -0.73126
H  -1.43836 -1.33446 -1.93603
H  -2.81699  0.03078  1.33854
H  -4.20283 -1.11442  1.40886
H  -2.53938 -1.68412  1.71343
H  -3.30231 -5.11538 -1.86927
H  -4.94532 -4.43781 -2.07900
H  -3.77670 -4.39570 -3.46174
H  -5.99005  1.25739 -1.40192
H  -5.87436  0.20871 -2.85071
H  -7.08392 -0.17480 -1.55889
```

II^{H}.T$_{NHC}$

```
79
BP86
SCF  =           -1968.23920169
H(0 K)=          -1967.609264
H(413 K)=        -1967.532073
G(413 K)=        -1967.740576
B97D      (1,2-C6Cl2H4)=       -
4529.99420611
Low Freq.= 12.0606cm-1,16.2613cm-
1
```

```
79
```

II^{H}.T$_{NHC}$

```
C  -2.04204  0.25442  1.55603
C  -0.67640 -0.11399  1.37181
C   0.11480 -0.34376  2.51745
C  -0.43730 -0.23465  3.79882
C  -1.77280  0.11789  4.00289
C  -2.55897  0.35799  2.86746
Pd  0.02137 -0.36439 -0.52707
Br  0.72216 -0.63633 -2.96217
F   0.36036 -0.46895  4.87772
C  -2.98396  0.53816  0.39531
C  -3.44848 -0.74040 -0.37950
C  -4.59006 -0.45453 -1.37605
O  -5.37053 -1.30301 -1.78289
C   1.74365  0.42395 -0.03036
N   2.09866  1.73988  0.06594
C   3.42786  1.91266  0.47173
C   3.93810  0.65726  0.63427
N   2.89292 -0.22953  0.33825
C   1.43429  3.06719 -0.05298
C   2.73261  3.94216 -0.13170
O   3.77992  3.22079  0.59578
C   0.60276  3.36869  1.21869
C  -0.14858  4.71154  1.11290
C  -1.00964  4.78216 -0.16182
C  -0.15936  4.51636 -1.41812
C   0.57315  3.16312 -1.32544
O   5.10733  0.02765  0.92542
C   4.71703 -1.37084  1.13302
C   3.45526 -1.61441  0.24444
C   2.50137 -2.67854  0.81346
C   3.04522 -4.10928  0.62124
C   3.37117 -4.39497 -0.85568
C   4.36688 -3.35733 -1.40393
C   3.83914 -1.91982 -1.22571
C  -3.95639 -1.80308  0.61699
O  -3.44216 -2.89370  0.80812
C  -2.28667 -1.32721 -1.20159
H  -2.56331 -2.24218 -1.74761
O  -5.04592 -1.34370  1.29030
C  -5.58619 -2.27617  2.25869
O  -4.58572  0.83857 -1.80222
C  -5.58915  1.13735 -2.80585
H  -0.16356  2.33684 -1.29614
H   1.20128  2.97795 -2.21444
H   0.57491  5.33737 -1.54671
H  -0.78938  4.52464 -2.32481
H  -1.81366  4.02269 -0.10096
H  -1.50765  5.76563 -0.23450
H  -0.77206  4.84844  2.01401
H   0.57317  5.55302  1.11138
H   1.26885  3.35741  2.10064
H  -0.12065  2.54431  1.35170
H   3.05624  4.06554 -1.18277
H   2.62881  4.92582  0.34704
H   4.48061 -1.51489  2.20507
H   5.57898 -1.99030  0.84994
H   4.58951 -1.18093 -1.56179
H   2.93855 -1.76940 -1.84921
H   4.56414 -3.53418 -2.47584
H   5.34183 -3.47915 -0.88906
H   2.43795 -4.35415 -1.44972
H   3.77704 -5.41638 -0.96730
H   3.95760 -4.25177  1.23528
H   2.30102 -4.82929  1.00459
H   1.53289 -2.57400  0.28559
H   2.30531 -2.47462  1.88138
H   1.17134 -0.61040  2.43707
H  -3.61017  0.64133  2.99716
H  -2.17338  0.20594  5.01617
H  -3.87822  1.06170  0.76985
H  -2.50205  1.20525 -0.34180
H  -1.48189 -1.69446 -0.49928
H  -1.92519 -0.59673 -1.94637
H  -6.59849  0.94797 -2.40797
H  -5.45518  2.20093 -3.04383
H  -5.43560  0.51362 -3.70038
H  -6.46593 -1.77655  2.68628
H  -5.87371 -3.21752  1.76509
H  -4.84032 -2.49236  3.03986
```

C-H Cyclisation Reactions

R = Me (8a)

```
97
```

III^{Me}

BP86
SCF = -2340.55058092
H(0 K)= -2339.760683
H(413 K)= -2339.667334
G(413 K)= -2339.910358
B97D (1,2-C6Cl2H4)= -2340.13766298
Low Freq.= 11.5849cm-1,15.4310cm-1

97
III^{Me}

```
C    0.64916 -1.17434  2.45198
C   -0.12938 -0.64905  1.39646
C   -1.53303 -0.90952  1.37528
C   -2.09035 -1.67727  2.42480
C   -1.32180 -2.17789  3.48529
C    0.04828 -1.91033  3.47767
Pd   0.71294  0.55638  0.02832
O    1.19293  2.29504 -1.27707
C    0.16618  2.90220 -0.79242
O   -0.52513  2.32399  0.13139
C   -2.40171 -0.43408  0.21594
C   -3.72735  0.34002  0.54959
C   -4.82044 -0.62979  1.03776
O   -5.36981 -0.61926  2.13096
F    0.82781 -2.38928  4.48864
C    2.12312 -0.76980 -0.33531
N    2.13840 -1.93968 -1.05369
C    3.41787 -2.50001 -1.16171
C    4.25669 -1.65726 -0.49265
N    3.44798 -0.63706  0.01943
C    1.14715 -2.84109 -1.70212
C    2.15380 -3.67555 -2.56704
O    3.43484 -3.66842 -1.85916
C    0.43153 -3.71266 -0.63999
C   -0.62068 -4.64450 -1.27541
C   -1.62567 -3.86291 -2.14176
C   -0.90551 -3.00883 -3.20257
C    0.13376 -2.06507 -2.56265
O    5.58085 -1.47212 -0.24057
C    5.61107 -0.40620  0.76456
C    4.31270  0.44575  0.56715
C    3.76708  1.01676  1.88870
C    4.61532  2.19660  2.40502
C    4.74774  3.30263  1.34213
C    5.33215  2.73915  0.03404
C    4.49221  1.55980 -0.49646
C   -0.26466  4.26613 -1.34952
C    0.98689  5.12732 -1.63064
C   -1.01656  3.98337 -2.67974
C   -1.20506  4.98236 -0.35934
C   -4.20546  0.99211 -0.76875
O   -3.49148  1.32015 -1.70687
C   -3.45699  1.45270  1.61023
O   -5.55144  1.21127 -0.75064
C   -6.07367  1.86745 -1.93021
O   -5.09136 -1.57151  0.08099
C   -6.11852 -2.51942  0.44918
H    3.48673  1.91849 -0.78307
H    4.95449  1.12283 -1.40084
H    6.37879  2.41767  0.21028
H    5.38152  3.52408 -0.74112
H    3.74791  3.73215  1.13849
H    5.37786  4.12642  1.72278
H    4.15628  2.59440  3.32715
H    5.62495  1.83907  2.69265
H    3.71247  0.21193  2.64433
H    2.72732  1.35286  1.70090
H    6.53776  0.16209  0.60289
H    5.62535 -0.87164  1.76881
H    1.86070 -4.72728 -2.69067
H    2.29705 -3.20591 -3.55937
H    0.67275 -1.48421 -3.33297
H   -0.37237 -1.32888 -1.91021
H   -1.63347 -2.40925 -3.77662
H   -0.40784 -3.67486 -3.93656
H   -2.22767 -3.20018 -1.49094
H   -2.33496 -4.55730 -2.62713
H   -0.12180 -5.41374 -1.89871
H   -1.14424 -5.19662 -0.47508
H   -0.05541 -3.03284  0.08171
H    1.18538 -4.29501 -0.07946
H    1.72907 -1.01387  2.49460
H   -3.16580 -1.88065  2.43314
H   -1.76792 -2.76037  4.29570
H   -1.80839  0.24547 -0.41708
H   -2.67800 -1.29355 -0.42286
C   -4.59802  2.44210  1.89223
H   -3.19145  0.92859  2.54371
H   -2.55432  1.99250  1.27677
H   -6.23390 -3.18071 -0.42057
H   -5.81742 -3.09718  1.33815
H   -7.06389 -1.99902  0.67141
H   -5.60934  2.85775 -2.06142
H   -5.87629  1.26120 -2.82866
H   -7.15362  1.96530 -1.75571
H   -1.89571  3.34029 -2.50811
H   -1.35801  4.93574 -3.12329
H   -0.35438  3.48113 -3.40438
H    1.52817  5.36327 -0.69771
H    1.68037  4.60356 -2.30678
H    0.68966  6.08145 -2.10021
H   -0.70331  5.17691  0.60362
H   -1.52480  5.95126 -0.78192
H   -2.10156  4.37694 -0.15559
H   -4.29514  3.12584  2.70360
H   -4.84273  3.06248  1.01357
H   -5.51330  1.92044  2.21138
```

97
TS(III^{Me}-IV^{Me})1
BP86
SCF = -2340.52746081
H(0 K)= -2339.738560
H(413 K)= -2339.646078
G(413 K)= -2339.886411
B97D (1,2-C6Cl2H4)= -2340.12503583
Low Freq.=-31.7779cm-1,11.7675cm-1

97

TS(III^{Me}-IV^{Me})1

```
C   0.27401 -1.52660  2.55330
C  -0.47536 -1.03091  1.46487
C  -1.85174 -1.40194  1.37165
C  -2.42112 -2.21075  2.38042
C  -1.67974 -2.67456  3.47547
C  -0.33114 -2.31735  3.53683
Pd  0.26713  0.27176  0.11898
O   0.84480  1.68253 -1.39956
C   0.64129  2.85835 -0.87105
O   0.28208  3.01157  0.32709
C  -2.72997 -0.97908  0.20695
C  -3.48868  0.38006  0.38798
C  -4.49610  0.27590  1.54768
O  -4.50255  0.96080  2.55900
F   0.42795 -2.76570  4.57565
C   1.82940 -0.84649 -0.27340
N   1.94893 -1.93293 -1.10497
C   3.28178 -2.28834 -1.33715
C   4.03711 -1.44431 -0.57839
N   3.13784 -0.57512  0.05461
C   1.06762 -2.66966 -2.05548
C   2.08298 -3.78534 -2.48468
O   3.43268 -3.26368 -2.27045
C  -0.17063 -3.26715 -1.36483
C  -1.11367 -3.95393 -2.37382
C  -1.53314 -2.98748 -3.49746
C  -0.29755 -2.41278 -4.21414
C   0.66883 -1.73321 -3.22383
O   5.33718 -1.20173 -0.26444
C   5.31557  0.15630  0.27931
C   3.90532  0.36964  0.92731
C   3.87076 -0.10056  2.40255
C   4.64757  0.85006  3.33676
C   4.15591  2.30228  3.19510
C   4.22115  2.76571  1.72823
C   3.42226  1.82348  0.80399
C   0.83303  4.07133 -1.82093
C   0.77552  5.38764 -1.02268
C   2.18793  3.94905 -2.55578
C  -0.31626  4.03369 -2.86098
C  -4.21322  0.67276 -0.94111
O  -3.79623  0.35483 -2.04762
C  -2.46787  1.51714  0.67147
O  -5.35811  1.38202 -0.74761
C  -6.05689  1.74740 -1.96316
O  -5.39343 -0.73191  1.32016
C  -6.39095 -0.88299  2.35764
H   2.34987  1.86747  1.07153
H   3.48972  2.14188 -0.25069
H   5.28049  2.81675  1.40312
H   3.81751  3.78798  1.62791
H   3.10845  2.36921  3.54788
H   4.75181  2.97307  3.83953
H   4.53863  0.49890  4.37816
H   5.73195  0.80385  3.11220
H   4.25661 -1.13380  2.47352
H   2.81341 -0.11880  2.71891
H   5.47143  0.87153 -0.55083
H   6.14417  0.22892  0.99698
H   1.95583 -4.68951 -1.85968
H   2.00615 -4.05611 -3.54756
H   1.58331 -1.38486 -3.73806
H   0.19087 -0.83868 -2.78323
H  -0.59846 -1.68008 -4.98344
H   0.22420 -3.22818 -4.75548
H  -2.12575 -2.15745 -3.06707
H  -2.18917 -3.50371 -4.22116
H  -0.61701 -4.84184 -2.81582
H  -1.99965 -4.33514 -1.83618
H  -0.70255 -2.44565 -0.85825
H   0.15128 -3.96786 -0.57353
H   1.33928 -1.31257  2.65646
H  -3.47610 -2.49588  2.29027
H  -2.12086 -3.30577  4.25130
H  -2.12221 -0.85359 -0.70681
H  -3.47816 -1.76202 -0.00299
C  -2.97683  2.96239  0.59825
H  -2.04106  1.33221  1.67048
H  -1.64624  1.40198 -0.08032
H  -5.91381 -1.10119  3.32632
H  -6.98653  0.03861  2.45656
H  -7.02375 -1.72156  2.03623
H  -5.41683  2.37556 -2.60269
H  -6.34832  0.84651 -2.52589
H  -6.94346  2.30488 -1.63285
H   2.30999  4.77833 -3.27584
H   3.03298  3.99818 -1.84569
H   2.25220  2.99510 -3.10224
H   0.88446  6.25041 -1.70431
H  -0.17969  5.48379 -0.48329
H   1.58271  5.43616 -0.27285
H  -0.22113  4.87915 -3.56610
H  -0.29457  3.09359 -3.43562
H  -1.30008  4.11338 -2.36659
H  -2.13974  3.64042  0.82248
H  -3.36279  3.21681 -0.40347
H  -3.78080  3.12221  1.33347
```

97

Int(III^{Me}-IV^{Me})

```
BP86
SCF  =          -2340.53405325
H(0 K)=         -2339.744852
H(413 K)=       -2339.651592
G(413 K)=       -2339.893454
B97D      (1,2-C6Cl2H4)=       -
2340.12977199
Low Freq.=14.0274cm-1, 18.5526cm-
1
```

97

Int(III^{Me}-IV^{Me})

```
C   0.83927 -0.40406  3.28836
C   1.66725 -1.53404  2.64928
C   0.83465 -2.82894  2.47954
C   0.15956 -3.25918  3.79742
C  -0.68077 -2.11830  4.39996
C   0.17431 -0.85415  4.60419
C   3.01675 -1.79173  3.40650
O   4.02608 -2.12808  2.39834
```

C	3.58794	-1.51203	1.26817
N	2.24643	-1.11756	1.34112
C	1.80347	-0.53192	0.18685
N	2.91427	-0.57887	-0.61742
C	4.01991	-1.15074	0.02398
O	5.15160	-1.11669	-0.73011
C	4.67769	-0.77689	-2.07562
C	3.35875	0.04586	-1.89758
C	3.63255	1.55446	-1.66896
C	4.03138	2.27725	-2.97127
C	2.98777	2.05717	-4.08122
C	2.76401	0.55475	-4.33110
C	2.34971	-0.17685	-3.03831
Pd	0.05165	0.23019	-0.26344
O	0.80002	2.22570	-0.01525
C	0.32016	2.82494	1.04514
C	0.84947	4.26382	1.32811
C	1.81590	4.76527	0.23948
C	-0.69662	-1.61138	-0.63799
C	0.04116	-2.61799	-1.29922
C	-0.55104	-3.84590	-1.61498
C	-1.87522	-4.13770	-1.28223
C	-2.60933	-3.14352	-0.62013
C	-2.05184	-1.88798	-0.28872
C	-2.94059	-0.87871	0.42241
C	-3.42539	0.31027	-0.48211
C	-3.97121	-0.25925	-1.80692
O	-5.08710	-1.00531	-1.58450
C	-5.65065	-1.60881	-2.77425
F	0.19611	-4.78824	-2.25600
C	-2.23612	1.27258	-0.75479
H	-1.52340	0.67688	-1.40519
C	-4.54645	1.12362	0.19128
O	-4.45223	1.09000	1.54831
C	-5.42702	1.91155	2.23647
O	-5.38909	1.77183	-0.41485
O	-0.52194	2.32177	1.82695
C	-0.37582	5.20413	1.42483
C	1.56916	4.22538	2.69889
O	-3.45614	-0.11863	-2.90662
H	0.06728	-0.07731	2.56650
H	1.48466	0.47713	3.45176
H	0.94770	-1.05541	5.37369
H	-0.44124	-0.02522	4.99436
H	-1.51916	-1.88237	3.71599
H	-1.13143	-2.43760	5.35689
H	-0.46580	-4.14955	3.60836
H	0.92583	-3.57529	4.53398
H	1.48545	-3.63183	2.08718
H	0.06410	-2.63609	1.71218
H	3.34566	-0.87906	3.93889
H	2.96977	-2.63676	4.10765
H	4.48381	-1.71709	-2.62690
H	5.48282	-0.21307	-2.56687
H	4.42404	1.66137	-0.90396
H	2.71280	2.00333	-1.25043
H	4.15562	3.35406	-2.76062
H	5.01994	1.91899	-3.32424
H	2.02955	2.52189	-3.77859
H	3.30484	2.55760	-5.01370
H	3.69300	0.10614	-4.73799
H	1.98527	0.39766	-5.09791
H	1.36682	0.19983	-2.69134
H	2.22669	-1.25944	-3.22154
H	1.08623	-2.46695	-1.58060
H	-3.65141	-3.34502	-0.34474
H	-2.30702	-5.11072	-1.53096
H	-3.82998	-1.39513	0.81807
H	-2.41481	-0.42946	1.28325
C	-2.51428	2.56719	-1.53550
H	-1.80220	1.53645	0.23542
H	-6.44924	1.57543	2.00056
H	-5.20967	1.78651	3.30545
H	-5.32096	2.96620	1.93774
H	-4.93089	-2.30942	-3.22653
H	-6.55085	-2.13874	-2.43464
H	-5.91010	-0.83355	-3.51200
H	0.89088	3.83829	3.47599
H	2.46254	3.57637	2.66233
H	1.90014	5.23880	2.98920
H	-1.08809	4.82673	2.17516
H	-0.05842	6.22239	1.71316
H	-0.90147	5.27550	0.45622
H	1.33026	4.78253	-0.75007
H	2.15451	5.79084	0.47501
H	2.70599	4.11917	0.16557
H	-2.92186	2.34613	-2.53315
H	-3.23163	3.20780	-0.99859
H	-1.57284	3.12804	-1.65178

97

TS(III^{Me}-IV^{Me})2

BP86

SCF = -2340.50833328

H(0 K)= -2339.723377

H(413 K)= -2339.631240

G(413 K)= -2339.868484

B97D (1,2-C6Cl2H4)= -2340.09683792

Low Freq.=-834.111cm-1,15.1032cm-1

97

TS(III^{Me}-IV^{Me})2

C	-1.73339	-2.13666	-0.55490
C	-0.45829	-1.60329	-0.89611
C	0.39642	-2.37570	-1.71175
C	-0.01170	-3.63417	-2.17185
C	-1.25155	-4.18175	-1.83790
C	-2.10245	-3.41412	-1.02740
Pd	0.04737	0.24024	-0.25676
C	-2.06570	0.82519	-0.91641
C	-2.44520	2.22831	-1.44506
F	0.84162	-4.35106	-2.95893
C	-2.70552	-1.34697	0.29366
C	-3.25117	-0.06464	-0.42050
C	-4.15209	0.66507	0.59747
O	-4.11550	0.52302	1.81161
C	1.93885	-0.35656	0.13404
N	2.44158	-1.07492	1.19001
C	3.82887	-1.25358	1.13139
C	4.23956	-0.61321	-0.00210

```
N   3.07861 -0.09801 -0.59072
C   1.89239 -1.83315  2.34706
C   3.22972 -1.99214  3.14734
O   4.31765 -1.99222  2.16567
C   1.33181 -3.19941  1.87802
C   0.68863 -3.99090  3.03439
C  -0.38617 -3.15917  3.75784
C   0.19552 -1.82444  4.25935
C   0.82866 -1.01957  3.10675
O   5.37361 -0.29069 -0.68423
C   4.88470  0.18862 -1.98052
C   3.45621  0.77792 -1.73384
C   2.50980  0.60159 -2.93483
C   2.83260  1.58035 -4.08160
C   2.83487  3.04033 -3.59315
C   3.81955  3.22416 -2.42412
C   3.50972  2.25515 -1.26545
O   0.59787  2.25307  0.49461
C  -0.31381  2.74318  1.22933
O  -1.49202  2.20483  1.32904
C  -0.06819  4.01723  2.05829
C  -4.11602 -0.51291 -1.62471
O  -3.86630 -0.32366 -2.80591
O  -5.20990 -1.21741 -1.19997
C  -6.06430 -1.69339 -2.26513
O  -5.01323  1.52237 -0.02649
C  -5.88099  2.25480  0.86899
H   0.04802 -0.72972  2.37581
H   1.28018 -0.08035  3.47554
H   0.95432 -2.02392  5.04339
H  -0.58991 -1.21354  4.73776
H  -1.21828 -2.95143  3.05786
H  -0.81420 -3.73155  4.60026
H   0.25580 -4.92471  2.63444
H   1.46319 -4.30159  3.76472
H   2.14427 -3.78053  1.40466
H   0.57755 -3.00306  1.09591
H   3.37816 -1.13784  3.83594
H   3.29879 -2.93720  3.70394
H   4.83974 -0.66938 -2.67874
H   5.61031  0.92981 -2.34377
H   4.26526  2.34835 -0.46352
H   2.53137  2.50091 -0.81602
H   3.78875  4.26174 -2.04655
H   4.85377  3.06478 -2.79114
H   1.81495  3.31294 -3.25876
H   3.09060  3.72250 -4.42371
H   3.82248  1.33728 -4.51842
H   2.09624  1.44130 -4.89251
H   1.47644  0.77280 -2.57074
H   2.55024 -0.44459 -3.28812
H   1.38925 -2.01973 -1.99795
H  -3.08372 -3.81833 -0.75036
H  -1.53489 -5.17193 -2.20453
H  -3.56020 -1.97685  0.59088
H  -2.22287 -0.99438  1.22135
H  -1.71593  0.29173 -1.81914
H  -1.55243  1.40319  0.44247
H  -6.49654  2.89483  0.22214
H  -6.51355  1.56240  1.44755
H  -5.28903  2.86326  1.57108
H  -6.88024 -2.23575 -1.76761
H  -6.46012 -0.84815 -2.85105
H  -5.50674 -2.36118 -2.94114
H  -2.85316  2.89375 -0.66770
H  -1.54527  2.71415 -1.86165
H  -3.19001  2.15139 -2.25622
C  -0.28522  3.66161  3.55189
C   1.36190  4.54495  1.83890
C  -1.10655  5.08373  1.62694
H  -0.15390  4.56503  4.17265
H  -1.29780  3.26234  3.71864
H   0.44536  2.90739  3.89247
H   1.52312  5.44909  2.45133
H   2.11541  3.79350  2.12486
H   1.53426  4.80900  0.78289
H  -0.97386  5.99887  2.22995
H  -0.98336  5.35616  0.56459
H  -2.13305  4.71286  1.77215
```

97

IV^{Me}

```
BP86
SCF  =           -2340.52141006
H(0 K)=          -2339.732319
H(413 K)=        -2339.639577
G(413 K)=        -2339.878699
B97D      (1,2-C6Cl2H4)=       -
2340.11265922
Low Freq.=9.5331cm-1, 17.6826cm-1
```

97

IV^{Me}

```
C   0.39624  2.15088  2.10688
C  -0.56917  1.50393  1.30184
C  -1.89266  2.02294  1.32325
C  -2.22371  3.10901  2.15782
C  -1.26518  3.72871  2.97423
C   0.03854  3.23221  2.92119
Pd -0.10222 -0.16190  0.27875
C  -1.94689 -0.91362  0.98833
C  -3.21120 -0.08503  0.57007
C  -3.72914 -0.51574 -0.80072
O  -3.10418 -1.10591 -1.68879
C  -2.93529  1.45534  0.39280
F   1.00510  3.81947  3.68881
C   1.76097  0.57742 -0.32269
N   3.00583  0.42638  0.25588
C   4.02135  1.14080 -0.39394
C   3.41027  1.80750 -1.41498
N   2.06169  1.44075 -1.35088
C   3.62366 -0.48102  1.26203
C   4.98747  0.26933  1.41503
O   5.25977  0.91714  0.13043
C   2.82202 -0.54866  2.57423
C   3.40460 -1.57873  3.56346
C   3.52569 -2.97451  2.92625
C   4.36814 -2.91414  1.63946
C   3.79526 -1.88934  0.64125
O   3.67895  2.69752 -2.41239
C   2.48768  2.64877 -3.26262
C   1.28886  2.22190 -2.34865
```

C 0.60883 3.43658 -1.66838
C -0.26146 4.24322 -2.65251
C -1.29188 3.34668 -3.36356
C -0.60299 2.16132 -4.06532
C 0.25571 1.34484 -3.07916
O 0.45856 -2.20848 -0.58699
C -0.05931 -2.98824 -1.41119
O -1.20230 -2.77277 -2.04835
C 0.56601 -4.34333 -1.79953
C -0.41680 -5.46074 -1.35793
C 0.74302 -4.38778 -3.33924
C 1.92565 -4.53855 -1.10833
C -4.34940 -0.27158 1.59460
O -4.74809 0.55971 2.39561
O -4.84208 -1.54964 1.53196
C -5.88219 -1.83286 2.49532
O -4.98845 -0.07068 -1.02168
C -5.53910 -0.36398 -2.32942
H -0.38723 0.88441 -2.30107
H 0.77568 0.51613 -3.59414
H 0.02994 2.54206 -4.89281
H -1.35142 1.49703 -4.53294
H -2.01042 2.95704 -2.61599
H -1.87898 3.93609 -4.09094
H -0.76669 5.05883 -2.10569
H 0.37745 4.73580 -3.41349
H 1.38450 4.07764 -1.21131
H -0.01391 3.04842 -0.84207
H 2.65546 1.89988 -4.06119
H 2.36876 3.64686 -3.70771
H 4.91718 1.05026 2.19685
H 5.83645 -0.39612 1.62699
H 4.43803 -1.81087 -0.25535
H 2.79636 -2.21411 0.30139
H 4.41533 -3.90616 1.15535
H 5.41373 -2.65253 1.89959
H 2.51285 -3.35098 2.68272
H 3.96702 -3.68896 3.64439
H 4.40450 -1.24627 3.90920
H 2.76575 -1.61491 4.46345
H 1.77796 -0.81534 2.31430
H 2.78470 0.45200 3.03930
H 1.43983 1.83030 2.11329
H -3.25717 3.47383 2.17042
H -1.51257 4.56984 3.62723
H -3.88972 1.99810 0.48731
H -2.57210 1.59536 -0.64406
C -1.84012 -1.14757 2.51070
H -2.05097 -1.90881 0.51374
H -1.72586 -1.96402 -1.72431
H -5.55723 -1.45087 -2.50492
H -6.55789 0.04525 -2.31143
H -4.94008 0.11750 -3.11840
H -6.74114 -1.15756 2.35088
H -6.17404 -2.87675 2.31530
H -5.50328 -1.70742 3.52245
H -0.22019 -4.25394 -3.85489
H 1.43477 -3.59995 -3.68460
H 1.16698 -5.36331 -3.63422
H -1.39779 -5.34047 -1.84305
H -0.00516 -6.44639 -1.63628
H -0.56615 -5.45205 -0.26437
H 1.82928 -4.49188 -0.01163
H 2.33778 -5.52628 -1.37741
H 2.64898 -3.76854 -1.42086
H -0.92112 -1.71753 2.73273
H -1.78438 -0.20208 3.07385
H -2.68858 -1.73696 2.91569

96
V^{Me}.OPiv
BP86
SCF = -2339.98673761
H(0 K)= -2339.211662
H(413 K)= -2339.118913
G(413 K)= -2339.360590
B97D (1,2-C6Cl2H4)= -2339.63050674
Low Freq.=5.0613cm-1, 11.9095cm-1

96
V^{Me}.OPiv
C -0.57659 -2.74443 -0.64198
C -1.14680 -1.44718 -0.72885
C -2.34438 -1.33664 -1.50316
C -2.90793 -2.46553 -2.13326
C -2.33006 -3.74221 -2.03099
C -1.16552 -3.84698 -1.26881
Pd -0.25015 0.18628 0.04419
C -2.10296 1.15549 0.42208
C -3.40116 0.56812 -0.21030
C -4.07279 -0.55040 0.60459
O -3.68113 -1.09667 1.62342
C -3.06663 -0.01049 -1.62000
F -0.56846 -5.07901 -1.14166
C 1.63839 -0.62686 -0.12615
N 2.47522 -0.73108 -1.21320
C 3.74308 -1.23645 -0.90508
C 3.69999 -1.55505 0.41851
N 2.41952 -1.19311 0.85388
C 2.46982 -0.19082 -2.58909
C 3.74687 -0.92953 -3.11860
O 4.60524 -1.20830 -1.96783
C 1.19439 -0.58674 -3.35678
C 1.14550 0.04117 -4.76350
C 1.28221 1.57414 -4.69809
C 2.57058 1.97831 -3.95722
C 2.63435 1.34916 -2.55230
O 4.47749 -2.06969 1.41967
C 3.81450 -1.60297 2.63978
C 2.29037 -1.44037 2.30894
C 1.48653 -2.73949 2.57017
C 1.27939 -3.00989 4.07419
C 0.63641 -1.80115 4.77977
C 1.46454 -0.52390 4.54289
C 1.65023 -0.24584 3.03925
O 0.51620 2.16617 0.60800
C 1.72270 2.58323 0.81944
O 2.78495 1.91203 0.83513
C 1.85192 4.13048 1.09978
C 2.84520 4.71082 0.06709
C 0.50275 4.86433 1.00751

C   2.44999 4.29198 2.51697
C -4.38191 1.73073 -0.39604
O -4.49525 2.43174 -1.39678
O -5.10503 1.97915 0.75151
C -5.96395 3.13110 0.66611
O -5.25046 -0.92742 -0.01451
C -5.91561 -2.03360 0.62024
H   0.66495 -0.06309 2.56458
H   2.25364 0.65618 2.84355
H   2.45195 -0.62880 5.04061
H   0.97405 0.34616 5.01548
H -0.38287 -1.64746 4.37610
H   0.52688 -2.00112 5.86250
H   0.65510 -3.91337 4.20097
H   2.25213 -3.23888 4.55716
H   1.99456 -3.59183 2.08150
H   0.50245 -2.62026 2.08168
H   4.24742 -0.62515 2.92490
H   4.01984 -2.34965 3.42160
H   3.46605 -1.89179 -3.59156
H   4.33929 -0.32945 -3.82582
H   3.57381 1.60595 -2.03328
H   1.81577 1.74193 -1.92266
H   2.64018 3.07791 -3.86910
H   3.44901 1.66402 -4.55937
H   0.40943 1.98968 -4.15811
H   1.26630 2.00841 -5.71554
H   1.95923 -0.37186 -5.39639
H   0.19816 -0.24283 -5.25636
H   0.33187 -0.24253 -2.75420
H   1.11768 -1.68784 -3.40406
H   0.35308 -2.91506 -0.09271
H -3.82749 -2.34228 -2.72049
H -2.76111 -4.62261 -2.51696
H -2.44541 0.74964 -2.12681
H -3.99397 -0.12635 -2.20651
H -1.90761 2.08631 -0.15042
H -5.28526 -2.93797 0.58662
H -6.14610 -1.80587 1.67475
H -6.84245 -2.19108 0.04827
H -5.37898 4.04665 0.47618
H -6.70027 3.01550 -0.14739
H -6.47158 3.19485 1.63987
H   0.63524 5.94587 1.20811
H   0.05559 4.74777 0.00675
H -0.22070 4.46027 1.73378
H   3.06066 5.77606 0.27768
H   3.78744 4.14016 0.09416
H   2.43398 4.64647 -0.95682
H   2.65572 5.35596 2.74309
H   1.75314 3.91190 3.28590
H   3.39017 3.72163 2.59318
C -2.19371 1.51744 1.91425
H -3.05737 2.17671 2.14490
H -2.29275 0.61958 2.54419
H -1.27526 2.05716 2.19714

80
V^Me^
BP86
SCF =   -1993.46727408
H(0 K)=   -1992.823453
H(413 K)=   -1992.747341
G(413 K)=   -1992.951918
B97D   (1,2-C6Cl2H4)=   -1993.13837993
Low Freq.=8.8938cm-1, 14.7147cm-1

80
V^Me^
C -2.10697 1.07550 1.26487
C -0.86069 0.39888 1.19128
C -0.06550 0.29514 2.35305
C -0.51050 0.85606 3.55640
C -1.72309 1.54330 3.65226
C -2.51030 1.63979 2.49454
Pd -0.11950 -0.27858 -0.53620
C -2.00380 -0.81735 -1.19861
C -1.78955 -0.43583 -2.67016
F   0.27474 0.73494 4.66609
C -3.03107 1.15799 0.06616
C -3.28239 -0.25880 -0.53331
C -4.42697 -0.27309 -1.56309
O -5.05580 -1.27149 -1.89129
C   1.94545 -0.00086 -0.35489
N   2.73710 1.11548 -0.51006
C   4.10608 0.86489 -0.40020
C   4.21845 -0.48325 -0.19427
N   2.90968 -0.96566 -0.15406
C   2.50021 2.57782 -0.59279
C   3.94809 3.00701 -1.01208
O   4.86842 1.98948 -0.49270
C   2.10004 3.12722 0.80022
C   1.76090 4.63040 0.75966
C   0.68068 4.93607 -0.29396
C   1.10804 4.42580 -1.68281
C   1.43510 2.91948 -1.65171
O   5.15639 -1.46260 -0.06824
C   4.40353 -2.62423 0.41761
C   2.91603 -2.44489 -0.04582
C   1.89552 -2.94195 0.99621
C   1.82062 -4.48050 1.05083
C   1.51672 -5.07778 -0.33559
C   2.55412 -4.61418 -1.37476
C   2.64319 -3.07666 -1.43506
C -3.73704 -1.22153 0.59340
O -3.23599 -2.29578 0.88321
O -4.81818 -0.69853 1.24310
C -5.32613 -1.52429 2.31571
O -4.63247 0.95489 -2.13033
C -5.65756 0.96959 -3.15136
H   0.52610 2.33729 -1.39664
H   1.77518 2.56341 -2.64182
H   1.99270 4.99798 -2.02957
H   0.31292 4.61015 -2.42692
H -0.26449 4.44038 0.00058
H   0.47451 6.02086 -0.33039
H   1.43053 4.95417 1.76233
H   2.67120 5.22144 0.53171
H   2.91712 2.92597 1.51726
H   1.21995 2.55342 1.14338
H   4.04475 3.03435 -2.11453

```
H   4.26206  3.97082 -0.58678
H   4.46666 -2.64373  1.52230
H   4.88992 -3.51841  0.00220
H   3.42372 -2.74863 -2.14612
H   1.68080 -2.66540 -1.79544
H   2.30609 -5.00465 -2.37777
H   3.54557 -5.03947 -1.11874
H   0.50729 -4.75406 -0.65559
H   1.49404 -6.18091 -0.28259
H   2.77785 -4.89434  1.42886
H   1.04621 -4.77846  1.77884
H   0.90611 -2.52365  0.71764
H   2.14961 -2.52134  1.98619
H   0.90184 -0.21344  2.33732
H  -3.47092  2.16604  2.54644
H  -2.03735  1.97670  4.60548
H  -3.98798  1.62274  0.35336
H  -2.59085  1.78144 -0.73321
H  -2.01153 -1.91542 -1.07586
H  -6.62724  0.65799 -2.73135
H  -5.70303  2.00849 -3.50612
H  -5.39619  0.28652 -3.97553
H  -6.20611 -0.99282  2.70374
H  -5.60727 -2.51995  1.93742
H  -4.56562 -1.64215  3.10418
H  -1.99589  0.62900 -2.87117
H  -0.73730 -0.63133 -2.98467
H  -2.41052 -1.04563 -3.35967
```

80

TS(V^{Me}-VI^{Me})

BP86
SCF = -1993.45155254
H(0 K)= -1992.809508
H(0 K)= -1992.808252
H(413 K)= -1992.732979
G(413 K)= -1992.936168
B97D (1,2-C6Cl2H4)= -1993.11799967
Low Freq.=-311.609cm-1, 12.030cm-1

80

TS(V^{Me}-VI^{Me})

```
C  -1.39470  2.49568  1.83199
C  -2.61350  2.26673  0.91807
C  -2.49627  3.10568 -0.37934
C  -2.25013  4.59967 -0.08927
C  -1.02664  4.80323  0.82344
C  -1.16908  3.99243  2.12514
C  -3.97592  2.48541  1.66431
O  -4.92099  1.48610  1.15839
C  -4.13164  0.47389  0.70323
N  -2.78653  0.83285  0.58085
C  -1.99127 -0.15230  0.03915
N  -2.92052 -1.15246 -0.15063
C  -4.21211 -0.81602  0.25948
O  -5.09826 -1.84521  0.14123
C  -4.40868 -2.82295 -0.70466
C  -2.86727 -2.58039 -0.54312
C  -2.24115 -3.41898  0.60020
C  -2.07903 -4.90353  0.21931
C  -1.28544 -5.06460 -1.09033
C  -1.93937 -4.26376 -2.23189
C  -2.09149 -2.77582 -1.86004
Pd  0.01487 -0.19795 -0.36554
C   2.10647 -0.73695 -0.70848
C   3.01396 -0.47579  0.54135
C   4.51102 -0.49414  0.15777
O   4.94269 -1.77663 -0.03737
C   6.33259 -1.88732 -0.42716
C   1.39859  1.18978 -0.91686
C   1.09389  1.86172 -2.12500
C   1.56374  3.16907 -2.30398
C   2.36184  3.82292 -1.35714
C   2.71936  3.11780 -0.19378
C   2.25493  1.81174  0.02605
C   2.68660  0.92856  1.16737
F   1.22918  3.83049 -3.44931
C   2.78974 -1.57538  1.60373
O   3.54745 -1.30594  2.70725
C   3.41829 -2.27036  3.77792
O   2.04510 -2.54223  1.52568
O   5.22974  0.48350  0.02194
H  -0.51274  2.07392  1.31193
H  -1.52480  1.91811  2.76579
H  -2.01427  4.39367  2.72156
H  -0.26824  4.10809  2.75358
H  -0.11372  4.47551  0.28998
H  -0.89506  5.87616  1.05299
H  -2.11677  5.13774 -1.04447
H  -3.14499  5.04588  0.39072
H  -3.40839  2.96430 -0.98822
H  -1.64816  2.69360 -0.95678
H  -3.85284  2.32910  2.75324
H  -4.42340  3.47282  1.48057
H  -4.71982 -2.65868 -1.75425
H  -4.73346 -3.82014 -0.37390
H  -2.85690 -3.30692  1.51164
H  -1.24966 -2.98114  0.82245
H  -1.57931 -5.43875  1.04600
H  -3.07495 -5.37781  0.10451
H  -0.25171 -4.69952 -0.93525
H  -1.20882 -6.13208 -1.36488
H  -2.93271 -4.69905 -2.46490
H  -1.34308 -4.34888 -3.15774
H  -1.09203 -2.31196 -1.72220
H  -2.59071 -2.20844 -2.66676
H   0.48290  1.40134 -2.90606
H   3.39426  3.58284  0.53363
H   2.72261  4.83571 -1.55400
H   3.55998  1.32544  1.70678
H   1.86122  0.79712  1.89228
C   2.78394 -0.86559 -2.07717
H   1.56285 -1.69326 -0.51105
H   2.37983 -2.30666  4.14429
H   3.70812 -3.27502  3.43077
H   4.09615 -1.92157  4.56885
H   6.50566 -1.37877 -1.38905
H   6.98549 -1.43451  0.33587
H   6.52617 -2.96473 -0.51729
H   3.41496 -1.77252 -2.11504
```

H 2.01503 -0.95853 -2.86148
H 3.41052 0.00698 -2.32567

80
VI^{Me}
BP86
SCF = -1993.50493961
H(0 K)= -1992.859247
H(413 K)= -1992.783641
G(413 K)= -1992.986196
B97D (1,2-C6Cl2H4) = -1993.17386657
Low Freq. = 10.8743cm-1, 19.8459cm-1

80
VI^{Me}
C -1.97841 2.44727 0.43657
C -1.86100 1.33274 1.33205
C -0.96455 1.39773 2.44423
C -0.22670 2.61121 2.59609
C -0.37146 3.70993 1.75193
C -1.26609 3.62470 0.65430
Pd 0.14044 0.35020 0.87238
C -3.05978 0.40939 1.12447
C -2.93951 -1.02909 1.62801
F 0.61114 2.70781 3.66512
C -3.08223 2.18224 -0.56959
C -3.39320 0.65423 -0.39421
C -2.56237 -0.25678 -1.31999
O -2.59605 -1.48196 -1.26413
C 1.67140 -0.62334 -0.03048
N 2.73535 -0.07483 -0.72191
C 3.70581 -1.00001 -1.10762
C 3.24199 -2.21577 -0.68915
N 2.03350 -1.95606 -0.03945
C 3.22328 1.30103 -0.98259
C 4.36504 0.94217 -1.99415
O 4.79096 -0.42990 -1.70372
C 3.75935 1.92259 0.33188
C 4.21252 3.38411 0.14911
C 3.09433 4.24687 -0.46416
C 2.59726 3.64320 -1.79080
C 2.12754 2.18729 -1.60252
O 3.55970 -3.54007 -0.73124
C 2.66794 -4.15215 0.25817
C 1.40254 -3.23377 0.37425
C 0.85338 -3.15402 1.81047
C 0.11853 -4.44498 2.22277
C -0.99851 -4.80396 1.22485
C -0.44484 -4.91715 -0.20732
C 0.29183 -3.63061 -0.63042
C -4.88238 0.38643 -0.67319
O -5.76021 0.29355 0.17270
O -5.11757 0.32523 -2.01382
C -6.50368 0.10785 -2.37327
O -1.84362 0.43341 -2.24420
C -1.02329 -0.38998 -3.10450
H 1.25407 2.14901 -0.91874
H 1.80045 1.74623 -2.56205
H 3.41088 3.68726 -2.54386
H 1.76610 4.24338 -2.20207
H 2.24600 4.29766 0.24529
H 3.44771 5.28230 -0.61965
H 4.52909 3.79244 1.12548
H 5.10786 3.42680 -0.50417
H 4.58346 1.29678 0.72134
H 2.93393 1.87099 1.06701
H 3.98921 0.97861 -3.03506
H 5.25432 1.58203 -1.89949
H 3.20295 -4.20360 1.22604
H 2.44304 -5.16813 -0.09679
H 0.73998 -3.74725 -1.63498
H -0.42884 -2.79459 -0.69033
H -1.26024 -5.11944 -0.92388
H 0.24039 -5.78734 -0.26679
H -1.77649 -4.01744 1.24993
H -1.48993 -5.74784 1.52270
H 0.83928 -5.28603 2.28559
H -0.29457 -4.32020 3.23966
H 0.16610 -2.28392 1.84685
H 1.68164 -2.92509 2.50602
H -1.01450 0.70035 3.28769
H -1.39479 4.48922 -0.00615
H 0.20335 4.61804 1.95289
H -3.98585 2.76483 -0.29953
H -2.82132 2.43363 -1.60755
H -3.92117 0.86785 1.65513
H -1.61066 -1.22511 -3.51653
H -0.67228 0.27834 -3.90283
H -0.17136 -0.78420 -2.52627
H -6.51987 0.08174 -3.47107
H -6.86482 -0.84501 -1.95512
H -7.13568 0.92594 -1.99249
H -2.07300 -1.53656 1.17717
H -2.80768 -1.02264 2.72457
H -3.85323 -1.60049 1.40005

97
TS(IV^{Me}-VI^{Me}).HOPiv
BP86
SCF = -2340.49124639
H(0 K)= -2339.705148
H(413 K)= -2339.611962
G(413 K)= -2339.855962
B97D (1,2-C6Cl2H4)= -2340.08155398
Low Freq.=-277.690cm-1, 9.3055cm-1

97
TS(IV^{Me}-VI^{Me}).HOPiv
C -0.91196 -2.61634 -1.81030
C -1.35833 -1.65857 -0.86909
C -2.35204 -2.02109 0.07100
C -2.80782 -3.34733 0.14122
C -2.30824 -4.32831 -0.73307
C -1.38260 -3.93190 -1.70517
Pd 0.01939 -0.18310 -0.55103
C -2.00225 0.30536 -1.24945
C -3.21498 0.24255 -0.26160
C -3.42517 1.60115 0.44577

O	-2.75464	2.61439	0.31357
C	-2.96237	-0.86568	0.81929
F	-0.91208	-4.86218	-2.58421
C	1.97875	-0.50741	-0.06672
N	3.11254	-0.14539	-0.76359
C	4.29772	-0.63907	-0.21393
C	3.92290	-1.36708	0.87960
N	2.53240	-1.25221	0.95474
C	3.43111	0.75848	-1.89959
C	4.89138	0.25853	-2.16611
O	5.41148	-0.23964	-0.88900
C	2.48591	0.52286	-3.09183
C	2.73666	1.51880	-4.24130
C	2.65263	2.97602	-3.75052
C	3.63450	3.22117	-2.59030
C	3.39432	2.23394	-1.43073
O	4.45124	-2.17435	1.84075
C	3.35333	-2.36111	2.79364
C	2.01980	-2.18273	1.99381
C	1.54717	-3.50287	1.33285
C	0.95830	-4.48991	2.36068
C	-0.16640	-3.83977	3.18731
C	0.32171	-2.54529	3.86452
C	0.89402	-1.55326	2.83197
O	0.67859	3.27881	0.21901
C	0.42302	3.10276	1.40819
O	0.18857	1.87953	1.94545
C	0.34468	4.22919	2.45822
C	-1.05539	4.20057	3.12065
C	1.43696	3.98799	3.53117
C	0.57468	5.58198	1.75819
C	-4.53865	-0.09426	-0.98960
O	-5.12125	-1.16671	-0.96960
O	-4.99553	0.99387	-1.67927
C	-6.22923	0.76989	-2.40248
O	-4.49081	1.50989	1.29471
C	-4.80971	2.72740	2.00778
H	0.09855	-1.24777	2.12396
H	1.26165	-0.63131	3.31683
H	1.09380	-2.79077	4.62219
H	-0.50352	-2.06007	4.41559
H	-1.01276	-3.60161	2.51474
H	-0.54636	-4.54956	3.94423
H	0.58287	-5.38244	1.82998
H	1.75469	-4.85102	3.04277
H	2.39362	-3.95943	0.78750
H	0.77787	-3.24176	0.58337
H	3.44018	-1.59087	3.58417
H	3.47481	-3.36152	3.23225
H	4.89466	-0.57532	-2.89464
H	5.57281	1.05233	-2.50340
H	4.14772	2.37439	-0.63331
H	2.40443	2.42693	-0.97959
H	3.53632	4.25318	-2.21030
H	4.67396	3.12931	-2.96662
H	1.62227	3.18516	-3.40398
H	2.85770	3.67283	-4.58319
H	3.73610	1.33671	-4.68656
H	2.00434	1.33570	-5.04774
H	1.44837	0.63311	-2.71196
H	2.58526	-0.52255	-3.43782
H	-0.18329	-2.37329	-2.58836
H	-3.59458	-3.61151	0.85645
H	-2.65860	-5.36311	-0.70284
H	-3.90032	-1.13134	1.32990
H	-2.26048	-0.45729	1.57118
C	-2.28597	0.23653	-2.75148
H	-1.48090	1.27583	-1.03886
H	0.21534	1.20690	1.18812
H	-4.96408	3.55949	1.30263
H	-5.73304	2.50853	2.56136
H	-3.99737	2.99324	2.70279
H	-7.03113	0.46212	-1.71273
H	-6.46896	1.73135	-2.87628
H	-6.09453	-0.01597	-3.16280
H	1.29430	3.01816	4.03372
H	2.44645	4.00180	3.08466
H	1.39373	4.78541	4.29381
H	-1.21497	3.25527	3.66379
H	-1.14667	5.03593	3.83724
H	-1.84840	4.29995	2.36088
H	-0.19110	5.76485	0.98757
H	0.53068	6.39946	2.49868
H	1.55822	5.61147	1.26249
H	-1.33380	0.24702	-3.30723
H	-2.82477	-0.68412	-3.03416
H	-2.88099	1.10580	-3.08393

97
VI^{Me}.HOPiv
BP86
SCF = -2340.55259326
H(0 K)= -2339.764055
H(413 K)= -2339.670419
G(413 K)= -2339.917252
B97D (1,2-C6Cl2H4)= -2340.13733428
Low Freq.=10.5826cm-1, 14.1264cm-1

97
VI^{Me}.HOPiv

C	-0.75516	2.17460	0.48238
C	-1.76428	1.42106	-0.19318
C	-2.15472	1.77664	-1.52252
C	-1.57615	2.86191	-2.17749
C	-0.56565	3.61590	-1.53253
C	-0.17596	3.25382	-0.24636
Pd	0.28762	0.39986	-0.37460
C	-2.75020	0.41330	0.41010
C	-3.95274	0.43091	-0.62208
C	-4.57772	-0.96071	-0.77758
O	-4.21865	-1.98842	-0.21840
C	-3.31888	0.92093	-1.97038
F	0.77758	3.99999	0.38277
C	1.75149	-0.88398	-0.86427
N	2.93564	-0.59977	-1.51288
C	3.77486	-1.70276	-1.67401
C	3.09588	-2.76319	-1.14210
N	1.89859	-2.23789	-0.64962
C	3.63851	0.63686	-1.93258
C	4.78102	-0.03453	-2.77005

```
O   4.97351 -1.38874 -2.24123
C   2.72538  1.54529 -2.77783
C   3.39284  2.89112 -3.12200
C   3.86744  3.62320 -1.85258
C   4.82519  2.73395 -1.03866
C   4.17222  1.38408 -0.68405
O   3.19407 -4.11358 -0.98769
C   2.13225 -4.44289 -0.03239
C   1.02911 -3.33807 -0.16675
C  -0.03360 -3.68282 -1.24055
C  -1.00186 -4.78664 -0.77009
C  -1.65973 -4.42959  0.57596
C  -0.59658 -4.11010  1.64379
C   0.35752 -2.99377  1.17461
O   0.08582  1.04169  3.45778
C   1.25474  0.65228  3.46302
O   1.91487  0.22741  2.36546
C   2.13829  0.60725  4.72910
C   2.67106 -0.83307  4.93068
C   3.33036  1.57804  4.52844
C   1.29838  1.04131  5.94519
C  -5.03173  1.44066 -0.18770
O  -5.10543  2.60289 -0.55757
O  -5.89455  0.87729  0.70621
C  -6.91113  1.77752  1.20904
O  -5.59274 -0.91928 -1.68716
C  -6.24809 -2.18861 -1.91921
H  -0.20285 -2.04891  1.02203
H   1.13398 -2.77873  1.93036
H  -0.01733 -5.02691  1.87855
H  -1.07893 -3.80242  2.58846
H  -2.32080 -3.55340  0.43967
H  -2.30411 -5.26045  0.91616
H  -1.77052 -4.95189 -1.54608
H  -0.45713 -5.74775 -0.67200
H   0.47549 -3.97291 -2.17829
H  -0.59417 -2.75191 -1.44738
H   2.56376 -4.43801  0.98703
H   1.77782 -5.45327 -0.28151
H   4.48871 -0.11420 -3.83475
H   5.74781  0.48301 -2.68819
H   4.88008  0.72823 -0.14452
H   3.30920  1.54981 -0.01308
H   5.13606  3.24288 -0.10893
H   5.75402  2.56693 -1.62163
H   2.99026  3.87947 -1.22822
H   4.36090  4.57508 -2.12012
H   4.25804  2.72696 -3.79686
H   2.67780  3.51538 -3.68687
H   1.80371  1.71816 -2.18419
H   2.41635  1.00445 -3.69136
H  -0.57970  2.08672  1.56122
H  -1.91790  3.15739 -3.17517
H  -0.10256  4.48187 -2.01257
H  -4.05184  1.46987 -2.58104
H  -2.97089  0.04933 -2.55629
C  -3.09730  0.66170  1.88475
H  -2.32541 -0.60534  0.33678
H   1.30862  0.28678  1.53739
H  -6.69270 -2.56944 -0.98597
H  -7.02738 -1.98315 -2.66543
H  -5.53062 -2.93274 -2.30043
H  -7.52082  2.17239  0.38095
H  -7.52366  1.17407  1.89227
H  -6.44865  2.62214  1.74406
H   3.93941  1.28397  3.65915
H   2.97952  2.61269  4.37158
H   3.97398  1.57050  5.42577
H   3.26857 -1.16069  4.06550
H   3.30856 -0.87386  5.83147
H   1.84246 -1.54884  5.07177
H   0.43961  0.36758  6.09755
H   1.92083  1.02576  6.85673
H   0.89930  2.05908  5.80972
H  -2.18636  0.57418  2.49914
H  -3.51105  1.67309  2.04191
H  -3.83338 -0.07663  2.23980
```

96

TS(IV^{Me}-VI^{Me}).OPiv

```
BP86
SCF  =          -2339.94216181
H(0 K)=         -2339.169030
H(413 K)=       -2339.076691
G(413 K)=       -2339.317835
B97D       (1,2-C6Cl2H4)=       -
2339.58577975
Low Freq.=-322.6496cm-1, 5.556cm-
1
```

96

TS(IV^{Me}-VI^{Me}).OPiv

```
C  -3.12382  1.17523  1.63760
C  -3.66224  0.96616  0.21208
C  -3.61187  2.27910 -0.61025
C  -4.31362  3.44929  0.10847
C  -3.76084  3.65303  1.53160
C  -3.82496  2.34926  2.35113
C  -5.08749  0.32048  0.18855
O  -5.16150 -0.50864 -1.01605
C  -3.86179 -0.88349 -1.22725
N  -2.94067 -0.11079 -0.50550
C  -1.61544 -0.43911 -0.74070
N  -1.78161 -1.46770 -1.65429
C  -3.11907 -1.76681 -1.94734
O  -3.26022 -2.83327 -2.79395
C  -1.91122 -3.03514 -3.32681
C  -0.90985 -2.50910 -2.24692
C  -0.57409 -3.58928 -1.18650
C   0.38609 -4.66383 -1.73605
C   1.65964 -4.03638 -2.33201
C   1.31247 -2.99360 -3.41085
C   0.36788 -1.91071 -2.85443
Pd  0.08042  0.23417  0.16611
O   0.30313 -1.77545  1.46919
C  -0.10326 -1.52945  2.67079
C  -0.23545 -2.76110  3.65011
C   0.67067 -2.49024  4.87261
C   0.67933  2.09526 -0.41574
C  -0.15156  3.18725 -0.00662
C  -0.23219  4.34078 -0.78521
C   0.51952  4.54377 -1.94899
```

```
C   1.43873  3.52852 -2.29037
C   1.52783  2.34551 -1.54994
C   2.59955  1.31173 -1.72449
C   3.10328  0.93997 -0.25960
C   3.38147 -0.57757 -0.17929
O   3.63118 -0.96306  1.10055
C   3.65917 -2.39135  1.31976
F  -1.10965  5.32993 -0.38780
C   1.97750  1.30930  0.77962
C   4.40354  1.72887 -0.05810
O   5.50923  0.90692 -0.03705
C   6.76190  1.60990  0.07280
O   4.50324  2.94603  0.00532
O  -0.41455 -0.40433  3.14224
C  -1.70793 -2.81856  4.11900
C   0.15201 -4.09377  2.98596
O   3.36962 -1.35772 -1.12741
H  -2.03254  1.34707  1.56694
H  -3.22666  0.24051  2.21568
H  -4.88926  2.09373  2.54071
H  -3.36291  2.49347  3.34416
H  -2.71437  3.99910  1.45277
H  -4.32012  4.45657  2.04734
H  -4.18186  4.37055 -0.48671
H  -5.40724  3.26529  0.16080
H  -4.06348  2.10071 -1.60406
H  -2.54776  2.52777 -0.77215
H  -5.23129 -0.32786  1.07560
H  -5.90468  1.05421  0.12574
H  -1.81027 -2.45428 -4.26560
H  -1.80553 -4.10913 -3.54031
H  -1.51815 -4.05164 -0.84047
H  -0.12746 -3.07807 -0.31189
H   0.64366 -5.36857 -0.92410
H  -0.12172 -5.27023 -2.51618
H   2.23320 -3.52605 -1.53731
H   2.31439 -4.82235 -2.75456
H   0.84589 -3.49795 -4.28395
H   2.23376 -2.51015 -3.78019
H   0.87692 -1.34868 -2.04731
H   0.09415 -1.17492 -3.63335
H  -0.75015  3.12344  0.90607
H   2.11544  3.68179 -3.14171
H   0.43022  5.47341 -2.51666
H   2.22159  0.37228 -2.16206
H   3.42772  1.66027 -2.36795
H   1.82523  0.42487  1.43531
C   2.21043  2.47450  1.74715
H   6.90255  2.30011 -0.77607
H   6.80286  2.19605  1.00593
H   7.53837  0.83125  0.07146
H   2.61921 -2.76022  1.31858
H   4.25323 -2.89538  0.54090
H   4.11426 -2.52836  2.31150
H   0.01072 -4.93745  3.69029
H  -0.46145 -4.28202  2.08966
H   1.20790 -4.09073  2.66700
H  -1.85390 -3.60445  4.88548
H  -1.99874 -1.84375  4.54216
H  -2.38392 -3.04090  3.27318
H   0.54290 -3.27076  5.64783
H   1.73656 -2.47428  4.58050
H   0.42340 -1.50745  5.30502
H   3.09117  2.28942  2.39230
H   2.35495  3.43514  1.22998
H   1.32962  2.55251  2.40598
```

96
VI^{Me}.OPiv
BP86
SCF =   -2340.00378126
H(0 K)=   -2339.228200
H(413 K)=   -2339.135026
G(413 K)=   -2339.382510
B97D (1,2-C6Cl2H4) =   -2339.64604150
Low   Freq.   =   9.6077cm-1, 13.9281cm-1

96
VI^{Me}.OPiv

```
C  -2.49844  2.78989  1.47646
C  -2.83532  2.42279  0.01737
C  -1.72199  2.90858 -0.94390
C  -1.36756  4.39537 -0.75146
C  -1.00262  4.69196  0.71484
C  -2.15435  4.28083  1.65054
C  -4.26737  2.88672 -0.41875
O  -4.83159  1.84483 -1.28282
C  -4.16087  0.70651 -0.91822
N  -3.03002  0.96730 -0.13714
C  -2.27227 -0.15774  0.18143
N  -3.03614 -1.13126 -0.45638
C  -4.18311 -0.64392 -1.09682
O  -4.95986 -1.64410 -1.62831
C  -4.09031 -2.82105 -1.60332
C  -3.03537 -2.60422 -0.46532
C  -3.50828 -3.14063  0.91041
C  -3.41413 -4.67470  1.01608
C  -1.99274 -5.17145  0.69134
C  -1.54355 -4.68395 -0.69869
C  -1.64058 -3.15251 -0.81375
Pd -0.70296 -0.20352  1.30805
O   0.85938 -0.14900  2.73593
C   1.44687  0.96599  3.04039
C   2.28669  0.93417  4.37283
C   3.65971  1.58849  4.10732
C   2.00290  1.14067 -2.04526
C   1.89721  2.53878 -2.03195
C   1.49080  3.16893 -3.21441
C   1.19443  2.46682 -4.38811
C   1.30354  1.06283 -4.38073
C   1.70580  0.40626 -3.21119
C   1.86129 -1.07720 -2.95788
C   2.66906 -1.15395 -1.62224
C   2.22039 -2.36143 -0.77677
O   2.47440 -2.16394  0.53387
C   1.89330 -3.10650  1.46556
F   1.37112  4.53374 -3.22212
C   2.37366  0.23449 -0.89084
C   4.16473 -1.24820 -1.96639
O   4.88835 -1.84424 -0.97069
```

C 6.30849 -1.89183 -1.21919
O 4.67954 -0.83146 -2.99657
O 1.40035 2.05583 2.41649
C 1.51110 1.79007 5.40415
C 2.47087 -0.49444 4.91559
O 1.69773 -3.36857 -1.24924
H -1.62844 2.15816 1.76643
H -3.33968 2.48959 2.12949
H -3.04483 4.91339 1.44313
H -1.87928 4.46661 2.70460
H -0.10183 4.11428 0.99599
H -0.76198 5.76433 0.84383
H -0.53582 4.66028 -1.42797
H -2.22532 5.03524 -1.05139
H -2.02502 2.70152 -1.98739
H -0.83423 2.28377 -0.73219
H -4.92310 3.00098 0.46736
H -4.26664 3.82172 -1.00003
H -3.58169 -2.90844 -2.58419
H -4.73775 -3.69761 -1.44472
H -4.54000 -2.79029 1.10209
H -2.85510 -2.66326 1.66699
H -3.71841 -4.99492 2.03013
H -4.13322 -5.15018 0.31608
H -1.29231 -4.77858 1.45375
H -1.94554 -6.27541 0.75078
H -2.17209 -5.17074 -1.47460
H -0.50056 -4.98218 -0.90189
H -0.93304 -2.66278 -0.11227
H -1.35464 -2.81300 -1.82574
H 2.11132 3.12787 -1.13603
H 1.07863 0.49666 -5.29201
H 0.88846 3.01697 -5.28247
H 0.88144 -1.56474 -2.80905
H 2.37459 -1.60505 -3.77780
H 1.45138 0.07004 -0.27255
C 3.46093 0.78053 0.04350
H 6.52526 -2.43595 -2.15330
H 6.72370 -0.87402 -1.30248
H 6.73984 -2.41475 -0.35407
H 0.89758 -2.71907 1.74763
H 1.82498 -4.10495 1.00689
H 2.55271 -3.10919 2.34426
H 3.03438 -0.48105 5.86979
H 1.49581 -0.97779 5.08499
H 3.02821 -1.12019 4.19752
H 2.07048 1.86877 6.35677
H 1.34752 2.80415 5.00358
H 0.52431 1.34401 5.62225
H 4.23407 1.70842 5.04641
H 4.26485 0.97434 3.41603
H 3.51338 2.57579 3.64136
H 3.77618 0.00146 0.75312
H 4.34348 1.13643 -0.51991
H 3.04319 1.60892 0.63934

R = H (8b)

III^{H}
BP86
SCF = -2301.24085393
H(0 K)= -2300.478372
H(413 K)= -2300.387627
G(413 K)= -2300.623912
B97D (1,2-C6Cl2H4) = -2300.84239275
Low Freq. = 11.4866cm^{-1}, 13.0696cm^{-1}

94
III^{H}
C 2.59316 -3.09197 -0.53838
C 1.61568 -2.85840 -1.70473
C 0.29680 -3.64712 -1.51239
C 0.55465 -5.14570 -1.24658
C 1.53090 -5.36208 -0.07480
C 2.84744 -4.59532 -0.30287
C 2.27129 -3.17339 -3.09386
O 1.70338 -2.23039 -4.05528
C 1.42651 -1.13262 -3.30048
N 1.35064 -1.40404 -1.92763
C 1.09670 -0.27841 -1.17334
N 0.99962 0.69795 -2.14586
C 1.21662 0.20753 -3.43686
O 1.21578 1.17394 -4.39276
C 0.68183 2.35350 -3.70150
C 1.06464 2.19169 -2.19685
C 2.52054 2.64898 -1.91555
C 2.65639 4.18525 -1.91761
C 1.66373 4.83646 -0.93765
C 0.22007 4.40636 -1.25549
C 0.06825 2.87081 -1.24517
Pd 1.00680 0.04758 0.78107
O 1.31117 0.63328 2.82319
C 2.28193 1.42271 2.50248
C 3.03223 2.17237 3.61394
C -0.43461 -1.30640 1.08430
C -0.02517 -2.32333 1.97929
C -0.89274 -3.36982 2.30461
C -2.18145 -3.45497 1.77109
C -2.59676 -2.42397 0.91835
C -1.76058 -1.33540 0.57010
C -2.32694 -0.24887 -0.32597
C -3.29130 0.75597 0.39498
C -3.74676 1.80911 -0.63646
O -4.80197 2.52671 -0.15853
C -5.28410 3.56394 -1.04545
F -0.44841 -4.35749 3.13458
O 2.60794 1.54846 1.26494
C -2.59921 1.47917 1.57263
C -4.55237 0.04137 0.92438
O -5.25012 -0.52849 -0.10897
C -6.47245 -1.19036 0.29356
O -4.90479 -0.02669 2.08995
O -3.23111 2.02532 -1.72658
H 2.15361 -2.63952 0.36796
H 3.53729 -2.55319 -0.73911
H 3.38240 -5.02849 -1.17262
H 3.52268 -4.71711 0.56255
H 1.06161 -5.01432 0.86415
H 1.73621 -6.44005 0.05467
H -0.40988 -5.64351 -1.04397

```
H   0.96336 -5.63157 -2.15568
H  -0.33763 -3.51129 -2.40753
H  -0.24478 -3.20882 -0.65860
H   3.36737 -3.01990 -3.05321
H   2.04927 -4.18432 -3.46257
H  -0.41728  2.36778 -3.82638
H   1.13085  3.23533 -4.17892
H   3.19373  2.19502 -2.66604
H   2.80889  2.25843 -0.92412
H   3.69525  4.45511 -1.65722
H   2.48285  4.58689 -2.93656
H   1.91670  4.52151  0.09290
H   1.75439  5.93728 -0.97070
H  -0.07642  4.80822 -2.24562
H  -0.48536  4.84241 -0.52586
H   0.27442  2.48596 -0.22645
H  -0.96138  2.57302 -1.50855
H   0.96986 -2.31125  2.43485
H  -3.60337 -2.46902  0.48748
H  -2.83152 -4.29627  2.02499
H  -1.50338  0.34567 -0.74873
H  -2.87876 -0.69332 -1.17200
H  -1.70729  2.01743  1.21417
H  -3.28938  2.18690  2.05495
H  -2.27468  0.74137  2.32100
H  -6.25702 -2.00942  0.99859
H  -7.15513 -0.47641  0.78151
H  -6.91416 -1.58092 -0.63335
H  -4.49254  4.30448 -1.24248
H  -5.61262  3.13138 -2.00386
H  -6.12773  4.02947 -0.51850
C   4.08761  3.12131  3.01474
C   2.00020  2.96815  4.45095
C   3.71597  1.11065  4.51464
H   4.62841  3.63982  3.82595
H   4.82062  2.57067  2.40361
H   3.62097  3.88444  2.37019
H   2.50665  3.47414  5.29171
H   1.50211  3.74136  3.84023
H   1.22583  2.29866  4.85689
H   4.23901  1.60738  5.35079
H   2.97174  0.41319  4.93063
H   4.46063  0.52623  3.94671
```

TS(III^{H}-IV^{H})1

```
BP86
SCF  =    -2301.21570234
H(0 K)=   -2300.454674
H(413 K)=        -2300.364549
G(413 K)=        -2300.599997
B97D (1,2-C6Cl2H4) = -2300.82446342
```

Low Freq. = -35.8643cm^{-1}, 14.2399cm^{-1}

94

TS(III^{H}-IV^{H})1

```
C   0.40319 -1.84316 -3.12586
C   0.86355 -2.74611 -1.95440
C  -0.33662 -3.32490 -1.18373
C  -1.32589 -4.04281 -2.12470
C  -1.80497 -3.11239 -3.25511
C  -0.60957 -2.55161 -4.04736
C   1.85136 -3.88011 -2.40451
O   3.21496 -3.37771 -2.24225
C   3.11016 -2.37353 -1.33424
N   1.79266 -1.98606 -1.07018
C   1.72031 -0.87272 -0.26808
N   3.04406 -0.61819  0.00980
C   3.90543 -1.52303 -0.62528
O   5.21950 -1.30039 -0.35817
C   5.24210  0.07016  0.15294
C   3.85855  0.32514  0.84093
C   3.40475  1.78748  0.71078
C   4.25098  2.72423  1.59771
C   4.21885  2.28262  3.07235
C   4.68447  0.82253  3.22039
C   3.86134 -0.12456  2.32323
Pd  0.19934  0.30593  0.11010
O   0.81794  1.66222 -1.44493
C   0.65965  2.85446 -0.93832
C   0.88682  4.06642 -1.88259
C  -0.57358 -0.92049  1.51208
C  -1.95846 -1.26558  1.44828
C  -2.53856 -1.99658  2.50906
C  -1.80029 -2.40982  3.62629
C  -0.44386 -2.08014  3.65846
C   0.17139 -1.36356  2.62554
C  -2.84110 -0.89385  0.26979
C  -3.47713  0.53016  0.33508
C  -4.29644  0.74154 -0.95496
O  -5.12529  1.81461 -0.82942
C  -5.90019  2.12407 -2.01322
F   0.31208 -2.48014  4.71891
O   0.30575  3.04620  0.25548
C  -2.38117  1.60946  0.40086
C  -4.39864  0.65937  1.56192
O  -5.43013 -0.23580  1.48149
C  -6.35351 -0.17893  2.59504
O  -4.24232  1.42762  2.49665
O  -4.19572  0.08024 -1.97959
H   2.34183  1.86103  1.00766
H   3.44865  2.08993 -0.34995
H   5.30120  2.74707  1.24134
H   3.86624  3.75328  1.49353
H   3.18383  2.37674  3.45448
H   4.84716  2.94947  3.68950
H   4.59962  0.48790  4.26946
H   5.76042  0.75061  2.96439
H   4.22950 -1.16406  2.39590
H   2.81412 -0.11952  2.67193
H   5.38355  0.76269 -0.69878
H   6.09517  0.14341  0.84132
H   1.73303 -4.77609 -1.76638
H   1.73412 -4.16147 -3.46106
H   1.28889 -1.50532 -3.69444
H  -0.05472 -0.93821 -2.68466
H  -0.95233 -1.84081 -4.81971
H  -0.11128 -3.37879 -4.59295
H  -2.38645 -2.27511 -2.82368
H  -2.48965 -3.65499 -3.93138
H  -0.84714 -4.94186 -2.56400
```

H -2.18279 -4.41065 -1.53346
H -0.84515 -2.49243 -0.67156
H 0.02860 -4.00364 -0.39197
H 1.24073 -1.16258 2.71179
H -3.60161 -2.25843 2.44483
H -2.25050 -2.98126 4.44216
H -2.25865 -0.91561 -0.66833
H -3.65523 -1.62906 0.15938
H -2.78382 2.63071 0.33792
H -1.82865 1.54230 1.35197
H -1.69275 1.48180 -0.46286
C 1.52174 3.64416 -3.22126
C -0.50287 4.70575 -2.13485
C 1.79008 5.08976 -1.15647
H -5.83204 -0.39358 3.54134
H -6.81415 0.81949 2.66343
H -7.11328 -0.94423 2.38558
H -5.23549 2.36381 -2.85835
H -6.53714 1.26969 -2.29202
H -6.51213 2.99455 -1.74161
H 1.65926 4.52709 -3.87178
H 2.50856 3.17580 -3.06743
H 0.88857 2.91543 -3.75170
H 1.88920 6.01111 -1.75797
H 1.36428 5.34809 -0.17439
H 2.80482 4.68340 -0.99572
H -0.40114 5.60979 -2.76181
H -1.17399 4.00325 -2.65993
H -0.97536 4.99095 -1.18093

Int(III^{H}-IV^{H}
BP86
SCF = -2301.22259432
H(0 K)= -2300.461131
H(413 K)= -2300.370451
G(413 K)= -2300.607684
B97D (1,2-C6Cl2H4) = -2300.82757726
Low Freq. = 13.0205cm^{-1}, 15.9485cm^{-1}

94
Int(III^{H}-IV^{H})
C 3.71118 1.23204 -1.76983
C 3.38475 -0.28328 -1.80258
C 2.40700 -0.62533 -2.94107
C 2.89159 -0.09016 -4.30380
C 3.16522 1.42372 -4.24976
C 4.18091 1.75574 -3.14180
C 4.67489 -1.16826 -1.81466
O 5.09006 -1.33281 -0.41777
C 3.93350 -1.22189 0.28979
N 2.87254 -0.71023 -0.46757
C 1.73880 -0.51207 0.27947
N 2.12032 -0.94179 1.52064
C 3.44714 -1.38765 1.55517
O 3.82336 -1.85114 2.77676
C 2.79657 -1.33961 3.68894
C 1.48376 -1.14017 2.85292
C 0.69031 0.10457 3.29167
C -0.03372 -0.12073 4.63371
C -0.94063 -1.36402 4.58276
C -0.13743 -2.61481 4.18142
C 0.59693 -2.40960 2.84107
Pd 0.03573 0.23810 -0.33894
O 0.85666 2.22158 -0.32150
C 0.33502 2.97828 0.60939
C 0.88312 4.43331 0.71059
C -0.76840 -1.61489 -0.48045
C -2.13910 -1.80269 -0.13126
C -2.72927 -3.07465 -0.30619
C -2.01371 -4.17014 -0.81000
C -0.67390 -3.96471 -1.14385
C -0.04953 -2.72293 -0.98052
C -3.01294 -0.68183 0.41148
C -3.40654 0.39037 -0.65734
C -3.96848 -0.31010 -1.91352
O -5.15151 -0.92275 -1.62932
C -5.74329 -1.62881 -2.74761
F 0.05646 -5.00604 -1.63322
C -2.18226 1.23407 -1.05188
C -4.48598 1.37824 -0.16457
O -4.77759 1.23033 1.15420
C -5.75068 2.17911 1.65760
O -4.99693 2.22533 -0.88486
O -0.57175 2.61291 1.39729
O -3.42701 -0.34532 -3.00699
H -2.43426 2.03742 -1.76077
H -0.04405 0.35507 2.50299
H 1.37142 0.97216 3.34972
H 0.70628 -0.24022 5.45155
H -0.62111 0.78125 4.87789
H -1.74640 -1.19500 3.84210
H -1.43406 -1.52149 5.55870
H -0.79830 -3.49575 4.09916
H 0.59045 -2.85327 4.98330
H 1.22061 -3.28787 2.59261
H -0.13989 -2.29759 2.02592
H 3.14618 -0.37437 4.10201
H 2.69122 -2.07572 4.49826
H 4.46160 -2.16901 -2.23713
H 5.51770 -0.70726 -2.34816
H 4.48110 1.41419 -0.99710
H 2.79766 1.76681 -1.45066
H 4.34193 2.84602 -3.07381
H 5.16488 1.31730 -3.40590
H 2.21669 1.95770 -4.04743
H 3.53094 1.78206 -5.22869
H 3.81541 -0.62112 -4.61113
H 2.13301 -0.32341 -5.07143
H 1.42793 -0.17294 -2.68576
H 2.24949 -1.71818 -2.98184
H 1.00572 -2.64232 -1.25278
H -3.78300 -3.20705 -0.03273
H -2.47160 -5.15457 -0.93778
H -3.93637 -1.10890 0.83264
H -2.49938 -0.14391 1.22880
H -1.47137 0.58786 -1.64277
H -1.74959 1.71060 -0.14563
H -5.86908 1.93706 2.72219
H -5.38394 3.20941 1.52890
H -6.70658 2.07056 1.12120

H	-6.69097	-2.03482	-2.36876
H	-5.92196	-0.93853	-3.58686
H	-5.07746	-2.43895	-3.08465
C	1.42797	4.62619	2.14662
C	1.99118	4.72178	-0.31871
C	-0.31194	5.39207	0.48685
H	1.75124	5.67164	2.29911
H	0.64879	4.38375	2.88646
H	2.29951	3.97332	2.33376
H	2.34238	5.76489	-0.21648
H	2.85632	4.05378	-0.17348
H	1.62650	4.58070	-1.34924
H	0.00456	6.44119	0.62789
H	-0.71498	5.29507	-0.53677
H	-1.12178	5.16634	1.19841

TS(III^{H}-IV^{H})2

BP86

SCF = -2301.20249220

H(0 K)= -2300.445207

H(413 K)= -2300.355627

G(413 K)= -2300.589354

B97D (1,2-C6Cl2H4) = -2300.80044069

Low Freq. = -948.4638cm^{-1}, 12.9610cm^{-1}

94

TS(III^{H}-IV^{H})2

C	3.57737	2.09330	-1.21924
C	3.44785	0.61897	-1.68234
C	2.51619	0.49066	-2.90076
C	2.91514	1.44427	-4.04483
C	2.98997	2.90401	-3.56183
C	3.96267	3.03721	-2.37610
C	4.84498	-0.05124	-1.89850
O	5.28357	-0.54274	-0.58893
C	4.12124	-0.79865	0.07343
N	3.00042	-0.22808	-0.54175
C	1.83555	-0.42151	0.16354
N	2.27949	-1.15594	1.23415
C	3.65643	-1.40666	1.20373
O	4.08681	-2.15959	2.25333
C	2.98243	-2.09301	3.21422
C	1.67031	-1.87396	2.38687
C	0.63527	-1.00059	3.11946
C	-0.05624	-1.76194	4.26817
C	-0.69407	-3.07218	3.77016
C	0.34997	-3.96252	3.07179
C	1.05019	-3.21476	1.91948
Pd	-0.01047	0.26503	-0.26937
O	0.64366	2.25455	0.44977
C	-0.26255	2.88071	1.08805
C	0.10548	4.18909	1.81695
C	-0.58377	-1.55905	-0.91183
C	-1.87778	-2.05281	-0.58139
C	-2.28263	-3.31785	-1.05760
C	-1.45008	-4.11275	-1.86065
C	-0.19096	-3.60459	-2.18364
C	0.25251	-2.35966	-1.71948
C	-2.83480	-1.23094	0.25352
C	-3.29069	0.07618	-0.46742
C	-4.16876	-0.30160	-1.68508
O	-5.33496	-0.88721	-1.27327
C	-6.21335	-1.29120	-2.34894
F	0.64656	-4.34753	-2.96350
C	-2.07621	0.91621	-0.93399
C	-4.15589	0.88425	0.52203
O	-4.73516	1.94933	-0.10684
C	-5.53383	2.79014	0.75678
O	-4.30498	0.64232	1.71085
O	-1.48641	2.46671	1.14430
O	-3.87965	-0.14459	-2.86089
H	-2.40811	1.94049	-1.18597
H	-0.11686	-0.68018	2.37138
H	1.12592	-0.08077	3.48681
H	0.67840	-1.98952	5.06742
H	-0.81895	-1.10871	4.72719
H	-1.50460	-2.83195	3.05543
H	-1.16224	-3.61433	4.61119
H	-0.12194	-4.87764	2.67287
H	1.09631	-4.30405	3.81757
H	1.84039	-3.83975	1.46478
H	0.31924	-2.98889	1.12335
H	3.16208	-1.24019	3.89711
H	2.99222	-3.03444	3.78105
H	4.76398	-0.91296	-2.58885
H	5.61699	0.64451	-2.25587
H	4.32303	2.14703	-0.40441
H	2.60608	2.39553	-0.78896
H	3.98482	4.07687	-2.00346
H	4.99243	2.81723	-2.72382
H	1.98111	3.23442	-3.24669
H	3.29833	3.56710	-4.38996
H	3.89785	1.14399	-4.46174
H	2.18745	1.34256	-4.86899
H	1.48719	0.72132	-2.55793
H	2.50509	-0.55749	-3.25016
H	1.25873	-2.03697	-1.99749
H	-3.27868	-3.69054	-0.78912
H	-1.76189	-5.09340	-2.22972
H	-3.72492	-1.82093	0.52692
H	-2.35781	-0.90830	1.19566
H	-1.75557	0.51847	-1.91335
H	-1.56497	1.58722	0.29510
H	-5.93966	3.57702	0.10618
H	-6.34749	2.20942	1.22040
H	-4.90913	3.22585	1.55262
H	-7.09155	-1.73541	-1.86034
H	-6.50557	-0.42067	-2.95810
H	-5.71549	-2.02688	-3.00068
C	1.24647	3.87708	2.81692
C	0.61166	5.19559	0.75260
C	-1.11142	4.77207	2.56136
H	1.56775	4.80498	3.32132
H	0.91196	3.16801	3.59400
H	2.11377	3.43920	2.29824
H	0.91397	6.13757	1.24250
H	1.47916	4.78977	0.20848
H	-0.17817	5.43287	0.01902
H	-0.82061	5.70427	3.07666
H	-1.93361	5.00283	1.86536

H -1.49824 4.06584 3.31351

IV^H
BP86
SCF = -2301.21795814
H(0 K)= -2300.456001
H(413 K)= -2300.366041
G(413 K)= -2300.599593
B97D (1,2-C6Cl2H4) = -2300.81732022
Low Freq. = 12.6670cm^{-1}, 17.9582cm^{-1}

94
IV^H

```
C   3.17801  2.49754 -1.03642
C   3.39097  1.01757 -1.44460
C   2.67003  0.69393 -2.76623
C   3.04545  1.67884 -3.89072
C   2.78350  3.13690 -3.47048
C   3.53778  3.47631 -2.17189
C   4.90485  0.61638 -1.45403
O   5.24288  0.18424 -0.09415
C   4.06233 -0.27589  0.40848
N   2.95141  0.12303 -0.34289
C   1.74354 -0.28207  0.17966
N   2.16697 -0.96616  1.29809
C   3.55879 -0.99353  1.45449
O   3.96402 -1.72167  2.53412
C   2.74071 -1.90458  3.31775
C   1.54284 -1.85656  2.31087
C   0.26918 -1.23813  2.91152
C  -0.42429 -2.18807  3.90818
C  -0.73598 -3.54873  3.25797
C   0.53902 -4.19039  2.68106
C   1.24748 -3.24569  1.68999
Pd -0.16772  0.25748 -0.50462
O   0.10759  2.11996  0.71430
C  -0.58383  2.48769  1.68655
C  -0.28208  3.78320  2.46014
C  -0.48944 -1.40888 -1.61668
C  -1.72763 -1.79790 -2.21597
C  -1.83040 -3.04209 -2.88226
C  -0.75093 -3.92172 -3.02729
C   0.46426 -3.51684 -2.47592
C   0.59848 -2.30848 -1.78717
C  -2.97571 -0.93269 -2.30333
C  -3.17827  0.11038 -1.18537
C  -4.42025  0.95666 -1.54565
O  -4.98003  1.51007 -0.42401
C  -6.08999  2.40018 -0.68507
F   1.55706 -4.32996 -2.59340
O  -1.63432  1.83106  2.16165
C  -1.97118  1.06352 -1.14287
C  -3.43017 -0.55377  0.17149
O  -4.36811 -1.52385  0.08655
C  -4.71658 -2.17110  1.33331
O  -2.89419 -0.27522  1.25127
O  -4.82656  1.18270 -2.67617
H  -0.41703 -1.01429  2.07193
H   0.51767 -0.27322  3.38962
H   0.21933 -2.34389  4.79795
H  -1.35006 -1.71195  4.27606
H  -1.46527 -3.39600  2.43831
H  -1.21211 -4.22643  3.98954
H   0.30132 -5.13996  2.16957
H   1.22229 -4.45504  3.51337
H   2.19255 -3.69098  1.32907
H   0.60823 -3.08571  0.80293
H   2.66550 -1.07908  4.05243
H   2.83638 -2.86438  3.84491
H   5.07899 -0.23084 -2.14501
H   5.58014  1.44701 -1.70424
H   3.77446  2.71018 -0.12977
H   2.11525  2.61399 -0.75791
H   3.31419  4.50854 -1.84781
H   4.62905  3.44838 -2.36723
H   1.69685  3.27722 -3.30886
H   3.07788  3.83028 -4.27889
H   4.11496  1.56083 -4.16041
H   2.47015  1.42691 -4.79901
H   1.58026  0.74141 -2.56609
H   2.88695 -0.34891 -3.05934
H   1.58391 -2.07721 -1.37846
H  -2.79687 -3.32050 -3.32150
H  -0.84197 -4.87912 -3.54726
H  -2.95717 -0.34811 -3.24275
H  -3.86700 -1.57895 -2.37183
H  -1.83626  1.47122 -2.16254
H  -2.18957  1.92111 -0.47926
H  -1.90779  1.01866  1.60823
C   0.96263  4.47360  1.87272
C  -1.51626  4.71549  2.34177
C  -0.04283  3.42581  3.94985
H  -3.84310 -2.69379  1.75374
H  -5.07959 -1.43017  2.06304
H  -5.50808 -2.88767  1.07640
H  -5.76209  3.26147 -1.28921
H  -6.89189  1.87361 -1.22699
H  -6.43935  2.73117  0.30253
H   1.17180  5.39990  2.43477
H   1.84955  3.82272  1.93422
H   0.81185  4.73764  0.81371
H   0.15439  4.34754  4.52404
H  -0.92083  2.92385  4.38516
H   0.83190  2.76247  4.06569
H  -1.32757  5.65078  2.89662
H  -1.71690  4.97996  1.28922
H  -2.41693  4.23694  2.75670
```

V^H
BP86
SCF = -1954.15852395
H(0 K)= -1953.541487
H(413 K)= -1953.468209
G(413 K)= -1953.667920
B97D (1,2-C6Cl2H4) = -1953.84060212
Low Freq. = 7.3694cm^{-1}, 14.4962cm^{-1}

77

V^H

```
C   0.03574  0.33305 -2.74435
C   0.83315  0.26463 -1.58541
C   2.13735  0.82812 -1.58828
C   2.60362  1.45775 -2.75994
C   1.81703  1.53772 -3.92080
C   0.54153  0.96876 -3.88716
Pd  0.14336 -0.61264  0.05972
C   1.97051 -1.55980 -0.07779
C   3.13593 -0.62505  0.31293
C   3.14654 -0.43917  1.83885
O   2.22052 -0.67954  2.60424
C   2.99283  0.80307 -0.33703
F  -0.24258  1.02842 -5.00188
C  -1.78322  0.14385  0.34917
N  -2.97022 -0.55473  0.27464
C  -4.09963  0.19959  0.59474
C  -3.63601  1.45964  0.85656
N  -2.24853  1.38638  0.71849
C  -3.34551 -1.97549  0.08422
C  -4.89061 -1.77293 -0.09421
O  -5.25056 -0.52972  0.59439
C  -2.66871 -2.57701 -1.16264
C  -2.96499 -4.08145 -1.31972
C  -2.57545 -4.86776 -0.05417
C  -3.28052 -4.29478  1.18893
C  -2.99303 -2.78998  1.35571
O  -4.08222  2.70884  1.16277
C  -2.87266  3.43498  1.56525
C  -1.65863  2.74074  0.85730
C  -1.37133  3.33824 -0.54397
C  -0.67661  4.71175 -0.46118
C   0.60377  4.64221  0.39072
C   0.29781  4.09330  1.79696
C  -0.38755  2.71426  1.72652
C   4.46698 -1.25547 -0.14292
O   5.20473 -0.84115 -1.02605
O   4.70261 -2.41496  0.54544
C   5.90794 -3.10726  0.15136
O   4.32876  0.10526  2.25260
C   4.41889  0.35383  3.67432
H   0.29828  1.96861  1.27770
H  -0.64031  2.33996  2.73541
H  -0.35113  4.81147  2.33870
H   1.22424  4.00891  2.39237
H   1.33486  3.97816 -0.10983
H   1.07418  5.63937  0.46158
H  -0.44723  5.06377 -1.48233
H  -1.36568  5.46352 -0.02526
H  -2.31925  3.40760 -1.10885
H  -0.72317  2.62468 -1.08436
H  -2.77558  3.36687  2.66577
H  -3.01420  4.48383  1.26799
H  -5.14700 -1.66617 -1.16561
H  -5.49499 -2.57483  0.35367
H  -3.53995 -2.37201  2.22066
H  -1.91431 -2.63999  1.55307
H  -2.96323 -4.83025  2.10126
H  -4.37282 -4.46289  1.10051
H  -1.47867 -4.80703  0.08780
H  -2.82084 -5.93796 -0.17568
H  -4.04294 -4.23537 -1.53073
H  -2.42175 -4.46844 -2.19969
H  -1.57549 -2.41078 -1.05486
H  -2.98644 -2.01269 -2.05835
H  -0.96931 -0.09561 -2.77622
H   3.61154  1.88875 -2.76589
H   2.17979  2.01859 -4.83301
H   3.99877  1.20073 -0.54512
H   2.52493  1.46570  0.41633
H   2.14950 -1.97319 -1.08455
H   1.89528 -2.40112  0.63815
H   3.64776  1.07320  3.99440
H   4.28703 -0.58196  4.24066
H   5.42385  0.76701  3.83631
H   5.85826 -3.40244 -0.90933
H   6.79192 -2.46527  0.29694
H   5.96212 -3.99447  0.79737
```

TS(V^H-VI^H)

BP86

SCF = -1954.14324385

H(0 K)= -1953.527155

H(413 K)= -1953.454534

G(413 K)= -1953.652742

B97D (1,2-C6Cl2H4) = -1953.81865833

Low Freq. = -305.9640cm^{-1}, 7.2280cm^{-1}

77

TS(V^H-VI^H)

```
C   1.22190  1.96262 -2.06953
C   1.49664  1.23371 -0.88733
C   2.32759  1.80863  0.11077
C   2.78290  3.12829 -0.02861
C   2.44988  3.89065 -1.16322
C   1.68597  3.28074 -2.16597
Pd  0.07724 -0.15113 -0.42801
C   2.15389 -0.65709 -0.75590
C   3.06930 -0.49719  0.48633
C   2.86665 -1.67208  1.46615
O   2.08182 -2.60231  1.35179
C   2.75326  0.86088  1.20518
F   1.38095  3.99720 -3.28559
C  -1.92975 -0.18067 -0.00920
N  -2.82132 -1.21595 -0.18566
C  -4.12145 -0.92578  0.23264
C  -4.08636  0.36943  0.66712
N  -2.75663  0.77753  0.53177
C  -2.71684 -2.64333 -0.57074
C  -4.24929 -2.94556 -0.71688
O  -4.96915 -1.98801  0.12731
C  -1.94602 -2.81639 -1.89378
C  -1.74038 -4.29962 -2.25900
C  -1.04663 -5.06847 -1.11892
C  -1.83488 -4.93024  0.19652
C  -2.04822 -3.45050  0.57093
O  -4.90921  1.35481  1.12201
C  -3.99847  2.39106  1.61634
C  -2.63172  2.21701  0.86570
C  -2.54630  3.05649 -0.43381
```

C -2.34856 4.55848 -0.14829
C -1.13030 4.80490 0.76086
C -1.24283 3.99319 2.06491
C -1.41865 2.48905 1.77571
C 4.55312 -0.49647 0.05177
O 5.34979 0.41777 0.19339
O 4.86490 -1.68251 -0.55064
C 6.23222 -1.77600 -1.01598
O 3.69852 -1.52266 2.53808
C 3.58671 -2.56134 3.53956
H -0.52364 2.09578 1.25544
H -1.52719 1.90987 2.71125
H -2.09971 4.36770 2.66204
H -0.34494 4.14044 2.69086
H -0.20787 4.50581 0.22689
H -1.03351 5.88221 0.98702
H -2.23579 5.09836 -1.10517
H -3.25641 4.97605 0.33304
H -3.45478 2.88336 -1.03989
H -1.68673 2.67047 -1.01241
H -3.86512 2.24727 2.70576
H -4.48214 3.36039 1.42784
H -4.57555 -2.80181 -1.76486
H -4.53284 -3.95165 -0.37538
H -2.65870 -3.35603 1.48795
H -1.07106 -2.97557 0.77970
H -1.30820 -5.44161 1.02155
H -2.81333 -5.44212 0.09297
H -0.02669 -4.66310 -0.97392
H -0.93174 -6.13379 -1.38859
H -2.71828 -4.77384 -2.48130
H -1.14903 -4.36674 -3.18949
H -0.96367 -2.31449 -1.76779
H -2.47374 -2.27320 -2.69900
H 0.63708 1.53769 -2.88928
H 3.43142 3.56149 0.74118
H 2.80638 4.91518 -1.29664
H 3.63522 1.21666 1.75892
H 1.92847 0.69137 1.92292
H 2.69286 -0.55754 -1.70613
H 1.67785 -1.66419 -0.74865
H 2.57459 -2.57192 3.97489
H 3.79431 -3.54917 3.09823
H 4.33445 -2.31125 4.30429
H 6.43574 -1.00534 -1.77668
H 6.93651 -1.64209 -0.17933
H 6.32615 -2.78155 -1.44784

VI^{H}
BP86
SCF = -1954.19392350
H(0 K)= -1953.575507
H(413 K)= -1953.502407
G(413 K)= -1953.701568
B97D (1,2-C6Cl2H4) = -1953.86639656
Low Freq. = 9.0669cm^{-1}, 20.2743cm^{-1}

77
VI^{H}
C 0.70028 2.05751 -1.34912
C 1.62035 1.41818 -0.45549
C 1.89081 1.99954 0.82766
C 1.29232 3.19437 1.21959
C 0.37795 3.84158 0.35061
C 0.09951 3.26628 -0.88399
Pd -0.41300 0.44173 -0.37248
C 2.66292 0.34999 -0.77674
C 3.73341 0.50939 0.35576
C 4.28955 -0.84926 0.80686
O 3.82399 -1.94836 0.53985
C 2.98382 1.22336 1.53307
F -0.75539 3.91044 -1.73221
C -2.01237 -0.74067 -0.03011
N -2.18693 -2.10401 -0.13980
C -3.47676 -2.54111 0.17131
C -4.18798 -1.41172 0.46627
N -3.27878 -0.35796 0.36206
C -1.30102 -3.26681 -0.37959
C -2.41223 -4.35588 -0.57995
O -3.60996 -3.89725 0.12863
C -0.43067 -3.06579 -1.63465
C 0.55558 -4.23107 -1.84826
C 1.43727 -4.46169 -0.60645
C 0.57360 -4.68756 0.64842
C -0.41945 -3.52911 0.86706
O -5.45422 -1.00269 0.76332
C -5.29548 0.39503 1.17850
C -3.98659 0.93615 0.50766
C -4.23906 1.54942 -0.89357
C -4.88649 2.94552 -0.81936
C -4.05959 3.89907 0.06249
C -3.86163 3.30523 1.46998
C -3.20431 1.91250 1.40640
C 4.90026 1.40011 -0.11952
O 5.22080 2.48253 0.34577
O 5.53989 0.81025 -1.17198
C 6.65527 1.56932 -1.69647
O 5.37053 -0.66949 1.61620
C 5.94696 -1.89103 2.13855
H -2.18152 1.99017 0.98153
H -3.09452 1.47344 2.41512
H -4.84349 3.24121 1.98286
H -3.23581 3.97275 2.08873
H -3.07194 4.06239 -0.40902
H -4.55229 4.88616 0.12801
H -4.99413 3.35241 -1.84071
H -5.91489 2.86714 -0.41104
H -4.85956 0.85332 -1.48793
H -3.25405 1.61722 -1.39295
H -5.20781 0.42370 2.28166
H -6.20427 0.92872 0.86442
H -2.66062 -4.46399 -1.65337
H -2.14681 -5.33728 -0.16091
H -1.07094 -3.72142 1.73959
H 0.13291 -2.59279 1.07195
H 1.21145 -4.79482 1.54378
H 0.02149 -5.64461 0.54985
H 2.08868 -3.58261 -0.44484
H 2.10616 -5.32631 -0.76934
H -0.00454 -5.15993 -2.08240

H 1.18152 -4.02073 -2.73393
H 0.11768 -2.11096 -1.49763
H -1.08545 -2.92689 -2.51457
H 0.65556 1.82367 -2.41863
H 1.54351 3.65483 2.18085
H -0.09649 4.78837 0.62156
H 3.67701 1.86241 2.10128
H 2.55773 0.46940 2.22099
H 3.10707 0.46931 -1.77880
H 2.24666 -0.67015 -0.72166
H 5.21082 -2.43434 2.75217
H 6.27673 -2.54504 1.31580
H 6.80175 -1.57186 2.74966
H 6.31552 2.54846 -2.07009
H 7.41387 1.73216 -0.91435
H 7.06363 0.96166 -2.51528

TS(IV^{H}-VI^{H})
BP86
SCF = -2301.18355081
H(0 K)= -2300.423352
H(413 K)= -2300.333000
G(413 K)= -2300.573924
B97D (1,2-C6Cl2H4) = -2300.77834193
Low Freq. = -305.7741cm^{-1}, 8.2006cm^{-1}

94
TS(IV^{H}-VI^{H})
C 0.25188 3.83455 -0.87847
C -0.73857 3.72981 0.30805
C -0.00035 3.33237 1.60030
C 1.16316 4.29517 1.91077
C 2.15129 4.38216 0.73213
C 1.42943 4.78078 -0.56890
C -1.61149 5.02143 0.48893
O -2.84273 4.83418 -0.28401
C -2.98792 3.48267 -0.36099
N -1.83180 2.78040 -0.01630
C -1.94474 1.41361 -0.14988
N -3.24418 1.30895 -0.59809
C -3.90710 2.53199 -0.70786
O -5.21860 2.40790 -1.05435
C -5.35052 1.01409 -1.48749
C -4.20599 0.19701 -0.79433
C -3.62563 -0.90386 -1.70098
C -4.57916 -2.10761 -1.83583
C -4.95607 -2.68216 -0.45731
C -5.56844 -1.59327 0.44278
C -4.62620 -0.38140 0.58065
Pd -0.57061 -0.05286 0.24686
O 3.60671 1.21027 0.23380
C 4.68263 0.74189 -0.13999
C 6.06557 1.26657 0.29916
C -0.56467 -1.79636 1.30295
C -0.79921 -3.05548 0.68757
C -1.42718 -4.08434 1.40667
C -1.81081 -3.89817 2.74714
C -1.51174 -2.67178 3.35266
C -0.87127 -1.62689 2.67510
C -0.23447 -3.19627 -0.70380
C 1.15613 -2.48399 -0.67760
C 2.25080 -3.54212 -0.34048
O 3.43215 -2.93046 -0.06467
C 4.52891 -3.82551 0.24148
F -1.85427 -2.48497 4.65924
O 4.78272 -0.29275 -0.99931
C 1.15197 -1.34936 0.39225
C 1.56487 -1.90205 -2.04522
O 0.86183 -2.43883 -3.06733
C 1.23016 -1.95708 -4.38541
O 2.45887 -1.07517 -2.23578
O 2.09192 -4.75330 -0.32942
H -2.66796 -1.22259 -1.24301
H -3.37968 -0.47267 -2.68897
H -5.50026 -1.80252 -2.37370
H -4.10164 -2.88234 -2.46197
H -4.04739 -3.08580 0.02943
H -5.66035 -3.52573 -0.57300
H -5.78988 -1.99718 1.44648
H -6.54243 -1.27237 0.02008
H -5.09392 0.41871 1.18362
H -3.69909 -0.68469 1.10155
H -5.24252 0.97936 -2.58867
H -6.35999 0.68309 -1.20379
H -1.88512 5.15967 1.55250
H -1.12958 5.93371 0.10930
H -0.29437 4.16097 -1.78288
H 0.63417 2.81531 -1.07482
H 2.13271 4.77428 -1.42070
H 1.06193 5.82406 -0.48543
H 2.64129 3.39980 0.59618
H 2.94876 5.11301 0.96049
H 0.76440 5.30484 2.14141
H 1.68209 3.95438 2.82405
H 0.38303 2.30126 1.45935
H -0.72315 3.29223 2.43596
H -0.65512 -0.69656 3.20628
H -1.59599 -5.05513 0.92657
H -2.29388 -4.68911 3.32641
H -0.12368 -4.24667 -1.01404
H -0.87403 -2.68250 -1.44189
H 1.70154 -1.62515 1.29989
H 1.63603 -0.41776 0.01858
H 3.86070 -0.57089 -1.27600
C 5.88061 2.37181 1.35506
C 6.89244 0.09709 0.88891
C 6.78965 1.83336 -0.94938
H 1.02391 -0.87859 -4.46789
H 2.30018 -2.13470 -4.57440
H 0.60671 -2.52876 -5.08531
H 4.33533 -4.36066 1.18490
H 4.66277 -4.56279 -0.56578
H 5.40923 -3.17659 0.33225
H 6.86518 2.75421 1.67589
H 5.29404 3.21312 0.95298
H 5.34891 1.99077 2.24212
H 7.78738 2.21171 -0.66505
H 6.91657 1.05686 -1.72032
H 6.22341 2.67085 -1.39238
H 7.88599 0.46171 1.20416

H 6.39583 -0.33674 1.77422
H 7.03557 -0.70148 0.14406

VI^H
BP86
SCF = -2301.23185807
H(0 K)= -2300.470070
H(413 K)= -2300.378763
G(413 K)= -2300.625156
B97D (1,2-C6Cl2H4) = -2300.82563364
Low Freq. = 6.1854cm^{-1}, 11.2559cm^{-1}

94
VI^H
C -0.29836 3.31895 -1.20416
C -1.33407 3.47157 -0.06233
C -0.65463 3.34076 1.31373
C 0.53176 4.31250 1.46831
C 1.56197 4.13051 0.33785
C 0.89736 4.27826 -1.04371
C -2.20021 4.77261 -0.19386
O -3.39303 4.43051 -0.97484
C -3.54689 3.08956 -0.78443
N -2.42031 2.47831 -0.23115
C -2.53019 1.11058 -0.09410
N -3.80160 0.91272 -0.59151
C -4.44761 2.08214 -0.99354
O -5.73234 1.87487 -1.40090
C -5.83478 0.42086 -1.55878
C -4.73998 -0.23165 -0.64472
C -4.08285 -1.47000 -1.28365
C -5.00621 -2.70334 -1.25022
C -5.46930 -3.01627 0.18480
C -6.16373 -1.79504 0.81509
C -5.25383 -0.55200 0.78177
Pd -1.25206 -0.23406 0.69456
O 3.81380 1.21742 0.82616
C 4.86139 1.32950 0.19074
C 6.08077 2.15356 0.65219
C 0.39744 -1.77548 0.86337
C -0.04827 -2.99093 0.24709
C -0.96368 -3.82708 0.88215
C -1.49257 -3.46193 2.14533
C -1.06991 -2.27428 2.73738
C -0.10673 -1.39936 2.15069
C 0.72793 -3.24246 -1.02948
C 2.04206 -2.42750 -0.80556
C 3.08134 -3.33144 -0.09728
O 4.27431 -2.68140 0.01261
C 5.30855 -3.42925 0.69867
F -1.53645 -1.95453 3.97496
O 5.07619 0.73757 -1.00528
C 1.63376 -1.24395 0.14458
C 2.68127 -1.91580 -2.09600
O 2.66588 -2.86592 -3.06091
C 3.27951 -2.47736 -4.31709
O 3.16338 -0.79759 -2.27836
O 2.87920 -4.45931 0.32237
H -3.15199 -1.67003 -0.71243
H -3.77496 -1.22693 -2.31746
H -5.89340 -2.53372 -1.89459
H -4.47385 -3.56896 -1.68337
H -4.58686 -3.28784 0.79599
H -6.14615 -3.88981 0.19007
H -6.45420 -2.00687 1.85964
H -7.10835 -1.59052 0.27085
H -5.77516 0.33411 1.18867
H -4.35892 -0.72188 1.41040
H -5.65263 0.17183 -2.62196
H -6.86180 0.13926 -1.28381
H -2.52541 5.12508 0.80378
H -1.69057 5.58847 -0.72669
H -0.80246 3.47052 -2.17668
H 0.05279 2.27020 -1.17813
H 1.62795 4.08770 -1.85018
H 0.56029 5.32637 -1.17930
H 2.02544 3.12954 0.42333
H 2.37842 4.86795 0.44634
H 0.16436 5.35968 1.47004
H 1.00690 4.15229 2.45232
H -0.31036 2.28923 1.40464
H -1.40689 3.49532 2.10898
H 0.38833 -0.65458 2.78339
H -1.25709 -4.77962 0.42816
H -2.20083 -4.10278 2.67710
H 0.94266 -4.30723 -1.20594
H 0.19313 -2.84673 -1.91267
H 2.44773 -0.93602 0.81894
H 1.39939 -0.35827 -0.46959
H 4.26205 0.22197 -1.26433
C 5.76776 2.79783 2.01528
C 7.30455 1.21028 0.77315
C 6.37442 3.24739 -0.40563
H 2.73452 -1.63163 -4.76472
H 4.32860 -2.18348 -4.15758
H 3.21357 -3.36643 -4.95779
H 5.01912 -3.61180 1.74574
H 5.47957 -4.39752 0.20229
H 6.20521 -2.79780 0.64825
H 6.63421 3.38771 2.36129
H 4.89443 3.46619 1.94796
H 5.53930 2.03268 2.77456
H 7.24987 3.84411 -0.09436
H 6.58678 2.80137 -1.38995
H 5.51835 3.93526 -0.51572
H 8.19104 1.78571 1.09306
H 7.12652 0.42102 1.52423
H 7.53049 0.72779 -0.19084

V^H.OPiv
BP86
SCF = -2300.67872598
H(0 K)= -2299.931142
H(413 K)= -2299.840910
G(413 K)= -2300.078257
B97D (1,2-C6Cl2H4) = -2300.33429096
Low Freq. = 7.3099cm^{-1}, 15.8684cm^{-1}

93
V^H.OPiv
C -0.04073 -2.87011 -0.30750
C -0.83093 -1.73367 -0.63476
C -2.02503 -2.00470 -1.37971
C -2.36992 -3.32431 -1.74187
C -1.57461 -4.43069 -1.40206
C -0.41298 -4.16580 -0.67693
Pd -0.20392 0.13882 -0.17290
C -2.13308 0.91572 -0.24642
C -3.34009 0.02972 -0.60945
C -3.82161 -0.80898 0.58635
O -3.39450 -0.80288 1.72867
C -2.97440 -0.89805 -1.79806
F 0.39969 -5.21665 -0.31924
C 1.78669 -0.40288 0.05969
N 2.76525 -0.55464 -0.89582
C 4.02926 -0.86512 -0.38304
C 3.86011 -0.96383 0.96726
N 2.51207 -0.66614 1.20217
C 2.87154 -0.22442 -2.33290
C 4.25130 -0.91193 -2.60802
O 5.00074 -0.90863 -1.34786
C 1.71457 -0.83244 -3.14647
C 1.74233 -0.39294 -4.62299
C 1.74460 1.14202 -4.74874
C 2.93072 1.75046 -3.97810
C 2.92395 1.31691 -2.49940
O 4.54901 -1.27799 2.10455
C 3.68238 -0.81958 3.19433
C 2.20905 -0.73469 2.65274
C 1.37266 -1.99648 2.97552
C 0.95735 -2.06140 4.45858
C 0.21734 -0.78082 4.88748
C 1.07908 0.46482 4.61092
C 1.48759 0.53962 3.12839
O 0.43619 2.22890 -0.07030
C 0.14389 3.04059 0.89968
O -0.38914 2.75774 1.99756
C 0.50578 4.55240 0.64342
C 1.42352 5.01685 1.79705
C 1.19990 4.77754 -0.71132
C -0.81811 5.34999 0.69874
C -4.47020 0.96919 -1.04356
O -4.74799 1.29167 -2.19401
O -5.12495 1.50089 0.04446
C -6.11821 2.48617 -0.28763
O -4.86942 -1.62223 0.19874
C -5.36529 -2.47293 1.24685
H 0.58552 0.70169 2.50796
H 2.13429 1.41546 2.94108
H 1.97737 0.44306 5.26539
H 0.52447 1.38479 4.86403
H -0.72548 -0.69781 4.31463
H -0.05674 -0.83544 5.95803
H 0.32398 -2.95305 4.61869
H 1.85280 -2.20044 5.10087
H 1.93830 -2.90054 2.68114
H 0.46683 -1.95562 2.34374
H 4.03136 0.17971 3.51898
H 3.80054 -1.53648 4.02141
H 4.10199 -1.96255 -2.92625
H 4.86759 -0.38229 -3.35018
H 3.81723 1.70348 -1.97354
H 2.04094 1.73355 -1.97853
H 2.90782 2.85388 -4.03454
H 3.87805 1.43677 -4.46527
H 0.79956 1.53841 -4.33051
H 1.77843 1.44401 -5.81242
H 2.64063 -0.80321 -5.13121
H 0.86908 -0.82337 -5.14544
H 0.77353 -0.49879 -2.66320
H 1.73477 -1.93296 -3.05122
H 0.89813 -2.76044 0.24114
H -3.29415 -3.49107 -2.31078
H -1.84176 -5.45447 -1.68067
H -2.51711 -0.24654 -2.56561
H -3.88851 -1.32876 -2.24133
H -2.05182 1.70841 -1.01655
H -2.27154 1.39009 0.73874
H -4.58261 -3.17171 1.58678
H -5.70274 -1.87860 2.11297
H -6.20787 -3.02766 0.80715
H -5.65872 3.35476 -0.78919
H -6.88715 2.06527 -0.95797
H -6.56478 2.79119 0.67013
H 1.42656 5.85196 -0.85978
H 2.14597 4.21376 -0.77054
H 0.56085 4.43742 -1.54236
H 1.61650 6.10561 1.74110
H 0.94826 4.78334 2.76330
H 2.39967 4.49894 1.76084
H -0.63054 6.43820 0.61701
H -1.49013 5.05644 -0.12745
H -1.33722 5.14374 1.64856

TS(V^H-VI^H).OPiv
BP86
SCF = -2300.63718658
H(0 K)= -2299.891502
H(413 K)= -2299.801763
G(413 K)= -2300.037327
B97D (1,2-C6Cl2H4) = -2300.28997189
Low Freq. = -357.8861cm^{-1}, 6.0651cm^{-1}

93
TS(V^H-VI^H).OPiv
C -0.34280 3.22784 0.16459
C 0.55394 2.20944 -0.29686
C 1.37638 2.56043 -1.42784
C 1.19667 3.76210 -2.12007
C 0.21456 4.69853 -1.73167
C -0.50755 4.40103 -0.56789
Pd 0.07307 0.28111 0.20028
C 1.86263 1.45671 0.81783
C 3.02648 1.21761 -0.17853
C 4.23668 2.08876 0.18942
O 4.24183 3.06659 0.92251
C 2.53369 1.62307 -1.63249
F -1.44254 5.31513 -0.12544

C	-1.56750	-0.49529	-0.72109
N	-1.66055	-1.51009	-1.66075
C	-2.97379	-1.89174	-1.96805
C	-3.77741	-1.07914	-1.23026
N	-2.91322	-0.26465	-0.48467
C	-0.71849	-2.47954	-2.26758
C	-1.67629	-3.04664	-3.36603
O	-3.03918	-2.94546	-2.83959
C	0.52312	-1.78859	-2.85234
C	1.53903	-2.79883	-3.42091
C	1.94333	-3.83919	-2.35986
C	0.70728	-4.55828	-1.78921
C	-0.32137	-3.55689	-1.22591
O	-5.10035	-0.79675	-1.01679
C	-5.08658	0.00684	0.20692
C	-3.70617	0.74277	0.25621
C	-3.73510	2.07943	-0.52779
C	-4.51696	3.18017	0.21742
C	-3.98745	3.37971	1.65000
C	-3.96775	2.05189	2.43240
C	-3.18757	0.94749	1.69009
O	0.53627	-1.75496	1.42027
C	0.17833	-1.57190	2.64813
O	-0.18307	-0.48501	3.17035
C	0.18214	-2.83669	3.59444
C	-1.25830	-3.01134	4.12944
C	0.62837	-4.11965	2.87187
C	1.12515	-2.53547	4.78119
C	3.44606	-0.26443	-0.15331
O	3.39659	-1.04893	-1.09387
O	3.87180	-0.61533	1.09144
C	4.01971	-2.03904	1.30012
O	5.36528	1.65885	-0.47098
C	6.54053	2.44874	-0.20550
H	-2.11013	1.19494	1.62621
H	-3.23214	-0.00839	2.24089
H	-5.01325	1.72059	2.60885
H	-3.52014	2.19891	3.43166
H	-2.96667	3.79805	1.58885
H	-4.60294	4.12832	2.18406
H	-4.44290	4.12487	-0.35045
H	-5.59638	2.92319	0.25551
H	-4.16620	1.90110	-1.53065
H	-2.68755	2.40007	-0.67153
H	-5.19312	-0.66999	1.07792
H	-5.94937	0.68731	0.15562
H	-1.60790	-2.44028	-4.29157
H	-1.49895	-4.10655	-3.60140
H	-1.23638	-4.08397	-0.89451
H	0.09132	-3.03585	-0.34012
H	1.00260	-5.26203	-0.98935
H	0.24516	-5.17896	-2.58643
H	2.47481	-3.31036	-1.54834
H	2.65067	-4.57292	-2.79172
H	1.11240	-3.31457	-4.30773
H	2.43038	-2.25046	-3.77286
H	0.99125	-1.21047	-2.03195
H	0.20860	-1.05790	-3.62077
H	-0.91834	3.08966	1.08375
H	1.84854	3.99258	-2.97388
H	0.05284	5.63913	-2.26400
H	2.24262	0.67848	-2.12524
H	3.34867	2.06532	-2.23279
H	1.78820	0.65580	1.58346
H	2.01956	2.38656	1.37879
H	6.39307	3.49396	-0.52525
H	6.78130	2.44538	0.87071
H	7.34958	1.97915	-0.78372
H	3.01478	-2.49436	1.31177
H	4.63805	-2.49040	0.50750
H	4.50387	-2.14245	2.28179
H	0.58343	-4.99009	3.55620
H	-0.01531	-4.32749	2.00147
H	1.66356	-4.03245	2.50085
H	-1.31205	-3.82623	4.87753
H	-1.59502	-2.07120	4.59487
H	-1.95748	-3.25755	3.30950
H	1.08787	-3.34220	5.53909
H	2.17257	-2.43873	4.44147
H	0.83285	-1.58307	5.25177

VI^{H}.OPiv

BP86

SCF = -2300.69603361

H(0 K)= -2299.947529

H(413 K)= -2299.857305

G(413 K)= -2300.098331

B97D (1,2-C6Cl2H4) = -2300.34312700

Low Freq. = 10.4753cm^{-1}, 13.5879cm^{-1}

93

VI^{H}.OPiv

C	0.40649	3.77597	0.47853
C	1.20480	2.96247	-0.33927
C	1.43295	3.30564	-1.69092
C	0.87442	4.46918	-2.23487
C	0.07645	5.30072	-1.42536
C	-0.13801	4.93333	-0.09197
Pd	-0.63379	-0.92902	0.94997
C	1.87997	1.65966	0.01114
C	2.90668	1.46919	-1.15511
C	4.26054	2.08817	-0.75878
O	4.54701	2.61852	0.30306
C	2.29070	2.25170	-2.35981
F	-0.91626	5.74718	0.68862
C	-2.14990	-0.73880	-0.22904
N	-2.39648	-1.42452	-1.41596
C	-3.66346	-1.20426	-1.96561
C	-4.26536	-0.29328	-1.15016
N	-3.35153	-0.04490	-0.11718
C	-1.72404	-2.54181	-2.10594
C	-2.60161	-2.55894	-3.40255
O	-3.91095	-1.98830	-3.06321
C	-0.24320	-2.21457	-2.36967
C	0.54602	-3.40470	-2.94222
C	0.42444	-4.63584	-2.02554
C	-1.05553	-5.01526	-1.83062
C	-1.85808	-3.83253	-1.25656
O	-5.40641	0.46348	-1.04124
C	-5.36972	0.96697	0.33193

C -3.86960 0.99329 0.79006
C -3.17155 2.34517 0.50023
C -3.56423 3.45514 1.49405
C -3.33788 3.00808 2.95026
C -4.08873 1.69693 3.24718
C -3.68172 0.58354 2.26426
O 1.84035 -3.12512 1.36246
C 1.70153 -2.29934 2.30189
O 0.90268 -1.28185 2.35386
C 2.56093 -2.47842 3.60800
C 1.59263 -2.79232 4.77342
C 3.56709 -3.63128 3.43774
C 3.30734 -1.15946 3.90940
C 3.12575 -0.01775 -1.49700
O 2.69147 -0.58062 -2.49469
O 3.86292 -0.60326 -0.52618
C 4.05818 -2.04338 -0.62920
O 5.15198 1.98479 -1.79952
C 6.45949 2.51841 -1.50941
H -2.60633 0.32023 2.38002
H -4.24744 -0.34720 2.45636
H -5.18256 1.88278 3.18874
H -3.88863 1.36246 4.28192
H -2.25508 2.84421 3.11398
H -3.65122 3.80372 3.65239
H -2.97892 4.36486 1.27324
H -4.63241 3.72812 1.36062
H -3.38761 2.64975 -0.54078
H -2.08364 2.15360 0.55966
H -5.95539 0.28181 0.97740
H -5.84430 1.96070 0.32443
H -2.13878 -1.93125 -4.18953
H -2.78404 -3.57069 -3.79695
H -2.92973 -4.08806 -1.14889
H -1.47521 -3.57227 -0.24970
H -1.15088 -5.88398 -1.15278
H -1.48492 -5.33526 -2.80461
H 0.86838 -4.39327 -1.04121
H 0.98945 -5.49076 -2.44395
H 0.18043 -3.66322 -3.95994
H 1.60018 -3.09755 -3.05464
H 0.18347 -1.91525 -1.38728
H -0.17026 -1.33060 -3.02853
H 0.21411 3.53235 1.52700
H 1.05264 4.74063 -3.28187
H -0.37286 6.22099 -1.80903
H 1.68471 1.53890 -2.94974
H 3.06410 2.65593 -3.03361
H 1.12661 0.82790 0.04396
H 2.38532 1.66737 0.98826
H 6.40179 3.59431 -1.27442
H 6.91145 1.99739 -0.64937
H 7.05489 2.35262 -2.41874
H 3.26871 -2.54295 -0.03729
H 4.02663 -2.34944 -1.68569
H 5.05041 -2.23686 -0.19418
H 4.14673 -3.78516 4.36884
H 3.04848 -4.56901 3.18120
H 4.27674 -3.41963 2.62000
H 2.14202 -2.88924 5.73042
H 0.84566 -1.98817 4.87050
H 1.05246 -3.73948 4.59444
H 3.88891 -1.23888 4.84884
H 4.01002 -0.90563 3.09571
H 2.58751 -0.33129 4.00303

R = CO_2Me (8c)

III^{CO2Me}
100
BP86
SCF = -2529.11499536
H(0 K)= -2528.310796
H(413 K)= -2528.212523
G(413 K)= -2528.467372
B97D (1,2-C6Cl2H4) = -
2528.66929103
Low Freq. = 10.2518cm-1,
13.4484cm-1

100
III^{CO2Me}
C 1.53594 1.24334 0.86954
C 0.16833 1.16729 1.25866
C -0.30561 2.02846 2.27756
C 0.54440 2.97339 2.85839
C 1.87683 3.10788 2.45965
C 2.35089 2.23166 1.47339
Pd -1.29489 -0.02172 0.58058
O -3.07827 -1.37447 0.57882
C -2.96486 -1.46875 1.85433
C -4.01908 -2.20947 2.69331
C -4.88149 -1.12353 3.39236
F 0.04723 3.80890 3.81513
C 2.12295 0.30637 -0.17622
C 3.43684 -0.43426 0.23346
C 4.65256 0.51001 0.04243
O 4.78243 1.27135 -0.90682
C -1.07765 0.57593 -1.29277
N -1.18899 1.80770 -1.89980
C -1.06942 1.73642 -3.29412
C -0.87648 0.41982 -3.59379
N -0.86767 -0.25724 -2.37097
C -1.43743 3.22675 -1.50376
C -1.88003 3.76551 -2.90800
O -1.20422 2.94344 -3.90950
C -0.13394 3.91063 -1.02302
C -0.37767 5.36619 -0.57177
C -1.49486 5.45789 0.48452
C -2.79284 4.80570 -0.02795
C -2.55904 3.34177 -0.45512
O -0.75148 -0.40095 -4.66912
C -0.34612 -1.68716 -4.08378
C -0.95582 -1.72616 -2.64813
C -0.13785 -2.57603 -1.66586
C -0.32596 -4.08624 -1.91493
C -1.81148 -4.48868 -1.88817
C -2.61953 -3.66302 -2.90578
C -2.44891 -2.14963 -2.66235
O -1.97406 -0.88612 2.44722
C -4.90832 -3.08781 1.79144
C -3.31306 -3.07185 3.76453

```
C    3.69348 -1.55747 -0.81299
O    3.12982 -1.67010 -1.89085
C    3.38790 -1.01614  1.66693
O    4.71133 -2.36572 -0.40762
C    5.02007 -3.44369 -1.32319
O    5.58171  0.37642  1.02805
C    6.76220  1.19746  0.85140
H   -2.26507  2.73753  0.42194
H   -3.48407  2.88990 -0.85783
H   -3.18762  5.39047 -0.88375
H   -3.57639  4.83525  0.74992
H   -1.16821  4.94579  1.40905
H   -1.67935  6.51338  0.75449
H    0.56613  5.77990 -0.17521
H   -0.64601  5.99867 -1.44215
H    0.61507  3.87106 -1.83492
H    0.26922  3.32725 -0.17957
H   -2.97438  3.65760 -3.04098
H   -1.58244  4.80740 -3.09037
H    0.75824 -1.72024 -4.03863
H   -0.72757 -2.47466 -4.74819
H   -2.98498 -1.56600 -3.43363
H   -2.88159 -1.88622 -1.68141
H   -3.69317 -3.91774 -2.85286
H   -2.29355 -3.92670 -3.93244
H   -2.21771 -4.30804 -0.87420
H   -1.92414 -5.56911 -2.09234
H    0.11314 -4.36622 -2.89435
H    0.24536 -4.64022 -1.15026
H   -0.47599 -2.32918 -0.64084
H    0.92734 -2.30113 -1.72625
H   -1.33905  1.96838  2.63328
H    3.39126  2.33837  1.14867
H    2.51617  3.87014  2.91275
H    1.37354 -0.46193 -0.41055
H    2.34207  0.84232 -1.11607
C    2.27108 -2.03114  1.84420
H    4.34007 -1.52389  1.89144
H    3.26794 -0.20790  2.40311
H    7.27762  0.93410 -0.08576
H    6.49074  2.26454  0.81812
H    7.39817  0.98352  1.72076
H    4.16171 -4.12923 -1.40371
H    5.25966 -3.04732 -2.32247
H    5.88678 -3.95852 -0.88706
H   -5.68454 -3.58470  2.39963
H   -5.40380 -2.48636  1.01340
H   -4.31719 -3.86967  1.28564
H   -4.06325 -3.55751  4.41306
H   -2.70446 -3.86683  3.29902
H   -2.65303 -2.45405  4.39331
H   -5.64997 -1.60360  4.02366
H   -4.25756 -0.47825  4.03189
H   -5.39621 -0.48768  2.65167
O    1.83622 -2.03921  3.12767
O    1.84593 -2.77461  0.96353
C    0.75507 -2.96393  3.41582
H    0.72867 -3.04825  4.51086
H   -0.19163 -2.54221  3.04179
H    0.94865 -3.94180  2.94854
```

TS(III^{CO2Me}-IV^{CO2Me})1

```
100
BP86
SCF  =          -2529.09148266
H(0 K)=         -2528.288795
H(413 K)=       -2528.191339
G(413 K)=       -2528.444816
B97D  (1,2-C6Cl2H4)  =        -
2528.65070039
Low   Freq.   =   -24.6515cm-1,
13.6941cm-1

100
```

TS(III^{CO2Me}-IV^{CO2Me})1

```
C    2.32686 -1.69599  2.53098
C    3.22171 -1.90288  1.29424
C    2.84547 -3.19658  0.53012
C    2.81631 -4.42912  1.45603
C    1.89778 -4.20149  2.67042
C    2.31395 -2.93750  3.44514
C    4.75135 -1.83714  1.63509
O    5.42311 -1.19610  0.50050
C    4.46492 -0.40365 -0.05102
N    3.17197 -0.73056  0.37727
C    2.21108  0.04559 -0.20862
N    2.94261  0.86697 -1.02523
C    4.31976  0.63051 -0.93221
O    5.05955  1.50250 -1.66856
C    4.08108  2.16056 -2.54249
C    2.70376  2.11809 -1.79999
C    2.52340  3.30749 -0.82212
C    2.18990  4.61973 -1.55994
C    0.96785  4.45534 -2.48171
C    1.19208  3.30768 -3.48313
C    1.50441  1.98449 -2.75597
Pd   0.28582  0.09622  0.11133
O    0.54396  1.77372  1.43095
C   -0.07795  1.44039  2.52648
C   -0.06479  2.45370  3.70038
C   -1.53399  2.86775  3.96609
C   -0.02570 -1.45634 -1.14219
C    0.80432 -1.74618 -2.25007
C    0.50688 -2.81118 -3.10651
C   -0.60201 -3.63584 -2.90623
C   -1.43103 -3.35031 -1.81358
C   -1.17339 -2.27909 -0.92585
C   -2.16467 -2.05216  0.20755
C   -3.23404 -0.91841 -0.07296
C   -3.88828 -1.21763 -1.43132
O   -3.79956 -0.16315 -2.28972
C   -4.35850 -0.41275 -3.60232
F    1.33482 -3.05361 -4.16199
C   -2.49687  0.44089  0.01506
H   -1.64730  0.43040 -0.71373
C   -4.26915 -1.00105  1.07652
O   -5.54635 -1.10641  0.63207
C   -6.53617 -1.18261  1.68523
O   -3.95591 -0.98481  2.25718
O   -0.69569  0.34674  2.65303
C    0.49215  1.72632  4.94793
C    0.78759  3.69620  3.38149
```

O -4.39541 -2.28935 -1.72930
H 1.29897 -1.47444 2.18466
H 2.66583 -0.80002 3.08166
H 3.31770 -3.09301 3.89111
H 1.62606 -2.75358 4.28846
H 0.85321 -4.08542 2.32210
H 1.91505 -5.08332 3.33566
H 2.48708 -5.30855 0.87491
H 3.84006 -4.66406 1.81154
H 3.55444 -3.34520 -0.30497
H 1.84788 -3.04301 0.08177
H 4.92383 -1.22005 2.53743
H 5.21347 -2.82583 1.76422
H 4.03187 1.59994 -3.49564
H 4.44867 3.17992 -2.72505
H 3.44472 3.41848 -0.22067
H 1.71034 3.04957 -0.11993
H 2.01257 5.41478 -0.81432
H 3.05973 4.95392 -2.16193
H 0.07226 4.23198 -1.87135
H 0.76324 5.39826 -3.02002
H 2.02362 3.56939 -4.16906
H 0.30047 3.16573 -4.11869
H 0.62941 1.67864 -2.14929
H 1.69298 1.16784 -3.47631
H 1.69517 -1.15160 -2.46711
H -2.32101 -3.96558 -1.64327
H -0.80450 -4.46826 -3.58530
H -2.71713 -2.98819 0.39089
H -1.66210 -1.77197 1.15033
C -3.22553 1.73799 -0.29501
H -2.10609 0.51974 1.05448
H -6.34934 -2.05625 2.32950
H -6.50923 -0.27219 2.30495
H -7.50165 -1.27841 1.17050
H -4.19007 0.51111 -4.17145
H -3.84908 -1.26545 -4.07751
H -5.43429 -0.63697 -3.52530
H 0.77276 4.39550 4.23715
H 1.83621 3.42176 3.17945
H 0.40380 4.22667 2.49507
H 0.44220 2.38806 5.83111
H -0.09093 0.81485 5.15301
H 1.54856 1.43746 4.80270
H -1.95622 3.40787 3.10031
H -2.15613 1.97908 4.15736
H -1.59006 3.53743 4.84300
O -4.51461 1.71198 0.14625
O -2.70983 2.71615 -0.81896
C -5.25064 2.93646 -0.08182
H -6.26109 2.74730 0.30478
H -4.77983 3.77680 0.45271
H -5.28298 3.17277 -1.15728

Int(III^{CO2Me}-IV^{CO2Me})
100
BP86
SCF = -2529.09554665
H(0 K)= -2528.292748
H(413 K)= -2528.194286
G(413 K)= -2528.449641
B97D (1,2-C6Cl2H4) = -2528.65791289
Low Freq. = 13.9397cm-1, 16.4234cm-1

100
Int(III^{CO2Me}-IV^{CO2Me})
C -1.60355 -2.19793 -0.78777
C -0.35847 -1.56977 -1.08908
C 0.45575 -2.14760 -2.08897
C 0.04363 -3.29877 -2.76876
C -1.16758 -3.93296 -2.48481
C -1.97831 -3.36437 -1.49299
Pd 0.14616 0.13969 -0.13036
C -2.34318 0.83512 -0.19984
C -2.99471 2.17832 -0.49280
O -4.10183 2.51042 -0.09111
F 0.85912 -3.82147 -3.72696
C -2.56594 -1.65786 0.25991
C -3.33108 -0.35063 -0.16995
C -4.46360 -0.09594 0.85934
O -5.65854 -0.23274 0.65493
C 2.01729 -0.43828 -0.02824
N 2.63066 -1.30068 0.83752
C 4.00453 -1.42344 0.59594
C 4.27751 -0.59669 -0.45634
N 3.05307 -0.02731 -0.82782
C 2.22618 -2.22097 1.93741
C 3.65617 -2.49249 2.52249
O 4.61632 -2.30485 1.43101
C 1.57930 -3.50242 1.35614
C 1.08108 -4.45268 2.46385
C 0.13639 -3.73262 3.44352
C 0.81337 -2.48805 4.04648
C 1.29740 -1.52171 2.94729
O 5.32659 -0.12744 -1.18403
C 4.69908 0.56612 -2.31377
C 3.29974 1.06073 -1.81915
C 2.23786 1.08780 -2.93271
C 2.43743 2.27441 -3.89741
C 2.47625 3.61460 -3.14103
C 3.57191 3.60047 -2.05986
C 3.38599 2.42244 -1.08250
O 0.67021 1.99674 0.80083
C 0.13576 2.12079 1.98982
O -0.65442 1.28913 2.49722
C 0.55814 3.40289 2.76638
C -3.96287 -0.51793 -1.56509
O -3.80962 0.27053 -2.48959
O -4.69081 -1.65569 -1.65469
C -5.33669 -1.85427 -2.93525
O -3.92274 0.23485 2.06102
C -4.89220 0.55093 3.08756
O -2.17411 2.98620 -1.21441
C -2.72833 4.29411 -1.49635
H 0.42950 -1.10628 2.40090
H 1.82020 -0.65268 3.38512
H 1.66778 -2.80522 4.67888
H 0.11792 -1.95019 4.71378
H -0.77970 -3.42159 2.90467
H -0.18493 -4.42249 4.24435

H	0.57698	-5.31710	1.99680
H	1.94140	-4.86865	3.02629
H	2.30654	-4.01023	0.69639
H	0.73023	-3.19375	0.72103
H	3.89374	-1.76835	3.32486
H	3.78882	-3.51840	2.89354
H	4.59019	-0.15183	-3.14933
H	5.37495	1.38198	-2.60520
H	4.21859	2.38205	-0.35578
H	2.45340	2.55043	-0.50234
H	3.56887	4.54559	-1.48856
H	4.56589	3.54320	-2.54846
H	1.49367	3.78900	-2.66202
H	2.64168	4.44817	-3.84699
H	3.38009	2.14450	-4.46685
H	1.62400	2.27047	-4.64400
H	1.24552	1.16363	-2.44496
H	2.24928	0.12926	-3.48217
H	1.42393	-1.71656	-2.35494
H	-2.93814	-3.83581	-1.25371
H	-1.45786	-4.83904	-3.02316
H	-3.31873	-2.42965	0.49087
H	-2.03732	-1.41567	1.19909
H	-1.60131	0.66485	-1.03091
H	-1.86602	0.92040	0.80866
H	-4.30035	0.78003	3.98332
H	-5.49218	1.42201	2.78053
H	-5.56357	-0.30455	3.26471
H	-4.58508	-1.94807	-3.73515
H	-5.91242	-2.78354	-2.82973
H	-6.00248	-1.00735	-3.16247
H	-2.96092	4.82698	-0.56078
H	-1.95122	4.82410	-2.06268
H	-3.64937	4.19760	-2.09254
C	-0.23189	3.50339	4.08503
C	2.07482	3.31071	3.06560
C	0.28295	4.64549	1.88742
H	2.41497	4.21172	3.60744
H	2.30311	2.43339	3.69669
H	2.65523	3.22464	2.13282
H	0.59731	5.56498	2.41374
H	0.82947	4.58065	0.93354
H	-0.79316	4.73864	1.65888
H	0.07482	4.40585	4.64410
H	-1.31577	3.56337	3.89465
H	-0.05844	2.62021	4.72012

TS(III^{CO2Me}-IV^{CO2Me})2

100
BP86
SCF = -2529.07911925
H(0 K)= -2528.280586
H(0 K)= -2528.280590
H(413 K)= -2528.183156
G(413 K)= -2528.434677
B97D (1,2-C6Cl2H4) = -2528.63568073
Low Freq. = -977.6248cm-1, 8.8535cm-1

100

TS(III^{CO2Me}-IV^{CO2Me})2

C	-1.46223	-2.28186	-0.76979
C	-0.25925	-1.58921	-1.08487
C	0.58714	-2.12430	-2.07978
C	0.24015	-3.30857	-2.74114
C	-0.92888	-4.01068	-2.44161
C	-1.77095	-3.47822	-1.45412
Pd	0.15254	0.13857	-0.12892
C	-2.11818	0.69280	-0.40991
C	-2.71949	2.05201	-0.60545
O	-3.79010	2.45222	-0.15810
F	1.08215	-3.79495	-3.69677
C	-2.43551	-1.75182	0.26303
C	-3.15885	-0.43389	-0.18359
C	-4.12517	-0.06236	0.96840
O	-3.78137	-0.00531	2.14053
C	2.06747	-0.38971	0.03894
N	2.67429	-1.24615	0.91952
C	4.06194	-1.31912	0.74827
C	4.35781	-0.46729	-0.27701
N	3.13357	0.06676	-0.69734
C	2.24957	-2.21858	1.96437
C	3.64837	-2.42195	2.64054
O	4.66079	-2.18880	1.60686
C	1.72734	-3.52170	1.30899
C	1.21576	-4.53126	2.35660
C	0.15808	-3.90012	3.28036
C	0.70483	-2.62995	3.95795
C	1.20436	-1.60886	2.91638
O	5.42343	0.04190	-0.95362
C	4.82295	0.71450	-2.11026
C	3.38941	1.16374	-1.67444
C	2.37273	1.16324	-2.82958
C	2.57489	2.35699	-3.78474
C	2.54380	3.69598	-3.02585
C	3.59586	3.71202	-1.90226
C	3.40761	2.52641	-0.93442
O	0.65867	1.98090	0.94307
C	-0.20483	2.31026	1.82417
O	-1.35149	1.73414	1.93778
C	0.14139	3.45076	2.80533
C	-3.93622	-0.68445	-1.49715
O	-3.87966	0.01419	-2.49915
O	-4.68581	-1.82322	-1.42495
C	-5.45173	-2.10384	-2.61830
O	-5.39869	0.12665	0.54022
C	-6.30852	0.58787	1.56404
O	-1.87782	2.86495	-1.33180
C	-2.38617	4.20019	-1.53434
H	0.35515	-1.25666	2.29835
H	1.63039	-0.71370	3.40480
H	1.53093	-2.90456	4.64513
H	-0.07273	-2.15689	4.58273
H	-0.73624	-3.63597	2.68362
H	-0.17212	-4.62909	4.04186
H	0.80283	-5.41260	1.83509
H	2.05916	-4.90737	2.97044
H	2.53056	-3.96421	0.69193
H	0.90627	-3.24888	0.62300
H	3.80117	-1.68634	3.45362
H	3.80735	-3.44008	3.02217

H 4.77085 -0.00695 -2.94831
H 5.48494 1.55062 -2.37507
H 4.21079 2.50918 -0.17454
H 2.45060 2.62633 -0.39203
H 3.54349 4.65609 -1.33125
H 4.60954 3.68418 -2.35100
H 1.53784 3.83932 -2.58672
H 2.71216 4.53608 -3.72332
H 3.54271 2.25652 -4.31672
H 1.79167 2.33110 -4.56241
H 1.35965 1.20905 -2.38223
H 2.43499 0.20667 -3.37909
H 1.52621 -1.63819 -2.35454
H -2.70084 -4.00194 -1.20473
H -1.16636 -4.93844 -2.96890
H -3.20095 -2.51457 0.48075
H -1.92551 -1.51045 1.21164
H -1.61491 0.48261 -1.37843
H -1.55702 1.08053 0.90446
H -7.29666 0.62020 1.08504
H -6.31089 -0.09933 2.42499
H -6.00788 1.59262 1.90078
H -4.78479 -2.23629 -3.48524
H -6.00029 -3.03163 -2.40403
H -6.15086 -1.27924 -2.83065
H -2.56267 4.70618 -0.57070
H -1.61236 4.72626 -2.11087
H -3.33446 4.17489 -2.09540
C -1.02742 3.71720 3.77379
C 1.40790 3.03462 3.59464
C 0.44716 4.72205 1.97483
H 1.70721 3.84689 4.27985
H 1.21891 2.13288 4.20298
H 2.24612 2.82449 2.91133
H 0.72951 5.55011 2.64829
H 1.27547 4.54393 1.27092
H -0.43620 5.04162 1.39586
H -0.76000 4.53815 4.46230
H -1.94163 4.00118 3.22909
H -1.26455 2.82314 4.37203

IV^{CO2Me}
100
BP86
SCF = -2529.09989137
H(0 K)= -2528.296356
H(413 K)= -2528.198428
G(413 K)= -2528.450911
B97D (1,2-C6Cl2H4) = -2528.65885418
Low Freq. = 8.4940cm-1, 17.9210cm-1

100
IV^{CO2Me}
C 0.25552 -1.84392 -2.27089
C -0.57743 -1.37975 -1.22938
C -1.86135 -1.97243 -1.09089
C -2.28684 -2.96471 -1.99600
C -1.46333 -3.41048 -3.04088
C -0.19677 -2.83407 -3.15172
Pd -0.02296 0.13244 -0.03118
C -2.00311 0.92207 -0.27598
C -3.17354 -0.04965 0.04498
C -3.63226 0.19165 1.48745
O -2.93165 0.60199 2.41879
C -2.76200 -1.57046 0.05016
F 0.63805 -3.24959 -4.15025
C 1.90950 -0.56801 0.09446
N 3.00988 -0.17807 -0.63844
C 4.17571 -0.88384 -0.31380
C 3.81024 -1.78649 0.64244
N 2.44790 -1.56496 0.87106
C 3.36296 0.95476 -1.54229
C 4.73239 0.39619 -2.05187
O 5.27816 -0.43851 -0.97779
C 2.33889 1.15977 -2.67341
C 2.66779 2.39532 -3.53639
C 2.78184 3.66667 -2.67668
C 3.83700 3.48143 -1.57124
C 3.53051 2.24524 -0.70330
O 4.31979 -2.80688 1.38970
C 3.29879 -3.04279 2.41289
C 1.92710 -2.60027 1.79946
C 1.24868 -3.74201 1.00059
C 0.62796 -4.80929 1.92370
C -0.34371 -4.18131 2.93939
C 0.35083 -3.07503 3.75560
C 0.96319 -1.99829 2.83778
O 0.59988 1.92594 1.22483
C 0.19643 2.44327 2.28419
O -0.86013 2.03587 2.97973
C 0.87923 3.65932 2.93903
C 1.26546 3.28947 4.39456
C 2.13236 4.07122 2.14859
C -0.14467 4.82605 2.95230
C -4.41051 0.13081 -0.87264
O -4.99969 -0.75085 -1.47618
O -4.79910 1.44109 -0.87463
C -5.94494 1.71673 -1.70927
O -4.90667 -0.20556 1.69412
C -5.38498 -0.09717 3.05896
H 0.16303 -1.47769 2.27323
H 1.49164 -1.22559 3.42582
H 1.14198 -3.52676 4.38808
H -0.36226 -2.59759 4.45103
H -1.20463 -3.74530 2.39583
H -0.75242 -4.95627 3.61264
H 0.11155 -5.56555 1.30667
H 1.42457 -5.35330 2.47071
H 1.98903 -4.19436 0.31593
H 0.46390 -3.28736 0.36953
H 3.54396 -2.43093 3.30291
H 3.34220 -4.11037 2.67098
H 4.58710 -0.23601 -2.94906
H 5.47612 1.17781 -2.26148
H 4.32567 2.08463 0.04870
H 2.58577 2.39813 -0.15355
H 3.88687 4.37484 -0.92299
H 4.84049 3.38526 -2.03356
H 1.79716 3.87632 -2.21738
H 3.03509 4.53701 -3.30862

H 3.61650 2.23327 -4.08805
H 1.88120 2.51573 -4.30141
H 1.33984 1.28685 -2.21235
H 2.29149 0.25102 -3.29951
H 1.26323 -1.44978 -2.41608
H -3.29087 -3.38874 -1.88349
H -1.78601 -4.17904 -3.74828
H -3.68300 -2.17542 0.05727
H -2.22279 -1.74778 1.00014
C -1.88645 1.49790 -1.64251
H -2.02099 1.77896 0.41358
H -1.43841 1.34878 2.51521
H -4.78382 -0.73176 3.72884
H -5.32806 0.94666 3.40435
H -6.42663 -0.44229 3.02633
H -6.13699 2.79298 -1.60016
H -5.72373 1.46660 -2.75918
H -6.81724 1.12807 -1.38143
H 2.59866 4.94848 2.62897
H 2.87586 3.25864 2.12024
H 1.87961 4.33970 1.11046
H 1.73127 4.16177 4.88475
H 0.38141 2.99075 4.97855
H 1.99411 2.46050 4.41643
H -0.44787 5.10163 1.92771
H -1.04774 4.55548 3.52107
H 0.31407 5.71328 3.42210
O -2.47918 0.72322 -2.61665
O -1.29534 2.54661 -1.92313
C -2.29418 1.19335 -3.96485
H -2.92213 0.54399 -4.59098
H -2.59742 2.24858 -4.06147
H -1.23743 1.10064 -4.26657

V^{CO2Me}.OPiv
99
BP86
SCF = -2528.56782081
H(0 K)= -2527.778494
H(413 K)= -2527.680633
G(413 K)= -2527.935082
B97D (1,2-C6Cl2H4) = -2528.17820559
Low Freq. = 6.9227cm-1, 16.1500cm-1

99
V^{CO2Me}.OPiv
C -1.14471 2.30731 2.37031
C -2.19558 2.66507 1.30644
C -1.70171 3.81723 0.39207
C -1.23280 5.04387 1.19982
C -0.17089 4.65723 2.24597
C -0.69479 3.54288 3.17245
C -3.61433 2.95844 1.89572
O -4.58804 2.53162 0.88302
C -3.94768 1.50897 0.23724
N -2.56890 1.49405 0.48019
C -1.90564 0.46733 -0.14093
N -2.93989 -0.13800 -0.82105
C -4.18851 0.44933 -0.58690
O -5.21208 -0.20779 -1.21353
C -4.53565 -1.06693 -2.18937
C -3.10222 -1.36855 -1.63358
C -3.07034 -2.62221 -0.72436
C -3.13169 -3.93423 -1.53148
C -2.02138 -3.98903 -2.59699
C -2.11349 -2.77260 -3.53562
C -2.03237 -1.45635 -2.73907
Pd 0.02518 -0.17127 -0.08090
O -0.46730 -1.88296 1.15228
C -1.06692 -1.80377 2.30287
C -1.00627 -3.11691 3.16721
C -0.05809 -2.80984 4.35295
C 0.73052 1.33976 -1.19918
C 0.07968 1.83622 -2.35405
C 0.65799 2.85991 -3.11384
C 1.87956 3.44506 -2.77489
C 2.53098 2.96307 -1.62605
C 1.97747 1.93347 -0.84169
C 2.70134 1.46594 0.40366
C 3.13195 -0.04596 0.37141
C 4.46827 -0.15613 -0.40485
O 4.90947 -1.45753 -0.40190
C 6.13257 -1.66189 -1.12944
F -0.00367 3.31147 -4.23090
C 2.01874 -1.00152 -0.13050
C 2.06638 -1.52349 -1.50848
O 1.50933 -2.54335 -1.93716
C 3.49116 -0.44181 1.82721
O 4.47571 0.38882 2.31612
C 4.86127 0.10477 3.67494
O 3.00594 -1.33248 2.50600
O -1.64921 -0.81055 2.79721
C -2.42209 -3.41219 3.70849
C -0.47237 -4.32287 2.37349
O 5.10875 0.75550 -0.90611
O 2.80829 -0.71990 -2.37437
C 2.75719 -1.11550 -3.75018
H -0.28188 1.87164 1.82796
H -1.52131 1.48896 3.00545
H -1.53799 3.93676 3.77837
H 0.08546 3.24200 3.89501
H 0.73390 4.29438 1.72105
H 0.13232 5.54441 2.83363
H -0.83714 5.80823 0.50631
H -2.09584 5.51449 1.71552
H -2.50831 4.09526 -0.31178
H -0.86522 3.42444 -0.21356
H -3.77751 2.36557 2.81608
H -3.80313 4.02440 2.09091
H -4.47334 -0.52309 -3.15313
H -5.15551 -1.96778 -2.31024
H -3.90471 -2.56167 -0.00079
H -2.12996 -2.58182 -0.14366
H -3.04609 -4.78885 -0.83582
H -4.12041 -4.03987 -2.02753
H -1.03238 -3.97577 -2.10238
H -2.08787 -4.92930 -3.17631
H -3.06123 -2.81711 -4.11379
H -1.29324 -2.79431 -4.27482
H -1.04093 -1.38636 -2.24769

H -2.12493 -0.57925 -3.40673
H -0.88500 1.43120 -2.67608
H 3.50169 3.38634 -1.34112
H 2.30170 4.24285 -3.39326
H 3.59347 2.08403 0.59425
H 2.02066 1.57204 1.26950
H 1.87932 -1.83965 0.56460
H 4.00613 0.22684 4.36040
H 5.23669 -0.92792 3.76996
H 5.65413 0.82852 3.91414
H 6.37199 -2.72904 -1.01411
H 5.99442 -1.41170 -2.19457
H 6.94499 -1.03375 -0.72566
H -0.42315 -5.22011 3.02149
H -1.12491 -4.55707 1.51479
H 0.53255 -4.11826 1.97365
H -2.40311 -4.24874 4.43313
H -2.82644 -2.51375 4.20125
H -3.11154 -3.69038 2.89029
H 0.95774 -2.57775 3.98996
H -0.43047 -1.93787 4.91640
H 0.00496 -3.67608 5.03980
H 3.47434 -0.46504 -4.27368
H 3.02787 -2.17835 -3.87390
H 1.74513 -0.96441 -4.16509

V^{CO2Me}
83
BP86
SCF = -2182.04312787
H(0 K)= -2181.384578
H(413 K)= -2181.303440
G(413 K)= -2181.520792
B97D (1,2-C6Cl2H4) = -2181.68271176
Low Freq. = 8.8186cm-1, 14.5324cm-1

83
V^{CO2Me}
C -1.39061 3.12090 1.36444
C -2.53936 2.73171 0.41439
C -2.23330 3.16218 -1.04272
C -1.85766 4.65383 -1.14319
C -0.69452 5.00639 -0.19803
C -1.02774 4.61501 1.25386
C -3.94006 3.23566 0.90818
O -4.92143 2.20221 0.56114
C -4.19326 1.05359 0.50437
N -2.81608 1.27482 0.46459
C -2.06975 0.12454 0.35539
N -3.06492 -0.82865 0.32879
C -4.35214 -0.30501 0.44304
O -5.31837 -1.25952 0.51490
C -4.64170 -2.49836 0.11248
C -3.10832 -2.31183 0.38769
C -2.68479 -2.79646 1.79802
C -2.63511 -4.33380 1.90071
C -1.74058 -4.94170 0.80452
C -2.20624 -4.49208 -0.59267
C -2.22588 -2.95410 -0.70031
Pd -0.04182 -0.23361 0.41617
C 1.95365 -0.80410 0.77163
C 3.03436 0.28800 0.59967
C 4.37084 -0.23576 0.01991
O 4.84794 -1.25134 0.80021
C 6.09573 -1.81543 0.34181
C 0.55473 0.40324 -1.37024
C -0.20272 0.08946 -2.51434
C 0.26433 0.49905 -3.77123
C 1.45421 1.21441 -3.92672
C 2.19266 1.52275 -2.77369
C 1.76281 1.13254 -1.48885
C 2.55847 1.51382 -0.26256
F -0.47410 0.18611 -4.87404
C 3.35026 0.86837 2.00321
O 4.45996 1.66029 1.94846
C 4.81726 2.28760 3.20244
O 2.69289 0.71334 3.02274
O 4.95958 0.20255 -0.95518
H -0.51639 2.49538 1.09453
H -1.66347 2.85063 2.40107
H -1.87042 5.23702 1.61877
H -0.17542 4.83200 1.92175
H 0.21398 4.46299 -0.52272
H -0.46070 6.08426 -0.26109
H -1.59647 4.89191 -2.18930
H -2.73445 5.28586 -0.89517
H -3.10642 2.93027 -1.68004
H -1.39440 2.53979 -1.40383
H -3.94431 3.36462 2.00746
H -4.26832 4.16588 0.42304
H -4.82744 -2.66000 -0.96623
H -5.09515 -3.31448 0.69296
H -3.36626 -2.36491 2.55438
H -1.67513 -2.38761 1.99392
H -2.27104 -4.61655 2.90433
H -3.65970 -4.75064 1.81841
H -0.69571 -4.61082 0.95334
H -1.74801 -6.04423 0.87494
H -3.21515 -4.90259 -0.80433
H -1.53738 -4.89817 -1.37130
H -1.19624 -2.56593 -0.56942
H -2.57697 -2.62524 -1.69590
H -1.13976 -0.46882 -2.45053
H 3.13630 2.07082 -2.86963
H 1.78870 1.51262 -4.92378
H 3.44223 2.11002 -0.53795
H 1.91845 2.13562 0.39200
C 2.02019 -2.08785 0.03121
H 1.82184 -1.03641 1.84410
H 4.01011 2.95369 3.54758
H 5.00401 1.52647 3.97657
H 5.73003 2.86125 2.99194
H 5.97387 -2.25083 -0.66302
H 6.88097 -1.04322 0.30008
H 6.35304 -2.59440 1.07261
O 2.72541 -2.01431 -1.14679
O 1.46035 -3.13459 0.38528
C 2.71606 -3.21968 -1.93648
H 3.37638 -3.01500 -2.79077
H 3.08473 -4.07817 -1.35171

```
H   1.69633 -3.44574 -2.28966
```

TS(V^{CO2Me}-VI^{CO2Me})

```
83
BP86
SCF  =          -2182.01997179
H(0 K)=         -2181.362950
H(0 K)=         -2181.362979
H(413 K)=       -2181.282506
G(413 K)=       -2181.499324
B97D  (1,2-C6Cl2H4)  =        -
2181.65158327
Low   Freq.   =   -269.5347cm-1,
2.8971cm-1
```

83

TS(V^{CO2Me}-VI^{CO2Me})

```
C  -1.66027  2.72187  1.68281
C  -2.78479  2.42275  0.67357
C  -2.49033  3.08197 -0.69775
C  -2.19491  4.58938 -0.57054
C  -1.05754  4.85647  0.43227
C  -1.37620  4.23238  1.80388
C  -4.20981  2.77554  1.22485
O  -5.13745  1.75656  0.72461
C  -4.35046  0.67017  0.48661
N  -2.98563  0.96503  0.49782
C  -2.17991 -0.10220  0.17163
N  -3.12500 -1.08454 -0.02430
C  -4.43855 -0.65941  0.17804
O  -5.35246 -1.66233  0.05973
C  -4.61267 -2.75595 -0.57956
C  -3.09095 -2.54830 -0.25891
C  -2.64194 -3.27766  1.03287
C  -2.50333 -4.79913  0.82981
C  -1.57225 -5.12706 -0.35195
C  -2.05298 -4.43419 -1.64051
C  -2.17568 -2.91049 -1.44487
Pd -0.15345 -0.27514 -0.00405
C   1.98983 -0.72768 -0.09192
C   2.82032  0.03982  0.97444
C   4.35555 -0.15697  0.83450
O   4.66500 -1.47436  1.00871
C   6.07415 -1.77563  0.89435
C   1.21081  0.82951 -1.06349
C   0.87536  0.90150 -2.43742
C   1.20610  2.06509 -3.14165
C   1.90503  3.13044 -2.56007
C   2.30930  3.00425 -1.21924
C   1.98464  1.86083 -0.47181
C   2.48824  1.57537  0.91798
F   0.83445  2.16158 -4.44963
C   2.46678 -0.51873  2.37791
O   3.11768  0.20591  3.33203
C   2.87191 -0.22822  4.69129
O   1.72403 -1.45410  2.63636
O   5.18395  0.72039  0.66152
H  -0.75664  2.18925  1.32473
H  -1.92144  2.28192  2.66288
H  -2.25216  4.74701  2.24939
H  -0.53881  4.39060  2.50677
H  -0.11985  4.41694  0.04074
H  -0.88423  5.94280  0.53637
H  -1.93970  4.99463 -1.56557
H  -3.10672  5.12990 -0.24415
H  -3.34258  2.90051 -1.37831
H  -1.61308  2.56418 -1.12791
H  -4.21876  2.74227  2.33127
H  -4.58597  3.74947  0.88003
H  -4.79075 -2.70546 -1.67067
H  -5.02097 -3.69574 -0.18056
H  -3.35295 -3.04724  1.84777
H  -1.66428 -2.84926  1.32467
H  -2.12889 -5.25787  1.76222
H  -3.50032 -5.24919  0.64649
H  -0.54719 -4.78111 -0.11719
H  -1.51337 -6.22011 -0.50211
H  -3.03169 -4.85921 -1.94479
H  -1.35542 -4.63624 -2.47185
H  -1.17570 -2.47469 -1.23928
H  -2.55165 -2.41733 -2.36002
H   0.35202  0.08823 -2.94596
H   2.91562  3.79435 -0.76167
H   2.15844  4.00800 -3.16021
H   3.38655  2.16020  1.16834
H   1.71341  1.79746  1.67505
C   2.59684 -1.35469 -1.31121
H   1.42545 -1.55625  0.41653
H   1.80465 -0.12358  4.94382
H   3.16634 -1.28191  4.81950
H   3.48631  0.42832  5.32199
H   6.43404 -1.53692 -0.11918
H   6.65681 -1.19240  1.62522
H   6.16142 -2.85229  1.09342
O   3.78636 -0.78903 -1.67808
O   2.06169 -2.25345 -1.95627
C   4.35206 -1.31900 -2.89696
H   5.29542 -0.77467 -3.04092
H   4.53302 -2.40239 -2.80711
H   3.67020 -1.14215 -3.74401
```

VI^{CO2Me}

```
83
BP86
SCF  =          -2182.06814282
H(0 K)=         -2181.408434
H(413 K)=       -2181.327496
G(413 K)=       -2181.544437
B97D  (1,2-C6Cl2H4)  =         -
2181.69927974
Low    Freq.   =   10.6106cm-1,
18.9643cm-1
```

83

VI^{CO2Me}

```
C   2.87903 -2.04785  1.61572
C   3.80954 -1.27556  0.66169
C   4.00846 -2.05070 -0.66532
C   4.46405 -3.50461 -0.43522
C   3.50453 -4.25114  0.50969
C   3.35060 -3.49722  1.84369
C   5.16325 -0.85299  1.32966
```

```
O   5.51765  0.46877  0.80302
C   4.32661  1.01051  0.42027
N   3.28742  0.08106  0.37447
C   2.09584  0.58759 -0.10378
N   2.45376  1.90182 -0.31970
C   3.78263  2.19117 -0.00551
O   4.10438  3.50310 -0.18541
C   3.00016  4.03238 -0.99490
C   1.74205  3.15086 -0.68899
C   0.93658  3.67630  0.52582
C   0.10125  4.92427  0.17875
C  -0.82168  4.65776 -1.02415
C  -0.00865  4.18654 -2.24425
C   0.83341  2.93880 -1.91376
Pd  0.37827 -0.39559 -0.53428
C  -2.71383 -0.18020 -0.43929
C  -2.97619 -0.36033  1.09977
C  -4.38888  0.04624  1.56343
O  -4.80357  1.17660  0.92999
C  -6.10840  1.65033  1.34135
C  -1.65702 -1.26595 -0.70170
C  -0.92892 -1.50300 -1.91375
C  -0.34427 -2.79610 -2.05717
C  -0.46465 -3.80338 -1.10321
C  -1.18093 -3.54348  0.09276
C  -1.76267 -2.29265  0.29659
C  -2.69415 -1.86012  1.40764
F   0.32504 -3.05832 -3.21298
C  -2.01070  0.61879  1.82839
O  -1.49406  0.08099  2.96066
C  -0.59486  0.95612  3.68450
O  -1.80711  1.77216  1.46840
O  -5.03873 -0.53961  2.41603
H   1.86900 -2.04052  1.15536
H   2.79772 -1.49807  2.57161
H   4.31974 -3.50247  2.38370
H   2.63015 -4.01540  2.50144
H   2.51128 -4.33345  0.02720
H   3.86268 -5.28123  0.68782
H   4.53536 -4.02257 -1.40825
H   5.48628 -3.52019 -0.00512
H   4.72841 -1.50409 -1.30198
H   3.03393 -2.03811 -1.18931
H   5.04742 -0.77153  2.42773
H   5.99942 -1.52748  1.09472
H   3.28216  3.95513 -2.06259
H   2.87611  5.08878 -0.71702
H   1.63600  3.88636  1.35680
H   0.25927  2.86822  0.85589
H  -0.48941  5.22128  1.06339
H   0.76651  5.78214 -0.04981
H  -1.55337  3.87544 -0.74669
H  -1.39943  5.56547 -1.27599
H   0.65160  5.00980 -2.58682
H  -0.67830  3.95712 -3.09216
H   0.17349  2.07740 -1.67735
H   1.45005  2.63014 -2.77794
H  -1.03879 -0.86780 -2.79876
H  -1.28980 -4.33668  0.84023
H  -0.00989 -4.77892 -1.29587
H  -3.64710 -2.41510  1.36399
H  -2.28112 -1.99830  2.41693
C  -3.90190 -0.41917 -1.37210
H  -2.35632  0.83694 -0.65059
H   0.30670  1.14558  3.08028
H  -1.08924  1.91289  3.91469
H  -0.34042  0.41357  4.60482
H  -6.87567  0.88325  1.15097
H  -6.10858  1.89342  2.41587
H  -6.29526  2.54868  0.73835
O  -4.74896 -1.38000 -0.89401
O  -4.04281  0.12569 -2.45729
C  -5.84504 -1.71025 -1.78057
H  -6.44423 -2.45550 -1.24052
H  -6.44425 -0.81417 -2.00700
H  -5.46200 -2.12805 -2.72526
```

TS(IV^{CO2Me}-VI^{CO2Me}).HOPiv

100
BP86
SCF =   -2529.05918656
H(0 K)=   -2528.258488
H(413 K)=   -2528.160014
G(413 K)=   -2528.421215
B97D (1,2-C6Cl2H4) = -2528.61324592
Low Freq. = -278.3649cm-1, 4.4984cm-1

100
TS(IV^{CO2Me}-VI^{CO2Me}).HOPiv

```
C  -1.17689 -1.43753  2.58837
C  -0.81442 -1.70478  1.24522
C  -1.16086 -2.94889  0.65768
C  -1.96899 -3.85328  1.36517
C  -2.41619 -3.55932  2.66486
C  -1.99615 -2.35824  3.25087
Pd -0.61077  0.02368  0.15916
C   0.97213 -1.46656  0.42628
C   0.89936 -2.57879 -0.66130
C   1.24403 -1.95505 -2.03518
O   1.86753 -0.91620 -2.24404
C  -0.52833 -3.23396 -0.67780
F  -2.39627 -2.07521  4.52245
C  -1.94324  1.53306 -0.18148
N  -1.84818  2.85352  0.19518
C  -2.95322  3.62649 -0.16141
C  -3.82231  2.76600 -0.77465
N  -3.17761  1.52884 -0.79252
C  -0.79295  3.71469  0.78745
C  -1.67846  4.97560  1.08282
O  -2.81844  4.94177  0.15842
C  -0.20583  3.08992  2.06749
C   0.93411  3.94621  2.65324
C   2.03821  4.19437  1.60928
C   1.46915  4.83471  0.33044
C   0.31863  3.99185 -0.25416
O  -5.08019  2.72557 -1.29791
C  -5.15059  1.42861 -1.97609
C  -4.10411  0.48468 -1.28889
C  -4.69765 -0.30167 -0.09238
C  -5.60626 -1.46081 -0.54607
```

C	-4.87169	-2.40038	-1.51970
C	-4.32295	-1.61878	-2.72825
C	-3.40597	-0.46293	-2.28177
O	3.23684	2.23551	-1.05524
C	4.24753	1.53504	-1.06720
O	4.23087	0.20758	-1.32719
C	5.67708	2.04607	-0.79438
C	6.56518	1.73358	-2.02539
C	5.63522	3.56436	-0.54219
C	6.23680	1.31086	0.45015
C	1.97459	-3.69837	-0.48134
O	1.75553	-4.89199	-0.37009
O	3.21845	-3.15044	-0.54790
C	4.30240	-4.09391	-0.38041
O	0.80045	-2.74220	-3.04377
C	1.13094	-2.27525	-4.37747
H	-2.50911	-0.86117	-1.76510
H	-3.04060	0.11887	-3.14799
H	-5.16944	-1.22090	-3.32450
H	-3.76249	-2.29000	-3.40347
H	-4.03133	-2.88522	-0.98599
H	-5.54535	-3.20796	-1.85856
H	-5.95700	-2.01660	0.34132
H	-6.51677	-1.06191	-1.03785
H	-5.24499	0.39810	0.56558
H	-3.84667	-0.69969	0.49074
H	-4.89820	1.57835	-3.04352
H	-6.18766	1.07429	-1.88640
H	-2.07086	4.94569	2.11728
H	-1.15490	5.92685	0.91137
H	-0.12568	4.48502	-1.13879
H	0.72232	3.01861	-0.58787
H	2.26123	4.92504	-0.43162
H	1.11874	5.86397	0.55145
H	2.50953	3.23211	1.33634
H	2.83105	4.83443	2.03797
H	0.53270	4.91751	3.00965
H	1.34710	3.43836	3.54263
H	0.17785	2.08374	1.80023
H	-1.01515	2.93798	2.80551
H	-0.85461	-0.52882	3.10212
H	-2.22717	-4.81664	0.91050
H	-3.03795	-4.25497	3.23400
H	-0.44024	-4.31341	-0.87598
H	-1.11383	-2.78698	-1.50041
C	1.72133	-1.63805	1.71454
H	1.37883	-0.51892	-0.02137
H	3.29457	-0.06924	-1.52742
H	0.65200	-1.30313	-4.57210
H	2.22118	-2.16713	-4.48684
H	0.74257	-3.04576	-5.05634
H	5.22118	-3.50866	-0.51687
H	4.26719	-4.53606	0.62790
H	4.23290	-4.90095	-1.12694
H	6.65516	3.94257	-0.35436
H	5.21918	4.10073	-1.41044
H	5.00821	3.80624	0.33091
H	7.59611	2.08450	-1.84217
H	6.59472	0.65172	-2.22904
H	6.18975	2.24461	-2.92898
H	5.61445	1.50319	1.34079
H	6.27146	0.22264	0.28471
H	7.26019	1.66576	0.66560
O	1.91761	-2.94880	2.05230
O	2.08048	-0.70464	2.42681
C	2.55975	-3.14872	3.33131
H	2.64780	-4.23774	3.44586
H	3.55192	-2.66952	3.35091
H	1.94326	-2.72282	4.13877

VI^{CO2Me}.HOPiv

100

BP86

SCF = -2529.10990178

H(0 K)= -2528.306183

H(413 K)= -2528.207460

G(413 K)= -2528.468543

B97D (1,2-C6Cl2H4) = -2528.66005599

Low Freq. = 7.8374cm-1, 10.0145cm-1

100

VI^{CO2Me}.HOPiv

C	1.78213	2.42018	1.49562
C	0.76745	2.51950	0.48744
C	1.07660	3.06178	-0.80534
C	2.36309	3.51402	-1.09636
C	3.38234	3.42579	-0.11463
C	3.08194	2.88165	1.13157
Pd	1.56987	0.45061	0.54811
C	-0.75679	2.50028	0.68949
C	-1.27554	2.49097	-0.79678
C	-1.48929	1.00346	-1.18895
O	-2.09449	0.20815	-0.46412
C	-0.17928	3.20996	-1.63529
F	4.05708	2.82334	2.07852
C	1.97726	-1.52478	0.37502
N	1.47981	-2.60079	1.07851
C	2.06148	-3.82297	0.74067
C	2.99933	-3.53373	-0.21274
N	2.90602	-2.15831	-0.42545
C	0.37390	-2.81435	2.04325
C	0.75534	-4.26535	2.49868
O	1.51937	-4.88012	1.40693
C	0.42569	-1.78934	3.19175
C	-0.77416	-1.92399	4.14915
C	-2.11074	-1.82816	3.39068
C	-2.18545	-2.87697	2.26601
C	-0.98622	-2.75848	1.30526
O	3.98635	-4.12644	-0.94231
C	4.37453	-3.10872	-1.92392
C	3.99619	-1.71076	-1.32389
C	5.13915	-1.08525	-0.48471
C	6.26947	-0.51398	-1.36233
C	5.72489	0.48663	-2.39829
C	4.62333	-0.15954	-3.25891
C	3.48403	-0.71719	-2.38352
O	-4.33268	-1.69591	-2.52369
C	-4.87296	-1.83839	-1.42756
O	-4.35137	-1.36231	-0.27285
C	-6.21606	-2.56112	-1.20951

C -7.23003 -1.55089 -0.61390
C -6.72514 -3.09344 -2.56230
C -6.00850 -3.73204 -0.21632
C -2.65301 3.16052 -0.99706
O -2.95535 3.85031 -1.95842
O -3.50154 2.84612 0.01691
C -4.83120 3.40545 -0.11823
O -1.02706 0.71106 -2.41175
C -1.19386 -0.67458 -2.83525
H 2.98212 0.10769 -1.83625
H 2.70896 -1.20821 -3.00030
H 5.06453 -0.97126 -3.87304
H 4.20817 0.57396 -3.97287
H 5.30077 1.36210 -1.86920
H 6.54340 0.86272 -3.03828
H 7.02598 -0.03435 -0.71595
H 6.79515 -1.33605 -1.88951
H 5.52762 -1.84139 0.22223
H 4.68482 -0.27698 0.11911
H 3.81416 -3.29550 -2.86023
H 5.45190 -3.23230 -2.10633
H 1.39800 -4.23645 3.39939
H -0.11575 -4.91025 2.68384
H -1.01447 -3.55471 0.53803
H -1.03315 -1.79069 0.77311
H -3.11771 -2.75767 1.68730
H -2.21882 -3.89195 2.71218
H -2.20658 -0.81736 2.94949
H -2.95856 -1.95167 4.08836
H -0.72383 -2.89350 4.68630
H -0.71003 -1.14004 4.92463
H 0.43606 -0.78269 2.72270
H 1.38502 -1.89249 3.73150
H 1.54620 2.27423 2.55480
H 2.59013 3.95680 -2.07209
H 4.39723 3.78179 -0.31149
H -0.45922 4.27186 -1.74339
H -0.09863 2.78935 -2.64774
C -1.14899 3.70690 1.54479
H -1.11502 1.61383 1.23039
H -3.50671 -0.86970 -0.47848
H -0.59494 -1.32405 -2.17685
H -2.25625 -0.96526 -2.80160
H -0.80562 -0.70102 -3.86185
H -5.38145 3.06343 0.76805
H -4.78482 4.50543 -0.15175
H -5.30909 3.04114 -1.04127
H -7.69257 -3.60636 -2.42409
H -6.86287 -2.27433 -3.28607
H -6.01131 -3.80745 -3.00382
H -8.19958 -2.05114 -0.44435
H -6.87175 -1.14829 0.34668
H -7.40209 -0.70552 -1.30244
H -5.27727 -4.46185 -0.60477
H -5.65078 -3.36916 0.76012
H -6.96445 -4.26227 -0.06091
O -1.23422 4.85494 0.80860
O -1.29114 3.66887 2.75819
C -1.51259 6.04703 1.58256
H -1.58978 6.86072 0.84917
H -2.45340 5.93370 2.14396
H -0.69541 6.24057 2.29554

TS(IV^{CO2Me}-VI^{CO2Me}).OPiv
99
BP86
SCF = -2528.51429762
H(0 K)= -2527.727552
H(413 K)= -2527.629878
G(413 K)= -2527.884279
B97D (1,2-C6Cl2H4) = -2528.12293801
Low Freq. = -278.7823cm-1, 10.9044cm-1

99
TS(IV^{CO2Me}-VI^{CO2Me}).OPiv
C -2.19044 2.53068 1.50440
C -2.85263 2.56137 0.11706
C -2.24265 3.67398 -0.77321
C -2.27952 5.05491 -0.08702
C -1.62256 5.01413 1.30598
C -2.24390 3.91194 2.18681
C -4.41256 2.65940 0.18501
O -4.93311 1.93793 -0.98110
C -3.98523 0.97810 -1.20557
N -2.76555 1.25294 -0.57307
C -1.79609 0.29635 -0.77901
N -2.47889 -0.57683 -1.60699
C -3.80621 -0.20497 -1.85468
O -4.49073 -1.12248 -2.60555
C -3.44182 -1.99718 -3.13204
C -2.25442 -1.95095 -2.11567
C -2.41913 -2.97934 -0.96785
C -2.11929 -4.41973 -1.42931
C -0.73038 -4.52878 -2.08521
C -0.58849 -3.53141 -3.24956
C -0.87865 -2.09185 -2.78461
Pd 0.02079 0.01894 0.10866
O -0.60134 -1.70073 1.56016
C -1.53597 -1.52951 2.43945
C -1.66919 -2.66253 3.53307
C -1.39324 -2.00409 4.90495
C 1.45605 1.34553 -0.59681
C 1.30484 2.70088 -0.15772
C 1.62190 3.74361 -1.02760
C 2.14667 3.55685 -2.31241
C 2.42211 2.22506 -2.69588
C 2.10932 1.15036 -1.86326
C 2.54877 -0.27135 -2.06178
C 3.07371 -0.74944 -0.64974
C 2.86047 -2.27182 -0.50157
O 2.95006 -2.66635 0.79407
C 2.75502 -4.07877 1.01524
F 1.38997 5.03464 -0.59704
C 2.26659 0.02765 0.44318
C 4.60758 -0.53328 -0.56556
O 4.96908 0.66029 -1.11081
C 6.36022 0.98390 -0.93418
O 5.41344 -1.36493 -0.16692
O -2.34303 -0.57179 2.52803
C -3.12536 -3.17832 3.50546

C -0.68845 -3.82457 3.29989
O 2.63908 -3.04337 -1.42913
H -1.13909 2.21811 1.35456
H -2.63749 1.72853 2.11560
H -3.29602 4.18029 2.42061
H -1.72245 3.85637 3.15980
H -0.54190 4.82394 1.17317
H -1.71354 6.00052 1.79957
H -1.76642 5.79306 -0.72933
H -3.32875 5.40395 0.01034
H -2.77722 3.70192 -1.74117
H -1.19518 3.39240 -0.98217
H -4.78765 2.16716 1.10296
H -4.79599 3.68834 0.12238
H -3.12153 -1.61186 -4.12101
H -3.88303 -2.99828 -3.24794
H -3.44446 -2.90152 -0.56117
H -1.72671 -2.68458 -0.15572
H -2.19387 -5.10099 -0.56197
H -2.88979 -4.76185 -2.15258
H 0.05399 -4.30354 -1.33884
H -0.54945 -5.56122 -2.43922
H -1.27998 -3.81569 -4.07079
H 0.43316 -3.57573 -3.66553
H -0.13055 -1.78676 -2.02589
H -0.79773 -1.37202 -3.62012
H 0.92009 2.93068 0.83872
H 2.92226 2.03352 -3.65423
H 2.38368 4.41395 -2.94737
H 1.70959 -0.93441 -2.33226
H 3.32155 -0.38538 -2.83993
H 1.61662 -0.70666 1.00720
C 3.01848 0.72255 1.51945
H 6.57820 1.09195 0.14080
H 7.00813 0.19915 -1.35923
H 6.50741 1.93971 -1.45713
H 1.69377 -4.34049 0.87576
H 3.37561 -4.66871 0.32212
H 3.05195 -4.25479 2.05838
H -0.80069 -4.59731 4.08621
H -0.86561 -4.30031 2.32042
H 0.35346 -3.46591 3.30647
H -3.31262 -3.89580 4.32790
H -3.81952 -2.32813 3.60111
H -3.34650 -3.69247 2.55215
H -1.55882 -2.71995 5.73355
H -0.34975 -1.64612 4.96829
H -2.06014 -1.13716 5.04039
O 2.15181 1.17230 2.49262
O 4.23297 0.91687 1.59572
C 2.79348 1.86904 3.57473
H 1.98348 2.14943 4.26321
H 3.52506 1.21920 4.08456
H 3.31930 2.76742 3.20970

VI^{CO2Me}.OPiv
99
BP86
SCF = -2528.56407337
H(0 K)= -2527.774499
H(413 K)= -2527.676644
G(413 K)= -2527.929749
B97D (1,2-C6Cl2H4) = -2528.17513332
Low Freq. = 13.5908cm-1, 16.1722cm-1

99
VI^{CO2Me}.OPiv
C -2.63065 1.54856 1.95457
C -3.28936 1.77792 0.58277
C -2.97155 3.19669 0.04632
C -3.32756 4.30281 1.05992
C -2.65713 4.05241 2.42365
C -3.00850 2.65230 2.96154
C -4.82961 1.48560 0.60570
O -5.18170 0.92929 -0.70081
C -4.03523 0.29223 -1.08936
N -2.89781 0.74894 -0.41107
C -1.74937 0.07108 -0.74858
N -2.21679 -0.76260 -1.74004
C -3.60123 -0.69498 -1.91963
O -4.08479 -1.63707 -2.78256
C -2.89451 -2.21260 -3.40559
C -1.66811 -1.92897 -2.46792
C -1.37562 -3.08276 -1.47767
C -0.74102 -4.30257 -2.17241
C 0.52523 -3.91102 -2.95693
C 0.21617 -2.78979 -3.96719
C -0.40552 -1.56249 -3.27169
Pd 0.11082 0.16272 0.08510
O -0.39421 -1.10167 1.78544
C -1.47861 -1.76130 2.04363
C -1.40861 -2.66592 3.33252
C -2.67295 -2.39757 4.17859
C 0.92168 1.23228 -1.41317
C 0.29013 2.33717 -2.02789
C 0.94154 3.06465 -3.03092
C 2.22071 2.73656 -3.48143
C 2.84868 1.62750 -2.88840
C 2.22521 0.87776 -1.87392
C 2.89792 -0.36262 -1.32731
C 3.19014 -0.35444 0.20690
C 3.37601 -1.83417 0.63055
O 3.47858 -1.98533 1.97987
C 3.68190 -3.34518 2.40976
F 0.29247 4.13438 -3.60035
C 2.03562 0.27604 1.04407
C 4.53992 0.34465 0.55611
O 5.20157 0.79170 -0.55712
C 6.37537 1.56650 -0.25157
O 5.00752 0.44989 1.68127
O -2.54969 -1.77545 1.38796
C -1.42863 -4.13609 2.84920
C -0.14312 -2.40170 4.16716
O 3.44538 -2.78719 -0.14094
H -1.53316 1.53154 1.80678
H -2.90704 0.54405 2.31483
H -4.09433 2.60999 3.19003
H -2.48624 2.46455 3.91672
H -1.55878 4.12467 2.30505
H -2.95343 4.83494 3.14748

H -3.02755 5.28515 0.65112
H -4.42735 4.35194 1.19790
H -3.49731 3.35371 -0.91367
H -1.88764 3.23246 -0.16436
H -5.05976 0.73407 1.38481
H -5.44875 2.38358 0.75036
H -2.74731 -1.72963 -4.39186
H -3.09054 -3.28626 -3.54931
H -2.30125 -3.34955 -0.93965
H -0.68401 -2.67842 -0.71567
H -0.50597 -5.07157 -1.41434
H -1.47023 -4.77084 -2.86723
H 1.29910 -3.55929 -2.24802
H 0.94606 -4.79204 -3.47709
H -0.47402 -3.17726 -4.74647
H 1.13545 -2.47986 -4.49592
H 0.31866 -1.12122 -2.56392
H -0.65078 -0.76899 -4.00085
H -0.72178 2.64114 -1.74631
H 3.85453 1.34035 -3.21880
H 2.69955 3.32769 -4.26780
H 2.22613 -1.22350 -1.48889
H 3.82466 -0.58456 -1.88040
H 1.81434 -0.29944 1.95154
C 2.25072 1.69714 1.37103
H 6.09621 2.45340 0.34001
H 7.10684 0.97200 0.32196
H 6.79388 1.86547 -1.22395
H 2.81583 -3.97342 2.14368
H 4.58471 -3.77442 1.94353
H 3.79582 -3.29152 3.50163
H -0.11410 -3.06951 5.05060
H 0.76672 -2.56438 3.56876
H -0.11141 -1.35771 4.52092
H -1.45344 -4.83507 3.70720
H -2.31635 -4.31534 2.22022
H -0.52976 -4.36652 2.24950
H -2.73254 -3.09476 5.03616
H -2.66788 -1.36832 4.58168
H -3.57188 -2.51457 3.55265
O 1.46768 2.10223 2.45359
O 3.00555 2.50066 0.81223
C 1.60570 3.49228 2.77368
H 1.03589 3.64310 3.70400
H 2.66416 3.76713 2.92222
H 1.19679 4.13370 1.97162

R = C(CH_2)$_2$ (8d)

III$^{C(CH2)2}$
98
BP86
SCF = -2378.61859629
H(0 K)= -2377.822899
H(413 K)= -2377.728881
G(413 K)= -2377.972517
B97D (1,2-C6Cl2H4) = -2378.19422104
Low Freq. = 10.1864cm-1, 12.6476cm-1

98
III$^{C(CH2)2}$
C -1.09084 -1.88679 0.46050
C 0.06903 -1.34531 1.08173
C 0.73066 -2.10523 2.07552
C 0.29053 -3.39423 2.38919
C -0.79959 -3.98124 1.74187
C -1.48051 -3.20699 0.79313
Pd 0.93251 0.43521 0.79080
O 1.81028 2.44645 1.27387
C 1.38744 2.28792 2.47743
C 1.68978 3.31659 3.57822
F 0.98012 -4.11478 3.32129
C -1.94067 -1.10122 -0.52273
C -3.31164 -0.58683 0.06253
C -4.21684 -1.77618 0.46119
O -4.63667 -2.02634 1.57748
C 1.39913 0.04464 -1.09959
N 2.14266 -0.94688 -1.70530
C 2.29098 -0.74802 -3.08483
C 1.62629 0.40487 -3.37861
N 1.07952 0.85403 -2.17318
C 2.89218 -2.18000 -1.31211
C 3.79104 -2.30382 -2.59089
O 3.04440 -1.70345 -3.69380
C 1.94234 -3.39504 -1.17339
C 2.69794 -4.66990 -0.74083
C 3.53306 -4.44116 0.53346
C 4.49017 -3.24607 0.36243
C 3.73117 -1.96416 -0.03962
O 1.40149 1.24234 -4.42532
C 0.39648 2.18223 -3.91556
C 0.61116 2.26165 -2.37182
C -0.67629 2.58350 -1.60019
C -1.08278 4.06357 -1.75907
C 0.05614 5.01389 -1.34704
C 1.33619 4.71317 -2.14756
C 1.75822 3.23747 -1.99710
O 0.71554 1.22579 2.77717
C -4.02530 0.19860 -1.05757
O -3.48908 0.69912 -2.03846
C -3.06892 0.29166 1.30106
C -4.11805 1.18286 1.92468
C -2.91807 1.79998 1.22836
O -5.36398 0.30113 -0.81843
C -6.10646 1.03140 -1.82569
O -4.48796 -2.55465 -0.63693
C -5.36360 -3.67427 -0.36805
H 3.04638 -1.66287 0.77206
H 4.42786 -1.12101 -0.19989
H 5.25256 -3.48932 -0.40559
H 5.04780 -3.05871 1.29716
H 2.85742 -4.25225 1.38783
H 4.10568 -5.35343 0.78024
H 1.96672 -5.48212 -0.58325
H 3.36506 -5.01489 -1.55661
H 1.42146 -3.55601 -2.13514
H 1.17428 -3.14829 -0.42296
H 4.73813 -1.74499 -2.45891
H 4.00726 -3.34311 -2.87416
H -0.60907 1.78576 -4.15181

```
H   0.55868  3.13759 -4.43340
H   2.64130  3.01668 -2.62482
H   2.03430  3.04215 -0.94636
H   2.16630  5.36084 -1.81379
H   1.16745  4.95957 -3.21553
H   0.26692  4.88009 -0.26869
H  -0.24909  6.06652 -1.48848
H  -1.37158  4.26572 -2.81058
H  -1.98643  4.25502 -1.15336
H  -0.48451  2.36496 -0.53127
H  -1.49037  1.91316 -1.92550
H   1.59394 -1.70420  2.61552
H  -2.34032 -3.64605  0.27545
H  -1.10516 -5.00199  1.98569
H  -1.38647 -0.21190 -0.85698
H  -2.16781 -1.70996 -1.41484
H  -2.40038 -0.21021  2.00802
H  -6.33406 -3.32173  0.01659
H  -5.48946 -4.18753 -1.33124
H  -4.91451 -4.34983  0.37801
H  -6.01535  0.53474 -2.80459
H  -7.14884  1.02452 -1.48046
H  -5.73090  2.06310 -1.91315
H  -5.08894  1.25906  1.42813
H  -4.16548  1.20759  3.01720
H  -3.08961  2.29528  0.26672
H  -2.12410  2.25021  1.83118
C   0.36274  3.70172  4.27635
C   2.63117  2.63253  4.60468
C   2.36992  4.56515  2.98483
H   2.16344  1.72621  5.02117
H   3.58925  2.34618  4.13714
H   2.85128  3.32759  5.43410
H  -0.14230  2.80924  4.67765
H   0.56302  4.39833  5.10940
H  -0.32677  4.20312  3.57476
H   1.71564  5.06959  2.25453
H   2.60282  5.28315  3.79081
H   3.30734  4.30312  2.46891
```

TS(III$^{C(CH2)2}$-IV$^{C(CH2)2}$)1

98
BP86
SCF = -2378.60125282
H(0 K)= -2377.806386
H(413 K)= -2377.713222
G(413 K)= -2377.954520
B97D (1,2-C6Cl2H4) = -2378.18087495
Low Freq. = -43.7013cm-1, 10.4867cm-1

98
TS(III$^{C(CH2)2}$-IV$^{C(CH2)2}$)1

```
C   0.25947 -2.22508 -1.68005
C  -0.46496 -1.38887 -0.79949
C  -1.79633 -1.76388 -0.45091
C  -2.32682 -2.96799 -0.96900
C  -1.60675 -3.79534 -1.84201
C  -0.31790 -3.39082 -2.19457
Pd  0.26401  0.33076 -0.05649
C  -2.75289  1.38307 -0.64528
C  -1.83241  1.49432 -1.87181
C  -3.07791  2.35582 -1.75031
C  -2.68323 -0.92630  0.45744
C  -3.57987  0.13855 -0.27820
C  -4.66825  0.60338  0.72515
O  -4.72708  0.31030  1.90898
F   0.41460 -4.16610 -3.04391
C   2.05876 -0.42875  0.01913
N   3.11514 -0.22036 -0.83170
C   4.29080 -0.84490 -0.39446
C   3.96127 -1.50675  0.75346
N   2.60716 -1.23189  0.98266
C   3.43813  0.70721 -1.95206
C   4.79066  0.04559 -2.37966
O   5.36781 -0.56003 -1.17574
C   2.37279  0.67063 -3.06259
C   2.65574  1.70389 -4.17176
C   2.80064  3.12400 -3.59484
C   3.89887  3.16733 -2.51682
C   3.63413  2.14161 -1.39663
O   4.50508 -2.32571  1.69324
C   3.54973 -2.28173  2.80412
C   2.14040 -1.96396  2.19355
C   1.37543 -3.24785  1.78709
C   0.81752 -4.00110  3.01162
C  -0.04810 -3.08260  3.89340
C   0.74113 -1.83344  4.32654
C   1.28764 -1.06365  3.10755
O   0.95618  2.24338  0.68240
C   0.09429  2.52151  1.61917
O  -0.88967  1.77066  1.87153
C   0.33866  3.82738  2.41663
C  -4.29553 -0.51668 -1.47294
O  -4.09144 -0.31586 -2.66129
O  -5.22105 -1.41830 -1.02282
C  -5.93975 -2.11088 -2.07124
O  -5.56109  1.42928  0.10663
C  -6.60498  1.93367  0.97218
H   0.44724 -0.66340  2.50805
H   1.88756 -0.19072  3.42276
H   1.57586 -2.13666  4.99130
H   0.10267 -1.15712  4.92108
H  -0.94273 -2.76567  3.32355
H  -0.41326 -3.63268  4.77909
H   0.23487 -4.87213  2.66343
H   1.64895 -4.41309  3.61889
H   2.04453 -3.89724  1.19328
H   0.54453 -2.94860  1.12425
H   3.86234 -1.48205  3.50250
H   3.59797 -3.25749  3.30742
H   4.61784 -0.75347 -3.12599
H   5.52865  0.76382 -2.76330
H   4.46759  2.13191 -0.66999
H   2.72226  2.41554 -0.83550
H   3.97052  4.17593 -2.07276
H   4.88212  2.97275 -2.99144
H   1.83677  3.43479 -3.14709
H   3.02381  3.84466 -4.40186
H   3.58296  1.43083 -4.71528
H   1.84109  1.66587 -4.91612
```

H 1.39372 0.88262 -2.59026
H 2.30660 -0.35010 -3.48064
H 1.28477 -1.99017 -1.97633
H -3.33900 -3.26291 -0.66834
H -2.02135 -4.72776 -2.23438
H -3.35700 -1.58420 1.02975
H -2.08187 -0.36536 1.19536
H -2.34593 1.83629 0.26689
H -6.17295 2.52076 1.79828
H -7.23430 2.56767 0.33285
H -7.19170 1.10335 1.39717
H -5.24420 -2.70136 -2.68884
H -6.65338 -2.76552 -1.55279
H -6.46713 -1.39205 -2.71810
H -1.86508 0.68993 -2.60807
H -0.85559 1.97663 -1.73792
H -3.89565 2.10289 -2.42830
H -2.96340 3.42180 -1.53038
C -0.78637 4.04353 3.44644
C 1.70508 3.70917 3.13629
C 0.38524 5.01267 1.42142
H -0.60658 4.97425 4.01400
H -1.76871 4.12085 2.95306
H -0.84219 3.20460 4.15834
H 1.92580 4.63755 3.69313
H 1.70187 2.87545 3.86054
H 2.51561 3.53368 2.41092
H 0.58725 5.95568 1.96041
H 1.17559 4.86249 0.66898
H -0.57726 5.12666 0.89231

Int(III$^{C(CH2)2}$-IV$^{C(CH2)2}$)
98
BP86
SCF = -2378.60467141
H(0 K)= -2377.809635
H(413 K)= -2377.715507
G(413 K)= -2377.958280
B97D (1,2-C6Cl2H4) = -2378.18472640
Low Freq. = 13.3168cm-1, 19.2923cm-1

98
Int(III$^{C(CH2)2}$-IV$^{C(CH2)2}$)
C 0.05078 -2.53519 -1.63329
C -0.62531 -1.56194 -0.86771
C -1.96571 -1.82726 -0.46546
C -2.58032 -3.03760 -0.85706
C -1.91252 -3.99586 -1.63371
C -0.59732 -3.71735 -2.01123
Pd 0.18533 0.22496 -0.41976
C -2.26520 1.37719 -0.78503
C -1.42980 1.31760 -2.11795
C -2.44445 2.41118 -1.86326
C -2.75781 -0.85059 0.38306
C -3.35980 0.36236 -0.40116
C -4.31141 1.11243 0.57081
O -4.36735 0.94519 1.77878
F 0.08644 -4.63001 -2.75793
C 1.77191 -0.66422 0.32069
N 2.98488 -0.82906 -0.30855
C 3.92736 -1.47403 0.50253
C 3.28530 -1.76482 1.67060
N 1.98706 -1.25765 1.53624
C 3.69009 -0.24115 -1.48721
C 4.94401 -1.17495 -1.45951
O 5.16467 -1.54397 -0.05973
C 2.88152 -0.36620 -2.79017
C 3.57200 0.33942 -3.97550
C 3.87620 1.81429 -3.65711
C 4.72960 1.93079 -2.38152
C 4.05148 1.23605 -1.18384
O 3.50077 -2.38535 2.86047
C 2.39698 -1.92991 3.70839
C 1.19792 -1.56322 2.76402
C 0.25963 -2.76953 2.51725
C -0.61774 -3.07969 3.74748
C -1.39376 -1.83714 4.22242
C -0.43902 -0.66120 4.49959
C 0.42484 -0.32977 3.26637
O 1.04529 2.17643 -0.09536
C 0.53227 2.85652 0.90141
O -0.43531 2.47926 1.60072
C 1.23914 4.21643 1.19394
C -4.20149 -0.16420 -1.57931
O -4.00626 0.01071 -2.77364
O -5.23919 -0.91221 -1.10025
C -6.09139 -1.48064 -2.12375
O -5.06759 2.02178 -0.10540
C -5.95544 2.80271 0.73136
H -0.21074 0.05470 2.44763
H 1.13903 0.48233 3.49115
H 0.21127 -0.91136 5.36312
H -1.00665 0.23959 4.78964
H -2.12173 -1.53628 3.44508
H -1.98047 -2.07607 5.12761
H -1.31069 -3.90246 3.49718
H 0.01273 -3.45538 4.57885
H 0.86556 -3.64924 2.23199
H -0.38210 -2.52840 1.65149
H 2.73462 -1.04014 4.27309
H 2.16822 -2.74853 4.40518
H 4.75926 -2.09796 -2.04240
H 5.86263 -0.68606 -1.81284
H 4.70625 1.26578 -0.29313
H 3.11474 1.76120 -0.91830
H 4.90995 2.99042 -2.12801
H 5.72885 1.48819 -2.57024
H 2.92380 2.35961 -3.51024
H 4.38928 2.29235 -4.51077
H 4.51606 -0.18458 -4.22798
H 2.92725 0.25652 -4.86819
H 1.88666 0.08616 -2.60835
H 2.71077 -1.43324 -3.02059
H 1.08822 -2.39503 -1.94550
H -3.60914 -3.23493 -0.53291
H -2.38538 -4.93564 -1.93091
H -3.58868 -1.37193 0.88434
H -2.12264 -0.41786 1.17559
H -1.74491 1.74637 0.11621
H -5.37768 3.38491 1.46654

H -6.49348 3.46895 0.04369
H -6.65744 2.14566 1.26905
H -5.51722 -2.17155 -2.76113
H -6.88123 -2.01672 -1.58047
H -6.52055 -0.68618 -2.75443
H -1.71117 0.54659 -2.83511
H -0.37609 1.63104 -2.12641
H -3.35523 2.35293 -2.46238
H -2.07626 3.41941 -1.65266
C 0.39543 5.04153 2.18481
C 2.62293 3.90675 1.81808
C 1.43355 5.00467 -0.12131
H 0.90604 5.99279 2.42071
H -0.59592 5.27662 1.76297
H 0.22939 4.48588 3.12069
H 3.16060 4.84505 2.04581
H 2.51497 3.34265 2.76126
H 3.24290 3.31054 1.12870
H 1.95930 5.95680 0.07518
H 2.02142 4.42169 -0.84735
H 0.46227 5.24848 -0.58709

TS(III$^{C(CH2)2}$-IV$^{C(CH2)2}$)2
98
BP86
SCF = -2378.58137032
H(0 K)= -2377.796362
H(0 K)= -2377.791084
H(413 K)= -2377.698022
G(413 K)= -2377.937458
B97D (1,2-C6Cl2H4) = -2378.15466926
Low Freq. = -102.8943cm-1, 11.1539cm-1

98
TS(III$^{C(CH2)2}$-IV$^{C(CH2)2}$)2
C 0.39697 -2.26618 -1.98070
C -0.44812 -1.55821 -1.10219
C -1.73627 -2.08993 -0.82435
C -2.14243 -3.29670 -1.42822
C -1.30426 -3.99904 -2.30996
C -0.04396 -3.45848 -2.57138
Pd 0.06438 0.20380 -0.27597
C -1.96990 0.85569 -1.02350
C -1.69620 0.89496 -2.55512
C -2.31759 2.08475 -1.92142
C -2.65397 -1.35064 0.11793
C -3.16125 0.01687 -0.45274
C -3.75737 0.80749 0.74453
O -3.52840 0.59561 1.92683
F 0.79839 -4.11775 -3.41928
C 1.87644 -0.52608 0.23709
N 3.08894 -0.30842 -0.37879
C 4.15800 -0.94486 0.26448
C 3.61155 -1.62259 1.31462
N 2.23983 -1.34526 1.27687
C 3.63147 0.62569 -1.40503
C 5.02895 -0.05362 -1.58318
O 5.36770 -0.66056 -0.29412
C 2.79272 0.63116 -2.69446
C 3.29486 1.67260 -3.71469
C 3.35937 3.08148 -3.09770
C 4.23907 3.08476 -1.83437
C 3.75126 2.04924 -0.80134
O 3.96330 -2.45905 2.32928
C 2.80494 -2.43590 3.22547
C 1.54850 -2.11915 2.34401
C 0.91460 -3.40372 1.75435
C 0.13609 -4.20552 2.81668
C -0.91332 -3.33176 3.52825
C -0.26393 -2.07820 4.14380
C 0.50419 -1.26321 3.08397
O 0.70183 2.09150 0.69037
C -0.21124 2.87703 1.09614
O -1.45890 2.72031 0.79856
C 0.17915 4.09335 1.96277
C -4.34357 -0.25992 -1.41623
O -4.45186 0.03202 -2.59869
O -5.33767 -0.92276 -0.74131
C -6.51382 -1.20473 -1.53302
O -4.55725 1.81963 0.30558
C -5.07467 2.67356 1.35301
H -0.20010 -0.86665 2.32688
H 1.00322 -0.38733 3.53709
H 0.42402 -2.38170 4.95939
H -1.03004 -1.43377 4.60911
H -1.68328 -3.01795 2.79762
H -1.43466 -3.91522 4.30830
H -0.34340 -5.07437 2.33228
H 0.83478 -4.62310 3.56987
H 1.70825 -4.02167 1.29596
H 0.23188 -3.10058 0.94095
H 2.96213 -1.64270 3.98177
H 2.75933 -3.41734 3.71785
H 4.98121 -0.85254 -2.34831
H 5.83451 0.65258 -1.82864
H 4.43335 2.01175 0.06811
H 2.75479 2.33219 -0.41719
H 4.24900 4.08610 -1.36787
H 5.28895 2.87371 -2.12189
H 2.33536 3.40862 -2.83156
H 3.74312 3.80723 -3.83700
H 4.30068 1.38583 -4.08297
H 2.63104 1.66313 -4.59706
H 1.74683 0.85725 -2.40514
H 2.78715 -0.38072 -3.13795
H 1.40460 -1.91504 -2.21714
H -3.13693 -3.69967 -1.19999
H -1.61086 -4.93658 -2.78125
H -3.52236 -1.96756 0.39989
H -2.11904 -1.09798 1.04947
H -1.54098 1.79939 0.02366
H -4.24481 3.19045 1.85955
H -5.72989 3.39456 0.84519
H -5.64002 2.08369 2.09198
H -6.25935 -1.83821 -2.39779
H -7.20281 -1.72894 -0.85655
H -6.96614 -0.26994 -1.90171
H -2.33028 0.27466 -3.19066
H -0.64602 0.94092 -2.85992
H -3.38163 2.25518 -2.10684

H -1.72907 2.99645 -1.77125
C -1.07032 4.79830 2.52641
C 1.08567 3.60471 3.11755
C 0.97407 5.07028 1.05761
H -0.76451 5.66548 3.13765
H -1.72716 5.15706 1.71829
H -1.65892 4.11798 3.16350
H 1.42299 4.46546 3.72094
H 0.54105 2.91482 3.78510
H 1.97017 3.07886 2.72588
H 1.29245 5.94916 1.64502
H 1.87305 4.58349 0.64611
H 0.35602 5.42985 0.21681

IV$^{C(CH2)2}$
98
BP86
SCF = -2378.59432176
H(0 K)= -2377.799633
H(413 K)= -2377.706052
G(413 K)= -2377.946192
B97D (1,2-C6Cl2H4) = -2378.17034509
Low Freq. = 11.4457cm-1, 19.5931cm-1

98
IV$^{C(CH2)2}$
C 0.37400 -0.83062 3.25021
C 1.26631 -1.91239 2.61514
C 0.43326 -3.15065 2.19750
C -0.42201 -3.69487 3.35907
C -1.31332 -2.59716 3.96867
C -0.47211 -1.38591 4.41311
C 2.49279 -2.30084 3.50901
O 3.59953 -2.61460 2.60285
C 3.33345 -1.87530 1.48857
N 2.02339 -1.38504 1.45169
C 1.72354 -0.67024 0.31543
N 2.92489 -0.74527 -0.36088
C 3.91877 -1.44878 0.33285
O 5.12583 -1.44396 -0.30046
C 4.81170 -0.98843 -1.65603
C 3.54094 -0.08314 -1.54485
C 3.89374 1.38127 -1.18633
C 4.47557 2.16285 -2.38032
C 3.54003 2.10098 -3.60100
C 3.23880 0.64039 -3.98138
C 2.64537 -0.14673 -2.79507
Pd -0.08626 0.17237 -0.28188
O 0.79799 2.20242 0.30585
C 0.41266 3.12240 1.05668
C 1.27873 4.35517 1.38827
C 0.53039 5.61729 0.88387
C -0.79511 -1.58266 -0.95188
C 0.01730 -2.46976 -1.69297
C -0.50980 -3.66432 -2.19836
C -1.83688 -4.04371 -1.98792
C -2.64506 -3.17259 -1.24122
C -2.14596 -1.96021 -0.72610
C -3.03206 -1.07859 0.11950
C -3.21739 0.38434 -0.43721
C -4.35082 0.49006 -1.47222
O -4.64227 -0.70210 -2.06153
C -5.63457 -0.62219 -3.11093
F 0.31156 -4.49069 -2.91260
C -1.88973 0.94432 -1.02977
C -1.89860 2.38707 -1.53498
C -1.83760 1.25053 -2.52977
C -3.65884 1.18004 0.79574
O -4.95685 0.95765 1.10155
C -5.44297 1.62073 2.29495
O -2.94157 1.86507 1.53040
O -0.76504 3.17248 1.65732
C 1.44969 4.43537 2.92769
C 2.65623 4.25612 0.71218
O -4.89569 1.54318 -1.78879
H -0.28591 -0.44143 2.44803
H 1.00253 0.01575 3.58291
H 0.18859 -1.68563 5.25208
H -1.12171 -0.58366 4.80610
H -2.05175 -2.26753 3.21180
H -1.89024 -2.99822 4.82154
H -1.03608 -4.53671 2.99343
H 0.23057 -4.11446 4.15141
H 1.11327 -3.93063 1.80931
H -0.21924 -2.84748 1.35893
H 2.79332 -1.44957 4.15068
H 2.32184 -3.19224 4.12906
H 4.60570 -1.87431 -2.28759
H 5.69889 -0.45947 -2.03221
H 4.59909 1.38216 -0.33458
H 2.96258 1.86163 -0.83865
H 4.65465 3.20996 -2.07662
H 5.46754 1.75368 -2.65968
H 2.58996 2.61682 -3.36047
H 3.98587 2.63772 -4.45769
H 4.17200 0.15210 -4.32856
H 2.53517 0.59585 -4.83127
H 1.65569 0.26843 -2.51724
H 2.47721 -1.20059 -3.07856
H 1.07196 -2.25762 -1.87873
H -3.69215 -3.43906 -1.05542
H -2.21450 -4.98644 -2.39283
H -4.02395 -1.53483 0.27310
H -2.55793 -0.97265 1.11446
H -1.42650 2.44873 1.38504
H -5.32296 2.71122 2.20312
H -6.50513 1.35074 2.36249
H -4.89356 1.27125 3.18347
H -5.31139 0.07620 -3.89920
H -5.72172 -1.64276 -3.50744
H -6.59943 -0.27510 -2.70686
H -2.71903 1.10116 -3.16501
H -0.89425 1.04092 -3.04473
H -2.84332 2.94326 -1.49631
H -1.00366 2.99933 -1.38050
H 2.04595 5.32728 3.18730
H 0.47424 4.50661 3.43281
H 1.97989 3.54897 3.31728
H 3.25482 5.14903 0.96177
H 3.20818 3.36521 1.05165

H   2.56212 4.20165 -0.38444
H   1.12220 6.51823 1.12171
H   0.38572 5.58587 -0.20988
H -0.45753 5.70975 1.36060

V^C(CH2)2.OPiv
97
BP86
SCF =   -2378.05698992
H(0 K)=   -2377.275741
H(413 K)=   -2377.182464
G(413 K)=   -2377.421787
B97D (1,2-C6Cl2H4) = -2377.68646288
Low Freq. = 13.1852cm-1, 21.5636cm-1

97
V^C(CH2)2.OPiv
C   2.90608 0.79238 2.87401
C   2.38647 -0.63998 2.59498
C   0.97343 -0.85173 3.16573
C   0.86439 -0.43235 4.64534
C   1.34701 1.01368 4.85921
C   2.79452 1.17721 4.36192
C   3.42632 -1.71848 3.03594
O   4.40234 -1.82771 1.94306
C   3.66687 -1.52762 0.82993
N   2.44593 -0.90780 1.13029
C   1.68576 -0.60187 0.02185
N   2.50896 -1.05863 -0.98101
C   3.71102 -1.61473 -0.53069
O   4.53330 -2.00866 -1.55134
C   3.65771 -2.02292 -2.72612
C   2.49430 -1.00713 -2.45949
C   2.83309 0.43364 -2.92204
C   2.75161 0.59609 -4.45242
C   1.38573 0.13787 -4.99615
C   1.08773 -1.31274 -4.57285
C   1.14667 -1.47098 -3.04155
Pd -0.23462 0.15582 -0.14602
O   0.71776 2.10586 -0.56939
C   1.06728 2.99802 0.29882
C   1.62193 4.34801 -0.29070
C   0.62071 5.45641 0.11477
C -1.01443 -1.71949 -0.16839
C -0.31027 -2.82690 0.37874
C -0.78363 -4.13682 0.26211
C -1.96710 -4.44869 -0.40731
C -2.68431 -3.37004 -0.94887
C -2.23610 -2.03656 -0.84027
C -3.11752 -0.94794 -1.40998
C -3.40387 0.16530 -0.35615
C -4.63056 0.94602 -0.85497
O -5.26002 1.61872 0.16912
C -6.37592 2.42505 -0.25249
F -0.04880 -5.15732 0.81960
C -2.14586 1.06157 -0.22522
C -3.78303 -0.48798 0.99226
O -4.85562 -1.34081 0.80654
C -5.25367 -2.04680 1.99473
O -3.26837 -0.32362 2.08436
O   0.98636 2.91454 1.55150
C   2.98877 4.63195 0.37182
C   1.77174 4.31325 -1.82186
O -5.02133 1.02703 -2.01407
H   2.29084 1.49733 2.28269
H   3.95293 0.86701 2.52340
H   3.47181 0.55516 4.98610
H   3.13206 2.22203 4.48693
H   0.69727 1.70108 4.28666
H   1.26703 1.29306 5.92698
H -0.18595 -0.54421 4.96837
H   1.46262 -1.11752 5.28325
H   0.67096 -1.90590 3.03420
H   0.27563 -0.25045 2.54923
H   3.98767 -1.44491 3.94104
H   2.93900 -2.70437 3.17246
H   3.25346 -3.04665 -2.85107
H   4.28032 -1.76032 -3.59468
H   3.84023 0.70241 -2.55145
H   2.11372 1.11114 -2.42396
H   2.94223 1.65192 -4.71778
H   3.55400 0.00616 -4.94344
H   0.59523 0.79932 -4.59290
H   1.35622 0.23474 -6.09773
H   1.81712 -1.99398 -5.06023
H   0.08972 -1.62332 -4.93081
H   0.35870 -0.85670 -2.55874
H   0.95073 -2.51344 -2.73353
H   0.63720 -2.69054 0.90527
H -3.62934 -3.56811 -1.47131
H -2.31078 -5.48422 -0.48845
H -2.63758 -0.43802 -2.26480
H -4.06607 -1.37803 -1.77145
C -2.19823 2.41494 -0.94581
C -2.26921 2.35058 0.57078
H -4.44714 -2.71614 2.33798
H -5.49815 -1.34478 2.80982
H -6.14023 -2.63335 1.71016
H -6.05355 3.19895 -0.96891
H -7.14951 1.80447 -0.73572
H -6.76838 2.88895 0.66408
H   2.14055 5.28799 -2.19700
H   2.48720 3.53392 -2.13451
H   0.80885 4.09324 -2.31033
H   3.36896 5.63117 0.08525
H   2.88603 4.58554 1.46763
H   3.74264 3.88328 0.06675
H   0.97887 6.45096 -0.21413
H -0.36960 5.27866 -0.34017
H   0.49417 5.46496 1.20939
H -3.23412 2.58055 1.03868
H -1.39816 2.66548 1.15374
H -3.10521 2.70461 -1.49338
H -1.27047 2.77020 -1.40372

V^C(CH2)2
81
BP86
SCF =   -2031.53923616
H(0 K)=   -2030.889637

H(413 K)= -2030.812663
G(413 K)= -2031.018960
B97D (1,2-C6Cl2H4) = -2031.19699272
Low Freq. = 7.5544cm-1, 16.7262cm-1

81
$\mathbf{V}^{C(CH2)2}$
C -3.08757 2.63988 -1.57484
C -3.42026 1.92182 -0.24158
C -2.72107 2.60921 0.94667
C -3.01281 4.12182 0.99936
C -2.64312 4.81386 -0.32585
C -3.36819 4.15415 -1.51327
C -4.96285 1.74269 -0.02251
O -5.34116 0.45930 -0.62008
C -4.19549 -0.27770 -0.58469
N -3.05552 0.48836 -0.33433
C -1.87594 -0.22585 -0.37199
N -2.35644 -1.48719 -0.64762
C -3.74629 -1.55696 -0.76188
O -4.20828 -2.82098 -0.96687
C -3.01076 -3.58990 -1.32216
C -1.78114 -2.85406 -0.68667
C -0.52207 -2.91395 -1.57167
C 0.14073 -4.30552 -1.54190
C 0.46141 -4.74649 -0.10134
C -0.80451 -4.72480 0.77456
C -1.47954 -3.33928 0.75425
Pd 0.06998 0.48284 -0.14618
C 1.91901 1.35970 -0.13008
C 1.87252 2.69323 -0.86676
C 2.12319 2.65603 0.63148
C 0.69251 -0.18716 1.62609
C 1.98316 -0.76559 1.73956
C 2.42701 -1.21148 2.99988
C 1.62234 -1.09967 4.14596
C 0.35559 -0.52838 4.00275
C -0.12388 -0.06984 2.76735
C 2.84362 -0.95373 0.50706
C 3.08003 0.36339 -0.33417
C 4.43672 0.96459 0.08693
O 4.83768 1.93106 -0.79745
C 6.08371 2.57834 -0.44998
F -0.44729 -0.40902 5.09981
C 3.13077 -0.07553 -1.81115
O 4.32501 -0.67950 -2.09127
C 4.44956 -1.18015 -3.44208
O 2.22326 0.01220 -2.62752
O 5.07339 0.68000 1.09075
H 0.18186 -2.14627 -1.19308
H -0.78319 -2.61654 -2.60367
H -0.52787 -5.05361 -2.01496
H 1.05837 -4.28406 -2.15614
H 1.21280 -4.05837 0.33231
H 0.91390 -5.75443 -0.09970
H -0.56193 -4.99475 1.81744
H -1.51133 -5.49971 0.41408
H -2.41899 -3.34898 1.33713
H -0.81457 -2.59136 1.22349
H -2.92598 -3.61239 -2.42558
H -3.15878 -4.60975 -0.93937
H -5.20007 1.70957 1.05815
H -5.57068 2.51704 -0.51216
H -3.65163 2.16244 -2.39692
H -2.01342 2.47003 -1.77966
H -3.06347 4.62104 -2.46667
H -4.45848 4.33266 -1.42108
H -1.54873 4.74153 -0.48028
H -2.88553 5.89053 -0.27801
H -4.08716 4.29225 1.21561
H -2.45581 4.57065 1.84048
H -1.63086 2.43179 0.83358
H -3.02504 2.11248 1.88609
H -1.12452 0.36787 2.71686
H 3.42862 -1.64788 3.08844
H 1.96383 -1.43843 5.12761
H 3.82321 -1.37955 0.77467
H 2.33703 -1.67959 -0.15720
H 3.68438 -1.94645 -3.64664
H 4.33480 -0.36211 -4.17116
H 5.45698 -1.61429 -3.50201
H 6.00493 3.07482 0.53059
H 6.90270 1.84249 -0.40812
H 6.26326 3.31526 -1.24471
H 1.32581 2.97723 1.30969
H 3.12689 2.93182 0.97855
H 0.90031 3.05434 -1.22961
H 2.71103 2.95253 -1.52296

$\mathbf{TS(V^{C(CH2)2}}$-$\mathbf{VI^{C(CH2)2})}$
81
BP86
SCF = -2031.52232604
H(0 K)= -2030.873334
H(413 K)= -2030.797176
G(413 K)= -2031.000317
B97D (1,2-C6Cl2H4) = -2031.17648129
Low Freq. = -253.9790cm-1, 7.8393cm-1

81
$\mathbf{TS(V^{C(CH2)2}}$-$\mathbf{VI^{C(CH2)2})}$
C -1.95395 3.47947 -0.60102
C -2.67660 2.70019 0.52686
C -1.94238 2.86644 1.87089
C -1.71154 4.34753 2.22992
C -0.96672 5.09010 1.10468
C -1.71792 4.95753 -0.23286
C -4.20327 3.04691 0.62859
O -4.92635 2.10143 -0.22416
C -4.10550 1.01528 -0.29627
N -2.80859 1.27231 0.15238
C -1.94044 0.20900 0.00727
N -2.78356 -0.73035 -0.54756
C -4.09719 -0.28293 -0.71910
O -4.94131 -1.24405 -1.18594
C -4.05341 -2.31153 -1.65099
C -2.70163 -2.17738 -0.86614
C -1.48106 -2.50903 -1.74473

C -1.37755 -4.02105 -2.02964
C -1.33565 -4.83523 -0.72276
C -2.55358 -4.52119 0.16572
C -2.68070 -3.00936 0.44057
Pd 0.05778 0.16536 0.43610
C   2.17728 0.37598 0.77794
C   2.21225 1.69467 1.59825
C   2.97289 0.50036 2.07933
C   1.34439 -1.36595 0.88654
C   2.09226 -2.00697 -0.13866
C   2.46170 -3.35304 -0.01159
C   2.10662 -4.09518 1.13059
C   1.40613 -3.43476 2.14852
C   1.03413 -2.08797 2.06700
C   2.53182 -1.09343 -1.25476
C   2.91008 0.28940 -0.59584
C   4.44390 0.34410 -0.41672
O   4.85036 1.62916 -0.17314
C   6.27463 1.77764 0.04362
F   1.06647 -4.13474 3.26957
C   2.50099 1.42888 -1.55192
O   3.18356 1.28731 -2.72900
C   2.88236 2.28794 -3.72872
O   1.68878 2.32164 -1.35845
O   5.21022 -0.60536 -0.44327
H -0.58070 -2.16397 -1.20213
H -1.53777 -1.92660 -2.68289
H -2.23851 -4.35109 -2.64690
H -0.47407 -4.21581 -2.63423
H -0.40757 -4.58628 -0.17358
H -1.29471 -5.91675 -0.94634
H -2.48208 -5.06037 1.12699
H -3.47452 -4.89550 -0.32622
H -3.59066 -2.78815 1.02849
H -1.81341 -2.66238 1.03210
H -3.88696 -2.17904 -2.73742
H -4.57265 -3.26371 -1.47030
H -4.56105 2.92455 1.66911
H -4.44853 4.05704 0.26959
H -2.54042 3.39172 -1.53425
H -0.98224 2.98139 -0.77831
H -1.15464 5.44926 -1.04559
H -2.68657 5.49323 -0.16315
H   0.04720 4.65982 0.99376
H -0.83553 6.15487 1.36920
H -2.68440 4.84577 2.41880
H -1.14741 4.41012 3.17762
H -0.97072 2.33937 1.77649
H -2.50663 2.34308 2.66432
H   0.49011 -1.63151 2.89843
H   3.05751 -3.82737 -0.80004
H   2.39211 -5.14274 1.25403
H   3.39037 -1.49413 -1.81460
H   1.70340 -0.92175 -1.96718
H   1.81820 2.24651 -4.01148
H   3.11079 3.29676 -3.34900
H   3.52055 2.04326 -4.58872
H   6.59665 1.19494 0.92160
H   6.83637 1.42972 -0.83765
H   6.43513 2.85130 0.21018
H   2.58573 -0.06768 2.93180
H   4.06594 0.51624 2.01983
H   1.30917 1.99979 2.13436
H   2.76520 2.51897 1.13715

VI$^{C(CH2)2}$
81
BP86
SCF =   -2031.57585946
H(0 K)=   -2030.924912
H(413 K)=   -2030.848250
G(413 K)=   -2031.052862
B97D (1,2-C6Cl2H4) =   -
2031.22584273
Low Freq. = 11.5955cm-1,
18.3597cm-1

81
VI$^{C(CH2)2}$
C -0.09668 3.61567 -0.79697
C -0.97505 3.31313 0.44274
C -0.10247 2.98948 1.66889
C   0.93608 4.09475 1.94640
C   1.81328 4.36498 0.70946
C   0.94630 4.71596 -0.51414
C -2.02704 4.44106 0.72156
O -3.23460 4.10344 -0.03626
C -3.18263 2.74562 -0.15133
N -1.92433 2.21413 0.14331
C -1.83694 0.84505 -0.01573
N -3.12424 0.56264 -0.43263
C -3.96138 1.67929 -0.49893
O -5.24904 1.37065 -0.82480
C -5.17477 -0.01461 -1.29772
C -3.93210 -0.66931 -0.60653
C -3.22045 -1.70665 -1.49317
C -4.01501 -3.02451 -1.59654
C -4.33189 -3.60893 -0.20666
C -5.07439 -2.58233 0.66772
C -4.27631 -1.26897 0.78113
Pd -0.29417 -0.43290 0.29997
C   2.71899 -1.08697 0.93864
C   2.89358 -0.02288 1.99343
C   3.49404 -1.38917 2.22953
C   1.42616 -1.82575 0.68897
C   1.57860 -2.64162 -0.48339
C   0.72318 -3.71109 -0.73594
C -0.32942 -3.99823 0.17177
C -0.49871 -3.18666 1.28674
C   0.34084 -2.07379 1.59699
C   2.87701 -2.31236 -1.18937
C   3.42638 -1.04699 -0.44984
C   4.95911 -1.12385 -0.31960
O   5.47246 0.09564 0.01821
C   6.91124 0.11731 0.19146
F -1.51274 -3.46966 2.15978
C   3.07490 0.24517 -1.22149
O   3.54691 0.15841 -2.50063
C   3.29894 1.32552 -3.31861
O   2.48327 1.22426 -0.79211
O   5.64308 -2.12624 -0.45576
H -2.22455 -1.88999 -1.03981

H -3.03760 -1.27130 -2.49290
H -4.96321 -2.85411 -2.14691
H -3.43800 -3.74886 -2.19875
H -3.38852 -3.88496 0.30017
H -4.92791 -4.53429 -0.30641
H -5.25271 -2.98980 1.67879
H -6.07681 -2.38503 0.23621
H -4.82911 -0.51742 1.37442
H -3.31839 -1.45883 1.29941
H -5.04701 -0.00397 -2.39760
H -6.12948 -0.49439 -1.03890
H -2.28570 4.47442 1.79780
H -1.70504 5.43738 0.38622
H -0.75000 3.89593 -1.64411
H 0.42293 2.67901 -1.06735
H 1.57828 4.85719 -1.40927
H 0.44038 5.68807 -0.34226
H 2.40225 3.45844 0.47905
H 2.53025 5.17993 0.91879
H 0.42171 5.03042 2.24826
H 1.56185 3.79916 2.80764
H 0.40498 2.02603 1.45778
H -0.75380 2.82121 2.54634
H 0.32001 -1.67627 2.61834
H 0.87523 -4.35395 -1.60960
H -1.00603 -4.84184 0.01206
H 3.61523 -3.12498 -1.06010
H 2.77286 -2.13628 -2.27131
H 2.21626 1.48662 -3.44267
H 3.74453 2.22216 -2.85882
H 3.77080 1.10929 -4.28668
H 7.21453 -0.56719 0.99941
H 7.41472 -0.18691 -0.73965
H 7.15839 1.15599 0.44819
H 3.00596 -2.06774 2.93626
H 4.58260 -1.49840 2.18286
H 1.99614 0.26884 2.54907
H 3.57344 0.80008 1.75863

TS(IV$^{C(CH2)2}$-VI$^{C(CH2)2}$).OPiv
97
BP86
SCF = -2378.01501986
H(0 K)= -2377.235901
H(413 K)= -2377.143021
G(413 K)= -2377.380685
B97D (1,2-C6Cl2H4) = -2377.64119955
Low Freq. = -277.5096cm-1, 16.1993cm-1

97
TS(IV$^{C(CH2)2}$-VI$^{C(CH2)2}$).OPiv
C -3.02081 1.34900 1.63888
C -3.58936 1.14956 0.22573
C -3.53426 2.46204 -0.59881
C -4.20092 3.64420 0.13404
C -3.62033 3.83629 1.54783
C -3.69213 2.53174 2.36588
C -5.02538 0.53280 0.22275
O -5.14292 -0.25260 -1.00814
C -3.85758 -0.65768 -1.24889
N -2.90420 0.06005 -0.51199
C -1.59269 -0.30943 -0.76641
N -1.80237 -1.29907 -1.71454
C -3.15240 -1.54004 -2.00561
O -3.34008 -2.56891 -2.88894
C -2.00555 -2.79253 -3.44660
C -0.97699 -2.35081 -2.35511
C -0.68241 -3.48659 -1.34237
C 0.21695 -4.58431 -1.94607
C 1.51345 -3.99574 -2.53192
C 1.20618 -2.89102 -3.56091
C 0.32175 -1.78600 -2.94920
Pd 0.13098 0.27947 0.16326
O 0.18546 -1.80181 1.41357
C -0.44227 -1.73296 2.53800
C -0.67993 -3.08711 3.31926
C -0.03729 -2.94222 4.71761
C 0.81061 2.12298 -0.40286
C -0.02562 3.24295 -0.08121
C -0.02902 4.37530 -0.89293
C 0.79771 4.53131 -2.01283
C 1.70734 3.48327 -2.27343
C 1.72077 2.31781 -1.50244
C 2.77523 1.25444 -1.59148
C 3.14459 0.83583 -0.09427
C 3.25165 -0.70717 -0.01943
O 3.63195 -1.12008 1.21694
C 3.49209 -2.54367 1.44185
F -0.91044 5.38995 -0.58008
C 1.97327 1.34470 0.81194
C 4.52834 1.45219 0.15898
O 5.52238 0.56551 -0.19929
C 6.85590 1.09840 -0.09325
O 4.78940 2.59069 0.52429
O -0.89773 -0.69408 3.08970
C -2.20804 -3.25807 3.48445
C -0.09570 -4.31113 2.59370
O 3.03009 -1.48656 -0.94112
H -1.93063 1.51253 1.53913
H -3.10332 0.41480 2.21964
H -4.75702 2.29319 2.57285
H -3.21253 2.66663 3.35199
H -2.56858 4.16346 1.45266
H -4.15595 4.64942 2.07374
H -4.06422 4.56354 -0.46325
H -5.29643 3.48009 0.20633
H -4.01127 2.29196 -1.58211
H -2.47053 2.69337 -0.78541
H -5.15584 -0.14300 1.09093
H -5.83059 1.28185 0.20510
H -1.89305 -2.17289 -4.35909
H -1.93935 -3.85853 -3.70973
H -1.64406 -3.91559 -1.00195
H -0.20226 -3.03037 -0.45517
H 0.44905 -5.33213 -1.16548
H -0.32927 -5.13435 -2.74189
H 2.11592 -3.55041 -1.72048
H 2.12248 -4.79440 -2.99729
H 0.70635 -3.33441 -4.44864
H 2.14604 -2.43900 -3.92482

H 0.86716 -1.28567 -2.12640
H 0.07566 -1.00795 -3.69562
H -0.69637 3.21283 0.78135
H 2.43432 3.59464 -3.08950
H 0.76186 5.44465 -2.61170
H 2.42123 0.32897 -2.07734
H 3.66777 1.59165 -2.14954
C 1.86676 0.98621 2.33400
C 2.19556 2.37901 1.92144
H 6.98258 1.97853 -0.74606
H 7.07763 1.40151 0.94370
H 7.52477 0.28439 -0.40826
H 2.41816 -2.79569 1.41456
H 4.04509 -3.11648 0.67981
H 3.91181 -2.72122 2.44266
H -0.31480 -5.23997 3.15673
H -0.51875 -4.41219 1.58096
H 0.99927 -4.23046 2.48746
H -2.44716 -4.14077 4.10909
H -2.63099 -2.35728 3.95735
H -2.69984 -3.39370 2.50403
H -0.26371 -3.81826 5.35627
H 1.06194 -2.85393 4.64186
H -0.41795 -2.03077 5.20563
H 3.21760 2.73987 2.05289
H 1.42858 3.16168 1.98533
H 0.87347 0.72739 2.72000
H 2.67543 0.36175 2.72600

VI$^{C(CH2)2}$.OPiv
97
BP86
SCF = -2378.04656766
H(0 K)= -2377.266616
H(413 K)= -2377.172591
G(413 K)= -2377.420850
B97D (1,2-C6Cl2H4) = -2377.67841322
Low Freq. = 9.8894cm-1, 12.8172cm-1

97
VI$^{C(CH2)2}$.OPiv
C -3.98878 -0.51674 2.01103
C -4.37994 -0.82129 0.55240
C -4.69649 0.49032 -0.20874
C -5.75650 1.34646 0.51205
C -5.35820 1.63630 1.97176
C -5.05358 0.33199 2.73287
C -5.52649 -1.88666 0.43989
O -5.24214 -2.72080 -0.72714
C -3.88066 -2.63939 -0.86887
N -3.32259 -1.57720 -0.14585
C -1.95720 -1.43069 -0.31915
N -1.71057 -2.47130 -1.19945
C -2.83982 -3.22945 -1.52289
O -2.55391 -4.30687 -2.31398
C -1.17363 -4.07142 -2.75938
C -0.48783 -3.13783 -1.70643
C 0.19842 -3.92191 -0.55945
C 1.51377 -4.58530 -1.01160
C 2.48015 -3.55029 -1.61589
C 1.81364 -2.78765 -2.77668
C 0.49147 -2.13091 -2.33511
Pd -0.64238 -0.20003 0.43234
O 3.34310 -1.38426 0.81326
C 3.45463 -1.65410 2.06645
C 4.64089 -2.62088 2.49784
C 5.45660 -1.90674 3.59762
C -0.20058 3.07462 0.28603
C -1.09906 3.43685 1.30036
C -2.27589 4.08841 0.91783
C -2.58543 4.39355 -0.41303
C -1.66880 4.02658 -1.41798
C -0.48416 3.37152 -1.06699
C 0.61896 2.83655 -1.95287
C 1.81953 2.67137 -0.96711
C 2.77769 1.55514 -1.42685
O 3.92261 1.61333 -0.70854
C 4.88084 0.53870 -0.94802
F -3.17068 4.44528 1.89281
C 1.12951 2.39660 0.40225
C 2.60219 4.00123 -0.89961
O 3.18920 4.25749 -2.11672
C 3.95670 5.47678 -2.15485
O 2.69621 4.76331 0.04896
O 2.71974 -1.24721 3.00445
C 3.99698 -3.89119 3.09812
C 5.55082 -2.99635 1.31633
O 2.54155 0.74787 -2.31524
H -3.01268 0.01158 1.97471
H -3.80575 -1.46707 2.54536
H -5.99098 -0.25284 2.84581
H -4.70848 0.55682 3.75851
H -4.46684 2.28934 1.98418
H -6.16403 2.19689 2.48206
H -5.90470 2.29110 -0.04233
H -6.73686 0.82586 0.49742
H -5.01821 0.24513 -1.23816
H -3.74506 1.04952 -0.28768
H -5.53970 -2.53031 1.34201
H -6.52371 -1.44685 0.28665
H -1.20705 -3.57934 -3.75122
H -0.69295 -5.05679 -2.85034
H -0.51029 -4.66928 -0.15418
H 0.40770 -3.19082 0.24427
H 1.97978 -5.08878 -0.14510
H 1.30089 -5.38422 -1.75489
H 2.78583 -2.83300 -0.82680
H 3.39874 -4.05389 -1.97341
H 1.62877 -3.48035 -3.62695
H 2.48509 -1.99568 -3.15046
H 0.69481 -1.35741 -1.56577
H -0.00379 -1.61517 -3.17930
H -0.91058 3.22208 2.35606
H -1.89071 4.25679 -2.46649
H -3.51792 4.91594 -0.64387
H 0.36637 1.83085 -2.33922
H 0.87163 3.47683 -2.81337
C 1.24947 1.03135 1.14722
C 1.86732 2.29716 1.70639
H 3.31951 6.34988 -1.93607

H   4.77369 5.44723 -1.41504
H   4.36180 5.53927 -3.17505
H   4.54230 -0.35257 -0.38163
H   4.94572 0.33276 -2.02781
H   5.83714 0.92300 -0.56349
H   6.34947 -3.69669 1.63498
H   4.97158 -3.47041 0.50714
H   6.03472 -2.10139 0.88791
H   4.75995 -4.57602 3.51977
H   3.29201 -3.60075 3.89412
H   3.43049 -4.44820 2.32909
H   6.22298 -2.57547 4.03835
H   5.97685 -1.01874 3.19273
H   4.77061 -1.56469 4.38919
H   2.95856 2.34592 1.66740
H   1.41811 2.78369 2.57944
H   0.37178 0.68807 1.77990
H   1.96133 0.24265 0.83588

R = Ph (8f)

III^{Ph}
104
BP86
SCF =   -2532.28347255
H(0 K)=   -2531.441946
H(413 K)=   -2531.342322
G(413 K)=   -2531.599816
B97D (1,2-C6Cl2H4) =   -2531.79919880
Low   Freq.   =   8.0188cm-1, 10.5873cm-1

104
III^{Ph}
C -1.08182 -2.56001 -2.42130
C -2.45567 -2.60013 -2.09926
C -3.03026 -3.84582 -1.76781
C -2.25649 -5.01704 -1.74919
C -0.88951 -4.96246 -2.06705
C -0.30560 -3.72982 -2.40453
C -3.30713 -1.34342 -2.13296
C -3.27058 -0.48943 -0.82017
C -4.37209 0.59550 -0.90794
O -5.13540 0.77693 -1.84593
C -1.86742 0.10925 -0.50535
C -1.18529 0.99188 -1.54659
C   0.23342 1.14227 -1.52746
C   0.85251 1.98612 -2.47818
C   0.08319 2.68025 -3.41789
C -1.30621 2.56101 -3.45532
C -1.91788 1.71089 -2.52058
Pd 1.61166 0.23719 -0.38743
O   3.50535 -0.82816 0.15200
C   3.77183 -0.90367 -1.10517
C   5.10347 -1.51133 -1.57356
F   0.70972 3.49510 -4.31232
C   0.75509 0.72725 1.32143
N   0.46689 1.94273 1.89988
C -0.05541 1.81782 3.19391
C -0.10000 0.47957 3.45818
N   0.37828 -0.15602 2.30855
C   0.57841 3.38839 1.55018
C   0.47320 3.96689 3.00374
O -0.32205 3.01784 3.78197
C -0.61299 3.84270 0.67104
C -0.49314 5.32297 0.25297
C   0.85767 5.61095 -0.42782
C   2.03048 5.19474 0.47953
C   1.92886 3.70971 0.88306
O -0.40630 -0.38102 4.46331
C -0.40103 -1.70042 3.81046
C   0.61154 -1.59848 2.62681
C   0.27366 -2.53872 1.45876
C   0.61343 -4.00734 1.78604
C   2.08308 -4.17028 2.21377
C   2.40934 -3.25559 3.40840
C   2.08008 -1.78205 3.09391
O   2.92617 -0.43526 -1.95909
C -3.68033 -1.37947 0.38061
O -2.99447 -1.65027 1.35630
O -4.95184 -1.84203 0.21588
C -5.43198 -2.68716 1.28995
O -4.40008 1.33585 0.23752
C -5.42105 2.36313 0.26362
H   2.02086 3.06944 -0.01380
H   2.75056 3.42187 1.56415
H   2.04199 5.83396 1.38591
H   2.99568 5.36678 -0.02887
H   0.91379 5.04539 -1.37739
H   0.93556 6.68181 -0.68825
H -1.32963 5.57448 -0.42267
H -0.60491 5.98302 1.13709
H -1.55518 3.66225 1.22029
H -0.63338 3.20757 -0.23028
H   1.47681 4.04944 3.46481
H -0.04136 4.93668 3.05299
H -1.41841 -1.90665 3.43253
H -0.11253 -2.43280 4.57694
H   2.26202 -1.14289 3.97775
H   2.73693 -1.42544 2.28091
H   3.47639 -3.33603 3.68289
H   1.83955 -3.59587 4.29685
H   2.74025 -3.90192 1.36439
H   2.29767 -5.22475 2.46601
H -0.04928 -4.37650 2.59522
H   0.38999 -4.62649 0.89972
H   0.86660 -2.20896 0.58273
H -0.79064 -2.42951 1.19528
H   1.94138 2.09087 -2.51594
H -3.00500 1.60734 -2.57686
H -1.88979 3.10561 -4.20223
H -1.19874 -0.73815 -0.28839
H -1.95410 0.66161 0.44727
H -2.97656 -0.68897 -2.95584
H -4.35865 -1.60027 -2.33426
H -5.24608 3.10057 -0.53581
H -6.41934 1.91894 0.12483
H -5.33604 2.83403 1.25209
H -4.81197 -3.59401 1.37051
H -5.40227 -2.14643 2.24907
H -6.46452 -2.94436 1.01855

```
H  -4.10096 -3.89496 -1.53856
H  -2.72493 -5.97530 -1.49902
H  -0.28660 -5.87694 -2.06631
H   0.75594 -3.67479 -2.66736
H  -0.61674 -1.60605 -2.69364
C   5.11605 -1.68456 -3.10491
C   6.23094 -0.53840 -1.13702
C   5.30311 -2.87575 -0.87177
H   6.11665  0.44588 -1.62314
H   6.21945 -0.38984 -0.04514
H   7.21466 -0.94885 -1.42575
H   4.31828 -2.36883 -3.43772
H   4.96285 -0.72183 -3.61737
H   6.08629 -2.10262 -3.42653
H   5.26359 -2.76232  0.22300
H   4.52387 -3.59725 -1.17345
H   6.28386 -3.30227 -1.14657
```

TS(III^{Ph}-IV^{Ph})1

```
104
BP86
SCF  =          -2532.26405354
H(0 K)=         -2531.423898
H(413 K)=       -2531.325191
G(413 K)=       -2531.579101
B97D  (1,2-C6Cl2H4)  =        -
2531.78431616
Low   Freq.   =   -35.4417cm-1,
9.5932cm-1

104
```

TS(III^{Ph}-IV^{Ph})1

```
C  -4.11852  1.61135 -1.70068
C  -3.45984  1.29885 -0.49380
C  -3.70953  2.09575  0.64461
C  -4.60564  3.17481  0.57954
C  -5.25988  3.47666 -0.62632
C  -5.01209  2.69204 -1.76471
C  -2.48095  0.14047 -0.40644
C  -3.03553 -1.27113 -0.01425
C  -4.07266 -1.14630  1.12147
O  -3.83371 -1.27790  2.31197
C  -1.85498 -2.16630  0.50937
C  -0.82614 -2.51566 -0.55316
C   0.25195 -1.64735 -0.90246
C   1.11507 -2.04736 -1.94951
C   0.91641 -3.25976 -2.61850
C  -0.11483 -4.13543 -2.27407
C  -0.97616 -3.74192 -1.24083
Pd  0.44085  0.10213  0.08770
O   0.58984  1.99988  1.08832
C  -0.17709  1.88424  2.13721
O  -0.86425  0.85009  2.36013
F   1.77277 -3.60197 -3.62244
C   2.36997  0.09740 -0.19553
N   3.09151  0.80617 -1.12027
C   4.47415  0.64256 -0.96486
C   4.63528 -0.22885  0.07465
N   3.34478 -0.53545  0.52529
C   2.82995  1.90842 -2.08900
C   4.21906  1.87576 -2.80894
O   5.19939  1.41238 -1.82041
C   1.65183  1.58617 -3.02562
C   1.31415  2.76506 -3.96031
C   1.04538  4.05445 -3.16312
C   2.24471  4.39998 -2.26196
C   2.59888  3.23348 -1.31765
O   5.60812 -0.87878  0.76833
C   4.93174 -1.34894  1.98105
C   3.41699 -1.53985  1.62279
C   3.11802 -2.95769  1.07577
C   3.11700 -4.02119  2.19240
C   2.15684 -3.64115  3.33450
C   2.49494 -2.24802  3.89651
C   2.47770 -1.17615  2.78844
C  -0.19134  3.09343  3.10721
C  -3.68203 -1.93423 -1.24529
O  -3.67490 -1.49850 -2.38780
O  -4.24221 -3.13157 -0.90577
C  -4.86377 -3.83330 -2.00852
O  -5.30134 -0.83838  0.62253
C  -6.31930 -0.60720  1.62465
H   1.45252 -1.07367  2.38209
H   2.75550 -0.18367  3.18676
H   3.49211 -2.27786  4.38143
H   1.77679 -1.96166  4.68441
H   1.11792 -3.63480  2.95184
H   2.19556 -4.39914  4.13718
H   2.84155 -4.99849  1.75833
H   4.13988 -4.14666  2.60182
H   3.85551 -3.20813  0.29132
H   2.12752 -2.92718  0.58849
H   5.05318 -0.58147  2.76916
H   5.42992 -2.27998  2.28536
H   4.20795  1.15997 -3.65321
H   4.55626  2.86199 -3.15695
H   3.50568  3.46716 -0.72951
H   1.78064  3.06560 -0.59406
H   2.03272  5.30003 -1.65822
H   3.11612  4.65764 -2.89784
H   0.14483  3.91260 -2.53501
H   0.82630  4.89123 -3.85029
H   2.14887  2.93660 -4.67005
H   0.43766  2.49654 -4.57574
H   0.77715  1.35123 -2.38714
H   1.87494  0.67148 -3.60395
H   1.95861 -1.42722 -2.26281
H  -1.79733 -4.40680 -0.94826
H  -0.23156 -5.08777 -2.79805
H  -2.29123 -3.09020  0.92279
H  -1.40303 -1.61806  1.35507
H  -1.96426  0.01364 -1.37233
H  -1.74798  0.40022  0.39848
H  -6.07832  0.29629  2.20706
H  -7.25335 -0.46533  1.06472
H  -6.39435 -1.46795  2.30757
H  -5.66440 -3.22075 -2.45252
H  -4.11929 -4.06500 -2.78683
H  -5.27475 -4.75494 -1.57437
H  -3.18439  1.86413  1.57876
H  -4.78655  3.78575  1.47091
H  -5.95480  4.32175 -0.67993
```

```
H  -5.51650  2.92258 -2.70966
H  -3.93822  0.98505 -2.57979
C  -1.18051  2.84216  4.26104
C   1.24039  3.27927  3.66899
C  -0.60050  4.36063  2.31740
H  -1.16726  3.69574  4.96246
H  -2.21019  2.71848  3.88787
H  -0.92212  1.92657  4.81635
H   1.27650  4.15540  4.34115
H   1.55697  2.39575  4.25128
H   1.96365  3.43360  2.85227
H  -0.61293  5.23935  2.98716
H   0.10737  4.55604  1.49635
H  -1.60777  4.25047  1.87968
```

Int(III^{Ph}-IV^{Ph})
104
BP86
SCF = -2532.26592958
H(0 K)= -2531.425756
H(413 K)= -2531.326067
G(413 K)= -2531.581842
B97D (1,2-C6Cl2H4) = -2531.78764357
Low Freq. = 13.7884cm-1, 16.4377cm-1

104
Int(III^{Ph}-IV^{Ph})

```
C  -3.62345  1.91229 -1.56108
C  -3.15622  1.47872 -0.30330
C  -3.50211  2.22156  0.84637
C  -4.31285  3.36357  0.74062
C  -4.77893  3.78497 -0.51553
C  -4.42828  3.05748 -1.66462
C  -2.29488  0.23290 -0.15602
C  -3.01019 -1.15253 -0.02253
C  -4.13433 -1.02398  1.02481
O  -3.99206 -1.21786  2.22324
C  -1.98407 -2.22170  0.49556
C  -0.94861 -2.63801 -0.53538
C   0.12982 -1.79164 -0.92968
C   1.02207 -2.26600 -1.91707
C   0.84192 -3.52653 -2.49722
C  -0.20196 -4.37350 -2.12021
C  -1.08882 -3.90839 -1.13883
Pd  0.28346  0.05428 -0.11517
O   0.42741  2.04905  0.64067
C  -0.08899  2.18713  1.83664
O  -0.68239  1.27438  2.45994
F   1.72508 -3.94287 -3.44799
C   2.23464 -0.10921 -0.05852
N   3.14102  0.43851 -0.93031
C   4.46650  0.16873 -0.56683
C   4.39910 -0.60569  0.55611
N   3.03644 -0.75092  0.84415
C   3.12987  1.47402 -2.00478
C   4.58906  1.24323 -2.51910
O   5.37575  0.78567 -1.36910
C   2.05900  1.19551 -3.07459
C   1.98077  2.32158 -4.12545
C   1.75522  3.69402 -3.46558
C   2.85628  3.98964 -2.43112
C   2.94700  2.87617 -1.36864
O   5.20274 -1.26756  1.43044
C   4.33061 -1.55767  2.57247
C   2.86058 -1.63628  2.02990
C   2.47828 -3.06837  1.58128
C   2.22095 -4.00094  2.78230
C   1.17480 -3.40839  3.74420
C   1.59326 -2.00346  4.21514
C   1.83412 -1.05780  3.02187
C   0.10739  3.59634  2.47074
C  -3.55642 -1.61812 -1.38350
O  -3.34081 -1.08602 -2.46384
O  -4.26739 -2.77149 -1.24447
C  -4.76474 -3.32145 -2.48813
O  -5.29745 -0.61919  0.45064
C  -6.37533 -0.36435  1.38353
H   0.88356 -0.88003  2.48403
H   2.17569 -0.06488  3.36489
H   2.51236 -2.08115  4.83154
H   0.81881 -1.56451  4.86748
H   0.19837 -3.34080  3.22650
H   1.02826 -4.07706  4.61132
H   1.89551 -4.98779  2.40841
H   3.16494 -4.18067  3.33575
H   3.27577 -3.47126  0.93026
H   1.56507 -2.99624  0.96452
H   4.43234 -0.73611  3.30679
H   4.68386 -2.49981  3.01443
H   4.61262  0.45514 -3.29622
H   5.07096  2.15632 -2.89518
H   3.78786  3.06485 -0.67553
H   2.02365  2.85114 -0.76064
H   2.66852  4.95376 -1.92641
H   3.82746  4.10349 -2.95463
H   0.76975  3.69788 -2.96121
H   1.72436  4.48759 -4.23357
H   2.91577  2.34766 -4.72122
H   1.16889  2.09210 -4.83778
H   1.08566  1.09965 -2.55338
H   2.25772  0.22131 -3.55662
H   1.87297 -1.66723 -2.25113
H  -1.91877 -4.55143 -0.82267
H  -0.30783 -5.36012 -2.57901
H  -2.55487 -3.10593  0.82164
H  -1.51211 -1.79339  1.39723
H  -1.64530  0.14963 -1.06730
H  -1.70525  0.35857  0.78866
H  -6.12421  0.49604  2.02385
H  -7.25170 -0.13863  0.76155
H  -6.55652 -1.24674  2.01688
H  -5.41079 -2.59216 -3.00161
H  -3.92614 -3.58744 -3.15108
H  -5.33616 -4.21559 -2.20414
H  -3.10859  1.90975  1.82026
H  -4.57118  3.93081  1.64161
H  -5.40617  4.67910 -0.59921
H  -4.78348  3.38213 -2.64885
H  -3.36845  1.33064 -2.45174
C  -0.71785  3.70998  3.76676
```

C	1.61385	3.76256	2.79348
C	-0.32751	4.68811	1.46602
H	-0.54674	4.69329	4.24100
H	-1.79654	3.61056	3.56146
H	-0.44536	2.91871	4.48247
H	1.80386	4.75737	3.23557
H	1.95192	3.00176	3.51942
H	2.22407	3.66504	1.88076
H	-0.18142	5.69158	1.90582
H	0.25967	4.62366	0.53656
H	-1.39336	4.58214	1.20073

TS(III^{Ph}-IV^{Ph})2
104
BP86
SCF = -2532.23853824
H(0 K)= -2531.402543
H(413 K)= -2531.303934
G(413 K)= -2531.555644
B97D (1,2-C6Cl2H4) = -2531.75541537
Low Freq. = -908.2225cm-1, 13.4694cm-1

104
TS(III^{Ph}-IV^{Ph})2

C	-2.79003	2.12404	-1.72226
C	-2.85496	1.40892	-0.50178
C	-3.63314	1.97681	0.53670
C	-4.31384	3.19188	0.35816
C	-4.22709	3.88723	-0.85878
C	-3.45706	3.34477	-1.89995
C	-2.07305	0.11446	-0.36610
C	-2.85875	-1.22355	-0.14047
C	-3.91419	-1.11015	0.97383
O	-3.71007	-1.32015	2.16203
C	-1.86065	-2.35844	0.27847
C	-0.80988	-2.63427	-0.77842
C	0.19180	-1.67520	-1.10316
C	1.10488	-1.97852	-2.13627
C	1.02022	-3.19755	-2.82030
C	0.05865	-4.15991	-2.50649
C	-0.85267	-3.85717	-1.48326
Pd	0.23127	0.07022	-0.09854
O	0.25005	1.92365	1.09674
C	-0.56277	1.83349	2.07038
O	-1.42297	0.86573	2.16968
F	1.91942	-3.45506	-3.81279
C	2.23860	0.05033	-0.03653
N	3.10855	0.81742	-0.77374
C	4.45035	0.61581	-0.43036
C	4.44546	-0.34052	0.54412
N	3.09917	-0.65594	0.76493
C	3.00634	1.98141	-1.69707
C	4.48869	1.97524	-2.19886
O	5.30379	1.43280	-1.10716
C	1.98336	1.73781	-2.82099
C	1.79844	2.97676	-3.72006
C	1.42022	4.21965	-2.89373
C	2.47121	4.48979	-1.80176
C	2.66822	3.26174	-0.89034
O	5.29816	-1.06237	1.32216
C	4.43736	-1.62134	2.36801
C	3.00499	-1.76781	1.75045
C	2.82436	-3.12231	1.02000
C	2.65756	-4.29551	2.00652
C	1.51267	-4.03622	3.00281
C	1.72659	-2.70883	3.75362
C	1.88043	-1.52789	2.77432
C	-0.53732	2.90827	3.17324
C	-3.54108	-1.65604	-1.46392
O	-3.45819	-1.08166	-2.53869
O	-4.21905	-2.83190	-1.29730
C	-4.86158	-3.32219	-2.49652
O	-5.12439	-0.72946	0.47613
C	-6.16321	-0.56878	1.46790
H	0.93947	-1.38357	2.20788
H	2.07165	-0.58252	3.31419
H	2.62776	-2.78893	4.39521
H	0.88072	-2.50608	4.43367
H	0.55372	-3.99056	2.45142
H	1.42842	-4.87287	3.71929
H	2.47569	-5.22292	1.43547
H	3.59936	-4.46197	2.56773
H	3.68644	-3.28962	0.34870
H	1.92772	-3.04338	0.38072
H	4.42465	-0.91977	3.22444
H	4.88351	-2.57771	2.67448
H	4.60134	1.31677	-3.08162
H	4.88054	2.97663	-2.42561
H	3.47359	3.44350	-0.15467
H	1.74513	3.06431	-0.31712
H	2.17691	5.35576	-1.18263
H	3.43144	4.76988	-2.28075
H	0.43335	4.05488	-2.41987
H	1.31515	5.10023	-3.55260
H	2.73241	3.17795	-4.28319
H	1.02409	2.76056	-4.47690
H	1.01973	1.47470	-2.33973
H	2.28996	0.85933	-3.41726
H	1.89569	-1.28071	-2.42320
H	-1.62426	-4.59094	-1.22151
H	0.02750	-5.10751	-3.05079
H	-2.43857	-3.27449	0.48347
H	-1.40449	-2.03729	1.23033
H	-1.64934	-0.03669	-1.37741
H	-1.50153	0.38784	1.06906
H	-5.91814	0.25966	2.15231
H	-7.07573	-0.34016	0.90106
H	-6.28488	-1.49234	2.05576
H	-5.59583	-2.59002	-2.86906
H	-4.11603	-3.51002	-3.28565
H	-5.36043	-4.25596	-2.20186
H	-3.69029	1.47335	1.50636
H	-4.91340	3.59963	1.18026
H	-4.75576	4.83684	-0.99443
H	-3.38738	3.86551	-2.86164
H	-2.22025	1.69331	-2.55310
C	-1.58753	2.61473	4.26220
C	0.88312	2.93136	3.79085
C	-0.83273	4.27381	2.50063
H	-1.54168	3.39631	5.04075

```
H  -2.60594  2.60333  3.84289
H  -1.41263  1.63742  4.74010
H   0.94951  3.73288  4.54697
H   1.11706  1.97521  4.29114
H   1.64506  3.11448  3.01679
H  -0.81561  5.07397  3.26124
H  -0.07827  4.50359  1.73176
H  -1.82498  4.27348  2.01891
```

IV^{Ph}

```
104
BP86
SCF  =           -2532.26179070
H(0 K)=          -2531.421151
H(413 K)=        -2531.321920
G(413 K)=        -2531.575706
B97D  (1,2-C6Cl2H4)  =  -2531.78187051
Low  Freq.  =  15.5918cm-1, 18.1488cm-1

104
```

IV^{Ph}

```
C  -3.26194  2.51353  0.33635
C  -2.60918  1.61276 -0.54157
C  -2.35932  2.06988 -1.86138
C  -2.76309  3.34572 -2.28020
C  -3.41046  4.22103 -1.39018
C  -3.65140  3.79835 -0.07391
C  -2.18184  0.25712 -0.02727
C  -2.96656 -0.96234 -0.58327
C  -2.56575 -2.23613  0.17587
O  -1.84904 -2.32608  1.17969
C  -2.79471 -1.14811 -2.10198
C  -1.37385 -1.28606 -2.62614
C  -0.20249 -0.75250 -2.00431
C   1.00408 -0.85442 -2.75057
C   1.05290 -1.46341 -4.00690
C  -0.07818 -2.02401 -4.60083
C  -1.27836 -1.92017 -3.88866
Pd -0.09415  0.07452 -0.14970
O  -0.02178  0.95982  1.92005
C  -0.40762  0.44359  2.98829
O  -1.00619 -0.73873  3.08333
F   2.25142 -1.52442 -4.66079
C   1.99797  0.12608 -0.04403
N   2.91979 -0.78502  0.42336
C   4.23193 -0.29586  0.45803
C   4.15776  0.99411  0.01879
N   2.80967  1.21132 -0.28698
C   2.93305 -2.23881  0.73298
C   4.32228 -2.28865  1.45250
O   5.14171 -1.22533  0.86863
C   2.92836 -3.06348 -0.57952
C   2.88131 -4.58149 -0.31541
C   1.69515 -4.95965  0.59026
C   1.73042 -4.16305  1.90780
C   1.76194 -2.64458  1.64318
O   4.93713  2.09437 -0.17590
C   4.10269  2.97923 -0.99540
C   2.61021  2.63493 -0.66912
C   1.67876  2.77770 -1.88642
C   1.40394  4.25366 -2.23554
C   0.83536  5.01878 -1.02637
C   1.77864  4.90833  0.18546
C   2.07415  3.43787  0.54299
C  -0.22439  1.13826  4.34853
C   0.48865  2.48994  4.16093
C  -1.62557  1.35538  4.97731
C   0.61718  0.21158  5.26413
C  -4.47796 -0.73692 -0.31201
O  -5.30746 -0.37604 -1.13138
O  -4.76575 -0.92987  1.01373
C  -6.14115 -0.65474  1.37051
O  -3.13338 -3.33388 -0.37288
C  -2.86951 -4.58478  0.30672
H   0.82729 -2.33521  1.13645
H   1.81627 -2.07814  2.59047
H   2.61976 -4.46621  2.49705
H   0.85105 -4.40254  2.53115
H   0.75010 -4.73402  0.05798
H   1.69670 -6.04556  0.79506
H   2.82173 -5.11565 -1.28023
H   3.82455 -4.91695  0.16126
H   3.81664 -2.79589 -1.18037
H   2.04126 -2.75715 -1.16278
H   4.20683 -2.09131  2.53633
H   4.86441 -3.23287  1.30166
H   4.32454  2.78161 -2.06182
H   4.38256  4.01126 -0.74085
H   2.80609  3.37314  1.36968
H   1.15117  2.93959  0.88867
H   1.34465  5.41556  1.06572
H   2.72475  5.44251 -0.03762
H  -0.15400  4.59632 -0.76677
H   0.67048  6.08000 -1.28686
H   2.33717  4.74442 -2.58062
H   0.69977  4.29721 -3.08476
H   0.72735  2.26722 -1.63717
H   2.11109  2.23620 -2.74714
H   1.93927 -0.45199 -2.35675
H  -2.19186 -2.33236 -4.33589
H  -0.01732 -2.51308 -5.57672
H  -3.28091 -0.27112 -2.56699
H  -3.39453 -2.01487 -2.42515
H  -2.37267  0.26282  1.06145
H  -1.79254 -4.81380  0.28729
H  -3.20923 -4.53304  1.35317
H  -3.43569 -5.34177 -0.25196
H  -6.38820  0.39887  1.16501
H  -6.82756 -1.29781  0.79650
H  -6.21379 -0.86918  2.44551
H   0.61549  2.98013  5.14138
H   1.48407  2.35896  3.70716
H  -0.09219  3.16215  3.50937
H   0.74534  0.68646  6.25213
H   0.12475 -0.76303  5.40609
H   1.62168  0.03767  4.84067
H  -1.51701  1.84455  5.96071
H  -2.25057  2.00646  4.34224
H  -2.15180  0.39842  5.11781
H  -1.80980  1.42375 -2.55317
```

H -2.56097 3.66315 -3.30957
H -3.71805 5.21950 -1.71869
H -4.15168 4.46710 0.63599
H -3.46848 2.18954 1.36396
H -1.20854 -1.16924 2.18266

V^{Ph}.OPiv
103
BP86
SCF = -2531.72701190
H(0 K)= -2530.900505
H(413 K)= -2530.801190
G(413 K)= -2531.056675
B97D (1,2-C6Cl2H4) = -2531.29870975
Low Freq. = 12.2539cm-1, 16.3617cm-1

103
V^{Ph}.OPiv
C 1.77139 -2.71226 1.64512
C 2.68396 -2.66567 0.40627
C 2.16665 -3.61390 -0.70577
C 1.93940 -5.04914 -0.19013
C 1.01924 -5.06869 1.04498
C 1.56742 -4.15132 2.15488
C 4.19365 -2.91383 0.74348
O 4.98195 -2.08737 -0.17716
C 4.15558 -1.03063 -0.44613
N 2.81954 -1.29090 -0.12194
C 1.97289 -0.23916 -0.35511
N 2.84381 0.67861 -0.90251
C 4.17483 0.24367 -0.92941
O 5.04200 1.20363 -1.37657
C 4.17222 2.21906 -1.97184
C 2.77619 2.11245 -1.26867
C 2.67996 2.98376 0.00814
C 2.54180 4.48405 -0.31577
C 1.34930 4.74681 -1.25358
C 1.46697 3.90945 -2.54018
C 1.60210 2.40726 -2.22173
Pd -0.02932 -0.01656 0.00772
O 0.26522 1.21696 1.78761
C 1.20653 1.08377 2.67639
C 0.95729 1.94257 3.97392
C 0.64183 3.40286 3.58381
C -2.11224 0.35942 0.34134
C -2.99457 -0.92954 0.48388
C -4.40440 -0.75675 -0.14416
O -5.08390 0.28453 0.42479
C -6.33936 0.58755 -0.21409
H -2.04669 0.84542 1.32336
C -3.27934 -1.26263 1.97343
O -3.41291 -0.16273 2.77035
C -3.73597 -0.46880 4.14120
C -2.34815 -2.21645 -0.12538
C -1.68795 -2.01379 -1.47063
C -0.58148 -1.11956 -1.58193
C 0.04422 -1.01757 -2.84779
C -0.43263 -1.74623 -3.94296
C -1.52690 -2.60870 -3.84949
C -2.14577 -2.73195 -2.59318
F 0.20137 -1.61315 -5.15477
O -3.41207 -2.40114 2.41246
O 2.22871 0.36476 2.60974
C -0.26282 1.33739 4.70808
C 2.19593 1.89348 4.88749
O -4.88269 -1.44385 -1.03825
H 0.79858 -2.27409 1.34095
H 2.16922 -2.03316 2.41774
H 2.52650 -4.56497 2.53258
H 0.87798 -4.13772 3.01829
H 0.01234 -4.71457 0.75143
H 0.89777 -6.10300 1.41837
H 1.51440 -5.66662 -1.00242
H 2.91098 -5.51671 0.07420
H 2.87923 -3.60733 -1.55158
H 1.21631 -3.19282 -1.08140
H 4.41197 -2.59359 1.78028
H 4.51522 -3.95526 0.59339
H 4.07552 2.01009 -3.05602
H 4.66417 3.19217 -1.82475
H 3.56005 2.78981 0.64726
H 1.79189 2.63705 0.56713
H 2.42735 5.05023 0.62675
H 3.47102 4.86460 -0.79031
H 0.40968 4.47061 -0.73876
H 1.27891 5.82321 -1.49953
H 2.34316 4.25583 -3.12821
H 0.57927 4.06157 -3.17901
H 0.67749 2.04101 -1.73192
H 1.72451 1.81883 -3.14948
H 0.91030 -0.36626 -2.99848
H -3.01471 -3.39175 -2.48374
H -1.87542 -3.15917 -4.72839
H -3.10708 -3.01382 -0.17278
H -1.57982 -2.53828 0.59962
H -2.94390 -1.07966 4.60375
H -3.81579 0.50389 4.64659
H -4.68845 -1.02187 4.20444
H -6.16299 0.93814 -1.24451
H -6.99368 -0.29973 -0.24442
H -6.79113 1.38767 0.38966
H -0.50570 1.92039 5.61766
H -1.14199 1.32978 4.04375
H -0.05774 0.29648 5.01714
H 2.01338 2.45709 5.82276
H 2.45187 0.85258 5.14104
H 3.07589 2.32970 4.38509
H 0.41489 4.01119 4.48071
H 1.50165 3.87099 3.07054
H -0.22156 3.43812 2.90135
C -2.49142 1.40363 -0.66021
C -2.98249 1.14597 -1.97061
C -2.33076 2.77295 -0.30457
C -3.29307 2.18994 -2.85593
C -2.62775 3.81376 -1.19436
C -3.11780 3.53384 -2.48344
H -3.10247 0.11253 -2.30761
H -1.95413 3.00343 0.69899
H -3.66870 1.94420 -3.85718
H -2.49132 4.85410 -0.87239

H -3.36235 4.34498 -3.17903

V^{Ph}

87
BP86
SCF = -2185.20454316
H(0 K)= -2184.508968
H(413 K)= -2184.426525
G(413 K)= -2184.644754
B97D (1,2-C6Cl2H4) = -2184.80386613
Low Freq. = 10.2423cm-1, 13.2506cm-1

87
V^{Ph} C -1.62369 -1.21747 -1.68994
C -0.47669 -0.41886 -1.45844
C 0.25982 0.08180 -2.54980
C -0.16747 -0.20305 -3.85357
C -1.30341 -0.97440 -4.11423
C -2.02227 -1.47623 -3.01800
Pd 0.08521 0.05282 0.39899
C -1.93678 0.42132 0.78433
C -2.22868 1.81266 0.31412
F 0.55324 0.29272 -4.90005
C -2.38774 -1.80515 -0.52767
C -2.94040 -0.73123 0.47887
C -3.22291 -1.50198 1.79400
O -2.60586 -1.41416 2.84650
C 2.15225 -0.15519 0.36491
N 2.93246 -1.28777 0.43863
C 4.30452 -1.03631 0.42989
C 4.42951 0.32528 0.37701
N 3.12612 0.82069 0.31792
C 2.68686 -2.74958 0.37581
C 4.11714 -3.23043 0.80354
O 5.06118 -2.16756 0.44245
C 2.32705 -3.16423 -1.07332
C 1.98586 -4.66352 -1.18276
C 0.87645 -5.06338 -0.19276
C 1.26024 -4.68228 1.24937
C 1.58816 -3.18067 1.36612
O 5.37701 1.30180 0.41734
C 4.65625 2.52036 0.03522
C 3.14700 2.30286 0.39962
C 2.18740 2.95038 -0.61614
C 2.14889 4.48567 -0.47801
C 1.78375 4.90996 0.95641
C 2.75204 4.28912 1.98010
C 2.81140 2.75475 1.84415
C -4.29489 -0.21086 -0.04910
O -4.75902 -0.40889 -1.16298
O -4.92208 0.53035 0.90841
C -6.16203 1.13806 0.47917
O -4.25021 -2.38205 1.60915
C -4.57793 -3.18185 2.76915
H 0.68784 -2.57367 1.13922
H 1.89611 -2.91725 2.39471
H 2.13366 -5.28647 1.56950
H 0.44283 -4.93096 1.94905
H -0.06109 -4.54296 -0.46935
H 0.67100 -6.14671 -0.26212
H 1.68395 -4.89203 -2.22003
H 2.88919 -5.27571 -0.98522
H 3.16371 -2.89889 -1.74546
H 1.45695 -2.55730 -1.38379
H 4.16748 -3.38302 1.89867
H 4.44713 -4.14238 0.28584
H 4.77530 2.66909 -1.05496
H 5.12498 3.35405 0.57715
H 3.54857 2.32007 2.54415
H 1.82537 2.32291 2.09988
H 2.45384 4.55137 3.01087
H 3.76400 4.71931 1.83770
H 0.75072 4.57731 1.17459
H 1.78980 6.01129 1.04390
H 3.13322 4.91606 -0.75427
H 1.41878 4.89358 -1.19811
H 1.17683 2.53110 -0.43760
H 2.48194 2.65199 -1.63888
H 1.15446 0.69298 -2.40842
H -2.92280 -2.07485 -3.19380
H -1.61058 -1.16879 -5.14516
H -3.22705 -2.42602 -0.87871
H -1.70298 -2.45795 0.04672
H -1.79986 0.44012 1.88664
H -3.71817 -3.80184 3.07029
H -4.86359 -2.53699 3.61543
H -5.42172 -3.81342 2.45959
H -5.96835 1.86195 -0.32850
H -6.86609 0.37243 0.11589
H -6.56082 1.64822 1.36645
C -2.64188 2.13841 -1.00525
C -2.86166 3.46905 -1.38785
C -2.66997 4.52292 -0.47652
C -2.26148 4.22340 0.83374
C -2.05163 2.89127 1.22065
H -2.77813 1.33936 -1.73932
H -3.18033 3.68492 -2.41402
H -2.84454 5.56016 -0.78158
H -2.12532 5.02818 1.56548
H -1.76183 2.66675 2.25482

TS(V^{Ph}-VI^{Ph})

87
BP86
SCF = -2185.18545718
H(0 K)= -2184.490915
H(413 K)= -2184.409206
G(413 K)= -2184.626268
B97D (1,2-C6Cl2H4) = -2184.77860537
Low Freq. = -268.5727cm-1, 6.4073cm-1

87
TS(V^{Ph}-VI^{Ph})
C -1.63910 -2.18483 0.41683
C -1.05350 -1.34681 -0.56503
C -0.87048 -1.83642 -1.88047
C -1.17450 -3.17703 -2.14398
C -1.70714 -4.03812 -1.17514

C -1.95546 -3.51814 0.10790
Pd 0.24114 0.16177 -0.08962
C -1.94940 0.41066 -0.02206
C -2.82771 0.65164 -1.22122
F -0.95068 -3.66252 -3.39824
C -1.97934 -1.50043 1.71370
C -2.55768 -0.09006 1.33308
C -2.21876 0.92136 2.45616
O -1.59720 1.96912 2.35091
C 2.28026 0.29895 -0.01462
N 3.24728 -0.63192 0.29490
C 4.55186 -0.13227 0.24531
C 4.43015 1.19096 -0.07191
N 3.06203 1.41134 -0.24229
C 3.27939 -2.10101 0.49912
C 4.75882 -2.22243 1.00567
O 5.50298 -1.08125 0.46683
C 3.04435 -2.82759 -0.84895
C 3.01392 -4.36054 -0.68800
C 1.98690 -4.79723 0.37303
C 2.24663 -4.09181 1.71741
C 2.25527 -2.55828 1.55477
O 5.17911 2.32046 -0.21734
C 4.26722 3.28291 -0.84071
C 2.80499 2.85321 -0.46933
C 1.80815 3.07635 -1.62264
C 1.45662 4.56561 -1.80729
C 0.92505 5.18465 -0.50096
C 1.93316 4.99597 0.64746
C 2.29716 3.51068 0.83908
C -4.10229 -0.18441 1.27659
O -4.75870 -1.21502 1.28615
O -4.64693 1.06110 1.21202
C -6.08633 1.08291 1.05971
O -2.71056 0.46214 3.64277
C -2.45690 1.32488 4.77684
H 1.25998 -2.20731 1.22039
H 2.46954 -2.05352 2.51485
H 3.21583 -4.43387 2.13524
H 1.47713 -4.37464 2.45750
H 0.96838 -4.54359 0.02170
H 2.01591 -5.89408 0.50452
H 2.78499 -4.82349 -1.66418
H 4.01904 -4.73201 -0.40192
H 3.82431 -2.51958 -1.56961
H 2.07395 -2.47405 -1.24373
H 4.79698 -2.17370 2.11093
H 5.26872 -3.13215 0.65750
H 4.41609 3.24664 -1.93702
H 4.54212 4.27814 -0.46234
H 3.06180 3.38725 1.62805
H 1.40240 2.94621 1.16312
H 1.52602 5.39588 1.59295
H 2.84669 5.58695 0.43278
H -0.03032 4.69568 -0.22810
H 0.70346 6.25726 -0.64621
H 2.35296 5.12681 -2.14200
H 0.71264 4.66680 -2.61728
H 0.89405 2.49362 -1.38242
H 2.21929 2.64196 -2.55215
H -0.48989 -1.20283 -2.68552
H -2.42915 -4.15204 0.86609
H -1.95634 -5.06886 -1.43994
H -2.71031 -2.06422 2.31285
H -1.07023 -1.35047 2.32576
H -1.45087 1.38628 0.21293
H -1.37365 1.43663 4.94387
H -2.89654 2.32116 4.61028
H -2.93202 0.82778 5.63336
H -6.36620 0.64844 0.08675
H -6.57110 0.51041 1.86608
H -6.36866 2.14325 1.10403
C -3.76323 -0.29327 -1.70782
C -4.55822 -0.00802 -2.82821
C -4.42607 1.21558 -3.50582
C -3.49094 2.15676 -3.04623
C -2.70828 1.87672 -1.91670
H -3.86353 -1.26153 -1.20912
H -5.27505 -0.75679 -3.18290
H -5.04240 1.43081 -4.38515
H -3.37514 3.11586 -3.56310
H -1.98956 2.62137 -1.55472

VI^{Ph}
87
BP86
SCF = -2185.23625240
H(0 K)= -2184.539654
H(413 K)= -2184.457256
G(413 K)= -2184.678412
B97D (1,2-C6Cl2H4) = -2184.82485074
Low Freq. = 5.3790cm-1, 10.5617cm-1

87
VI^{Ph}
C -1.31000 -2.39235 -0.06728
C -1.34993 -1.12039 -0.72093
C -0.78574 -0.97173 -2.02999
C -0.20921 -2.13946 -2.61277
C -0.20143 -3.38564 -1.99138
C -0.76247 -3.50922 -0.69614
Pd 0.73770 -0.24692 -0.63107
C -2.28633 -0.15177 0.01668
C -3.26577 0.62240 -0.85738
F 0.29278 -2.02890 -3.87282
C -2.01457 -2.32370 1.26773
C -2.94158 -1.06429 1.15247
C -3.01651 -0.29869 2.48130
O -2.66956 0.85841 2.67719
C 2.43994 0.58752 0.05598
N 3.58827 -0.02834 0.50904
C 4.61147 0.85361 0.85837
C 4.09998 2.10624 0.66052
N 2.80953 1.91151 0.16520
C 4.08368 -1.42357 0.55056
C 5.37200 -1.17613 1.40964
O 5.76496 0.22385 1.22124
C 4.39912 -1.90718 -0.88768
C 4.83085 -3.38564 -0.93129
C 3.78949 -4.29446 -0.25280

C 3.51991 -3.83514 1.19223
C 3.07272 -2.36108 1.23755
O 4.42063 3.42096 0.82495
C 3.38449 4.14262 0.08029
C 2.12205 3.21398 0.00925
C 1.38743 3.30409 -1.34183
C 0.58784 4.61380 -1.48289
C -0.39924 4.79772 -0.31525
C 0.33086 4.73933 1.03931
C 1.14260 3.43779 1.18867
C -4.35008 -1.51645 0.72665
O -4.60135 -2.54213 0.11034
O -5.28607 -0.59930 1.09023
C -6.62586 -0.88489 0.62080
O -3.49670 -1.11468 3.46184
C -3.60274 -0.49271 4.76444
H 2.10588 -2.23504 0.70632
H 2.90973 -2.02287 2.27753
H 4.43789 -3.97418 1.79970
H 2.74376 -4.46590 1.66129
H 2.84293 -4.25337 -0.82596
H 4.12868 -5.34611 -0.26630
H 4.98892 -3.68892 -1.98170
H 5.81032 -3.51179 -0.42621
H 5.17323 -1.25325 -1.33005
H 3.47492 -1.76606 -1.47983
H 5.15997 -1.33721 2.48414
H 6.22910 -1.79422 1.10480
H 3.76871 4.35476 -0.93594
H 3.20546 5.08786 0.61311
H 1.70788 3.42889 2.13886
H 0.45969 2.56779 1.20960
H -0.38794 4.81839 1.87425
H 1.00225 5.61755 1.12966
H -1.15951 3.99381 -0.35697
H -0.94225 5.75472 -0.41776
H 1.28077 5.47956 -1.51887
H 0.05025 4.61085 -2.44773
H 0.70964 2.42628 -1.39820
H 2.11792 3.18902 -2.16350
H -1.01660 -0.11736 -2.67473
H -0.78347 -4.48888 -0.20692
H 0.21886 -4.24596 -2.51893
H -2.60376 -3.22674 1.49097
H -1.29092 -2.17937 2.09093
H -1.68478 0.59405 0.56619
H -2.61554 -0.14854 5.11205
H -4.28654 0.37012 4.72509
H -3.99873 -1.27239 5.42908
H -6.65627 -0.83727 -0.47924
H -6.94687 -1.88549 0.95024
H -7.26108 -0.10340 1.05866
C -3.88137 0.04231 -1.98748
C -4.78765 0.78042 -2.76652
C -5.09401 2.10911 -2.42932
C -4.49255 2.69318 -1.30253
C -3.58788 1.95466 -0.52436
H -3.65031 -0.99312 -2.25710
H -5.25033 0.31566 -3.64406
H -5.79533 2.68563 -3.04202
H -4.72737 3.72738 -1.02811
H -3.13512 2.39904 0.36866

TS(IV^{Ph}-VI^{Ph}).OPiv
103
BP86
SCF = -2531.68129551
H(0 K)= -2530.856708
H(413 K)= -2530.757986
G(413 K)= -2531.010433
B97D (1,2-C6Cl2H4) = -2531.24943888
Low Freq. = -299.3150cm-1, 14.2681cm-1

103
TS(IV^{Ph}-VI^{Ph}).OPiv
C -1.76454 2.76669 1.64060
C -2.65998 2.80047 0.38991
C -2.09079 3.75332 -0.69154
C -1.80000 5.16413 -0.14133
C -0.89342 5.11064 1.10259
C -1.47710 4.18116 2.18406
C -4.15577 3.12288 0.71641
O -4.97092 2.40155 -0.26379
C -4.20316 1.31377 -0.58120
N -2.86582 1.44888 -0.18230
C -2.05895 0.38012 -0.53124
N -2.98547 -0.42362 -1.17182
C -4.28345 0.10382 -1.19893
O -5.19539 -0.74029 -1.77247
C -4.36641 -1.74865 -2.43774
C -3.02183 -1.82783 -1.64405
C -3.11593 -2.79152 -0.43292
C -3.08323 -4.27154 -0.86479
C -1.85799 -4.58149 -1.74407
C -1.79832 -3.64373 -2.96409
C -1.81421 -2.16493 -2.53145
Pd -0.12239 -0.01111 -0.02565
O -0.81456 -1.56645 1.66940
C -0.52768 -1.11010 2.84171
C -1.13228 -1.87973 4.07908
C 0.03777 -2.29883 4.99750
C 1.23544 1.03846 -1.16087
C 1.25416 2.45081 -0.92651
C 1.52934 3.32842 -1.97230
C 1.88557 2.91058 -3.26143
C 2.01503 1.51910 -3.45998
C 1.71179 0.60387 -2.44520
C 2.00186 -0.86946 -2.51123
C 2.55056 -1.27905 -1.09517
C 1.93036 -2.64469 -0.68672
O 2.06387 -2.89213 0.63794
C 1.35734 -4.04758 1.14323
F 1.43875 4.68226 -1.72181
C 2.16390 -0.17835 -0.02833
C 4.08657 -1.49319 -1.18997
O 4.60968 -1.91454 -0.00537
C 6.04914 -1.99609 0.01324
O 4.77306 -1.31609 -2.18945
O 0.17732 -0.09781 3.10159
C -2.03564 -0.88116 4.84038

C -1.95355 -3.11313 3.66467
O   1.40119 -3.42592 -1.47421
H -0.82312 2.24977 1.36874
H -2.23332 2.13259 2.41377
H -2.41211 4.62952 2.58218
H -0.78455 4.10273 3.04097
H   0.10347 4.74744 0.79363
H -0.74517 6.12762 1.51213
H -1.32260 5.76690 -0.93403
H -2.74943 5.67884 0.11609
H -2.79921 3.80001 -1.53967
H -1.15524 3.30488 -1.06982
H -4.41709 2.76078 1.73055
H -4.40819 4.18940 0.62339
H -4.18319 -1.42408 -3.48156
H -4.93701 -2.68907 -2.43413
H -4.04542 -2.56990 0.12515
H -2.27341 -2.56367 0.24760
H -3.08753 -4.91200 0.03631
H -4.00749 -4.52810 -1.42496
H -0.93054 -4.44117 -1.15966
H -1.87720 -5.63796 -2.07267
H -2.65415 -3.85409 -3.64028
H -0.88032 -3.83714 -3.54577
H -0.90696 -1.93612 -1.93821
H -1.79995 -1.48843 -3.40599
H   1.03715 2.85589 0.06516
H   2.38692 1.14585 -4.42330
H   2.11314 3.64091 -4.04191
H   1.09582 -1.46876 -2.70049
H   2.73630 -1.11846 -3.29384
H   1.69364 -0.69703 0.83697
C   3.21472 0.70718 0.57779
H   6.41980 -2.63252 -0.80702
H   6.48326 -0.98755 -0.08810
H   6.30491 -2.42582 0.99194
H   0.32071 -3.73901 1.35963
H   1.38342 -4.86968 0.41114
H   1.87363 -4.32770 2.07291
H -2.39355 -3.60623 4.55379
H -2.77075 -2.83173 2.98078
H -1.32719 -3.85484 3.14048
H -2.42726 -1.32888 5.77431
H -1.45972 0.02444 5.08975
H -2.90004 -0.57924 4.22124
H -0.33315 -2.74886 5.93870
H   0.68679 -3.04267 4.50032
H   0.65459 -1.41789 5.23648
C   4.24502 1.31355 -0.18139
C   5.20434 2.13265 0.43485
C   5.13826 2.39508 1.81364
C   4.09426 1.83185 2.56823
C   3.14460 0.99564 1.96144
H   4.28481 1.15412 -1.26367
H   5.99611 2.58605 -0.17385
H   5.88440 3.04113 2.29130
H   4.01976 2.03896 3.64267
H   2.31790 0.56903 2.54529

VI^{Ph}.OPiv
103
BP86
SCF =   -2531.73908355
H(0 K)=   -2530.912176
H(413 K)=   -2530.812889
G(413 K)=   -2531.071860
B97D (1,2-C6Cl2H4) =   -2531.30416361
Low   Freq.   =   9.3073cm-1, 14.2789cm-1

103
VI^{Ph}.OPiv
C -3.25122 2.59476 1.78462
C -3.53749 2.57296 0.27126
C -2.42686 3.31967 -0.50978
C -2.15696 4.73461 0.03524
C -1.84961 4.70310 1.54352
C -2.98783 4.01481 2.31967
C -4.97874 3.06955 -0.09062
O -5.45359 2.26256 -1.21621
C -4.74376 1.09333 -1.11275
N -3.66052 1.19114 -0.23020
C -2.85540 0.05559 -0.17499
N -3.54409 -0.75389 -1.07462
C -4.68716 -0.16516 -1.63029
O -5.38335 -1.02060 -2.44531
C -4.44797 -2.12321 -2.68335
C -3.46215 -2.17408 -1.46749
C -3.97972 -3.06935 -0.31235
C -3.81694 -4.57289 -0.60642
C -2.35838 -4.91787 -0.96030
C -1.86264 -4.06732 -2.14452
C -2.02607 -2.56329 -1.86131
Pd -1.25898 -0.29532 0.85232
O   0.56378 -0.65938 1.87187
C   0.95044 -1.20468 2.98378
C -0.13803 -1.52763 4.07296
C   0.54411 -2.07010 5.34428
C   2.52376 1.80020 -0.74414
C   2.92545 3.05824 -0.27601
C   2.36276 4.18804 -0.88455
C   1.42887 4.10147 -1.92361
C   1.02903 2.82746 -2.37241
C   1.57895 1.68237 -1.78397
C   1.28247 0.22771 -2.05814
C   2.50632 -0.52358 -1.44542
C   2.11369 -1.94935 -1.02142
O   2.70123 -2.30043 0.13665
C   2.30738 -3.56051 0.72188
F   2.75232 5.42725 -0.45662
C   2.95176 0.42944 -0.23577
C   3.62999 -0.60842 -2.49125
O   4.44979 -1.67357 -2.24990
C   5.59345 -1.76319 -3.12356
O   3.80826 0.17469 -3.41580
O   2.14831 -1.49051 3.24610
C -0.92583 -0.24097 4.40805
C -1.11528 -2.58686 3.51202
O   1.36462 -2.66500 -1.68123
H -2.36266 1.94355 1.93849
H -4.08936 2.11173 2.32079

H -3.90829 4.63215 2.24306
H -2.74319 3.95940 3.39616
H -0.90995 4.14201 1.70760
H -1.68365 5.72766 1.92590
H -1.31445 5.18279 -0.52160
H -3.03512 5.39023 -0.14502
H -2.69792 3.35169 -1.58178
H -1.51250 2.70242 -0.42363
H -5.66417 2.91874 0.76730
H -5.01438 4.12238 -0.41052
H -3.89153 -1.91979 -3.61970
H -5.05062 -3.03669 -2.80263
H -5.03562 -2.81603 -0.10035
H -3.39017 -2.79142 0.58223
H -4.15742 -5.15860 0.26774
H -4.47585 -4.86978 -1.44950
H -1.71696 -4.71570 -0.08096
H -2.26072 -5.99617 -1.18803
H -2.42436 -4.35443 -3.05912
H -0.79679 -4.26747 -2.34726
H -1.37138 -2.25705 -1.01738
H -1.70676 -1.95930 -2.72973
H 3.66270 3.17133 0.52377
H 0.30176 2.74137 -3.18735
H 1.03881 5.01888 -2.37402
H 0.36887 -0.07367 -1.49743
H 1.13780 -0.00902 -3.12396
H 2.27576 0.14191 0.60050
C 4.38761 0.33668 0.26090
H 5.28605 -1.70264 -4.17999
H 6.29998 -0.94498 -2.90634
H 6.05728 -2.73475 -2.90251
H 1.27318 -3.80202 0.43464
H 2.98462 -4.35843 0.36943
H 2.39715 -3.40136 1.80556
H -1.90514 -2.81848 4.25302
H -1.59480 -2.20583 2.58759
H -0.58624 -3.52784 3.27216
H -1.71073 -0.45083 5.16060
H -0.25837 0.53920 4.81769
H -1.40687 0.15396 3.49331
H -0.21314 -2.30679 6.11677
H 1.12200 -2.98231 5.12367
H 1.25113 -1.33276 5.75932
C 5.45783 0.90764 -0.46394
C 6.77466 0.82820 0.01719
C 7.03970 0.18597 1.23850
C 5.97781 -0.36939 1.97134
C 4.65901 -0.29763 1.49215
H 5.25351 1.42521 -1.40813
H 7.59232 1.27977 -0.55755
H 8.06640 0.12870 1.61962
H 6.17280 -0.86182 2.93125
H 3.82662 -0.73043 2.06924

R = OMe (8g)

III^{OMe}
98
BP86
SCF = -2415.75457196
H(0 K)= -2414.960070
H(413 K)= -2414.865123
G(413 K)= -2415.111402
B97D (1,2-C6Cl2H4) = -2415.33560332
Low Freq. = 4.9536cm-1, 14.5239cm-1

98
III^{OMe}
C 0.98220 -1.96379 -0.52989
C -0.17629 -1.34791 -1.07987
C -0.93732 -2.05789 -2.03930
C -0.59582 -3.36858 -2.38385
C 0.48995 -4.02700 -1.80092
C 1.26944 -3.30314 -0.88891
Pd -0.92710 0.47115 -0.72033
O -1.77227 2.51019 -1.13543
C -1.47816 2.32937 -2.37409
C -1.90324 3.36470 -3.42798
F -1.37856 -4.03719 -3.28048
C 1.93252 -1.23462 0.40333
C 3.30519 -0.84422 -0.24682
C 4.11271 -2.08691 -0.66996
O 4.46799 -2.35986 -1.80576
C -1.27490 0.09805 1.19839
N -2.08022 -0.82679 1.82955
C -2.14926 -0.63124 3.21508
C -1.36973 0.45338 3.48655
N -0.83378 0.86150 2.26177
C -2.94708 -1.98668 1.45969
C -3.79406 -2.05383 2.77776
O -2.95479 -1.52540 3.85043
C -2.10612 -3.27187 1.26177
C -2.98028 -4.47510 0.84813
C -3.84982 -4.15943 -0.38383
C -4.69900 -2.89473 -0.15378
C -3.82235 -1.68437 0.22987
O -1.02566 1.25509 4.52825
C 0.04775 2.09682 3.98800
C -0.19983 2.20444 2.44973
C 1.09891 2.37053 1.64901
C 1.67999 3.79258 1.78681
C 0.65072 4.86837 1.39584
C -0.63358 4.72425 2.23252
C -1.23247 3.30891 2.10265
O -0.86815 1.24772 -2.72858
C 4.14793 -0.08443 0.80439
O 3.78122 0.24821 1.92204
C 3.14232 0.04781 -1.49857
O 2.69906 1.33398 -1.07250
C 2.51003 2.20780 -2.18437
O 5.39452 0.16859 0.31659
C 6.26055 0.89480 1.22018
O 4.39504 -2.86864 0.41669
C 5.18456 -4.04447 0.11552
H -3.15209 -1.42398 -0.60807
H -4.44077 -0.79081 0.43260
H -5.44345 -3.08929 0.64511
H -5.28097 -2.64804 -1.05947
H -3.19921 -4.01092 -1.26525

H -4.50429 -5.01889 -0.61635
H -2.32492 -5.34083 0.64739
H -3.63623 -4.77834 1.68915
H -1.55642 -3.48960 2.19589
H -1.35576 -3.07366 0.47928
H -4.69959 -1.42178 2.69283
H -4.07834 -3.07619 3.06293
H 1.01727 1.60570 4.19487
H -0.00691 3.06160 4.51118
H -2.11764 3.19633 2.75598
H -1.56270 3.14535 1.06192
H -1.39006 5.46573 1.91921
H -0.40571 4.95101 3.29395
H 0.39623 4.75598 0.32424
H 1.08027 5.87909 1.52041
H 2.01576 3.96674 2.82955
H 2.58423 3.86652 1.15774
H 0.87649 2.16397 0.58483
H 1.83877 1.61628 1.96640
H -1.80287 -1.59998 -2.52805
H 2.12629 -3.80039 -0.42098
H 0.71691 -5.06311 -2.06498
H 1.46337 -0.30028 0.74225
H 2.15335 -1.83981 1.29889
H 2.40990 -0.42045 -2.18663
H 4.11373 0.12283 -2.02532
H 4.64641 -4.70453 -0.58382
H 6.14594 -3.75744 -0.33922
H 5.34218 -4.54567 1.08001
H 7.21758 0.99480 0.69064
H 5.83621 1.88587 1.44653
H 6.39071 0.33915 2.16250
H 2.22116 3.18856 -1.77494
H 3.44601 2.32426 -2.77100
H 1.70334 1.84290 -2.85003
C -1.62569 4.78633 -2.88832
C -1.15451 3.13190 -4.75528
C -3.43057 3.18371 -3.64363
H -1.32892 2.11478 -5.13918
H -1.49902 3.85807 -5.51253
H -0.06616 3.25996 -4.62973
H -1.99016 5.54121 -3.60700
H -2.13151 4.94522 -1.92333
H -0.54434 4.95347 -2.74165
H -3.98067 3.33177 -2.69997
H -3.79556 3.92052 -4.38082
H -3.66020 2.17460 -4.02662

TS(III^{OMe}-IV^{OMe})1
98
BP86
SCF = -2415.73085922
H(0 K)= -2414.938146
H(413 K)= -2414.843931
G(413 K)= -2415.089545
B97D (1,2-C6Cl2H4) = -2415.31629843
Low Freq. = -30.0677cm-1, 10.5954cm-1

98

TS(III^{OMe}-IV^{OMe})1
C 3.47487 2.39416 -1.24984
C 3.35947 0.99606 -1.91028
C 2.27707 0.97556 -3.00425
C 2.47569 2.10393 -4.03632
C 2.54555 3.48380 -3.35668
C 3.65767 3.51497 -2.29250
C 4.74341 0.45568 -2.40151
O 5.37978 -0.19022 -1.24890
C 4.33681 -0.60069 -0.47711
N 3.11517 -0.03108 -0.85945
C 2.08734 -0.37066 -0.01762
N 2.70214 -1.19511 0.88496
C 4.06887 -1.35938 0.62633
O 4.67900 -2.20147 1.50273
C 3.73588 -2.30186 2.62069
C 2.30072 -2.03999 2.04433
C 1.39928 -1.26669 3.02588
C 0.92197 -2.15716 4.19050
C 0.21401 -3.42345 3.67469
C 1.12894 -4.21610 2.72327
C 1.62038 -3.34137 1.55235
Pd 0.23182 0.25384 -0.03968
O 0.79684 2.16232 0.78415
C -0.03830 2.32885 1.77451
C 0.11033 3.60682 2.64068
C -0.38075 -1.46452 -0.90112
C -1.68740 -1.95606 -0.59899
C -2.14861 -3.13138 -1.23411
C -1.37244 -3.83504 -2.16533
C -0.10402 -3.32985 -2.45824
C 0.39870 -2.18001 -1.83972
C -2.62551 -1.24499 0.36320
C -3.48717 -0.11734 -0.30349
C -4.36403 -0.69627 -1.43302
O -5.23912 -1.61717 -0.93148
C -6.11559 -2.20489 -1.92173
F 0.68028 -3.98515 -3.36039
C -2.54013 0.94497 -0.90790
O -3.17135 2.19647 -1.04394
C -2.40881 3.08993 -1.85175
C -4.39434 0.49749 0.79028
O -5.46909 1.11995 0.23629
C -6.35155 1.75042 1.19331
O -4.19139 0.42633 1.99149
O -0.94107 1.49329 2.04472
O -4.28678 -0.41481 -2.61862
H 0.52543 -0.88188 2.46518
H 1.94188 -0.37938 3.39912
H 1.78392 -2.44939 4.82474
H 0.24697 -1.57006 4.83694
H -0.70671 -3.12854 3.13522
H -0.10269 -4.05871 4.52121
H 0.60232 -5.09790 2.31755
H 1.99329 -4.61266 3.29371
H 2.32501 -3.89962 0.90897
H 0.76360 -3.05295 0.91822
H 4.00136 -1.53354 3.37172
H 3.85703 -3.30489 3.05309
H 4.60952 -0.30116 -3.19821
H 5.42650 1.24510 -2.74470

H 4.31928 2.38288 -0.53605
H 2.55652 2.56690 -0.65949
H 3.67454 4.48997 -1.77436
H 4.64261 3.41841 -2.79304
H 1.57323 3.70049 -2.87320
H 2.71031 4.27451 -4.11046
H 3.40734 1.92962 -4.61189
H 1.65151 2.07086 -4.77055
H 1.29665 1.09118 -2.50041
H 2.26393 -0.01349 -3.49682
H 1.40901 -1.85807 -2.10459
H -3.14816 -3.50486 -0.98125
H -1.73047 -4.74718 -2.64999
H -3.32059 -1.96881 0.81821
H -2.07409 -0.75985 1.18773
H -2.16942 0.57248 -1.88081
H -1.66974 1.05917 -0.18351
H -5.81410 2.53050 1.75539
H -7.16213 2.19066 0.59692
H -6.74743 1.00784 1.90471
H -5.53061 -2.72132 -2.69939
H -6.74584 -2.91579 -1.37017
H -6.73233 -1.42701 -2.39947
H -2.97019 4.03549 -1.89919
H -1.40893 3.28167 -1.41068
H -2.27809 2.69069 -2.87860
C 0.44839 3.15046 4.08201
C 1.21353 4.54097 2.10900
C -1.25485 4.33619 2.64637
H 0.49934 4.02287 4.75795
H -0.32205 2.45758 4.45591
H 1.42591 2.63718 4.11850
H 1.29370 5.43694 2.75104
H 2.19342 4.03646 2.09550
H 0.99430 4.87443 1.08113
H -1.22013 5.20974 3.32193
H -1.51706 4.69871 1.63681
H -2.05162 3.65483 2.98293

Int(III^{OMe}-IV^{OMe})
98
BP86
SCF = -2415.73144684
H(0 K)= -2414.938348
H(413 K)= -2414.843091
G(413 K)= -2415.091457
B97D (1,2-C6Cl2H4) = -2415.31884715
Low Freq. = 12.8994cm-1, 18.2328cm-1

98
Int(III^{OMe}-IV^{OMe})
C 3.61537 2.03740 -1.35308
C 3.42460 0.59449 -1.88783
C 2.39531 0.54796 -3.03100
C 2.72329 1.55831 -4.14907
C 2.86659 2.98727 -3.59433
C 3.93009 3.03846 -2.48259
C 4.78450 -0.08604 -2.25556
O 5.32143 -0.65582 -1.01590
C 4.21843 -0.91678 -0.26278
N 3.05821 -0.30903 -0.75960
C 1.97362 -0.49605 0.05923
N 2.48929 -1.26195 1.06813
C 3.85052 -1.54058 0.89513
O 4.36264 -2.33085 1.87546
C 3.36713 -2.26385 2.94991
C 1.97989 -1.95559 2.28498
C 1.10762 -1.02299 3.14644
C 0.51670 -1.75576 4.36724
C -0.26857 -3.01056 3.94359
C 0.61606 -3.96010 3.11497
C 1.21986 -3.24538 1.88923
Pd 0.15845 0.21930 -0.15197
O 0.79661 2.17708 0.45037
C 0.18624 2.50845 1.56292
C 0.55009 3.89246 2.17551
C -0.49850 -1.55350 -0.87660
C -1.81594 -2.00083 -0.55328
C -2.30677 -3.19142 -1.13489
C -1.54405 -3.96040 -2.02506
C -0.25913 -3.50667 -2.32877
C 0.26848 -2.33843 -1.76735
C -2.73154 -1.23552 0.38790
C -3.45182 -0.00870 -0.26683
C -4.30384 -0.45890 -1.46958
O -5.27979 -1.32197 -1.06586
C -6.13010 -1.80103 -2.13496
F 0.51499 -4.22876 -3.18729
C -2.38000 0.98816 -0.76519
O -2.89299 2.28887 -0.92319
C -2.06502 3.09617 -1.76366
C -4.34273 0.65218 0.81154
O -5.35902 1.35259 0.24506
C -6.20818 2.04379 1.19178
O -4.16156 0.55916 2.01539
O -0.65171 1.77958 2.14238
O -4.12451 -0.13512 -2.63355
H 0.29061 -0.61981 2.51819
H 1.70564 -0.14861 3.45992
H 1.32672 -2.04763 5.06696
H -0.13476 -1.05764 4.92047
H -1.14231 -2.70228 3.33755
H -0.66656 -3.53373 4.83152
H 0.03749 -4.83607 2.77205
H 1.42422 -4.36181 3.75943
H 1.90285 -3.91569 1.33560
H 0.41342 -2.95921 1.19108
H 3.65678 -1.45163 3.64335
H 3.39528 -3.22974 3.47346
H 4.62959 -0.90746 -2.98140
H 5.53749 0.61656 -2.63881
H 4.42331 2.03356 -0.59805
H 2.68528 2.33005 -0.83159
H 3.99809 4.05405 -2.05451
H 4.92594 2.82473 -2.92143
H 1.89328 3.31681 -3.18210
H 3.12343 3.68992 -4.40726
H 3.66358 1.26526 -4.65857
H 1.93176 1.51446 -4.91783
H 1.40355 0.77796 -2.59293

```
H   2.33246 -0.47864 -3.43474
H   1.28871 -2.05687 -2.03969
H  -3.31780 -3.52539 -0.87251
H  -1.92398 -4.88483 -2.46792
H  -3.51128 -1.90648  0.78224
H  -2.17743 -0.83782  1.25598
H  -1.96968  0.60113 -1.71640
H  -1.59811  1.02660  0.07410
H  -5.62119  2.77512  1.76933
H  -6.97052  2.54954  0.58409
H  -6.67374  1.32875  1.88887
H  -5.53473 -2.33490 -2.89250
H  -6.85008 -2.47799 -1.65533
H  -6.65010 -0.95922 -2.61923
H  -2.53864  4.08840 -1.81275
H  -1.04395  3.19502 -1.34489
H  -2.00333  2.66995 -2.78570
C   1.18793  3.62569  3.56150
C   1.52586  4.69087  1.29125
C  -0.76666  4.68250  2.36675
H   1.40069  4.57933  4.07741
H   0.50552  3.02908  4.18759
H   2.14086  3.07529  3.46289
H   1.75110  5.66681  1.75889
H   2.47569  4.14899  1.15198
H   1.09811  4.88030  0.29271
H  -0.57029  5.63856  2.88475
H  -1.23771  4.91349  1.39510
H  -1.48193  4.09290  2.96096
```

TS(III^{OMe}-IV^{OMe})2

```
98
BP86
SCF  =          -2415.71080838
H(0 K)=         -2414.922076
H(413 K)=       -2414.827788
G(413 K)=       -2415.071873
B97D  (1,2-C6Cl2H4)  =         -
2415.28719964
Low   Freq.   =   -1091.6782cm-1,
13.2131cm-1
```

98

TS(III^{OMe}-IV^{OMe})2

```
C   3.39406  2.53661 -0.99114
C   3.42799  1.12757 -1.63770
C   2.46828  1.03549 -2.83752
C   2.70573  2.16425 -3.86065
C   2.62326  3.55123 -3.19743
C   3.61646  3.65786 -2.02590
C   4.88562  0.66963 -1.97635
O   5.43375  0.06686 -0.75761
C   4.33881 -0.40730 -0.10089
N   3.13417  0.09550 -0.60648
C   2.03200 -0.31639  0.10400
N   2.60252 -1.11716  1.06045
C   3.99631 -1.19834  0.95786
O   4.55474 -2.01735  1.89098
C   3.49462 -2.19678  2.88686
C   2.12855 -2.02785  2.13804
C   1.04487 -1.36449  3.00782
C   0.49414 -2.32574  4.08009
C  -0.02948 -3.62922  3.44900
C   1.06477 -4.31309  2.60902
C   1.62795 -3.36324  1.53285
Pd  0.09835  0.20101 -0.16323
O   0.49069  2.15569  0.75931
C  -0.43878  2.44185  1.58631
C  -0.33141  3.69580  2.47577
C  -0.33343 -1.58036 -1.01747
C  -1.58462 -2.20899 -0.75094
C  -1.90587 -3.42259 -1.39562
C  -1.03156 -4.03695 -2.30542
C   0.18429 -3.40054 -2.56069
C   0.54452 -2.20166 -1.93239
C  -2.58777 -1.59737  0.20807
C  -3.22441 -0.26738 -0.30900
C  -4.09040 -0.56132 -1.55487
O  -5.11522 -1.40730 -1.23948
C  -5.97502 -1.74794 -2.35197
F   1.05997 -3.96784 -3.43949
C  -2.07812  0.72291 -0.68970
O  -2.53687  2.07010 -0.83925
C  -2.26022  2.62851 -2.12400
C  -4.12752  0.28802  0.81363
O  -5.05047  1.15552  0.31842
C  -5.91903  1.73612  1.31763
O  -4.04292 -0.00945  1.99659
O  -1.49832  1.71743  1.72005
O  -3.89202 -0.15071 -2.68971
H   0.22615 -1.04344  2.33401
H   1.45370 -0.44552  3.46603
H   1.28710 -2.56534  4.81776
H  -0.30790 -1.81699  4.64285
H  -0.89473 -3.39479  2.79931
H  -0.39749 -4.31449  4.23349
H   0.67045 -5.22022  2.11808
H   1.87783 -4.65790  3.27970
H   2.45478 -3.84151  0.97647
H   0.83981 -3.12396  0.79738
H   3.61173 -1.42053  3.66753
H   3.63317 -3.19387  3.32770
H   4.88026 -0.09637 -2.77569
H   5.55365  1.49681 -2.25442
H   4.15873  2.58229 -0.19379
H   2.41117  2.66009 -0.50231
H   3.52431  4.63722 -1.52372
H   4.65144  3.61497 -2.42185
H   1.59467  3.71217 -2.81962
H   2.81732  4.34471 -3.94127
H   3.70072  2.04130 -4.33451
H   1.96503  2.07591 -4.67482
H   1.43482  1.09719 -2.44057
H   2.56483  0.04359 -3.31488
H   1.52061 -1.77021 -2.16888
H  -2.86840 -3.90022 -1.17524
H  -1.27852 -4.97780 -2.80439
H  -3.40119 -2.31019  0.41930
H  -2.11404 -1.35338  1.17448
H  -1.73468  0.36610 -1.67719
H  -1.52741  1.00485  0.66991
H  -5.33052  2.30059  2.05798
```

H -6.59340 2.40499 0.76569
H -6.48937 0.95130 1.84066
H -5.39862 -2.24127 -3.15072
H -6.73204 -2.42922 -1.93976
H -6.44952 -0.84298 -2.76421
H -2.67951 3.64770 -2.12148
H -1.16712 2.69645 -2.30930
H -2.73159 2.03274 -2.92735
C -0.48057 3.25343 3.95318
C 1.01736 4.40927 2.26665
C -1.50150 4.64065 2.10039
H -0.45837 4.13837 4.61312
H -1.43101 2.71961 4.10798
H 0.34491 2.58530 4.25524
H 1.07364 5.29939 2.91752
H 1.86428 3.74776 2.51206
H 1.13858 4.73736 1.22171
H -1.48237 5.53453 2.74847
H -1.42345 4.97518 1.05204
H -2.46914 4.13106 2.22585

IV^{OMe}

98
BP86
SCF = -2415.73205349
H(0 K)= -2414.938617
H(413 K)= -2414.843792
G(413 K)= -2415.089150
B97D (1,2-C6Cl2H4) = -2415.31009897
Low Freq. = 12.9997cm-1, 16.8820cm-1

98
IV^{OMe}
C 3.66653 1.85634 -1.21113
C 3.51679 0.37301 -1.63116
C 2.59909 0.23480 -2.85958
C 3.03410 1.14912 -4.02259
C 3.13633 2.61977 -3.57967
C 4.09251 2.76363 -2.38196
C 4.90040 -0.33487 -1.81914
O 5.30692 -0.83281 -0.50346
C 4.12761 -1.04224 0.14822
N 3.03420 -0.42699 -0.47413
C 1.84793 -0.56251 0.21714
N 2.26510 -1.31543 1.29066
C 3.62862 -1.63004 1.27374
O 4.01354 -2.41200 2.32223
C 2.89168 -2.32429 3.26026
C 1.60827 -2.03729 2.41028
C 0.58428 -1.14929 3.13914
C -0.16089 -1.91362 4.25148
C -0.83159 -3.18794 3.70521
C 0.19603 -4.09545 3.00438
C 0.95012 -3.34102 1.89136
Pd -0.07884 0.11866 -0.30779
O 0.55486 2.17612 0.39618
C 0.14234 2.84774 1.36044
C 0.77659 4.18669 1.78155
C -0.66027 -1.66509 -1.03075
C -1.95785 -2.16891 -0.73137
C -2.38882 -3.39891 -1.26638
C -1.56176 -4.16472 -2.10408
C -0.28958 -3.66223 -2.38607
C 0.16991 -2.44606 -1.86457
C -2.85569 -1.37608 0.18978
C -3.21214 0.03557 -0.38653
C -4.32426 -0.06380 -1.46181
O -5.31983 -0.92008 -1.08041
C -6.39635 -1.04094 -2.03759
F 0.54375 -4.38513 -3.19189
C -1.95257 0.73723 -1.00051
O -2.16962 2.16258 -0.86130
C -1.72537 2.90031 -1.99179
C -3.69085 0.89526 0.78496
O -4.90719 1.44736 0.59929
C -5.36738 2.30406 1.67455
O -3.04435 1.06205 1.82394
O -0.87336 2.50664 2.14711
O -4.33283 0.52129 -2.53541
H -0.13634 -0.79095 2.37663
H 1.09305 -0.25415 3.54144
H 0.54593 -2.18780 5.06119
H -0.91112 -1.24590 4.71069
H -1.61705 -2.90113 2.97941
H -1.33695 -3.73565 4.52109
H -0.29919 -4.98153 2.56948
H 0.91203 -4.48395 3.75698
H 1.72706 -3.98225 1.43642
H 0.24884 -3.06295 1.08432
H 3.08685 -1.49326 3.96593
H 2.85442 -3.27678 3.80759
H 4.80649 -1.19624 -2.50850
H 5.69633 0.33784 -2.16927
H 4.39118 1.92177 -0.37809
H 2.68719 2.18354 -0.81943
H 4.13017 3.81183 -2.03497
H 5.12297 2.51299 -2.70576
H 2.13053 2.98164 -3.28918
H 3.47208 3.25285 -4.42071
H 4.01552 0.81663 -4.41784
H 2.31598 1.04164 -4.85458
H 1.56925 0.49024 -2.53591
H 2.57529 -0.82059 -3.18479
H 1.18554 -2.13009 -2.11464
H -3.39124 -3.76913 -1.01848
H -1.88408 -5.12067 -2.52540
H -3.78488 -1.92506 0.41296
H -2.32839 -1.18751 1.14114
H -1.97276 0.49665 -2.07984
H -1.41686 1.73979 1.79056
H -4.65661 3.12896 1.83566
H -6.34175 2.68584 1.34222
H -5.47078 1.72862 2.60791
H -6.01528 -1.39598 -3.00838
H -7.09562 -1.76916 -1.60364
H -6.89170 -0.06790 -2.18759
H -1.95293 3.96201 -1.79849
H -0.63125 2.79111 -2.14484
H -2.25575 2.57669 -2.90969
C 1.25582 4.06570 3.25167

C 1.96404 4.53242 0.86622
C -0.31018 5.28906 1.67965
H 1.69757 5.02389 3.57585
H 0.41952 3.82096 3.92457
H 2.02814 3.28416 3.35691
H 2.38649 5.50804 1.16190
H 2.76139 3.77595 0.94001
H 1.65169 4.59572 -0.18863
H 0.11729 6.25870 1.98863
H -0.67696 5.39281 0.64423
H -1.16903 5.06060 2.32928

V^{OMe}.OPiv
97
BP86
SCF = -2415.19170822
H(0 K)= -2414.412595
H(413 K)= -2414.317953
G(413 K)= -2414.562762
B97D (1,2-C6Cl2H4) = -2414.82828640
Low Freq. = 12.4059cm-1, 16.6191cm-1

97
V^{OMe}.OPiv
C -0.36911 -2.45142 -1.88833
C -1.03374 -1.55394 -1.01789
C -2.38752 -1.85585 -0.68285
C -3.02354 -2.99021 -1.22102
C -2.35929 -3.86348 -2.10061
C -1.03188 -3.56633 -2.41365
Pd -0.16676 0.12643 -0.32659
C -1.97883 1.04921 -0.84367
H -1.83763 2.12756 -0.62955
C -3.11413 -0.99164 0.32539
C -3.21368 0.52939 -0.02673
C -3.37322 1.29910 1.30407
O -3.67600 0.78087 2.37557
F -0.34850 -4.40642 -3.26268
C 1.63881 -0.78438 0.18064
N 2.85883 -0.77772 -0.46628
C 3.88133 -1.41797 0.24587
C 3.29001 -1.92124 1.36483
N 1.94816 -1.53287 1.28829
C 3.43398 -0.05973 -1.62444
C 4.80018 -0.82373 -1.69974
O 5.10730 -1.30463 -0.35473
C 2.58002 -0.24466 -2.89272
C 3.11480 0.57218 -4.08565
C 3.25092 2.06473 -3.73147
C 4.13902 2.25242 -2.48769
C 3.60313 1.44303 -1.29116
O 3.57385 -2.64352 2.49074
C 2.44247 -2.35275 3.37801
C 1.20816 -2.03979 2.46523
C 0.41616 -3.31762 2.08787
C -0.41513 -3.85594 3.26997
C -1.33572 -2.77049 3.85929
C -0.53491 -1.51365 4.25131
C 0.27758 -0.96893 3.06142
O 0.50154 2.12761 0.23905
C 1.30188 2.43783 1.21568
O 1.93167 1.66167 1.96969
C 1.43072 3.99333 1.44196
C 0.09523 4.48189 2.05086
C 2.58895 4.29334 2.41130
C 1.66123 4.71089 0.09484
C -4.48901 0.85771 -0.84781
O -5.04400 1.94725 -0.91609
O -4.95144 -0.24160 -1.51831
C -6.04991 0.03681 -2.40359
O -3.19304 2.64137 1.17500
C -3.44948 3.38534 2.38256
H -0.40548 -0.64972 2.24743
H 0.86777 -0.07272 3.31665
H 0.14085 -1.75708 5.09876
H -1.21724 -0.72523 4.61521
H -2.09441 -2.48839 3.10472
H -1.88796 -3.16743 4.73203
H -1.00633 -4.72779 2.93471
H 0.25954 -4.23213 4.06741
H 1.11865 -4.08756 1.71752
H -0.24951 -3.05468 1.24544
H 2.69624 -1.46924 3.99460
H 2.30515 -3.23502 4.02077
H 4.71674 -1.69618 -2.37870
H 5.64096 -0.18839 -2.01672
H 4.25837 1.54768 -0.40830
H 2.60675 1.82049 -0.99805
H 4.19567 3.32070 -2.20986
H 5.17737 1.94353 -2.73133
H 2.24592 2.47846 -3.52162
H 3.65885 2.62967 -4.59068
H 4.10407 0.17930 -4.40127
H 2.44037 0.43774 -4.95072
H 1.54950 0.07651 -2.63882
H 2.52518 -1.31924 -3.14574
H 0.67822 -2.29694 -2.16490
H -4.06664 -3.19676 -0.94895
H -2.84563 -4.74629 -2.52659
H -4.12081 -1.39276 0.53321
H -2.56333 -1.03648 1.28072
O -2.36637 0.92999 -2.23985
H -2.76430 3.07532 3.18787
H -3.28009 4.43844 2.11837
H -4.48799 3.23023 2.72028
H -5.75816 0.78626 -3.15789
H -6.28973 -0.92213 -2.88555
H -6.92192 0.42224 -1.84763
H 2.64816 5.37784 2.62636
H 2.45428 3.74539 3.35701
H 3.55536 3.97219 1.98599
H 1.69547 5.80907 0.23320
H 2.62006 4.40198 -0.36070
H 0.85364 4.46357 -0.61143
H 0.11066 5.57741 2.21217
H -0.74100 4.23200 1.37819
H -0.09059 3.99736 3.02634
C -1.45613 1.58742 -3.09340
H -0.44424 1.13107 -3.03021
H -1.83917 1.49033 -4.12456

H -1.35719 2.66836 -2.84302

V^OMe^

81
BP86
SCF = -2068.67494862
H(0 K)= -2068.026842
H(413 K)= -2067.950246
G(413 K)= -2068.153315
B97D (1,2-C6Cl2H4) = -2068.33595280
Low Freq. = -3.0770cm-1, 17.1365cm-1

81
V^OMe^

```
C  -2.14238  1.26286  1.34458
C  -0.86869  0.65778  1.51833
C  -0.14590  0.88031  2.70806
C  -0.69722  1.69484  3.70746
C  -1.94402  2.30736  3.56366
C  -2.65348  2.08153  2.37219
Pd -0.11316 -0.49370  0.07585
C  -2.00478 -1.32136  0.15854
O  -2.59681 -1.75403  1.37379
F   0.01281  1.89331  4.85615
C  -2.92948  1.10618  0.05510
C  -3.01584 -0.36352 -0.50836
C  -2.70281 -0.32923 -2.01246
O  -1.67217 -0.72222 -2.55021
C   1.87450  0.08899 -0.29112
N   2.42306  1.27302 -0.73389
C   3.80567  1.22650 -0.92673
C   4.17735 -0.05422 -0.62493
N   3.00490 -0.69921 -0.22895
C   1.94175  2.66617 -0.91232
C   3.17693  3.22411 -1.69886
O   4.33913  2.42026 -1.30566
C   1.76645  3.34659  0.46930
C   1.18716  4.77020  0.35094
C  -0.12991  4.77464 -0.44635
C   0.07084  4.14331 -1.83669
C   0.63697  2.71332 -1.72869
O   5.26572 -0.87483 -0.63402
C   4.83507 -2.05367  0.12222
C   3.27295 -2.13640  0.00745
C   2.60127 -2.63105  1.30274
C   2.78621 -4.14669  1.51283
C   2.28499 -4.94943  0.29779
C   2.98084 -4.48474 -0.99475
C   2.80773 -2.96927 -1.21489
C  -4.45611 -0.90695 -0.31412
O  -5.36715 -0.36038  0.28611
O  -4.57659 -2.12488 -0.92094
C  -5.87418 -2.74092 -0.76342
O  -3.72078  0.25602 -2.70354
C  -3.50877  0.36007 -4.13126
H  -0.08984  2.05296 -1.21643
H   0.81335  2.27363 -2.72737
H   0.75642  4.77840 -2.43436
H  -0.88317  4.11597 -2.39296
H  -0.89210  4.19651  0.11100
H  -0.51717  5.80473 -0.54470
H   1.03134  5.18233  1.36342
H   1.91766  5.44172 -0.14474
H   2.74032  3.35805  0.99288
H   1.08228  2.71703  1.06650
H   3.02942  3.11281 -2.79032
H   3.41432  4.27048 -1.45967
H   5.14002 -1.92326  1.17830
H   5.35551 -2.92067 -0.30954
H   3.35350 -2.63178 -2.11493
H   1.73836 -2.73863 -1.38311
H   2.58410 -5.02862 -1.87027
H   4.05885 -4.73801 -0.94090
H   1.19140 -4.80669  0.19438
H   2.45015 -6.03019  0.45600
H   3.85712 -4.37586  1.68782
H   2.25339 -4.45566  2.42937
H   1.52114 -2.38204  1.22857
H   2.99615 -2.05723  2.16084
H   0.83863  0.43325  2.87192
H  -3.63621  2.55025  2.24168
H  -2.34475  2.93375  4.36497
H  -3.95910  1.47514  0.18917
H  -2.46022  1.74119 -0.72092
H  -1.82588 -2.21024 -0.49814
H  -2.62339  0.97852 -4.34942
H  -3.36388 -0.63795 -4.57453
H  -4.41806  0.83249 -4.52674
H  -6.08487 -2.92643  0.30206
H  -6.66569 -2.09318 -1.17390
H  -5.81977 -3.68791 -1.31799
C  -1.80195 -2.69154  2.08142
H  -2.38534 -3.01364  2.95856
H  -1.56775 -3.58073  1.45496
H  -0.84990 -2.23091  2.41379
```

TS(V^OMe^-VI^OMe^)

81
BP86
SCF = -2068.65681754
H(0 K)= -2068.009421
H(413 K)= -2067.932119
G(413 K)= -2068.140405
B97D (1,2-C6Cl2H4) = -2068.31211844
Low Freq. = -291.1497cm-1, 7.7772cm-1

81
TS(V^OMe^-VI^OMe^)

```
C   2.01931  2.09459  0.48221
C   1.30628  1.54475 -0.61037
C   1.02374  2.36037 -1.73382
C   1.36843  3.71606 -1.68189
C   2.01899  4.29045 -0.58233
C   2.36067  3.45575  0.49544
Pd -0.05474  0.02415 -0.40018
C   2.12374 -0.29548 -0.62101
O   2.93860 -0.15612 -1.76287
F   1.05627  4.50902 -2.74747
```

```
C   2.46376  1.08569  1.50934
C   2.94669 -0.16314  0.69469
C   2.78958 -1.44612  1.53990
O   2.17164 -2.45966  1.24236
C  -2.03026 -0.37308 -0.04022
N  -3.02284  0.45116  0.43953
C  -4.27003 -0.16827  0.55443
C  -4.07544 -1.46422  0.16772
N  -2.73152 -1.54780 -0.20346
C  -3.14195  1.89738  0.74517
C  -4.55055  1.86672  1.43721
O  -5.25912  0.67950  0.95179
C  -3.13020  2.72000 -0.56758
C  -3.17946  4.23872 -0.30755
C  -2.05707  4.68618  0.64729
C  -2.10246  3.88766  1.96331
C  -2.02816  2.37076  1.69940
O  -4.73221 -2.65569  0.08231
C  -3.83373 -3.51164 -0.69649
C  -2.37893 -2.95288 -0.51796
C  -1.54128 -3.05266 -1.80729
C  -1.08043 -4.49574 -2.09237
C  -0.31191 -5.08682 -0.89554
C  -1.16398 -5.02771  0.38548
C  -1.63065 -3.58896  0.68098
C   4.45134  0.01029  0.36195
O   5.07220  1.05819  0.41621
O   5.00264 -1.18529  0.01343
C   6.39776 -1.11075 -0.36200
O   3.44026 -1.29025  2.72756
C   3.36839 -2.43400  3.61120
H  -1.05965  2.11459  1.22665
H  -2.08745  1.79571  2.64196
H  -3.03397  4.13291  2.51410
H  -1.26798  4.18293  2.62359
H  -1.07553  4.52477  0.16186
H  -2.13811  5.76902  0.85190
H  -3.10832  4.77498 -1.27050
H  -4.16197  4.51736  0.12528
H  -3.97281  2.39829 -1.20732
H  -2.19612  2.46135 -1.10013
H  -4.44250  1.78295  2.53559
H  -5.17702  2.73699  1.19326
H  -4.14039 -3.46523 -1.75919
H  -3.95919 -4.53702 -0.31948
H  -2.28322 -3.55714  1.57307
H  -0.75195 -2.95193  0.89404
H  -0.59251 -5.40891  1.25011
H  -2.04000 -5.69914  0.27562
H   0.61804 -4.50837 -0.73544
H  -0.01016 -6.12796 -1.10981
H  -1.95804 -5.13521 -2.31910
H  -0.45275 -4.50513 -3.00138
H  -0.66099 -2.39072 -1.67466
H  -2.12077 -2.64026 -2.65361
H   0.52364  1.97332 -2.62496
H   2.92946  3.86528  1.33774
H   2.28689  5.35001 -0.60086
H   3.27295  1.46088  2.15384
H   1.61938  0.77806  2.15376
H   1.64885 -1.30853 -0.62872
H   2.32285 -2.64636  3.88608
H   3.79780 -3.32477  3.12566
H   3.95287 -2.15447  4.49816
H   6.51925 -0.47703 -1.25484
H   6.99953 -0.68946  0.45910
H   6.69902 -2.14507 -0.57653
C   2.37157 -0.75055 -2.92771
H   3.07566 -0.56427 -3.75270
H   2.23469 -1.84403 -2.79703
H   1.38813 -0.29935 -3.16811
```

VI^{OMe}

```
81
BP86
SCF  =           -2068.70943351
H(0 K)=          -2068.059633
H(413 K)=        -2067.981969
G(413 K)=        -2068.190391
B97D  (1,2-C6Cl2H4)  =  -2068.36174101
Low  Freq.  =  10.5031cm-1, 18.4107cm-1

81
```

VI^{OMe}

```
C   1.68063  2.03539  0.98186
C   1.45348  1.46074 -0.30733
C   0.55697  2.08203 -1.23342
C  -0.07102  3.28157 -0.78605
C   0.15832  3.85454  0.46061
C   1.05054  3.21822  1.35987
Pd -0.54971  0.43580 -0.28295
C   2.51130  0.40812 -0.63186
O   3.05664  0.65619 -1.92355
F  -0.90566  3.92037 -1.65802
C   2.75558  1.26497  1.71571
C   3.54950  0.54609  0.56992
C   4.08680 -0.81412  1.02383
O   3.81068 -1.90572  0.54136
C  -2.15553 -0.75305 -0.00046
N  -3.43628 -0.37510  0.34804
C  -4.34279 -1.43336  0.42858
C  -3.61654 -2.56043  0.16368
N  -2.31954 -2.11814 -0.10822
C  -4.15543  0.91579  0.46504
C  -5.48357  0.37055  1.09368
O  -5.62014 -1.03050  0.68189
C  -4.36370  1.52392 -0.94552
C  -5.02514  2.91463 -0.89643
C  -4.23786  3.87684  0.01194
C  -4.08167  3.28668  1.42625
C  -3.41042  1.89962  1.38704
O  -3.74069 -3.91778  0.12708
C  -2.51830 -4.37457 -0.53843
C  -1.42036 -3.27744 -0.31018
C  -0.51057 -3.08193 -1.53770
C   0.48731 -4.24415 -1.70938
C   1.33128 -4.45877 -0.43873
C   0.42919 -4.67783  0.79015
C  -0.57835 -3.52485  0.96663
C   4.70992  1.45431  0.11837
```

O 4.77488 2.65875 0.30184
O 5.66516 0.73440 -0.53527
C 6.75993 1.52861 -1.05338
O 4.91286 -0.65807 2.09589
C 5.47152 -1.88920 2.61424
H -2.37494 1.98544 0.99629
H -3.33026 1.46295 2.39962
H -5.07936 3.21452 1.90647
H -3.48268 3.96061 2.06430
H -3.23669 4.04941 -0.42628
H -4.74215 4.85907 0.05947
H -5.10030 3.31902 -1.92163
H -6.06655 2.82780 -0.52442
H -4.95738 0.82162 -1.55965
H -3.36266 1.59931 -1.41067
H -5.43428 0.40615 2.19899
H -6.38400 0.89706 0.74533
H -2.73053 -4.49187 -1.61863
H -2.26118 -5.35142 -0.10377
H -1.25675 -3.71521 1.81878
H -0.04076 -2.58222 1.18239
H 1.03818 -4.77413 1.70678
H -0.11316 -5.63937 0.68240
H 1.97466 -3.57512 -0.26815
H 2.00901 -5.32144 -0.57243
H -0.06050 -5.17807 -1.95199
H 1.13956 -4.03979 -2.57737
H 0.02841 -2.12323 -1.39060
H -1.13684 -2.95315 -2.43957
H 0.57110 1.84614 -2.30204
H 1.26488 3.68079 2.32898
H -0.33459 4.79619 0.71644
H 3.41850 1.91073 2.31202
H 2.31603 0.50984 2.39296
H 2.09767 -0.61925 -0.59530
H 4.67108 -2.56633 2.95257
H 6.06550 -2.39940 1.83940
H 6.10788 -1.58863 3.45739
H 6.38802 2.25322 -1.79466
H 7.26016 2.07511 -0.23839
H 7.44654 0.81022 -1.52115
C 3.55851 -0.51671 -2.57388
H 3.88549 -0.19691 -3.57573
H 4.41325 -0.95857 -2.03163
H 2.76569 -1.28406 -2.67961

TS(IV^{OMe}-VI^{OMe}).OPiv
97
BP86
SCF = -2415.14760188
H(0 K)= -2414.370399
H(413 K)= -2414.276031
G(413 K)= -2414.520990
B97D (1,2-C6Cl2H4) = -2414.77866420
Low Freq. = -337.0721cm-1, 11.7153cm-1

97
TS(IV^{OMe}-VI^{OMe}).OPiv
C -2.84567 1.61920 1.77054
C -3.55959 1.39246 0.42591
C -3.40737 2.62272 -0.50427
C -3.85816 3.93134 0.17501
C -3.13198 4.15061 1.51557
C -3.29151 2.93230 2.44591
C -5.05555 0.96266 0.59457
O -5.37032 0.05286 -0.50919
C -4.16250 -0.51436 -0.81632
N -3.07506 0.17455 -0.26082
C -1.83735 -0.34084 -0.60647
N -2.23446 -1.39980 -1.40495
C -3.62293 -1.53859 -1.53220
O -3.98705 -2.63463 -2.26791
C -2.74317 -3.04450 -2.92422
C -1.56340 -2.57062 -2.01208
C -1.20962 -3.61098 -0.91822
C -0.43509 -4.81542 -1.49114
C 0.81361 -4.37024 -2.27457
C 0.44492 -3.36748 -3.38319
C -0.31507 -2.15740 -2.80817
Pd 0.02416 0.16831 0.04551
O 0.21542 -1.76088 1.49953
C 0.10363 -1.39041 2.72949
C -0.04399 -2.53064 3.81154
C 1.06931 -2.33628 4.86514
C 0.82410 1.89233 -0.72533
C 0.24378 3.10182 -0.23473
C 0.20761 4.23175 -1.04916
C 0.78914 4.28488 -2.32269
C 1.49339 3.13986 -2.74964
C 1.52478 1.97774 -1.97113
C 2.41990 0.79636 -2.22502
C 3.07167 0.45466 -0.83758
C 3.29649 -1.07198 -0.73207
O 3.51528 -1.47409 0.54755
C 3.48820 -2.89947 0.77783
F -0.43988 5.35594 -0.57589
C 2.11718 0.94148 0.32721
C 4.42077 1.20298 -0.72808
O 5.15629 0.78557 0.34698
C 6.38248 1.51994 0.53224
O 4.81695 2.06648 -1.49683
O 0.08441 -0.20497 3.16189
C -1.41967 -2.33783 4.49186
C 0.04197 -3.93958 3.19952
O 3.26262 -1.84502 -1.68662
H -1.75525 1.62317 1.57575
H -3.02118 0.75330 2.43310
H -4.35429 2.85223 2.75914
H -2.70864 3.07565 3.37343
H -2.06218 4.33061 1.30675
H -3.51422 5.06237 2.01248
H -3.66414 4.77921 -0.50601
H -4.95491 3.91435 0.34712
H -3.97784 2.44492 -1.43507
H -2.33990 2.69594 -0.78072
H -5.19494 0.42094 1.55114
H -5.76586 1.80053 0.53408
H -2.68787 -2.55323 -3.91631
H -2.79042 -4.13626 -3.05195
H -2.14494 -3.94602 -0.43080

H -0.61025 -3.09654 -0.14219
H -0.15406 -5.49262 -0.66374
H -1.09118 -5.40991 -2.16213
H 1.53037 -3.87891 -1.59151
H 1.33047 -5.24801 -2.70685
H -0.17135 -3.87491 -4.15569
H 1.35818 -3.01081 -3.89053
H 0.33684 -1.59649 -2.10847
H -0.60609 -1.44777 -3.60478
H -0.18505 3.15138 0.76946
H 2.05736 3.17355 -3.69066
H 0.75023 5.20451 -2.91250
H 1.85383 -0.09165 -2.55140
H 3.19441 1.00365 -2.98064
H 1.96975 0.09533 1.02910
O 2.62059 2.03933 1.08277
H 7.03059 1.43705 -0.35630
H 6.17118 2.58655 0.71341
H 6.86605 1.06688 1.40970
H 2.43930 -3.19480 0.94950
H 3.91208 -3.44147 -0.08169
H 4.08307 -3.06999 1.68714
H -0.10275 -4.71464 3.97801
H -0.72512 -4.07874 2.42053
H 1.02445 -4.11064 2.72762
H -1.55857 -3.05477 5.32441
H -1.49819 -1.31137 4.88485
H -2.24229 -2.49274 3.77017
H 0.94988 -3.04144 5.71060
H 2.06826 -2.50492 4.42291
H 1.03810 -1.30369 5.24796
C 2.85799 1.70607 2.46012
H 3.05711 2.65981 2.97858
H 1.97103 1.21405 2.90505
H 3.73977 1.04286 2.56540

VI^{OMe}.OPiv
97
BP86
SCF = -2415.19052599
H(0 K)= -2414.411086
H(413 K)= -2414.316478
G(413 K)= -2414.561846
B97D (1,2-C6Cl2H4)= -2414.82490879
Low Freq.= 9.0044cm-1, 15.3005cm-1

97
VI^{OMe}.OPiv
C -2.76672 -2.43788 0.65748
C -3.54962 -1.13177 0.86823
C -3.50045 -0.69591 2.35287
C -3.95112 -1.81758 3.30982
C -3.13864 -3.10573 3.08154
C -3.22463 -3.55678 1.61179
C -5.01664 -1.22614 0.31714
O -5.34792 0.05607 -0.30423
C -4.13356 0.57271 -0.66060
N -3.05390 -0.04614 -0.01606
C -1.82291 0.49518 -0.33475
N -2.21143 1.49071 -1.20688
C -3.58802 1.54069 -1.44905
O -3.93807 2.45446 -2.40457
C -2.72728 3.25713 -2.59240
C -1.50476 2.37917 -2.15874
C -0.92595 1.53158 -3.32067
C -0.11095 2.37874 -4.31759
C 0.99061 3.18650 -3.60605
C 0.39248 4.05720 -2.48498
C -0.39958 3.20316 -1.47588
Pd 0.12620 -0.01188 0.27313
O 0.16911 -1.58647 -1.25727
C 0.22495 -2.85051 -0.98412
C 0.40436 -3.80824 -2.22151
C 1.73862 -4.56363 -2.02017
C 0.34973 1.50024 1.59117
C -0.69300 1.90420 2.45965
C -0.48696 2.88887 3.43337
C 0.73945 3.53229 3.59395
C 1.77545 3.16335 2.71682
C 1.60172 2.17661 1.72741
C 2.72623 1.94597 0.73549
C 3.13348 0.46464 0.46558
C 3.42824 0.31151 -1.04599
O 3.70095 -0.97154 -1.41099
C 3.99776 -1.13198 -2.81200
F -1.53332 3.23527 4.25813
C 2.04123 -0.56192 0.89971
C 4.44615 0.15823 1.25453
O 4.85518 -1.13495 1.09307
C 6.00154 -1.48671 1.88630
O 5.06514 0.95418 1.94985
O 0.14699 -3.39237 0.14725
C -0.75696 -4.82823 -2.20286
C 0.41570 -3.05012 -3.56035
O 3.45509 1.23093 -1.86161
H -1.68757 -2.24909 0.81230
H -2.86618 -2.75446 -0.39640
H -4.26683 -3.86707 1.38338
H -2.58477 -4.43935 1.44046
H -2.07804 -2.91378 3.33309
H -3.48997 -3.90779 3.75766
H -3.84865 -1.46818 4.35350
H -5.03002 -2.03409 3.16349
H -4.11009 0.21626 2.49234
H -2.45243 -0.42811 2.57719
H -5.08536 -2.01887 -0.45343
H -5.76755 -1.40827 1.10073
H -2.80020 4.15830 -1.95246
H -2.69928 3.55622 -3.65104
H -1.75413 1.00616 -3.83237
H -0.28482 0.75511 -2.86366
H 0.32659 1.71557 -5.08621
H -0.78358 3.07519 -4.86173
H 1.73183 2.49434 -3.16481
H 1.53298 3.81792 -4.33490
H -0.26496 4.83525 -2.92861
H 1.19326 4.59761 -1.94949
H 0.27257 2.48382 -0.96972
H -0.84334 3.82542 -0.67808

H	-1.69096	1.46386	2.38480
H	2.75014	3.66035	2.80653
H	0.87229	4.29416	4.36803
H	2.42418	2.36425	-0.24008
H	3.62337	2.50434	1.04966
H	2.24525	-1.53933	0.42170
O	2.23732	-0.70900	2.32344
H	6.86515	-0.84194	1.64863
H	5.77570	-1.38424	2.96101
H	6.22087	-2.53558	1.63825
H	3.13779	-0.82689	-3.42940
H	4.87325	-0.52523	-3.09971
H	4.20470	-2.20306	-2.94634
H	0.55102	-3.75280	-4.40625
H	-0.52847	-2.50117	-3.71212
H	1.23030	-2.30903	-3.58812
H	-0.62118	-5.60206	-2.98287
H	-0.80584	-5.31552	-1.21597
H	-1.72628	-4.32999	-2.38883
H	1.90395	-5.30422	-2.82646
H	2.59133	-3.86195	-2.01471
H	1.72866	-5.08463	-1.04919
C	1.35153	-1.66020	2.89269
H	1.67395	-1.81428	3.93819
H	0.30599	-1.28362	2.87893
H	1.36823	-2.62068	2.34028

iPr analogue (i.e. C-H activation of a -CHMe2 group)

III^{CHMe2}
100
BP86
SCF = -2379.85842299
H(0 K)= -2379.040851
H(413 K)= -2378.945126
G(413 K)= -2379.191308
B97D (1,2-C6Cl2H4) = -2379.43607591
Low Freq. = 11.3913cm-1, 17.7061cm-1

100
III^{CHMe2}

C	4.72382	1.20134	-0.54968
C	4.43320	0.15245	0.55509
C	3.94662	0.82622	1.85099
C	4.90857	1.93454	2.32496
C	5.15137	2.98022	1.22129
C	5.67672	2.31098	-0.06182
C	5.63931	-0.81683	0.79086
O	5.50676	-1.90848	-0.17794
C	4.17181	-1.96979	-0.43307
N	3.46591	-0.85907	0.04245
C	2.13650	-0.87052	-0.32042
N	2.03969	-2.05641	-1.00348
C	3.25685	-2.74485	-1.08421
O	3.15953	-3.92838	-1.74902
C	1.89144	-3.82512	-2.47237
C	0.96815	-2.85957	-1.65000
C	0.06924	-1.99525	-2.55307
C	-1.05361	-2.83159	-3.19964
C	-1.89140	-3.56966	-2.13817
C	-1.00292	-4.43635	-1.22608
C	0.13377	-3.61368	-0.58520
Pd	0.85847	0.59305	-0.00427
O	-0.18730	2.48346	0.02288
C	0.55025	2.93968	-0.93337
C	0.24559	4.31536	-1.54199
C	-0.27960	5.26863	-0.44638
C	-0.06706	-0.47125	1.43100
C	0.69424	-1.07200	2.45701
C	0.06625	-1.72788	3.52125
C	-1.32071	-1.84144	3.59348
C	-2.08008	-1.26088	2.56495
C	-1.49487	-0.56401	1.48291
C	-2.33295	0.03032	0.35205
H	-2.31096	-0.66125	-0.51100
F	0.83809	-2.28219	4.49971
O	1.49845	2.20288	-1.39768
C	-3.83998	0.38739	0.60257
C	-4.05431	1.35556	1.83613
C	-5.49664	1.88837	1.96858
C	-4.68303	-0.89444	0.78252
O	-4.54477	-1.72483	-0.29594
C	-5.33543	-2.93410	-0.21871
C	-4.32798	1.10573	-0.67913
O	-5.65966	0.89907	-0.89216
C	-6.20259	1.59349	-2.04040
O	-5.37783	-1.18504	1.74798
C	1.50929	4.89670	-2.20866
C	-0.86009	4.08787	-2.61057
O	-3.64329	1.81466	-1.40383
H	3.75901	1.64630	-0.85482
H	5.14078	0.68607	-1.43466
H	6.68603	1.89390	0.13091
H	5.80426	3.05625	-0.86667
H	4.19950	3.49939	0.99757
H	5.86067	3.75093	1.57264
H	4.49114	2.41128	3.22923
H	5.87727	1.48931	2.63037
H	3.81114	0.06194	2.63766
H	2.94588	1.25728	1.64618
H	6.61881	-0.34969	0.61699
H	5.60278	-1.24663	1.81029
H	1.49034	-4.84395	-2.56483
H	2.09292	-3.40824	-3.47805
H	0.68868	-1.49616	-3.32022
H	-0.36506	-1.19193	-1.92716
H	-1.69486	-2.16980	-3.80801
H	-0.61592	-3.56829	-3.90379
H	-2.43536	-2.82782	-1.52286
H	-2.65740	-4.19694	-2.62999
H	-0.57959	-5.27373	-1.81669
H	-1.60473	-4.90394	-0.42696
H	-0.28772	-2.86323	0.10755
H	0.80271	-4.26257	0.00892
H	1.78604	-1.03720	2.44760
H	-3.16741	-1.35841	2.62698
H	-1.79262	-2.36811	4.42720
H	-1.85149	0.95461	-0.00512

H -3.85438 0.73003 2.72357
C -3.05824 2.52876 1.84039
H -5.10351 -3.49418 -1.13463
H -5.06736 -3.51827 0.67579
H -6.40858 -2.68880 -0.17291
H -6.07381 2.68208 -1.93181
H -5.69764 1.26696 -2.96348
H -7.26823 1.32872 -2.06439
H -1.75600 3.62735 -2.16378
H -1.14584 5.05514 -3.06107
H -0.49696 3.42826 -3.41719
H 2.30491 5.08184 -1.46657
H 1.90914 4.20822 -2.96929
H 1.26794 5.85791 -2.69559
H 0.48678 5.45158 0.32662
H -0.55146 6.24081 -0.89392
H -1.16735 4.84785 0.04972
H -5.59709 2.41643 2.93250
H -5.73750 2.61461 1.17233
H -6.24067 1.07954 1.94374
H -3.26418 3.17578 2.71069
H -2.01092 2.19894 1.90358
H -3.15799 3.14474 0.93036

TS(III^{CHMe2}-IV^{CHMe2})2
100
BP86
SCF = -2379.79564746
H(0 K)= -2378.983591
H(413 K)= -2378.889012
G(413 K)= -2379.130161
B97D (1,2-C6Cl2H4) = -2379.37520335
Low Freq. = -1096.2449cm-1, 15.5711cm-1

100
TS(III^{CHMe2}-IV^{CHMe2})2
C -1.73050 -2.11429 -0.47084
C -0.42289 -1.65085 -0.77941
C 0.43994 -2.50624 -1.49941
C 0.00202 -3.77296 -1.90631
C -1.27731 -4.24757 -1.61132
C -2.13285 -3.40036 -0.88979
Pd 0.09968 0.23251 -0.28389
C -2.09919 0.92986 -1.01606
C -2.57102 2.40664 -1.15786
F 0.86350 -4.57018 -2.60287
C -2.68535 -1.24792 0.31657
C -3.26073 -0.00672 -0.44503
C -4.09178 0.75194 0.63027
O -3.83661 0.77455 1.82576
C 1.95812 -0.38529 0.17481
N 2.41165 -1.01468 1.30689
C 3.79240 -1.24438 1.29743
C 4.24861 -0.73078 0.11803
N 3.11983 -0.23588 -0.54637
C 1.81459 -1.63031 2.52515
C 3.13269 -1.76368 3.36183
O 4.23605 -1.89588 2.40701
C 1.19960 -3.01112 2.18594
C 0.51239 -3.65572 3.40672
C -0.53135 -2.71156 4.03067
C 0.10326 -1.35909 4.40385
C 0.77976 -0.70080 3.18479
O 5.40926 -0.51147 -0.55945
C 4.96982 -0.13784 -1.90650
C 3.55759 0.51869 -1.75456
C 2.64045 0.26330 -2.96352
C 3.03190 1.12106 -4.18363
C 3.07067 2.61900 -3.83024
C 4.02713 2.87805 -2.65219
C 3.65097 2.03001 -1.42061
O 0.72685 2.28537 0.28167
C -0.12864 2.75139 1.09973
O -1.26526 2.16801 1.31094
C 0.15180 4.04428 1.88926
C -4.27629 -0.41959 -1.54132
O -4.54203 0.19773 -2.56277
O -4.91980 -1.58456 -1.20917
C -5.93140 -1.99063 -2.15926
O -5.17537 1.38472 0.09454
C -5.97741 2.11358 1.05238
H 0.01858 -0.45073 2.42016
H 1.26828 0.25030 3.46555
H 0.84577 -1.51102 5.21385
H -0.65919 -0.67059 4.80786
H -1.34867 -2.53957 3.30416
H -0.98977 -3.17862 4.92077
H 0.04322 -4.60561 3.09528
H 1.26648 -3.92483 4.17406
H 1.98958 -3.67062 1.78236
H 0.46124 -2.86083 1.37887
H 3.30336 -0.85520 3.97128
H 3.15535 -2.65448 4.00500
H 4.91135 -1.05457 -2.52450
H 5.72986 0.54230 -2.31583
H 4.38772 2.17093 -0.60822
H 2.67072 2.34901 -1.02466
H 4.02093 3.94620 -2.37130
H 5.06523 2.65299 -2.97047
H 2.05170 2.95377 -3.55446
H 3.37350 3.21339 -4.71088
H 4.02506 0.80679 -4.56384
H 2.31576 0.93155 -5.00242
H 1.60500 0.50239 -2.65115
H 2.65249 -0.81178 -3.21823
H 1.45973 -2.20889 -1.75609
H -3.14420 -3.74259 -0.64320
H -1.58421 -5.24594 -1.93448
H -3.52718 -1.85412 0.68733
H -2.17274 -0.83266 1.20048
H -1.41278 1.37723 0.35265
H -6.81315 2.53113 0.47468
H -6.34439 1.44108 1.84420
H -5.38655 2.91848 1.51843
H -6.36135 -2.91621 -1.75193
H -6.70402 -1.21133 -2.25916
H -5.48413 -2.17229 -3.14953
H -2.92228 2.86255 -0.22003
H -1.73484 3.02316 -1.53378
H -3.39066 2.45630 -1.89682

```
C   0.10633  3.69810  3.39977
C   1.52895  4.62576  1.51799
C  -0.96753  5.06477  1.56342
H   0.26276  4.61322  3.99734
H  -0.86603  3.25944  3.67320
H   0.90027  2.97934  3.66816
H   1.71681  5.54252  2.10369
H   2.33765  3.90760  1.72910
H   1.57953  4.88413  0.44778
H  -0.80672  5.99225  2.14036
H  -0.97073  5.32780  0.49166
H  -1.95690  4.65669  1.82239
C  -1.67911  0.55222 -2.47447
H  -0.74219  1.07560 -2.73892
H  -1.51465 -0.52265 -2.62941
H  -2.45632  0.88355 -3.18626
```

IV^{CHMe2}

```
100
BP86
SCF  =          -2379.82369857
H(0 K)=         -2379.007104
H(413 K)=       -2378.911971
G(413 K)=       -2379.153979
B97D  (1,2-C6Cl2H4)  =          -
2379.40788687
Low Freq. = 13.1477cm-1, 18.6857cm-
1
```

```
100
```

IV^{CHMe2}

```
C  -2.18377 -2.02710 -0.37328
C  -0.82918 -1.71556 -0.67198
C  -0.02872 -2.74295 -1.21981
C  -0.57166 -4.00745 -1.48020
C  -1.90426 -4.32012 -1.20480
C  -2.70081 -3.30919 -0.64349
Pd -0.11883  0.14168 -0.39080
C  -1.94511  0.80631 -1.30994
C  -1.92459  2.33529 -1.53503
F   0.23832 -4.96878 -2.01600
C  -3.04798 -0.99117  0.30193
C  -3.23026  0.37677 -0.47115
C  -3.53422  1.33669  0.68273
O  -2.70617  1.84952  1.44558
C   1.71672 -0.57419  0.31753
N   2.06824 -1.07755  1.54898
C   3.39222 -1.52490  1.62574
C   3.93294 -1.28828  0.39598
N   2.90086 -0.73511 -0.37337
C   1.35674 -1.43630  2.80333
C   2.61625 -1.62824  3.71312
O   3.71020 -2.05875  2.83937
C   0.57007 -2.75807  2.61254
C  -0.23969 -3.14014  3.86772
C  -1.16587 -1.99390  4.31374
C  -0.36852 -0.69531  4.53695
C   0.43065 -0.30339  3.27819
O   5.11838 -1.37199 -0.27168
C   4.74680 -1.18180 -1.67520
C   3.45287 -0.30170 -1.68728
C   2.51271 -0.63226 -2.86069
C   3.03697 -0.08427 -4.20363
C   3.30866  1.42905 -4.13282
C   4.28708  1.75303 -2.98965
C   3.77283  1.21268 -1.64096
O   0.78209  2.23773 -0.13043
C   0.58020  3.06981  0.77510
O  -0.40789  2.98837  1.66101
C   1.44459  4.32798  0.97919
C  -4.43692  0.36867 -1.42976
O  -4.92597  1.36791 -1.94502
O  -4.85521 -0.89667 -1.73113
C  -5.89860 -0.95339 -2.73029
O  -4.85788  1.46329  0.91950
C  -5.21674  2.27140  2.06771
H  -0.26304 -0.08038  2.44279
H   1.02431  0.61244  3.45156
H   0.31994 -0.82955  5.39613
H  -1.04386  0.13439  4.81115
H  -1.92991 -1.81979  3.53107
H  -1.71073 -2.27377  5.23345
H  -0.82364 -4.05351  3.65738
H   0.44434 -3.40022  4.70094
H   1.27551 -3.56340  2.33804
H  -0.11027 -2.62034  1.75303
H   2.90262 -0.67098  4.19108
H   2.49223 -2.40541  4.48041
H   4.54747 -2.17377 -2.12493
H   5.60513 -0.71082 -2.17503
H   4.50882  1.39851 -0.83643
H   2.84054  1.73284 -1.35890
H   4.44606  2.84319 -2.90744
H   5.27947  1.31733 -3.22393
H   2.35366  1.96203 -3.95855
H   3.70612  1.79432 -5.09687
H   3.97054 -0.61121 -4.48779
H   2.30302 -0.31198 -4.99662
H   1.52197 -0.19210 -2.62739
H   2.36485 -1.72496 -2.92108
H   1.02708 -2.58309 -1.44912
H  -3.75088 -3.52093 -0.40981
H  -2.29467 -5.31857 -1.41913
H  -4.04450 -1.40112  0.53717
H  -2.56008 -0.73525  1.26100
H  -1.10606  2.29502  1.41572
H  -6.31457  2.27726  2.08230
H  -4.81480  1.82943  2.99322
H  -4.82295  3.29335  1.95623
H  -6.09989 -2.02253 -2.88292
H  -6.80307 -0.43122 -2.37826
H  -5.56280 -0.48207 -3.66776
H  -1.87666  2.92344 -0.60564
H  -1.04832  2.60944 -2.14744
H  -2.83260  2.65786 -2.08383
C   2.00605  4.31564  2.42461
C   2.59965  4.36641 -0.03590
C   0.53094  5.56817  0.78831
H   2.61006  5.22391  2.59345
H   1.19384  4.29342  3.16753
H   2.65743  3.44045  2.59263
H   3.17990  5.29498  0.10106
```

H	3.28201	3.51269	0.09992
H	2.22502	4.34232	-1.07185
H	1.12158	6.48810	0.94043
H	0.10884	5.60010	-0.23077
H	-0.30199	5.56251	1.50817
C	-1.99274	0.17947	-2.73129
H	-1.03823	0.38112	-3.24820
H	-2.13938	-0.91020	-2.72424
H	-2.79402	0.63578	-3.35133

IV^{Me}.CsOPiv

BP86
SCF = -2360.15370866
H(0 K)= -2359.377194
H(413 K)= -2359.281191
G(413 K)= -2359.530156
B97D (1,2-C6Cl2H4)= -2359.85403798
Low Freq. = 10.240cm-1, 21.128cm-1

97

IV^{Me}.CsOPiv

C	0.67946	-2.88558	0.68746
C	1.27431	-1.60355	0.77986
C	2.38656	-1.47392	1.66385
C	2.83606	-2.58067	2.41461
C	2.23075	-3.84417	2.32024
C	1.16067	-3.96767	1.43359
Pd	0.46446	0.03405	-0.08775
C	2.37839	0.84600	-0.42281
C	3.60975	0.28212	0.33972
C	4.30681	-0.91202	-0.33848
O	3.96056	-1.52115	-1.33963
C	3.15581	-0.17328	1.75955
F	0.54415	-5.18293	1.29642
C	-1.43070	-0.81979	-0.08196
N	-2.31978	-1.18521	0.90810
C	-3.40691	-1.94785	0.45375
C	-3.22614	-2.08567	-0.89361
N	-2.05795	-1.37046	-1.19541
C	-2.47406	-0.93008	2.37156
C	-3.50117	-2.07056	2.67676
O	-4.27589	-2.28909	1.44561
C	-1.15081	-1.08787	3.13976
C	-1.31104	-0.75293	4.63601
C	-1.87809	0.66380	4.83763
C	-3.21886	0.82483	4.09897
C	-3.07622	0.48008	2.60172
O	-3.75870	-2.73157	-1.97047
C	-3.03409	-2.19282	-3.12292
C	-1.66232	-1.63354	-2.60885
C	-0.52580	-2.68335	-2.66877
C	-0.02976	-2.93683	-4.10562
C	0.40596	-1.62719	-4.78534
C	-0.73919	-0.59897	-4.76876
C	-1.23760	-0.33966	-3.33236
O	-0.17932	2.16501	-0.72709
C	-0.48986	3.08501	0.13179
O	-1.36821	2.97359	1.03930
C	0.23423	4.46802	0.00962
C	1.14616	4.61561	1.25448
C	1.07580	4.57759	-1.27402
C	-0.83583	5.58542	0.03692
C	4.60090	1.44524	0.50339
O	4.63120	2.22814	1.44594
O	5.42220	1.56903	-0.58751
C	6.32844	2.69271	-0.52549
O	5.41964	-1.26306	0.38638
C	6.12129	-2.41653	-0.12239
H	-0.43531	0.12913	-2.72873
H	-2.09237	0.36707	-3.35432
H	-1.57570	-0.96624	-5.39854
H	-0.41109	0.35686	-5.21418
H	1.27708	-1.20930	-4.24644
H	0.73092	-1.81879	-5.82354
H	0.80824	-3.65444	-4.07146
H	-0.82491	-3.41986	-4.71028
H	-0.87317	-3.62230	-2.20057
H	0.30750	-2.29518	-2.05379
H	-3.64532	-1.38399	-3.57359
H	-2.92681	-3.01131	-3.84906
H	-2.97565	-3.01073	2.92815
H	-4.22247	-1.81869	3.46668
H	-4.06995	0.52929	2.10858
H	-2.40331	1.22001	2.12346
H	-3.59606	1.85865	4.19708
H	-3.98029	0.17200	4.57212
H	-1.15650	1.40408	4.44279
H	-2.00635	0.88139	5.91325
H	-1.97810	-1.49178	5.12597
H	-0.32890	-0.85195	5.13005
H	-0.41463	-0.40535	2.67521
H	-0.75488	-2.10697	2.99226
H	-0.18105	-3.07442	0.04113
H	3.69034	-2.44967	3.09012
H	2.57909	-4.70453	2.89817
H	2.53966	0.64424	2.17457
H	4.03428	-0.29097	2.41497
H	2.19335	1.83609	0.04150
H	5.47587	-3.30928	-0.08560
H	6.44097	-2.25273	-1.16444
H	6.99348	-2.54422	0.53441
H	5.77154	3.64126	-0.45680
H	6.99106	2.61025	0.35134
H	6.91088	2.65189	-1.45649
H	1.57269	5.56416	-1.32007
H	1.84937	3.79591	-1.31304
H	0.45052	4.46856	-2.17766
H	1.63823	5.60537	1.25242
H	0.55349	4.52001	2.17876
H	1.93822	3.84709	1.27011
H	-0.35921	6.58198	0.05185
H	-1.48113	5.54503	-0.86261
H	-1.47344	5.48166	0.92890
C	2.55000	1.03871	-1.93757
H	3.45649	1.63076	-2.18207
H	2.64669	0.07413	-2.45847
H	1.68005	1.58957	-2.33353
Cs	-3.31474	1.86118	-0.83641

IV^{CO2Me}.CsOPiv

BP86
SCF = -2548.73251115

H(0 K)= -2547.942262
H(413 K)= -2547.840821
G(413 K)= -2548.106534
B97D (1,2-C6Cl2H4) = -2548.38507664
Low Freq. = 8.019cm-1, 16.046cm-1

100
IV^{CO2Me}.CsOPiv

```
C   0.85287  1.56276 -2.74176
C   1.51956  2.50910 -1.72422
C   0.64554  3.76246 -1.46632
C   0.25370  4.47678 -2.77505
C  -0.42379  3.51091 -3.76457
C   0.47419  2.29115 -4.04516
C   2.99317  2.88083 -2.11145
O   3.80373  2.87661 -0.87820
C   3.08944  2.10599 -0.00182
N   1.80001  1.81371 -0.44732
C   1.09121  0.99341  0.39493
N   1.99265  0.83197  1.42495
C   3.22107  1.45892  1.19515
O   4.15481  1.16876  2.15950
C   3.33849  0.68612  3.29235
C   2.03468  0.05459  2.69164
C   2.17366 -1.46003  2.40166
C   2.10913 -2.31998  3.68001
C   0.83350 -2.02389  4.48931
C   0.75367 -0.52645  4.83440
C   0.78703  0.33086  3.55454
Pd -0.67445  0.01683  0.18272
O   0.30142 -1.87421 -0.38916
C   1.35096 -2.12959 -1.07929
C   1.41992 -3.59242 -1.64166
C   0.55290 -3.61156 -2.92686
C  -1.82073  1.62274  0.56197
C  -1.49663  2.58614  1.54449
C  -2.35466  3.66463  1.79286
C  -3.54651  3.84623  1.08938
C  -3.86835  2.89793  0.10454
C  -3.02728  1.80239 -0.17232
C  -3.40099  0.83198 -1.27178
C  -3.60262 -0.64754 -0.78380
C  -5.03240 -0.78176 -0.20210
O  -5.26860 -2.08244  0.15862
C  -6.57688 -2.30979  0.71971
F  -2.00475  4.57365  2.75609
C  -2.47878 -1.14671  0.15876
C  -2.71784 -1.25105  1.61479
O  -2.08681 -1.96516  2.40805
C  -3.59501 -1.52313 -2.06281
O  -4.66238 -1.20183 -2.86053
C  -4.73652 -1.95025 -4.09281
O  -2.76824 -2.36230 -2.38851
O   2.29132 -1.32524 -1.36989
C   2.87304 -3.97065 -1.99648
C   0.84905 -4.59810 -0.62051
O  -5.87356  0.09892 -0.11859
O  -3.72723 -0.41890  2.06959
C  -3.91873 -0.41786  3.49269
H  -0.05471  1.15578 -2.25135
H   1.51070  0.69366 -2.91823
H   1.38901  2.62381 -4.57962
H  -0.03411  1.58184 -4.72219
H  -1.38138  3.16342 -3.33168
H  -0.66941  4.03497 -4.70592
H  -0.41327  5.32441 -2.53857
H   1.15187  4.91904 -3.25430
H   1.18255  4.44948 -0.78597
H  -0.26210  3.42598 -0.93290
H   3.41672  2.12988 -2.80714
H   3.09101  3.88355 -2.55179
H   3.09718  1.55518  3.93169
H   3.95882 -0.02919  3.85149
H   3.12672 -1.64083  1.86393
H   1.35724 -1.74118  1.71213
H   2.15061 -3.38681  3.39610
H   3.00076 -2.13305  4.31561
H  -0.05657 -2.29738  3.89257
H   0.81753 -2.63426  5.41032
H   1.58993 -0.24714  5.50937
H  -0.17652 -0.30622  5.38654
H  -0.09704  0.09231  2.93134
H   0.74155  1.40970  3.79128
H  -0.57846  2.51192  2.13433
H  -4.80525  3.00162 -0.45437
H  -4.19646  4.69702  1.31195
H  -4.31694  1.16009 -1.78839
H  -2.58147  0.80423 -2.01571
H  -2.06606 -2.09872 -0.20113
H  -3.85147 -1.75831 -4.72153
H  -4.79452 -3.03187 -3.88938
H  -5.64950 -1.59997 -4.59425
H  -6.62470 -3.38721  0.93159
H  -6.70472 -1.72757  1.64687
H  -7.36631 -2.01583  0.00830
H   0.84268 -5.61924 -1.04418
H   1.45419 -4.62002  0.30433
H  -0.17754 -4.32078 -0.34095
H   2.91349 -4.97331 -2.45888
H   3.30229 -3.24266 -2.70547
H   3.51063 -4.00442 -1.09059
H  -0.48488 -3.32125 -2.69795
H   0.95132 -2.90828 -3.68013
H   0.54752 -4.62320 -3.37348
H  -4.81797  0.18994  3.66928
H  -4.05404 -1.44221  3.87812
H  -3.05313  0.03729  4.00450
Cs  4.97551 -0.82174 -0.66582
```

Different H^+ Proton transfer Models

Model 1.

carbonate
BP86
SCF = -263.590109859
H(0 K)= -263.576709
H(413 K)= -263.570302
G(413 K)= -263.613280
B97D (1,2-C6Cl2H4) = -263.961554812
Low Freq.=639.019cm-1, 639.012cm-1

4
carbonate
```
C   0.00000  0.00000  0.00000
O   0.00000  1.32676  0.00000
O   1.14900 -0.66338  0.00000
O  -1.14900 -0.66338  0.00000
```

bicarbonate
```
BP86
SCF  =          -264.436236791
H(0 K)=         -264.410973
H(413 K)=       -264.403765
G(413 K)=       -264.448969
B97D (1,2-C6Cl2H4) = -264.505466102
Low Freq.=503.189cm-1, 547.052cm-1
```

5
bicarbonate
```
C   0.16270  0.07486 -0.00001
O  -0.15010  1.30286 -0.00006
O  -1.01546 -0.81350 -0.00018
O   1.25739 -0.52992  0.00008
H  -1.71074 -0.12468  0.00136
```

pivalate
```
BP86
SCF  =          -346.450405704
H(0 K)=         -346.321762
H(413 K)=       -346.305795
G(413 K)=       -346.371792
B97D (1,2-C6Cl2H4) = -346.462362766
Low Freq.=29.285cm-1, 186.277cm-1
```

16
pivalate
```
C   1.07257  0.03111  0.00005
O   1.59816 -1.12516  0.00004
C  -0.53990  0.01722  0.00001
O   1.62302  1.17114  0.00006
C  -1.10916  1.44393  0.00009
C  -0.99851 -0.75455 -1.25346
C  -0.99870 -0.75478  1.25327
H  -2.22105  1.45088  0.00056
H  -0.75061  1.99982  0.88321
H  -0.75141  1.99969 -0.88345
H  -2.09542 -0.93404 -1.26673
H  -0.73762 -0.20141 -2.17609
H  -0.46864 -1.72205 -1.27918
H  -2.09549 -0.93496  1.26592
H  -0.46825 -1.72195  1.27933
H  -0.73871 -0.20142  2.17601
```

Pivalic acid
```
BP86
SCF  =          -347.026668676
H(0 K)=         -346.883975
H(413 K)=       -346.868883
G(413 K)=       -346.931056
B97D (1,2-C6Cl2H4)= -346.947718832
Low Freq.=-16.420cm-1, 211.287cm-1
```

17
Pivalic acid
```
C   1.02161  1.50550 -0.00015
C   0.57482  0.03046 -0.00002
C   1.10377 -0.69414 -1.26428
C  -0.95485 -0.13354 -0.00049
O  -1.63702  1.05245 -0.00012
O  -1.53530 -1.21203 -0.00009
C   1.10291 -0.69363  1.26491
H   2.12434  1.55729  0.00020
H   0.65416  2.04033 -0.89090
H   0.65357  2.04068  0.89015
H   2.20648 -0.68706  1.26621
H   0.75846 -0.19336  2.18647
H   0.75412 -1.73811  1.28711
H   2.20733 -0.68759 -1.26482
H   0.75496 -1.73861 -1.28631
H   0.75996 -0.19423 -2.18627
H  -2.58442  0.78938  0.00001
```

bromide
```
BP86
SCF  =          -13.4997524507
H(0 K)=         -13.499752
H(413 K)=       -13.496483
G(413 K)=       -13.523224
B97D (1,2-C6Cl2H4) = -2575.64827484
```

1
bromide
```
Br  0.00000  0.00000  0.00000
```

Model 2.

Cs2CO3 ion-pair
```
BP86
SCF  =          -304.251137043
H(0 K)=         -304.236665
H(413 K)=       -304.223895
G(413 K)=       -304.292483
B97D (1,2-C6Cl2H4)=  -304.354182753
Low Freq. = 51.976cm-1, 58.964cm-1
```

6
Cs2CO3 ion-pair
```
C   0.00514 -1.19481  0.00000
O  -1.12472 -1.84297  0.00000
O   0.00000  0.14924 -0.00000
O   1.13969 -1.83481  0.00000
Cs -2.82314  0.31009  0.00000
Cs  2.82040  0.33349 -0.00000
```

CsHCO3
```
BP86
SCF  =          -284.654531767
H(0 K)=         -284.627769
H(413 K)=       -284.617628
G(413 K)=       -284.674969
B97D (1,2-C6Cl2H4) = -284.682870496
Low Freq.=66.034cm-1, 155.414cm-1
```

6
CsHCO3
```
C  -0.03154  1.98300  0.00000
O  -0.05639  3.38431  0.00000
O   1.13007  1.44660  0.00000
O  -1.16194  1.41682  0.00000
H   0.89536  3.61060  0.00000
Cs  0.00000 -1.19074  0.00000
```

CsOPiv
```
BP86
SCF  =          -366.667509522
H(0 K)=         -366.536915
H(413 K)=       -366.517859
G(413 K)=       -366.595674
B97D (1,2-C6Cl2H4)=  -366.645369028
Low Freq. = 34.770cm-1, 43.622cm-1
```

17
CsOPiv
```
C  -1.08948  0.03015 -0.00053
O  -0.51795 -1.11504 -0.00044
C  -2.66027  0.01362 -0.00011
O  -0.50037  1.16319 -0.00046
C  -3.23502  1.44055 -0.00290
C  -3.12910 -0.75581 -1.25671
C  -3.12761 -0.75007  1.26068
H  -4.34145  1.41753 -0.00193
H  -2.89601  2.00380  0.88201
H  -2.89766  1.99974 -0.89104
H  -4.22954 -0.86901 -1.26579
H  -2.83868 -0.22250 -2.18041
H  -2.66673 -1.75578 -1.27846
H  -4.22809 -0.86267  1.27190
H  -2.66584 -1.75025  1.28609
H  -2.83548 -0.21282  2.18154
Cs  2.13082 -0.00007  0.00001
```

Model 3.

Cs2CO3.2PhCF3
```
BP86
SCF  =          -1442.85518544
H(0 K)=         -1442.634742
H(413 K)=       -1442.586463
G(413 K)=       -1442.746119
B97D (1,2-C6Cl2H4) =
Low Freq. = 9.163cm-1, 12.124cm-1
```

36
Cs2CO3.2PhCF3
```
C  -0.00036 -0.59361  0.00190
O   0.65585  0.05886  0.93205
O  -0.00025 -1.91485  0.00425
O  -0.65623  0.05520 -0.93110
Cs  2.49542 -1.98875  1.48653
Cs -2.49425 -1.99518 -1.48002
C  -4.52255  2.32073  2.08977
C  -3.72613  2.81350  3.13305
C  -3.90733  1.68355  0.99396
H  -4.19842  3.31811  3.98292
C  -2.32860  2.65572  3.08890
C  -2.50853  1.53520  0.93461
H  -1.71450  3.03439  3.91419
H  -1.97764  1.05053  0.08921
C  -1.72275  2.01737  1.99454
H  -0.64277  1.83889  1.92718
H  -5.61034  2.43415  2.11818
C  -4.75145  1.07585 -0.08765
F  -4.20759  1.21604 -1.33595
F  -4.89525 -0.30109  0.08360
F  -6.01595  1.57441 -0.13809
C   2.32837  2.63905 -3.10111
C   1.72225  2.00694 -2.00329
C   3.72588  2.79682 -3.14567
H   0.64228  1.82857 -1.93528
H   4.19838  3.29657 -3.99829
C   2.50770  1.53109 -0.94024
C   4.52199  2.31030 -2.09925
H   1.97652  1.05096 -0.09238
H   5.60977  2.42377 -2.12792
C   3.90651  1.67942 -0.99993
H   1.71451  3.01281 -3.92881
C   4.75037  1.07828  0.08551
F   4.20604  1.22538  1.33266
F   4.89473 -0.29964 -0.07784
F   6.01473  1.57742  0.13345
```

CsHCO3.PhCF3
```
BP86
SCF  =          -853.949461130
H(0 K)=         -853.819930
H(413 K)=       -853.791841
G(413 K)=       -853.897884
B97D (1,2-C6Cl2H4)=  -853.879604336
Low Freq. = 8.309cm-1, 21.441cm-1
```

21
CsHCO3.PhCF3
```
C  -1.57517  2.32849 -0.07290
O  -1.07250  3.62280 -0.25014
O  -2.57990  2.00501 -0.78230
O  -0.95407  1.62140  0.78146
H  -1.67208  3.99542 -0.92762
Cs -2.41540 -0.77653  0.08876
C   3.86115 -0.66818 -0.21763
C   4.77690  0.39112 -0.14090
C   2.49284 -0.41786 -0.00617
H   5.84267  0.20015 -0.30309
C   4.32451  1.69030  0.14232
C   2.02677  0.88148  0.28101
H   5.04106  2.51679  0.19997
H   0.95705  1.09584  0.45262
C   2.95595  1.92993  0.35143
H   2.59479  2.93971  0.56974
H   4.20253 -1.68316 -0.43817
C   1.50048 -1.54078 -0.10516
F   0.71189 -1.64957  1.01999
F   0.60850 -1.36113 -1.14631
F   2.07425 -2.75781 -0.29806
```

CsOPiv.PhCF3
BP86
SCF =   -935.960531745
H(0 K)=   -935.727193
H(413 K)=   -935.690105
G(413 K)=   -935.816136
B97D (1,2-C6Cl2H4)= -935.843070637
Low Freq. = 8.021cm-1, 19.406cm-1

```
32
CsOPiv.PhCF3
C  -2.29742  2.62918 -0.46239
C  -3.65150  3.00048 -0.40657
C  -4.63263  2.03998 -0.11309
C  -4.26001  0.70877  0.12330
C  -2.90173  0.34749  0.06636
C  -1.90635  1.30264 -0.22669
C  -2.49591 -1.07956  0.30268
F  -3.53073 -1.88566  0.65804
F  -1.92339 -1.65194 -0.81848
F  -1.54309 -1.19926  1.28891
Cs  1.28945 -2.24664 -0.19064
O   1.14641  0.64659 -0.50780
C   2.31300  0.85224 -0.01046
O   3.12469 -0.05464  0.35955
C   2.74361  2.35147  0.16284
H  -5.68871  2.32468 -0.06723
H  -3.94294  4.03993 -0.59147
H  -0.83696  1.03644 -0.27293
H  -1.53023  3.37590 -0.69042
H  -5.01560 -0.04713  0.35324
C   4.22461  2.45666  0.56835
C   2.49648  3.09979 -1.16562
C   1.85221  2.96828  1.26762
H   2.69771  4.18192 -1.05572
H   3.15391  2.71528 -1.96635
H   1.45311  2.95848 -1.48985
H   4.51659  3.51391  0.71380
H   4.41545  1.90452  1.50219
H   4.87787  2.02059 -0.20604
H   2.07794  4.04255  1.40342
H   0.78556  2.86562  1.00701
H   2.01669  2.46441  2.23719
```

Alternative [Pd(IBiox)Ar(CO$_3$)]$^-$ Model

R = Me (8a)

Reactant, III^{Me} (CO$_3$$^{2-}$)

```
BP86
SCF  =          -2257.99645300
H(0 K)=         -2257.323490
H(413 K)=       -2257.240485
G(413 K)=       -2257.456797
B97D      (1,2-C6Cl2H4)=      -
2257.69211775
Low   Freq.   =   17.7096cm-1,
23.6798cm-1
```

85

III^{Me} (CO$_3$$^{2-}$)

```
C   1.93735 -2.07244  1.50280
C   0.89174 -1.47924  0.74550
C   0.37613 -2.23685 -0.35405
C   0.99558 -3.45551 -0.72033
C   2.07019 -3.99881  0.00115
C   2.49882 -3.29488  1.12968
Pd  0.38650  0.39583  1.35963
C  -2.16986 -3.98584 -1.38533
C  -2.17358 -2.50654 -0.89654
C  -3.29799 -1.75725 -1.62943
O  -3.17426 -0.81637 -2.40398
C  -0.82205 -1.74231 -1.14990
F   3.54774 -3.80020  1.86125
C   0.86943  1.33438 -0.33438
N   0.05395  2.19960 -1.04622
C   0.69660  2.82025 -2.12301
C   1.96118  2.31787 -2.14346
N   2.04442  1.43096 -1.05901
C  -1.23198  2.90938 -0.78943
C  -1.42892  3.48895 -2.22500
O  -0.08628  3.73352 -2.76856
C  -2.35501  1.95286 -0.35869
C  -3.64600  2.70553  0.02195
C  -3.38003  3.77077  1.10167
C  -2.30538  4.76398  0.62358
C  -1.00082  4.02695  0.26184
O   3.13044  2.41622 -2.84771
C   4.13156  1.91543 -1.90953
C   3.41680  0.86566 -0.98974
C   3.46651 -0.55627 -1.60134
C   4.87516 -1.17948 -1.51192
C   5.42230 -1.15874 -0.07170
C   5.38792  0.26585  0.51439
C   3.97277  0.87602  0.44628
O  -0.20725  2.03007  2.55927
C  -0.47418  1.21201  3.59595
O  -0.19462 -0.07734  3.29297
O  -0.93124  1.58490  4.68281
C  -2.48664 -2.52360  0.62223
O  -2.38520 -3.51150  1.33613
O  -2.88481 -1.29962  1.04140
C  -3.08549 -1.15447  2.48083
O  -4.51874 -2.31755 -1.34895
C  -5.63391 -1.66476 -1.98789
H   3.27349  0.30000  1.07915
H   3.96507  1.91157  0.83277
H   6.10475  0.90929 -0.03823
H   5.72976  0.25881  1.56513
H   4.81310 -1.82936  0.56060
H   6.45536 -1.55416 -0.05167
H   4.83442 -2.21569 -1.89286
H   5.57831 -0.63459 -2.17619
H   3.12587 -0.50763 -2.65242
H   2.74611 -1.18566 -1.05288
H   4.50989  2.76390 -1.30463
H   4.95112  1.48988 -2.50683
H  -1.94390  2.75663 -2.87662
H  -1.96172  4.45085 -2.24117
H  -0.24230  4.73380 -0.12494
H  -0.59837  3.54317  1.17159
H  -2.09000  5.50999  1.40999
H  -2.69243  5.33538 -0.24746
H  -3.01731  3.27322  2.02034
H  -4.31526  4.30306  1.35933
H  -4.08730  3.19067 -0.87471
H  -4.39396  1.97237  0.37449
H  -1.98186  1.39211  0.51984
H  -2.54655  1.21571 -1.15771
H   2.32469 -1.56959  2.39567
H   0.64474 -3.99211 -1.61072
H   2.55329 -4.93657 -0.28800
H  -0.98769 -0.68426 -0.89666
H  -0.62895 -1.78632 -2.23777
H  -3.12158 -4.44457 -1.06962
H  -1.37943 -4.50845 -0.82311
H  -5.71072 -0.61497 -1.66027
H  -5.52512 -1.68143 -3.08513
H  -6.52394 -2.23077 -1.67799
H  -3.51916 -2.07941  2.89007
H  -2.11239 -0.92237  2.95219
H  -3.77706 -0.30731  2.59205
C  -1.98273 -4.17389 -2.89815
H  -1.02094 -3.76773 -3.25545
H  -2.00418 -5.24739 -3.15476
H  -2.78199 -3.67911 -3.47840
```

TS(III^{Me}-IV^{Me})2 (CO$_3$$^{2-}$)

```
BP86
SCF  =          -2257.93964512
H(0 K)=         -2257.272821
H(413 K)=       -2257.190054
G(413 K)=       -2257.407729
B97D      (1,2-C6Cl2H4)=      -
2257.63634117
Low   Freq.   =   -980.6525cm-1,
12.7736cm-1
```

85

TS(III^{Me}-IV^{Me})2 (CO$_3$$^{2-}$)

```
C   0.92214  2.76999 -1.73111
C   1.84632  2.87127 -0.49934
C   1.10342  3.46674  0.72140
```

C 0.40466 4.79932 0.38551
C -0.52745 4.65706 -0.83231
C 0.23991 4.11339 -2.05167
C 3.18615 3.62753 -0.82223
O 4.27668 2.92903 -0.13397
C 3.81666 1.65083 0.00715
N 2.42870 1.55271 -0.15548
C 1.94987 0.28037 0.01123
N 3.09984 -0.41384 0.30266
C 4.24940 0.38587 0.27912
O 5.39903 -0.33350 0.45574
C 4.93163 -1.66445 0.86218
C 3.49640 -1.84375 0.26897
C 3.52150 -2.33152 -1.20363
C 3.81042 -3.84082 -1.31347
C 2.82065 -4.66306 -0.46848
C 2.86296 -4.21517 1.00423
C 2.56863 -2.70869 1.14079
Pd 0.11789 -0.39458 -0.22948
O 0.70492 -1.14302 -2.08874
C -0.03875 -0.57623 -3.08879
O 0.32106 -0.70459 -4.27421
C -0.50373 0.21205 1.61303
C 0.28903 0.07559 2.77852
C -0.22378 0.39445 4.04047
C -1.52471 0.86939 4.21901
C -2.31916 1.01899 3.06980
C -1.83569 0.70360 1.78251
C -2.74192 0.91924 0.58653
C -3.26896 -0.39080 -0.09976
C -4.13911 -1.15803 0.91753
O -5.27936 -0.44720 1.22261
C -6.12361 -1.08214 2.20235
F 0.58053 0.24359 5.14307
C -2.07247 -1.26802 -0.57337
C -4.16756 0.06584 -1.26936
O -4.89142 -0.98202 -1.77563
C -5.69418 -0.63772 -2.92345
O -4.26065 1.20249 -1.70734
O -1.12001 0.08736 -2.68275
O -3.88019 -2.22762 1.44828
H 0.15306 1.99457 -1.54346
H 1.50251 2.40117 -2.59551
H 0.99279 4.86389 -2.37472
H -0.44338 3.96636 -2.90566
H -1.34815 3.95733 -0.58432
H -0.99795 5.62916 -1.07119
H -0.15715 5.14818 1.27114
H 1.16197 5.58337 0.17406
H 1.81685 3.59231 1.55745
H 0.35475 2.72166 1.04667
H 3.38820 3.60406 -1.91017
H 3.19658 4.66798 -0.46436
H 4.90810 -1.70469 1.96935
H 5.65940 -2.39285 0.47532
H 4.28305 -1.74859 -1.75520
H 2.53967 -2.08648 -1.66131
H 3.75471 -4.13908 -2.37563
H 4.84615 -4.06906 -0.98132
H 1.79857 -4.51218 -0.86446
H 3.04395 -5.74360 -0.54776
H 3.86132 -4.44910 1.43028
H 2.12977 -4.78186 1.60637
H 1.53117 -2.49353 0.81215
H 2.64051 -2.38329 2.19532
H 1.32105 -0.28589 2.71759
H -3.34356 1.40021 3.17525
H -1.89236 1.11569 5.21949
H -3.61237 1.53408 0.87086
H -2.20450 1.46186 -0.21043
H -1.65449 -1.68105 0.36796
C -2.37990 -2.48940 -1.46061
H -1.47276 -0.51372 -1.47975
H -6.42967 0.14372 -2.66805
H -5.05623 -0.26846 -3.74249
H -6.20218 -1.56778 -3.21688
H -5.58307 -1.22247 3.15307
H -6.97964 -0.40531 2.34013
H -6.46442 -2.06907 1.84680
H -2.73007 -2.18775 -2.45961
H -1.44284 -3.05258 -1.60516
H -3.12989 -3.15888 -1.00213

R = H (8b)

Reactant, III^H (CO_3^{2-})

BP86
SCF = -2218.68187699
H(0 K)= -2218.037220
H(413 K)= -2217.956235
G(413 K)= -2218.170454
B97D (1,2-C6Cl2H4)= -2218.39137003
Low Freq. = 14.4080cm-1, 21.7301cm-1

82
Reactant, III^H (CO_3^{2-})
C -0.99773 -2.53916 -1.66352
C -0.24536 -1.58034 -0.93313
C 0.69323 -2.08873 0.01910
C 0.78079 -3.47904 0.26247
C -0.00940 -4.40944 -0.43005
C -0.87687 -3.90613 -1.40393
Pd -0.62477 0.36568 -1.39607
C 3.06961 -0.58577 -1.27747
C 3.07445 -1.06796 0.19171
C 3.79056 -2.42967 0.27125
O 4.21432 -3.08961 -0.66294
C 1.62340 -1.16179 0.78267
F -1.66907 -4.79009 -2.09813
C -1.31208 0.92076 0.39681
N -0.89206 2.03226 1.11286
C -1.61120 2.24513 2.29427
C -2.50335 1.22105 2.37880
N -2.30795 0.43982 1.22940
C -0.11359 3.26713 0.80676
C -0.02253 3.81071 2.26635
O -1.25198 3.38756 2.94990
C 1.25741 2.96292 0.18110
C 2.00366 4.24697 -0.23543

C 1.14698 5.10689 -1.18291
C -0.19291 5.46681 -0.51565
C -0.96003 4.19392 -0.10596
O -3.50439 0.74934 3.18425
C -4.28519 -0.09887 2.28759
C -3.29448 -0.67080 1.21435
C -2.65423 -2.00123 1.68239
C -3.65373 -3.17603 1.63352
C -4.30878 -3.31561 0.24586
C -4.96408 -1.99200 -0.19281
C -3.95749 -0.82400 -0.16722
O -0.81428 2.16718 -2.47702
C -0.30979 1.58218 -3.58718
O -0.02274 0.28328 -3.37088
O -0.12906 2.17189 -4.65904
C 3.88333 -0.07052 1.04195
O 3.44382 0.71451 1.87149
O 5.21675 -0.13396 0.72630
C 6.04474 0.80245 1.44601
O 3.90391 -2.84027 1.58133
C 4.56557 -4.11128 1.74045
H -3.15267 -0.99151 -0.90573
H -4.44401 0.12901 -0.44469
H -5.82293 -1.76702 0.47435
H -5.38358 -2.08790 -1.21058
H -3.54007 -3.60543 -0.49314
H -5.06019 -4.12748 0.25966
H -3.12656 -4.10803 1.90565
H -4.44703 -3.03699 2.39779
H -2.25562 -1.86809 2.70541
H -1.79721 -2.21482 1.02224
H -5.06609 0.51962 1.80105
H -4.75813 -0.87548 2.90639
H 0.84404 3.36998 2.79648
H 0.01594 4.90823 2.32457
H -1.90192 4.45026 0.41537
H -1.20379 3.61659 -1.01687
H -0.82248 6.05831 -1.20508
H -0.00467 6.11689 0.36595
H 0.93995 4.53109 -2.10405
H 1.69438 6.02273 -1.47587
H 2.27843 4.84219 0.66152
H 2.95526 3.96551 -0.72201
H 1.06164 2.34872 -0.71950
H 1.86045 2.35570 0.87927
H -1.68883 -2.21666 -2.44999
H 1.47701 -3.84316 1.02937
H 0.04623 -5.48453 -0.23616
H 1.20715 -0.14207 0.76111
H 1.71237 -1.46853 1.83971
H 2.57196 0.39318 -1.35512
H 4.09500 -0.52028 -1.67118
H 2.49290 -1.28840 -1.89569
H 4.00408 -4.91001 1.22808
H 5.58484 -4.07758 1.32094
H 4.59731 -4.29517 2.82407
H 5.73753 1.83961 1.23363
H 5.97499 0.63253 2.53327
H 7.06933 0.62340 1.09033

TS(III^{H}-IV^{H})2 (CO_3^{2-})

BP86
SCF = -2218.63231286
H(0 K)= -2217.992479
H(413 K)= -2217.912638
G(413 K)= -2218.124155
B97D (1,2-C6Cl2H4)= -2218.33938519
Low Freq. = -748.1851cm-1, 11.9388cm-1

82
TS(III^{H}-IV^{H})2 (CO_3^{2-})
C -1.93871 0.68738 1.78608
C -0.58080 0.26160 1.63310
C 0.23281 0.26684 2.79189
C -0.28244 0.65612 4.03330
C -1.60732 1.06549 4.19585
C -2.42349 1.07574 3.05205
Pd 0.06321 -0.49170 -0.15449
C -2.06581 -1.51744 -0.26731
H -2.35330 -2.44400 -0.78869
F 0.54351 0.64214 5.13001
C -2.86026 0.76740 0.58482
C -3.28841 -0.61150 -0.02555
C -3.96559 -0.31046 -1.39022
O -4.22128 0.80295 -1.82529
C 1.83787 0.34583 -0.02704
N 2.21102 1.63430 -0.30369
C 3.58941 1.85316 -0.18262
C 4.12585 0.65178 0.17817
N 3.04454 -0.23135 0.28964
C 1.52052 2.87525 -0.72560
C 2.78873 3.69384 -1.16122
O 3.94285 3.14754 -0.44040
C 0.78146 3.51729 0.47393
C -0.02423 4.76525 0.06101
C -0.98815 4.45740 -1.10006
C -0.23074 3.86448 -2.30272
C 0.56047 2.60489 -1.90242
O 5.33130 0.04379 0.39239
C 4.97695 -1.27760 0.92583
C 3.55561 -1.62339 0.37487
C 2.70893 -2.47825 1.33472
C 3.12641 -3.96174 1.32990
C 3.10906 -4.54286 -0.09598
C 4.02159 -3.72274 -1.02572
C 3.60785 -2.23890 -1.04816
O 0.69582 -1.41799 -1.91699
C -0.14944 -1.17299 -2.96873
O -1.27655 -0.54790 -2.65348
O 0.17973 -1.54236 -4.11304
C -4.29962 -1.30066 0.90559
O -4.11731 -2.31545 1.56185
O -5.48738 -0.60481 0.94535
C -6.47954 -1.18359 1.81431
O -4.20351 -1.46679 -2.06785
C -4.36035 -1.29324 -3.49840
H -0.13696 1.79612 -1.60724
H 1.13259 2.20237 -2.75717
H 0.45383 4.63350 -2.71991
H -0.93417 3.60010 -3.11101

H -1.74870 3.72884 -0.76047
H -1.53408 5.37171 -1.39865
H -0.57708 5.14734 0.93841
H 0.66571 5.57942 -0.24544
H 1.51490 3.76247 1.26481
H 0.10229 2.75187 0.89131
H 2.96741 3.57797 -2.24755
H 2.72777 4.76217 -0.90559
H 4.96799 -1.21754 2.03232
H 5.75714 -1.97805 0.59368
H 4.31253 -1.64753 -1.66285
H 2.60232 -2.12160 -1.50625
H 3.98214 -4.11957 -2.05581
H 5.07527 -3.83470 -0.69013
H 2.07520 -4.51051 -0.48867
H 3.41953 -5.60451 -0.08166
H 4.14406 -4.07333 1.75992
H 2.44760 -4.52935 1.99200
H 1.65526 -2.38118 1.00202
H 2.76067 -2.05321 2.35447
H 1.28356 -0.03820 2.74301
H -3.46697 1.40519 3.14511
H -1.97712 1.36828 5.17987
H -3.76898 1.34585 0.82076
H -2.34835 1.29486 -0.23901
H -1.65502 -1.84338 0.70447
H -1.50406 -0.98695 -1.28710
H -3.37430 -1.00171 -3.90072
H -4.67171 -2.27573 -3.88228
H -5.11864 -0.52534 -3.72101
H -6.11846 -1.21864 2.85585
H -7.36166 -0.53180 1.73277
H -6.72715 -2.21109 1.49893

R = CO2Me (8c)

Reactant, III^{CO2Me} (CO_3^{2-})
BP86
SCF = -2446.55735295
H(0 K)= -2445.870904
H(413 K)= -2445.782495
G(413 K)= -2446.016397
B97D (1,2-C6Cl2H4)= -2446.21671002
Low Freq. = 4.9068cm-1, 15.8959cm-1

88
Reactant, III^{CO2Me} (CO_3^{2-})
C 0.93880 -2.51383 1.89371
C 0.39117 -1.60656 0.94554
C -0.38342 -2.17541 -0.11499
C -0.51359 -3.58061 -0.22889
C 0.07375 -4.46166 0.69063
C 0.78163 -3.89428 1.75504
Pd 0.79067 0.34828 1.35800
C -2.72911 -0.33161 0.54030
C -2.60059 -1.00329 -0.85003
C -3.46157 -2.27841 -0.85899
O -4.28229 -2.58663 -0.00638
C -1.08102 -1.30456 -1.14832
F 1.36472 -4.73110 2.67394
C 1.72928 0.83432 -0.34024
N 1.48803 1.97290 -1.09586
C 2.35152 2.11357 -2.18835
C 3.16106 1.01987 -2.17074
N 2.77233 0.26653 -1.05260
C 0.80502 3.28375 -0.87323
C 0.90903 3.80734 -2.33770
O 2.16437 3.27187 -2.88455
C -0.64094 3.13558 -0.37596
C -1.28653 4.50394 -0.07490
C -0.44697 5.30123 0.94043
C 0.98790 5.49626 0.41693
C 1.65225 4.13950 0.10673
O 4.21134 0.46565 -2.85204
C 4.81954 -0.42083 -1.86393
C 3.67016 -0.90966 -0.91505
C 3.00074 -2.19823 -1.45310
C 3.89897 -3.44007 -1.27445
C 4.36167 -3.60439 0.18562
C 5.05866 -2.32694 0.69100
C 4.14969 -1.09092 0.53729
O 0.92610 2.16753 2.41857
C 0.26801 1.63076 3.47082
O -0.05377 0.34172 3.24223
O -0.00705 2.25011 4.50466
C -3.09665 -0.02865 -1.94369
O -2.52945 1.01143 -2.25036
O -4.22006 -0.47249 -2.57363
C -4.79597 0.48125 -3.48979
O -3.21052 -3.05693 -1.95809
C -4.03080 -4.24053 -2.03288
H 3.25208 -1.19369 1.17370
H 4.66579 -0.17070 0.86707
H 6.00523 -2.17603 0.13019
H 5.34481 -2.43760 1.75251
H 3.48349 -3.81369 0.82369
H 5.03992 -4.47352 0.27653
H 3.34449 -4.33622 -1.60640
H 4.78997 -3.36813 -1.93289
H 2.74219 -2.05083 -2.51839
H 2.05537 -2.34293 -0.90378
H 5.57728 0.14972 -1.28996
H 5.30847 -1.23608 -2.41717
H 0.06826 3.42873 -2.95037
H 0.96960 4.90273 -2.41061
H 2.66352 4.28427 -0.31887
H 1.73986 3.56170 1.04458
H 1.60294 6.03417 1.16152
H 0.96698 6.14050 -0.48832
H -0.40384 4.73699 1.89049
H -0.91844 6.27965 1.15237
H -1.40207 5.09329 -1.00959
H -2.30823 4.33790 0.31140
H -0.58883 2.55372 0.56411
H -1.23406 2.55654 -1.10367
H 1.48674 -2.13510 2.76332
H -1.08244 -3.99316 -1.07071
H -0.01564 -5.54807 0.59855
H -0.58166 -0.32332 -1.19575
H -1.02796 -1.76999 -2.14852

H -1.98487 0.48614 0.60616
H -2.44341 -1.04418 1.32664
H -3.86347 -4.88764 -1.15632
H -5.09900 -3.97044 -2.07194
H -3.72611 -4.75248 -2.95698
H -5.12483 1.37468 -2.93511
H -4.06564 0.77994 -4.25920
H -5.65583 -0.03040 -3.94518
C -4.09448 0.25095 0.83442
O -4.84883 0.77980 0.02027
O -4.36977 0.18881 2.16610
C -5.60716 0.81794 2.55271
H -6.46604 0.31213 2.08032
H -5.65398 0.72268 3.64630
H -5.61698 1.87983 2.25602

TS(III^{CO2Me}-IV^{CO2Me})2 (CO_3^{2-})
BP86
SCF = -2446.51215443
H(0 K)= -2445.831668
H(413 K)= -2445.743693
G(413 K)= -2445.974655
B97D (1,2-C6Cl2H4)= -2446.17408322
Low Freq. = -960.9912cm-1, 13.0121cm-1

88
TS(III^{CO2Me}-IV^{CO2Me})2 (CO_3^{2-})
C -1.48723 -2.44993 -2.18281
C -2.44296 -2.58873 -0.98010
C -1.85256 -3.51552 0.11095
C -1.40749 -4.87691 -0.45947
C -0.43847 -4.70286 -1.64382
C -1.06472 -3.82215 -2.74094
C -3.89130 -3.01446 -1.41631
O -4.84691 -2.27045 -0.58939
C -4.16688 -1.14273 -0.22937
N -2.78177 -1.26524 -0.40323
C -2.08902 -0.14963 -0.01725
N -3.09712 0.67296 0.42059
C -4.37018 0.10803 0.27700
O -5.37418 0.97357 0.60949
C -4.68286 2.09754 1.25365
C -3.22820 2.12902 0.68082
C -3.13727 2.89766 -0.66366
C -3.14052 4.42535 -0.46390
C -2.03287 4.86479 0.51062
C -2.18925 4.14997 1.86528
C -2.17641 2.61887 1.69255
Pd -0.17837 0.24691 -0.16749
O -0.63840 1.31517 -1.89879
C 0.05007 0.74931 -2.92582
O -0.22296 1.04145 -4.10365
C 0.29784 -0.80223 1.51336
C -0.53782 -0.84034 2.65715
C -0.15960 -1.54143 3.80600
C 1.04477 -2.24347 3.88563
C 1.88366 -2.21248 2.76030
C 1.53762 -1.51136 1.58408
C 2.51610 -1.53043 0.41925
C 3.24188 -0.15508 0.16144
C 3.90207 0.34933 1.46378
O 4.61152 -0.64145 2.08469
C 5.27085 -0.21982 3.29361
F -1.00285 -1.54979 4.88850
C 2.22716 0.88241 -0.36814
C 4.38978 -0.39470 -0.85119
O 3.88330 -0.83907 -2.02799
C 4.83940 -0.91949 -3.10041
O 5.58657 -0.27563 -0.62075
O 0.99871 -0.11850 -2.55738
O 3.80921 1.47912 1.92231
H -0.58866 -1.88331 -1.86804
H -1.96456 -1.83139 -2.96345
H -1.93830 -4.34993 -3.17946
H -0.34971 -3.66190 -3.56625
H 0.49140 -4.22142 -1.28495
H -0.15130 -5.68947 -2.05312
H -0.94044 -5.47262 0.34578
H -2.29227 -5.45669 -0.79650
H -2.59576 -3.64820 0.91930
H -0.98545 -2.99339 0.55421
H -4.06665 -2.74996 -2.47661
H -4.09735 -4.08375 -1.25978
H -4.67137 1.92064 2.34729
H -5.26221 3.00427 1.02598
H -3.98233 2.58616 -1.30613
H -2.21121 2.57378 -1.18044
H -3.01132 4.91436 -1.44600
H -4.12312 4.76817 -0.07436
H -1.04787 4.60621 0.07841
H -2.05069 5.96185 0.65090
H -3.13694 4.47337 2.34519
H -1.37717 4.44039 2.55583
H -1.18831 2.29076 1.30993
H -2.32694 2.10673 2.66104
H -1.50151 -0.32081 2.66677
H 2.84153 -2.74569 2.79428
H 1.30961 -2.78999 4.79556
H 3.28413 -2.30148 0.60189
H 1.99935 -1.77829 -0.52404
H 1.60081 1.23364 0.48791
C 2.83630 2.10052 -0.96717
H 1.60202 0.36536 -1.41672
H 4.26161 -1.23869 -3.97838
H 5.28820 0.07236 -3.27082
H 5.63919 -1.64331 -2.86663
H 4.53635 0.12281 4.04128
H 5.81073 -1.10513 3.66029
H 5.97431 0.60302 3.08539
O 2.01261 3.19671 -0.84269
O 3.91300 2.16099 -1.56383
C 2.46563 4.35086 -1.57058
H 2.49704 4.14256 -2.65332
H 1.73052 5.14121 -1.35864
H 3.47230 4.65916 -1.24059

R = C(CH_2)$_2$ (8d)

Reactant, III$^{C(CH2)2}$ (CO_3^{2-})
BP86

SCF = -2296.06126680
H(0 K)= -2295.383214
H(413 K)= -2295.299118
G(413 K)= -2295.518941
B97D (1,2-C6Cl2H4)= -2295.74397654
Low Freq. = 14.2275cm-1, 21.0199cm-1

86
Reactant, $III^{C(CH2)2}$ (CO_3^{2-})

```
C   4.12223 -0.86994  0.57119
C   3.64662 -0.68935 -0.88193
C   3.06390 -2.00622 -1.45248
C   4.04453 -3.18815 -1.29789
C   4.52244 -3.35626  0.15769
C   5.11483 -2.04299  0.70428
C   4.77620 -0.11345 -1.80484
O   4.12704  0.74587 -2.79024
C   3.02914  1.21610 -2.12194
N   2.67984  0.43123 -1.01227
C   1.57951  0.91007 -0.32101
N   1.26143  2.02537 -1.08388
C   2.13519  2.24084 -2.15533
O   1.87380  3.38547 -2.85101
C   0.56214  3.80932 -2.34504
C   0.44283  3.25896 -0.89001
C   1.14621  4.18359  0.13893
C   0.32797  5.45622  0.43457
C  -1.09697  5.09649  0.89342
C  -1.80267  4.23892 -0.17307
C  -1.00514  2.95382 -0.47543
Pd  0.61741  0.35219  1.34284
O  -0.36697  0.28571  3.16225
C  -0.12644  1.58855  3.41602
O  -0.56411  2.19974  4.39935
C   0.29807 -1.58210  0.80360
C   1.00473 -2.56888  1.54144
C   0.90621 -3.92392  1.21756
C   0.11442 -4.38562  0.16281
C  -0.63818 -3.42857 -0.53580
C  -0.58356 -2.05144 -0.22020
C  -1.49492 -1.09524 -0.97048
C  -2.98073 -1.05402 -0.43726
C  -3.76841 -0.08250 -1.33465
O  -5.12210 -0.26068 -1.19334
C  -5.92641  0.64429 -1.97910
F   1.65375 -4.83456  1.92860
O   0.63195  2.14993  2.45113
C  -3.01074 -0.59898  1.03338
C  -3.62427 -2.45146 -0.56677
O  -3.68737 -2.82420 -1.89486
C  -4.27337 -4.12344 -2.10797
O  -4.02253 -3.17015  0.33278
O  -3.29964  0.77136 -2.07499
H   3.22853 -1.04699  1.19550
H   4.57162  0.07527  0.92685
H   6.05319 -1.80704  0.15929
H   5.39892 -2.16071  1.76568
H   3.67050 -3.66642  0.78860
H   5.27336 -4.16647  0.21876
H   3.55194 -4.11249 -1.64934
H   4.92677 -3.04122 -1.95555
H   2.80157 -1.85216 -2.51569
H   2.12836 -2.22483 -0.91151
H   5.48612  0.49666 -1.21071
H   5.32583 -0.88688 -2.36090
H  -0.22249  3.37498 -2.99486
H   0.53483  4.90715 -2.40236
H   2.15495  4.43997 -0.23733
H   1.25166  3.60430  1.07440
H   0.84903  6.04451  1.21183
H   0.27393  6.10942 -0.46305
H  -1.03195  4.51982  1.83480
H  -1.68092  6.01290  1.10193
H  -1.94091  4.83594 -1.09986
H  -2.81612  3.95714  0.16522
H  -0.95135  2.33630  0.44122
H  -1.50386  2.34852 -1.25295
H   1.64429 -2.28062  2.38290
H  -1.28154 -3.76073 -1.36066
H   0.08113 -5.45074 -0.08377
H  -1.10027 -0.07173 -0.87129
H  -1.53669 -1.34560 -2.04491
H  -2.19347 -1.04859  1.60717
C  -3.35190  0.81441  1.46747
C  -4.28052 -0.33526  1.81151
H  -5.30073 -4.16047 -1.70872
H  -4.27480 -4.27355 -3.19744
H  -3.67825 -4.90681 -1.60966
H  -5.72084  0.51427 -3.05445
H  -6.96958  0.38534 -1.74924
H  -5.71746  1.69145 -1.70625
H  -5.23248 -0.39796  1.27630
H  -4.31111 -0.69358  2.84478
H  -3.69408  1.53167  0.71214
H  -2.71922  1.23682  2.25368
```

TS($III^{C(CH2)2}$-$IV^{C(CH2)2}$)2 (CO_3^{2-})
BP86
SCF = -2296.01108913
H(0 K)= -2295.337865
H(0 K)= -2295.338034
H(413 K)= -2295.254715
G(413 K)= -2295.471617
B97D (1,2-C6Cl2H4)= -2295.69228342
Low Freq. = -689.4221cm-1, 20.6191cm-1

86
TS($III^{C(CH2)2}$-$IV^{C(CH2)2}$)2 (CO_3^{2-})

```
C   3.83849 -1.51750 -1.57244
C   3.79375 -1.31340 -0.03509
C   3.09651 -2.48774  0.67313
C   3.67578 -3.85479  0.25833
C   3.64953 -4.03425 -1.27078
C   4.41454 -2.89176 -1.96288
C   5.19564 -0.96667  0.56173
O   5.38757  0.47700  0.38574
C   4.11733  0.98205  0.35156
N   3.13568 -0.01440  0.26465
```

C 1.86599 0.49271 0.10548
N 2.09803 1.84266 0.13997
C 3.45075 2.17035 0.29282
O 3.66792 3.51706 0.34722
C 2.44418 4.10349 -0.20773
C 1.27573 3.06448 -0.02880
C 0.39341 2.96782 -1.29214
C -0.51466 4.20154 -1.45242
C -1.38512 4.42072 -0.20145
C -0.51299 4.54251 1.06296
C 0.42486 3.33006 1.23578
Pd 0.16036 -0.43611 -0.17956
O 0.79789 -0.88783 -2.12073
C -0.00001 -0.39775 -3.12432
O 0.41437 -0.43556 -4.30039
C -0.41145 -0.28318 1.76992
C -1.76275 0.03683 2.10037
C -2.21332 -0.04735 3.43275
C -1.35876 -0.43674 4.47844
C -0.03433 -0.72986 4.14728
C 0.44414 -0.65457 2.83394
C -2.69091 0.53692 1.01868
C -3.16016 -0.51934 -0.04671
C -4.31614 -1.32129 0.56957
O -5.46602 -0.56109 0.58604
C -6.59974 -1.21307 1.18907
F 0.82956 -1.09948 5.14878
C -1.98015 -1.35285 -0.60653
C -3.71683 0.30390 -1.25245
O -4.16739 -0.52177 -2.23054
C -4.26140 0.10300 -3.53693
O -3.71286 1.52461 -1.33716
O -1.17322 0.07437 -2.73369
O -4.29158 -2.44968 1.04405
C -2.26166 -2.68394 -1.31955
H -0.22405 2.05007 -1.23120
H 1.04014 2.82984 -2.17714
H 0.09728 5.10978 -1.64152
H -1.15041 4.05888 -2.34279
H -2.08086 3.56665 -0.10199
H -2.01004 5.32580 -0.31975
H -1.14304 4.64437 1.96545
H 0.08597 5.47573 1.00143
H 1.09508 3.46870 2.10500
H -0.16643 2.41725 1.43114
H 2.61360 4.31161 -1.28139
H 2.26996 5.04826 0.32918
H 5.23081 -1.19630 1.64538
H 6.02714 -1.46766 0.04457
H 4.43910 -0.70300 -2.01936
H 2.80187 -1.40509 -1.95852
H 4.36319 -3.00078 -3.06082
H 5.49069 -2.96148 -1.69369
H 2.59968 -4.02521 -1.61993
H 4.07710 -5.01581 -1.54913
H 4.72048 -3.95111 0.62163
H 3.10107 -4.65653 0.75677
H 2.02432 -2.43389 0.39920
H 3.15394 -2.34913 1.76877
H 1.49737 -0.89417 2.65473
H -3.25608 0.20869 3.66367
H -1.69774 -0.50196 5.51645
H -3.58545 1.01927 1.45002
H -2.17066 1.31027 0.42874
C -1.54154 -2.72630 -0.01436
H -1.44237 -0.66612 -1.51377
H -3.23106 0.33128 -3.86234
H -4.72983 -0.65043 -4.18710
H -4.87461 1.01755 -3.49166
H -6.39491 -1.46724 2.24265
H -7.42637 -0.49132 1.11945
H -6.85106 -2.14211 0.65066
H -2.09769 -3.06665 0.86186
H -0.47071 -2.95602 -0.00536
H -3.30033 -3.02702 -1.32834
H -1.70931 -2.84747 -2.25013

R = Ph (8f)

Reactant, III^{Ph} (CO_3^{2-})

BP86
SCF = -2449.72431572
H(0 K)= -2449.000745
H(413 K)= -2448.910997
G(413 K)= -2449.145740
B97D (1,2-C6Cl2H4)=-2449.34405904
Low Freq. = 9.2457cm-1, 17.0515cm-1

92
Reactant, III^{Ph} (CO_3^{2-})
C 4.44824 -0.69079 0.79284
C 4.03943 -0.61288 -0.68985
C 3.53214 -1.98191 -1.20623
C 4.54220 -3.11642 -0.93249
C 4.94939 -3.17962 0.55232
C 5.47273 -1.81608 1.04311
C 5.19392 -0.05054 -1.59102
O 4.56785 0.73693 -2.64898
C 3.42941 1.21017 -2.05486
N 3.04322 0.46393 -0.93051
C 1.90737 0.95359 -0.30744
N 1.60676 2.03248 -1.12684
C 2.52274 2.21813 -2.16845
O 2.27427 3.32962 -2.92041
C 0.94219 3.76086 -2.47559
C 0.78537 3.27350 -1.00254
C 1.46901 4.23974 0.00186
C 0.65190 5.52939 0.21717
C -0.78863 5.20117 0.65044
C -1.46913 4.29602 -0.39289
C -0.67174 2.99219 -0.60398
Pd 0.90066 0.44304 1.34425
O -0.07788 0.41903 3.16267
C 0.13395 1.73372 3.37483
O -0.25942 2.35666 4.36727
C 0.69293 -1.53758 0.92298
C 1.33472 -2.41711 1.83628
C 1.33326 -3.79894 1.63547
C 0.69888 -4.39579 0.54180
C 0.01222 -3.54606 -0.33845

C -0.02021 -2.14327 -0.15892
C -0.83557 -1.30886 -1.13210
C -2.38677 -1.31985 -0.85993
C -3.06003 -0.34694 -1.84408
O -4.34399 -0.72406 -2.11473
C -5.09544 0.22485 -2.90175
F 2.01753 -4.60265 2.51611
O 0.82714 2.29139 2.35638
C -2.67552 -0.84682 0.61250
C -4.06850 -0.31375 0.89954
C -2.95118 -2.73343 -1.08878
O -2.72701 -3.13948 -2.38198
C -3.22973 -4.45762 -2.67983
O -3.49844 -3.45313 -0.26605
O -2.55959 0.67058 -2.30533
H 3.53145 -0.86615 1.38372
H 4.84585 0.28983 1.11249
H 6.42788 -1.58012 0.52812
H 5.70892 -1.85944 2.12169
H 4.07464 -3.47466 1.15934
H 5.71897 -3.96010 0.70242
H 4.09858 -4.07691 -1.25065
H 5.45212 -2.97740 -1.55317
H 3.31844 -1.90106 -2.28838
H 2.57794 -2.20320 -0.70018
H 5.85821 0.61066 -0.99886
H 5.79026 -0.83455 -2.08019
H 0.18289 3.28823 -3.12854
H 0.90638 4.85456 -2.58344
H 2.48861 4.47421 -0.35883
H 1.54660 3.70201 0.96436
H 1.15506 6.14895 0.98182
H 0.62886 6.14165 -0.70998
H -0.75479 4.66929 1.61937
H -1.37116 6.13027 0.79768
H -1.57484 4.84547 -1.35270
H -2.49595 4.04087 -0.07303
H -0.63332 2.43466 0.35184
H -1.15204 2.34137 -1.35473
H 1.84429 -2.01889 2.72050
H -0.49924 -3.98567 -1.20446
H 0.73609 -5.47968 0.39980
H -0.51441 -0.25727 -1.06380
H -0.68058 -1.64757 -2.17216
H -1.92366 -0.07662 0.85932
H -2.45342 -1.70731 1.26335
H -2.74978 -5.21373 -2.03674
H -4.31987 -4.50415 -2.52116
H -2.98370 -4.63545 -3.73653
H -5.21706 1.16713 -2.34352
H -4.58518 0.43413 -3.85590
H -6.07273 -0.24693 -3.07456
C -5.14822 -1.17167 1.20109
C -6.42364 -0.65401 1.47302
C -6.64817 0.73286 1.45053
C -5.58153 1.59842 1.16029
C -4.30530 1.07782 0.89010
H -4.97204 -2.25138 1.21080
H -7.24665 -1.33832 1.71095
H -7.64328 1.13623 1.67081
H -5.73638 2.68342 1.16088
H -3.46964 1.75769 0.69016

TS(III^{Ph}-IV^{Ph})2 (CO_3^{2-})

BP86
SCF = -2449.67426018
H(0 K)= -2448.956494
H(413 K)= -2448.867252
G(413 K)= -2449.100039
B97D (1,2-C6Cl2H4)= -2449.43791817
Low Freq. = -1113.9990cm-1, 10.2263cm-1

92
DFT_2_pd_nhc_aryl_f_ch2cco2me2_chph_hoco2_ts_ch_964_full
C -2.80197 3.29495 -0.36249
C -3.08624 2.40451 0.87593
C -2.03541 2.61101 1.98168
C -1.83119 4.09715 2.33442
C -1.49078 4.92695 1.08280
C -2.59069 4.77315 0.01690
C -4.56101 2.53463 1.37847
O -5.37159 1.61246 0.57345
C -4.48867 0.64648 0.17827
N -3.15726 0.98399 0.45013
C -2.25582 0.07534 -0.04881
N -3.08288 -0.86341 -0.60488
C -4.44248 -0.54883 -0.47811
O -5.25835 -1.50251 -1.01596
C -4.37379 -2.27199 -1.89705
C -2.90782 -2.13458 -1.34772
C -1.87140 -1.98329 -2.48028
C -1.62047 -3.31545 -3.21160
C -1.19606 -4.42090 -2.22734
C -2.24827 -4.60260 -1.11717
C -2.52518 -3.27851 -0.37703
Pd -0.29520 0.19070 -0.07250
O -0.48095 1.52005 -1.67688
C 0.15330 0.99530 -2.75744
O -0.05617 1.44162 -3.89876
C -0.07027 -1.12792 1.45740
C 1.01829 -2.05378 1.41403
C 1.22340 -2.95583 2.47997
C 0.38311 -2.98046 3.60569
C -0.67422 -2.06891 3.63649
C -0.91190 -1.16554 2.59513
C 1.97756 -2.08427 0.23797
C 2.94197 -0.84662 0.16465
C 3.70495 -0.77226 1.50544
O 4.53447 -1.85989 1.65226
C 5.23475 -1.89902 2.91154
F -1.51445 -2.06915 4.72160
C 2.11118 0.46116 -0.08241
C 2.86123 1.72666 -0.46505
C 3.95033 -1.11892 -0.96654
O 5.04252 -0.30308 -0.84785
C 5.99310 -0.42259 -1.92430
O 3.82771 -1.94188 -1.86132
O 0.99437 -0.01634 -2.48540
O 3.58100 0.07250 2.37931

H -0.92095 -1.61099 -2.05030
H -2.20228 -1.19714 -3.18183
H -2.53387 -3.63900 -3.75490
H -0.84087 -3.15610 -3.97620
H -0.22797 -4.14084 -1.76982
H -1.03298 -5.37575 -2.76119
H -1.92080 -5.36480 -0.38683
H -3.18650 -4.99102 -1.56614
H -3.32769 -3.40449 0.37378
H -1.62008 -2.95865 0.17022
H -4.44298 -1.84923 -2.91753
H -4.74440 -3.30788 -1.89684
H -4.64250 2.23509 2.44212
H -4.98347 3.54043 1.23830
H -3.64426 3.19114 -1.07222
H -1.90554 2.89168 -0.87624
H -2.33097 5.34186 -0.89369
H -3.53337 5.21803 0.40215
H -0.52940 4.57505 0.66352
H -1.35694 5.99118 1.35276
H -2.74806 4.50630 2.80876
H -1.02857 4.18121 3.08922
H -1.08645 2.17810 1.60358
H -2.31768 2.02381 2.87543
H -1.76744 -0.48793 2.68495
H 2.06205 -3.66213 2.42457
H 0.53511 -3.68061 4.43239
H 2.59461 -2.99756 0.27625
H 1.43288 -2.09164 -0.72195
H 1.67272 0.67197 0.91650
H 1.42895 0.18071 -1.21831
H 5.53569 -0.10729 -2.87609
H 6.82326 0.24730 -1.65909
H 6.34369 -1.46327 -2.02513
H 5.86174 -1.00099 3.03944
H 4.52248 -1.95195 3.75145
H 5.85883 -2.80403 2.87546
C 3.19040 2.01802 -1.81055
C 3.86526 3.20099 -2.14773
C 4.22147 4.12934 -1.15483
C 3.89062 3.86093 0.18351
C 3.22043 2.67457 0.52125
H 2.86470 1.32092 -2.59109
H 4.09395 3.40844 -3.19991
H 4.74080 5.05747 -1.42261
H 4.15764 4.57685 0.97087
H 2.98689 2.45517 1.56849

R = OMe (8g)

Reactant, III^{OMe} (CO_3^{2-})
BP86
SCF = -2333.20054331
H(0 K)= -2332.523109
H(413 K)= -2332.438447
G(413 K)= -2332.659004
B97D (1,2-C6Cl2H4)= -2332.88562941
Low Freq. = 17.8857cm-1, 26.2107cm-1

86
Reactant, III^{OMe} (CO_3^{2-})
C 4.15641 -0.02913 0.36652
C 3.57916 -0.04153 -1.06113
C 3.25564 -1.48355 -1.52438
C 4.46653 -2.42786 -1.37045
C 5.03819 -2.40301 0.06037
C 5.37535 -0.96469 0.49798
C 4.51123 0.69821 -2.08292
O 3.64476 1.34977 -3.06169
C 2.50991 1.62099 -2.34693
N 2.39238 0.84407 -1.18438
C 1.25631 1.12467 -0.44601
N 0.67081 2.10373 -1.23301
C 1.42071 2.43653 -2.36646
O 0.88462 3.46235 -3.09022
C -0.45883 3.62788 -2.52084
C -0.38294 3.14060 -1.03972
C 0.14900 4.24510 -0.08892
C -0.91465 5.32149 0.20509
C -2.20001 4.68374 0.76329
C -2.75113 3.63420 -0.21942
C -1.70420 2.54537 -0.52881
Pd 0.61003 0.50403 1.33805
O -0.00128 0.37686 3.31534
C 0.09595 1.71435 3.50100
O -0.20013 2.28983 4.55498
C 0.57077 -1.48111 0.88586
C 1.46507 -2.27717 1.65099
C 1.66121 -3.62890 1.36040
C 0.99829 -4.27236 0.31142
C 0.06314 -3.51531 -0.41215
C -0.18787 -2.15271 -0.12338
C -1.26983 -1.43211 -0.90857
C -2.74398 -1.77392 -0.48345
C -2.97267 -1.48632 1.02045
O -3.03854 -0.15763 1.25641
C -3.12059 0.22459 2.66475
F 2.58053 -4.34243 2.09434
O 0.53600 2.33517 2.38963
C -3.10046 -3.26137 -0.69292
O -2.93111 -3.58108 -2.07816
C -3.70546 -0.90713 -1.31767
O -5.00992 -1.18014 -0.99263
C -5.97674 -0.39515 -1.71867
O -3.40102 -0.08404 -2.16924
O -3.08588 -2.35790 1.87250
H 3.35122 -0.34475 1.05462
H 4.41696 1.00828 0.64523
H 6.21607 -0.58000 -0.11728
H 5.73094 -0.95295 1.54414
H 4.29731 -2.83490 0.75692
H 5.94015 -3.04093 0.12014
H 4.15905 -3.45315 -1.64383
H 5.26634 -2.14508 -2.08669
H 2.91177 -1.45559 -2.57505
H 2.41520 -1.85481 -0.91482
H 5.11169 1.47353 -1.56595
H 5.17678 0.02202 -2.63917
H -1.16626 3.00575 -3.10279
H -0.72078 4.69139 -2.61998

H 1.05534 4.69773 -0.53424
H 0.43473 3.75328 0.86023
H -0.49907 6.04948 0.92526
H -1.15869 5.89861 -0.71267
H -1.96104 4.19138 1.72441
H -2.96168 5.45954 0.96923
H -3.07041 4.13783 -1.15661
H -3.65513 3.15029 0.19246
H -1.46989 1.98428 0.39606
H -2.09676 1.81587 -1.25854
H 2.02370 -1.83536 2.48338
H -0.47865 -3.98590 -1.24283
H 1.20224 -5.32294 0.08471
H -1.13316 -0.34730 -0.78903
H -1.20727 -1.66621 -1.98564
H -4.14830 -3.44006 -0.37955
H -2.44158 -3.88008 -0.05425
H -5.88922 -0.57118 -2.80364
H -6.95945 -0.72320 -1.35076
H -5.83029 0.68022 -1.52535
H -3.82595 -0.43784 3.18944
H -2.10944 0.16787 3.10887
H -3.48030 1.26275 2.65753
C -3.28591 -4.92777 -2.34054
H -4.35149 -5.13281 -2.09531
H -3.12763 -5.10511 -3.41703
H -2.66097 -5.64300 -1.76293

TS(III^{OMe}-IV^{OMe})2 (CO_3^{2-})
BP86
SCF = -2333.13795003
H(0 K)= -2332.466960
H(413 K)= -2332.382321
G(413 K)= -2332.605938
B97D (1,2-C6Cl2H4)=-2332.8288168
Low Freq. = -974.1664cm-1, 7.3638cm-1

86
TS(III^{OMe}-IV^{OMe})2 (CO_3^{2-})
C -1.75117 0.90533 1.80181
C -0.42727 0.39070 1.62769
C 0.39321 0.31773 2.77966
C -0.08395 0.71659 4.03291
C -1.37554 1.21446 4.21530
C -2.19735 1.30310 3.07939
Pd 0.15383 -0.34648 -0.18734
C -2.10468 -1.21167 -0.31409
O -2.57056 -2.35966 -1.03169
F 0.74755 0.62401 5.12142
C -2.68485 1.07080 0.61734
C -3.27605 -0.25362 0.01856
C -4.09146 0.15163 -1.23315
O -4.12638 1.27159 -1.72286
C 2.00039 0.31301 -0.01754
N 2.48812 1.56578 -0.27885
C 3.88049 1.65757 -0.15655
C 4.30655 0.40784 0.18715
N 3.14945 -0.37456 0.29122
C 1.91102 2.86816 -0.68837
C 3.24944 3.58422 -1.09600
O 4.35040 2.91785 -0.39205
C 1.21212 3.54844 0.51393
C 0.51907 4.86672 0.11568
C -0.45075 4.66122 -1.06299
C 0.27305 4.03219 -2.26785
C 0.94873 2.70238 -1.88316
O 5.45252 -0.31213 0.38310
C 4.98219 -1.60552 0.89276
C 3.52854 -1.80773 0.35284
C 2.61693 -2.59294 1.31291
C 2.89582 -4.10839 1.28463
C 2.80947 -4.66762 -0.14741
C 3.77950 -3.92166 -1.08139
C 3.50624 -2.40543 -1.07880
O 0.68905 -1.21090 -2.01143
C -0.14416 -0.73665 -2.99430
O -1.18047 -0.02888 -2.55836
O 0.11675 -0.98173 -4.18678
C -4.20986 -0.90106 1.05805
O -3.96652 -1.87065 1.75887
O -5.38150 -0.18669 1.17139
C -6.29086 -0.70294 2.16218
O -4.80232 -0.89997 -1.72525
C -5.47765 -0.61587 -2.96709
H 0.17657 1.95160 -1.62191
H 1.49810 2.27096 -2.73880
H 1.02449 4.75058 -2.65990
H -0.43779 3.84128 -3.09003
H -1.27166 3.98933 -0.74796
H -0.91698 5.62261 -1.34865
H -0.01189 5.27741 0.99379
H 1.27864 5.62597 -0.16637
H 1.95182 3.71626 1.31917
H 0.46503 2.83503 0.90679
H 3.42188 3.48695 -2.18493
H 3.28125 4.64548 -0.80743
H 4.98879 -1.56945 2.00026
H 5.69114 -2.36803 0.53806
H 4.25589 -1.87395 -1.69474
H 2.51464 -2.18551 -1.52677
H 3.69030 -4.29977 -2.11546
H 4.82278 -4.13377 -0.76301
H 1.77719 -4.53970 -0.52463
H 3.02336 -5.75290 -0.14962
H 3.90385 -4.31815 1.70013
H 2.17582 -4.62055 1.94828
H 1.57268 -2.39192 0.99733
H 2.72102 -2.18866 2.33694
H 1.42061 -0.05599 2.71549
H -3.21460 1.70238 3.18803
H -1.71575 1.52367 5.20792
H -3.53209 1.72572 0.88140
H -2.15583 1.55208 -0.22321
H -1.73146 -1.55383 0.67381
H -1.47382 -0.55202 -1.26779
H -4.74523 -0.33354 -3.74012
H -5.99004 -1.54924 -3.24159
H -6.20348 0.20606 -2.84384
H -5.82386 -0.70569 3.16118
H -7.16232 -0.03214 2.14361
H -6.59255 -1.73505 1.91688

C	-1.64137	-3.43281	-0.95930
H	-2.05709	-4.25413	-1.56736
H	-0.65310	-3.13466	-1.36339
H	-1.51598	-3.79117	0.08851

10. References

1. Melot, R.; Zuccarello, M.; Cavalli, D.; Niggli, N.; Devereux, M.; Bürgi, T.; Baudoin, O., Palladium(0)-Catalyzed Enantioselective Intramolecular Arylation of Enantiotopic Secondary C−H Bonds. *Angew. Chem. Int. Ed.* **2021**, *60*, 7245-7250.

2. Frisch, M. J.; Trucks, G. W.; Schlegel, H. B.; Scuseria, G. E.; Robb, M. A.; Cheeseman, J. R.; Scalmani, G.; Barone, V.; Petersson, G. A.; Nakatsuji, H.; Li, X.; Caricato, M.; Marenich, A. V.; Bloino, J.; Janesko, B. G.; Gomperts, R.; Mennucci, B.; Hratchian, H. P.; Ortiz, J. V.; Izmaylov, A. F.; Sonnenberg, J. L.; Williams-Young, D.; Ding, F.; Lipparini, F.; Egidi, F.; Goings, J.; Peng, B.; Petrone, A.; Henderson, T.; Ranasinghe, D.; Zakrzewski, V. G.; Gao, J.; Rega, N.; Zheng, G.; W. Liang; Hada, M.; Ehara, M.; Toyota, K.; Fukuda, R.; Hasegawa, J.; Ishida, M.; Nakajima, T.; Honda, Y.; Kitao, O.; Nakai, H.; Vreven, T.; Throssell, K.; J. A. Montgomery, J.; Peralta, J. E.; Ogliaro, F.; Bearpark, M. J.; Heyd, J. J.; Brothers, E. N.; Kudin, K. N.; Staroverov, V. N.; Keith, T. A.; Kobayashi, R.; Normand, J.; Raghavachari, K.; Rendell, A. P.; Burant, J. C.; Iyengar, S. S.; Tomasi, J.; Cossi, M.; Millam, J. M.; Klene, M.; Adamo, C.; Cammi, R.; Ochterski, J. W.; Martin, R. L.; Morokuma, K.; Farkas, O.; Foresman, J. B.; Fox, D. J. Gaussian 16, Revision A.03, Gaussian, Inc: Wallingford CT, 2016

3. Becke, A. D., Density-functional exchange-energy approximation with correct asymptotic behavior. *Phys. Rev. A* **1988,** *38*, 3098-3100.
4. Perdew, J. P., Density-functional approximation for the correlation energy of the inhomogeneous electron gas. *Phys. Rev. B* **1986,** *33*, 8822-8824.
5. Andrae, D.; Häußermann, U.; Dolg, M.; Stoll, H.; Preuß, H., Energy-adjusted ab initio pseudopotentials for the second and third row transition elements. *Theor. Chim. Acta* **1990,** *77*, 123-141.
6. Hehre, W. J.; Ditchfield, R.; Pople, J. A., Self-Consistent Molecular Orbital Methods. XII. Further Extensions of Gaussian-Type Basis Sets for Use in Molecular Orbital Studies of Organic Molecules. *J. Chem. Phys,* **1972,** *56*, 2257-2261.
7. Hariharan, P. C.; Pople, J. A., The influence of polarization functions on molecular orbital hydrogenation energies. *Theor. Chim. Acta* **1973,** *28*, 213-222.
8. Höllwarth, A.; Böhme, M.; Dapprich, S.; Ehlers, A. W.; Gobbi, A.; Jonas, V.; Köhler, K. F.; Stegmann, R.; Veldkamp, A.; Frenking, G., A set of d-polarization functions for pseudo-potential basis sets of the main group elements Al-Bi and f-type polarization functions for Zn, Cd, Hg. *Chem. Phys. Lett.* **1993,** *208*, 237-240.
9. Häller, L. J. L.; Page, M. J.; Erhardt, S.; Macgregor, S. A.; Mahon, M. F.; Naser, M. A.; Vélez, A.; Whittlesey, M. K., Experimental and Computational Investigation of C−N Bond

Activation in Ruthenium N-Heterocyclic Carbene Complexes. *J. Am. Chem. Soc.* **2010,** *132*, 18408-18416.

10. Weigend, F.; Ahlrichs, R., Balanced basis sets of split valence, triple zeta valence and quadruple zeta valence quality for H to Rn: Design and assessment of accuracy. *Phys. Chem. Chem. Phys.* **2005,** *7*, 3297-3305.
11. Weigend, F.; Köhn, A.; Hättig, C., Efficient use of the correlation consistent basis sets in resolution of the identity MP2 calculations. *J. Chem. Phys.* **2002,** *116*, 3175-3183.
12. Grimme, S.; Ehrlich, S.; Goerigk, L., Effect of the damping function in dispersion corrected density functional theory. *J. Comput. Chem.* **2011,** *32*, 1456-1465.
13. Tomasi, J.; Mennucci, B.; Cammi, R., Quantum mechanical continuum solvation models. *Chem. Rev.* **2005,** *105*, 2999-3093.
14. E. D. Glendening, J. K. B., A. E. Reed, J. E. Carpenter, J. A. Bohmann, C. M. Morales, C. R. Landis, and F. Weinhold NBO 6.0, Theoretical Chemistry Institute, University of Wisconsin, Madison, WI 2013 http://nbo6.chem.wisc.edu/.
15. Frisch, M. J.; Trucks, G. W.; Schlegel, H. B.; Scuseria, G. E.; Robb, M. A.; Cheeseman, J. R.; Scalmani, G.; Barone, V.; Mennucci, B.; Petersson, G. A.; Nakatsuji, H.; Caricato, M.; Li, X.; Hratchian, H. P.; Izmaylov, A. F.; Bloino, J.; Zheng, G.; Sonnenberg, J. L.; Hada, M.; Ehara, M.; Toyota, K.; Fukuda, R.; Hasegawa, J.; Ishida, M.; Nakajima, T.; Honda, Y.; Kitao, O.; Nakai, H.; Vreven, T.; Montgomery, J. A.; Peralta, J. E.; Ogliaro, F.; Bearpark, M.; Heyd, J. J.; Brothers, E.; Kudin, K. N.; Staroverov, V. N.; Keith, T.; Kobayashi, R.; Normand, J.; Raghavachari, K.; Rendell, A.; Burant, J. C.; Iyengar, S. S.; Tomasi, J.; Cossi, M.; Rega, N.; Millam, J. M.; Klene, M.; Knox, J. E.; Cross, J. B.; Bakken, V.; Adamo, C.; Jaramillo, J.; Gomperts, R.; Stratmann, R. E.; Yazyev, O.; Austin, A. J.; Cammi, R.; Pomelli, C.; Ochterski, J. W.; Martin, R. L.; Morokuma, K.; Zakrzewski, V. G.; Voth, G. A.; Salvador, P.; Dannenberg, J. J.; Dapprich, S.; Daniels, A. D.; Farkas, O.; Foresman, J. B.; Ortiz, J. V.; Cioslowski, J.; Fox, D. J. Gaussian 09, Revision D.01, Gaussian, Inc: Wallingford CT, 2013.
16. Lee, C.; Yang, W.; Parr, R. G., Development of the Colle-Salvetti correlation-energy formula into a functional of the electron density. *Phys. Rev. B* **1988,** *37*, 785-789.
17. Miehlich, B.; Savin, A.; Stoll, H.; Preuss, H., Results obtained with the correlation energy density functionals of becke and Lee, Yang and Parr. *Chem. Phys. Lett.* **1989,** *157*, 200-206.
18. Becke, A. D., Density-functional thermochemistry. III. The role of exact exchange. *J. Chem. Phys.* **1993,** *98*, 5648-5652.
19. Perdew, J. P.; Burke, K.; Ernzerhof, M., Generalized Gradient Approximation Made Simple. *Phys. Rev. Lett.* **1996,** *77*, 3865-3868.
20. Adamo, C.; Barone, V., Toward reliable density functional methods without adjustable parameters: The PBE0 model. *J. Chem. Phys.* **1999,** *110*, 6158-6170.

21. Grimme, S., Semiempirical GGA-type density functional constructed with a long-range dispersion correction. *J. Comput. Chem.* **2006,** *27*, 1787-1799.
22. Zhao, Y.; Truhlar, D. G., The M06 suite of density functionals for main group thermochemistry, thermochemical kinetics, noncovalent interactions, excited states, and transition elements: two new functionals and systematic testing of four M06-class functionals and 12 other functionals. *Theor. Chem. Acc.* **2008,** *120*, 215-241.
23. Chai, J.-D.; Head-Gordon, M., Long-range corrected hybrid density functionals with damped atom–atom dispersion corrections. *Phys. Chem. Chem. Phys.* **2008,** *10*, 6615-6620.
24. Tao, J.; Perdew, J. P.; Staroverov, V. N.; Scuseria, G. E., Climbing the Density Functional Ladder: Nonempirical Meta--Generalized Gradient Approximation Designed for Molecules and Solids. *Phys. Rev. Lett.* **2003,** *91*, 146401.
25. Perdew, J. P.; Burke, K.; Wang, Y., Generalized gradient approximation for the exchange-correlation hole of a many-electron system. *Phys. Rev. B* **1996,** *54*, 16533-16539.
26. Chemcraft - graphical software for visualization of quantum chemistry computations, https://www.chemcraftprog.com.
